# A toolbox approach to revealing a series of naphthocarbazoles to showcase photocatalytic reductive syntheses†

**DOI:** 10.1039/d4sc03438d

**Published:** 2024-07-16

**Authors:** Sharmila Das, Samrat Kundu, Abhisek Metya, Modhu Sudan Maji

**Affiliations:** a Department of Chemistry, Indian Institute of Technology Kharagpur Kharagpur 721302 WB India msm@chem.iitkgp.ac.in

## Abstract

The development of highly reducing photocatalysts to functionalize arenes *via* the generation of reactive aryl radicals under mild and environmentally benign reaction conditions has emerged as a noteworthy approach in the realm of organic synthesis. Herein, we report a readily synthesized series of novel naphthocarbazole derivatives (NCs) as organo-photocatalysts, which, upon irradiation under 390 nm light, acquire high reducing power to catalyze several reductive transformations. The promising properties revealed by in depth photophysical and electrochemical studies (
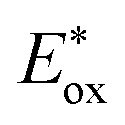
 = −1.9 V to −2.07 V *vs.* SCE, *τ* = 5.59 to 7.12 ns) demonstrate NCs to be versatile catalysts, and notably, rational variation of the substituents (NC1–NC6) modulates their success as efficient photoreductants. Detailed DFT calculations of the frontier MO diagrams and energy levels revealed them to be non-donor–acceptor type molecular scaffolds. The applicability of the NCs as catalysts was demonstrated in reductive dehalogenative borylation, phosphorylation, and dehydrohalide intramolecular C–C coupling reactions, as well as the dimerization of carbonyls and imines. Visible-light-irradiated selective reductive desulfonylation from heteroaromatics and peptides further enhances their synthetic utility.

## Abstract 2

Herein, non-donor acceptor-type naphthocarbazole derivatives (NCs), possessing fine-tuned redox properties, were reported as efficient photoredox catalyst for reductive transformation under mild and environmentally benign reaction conditions.

## Graphical abstract

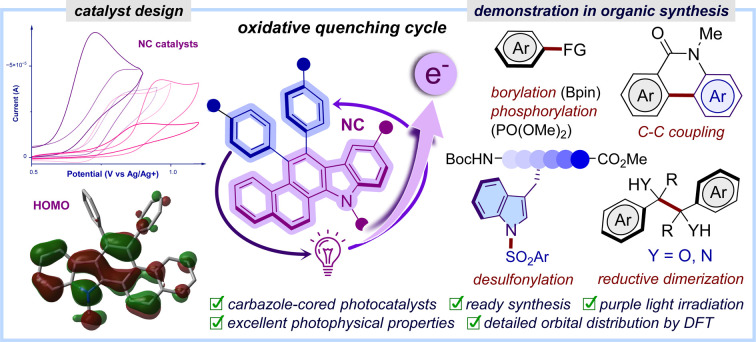

## Introduction

The emergence of photoredox catalysis has not only unveiled previously uncharted mechanistic landscapes, but also ushered in an era of mild and environmentally sustainable reaction conditions.^1^ In the quest for novel catalysts within this realm, notable strides have been taken in the exploration of basic organic molecules as photocatalysts.^2^ These offer distinct benefits over their metal counterparts in terms of accessibility on a large scale, sustainability, cost-effectiveness, and flexibility of scaffold customization for precise tuning of photophysical characteristics and structure–property correlations.^3^ Reductive synthetic transformations, particularly those requiring high reduction potentials, remain underexplored, and the development of stronger reducing photocatalysts has arisen as a valuable chemical toolset in this context. Despite the inherent merits of precious-metal complexes and structurally complex π-conjugated organic dyes, opening avenues to develop very simple, novel, and readily available highly reducing π-electron-rich organic photoredox catalysts holds paramount significance in harnessing novel reactivity and developing unique organic transformations.^4^

**Fig. 1:**
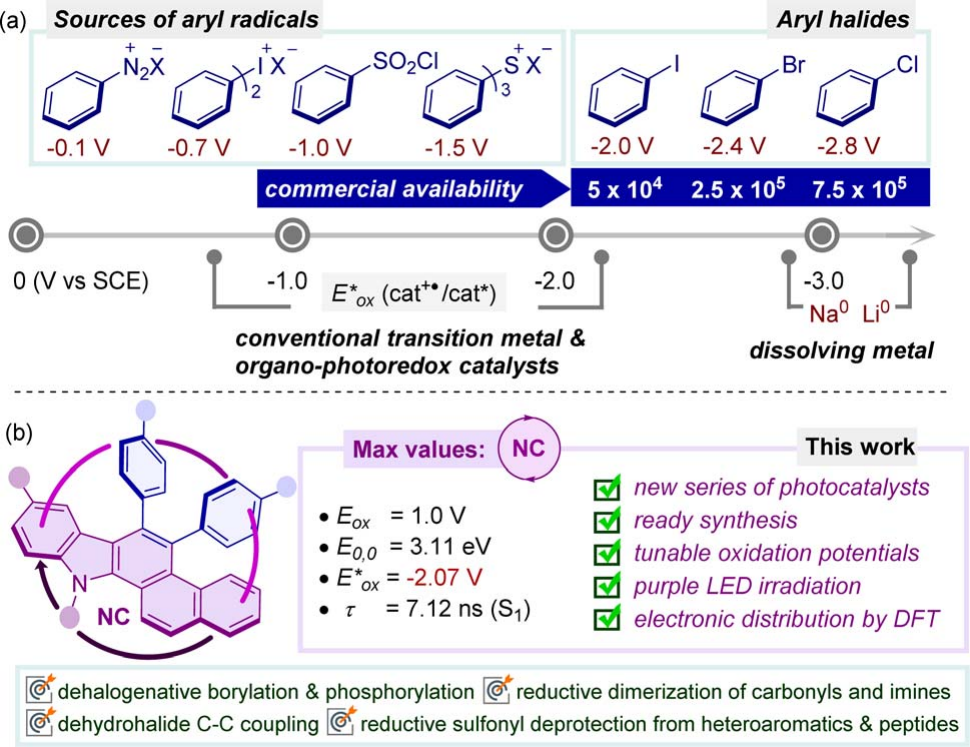
(a) Redox potential of aryl radical precursors and commercial availability of aryl halide sources: SciFinder, accessed April 2021.^4b^ (b) Introduction of naphthocarbazole-based organophotoredox catalysts in reductive synthetic transformations. All potentials *vs.* SCE.

The pathway of arene functionalization *via* the generation of highly reactive aryl radicals through a photoinduced electron transfer (PET) process aided by photoredox catalysts stands as a keystone within the domain of organic synthesis and holds foremost significance across various disciplines.^5^ Early instances of aryl radical precursors involved electron-deficient arenes and weakly coordinated leaving groups,^5^ which required additional effort due to their instability, prompting a shift towards the use of aryl halides as widely recognized and readily accessible sources of aryl synthons in organic synthesis (Fig. 1a).^6^ Activating relatively inert aryl halides, which possess a wide range of redox potentials, presents significant hurdles and often necessitates potent reducing agents such as alkali metals, transition metal complexes and highly reductive organo-photoredox catalysts under a given set of reaction conditions (Fig. 1a).^7^

Continuing our exploration into innovative synthetic approaches for accessing electron-rich nitrogen-containing molecular architectures, we embarked on the synthesis of a series of polycyclic aromatic hydrocarbons based on a carbazole moiety and applying them as catalysts for achieving various organic transformations.^8^ In our quest to introduce simple and readily accessible molecular scaffolds as potent highly reducing organo-photocatalysts, we selected a range of naphthocarbazole derivatives (NCs) considering the possibility to fine-tune the redox and photophysical properties required for visible-light-triggered photocatalytic synthetic transformations (Fig. 1b). Notably, these π-electron-rich carbazole-cored NCs may demonstrate versatility in various oxidative quenching processes, rendering them attractive candidates for unveiling new reactivity pathways and providing facile routes to novel structural frameworks.

## Results and discussion

### Photophysical characterization

**Fig. 2:**
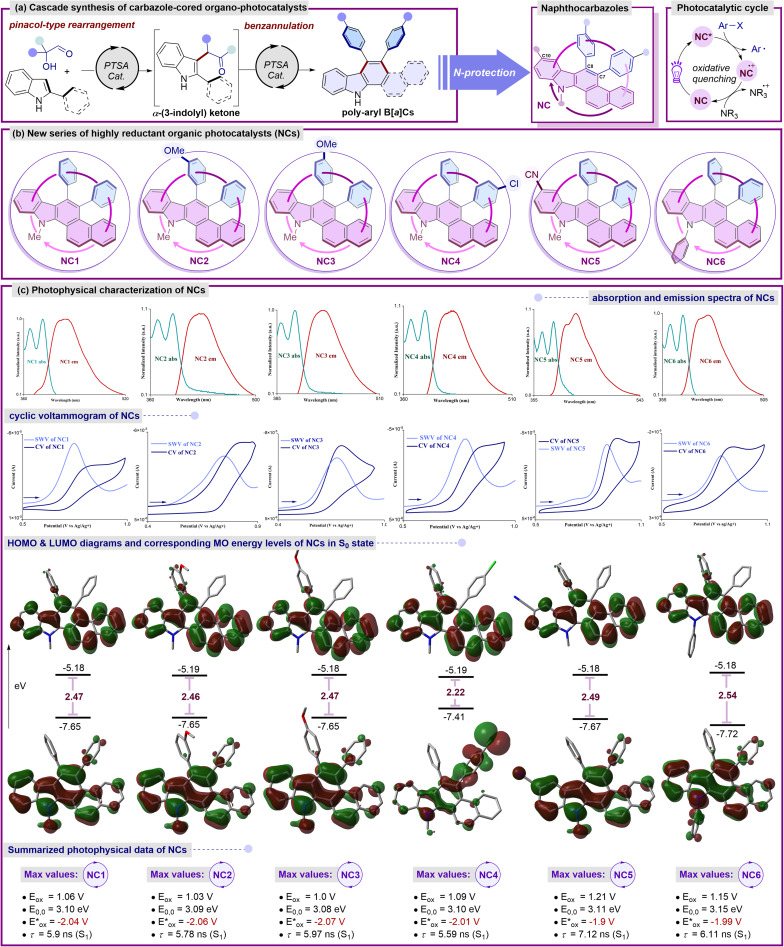
(a) Schematic diagram for synthesis of NCs. (b) Introduction of naphthocarbazole-based organic photocatalysts (NCs). (c) Detailed photophysical characterizations of NCs: normalized UV-Vis absorption (light cyan line) and emission (red line) spectra of NCs measured in DCM (excitation wavelength *λ*_max_ = 350 nm); cyclic voltammogram (CV) of NCs (deep blue line), square wave voltammetry (SWV) of NCs (light blue line) in MeCN/DMF 5 : 1 v/v; selected frontier molecular orbitals (LUMO and HOMO) in S_0_ states of NCs and their DFT-calculated (B3LYP-D3(BJ)/6-311+G**/SMD (*ε* = MeCN)) energy levels (eV); color coding: N: blue, C: grey, O: red. Combined photophysical data of NCs. All potentials *vs.* SCE.

In the pursuit of identifying novel photocatalysts with an optimal scaffold and adjustable redox properties, several essential prerequisites are crucial for understanding and optimizing their performance in various applications. Driven by the preliminary photophysical findings, we anticipated that a deliberate structural reassessment of the first-generation catalyst BPC^8*b*^ could potentially unlock even higher oxidation potentials. With the goal of crafting potent yet readily accessible organo-photocatalysts, we hypothesized that the presence of a basic benzo[*a*]carbazole unit might engender unique photophysical characteristics (Fig. 2). In this line, benzocarbazoles are known to be fascinating highly reducing organo-photoredox catalysts that are readily accessible in gram-scale starting from simple precursors such as α-hydroxy aldehydes and 2-naphthyl indoles (Fig. 2a and b).^9^

At the outset, an *N*-methyl-diphenyl-naphtho[*a*]carbazole (NC1) framework containing unsubstituted phenyl rings was first investigated. DFT calculations of the frontier MOs of NC1 revealed that the benzo[*a*]carbazole moiety was anticipated to house the HOMO of the S_0_ state with minimal involvement from the π-electron cloud of the phenyl ring at the C8 position. Similarly, the entire molecule was fully engaged with the electron density in the LUMO of the S_0_ state with an energy gap of 2.47 eV, excluding any nitrogenic character or π-character from the phenyl ring at the C7 position.^9^ These characteristic features indicate a non-donor–acceptor type molecular regime for the catalytic system. To further evaluate the photophysical characteristics of NC1, a cyclic voltammetry experiment was performed to determine its oxidation potential, which resulted in a value of 1.06 V *vs.* SCE. The first singlet excited state energy (*E*_0,0_ = 3.10 eV) was deduced from the intersection wavelength of the absorption and emission spectra. The excited state oxidation potential was estimated to be −2.04 V *vs.* SCE,^9^ indicating a higher value comparable to selected reported transition-metal-based and organo-photocatalysts.^3*b*,10^ The considerable excited state lifetime (NC1, *τ* = 5.9 ns) strongly suggested its enhanced photocatalytic efficacy in the excited state. Next, we wondered whether specific alterations in the substitution of the framework would induce notable variations in the redox properties while maintaining the reducing nature. Thus, to reassess the electronic properties, a series of naphtho[*a*]carbazole (NC) derivatives were synthesized. In this context, the incorporation of the *m*-anisole moiety at the C8 position of the NCs led to a slight increase in the excited state oxidation potential (−2.06 V *vs.* SCE), accompanied by a minor increase in the LUMO energy and an insignificant decrease in the energy gap to 2.46 eV.^9^ Interestingly, the incorporation of the *p*-anisole moiety resulted in a higher excited state oxidation potential (−2.07 V *vs.* SCE) while maintaining an energy gap comparable to that of NC1.^9^ The alteration in the position of the methoxy unit within the benzene ring at the C8 position of the NCs has a significant influence on the fluorescence lifetimes (NC2, *τ* = 5.78 ns and NC3, *τ* = 5.97 ns), underscoring the ongoing assessment of its catalytic activity in the excited state.^9^ Of note, the introduction of a haloarene (*p*-Cl-C_6_H_4_) at the C7 position (NC4) strongly localizes the HOMO within its vicinity, with minimal contribution from the rest of the molecule. In contrast, the entire molecule was fully involved with the electron density in the LUMO of the S_0_ state. A slight depletion in the excited state oxidation potential (NC4 = −2.01 V *vs.* SCE) and significant decrease in the lifetime (NC4, *τ* = 5.59 ns) were obtained.^9^ To further investigate the probability of potential alterations resulting from structural modifications on the naphthocarbazole core (NCs), an electron-withdrawing –CN group was incorporated at the C10 position. A depleted excited state oxidation potential (NC5 = −1.9 V *vs.* SCE) was noted, along with a marginal lowering of the HOMO and a slight increase in the energy gap to 2.49 eV.^9^ Additionally, the inclusion of an electron-withdrawing substituent exerts a notable impact on the fluorescence lifetime (NC5, *τ* = 7.12 ns), emphasizing the continual evaluation of its catalytic activity in the excited state.^9^ Finally, to assess the influence of extended π-conjugation, a phenyl group was introduced on the nitrogen of the benzo[*a*]carbazole unit (NC6). A comparable excited state oxidation potential (−1.99 V *vs.* SCE) was obtained, accompanied by a significant reduction in the HOMO level and a further increase in the energy gap to 2.54 eV.^9^ Additionally, the incorporation of extended conjugation demonstrates a promising excited state lifetime in NC6 (*τ* = 6.11 ns).^9^

TDDFT calculations for NC1–NC4 showed that the primary transition from S_0_ → S_1_ was contributed by the HOMO–LUMO charge transfer at around *λ*_abs_ = 375 nm with an oscillator strength (*f*) of approximately 0.15.^9^ A slight decrease in charge transfer was observed for NC5 at *λ*_abs_ = 372 nm with a much lower oscillator strength (*f* = 0.08). The protected benzo[*a*]carbazole unit with extended conjugation NC6 was associated with much lower HOMO–LUMO charge transfer at *λ*_abs_ = 368 nm and a comparable oscillator strength of *f* = 0.13. These theoretical outcomes are aligned well with the experimental findings for NC1–NC6. The information, including both photochemical and redox characteristics, is consolidated in Fig. 2c. The noteworthy photophysical traits of this fresh array of naphthocarbazole frameworks (NCs) make them promising candidates for evaluating their catalytic performance under exposure to light. The prospect of formulating a general catalyst system capable of initiating and catalyzing a diverse array of reductive synthetic transformations through the formation of new carbon–carbon or carbon–heteroatom bonds is undeniably captivating.

**Scheme 1:**
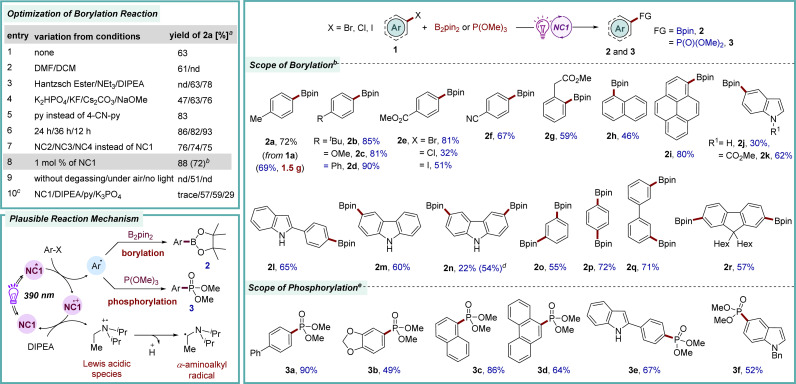
Photocatalytic borylation and phosphorylation reactions of aryl halides. Borylation reaction: ^*a*^1a (0.3 mmol), DIPEA (1.5 equiv.), NC1 (3 mol%), B_2_pin_2_ (2 equiv.), K_3_PO_4_ (2 equiv.), 4-CN-pyridine (10 mol%), DMSO (0.3 mL), under Ar, purple LED (390 nm), rt (∼35–38 °C), 48 h, ^1^H NMR yields using mesitylene as an internal standard. ^*b*^Aryl bromide 1 (0.5 mmol), DIPEA (1.5 equiv.), NC1 (1 mol%), B_2_pin_2_ (2 equiv.), K_3_PO_4_ (2 equiv.), pyridine (10 mol%), DMSO (0.5 mL), under Ar, purple LED (390 nm), rt (∼35–38 °C), 12 h. ^*c*^In the absence of NC1, DIPEA, py or K_3_PO_4_ under the optimized conditions. ^*d*^2m was isolated as the major product in 54% yield. Phosphorylation reaction: ^*e*^1 (0.2 mmol), DIPEA (3 equiv.), NC1 (2 mol%), P(OMe)_3_ (3 equiv.), MeCN (1 mL), under Ar, purple LED (390 nm), rt (∼35–38 °C), 2 h.

To validate naphthocarbazoles NC1–NC6 as organophotoredox catalysts, first, the dehalogenative borylation reaction was selected. Aryl boronic acids and their derivatives are valuable synthetic precursors for a range of key organic transformations such as C–C, C–N, C–O, and C–X bond formation reactions.^11^ In addition, they are also frequently found in pharmaceuticals, agrochemicals, and materials chemistry due to their stability and low toxicity.^12^ Traditionally, C–B bond formation is executed *via* a transition-metal-catalyzed C–B cross-coupling reaction employing aryl halides and alkoxydiborons or by the reaction of highly reactive aryl organometallic reagents with electrophilic borate esters.^13^ Recently, a much milder version of this C–B bond formation reaction was developed under photoredox catalysis by employing a suitable aryl halide and electrophilic alkoxydiboron reagents.^14^ These methods leverage visible light to activate the relatively inert aryl halide bonds, leading to the generation of a highly reactive aryl radical intermediates.^15^ Herein, the newly developed organophotocatalst NCs have been studied in a photocatalytic borylation process employing 4-bromotoluene 1a and B_2_pin_2_ as model precursors (Scheme 1). Through comprehensive evaluation of all NCs, NC1 was found to be a superior catalyst. The best yield for this reaction was recorded when DIPEA was utilized as the reducing agent in DMSO solvent in the presence of pyridine as an additive and K_3_PO_4_ as a base under irradiation with purple LED light (2a, 72% yield).^9^ Consequently, the efficacy of the novel catalytic system was assessed across a broad range of aryl bromides with various electron-donating and -withdrawing substituents at different positions of the benzene ring (2b–2i, 46–90% yields). Additionally, heteroaryl bromides were found to be compatible with this protocol, providing 2j–2m in 30–65% yields. When the suitability of other halides was tested, aryl iodide, along with more-challenging aryl chloride, also furnished the desired product 2e, albeit in lower yield. Notably, the photocatalytic reductive borylation of a protected indole was more efficient than that of the corresponding unprotected one (2j*vs.*2k). This might be due to the quenching of reactive aryl radical species with the acidic proton present on the substrate, leading to the formation of the dehalogenated product. Interestingly, a commendable yield for the borylation of unprotected indole was facilitated by incorporating steric hindrance around the free N–H moiety (2l, 65%). In this line, N–H free carbazole was also a suitable substrate (2m, 60%). Considering the importance of aryl-diborylated products to synthesize conjugated polyarylenes and cross-linkers in materials science, consecutive double borylations were successfully achieved to access 2n–2r in 22–72% yields.^16^

Organophosphorus compounds and phosphinic acid derivatives showcase significant structural diversity and have been demonstrated to have extensive applications in pharmaceuticals, agrochemicals, materials science, and catalysis.^17^ Moreover, the applicability of organophosphonates is further enhanced owing to the increased resistance of their C–P linkage to chemical hydrolysis, thermal decomposition, and photolysis.^18^ Many convenient strategies utilizing transition metal catalysts entail experimental complexity or necessitate prolonged reaction time at elevated temperatures to yield these phosphorus derivatives. In contrast, photocatalytic C–P bond formation reactions have been found to be mild and efficient.^19^ The efficacy of our newly introduced catalytic system was further demonstrated in the reductive phosphorylation reaction of aryl bromides 1 (Scheme 1). Brief optimization revealed that, under irradiation with purple light, NC1 was the most effective catalyst for this reductive C–P bond formation with the employment of P(OMe)_3_ as a phosphorylating agent to afford 3a in 90% yield. Under these standard conditions, various aryl bromides underwent smooth conversion to furnish 3b–3f in 49–86% yields. Thus, the newly developed catalyst is also highly efficient in carrying out the C–P bond formation reactions and could potentially be applied in the synthesis of phosphinates of biological significance.

**Scheme 2:**
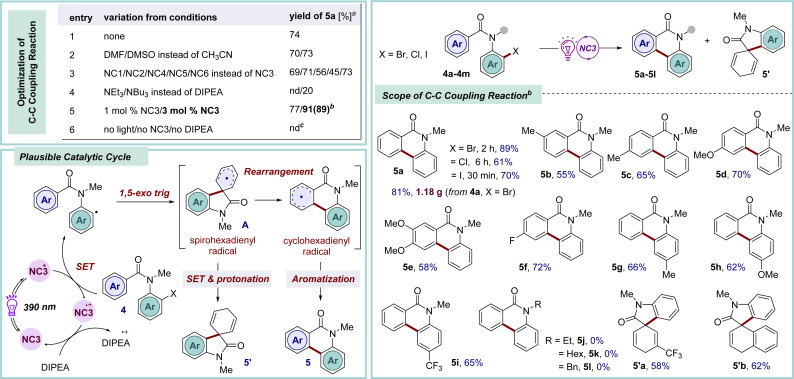
Photocatalytic dehydrohalide C–C coupling reactions. Reaction conditions: ^*a*^4a (0.2 mmol), DIPEA (5 equiv.), NC3 (2 mol%), MeCN (1 mL), under Ar, purple LED (390 nm), rt (∼35–38 °C), 2 h, ^1^H NMR yields using mesitylene as an internal standard. ^*b*^4a (0.5 mmol), DIPEA (5 equiv.), NC3 (3 mol%), MeCN (2.5 mL), under Ar, purple LED (390 nm), rt (∼35–38 °C), 2 h. ^*c*^In the absence of light, NC3, or DIPEA under the optimized conditions.

The homolytic fission of C–X bonds through halogen atom transfer (XAT) stands as the cornerstone method for generating carbon-centered radicals and is utilized in challenging organic transformations, particularly the synthesis of complex molecular archeitectures.^20^ In contemporary organic synthesis, the advent of C–C bond forming reactions, particularly through the selective functionalization of aromatic compounds, has emerged as a highly efficient strategy for the synthesis of natural products and the discovery of novel drug molecules.^21^ Notably, phenanthridinone serves as a crucial foundational unit present in diverse biologically active compounds and natural products, playing a particularly pivotal role in the development of anticancer medicines and the treatment of neurological disorders.^22^ The drawbacks associated with traditional methods to access these structural motifs^23^ can be addressed by photocatalysis in terms of enabling mild reaction conditions, efficiency, and practicality. With our modest interest, herein, a photochemical approach has been showcased for synthesizing phenanthridinones through the dehydrohalide intramolecular C–C coupling pathway by employing the newly designed NCs as photocatalysts (Scheme 2). Following the screening of the NCs in the dehydrohalide C–C coupling reaction, NC3 was found to be the most suitable catalyst to generate aryl radical from 4*via* single electron transfer (SET) under irradiation with purple LED lights (*λ*_max_ = 390 nm).^9^ Subsequently, 1,5-*exo*-trig type intramolecular cyclization yielded spirohexadienyl radical intermediate A. Following this, unstable A rearranged to the stable cyclohexadienyl radical intermediate, leading to the desired aromatized product 5*via* hydrogen abstraction.^23*a*^ Brief optimization of the starting material 4a revealed the optimal conditions, which involved the use of 3 mol% of catalyst NC3 in acetonitrile solvent and 5 equiv. of the base DIPEA, while maintaining a substrate concentration of 0.2 M to afford 5a in 89% yield.^9^ Following optimization, our efforts were redirected to investigating the scope and limitations of this method. Changing the halogen substituent in the parent molecule (X = Cl, 4b; X = I, 4c) yielded the desired phenanthridinone 5a in 61% and 70% yields, respectively. There was a mixed effect on the yield due to the electronic nature of the substituent in the carbonyl-tethered arene moiety (5b–5f, 55–72% yields). Notably, an *m*-methyl substituted arene furnished a mixture of regioisomers 5b in a 1 : 2.8 ratio. Electron-donating and -withdrawing substituents at the para position of the aniline moiety provided the corresponding phenanthridinones (5g–5i) in moderate-to-good yields. Interestingly, changing the protecting group on the amide nitrogen with alkyl or benzyl resulted in a failure to provide the desired cyclized products (5j–5l), and a complex reaction profile was observed. In contrast, the relatively long lifetime of spirohexadienyl radical A intermediate due to electron delocalization aids subsequent SET and protonation, yielding spirocyclic derivative 5′ without rearrangement to 5.^23*a*^

**Scheme 3:**
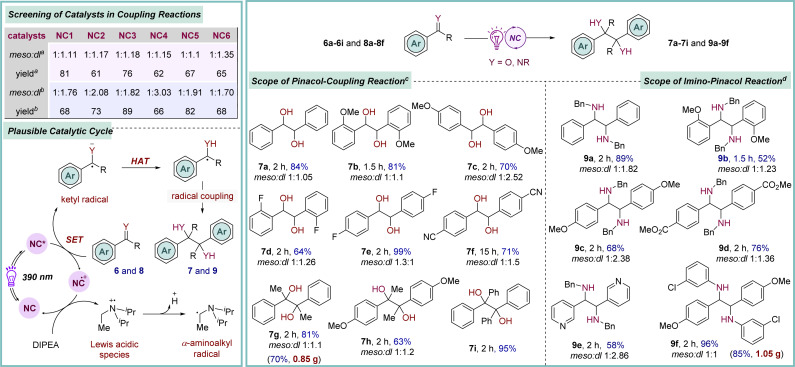
Photocatalytic reductive pinacol and imino-pinacol coupling reactions of carbonyls and imines. ^*a*^Pinacol reaction: 6g (0.5 mmol), DIPEA (1 equiv.), NC-cat. (0.5 mol%), MeCN (2 mL), under Ar, purple LED (390 nm), rt (∼35–38 °C). ^*b*^Imino-pinacol reaction: 8a (0.5 mmol), DIPEA (2 equiv.), NC-cat. (0.5 mol%), MeCN (2 mL), under Ar, purple LED (390 nm), rt (∼35–38 °C). ^*c*^Same as standard conditions for pinacol coupling reaction using carbonyls 6 and NC1 catalyst. ^*d*^Same as standard conditions for imino-pinacol coupling reaction using imine 8 and NC3 catalyst.

The electrophilic carbon of the aldehyde and ketone functional groups is readily transformed into a nucleophilic center through the generation of ketyl radical species *via* a single-electron transfer process. This Umpolung reactivity is widely utilized to execute a variety of organic transformations.^24^ Additionally, the classical pinacol coupling reaction also involves a ketyl radical intermediate, which is usually generated by employing strong metal reducing agents, thus requiring harsh reaction conditions, limiting its application to a broad range of functional groups.^25^ Recently, this issue has been largely addressed by developing highly reducing organophotoredox catalysts that operate under mild conditions.^8*c*,10*a*,*b*^ Moreover, extending this strategy to imines produces another key intermediate α-amino radical species, enabling access to biologically and pharmacologically important vicinal diamines, thereby broadening their further applications.^26^ The effectiveness of these newly introduced photo-catalysts (NCs) was investigated in this context. Extensive screening identified NC1 as a promising catalyst for this transformation when acetophenone 6g was employed as a prototype (Scheme 3). The best result was obtained by conducting the reaction in the presence of DIPEA as a terminal reducing agent in acetonitrile solvent, providing 7g in 81% yield.^9^ A brief examination of the scope revealed that benzaldehyde derivatives bearing both electron-donating and -withdrawing substituents were compatible under these optimized conditions (7a–7f, 64–99% yields). Moreover, acetophenone derivatives with significantly higher potential barriers were also identified as viable reaction partners (7h–7i, 63–95% yields). To assess the synthetic utility further, *N*-benzyl aldimines containing both electron-donating and -withdrawing substituents were subjected to the standard reaction conditions, yielding the desired symmetrical 1,2-diamines 9a–9e in good-to-excellent yields (Scheme 3). Furthermore, imines derived from 3-chloro-aniline also took part in the reaction (9f, 96% yield). A fluorescence quenching study suggested that the photo-excited NC1* is quenched by carbonyl 6g*via* a SET process,^9^ leading to the generation of a ketyl radical species. The catalyst returns to its ground state through reductive quenching by DIPEA. Finally, homo-coupling of the radical species furnishes the desired products 7 and 9.

**Scheme 4:**
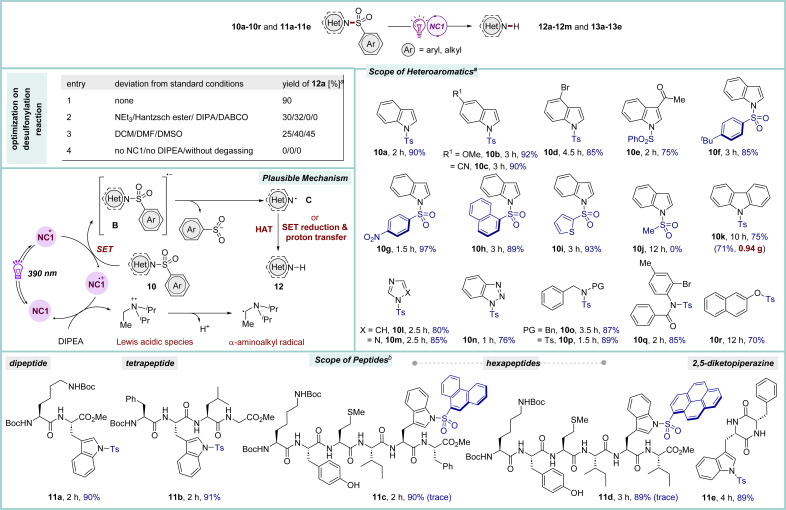
Reductive desulfonylation reactions in heteroaromatics and peptides. ^*a*^Reaction conditions: heteroaromatics 10 (0.5 mmol), DIPEA (3 equiv.), NC1 (0.5 mol%), MeCN (2 mL), under Ar, purple LED (390 nm), rt (∼35–38 °C). ^*b*^Using 0.1 mmol of *N*-sulfonylated peptide 11 in 1.5 mL of MeCN.

Nitrogen-based aromatic heterocycles are common structural motifs present in several drugs and pharmaceutically active compounds.^27^ Many nitrogen heterocycles possess one or more free N–H functional groups, which often interfere during reactions, thus necessitating their protection with synthetically compatible groups that can easily be incorporated, remain stable under various reaction conditions and can readily be removed at the end. In this regard, the sulfone protecting group has played a crucial role in organic synthesis.^28^ Although there are various classical methods available for their deprotection, the removal of sulfones under mild conditions, particularly in a very complex molecular architecture is highly desirable. In this study, first, efforts were devoted to investigating the effectiveness of NC catalysts in the reductive desulfonylation reactions of *N*-sulfonylated aza-heteroaromatic scaffolds (Scheme 4). Through a concise optimization process, NC1 was found to be the most suitable catalyst for the desulfonylation of *N*-tosylindole 10a under irradiation with purple LED light. Pleasingly, the reaction proceeded efficiently employing only 0.5 mol% of catalyst when the substrate concentration was maintained at 0.25 M to furnish 12a in 90% yield.^9^ A range of sulfonylated indoles and pharmacologically significant aza-heteroaromatic compounds participated in this reaction (10b–10i and 10k–10q, 75–97% yields). Notably, the mesyl-protected indole 10j remained unreacted under the reaction conditions, highlighting the chemoselectivity in favour of the aryl sulfone group. Of note, the O–S bond in β-naphthol 10r was also photocatalytically cleaved under the standard conditions.

Due to the difficulties of detection under UV light, the construction of longer oligopeptides in the solution phase poses significant challenges. This issue is mainly overcome by incorporating fluorophore-based protecting groups throughout the synthesis of the peptides, and then their selective removal at the end. Compared to the synthesis of small organic compounds, the protective group chemistry for peptides is still underdeveloped, as many common protecting groups may not be amenable for the synthesis of long peptides owing to the harsh reaction conditions associated with their installation and removal.^29^ The facile removal of sulfonyl protecting groups from different heteroaromatics triggered our interest in studying sulfonylated peptides as a substrate. In this line, *N*-sulfonylated tryptophan-derived oligopeptides were chosen as precursors.^28^ To our delight, the conditions used for the removal of sulfonyl groups in the various sulfonylated heterocycles 10 were also suitable for peptides 11 (Scheme 4). The tryptophan-based oligopeptides (up to hexapeptides) possessing nonpolar hydrophobic, polar, and basic side chains underwent smooth desulfonylation to furnish 13a–13d in 89–91% yields by employing 0.5 mol% of NC1. Moreover, tosylated Trp containing 2,5-diketopiperazine (DKP) 11e, the smallest cyclic peptide, which has immense biological significance, was also amenable to this photo-catalytic system.^30^ Luminescence quenching experiments suggest a plausible reaction mechanism in which photo-excited NC1* is quenched *via* a SET process by sulfone-protected aza-heteroaromatic system 10 to generate radical anion B, which undergoes fragmentation to form *N*-centered radical species C. The catalyst returns to its ground state through reductive quenching by DIPEA. The product 12 was afforded either *via* SET reduction and proton transfer or *via* hydrogen atom transfer (HAT) processes.

## Conclusion

In conclusion, we have presented a novel class of naphthocarbazole-based organic photoredox catalysts (NCs) in this report. Through a combination of comprehensive photophysical analysis and detailed DFT calculations, the complete structure–property relationships have been assessed for these efficient photo-reductants, which possess much lower excited state oxidation potentials and extended excited state lifetimes. Notably, under visible light irradiation, valuable dehalogenative reductive synthetic transformations of aryl halides, such as intermolecular C–B and C–P bond formation and intramolecular cyclization *via* C–C coupling were achieved through the NC-catalyzed generation of aryl radical intermediates. The effectiveness of the NCs as catalysts was also demonstrated in reductive pinacol and imino-pinacol coupling reactions. Along with the desulfonylation of a range of structurally diverse sulfonylated heteroaromatics, these newly developed catalysts were also superior in catalyzing the target-specific reductive desulfonylation of *N*-sulfonyl-tryptophan-containing oligopeptides in the solution phase. Overall, a total of six different types of reactions were demonstrated by employing these novel yet readily accessible naphthocarbazole catalysts featuring mild reaction conditions, excellent catalyst loading, broad substrate scope, adaptability to gram-scale synthesis, and functional group tolerance, which will certainly place them as a natural choice for finding unique reactivities and novel synthetic strategies.

## Data availability

The synthetic procedures, photophysical experimental data and diagrams (UV-visible, fluorescence, cyclic voltammetry, time resolved fluorescence measurement), NMR and computational data are available in the ESI.†

## Author contributions

S. D., S. K., A. M. and M. S. M. contributed to the design of the experiments, discussion, and preparation of the manuscript. All the experiments in the laboratory were carried out by S. D. and S. K. The DFT calculations were performed by A. M.

## Conflicts of interest

There are no conflicts to declare.

## Supplementary Material

SC-015-D4SC03438D-s001
