# Supplementary material for: A toolbox approach to revealing a series of naphthocarbazoles to showcase photocatalytic reductive syntheses
Source: Chem Sci. 2024 Jul 16;15(33):13466–74. doi: 10.1039/d4sc03438d (PMC11339970; doi:10.1039/d4sc03438d)

Supplementary Information (SI) for Chemical Science.
This journal is © The Royal Society of Chemistry 2024

Supporting Information

A toolbox approach to revealing series of naphthocarbazoles to showcase photocatalytic reductive syntheses

Sharmila Das, Samrat Kundu, Abhisek Metya and Modhu Sudan Maji*

Department of Chemistry, Indian Institute of Technology Kharagpur, Kharagpur-721302, India.

E-mail: msm@chem.iitkgp.ac.in

Table of contents	Page Number
General Remarks	**S1**
General Setup for the Reaction	**S2**
Experimental Section for Photo-Physical Studies	**S2-S13**
Luminescence Quenching Experiments	**S14-S16**
General Procedures	**S16-S22**
General Procedure for Borylation (GP-III)	**S22-S23**
General Procedure for the Phosphorylation (GP-IV)	**S24-S25**
General Procedure for the Reductive C-C Coupling of *ortho*-bromo substituted N-phenyl benzamide (GP-V)	**S25-S26**
General Procedure for the Pinacol and Imino-Pinacol Coupling (GP-VI and GP-VII)	**S27-S29**
General Procedure for the Reductive Desulfonylation of Heteroaromatic Compounds and Peptides (GP-VIII and GP-IX)	**S29-S31**
Gram Scale synthesis	**S31-S34**
Compound Characterization Data of Synthesized Compounds	**S34-S61**
Computational Details	**S61-S78**
References	**S79-S81**
^{1}H, ^{13}C, ^{11}B, ^{31}P and ^{19}F NMR Spectra	**S82-S197**

Experimental Sections

General Remarks: All reactions involving air or moisture-sensitive reagents were carried out in flame dried glassware under argon atmosphere. Ethyl acetate was obtained from SRL India. Acetonitrile (CH_3CN) and dichloromethane (CH_2Cl_2) were distilled over calcium hydride. Dimethyl sulfoxide (DMSO) and *N*, *N*-dimethylformamide (DMF) were distilled over potassium carbonate. All the dry solvents were stored over 4 Å molecular sieves under argon atmosphere. Tetrahydrofuran was distilled over sodium-metal in the presence of benzophenone. Aldehydes, ketones and all other reagents were used without further purification as received from commercial sources. All photoredox reactions were performed with Kessil® PR160L-390 nm Purple LED and when performing the photochemical reactions, test tube (made of borosilicate) was placed 4 cm away from light source. No filters were used between light sources and the reaction vessel for the execution of reactions. Reactions were monitored by thin-layer chromatography (TLC) using Merck silica gel 60 F254 pre-coated plates (0.25 mm), and visualized under UV light or by dipping into appropriate staining solutions ($KMnO_4$, DNP, anisaldehyde or ninhydrin solutions). Purification of compounds were carried out by flash column chromatography on Silica gel (particle size 100-200 mesh, purchased from SRL India) or activated neutral aluminium oxide using mixture of hexanes and ethyl acetate as eluents. The ^{1}H NMR spectroscopic data were recorded with a Bruker 400 or 500 MHz instruments. Proton decoupled ^{13}C NMR spectra ($^{13}C\{^{1}H\}$) was recorded at 101 or 126 MHz NMR instruments by using a broadband decoupled mode. ^{11}B, ^{19}F, and ^{31}P NMR spectra were recorded at 160 MHz, 471 MHz and 202 MHz, respectively. Proton and carbon NMR chemical shifts (δ) are reported in parts per million (ppm) relative to the residual proton or carbon signals in $CDCl_3$ (δ = 7.26, 77.16) or DMSO-d_6 (δ = 2.50, 39.52). Coupling constants (*J*) are reported in Hertz (Hz) and refer to apparent multiplicities. The following abbreviations are used for the multiplicities: s: singlet, d: doublet, t: triplet, q: quartet, dd: doublet of doublets, dt: doublet of triplet, m: multiplet, br: broad. Infrared (IR) spectra were recorded by Perkin Elmer FTIR spectrometer, and reported in terms of wave number (cm^{-1}). High resolution mass spectra (HRMS) were recorded in ESI (+ Ve) method using a time-of-flight (TOF) mass analyser.

General set-up for photochemical reaction:

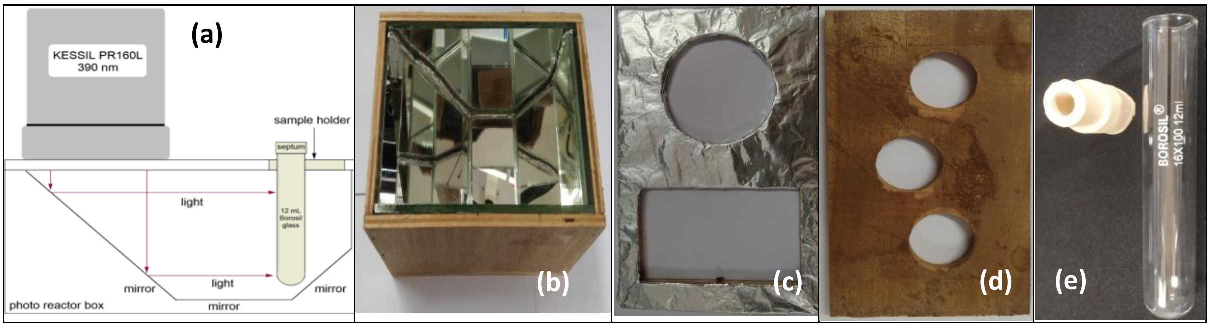

Figure S1: (a) Diagram of the photoreactor box. (b) Photoreactor box with several mirrors. (c) Box closer. (d) Sample holder rack. (e) 12 mL borosil reaction test tube and rubber septum.

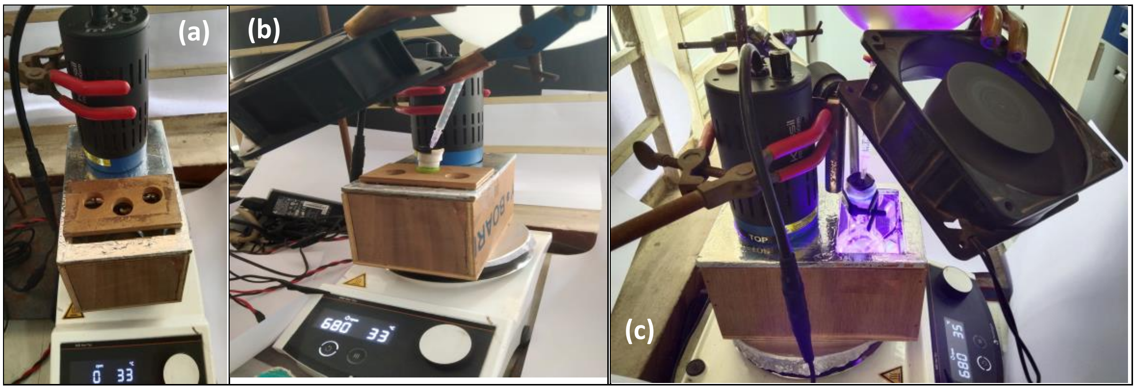

Figure S2: (a) Photoreactor set-up with Kessil light. (b) Complete reaction set-up with one cooling fan. (c) Set up of the gram scale reaction under irradiation Kessil purple LED (390 nm).

Experimental Section for Photo-Physical Studies:

Steady State Absorption and Fluorescence Measurements: UV-Vis and fluorescence measurements have been done in Shimadzu UV−Vis spectrophotometer (model UV 2450) and Hitachi F-7000 fluorimeter, respectively. All samples were taken in micro molar concentrations using dichloromethane as solvent. Molar extinction coefficients (ε) were calculated from Beer-Lambert law (A= εcl) where optical path length (l) was kept as 1 cm and micro molar concentration (c) of the samples were maintained.

Fluorescence Quantum Yield: For calculating quantum yield, all the measurements were performed by maintaining the concentration of the samples and reference at a low value of 3 μM to minimize error due or self-aggregation or self-quenching. The absorbance (A) values of all the solutions were kept below 0.1. Reference dye quinine sulphate (λ_{abs} = 350 nm) in 0.5 M H_2SO_4 (Φ_R= 0.546 at 298 K)[1] was used for measuring fluorescence quantum yields (Φs).

The quantum yield was calculated by using the following equation:[2]

$$\frac{\phi_s}{\phi_R} = \frac{Abs_R}{Abs_s}\frac{A_s}{A_R}\frac{\eta_s^2}{\eta_s^2}$$

Here Φ denotes the quantum yield, (Abs) denotes the absorbance, A_S and A_R denotes the area under the fluorescence emission curve and η is the refractive index of the medium. The subscript S and R represent the corresponding parameters for the samples and reference, respectively.

Time Resolved Decay Measurements: The excited state lifetime decay trace was monitored using Deltaflex TCSPC module in the reverse mode having repetition rate 20 MHz & time scale 50 ns. The signal was detected at a magic angle (54.7°) using Photon Counting Detector (PPD 800). The excitation source was a picosecond diode laser unit with excitation wavelength 376 nm. The instrument response function (IRF) for 376 nm excitation laser in this TCSPC set up was ~230 ps. The decay was collected in reverse mode and fitted using EzTime decay analysis software and using single exponential decay function.

$$(t) = b + a\exp(-\frac{t}{\tau})$$

Here, "b" is a baseline correction ("dc" offset), and "a" and "τ" are pre-exponential factor and excited-state fluorescence lifetime, respectively.

In all experiments, dichloromethane (DCM, spectroscopic grade, purchased from Spectrochem) was used as solvent. For photophysical studies, 2 mM solution of the photocatalyst (**NC**) was prepared in a sample vial by dissolving 0.0025 mmol of NC in 1.16 mL of DCM. The freshly prepared solution was used for the spectroscopic measurements. The required amount in micromolar concentration was taken using micro pipette from the mother solution as an aliquot and it was diluted further by dissolving in 2 mL of DCM in the cuvette such that the final micromolar concentration of catalyst will be optimum to obtain expected spectra for absorption and fluorescence measurements. For obtaining all the emission spectrum, 350 nm excitation light was used.

Cyclic Voltammetry:[3]

Electrochemical potentials were obtained with a standard set of conditions to maintain internal consistency. Cyclic voltammetry (CV) and square wave voltammetry (SWV) measurements were performed (maintaining IUPAC convention) by using CH Instruments Electrochemical Analyzer/Workstation Model 660E Series. The polishing material was used for the cyclic voltammetry experiments is named as Micropolish Powder 0.05 micron. To polish, small amount of alumina powder was put on the polishing pad and wet with distilled water. The electrode was hold vertically and firmly (did not apply too much pressure on this) while polishing. When the polishing pad get dry, distilled water was added and polished again. A standard three-electrode cell was employed with glassy carbon (3 mm diameter) as working electrode, platinum wire as auxiliary electrode and Ag/AgCl (KCl, 3M) as reference electrode; along with that tetra-*n*-butylammonium hexafluorophosphate ($^{n}Bu_4NPF_6$) was employed as supporting electrolyte. Scan rate was set to 100 mV/s. Samples were prepared with 0.0025 mmol of substrate in 6 mL of 0.1 M $^{n}Bu_4NPF_6$ in anhydrous acetonitrile and *N*, *N*-dimethylformamide solvent mixture (MeCN/DMF = 5:1 v/v, for enhancing solubility of the catalyst). Solutions were degassed with argon prior to measurements and experiments were performed under a protective atmosphere of argon. All potentials were measured relative to Ag/AgCl at 25 °C. The applied solvents are specified in the descriptions of the corresponding experiments. CV studies were also performed looking at variable scan rates (25 to 700 mV/s) to ultimately determine the reversibility of the catalysts.

The zero-zero vibrational state excitation energy $E_{0,0}$ was estimated by the corresponding energy of the wavelength at which emission and absorption overlap. This wavelength was determined setting the intensity of emission λ_{max} to the absorbance at excitation wavelength (350 nm). E_{ox} and E_{red} are the ground state oxidation and reduction potentials which were determined from cyclic voltammetry experiments. Excited state oxidation potential (E^*_{ox}) and excited state reduction potential (E^*_{red}) were calculated by the following approximating formulas:

(Excited state oxidation potential) E^*_{ox} ($NC^{\bullet+}$ / NC*) = E_{ox} ($NC^{\bullet+}$ / NC) − $E_{0,0}$

(Excited state reduction potential) E^*_{red} (NC* / $NC^{\bullet-}$) = E_{red} (NC / $NC^{\bullet}$) + $E_{0,0}$

Conversion of the potential from Ag/Ag+ to SCE:

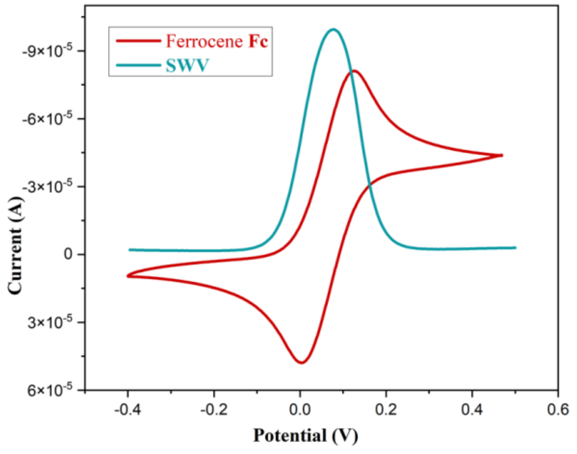

Figure S3: CV and SWV of Ferrocene vs Ag/Ag^+

The conversion of the redox potential from Ag/AgCl to SCE was done according to the literature procedure[4] by measuring the redox potential for ferrocene as reference in similar solvent mixture (MeCN/DMF 5:1 v/v). From cyclic voltammetry, (Fig S3) we observed the redox potential of ferrocene (Fc): 0.07 V vs Ag/AgCl. With the reference CV, the redox potential vs SCE in MeCN/DMF 5:1 v/v was calculated using the following equations.

$E_{1/2}$ ($NC^{\bullet+}$/NC) (vs Fc/Fc^+) = (Ground state oxidation potential of NC vs Ag/AgCl) – 0.07

$E_{1/2}$ ($NC^{\bullet+}$/NC) (vs SCE) = (Ground state oxidation potential of NC vs Fc/Fc^+) + 0.38

$E_{1/2}$ ($NC1^{\bullet+}$/NC1) = 0.753 V vs Ag/AgCl = 1.063 V vs SCE

$E_{1/2}$ ($NC2^{\bullet+}$/NC2) = 0.724 V vs Ag/AgCl = 1.034 V vs SCE

$E_{1/2}$ ($NC3^{\bullet+}$/NC3) = 0.696 V vs Ag/AgCl = 1.006 V vs SCE

$E_{1/2}$ ($NC4^{\bullet+}$/NC4) = 0.776 V vs Ag/AgCl = 1.086 V vs SCE

$E_{1/2}$ ($NC5^{\bullet+}$/NC5) = 0.904 V vs Ag/AgCl = 1.214 V vs SCE

$E_{1/2}$ ($NC6^{\bullet+}$/NC6) = 0.844 V vs Ag/AgCl = 1.154 V vs SCE

Table S1: Photoredox Properties of the Synthesized Photocatalysts

Catalyst (NC)	$\lambda_{abs,max}$ (nm)	ε^a	$\lambda_{em,max}$ (nm)	λ_{inter} (nm)	$E_{0,0}$ (eV)	τ (ns)	E_{ox} (V)	E^*_{ox} (V)	Φ (%)
**NC1**	302, 370, 390	7.13, 0.71, 0.81	425	399	3.10	5.90	1.063	-2.037	0.374
**NC2**	302, 370, 390	6.38, 0.78, 0.84	425	401	3.09	5.78	1.034	-2.056	0.371
**NC3**	302, 370, 390	8.00, 0.86, 1.01	429	402	3.08	5.97	1.006	-2.074	0.438
**NC4**	302, 370, 390	5.10, 0.75, 0.83	426	400	3.10	5.59	1.086	-2.014	0.385
**NC5**	304, 369, 390	6.61, 0.53, 0.61	427	398	3.11	7.12	1.214	-1.896	0.313
**NC6**	303, 367, 386	3.70, 0.47, 0.52	423	394	3.15	6.11	1.154	-1.996	0.618

[a]ε in units of 10^4 $M^{-1}cm^{-1}$. [b]All the ground state potentials were calculated in SCE. 'abs' denotes absorbance and 'em' denotes emission.

Photoredox Characteristics of Each Photocatalyst:

13-Methyl-7,8-diphenyl-13H-naphtho[2,1-a]carbazole (NC1):

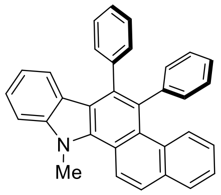

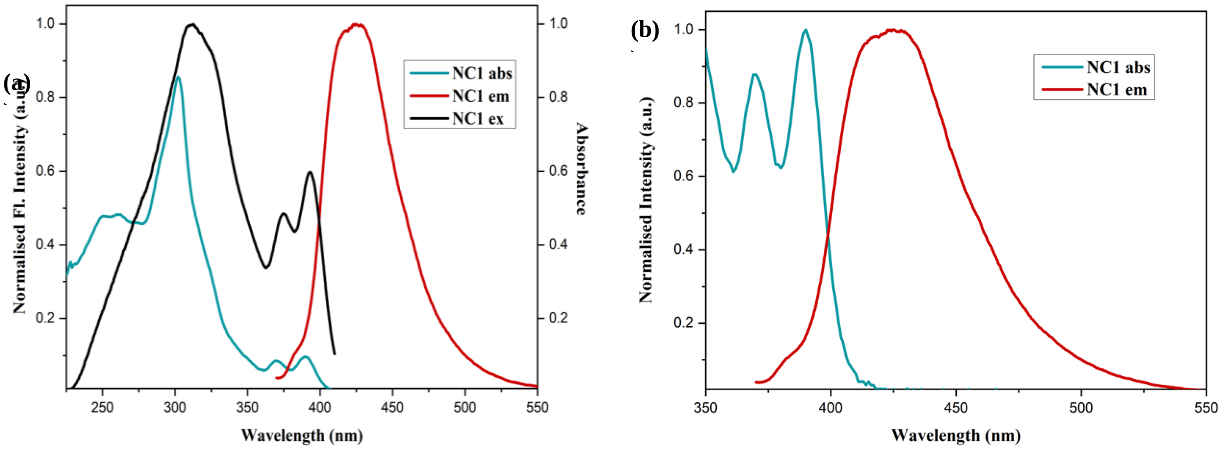

Figure S4A: (a) UV/Vis absorption (light cyan line), excitation (black line) and emission (red line) spectra of photocatalyst **NC1** (12 µM, in DCM). (b) Normalized absorption and emission spectra of **NC1**. Excitation wavelength λ_{max} in DCM = 350 nm, intersection wavelength $\lambda_{intersection}$ =399 nm; $E_{0,0}$ = 1240/$\lambda_{intersection}$ = 3.10 eV; Stokes shift = 35 nm.

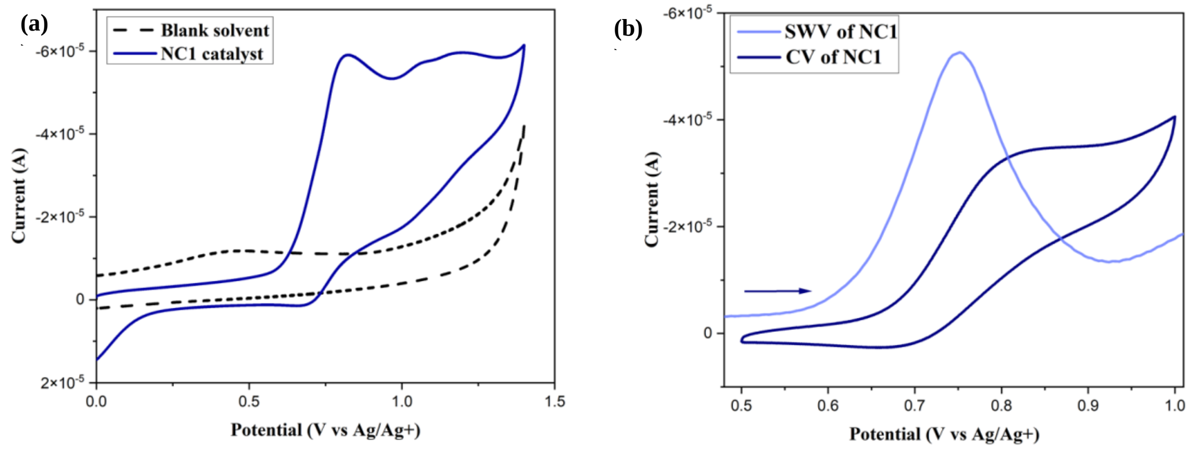

Figure S4B: (a, b) Cyclic voltammogram (CV) of **NC1** (blue line), blank (dash black line) and square wave voltammetry (SWV) of **NC1** (light blue line) in MeCN/DMF 5:1 v/v; concentration of analyte [**NC1**] = 7.6 x 10^{-4} M and electrolyte [$^{n}Bu_4NPF_6$] = 0.1 M; working electrode: glassy carbon, reference electrode: Ag/AgCl (3.0 M in KCl), counter electrode: platinum wire; for SWV frequency = 15 Hz, amplitude = 0.025 V; temperature = 25 ºC.

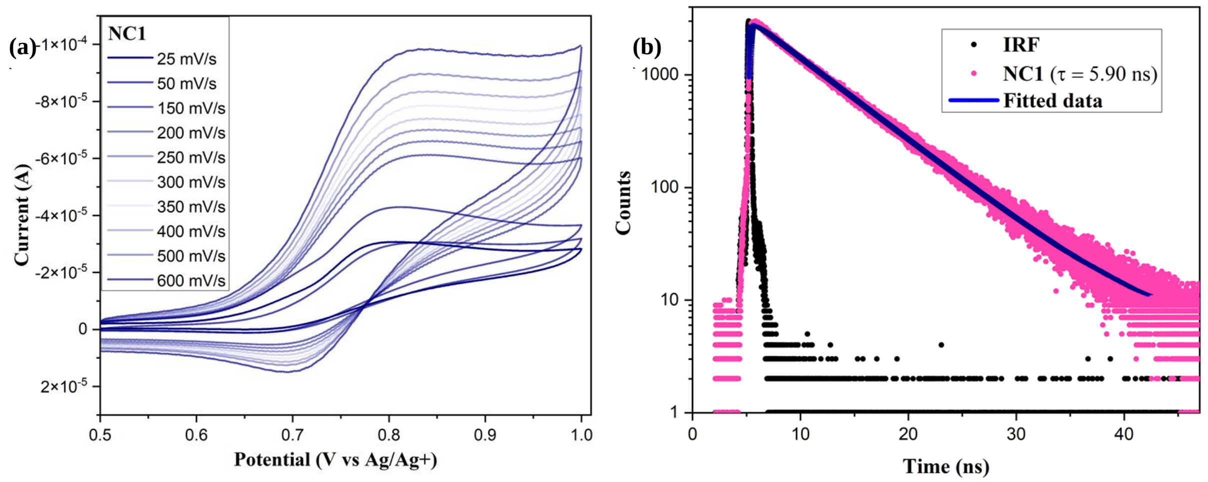

Figure S4C: (a) CV experiments at variable scan rates of **NC1**; (b) Fluorescence decay curve of **NC1** in DCM (12 μM). Lifetime τ in DCM: 5.90 ± 0.01 ns in DCM; χ^2 = 1.359.

8-(3-Methoxyphenyl)-13-methyl-7-phenyl-13H-naphtho[2,1-a]carbazole (NC2):

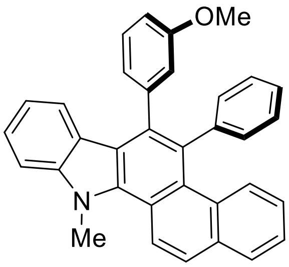

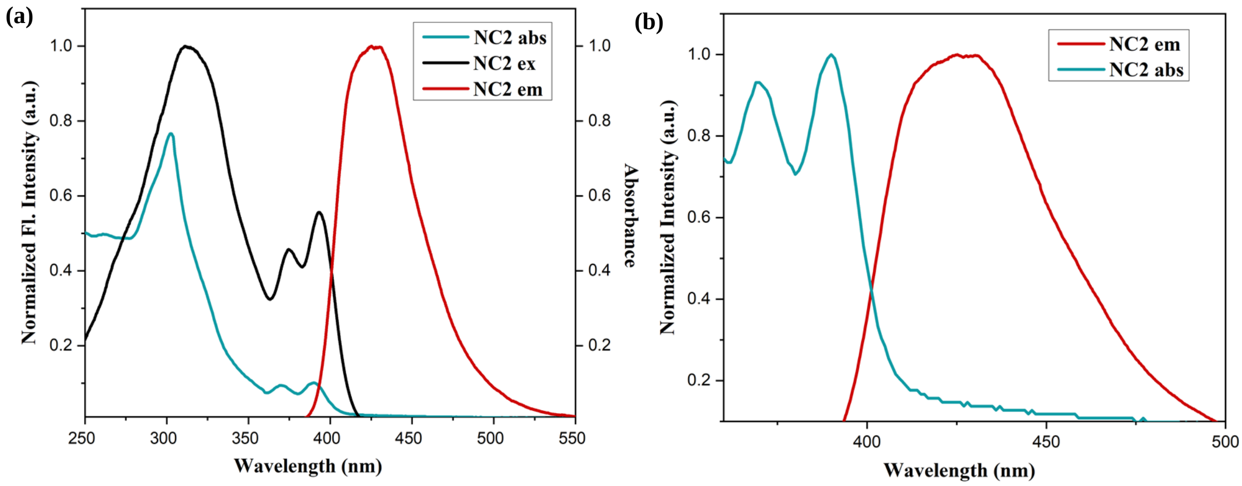

Figure S5A: (a) UV/Vis absorption (light cyan line), excitation (black line) and emission (red line) spectra of photocatalyst **NC2** (12 μM, in DCM), (b) Normalized absorption and emission spectra of **NC2**. Excitation wavelength λ_{max} in DCM = 350 nm, intersection wavelength $\lambda_{intersection}$ = 401 nm; $E_{0,0}$ = 1240/$\lambda_{intersection}$ = 3.09 eV; Stokes shift = 35 nm.

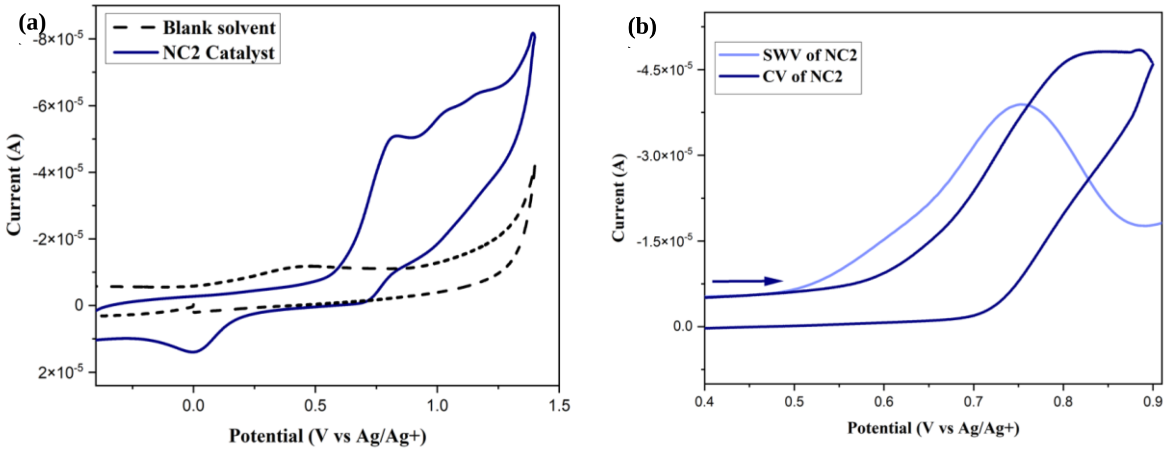

Figure S5B: (a, b) Cyclic voltammogram (CV) of **NC2** (blue line), blank (dash black line) and square wave voltammetry (SWV) of **NC2** (light blue line) in MeCN/DMF 5:1 v/v; concentration of analyte [**NC2**] = 7.2 x 10^{-4} M and electrolyte [$^{n}Bu_4NPF_6$] = 0.1 M; working electrode: glassy carbon, reference electrode: Ag/AgCl (3.0 M in KCl), counter electrode: platinum wire; for SWV frequency = 15 Hz, amplitude = 0.025 V; temperature = 25 ºC.

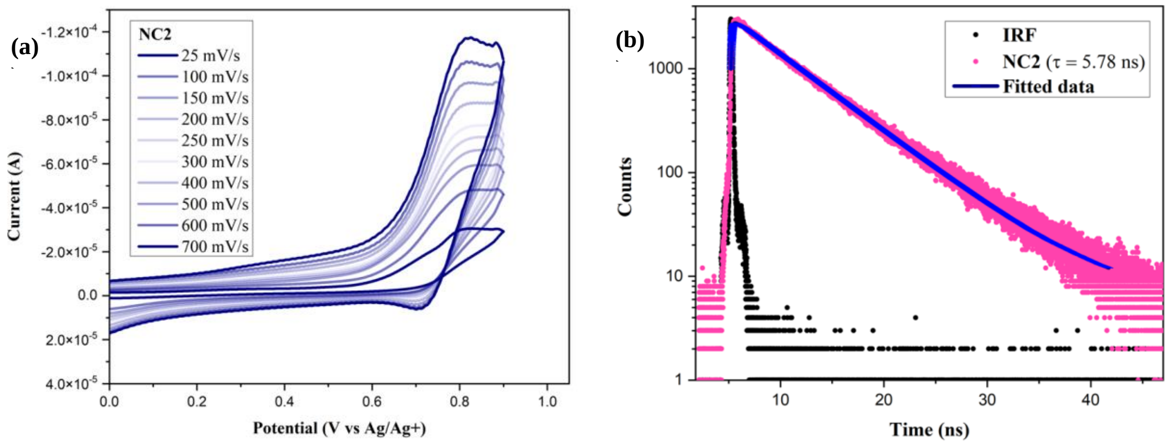

Figure S5C: (a) CV experiments at variable scan rates of **NC2**; (b) Fluorescence decay curve of **NC2** in DCM (12 µM). Lifetime τ in DCM: 5.78±0.01 ns in DCM; χ^2 = 1.408.

8-(4-Methoxyphenyl)-13-methyl-7-phenyl-13H-naphtho[2,1-a]carbazole (NC3):

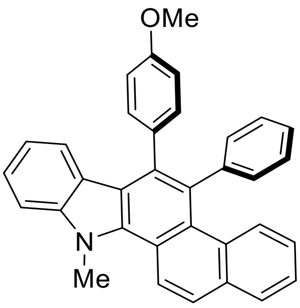

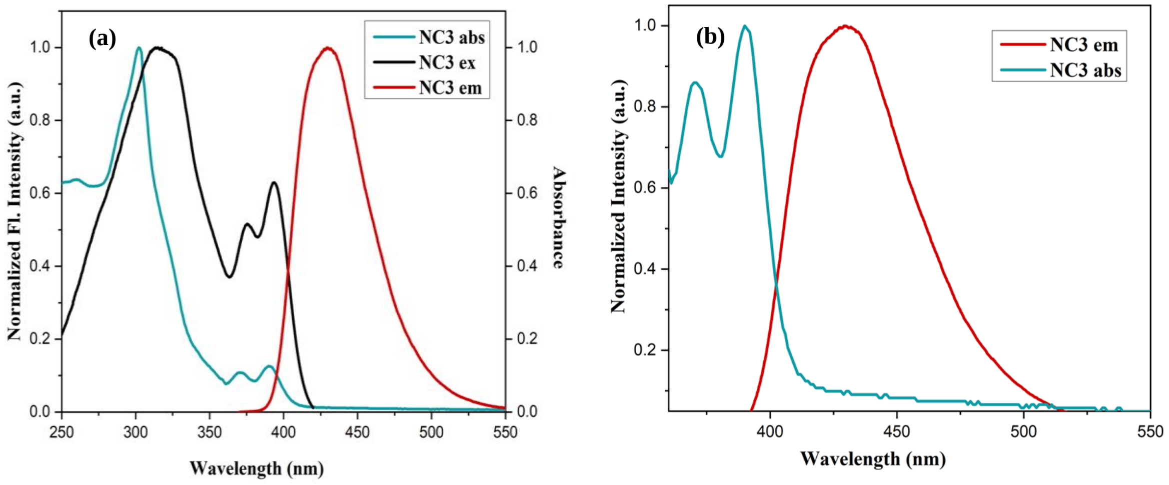

Figure S6A: (a) UV/Vis absorption (light cyan line), excitation (black line) and emission (red line) spectra of photocatalyst **NC3** (12 µM, in DCM), (b) Normalized absorption and emission spectra of **NC3**. Excitation wavelength λ_{max} in DCM = 350 nm, intersection wavelength $\lambda_{intersection}$ = 402 nm; $E_{0,0}$ = 1240/$\lambda_{intersection}$ = 3.08 eV. Stokes shift = 39 nm.

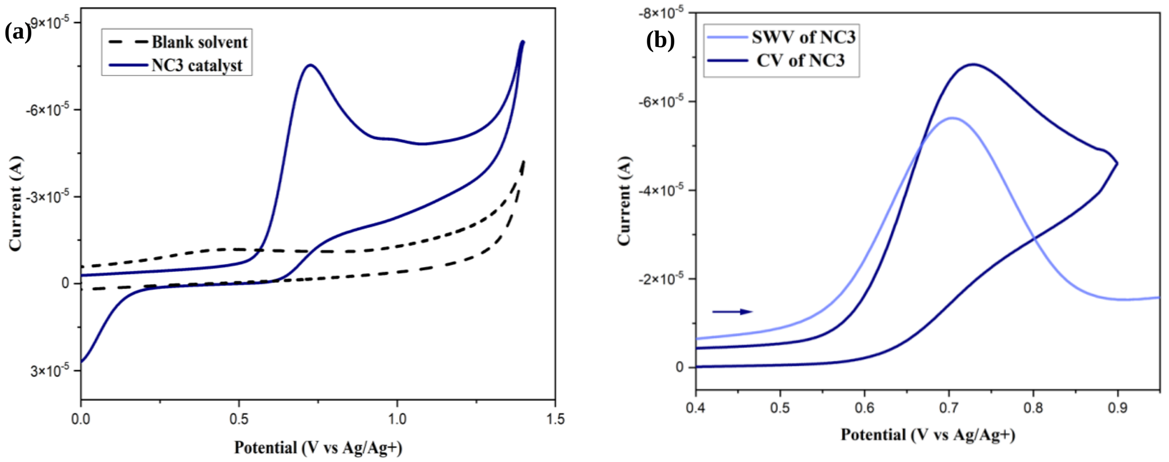

Figure S6B: (a, b) Cyclic voltammogram (CV) of **NC3** (blue line), blank (dash black line) and square wave voltammetry (SWV) of **NC3** (light blue line) in MeCN/DMF 5:1 v/v; concentration of analyte [**NC3**] = 7.2 x 10^{-4} M and electrolyte [$^{n}Bu_4NPF_6$] = 0.1 M; working electrode: glassy carbon, reference electrode: Ag/AgCl (3.0 M in KCl), counter electrode: platinum wire; for SWV frequency = 15 Hz, amplitude = 0.025 V; temperature = 25 ºC.

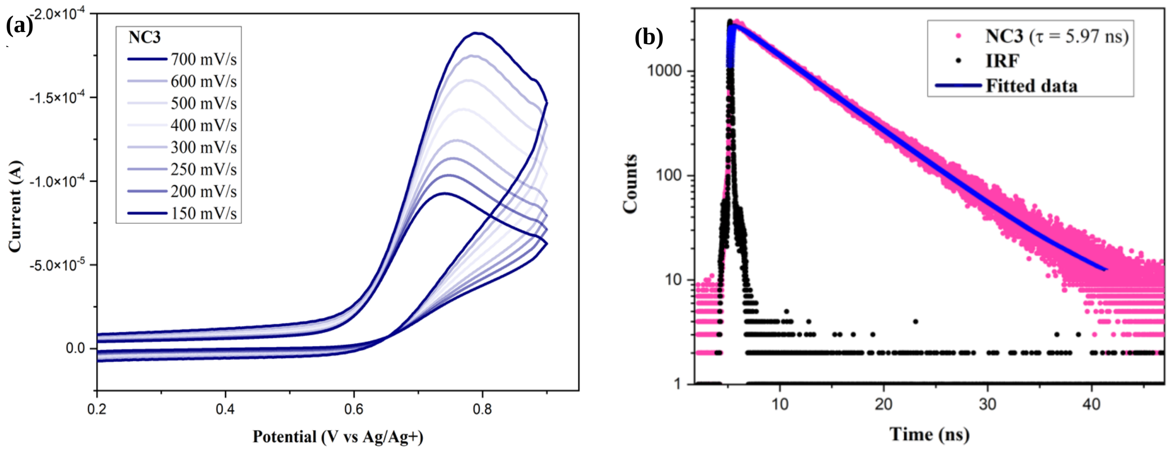

Figure 6C: (a) CV experiments at variable scan rates of **NC3**; (b) Fluorescence decay curve of **NC3** in DCM (12 µM). Lifetime τ in DCM: 5.97 ± 0.01 ns in DCM; χ^2 = 1.245.

8-(4-Chlorophenyl)-13-methyl-7-phenyl-13H-naphtho[2,1-a]carbazole (NC4):

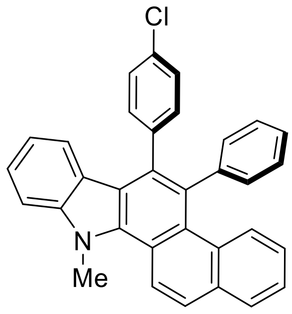

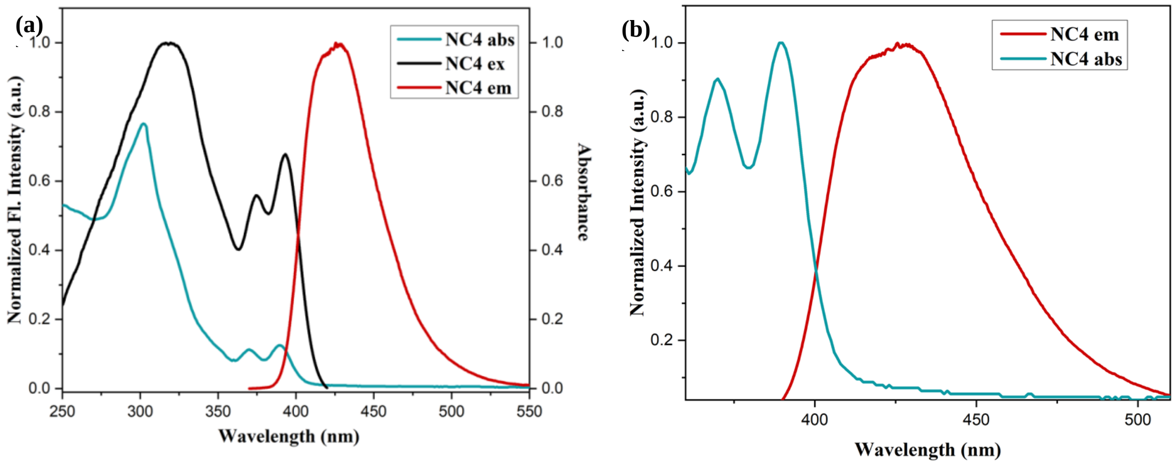

Figure S7A: (a) UV/Vis absorption (light cyan line), excitation (black line) and emission (red line) spectra of photocatalyst **NC4** (15 µM, in DCM). (b) Normalized absorption and emission spectra of **NC4**. Excitation wavelength λ_{max} in DCM = 350 nm, intersection wavelength $\lambda_{intersection}$ = 400 nm; $E_{0,0}$ = 1240/$\lambda_{intersection}$ = 3.10 eV; Stokes shift = 35 nm.

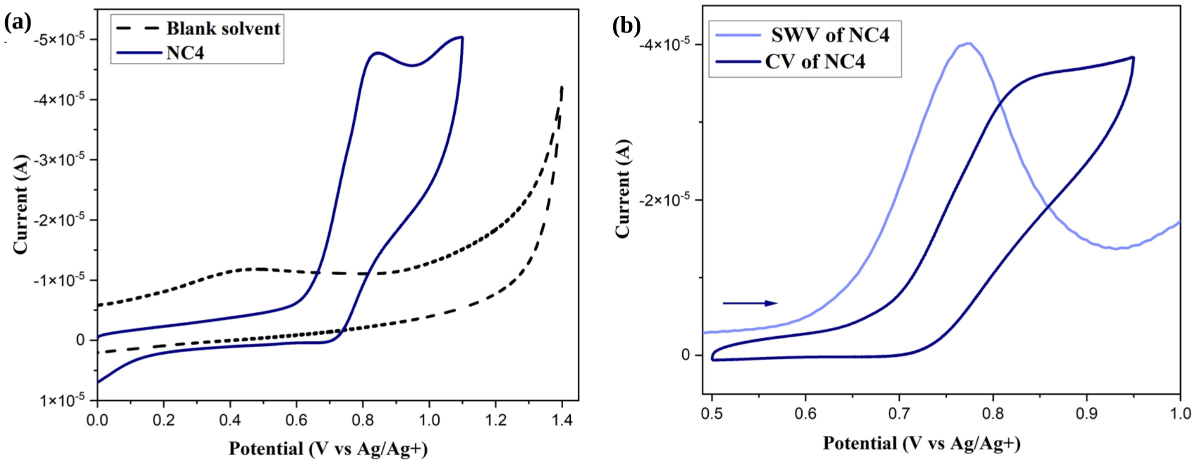

Figure S7B: (a, b) Cyclic voltammogram (CV) of **NC4** (blue line), blank (dash black line) and square wave voltammetry (SWV) of **NC4** (light blue line) in MeCN/DMF 5:1 v/v; concentration of analyte [**NC4**] = 7.1 x 10^{-4} M and electrolyte [$^{n}Bu_4NPF_6$] = 0.1 M; working electrode: glassy carbon, reference electrode: Ag/AgCl (3.0 M in KCl), counter electrode: platinum wire; for SWV frequency = 15 Hz, amplitude = 0.025 V; temperature = 25 ºC.

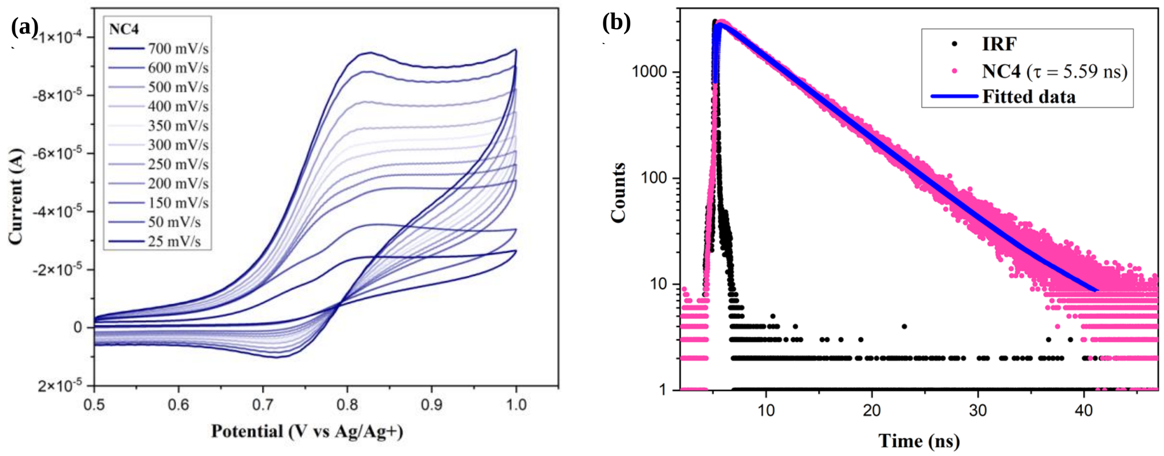

Figure S7C: (a) CV experiments at variable scan rates of **NC4**; (b) Fluorescence decay curve of **NC4** in DCM (15 µM). Lifetime τ in DCM: 5.59 ± 0.01 ns in DCM; χ^2 = 1.173.

13-Methyl-7,8-diphenyl-13H-naphtho[2,1-a]carbazole-10-carbonitrile (NC5):

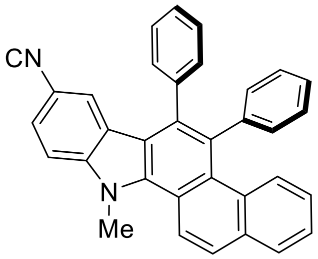

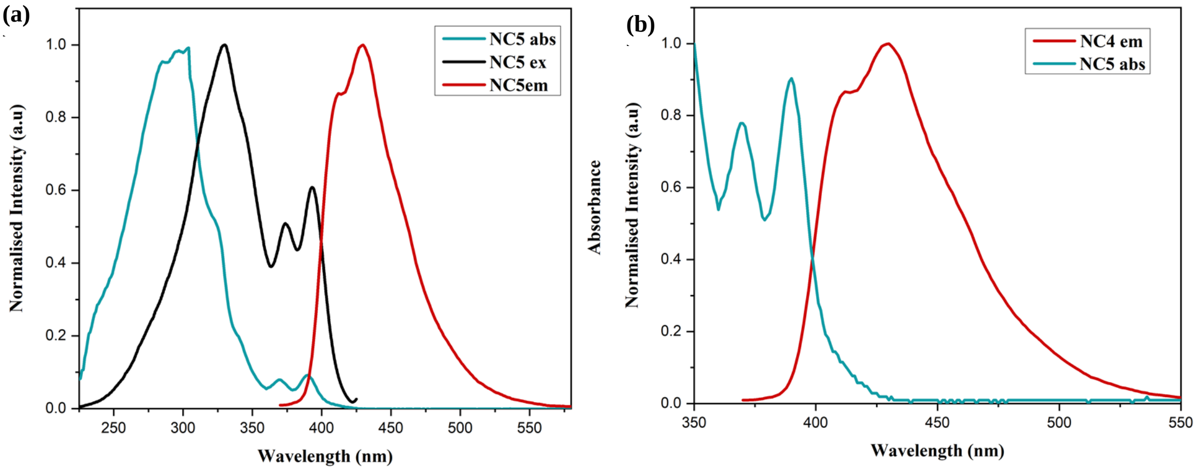

Figure S8A: (a) UV/Vis absorption (light cyan line), excitation (black line) and emission (red line) spectra of photocatalyst **NC5** (15 µM, in DCM). (b) Normalized absorption and emission spectra of **NC5**. Excitation wavelength λ_{max} in DCM = 350 nm, intersection wavelength $\lambda_{intersection}$ = 398 nm; $E_{0,0}$ = 1240/$\lambda_{intersection}$ = 3.11 eV; Stokes shift = 36 nm.

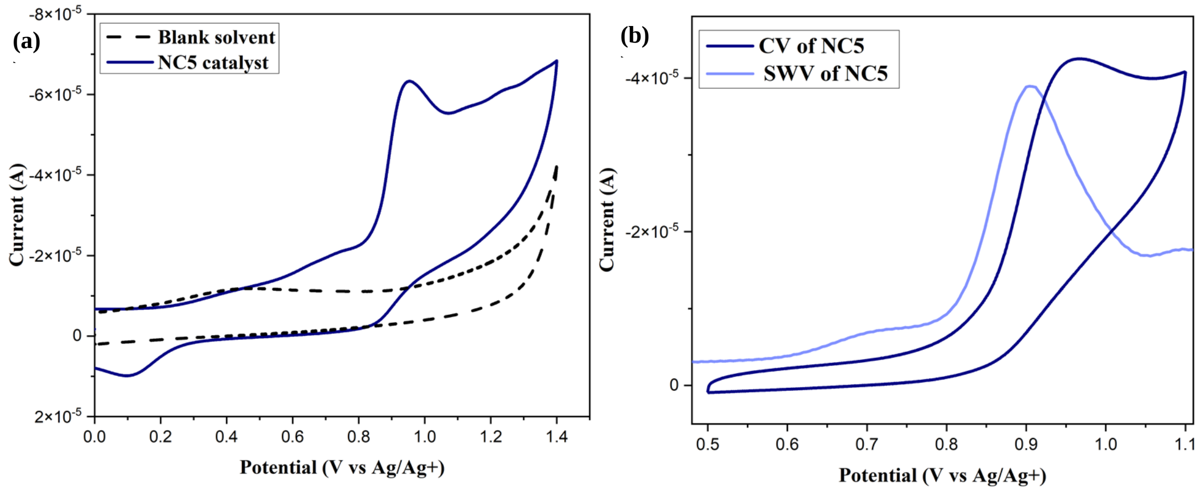

Figure S8B: (a, b) Cyclic voltammogram (CV) of **NC5** (blue line), blank (dash black line) and square wave voltammetry (SWV) of **NC5** (light blue line) in MeCN/DMF 5:1 v/v; concentration of analyte [**NC5**] = 7.3 x 10^{-4} M and electrolyte [$^{n}Bu_4NPF_6$] = 0.1 M; working electrode: glassy carbon, reference electrode: Ag/AgCl (3.0 M in KCl), counter electrode: platinum wire; for SWV frequency = 15 Hz, amplitude = 0.025 V; temperature = 25 ºC.

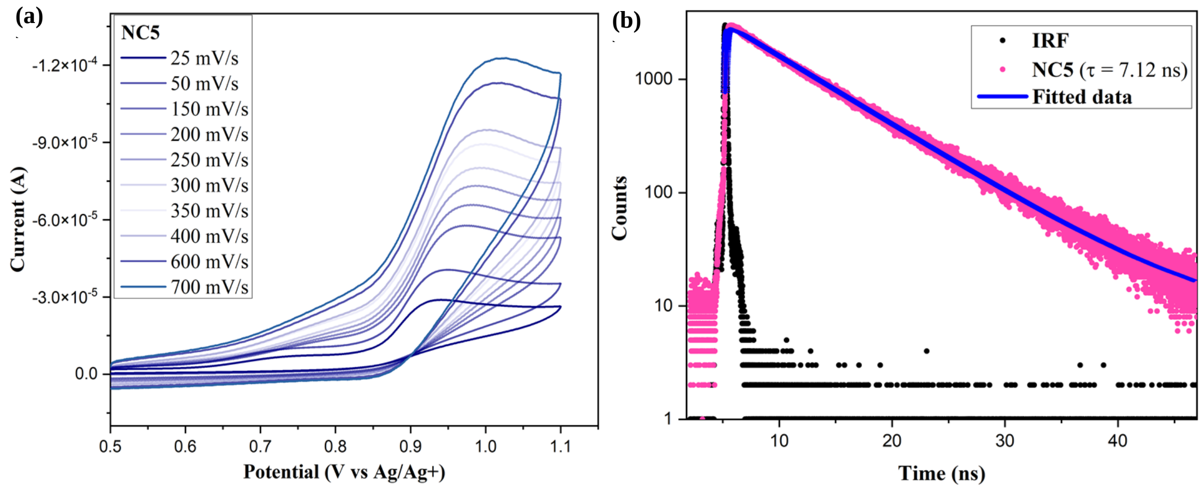

Figure S8C: (a) CV experiments at variable scan rates of **NC5**; (b) Fluorescence decay curve of **NC5** in DCM (15 µM). Lifetime τ in DCM: 7.12 ± 0.01 ns in DCM; χ^2 = 1.467.

7,8,13-Triphenyl-13H-naphtho[2,1-a]carbazole (NC6):

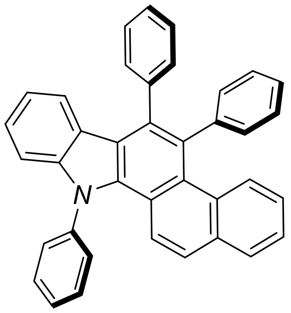

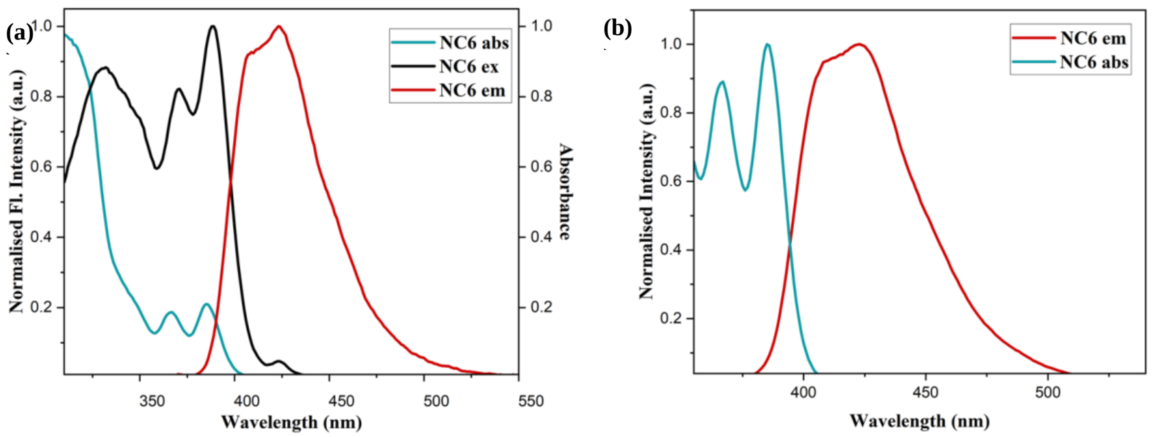

Figure S9A: (a) UV/Vis absorption (light cyan line), excitation (black line) and emission (red line) spectra of photocatalyst **NC6**(40 µM, in DCM). (b) Normalized absorption and emission spectra of **NC6**. Excitation wavelength λ_{max} in DCM = 350 nm, intersection wavelength $\lambda_{intersection}$ = 394 nm; $E_{0,0}$ = 1240/$\lambda_{intersection}$ = 3.15 eV; Stokes shift = 37 nm.

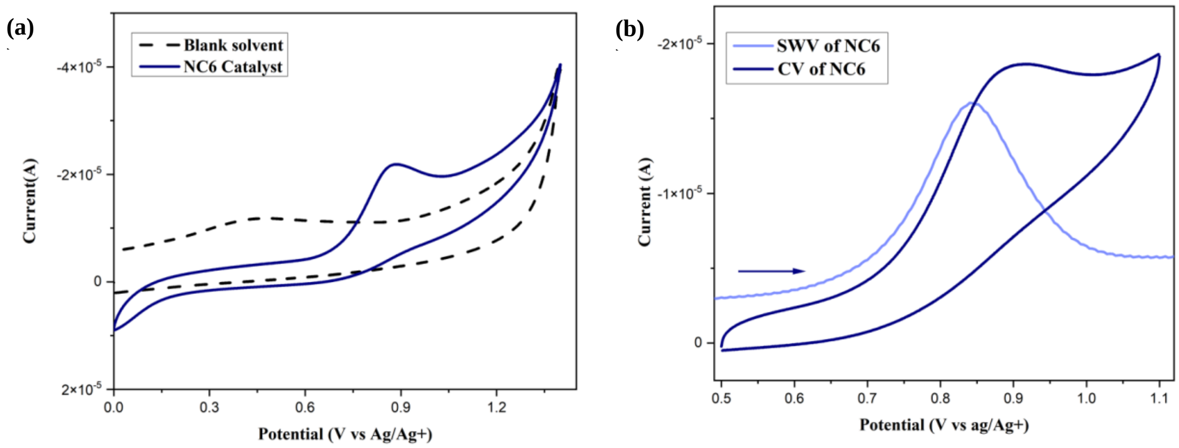

Figure S9B: (a, b) Cyclic voltammogram (CV) of **NC6** (blue line), blank (dash black line) and square wave voltammetry (SWV) of NC6 (light blue line) in MeCN/DMF 5:1 v/v; concentration of analyte [**NC6**] = 6.7 x 10^{-4} M and electrolyte [$^{n}Bu_4NPF_6$] = 0.1 M; working electrode: glassy carbon, reference electrode: Ag/AgCl (3.0 M in KCl), counter electrode: platinum wire; for SWV frequency = 15 Hz, amplitude = 0.025 V; temperature = 25 ºC

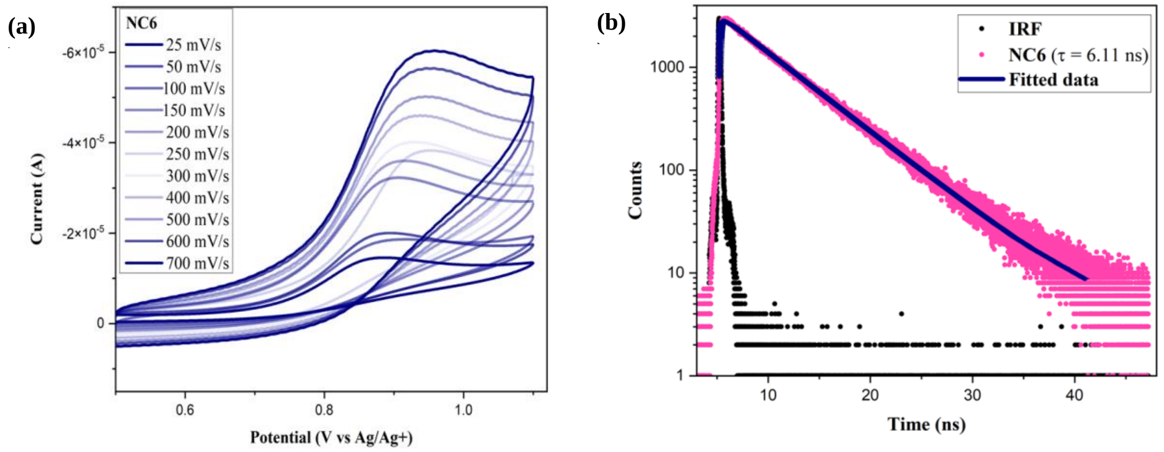

Figure S9C: (a) CV experiments at variable scan rates of **NC6**; (b) Fluorescence decay curve of **NC6** in DCM (40 µM). Lifetime τ in DCM: 6.11 ± 0.01 ns in DCM; χ^2 = 1.745.

Luminescence Quenching Experiments:[5]

Preparation of the stock solution: A 2 mM solution of the photocatalyst was prepared in a sample vial by dissolving 0.0025 mmol of the respective NC catalyst in 1.16 mL of dry dichloromethane (DCM) (spectroscopic grade, purchased from Spectrochem). The freshly prepared solution was used for the spectroscopic measurement. The required amount (12 µL) was taken using micro pipette from the mother solution as an aliquot and it was diluted further by dissolving in 2 mL of DCM in the cuvette such that the final concentration of photocatalyst was 3 µM. Similarly, 0.24 mL 1 M solution of 1-bromo-4-methylbenzene (**1a**), *N*-(2-bromophenyl)-*N*-methylbenzamide (**4a**), acetophenone (**6g**), 1-((4-(*tert*-butyl)phenyl)sulfonyl)-1*H*-indole (**10f**) and 0.35 mL 1 M solution of *N*,*N*-diisopropylethylamine (DIPEA) were prepared by dissolving the requisite amount of each quencher in dry, degassed DCM. Freshly prepared those solutions were used for the quenching experiments.

Experimental procedures: Fluorescence emission spectra of the photocatalyst in absence and presence of different reactions components were recorded and analysed in detail to estimate the light emission properties of the pure catalyst system and their distractions by external interference from the substrates. Emission intensities of photocatalyst were recorded with a "Hitachi F-7000 FL Spectrophotometer" using 10.0 mm quartz cuvette. The sample solution of NC with a proper concentration of in DCM (degassed under inert atmosphere of argon for 5 minutes before recording the spectra) was excited at the wavelength of 350 nm and to observe quenching, the fluorescence intensity was monitored at $\lambda_{em,max}$ (425 nm for NC1 and 429 nm for NC3). The individual substrate and DIPEA did not show any emission in that region. To study the quenching behaviour of photocatalyst, small aliquots of substrate was progressively added to the catalyst solution and the emission spectra were measured following the aforementioned procedure. Few sets of solutions with different concentration of quencher were used; the experiments were repeated and finally the Stern-Volmer plot was obtained according to the following equation.

$$\frac{F_0}{F} = 1 + K_{SV}\,[Q]$$

Where, F_0 and F are fluorescence intensities at $\lambda_{em,max}$ of respective catalyst in absence and presence of quencher, [Q] is corresponding molar concentration of quencher and K_{SV} is the Stern-Volmer constant. DIPEA was found to be comparatively less effective to facilitate

quenching of the emission of the catalyst. The corresponding Stern-Volmer plot was illustrated for all the cases (Fig S10 - Fig S13).

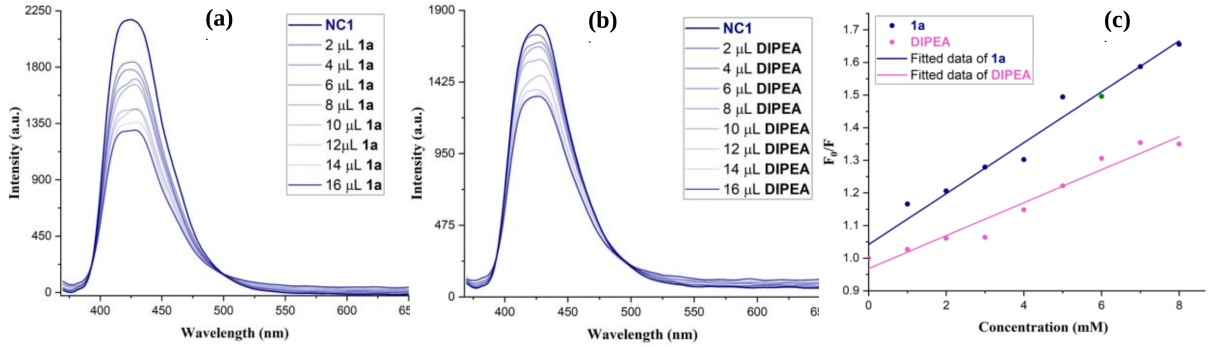

Figure S10: (a, b) Fluorescence quenching study of NC1 catalyst with 1-bromo-4-methylbenzene **1a** and DIPEA for borylation reaction. (c) Stern-Volmer Plot of NC1 catalyst in presence of 1-bromo-4-methylbenzene **1a** and amine DIPEA separately.

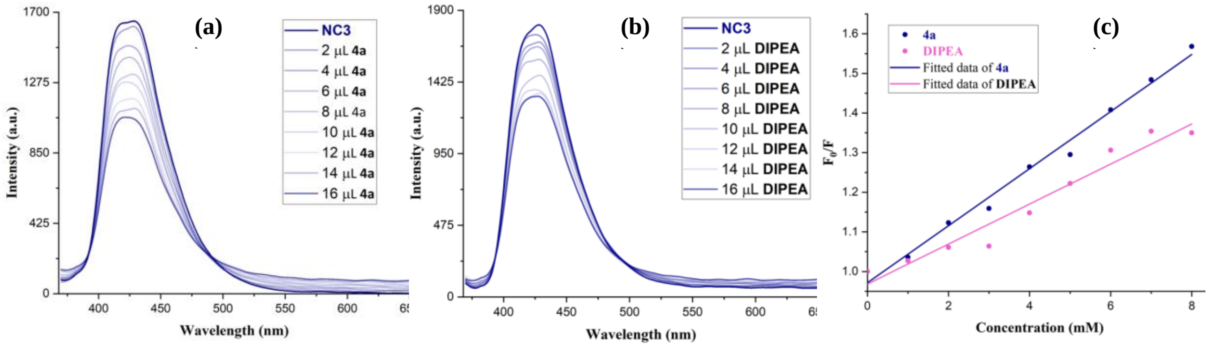

Figure S11: (a, b) Fluorescence quenching study of NC3 catalyst with *N*-(2-bromophenyl)-*N*-methylbenzamide (**4a**) and DIPEA, respectively. (c) Stern-Volmer Plot of NC3 catalyst in presence of *N*-(2-bromophenyl)-*N*-methylbenzamide (**4a**) and amine DIPEA separately.

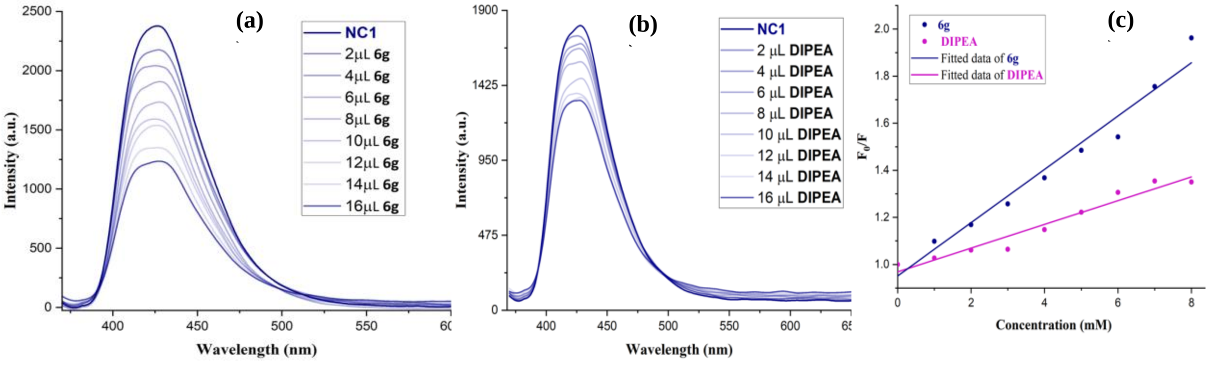

Figure S12: (a, b) Fluorescence quenching study of NC1 catalyst with acetophenone (**6g**) and DIPEA, respectively. (c) Stern-Volmer Plot of NC1 catalyst in presence of acetophenone (**6g**) and amine DIPEA separately.

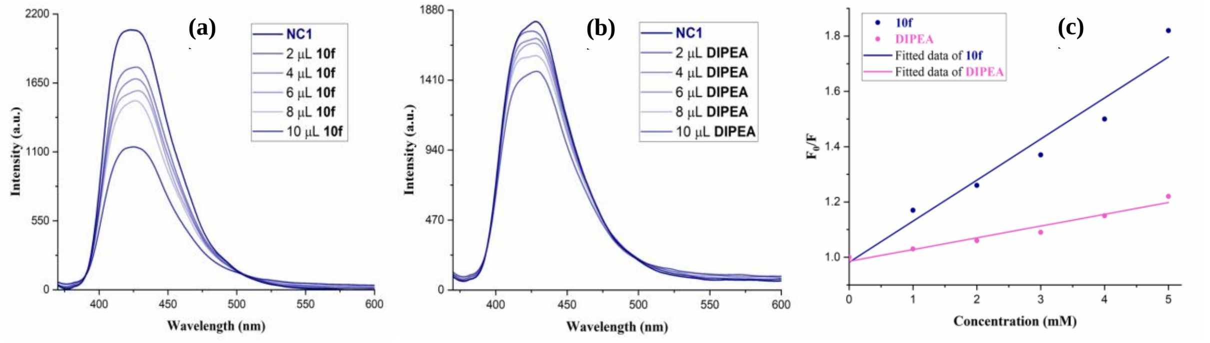

Figure S13: (a, b) Fluorescence quenching study of NC1 catalyst with -((4-(*tert*-butyl)phenyl) sulfonyl)-1*H*-indole (**10f**) and DIPEA, respectively. (c) Stern-Volmer Plot of NC1 catalyst in presence of -((4-(*tert*-butyl)phenyl)sulfonyl)-1*H*-indole (**10f**) and amine DIPEA separately.

General Procedure for the Synthesis of Photocatalysts (NC1-NC4) (GP-I):

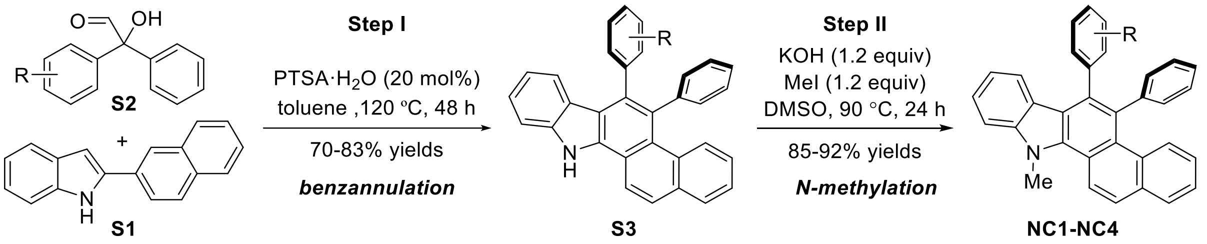

2-Naphthylindole **S1** and di-aryl *α*-hydroxyl aldehydes **S2** were synthesized according to the literature procedures.[6a,6b]

The photocatalysts **NC1-NC4** were synthesized according to the modified reported literature procedure.[6b]

Step I: In a 100 mL round bottom flask, 2-(naphthalen-2-yl)-1*H*-indole **S1** (2.43 g, 10.0 mmol, 1.0 equiv) and *p*-toluenesulfonic acid monohydrate (380 mg, PTSA·H_2O, 2.0 mmol, 20 mol%) were dissolved in 30 mL of toluene by stirring at room temperature for 5 min. Then, di-aryl *α*-hydroxyl aldehydes **S2** (11.0 mmol, 1.1 equiv) were added to it and the reaction mixture was heated to reflux at 120 °C for 48 h with constant stirring. Upon completion of the reaction (as monitored by TLC, the products are $KMnO_4$ and DNP active), the crude reaction mixture was diluted with ethyl acetate and the solvent was evaporated under reduced pressure. The substituted benzo[*a*]carbazoles **S3** were isolated (70-83% yields) as yellow solid after silica gel column chromatography using ethyl acetate in hexane as eluent and were used for the next step.

Step II: In a 50 mL round bottom flask, substituted benzo[*a*]carbazole **S3** (5.0 mmol, 1.0 equiv) was dissolved in 15 mL dimethyl sulfoxide (DMSO) by stirring at room temperature for 5 min. Then potassium hydroxide (KOH, 336.63 mg, 6.0 mmol, 1.2 equiv) was added to the reaction mixture and stirring was continued for another 30 min at room temperature. After that, methyl

iodide (0.47 mL, 7.5 mmol, 1.5 equiv) was added to it at the same temperature and the reaction mixture was heated to reflux at 90 °C for overnight with constant stirring. Upon completion of the reaction (as indicated by TLC), sufficient amount of cold water was added to the reaction mixture and extracted with ethyl acetate (3 x 20 mL). The organic layers were dried over anhydrous Na_2SO_4 and evaporated under reduced pressure. The crude residue was purified by silica gel column chromatography using ethyl acetate in hexane as eluent to afford desired photocatalysts **NCs** in 85-92% yields.

Procedure for the Synthesis of Photocatalyst 13-Methyl-7,8-diphenyl-13*H*-naphtho[2,1-*a*]carbazole-10-carbonitrile (NC5):

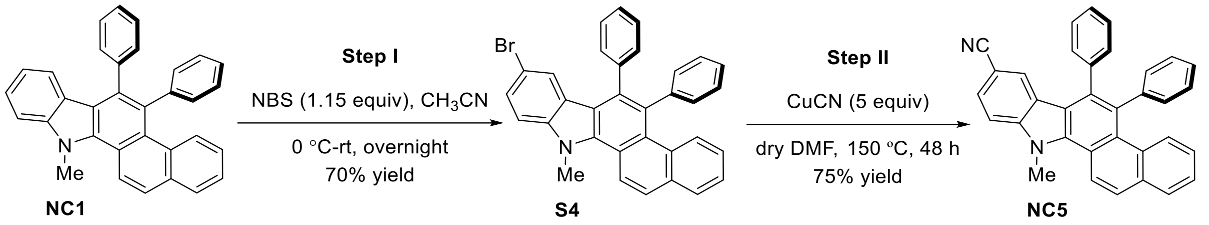

The photocatalyst **NC4** was synthesized according to the modified reported literature procedures.[7a-7b]

Step I: In a 50 mL round bottom flask, 13-methyl-7,8-diphenyl-13*H*-naphtho[2,1-*a*]carbazole **NC1** (433 mg, 1.0 mmol, 1.0 equiv) was dissolved in 15 mL acetonitrile (CH_3CN) at room temperature by stirring for 10 min. After that, reaction mixture was cool down to 0 ºC and *N*-bromosuccinimide (NBS, 204 mg, 1.15 mmol, 1.15 equiv, dissolved in 15 mL acetonitrile) was added dropwise to it at the same temperature, and stirring was continued for another 30 min. Then the reaction mixture was allowed to reach room temperature and stirred for overnight. Upon completion (as monitored by TLC), reaction mixture was quenched with water and extracted with ethyl acetate (3 x 12 mL). The organic layers were dried over anhydrous Na_2SO_4 and evaporated under reduced pressure. The crude product was purified by silica gel column chromatography using 2% ethyl acetate in hexane as eluent to afford the desired product 10-bromo-13-methyl-7,8-diphenyl-13*H*-naphtho[2,1-*a*]carbazole **S4** in 70% yield and used for the next step.

Step II: The *N*-methylated bromocarbazole **S4** (512 mg, 1.0 mmol, 1.0 equiv) was taken in a 30 mL screw capped sealed tube and 10 mL anhydrous *N*,*N*-dimethylformamide (DMF) solvent was added. The resulting suspension was then stirred for 10 min until complete dissolution of starting material **S4**. After that, copper(I) cyanide (CuCN, 448 mg, 5.0 mmol, 5.0 equiv, dissolved in 5 mL anhydrous DMF) was added to the reaction mixture, the sealed tube was

closed with a screw cap and stirring was continued for 5 min at room temperature. Finally, the temperature of the oil bath was gradually increased to 150 °C (over 15 min) before stirring the reaction mixture at the same temperature for 48 h. After completion (as monitored by TLC), the reaction was cool down to room temperature and poured into a cold solution of ferric chloride (7.5 g of $FeCl_3$ dissolved in 23 mL of concentrated hydrochloric acid) and the crude reaction mixture was stirred for another 2 h at room temperature. A brown solid precipitate was generated, collected by filtration and washed with water. Then the residue was dissolved in 30 mL dichloromethane (DCM) and the accumulated filtrate was concentrated under vacuum. Then the crude product was directly purified by silica gel column chromatography using 30% ethyl acetate in hexane as eluent to obtain the desired product 13-methyl-7,8-diphenyl-13*H*-naphtho[2,1-*a*]carbazole-10-carbonitrile **NC5** as light yellow solid (344 mg, 75% yield). **^{1}H NMR** (500 MHz, $CDCl_3$): δ (ppm) 8.65 (d, *J* = 9.2 Hz, 1H), 7.88 (dd, *J* = 8.3, 4.6 Hz, 2H), 7.70 (d, *J* = 8.8 Hz, 1H), 7.62 (dd, *J* = 8.5, 1.0 Hz, 1H), 7.51 (d, *J* = 8.6 Hz, 1H), 7.43 (t, *J* = 7.3 Hz, 1H), 7.35 – 7.30 (m, 3H), 7.21 – 7.18 (m, 3H), 7.12 – 7.09 (m, 4H), 7.06 (t, *J* = 7.8 Hz, 1H), 6.80 (s, 1H), 4.41 (s, 3H). **^{13}C{^{1}H} NMR** (126 MHz, $CDCl_3$): δ (ppm) 144.1, 142.8, 139.8, 137.4, 136.7, 133.0, 132.8, 131.6, 130.08, 129.7, 129.2, 128.4, 128.34, 128.29, 128.2, 127.44, 127.35, 127.0, 126.5, 126.1, 125.0, 123.3, 120.6, 120.3, 119.1, 109.8, 102.4, 35.5. **FTIR**: (neat)/ cm^{-1} = 3055, 2952, 2854, 2220, 1954, 1883,1744, 1610, 1524, 1478, 1441, 1384, 1208, 1128. **HRMS (ESI) *m/z***: $[M+H]^+$ calcd for $C_{34}H_{23}N_2$, 459.1856; found 459.1864.

Procedure for the Synthesis of Photocatalyst 7,8,13-triphenyl-13*H*-naphtho[2,1-*a*]carbazole (NC6):

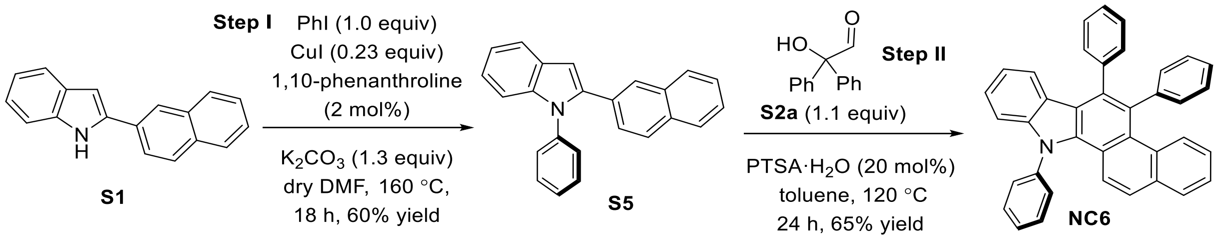

The photocatalyst **NC6** was synthesized according to the modified reported literature procedures.[8,6b]

Step I: In a 50 mL two necked round bottom flask, 2-naphthylindole **S1** (1.2 g, 5.0 mmol, 1.0 equiv), K_2CO_3 (898 mg, 6.5 mmol, 1.3 equiv), copper iodide (CuI, 219 mg, 1.15 mmol, 0.23 equiv), and 1,10-Phenanthroline (20 mg, 0.1 mmol, 2 mol%) were taken. Then the system was evacuated and back-filled with argon (repeated twice). To the reaction flask, 13 mL anhydrous DMF was added under positive argon atmosphere and the solution was stirred for 20 min under

constant purging with argon gas applied through an Ar-filled balloon (to degas the resulting solution). After that iodobenzene (PhI, 0.6 mL, 5 mmol, 1.0 equiv) was added to the reaction mixture at room temperature and the stirring was continued for another 10 min. Then, the reaction mixture was heated to reflux at 160 °C maintaining inert atmosphere while constant stirring for 18 h. Upon completion of the reaction (as monitored by TLC), cold water was added to quench the reaction mixture and extracted with ethyl acetate (3 x 20 mL). The organic layer was then washed with saturated ammonium chloride (aq. NH_4Cl) followed by brine. The organic layer was dried over anhydrous Na_2SO_4 and evaporated under reduced pressure. Then the crude residue was directly purified by silica gel column chromatography using 1% ethyl acetate in hexane as eluent to afford the desired product **S5** and used for the next step (0.96 g, 60% yield).

Step II: In a 15 mL culture tube, **S5** (319 mg, 1.0 mmol, 1.0 equiv) and *p*-toluenesulfonic acid monohydrate (38 mg, PTSA·H_2O, 0.2 mmol, 20 mol%) were dissolved in 30 mL of toluene by stirring at room temperature for 5 min. Then, di-phenyl *α*-hydroxyl aldehyde **S2a** (299 mg, 1.1 mmol, 1.1 equiv) was added and the resulting reaction mixture was heated to reflux at 120 °C for 48 h with constant stirring. Upon completion of the reaction (as monitored by TLC, the products are KMnO4 and DNP active), the crude reaction mixture was diluted with ethyl acetate, transferred to a 25 mL round-bottomed flask and the solvent was evaporated under reduced pressure. The crude residue was purified by using silica gel column chromatography using hexane as eluent to afford 7,8,13-triphenyl-13*H*-naphtho[2,1-*a*]carbazole **NC6** as yellow solid (322 mg, 65% yield). **^{1}H NMR** (500 MHz, $CDCl_3$): δ (ppm) 7.75 (d, *J* = 7.8 Hz, 1H), 7.70 – 7.68 (m, 3H), 7.66 – 7.64 (m, 2H), 7.61 (d, *J* = 7.4 Hz, 2H), 7.50 (d, *J* = 9.1 Hz, 1H), 7.37 – 7.34 (m, 4H), 7.32 – 7.30 (m, 3H), 7.21 –7.20 (m, 6H), 7.03 – 7.00 (m, 1H), 6.98 (t, *J* = 7.5 Hz, 1H), 6.64 (d, *J* = 7.9 Hz, 1H). **^{13}C {^{1}H} NMR** (126 MHz, $CDCl_3$): δ (ppm) 143.6, 143.4, 141.0, 140.9, 136.9, 136.3, 133.0, 132.1, 131.9, 131.7, 130.6, 130.4, 129.0, 128.74, 128.66, 128.3, 128.2, 128.1, 126.8, 126.23, 126.21, 125.6, 125.5, 124.7, 123.5, 122.2, 121.4, 120.4, 120.3, 120.1, 110.3. **FTIR**: (neat)/ cm^{-1} = 3746, 3645, 3049, 2325, 2163, 2080, 1966, 1907, 1826, 1744, 1591, 1496, 1452,1389, 1316, 1204, 1140. **HRMS (ESI) *m/z***: $[M+H]^+$ calcd for $C_{38}H_{26}N$, 496.2060; found 496.2060.

Synthesis of Bromoarene Precursors: Bromoarenes were used as received from commercial sources without further purification. Bromoarene **1n**, **1s** and **1t** were synthesised according to the literature procedures.[9a-9c]

General Procedure for the Synthesis of *ortho*-Bromo Substituted *N*-Aryl Benzamide Derivatives 4a-4m (GP-II):[10a,10b]

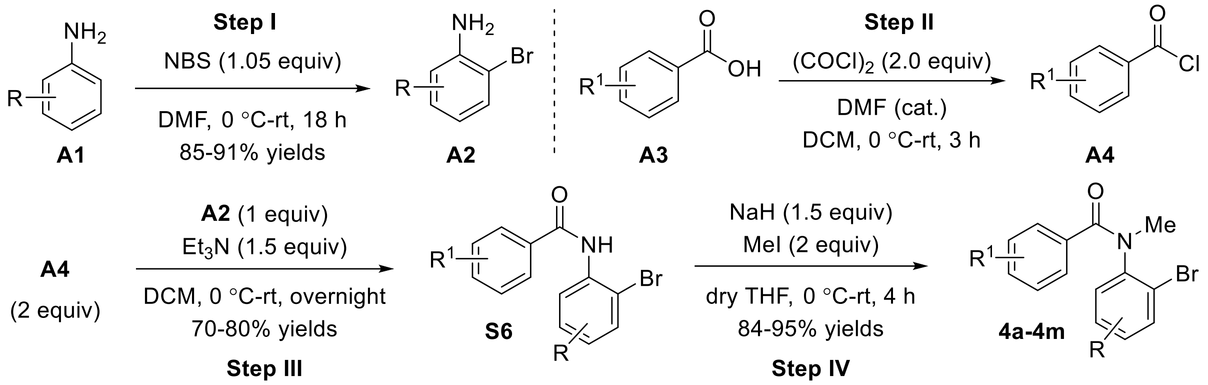

Step I: According to literature procedures, in a 50 mL round bottom flask, aniline derivatives **A1** (5.0 mmol, 1.0 equiv) were dissolved in 10 mL *N*, *N*-dimethylformamide (DMF) by stirring at room temperature for 10 min. After that, the resulting solution was cool down to 0 ºC and *N*-bromosuccinimide (NBS, 0.94 g, dissolved in 5 mL DMF) was added portion wise and stirring was continued for another 30 min at the same temperature. The reaction mixture was allowed to reach room temperature gradually and stirring was continued for 18 h at the same temperature. Upon completion of the reaction (as monitored by TLC), the reaction mixture was quenched with cold water and extracted with ethyl acetate (3 x 10 mL). The organic layers were dried over anhydrous Na_2SO_4 and evaporated under reduced pressure. The crude residue was purified by silica gel chromatography by using ethyl acetate in hexane as eluent to afford the *ortho*-bromoaniline derivatives **A2** in 85-91% yields.

Step II: In a 25 mL round bottom flask, aryl carboxylic acid derivative **A3** (3.0 mmol, 1.0 equiv) was dissolved in dichloromethane (DCM) (9.9 mL, 0.3 M) and catalytic amount of *N*, *N*-dimethylformamide (DMF, 3 drops) was added dropwise and stirring was continued for 10 min. Then the solution was cool down to 0 ºC and to this mixture, oxalyl chloride (0.53 mL, 6.0 mmol, 2.0 equiv) was added dropwise at the same temperature. After that, the reaction mixture was allowed to reach room temperature and stirring was continued for another 3 h. Upon completion of the reaction (as monitored by TLC), the resulting reaction mixture was concentrated under reduced pressure to afford the acyl chloride derivatives **A4** as a yellow slurry which was used for the next step without further purification.

Step III: In a 25 mL round bottom flask, *ortho*-bromoaniline derivative **A2** (1.5 mmol, 1.0 equiv) was dissolved in 5 mL of DCM at room temperature by stirring for 10 min. After that reaction mixture was cool down to 0 ºC, triethylamine (0.3 mL, 2.25 mmol, 1.5 equiv) was

added dropwise and stirring was continued for another 30 min at same temperature. Then, previously prepared acyl chloride derivative **A4** (3 mmol, 2.0 equiv, dissolved in 5 mL of DCM) was added dropwise at 0 ºC to the reaction mixture while stirring, the mixture was allowed to reach room temperature and stirring was continued for overnight. After complete consumption of the *ortho*-bromoaniline derivative **A2** (as monitored by TLC), reaction mixture was quenched by addition of saturated aq. NH_4Cl solution and organic layer was extracted with DCM (2 x 10 mL). The accumulated organic layers were dried over anhydrous Na_2SO_4 and evaporated under reduced pressure. The crude residue was purified by silica gel chromatography by using ethyl acetate in hexane as eluent to afford the desired products **S6** in 70-80% yields.

Step IV: In a 50 mL two necked round bottom flask, *N*-(2-bromophenyl)benzamide derivatives **S6** (1.5 mmol, 1.0 equiv) were taken, and the flask was evacuated and backfilled with argon (repeated twice). After that 10 mL of anhydrous tetrahydrofuran (THF) was added to it under positive argon pressure and the reaction mixture was cool down to 0 ºC. Then, NaH (60% dispersion in mineral oil, 120 mg, 5.0 mmol, 2.0 equiv) was added portion wise to the reaction mixture under positive argon pressure while stirring at the same temperature. After complete addition of NaH, the resulting mixture was stirred at room temperature for 30 min. Then, the reaction mixture was cool down to 0 ºC and iodomethane (0.2 mL, 3.3 mmol, 2.2 equiv) was added. The reaction mixture was then allowed to reach room temperature and stirring was continued for another 3 h. Upon completion of the reaction (as monitored by TLC), the reaction mixture was quenched with saturated aq. NH_4Cl solution and extracted with ethyl acetate (3 x 10 mL). The organic layers were dried over anhydrous Na_2SO_4 and evaporated under reduced pressure. The crude residue was purified by silica gel chromatography by using ethyl acetate in hexane as eluent to afford *ortho*-bromo substituted *N*-aryl benzamide derivatives **4a-4m** in 84-95% yields.

The benzamide derivative **4p** was synthesized according to GP II by using benzylbromide (0.27 mL, 2.25 mmol, 1.5 equiv).[10c]

Synthesis of Imine Precursors: Aldehydes and ketones were used as received from commercial sources without further purification. All the imines were synthesised according to the literature procedures.[11]

Synthesis of Sulfonylated Heteroaromatic and Peptide Derivatives: All the sulfonylated heteroaromatic derivatives and sulfone protected tryptophan containing peptides were synthesised according to the literature procedures.[12]

General Procedure for Borylation of Aryl Halides (GP-III):

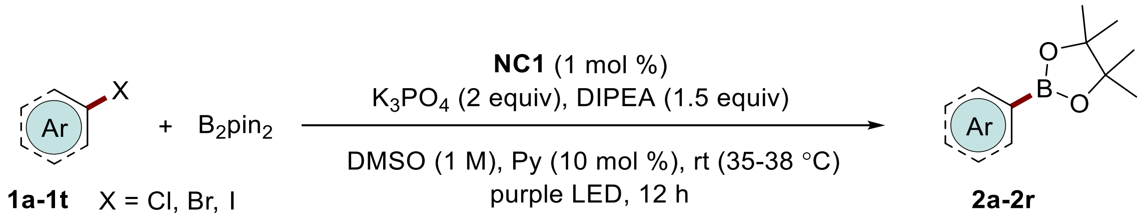

Aryl halide **1** (if solid, 0.5 mmol, 1.0 equiv), photocatalyst **NC1** (1.1 mg, 0.005 mmol, 1 mol%), bis(pinacolato)diboron (B_2pin_2, 254 mg, 1.0 mmol, 2.0 equiv), and tripotassium phosphate (K_3PO_4, 212 mg, 1.0 mmol, 2.0 equiv) were taken in an oven dried 12 mL glass reaction tube, and it was capped with rubber septum. Then, the reaction vessel was evacuated and backfilled with argon (repeated twice). After that, aryl halide **1** (if liquid, 0.5 mmol, 1.0 equiv), *N*,*N*-disopropylethylamine (DIPEA, 0.13 mL, 0.75 mmol, 1.5 equiv) and pyridine (5 µL, 10 mol%) were added successively by using Hamilton syringe under positive argon pressure. Anhydrous degassed 0.5 mL DMSO was added to the reaction vessel using syringe. Finally, the tube was placed 4 cm away from Kessil purple light (390 nm) and the reaction mixture was stirred under the irradiation of 390 nm at room temperature (maintaining ~35-38 °C temperature by employing one cooling fan) for 12 h. Upon complete consumption of starting material (as monitored by TLC), the reaction mixture was quenched with saturated aq. NH_4Cl solution and the organic layer was extracted with ethyl acetate. Then, the organic solvent was evaporated under reduced pressure. The crude residue was purified by column chromatography on silica gel by using ethyl acetate in hexane as eluents to afford borylated products **2a-2r** in 30-90% yields.

Table S2: Optimization of Borylation Reaction Conditions[a]

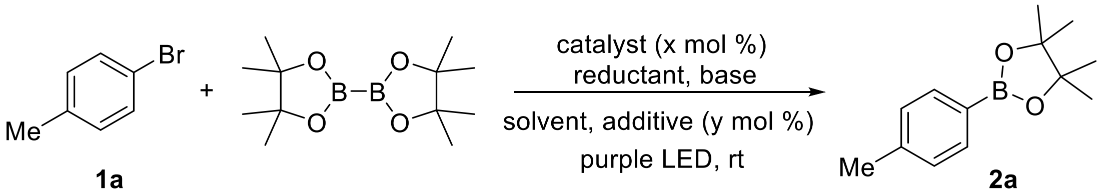

entry	catalyst (x mol%)	solvent	reductant	additive (y mol%)	base	time (h)	yield of **2a** [%][b]
1	NC1 (3)	DCM	$Na_2C_2O_4$	4-CN-Py (10)	K_3PO_4	48	nd
2	NC1 (3)	DMSO	$Na_2C_2O_4$	4-CN-Py (10)	K_3PO_4	48	63
3	NC1 (3)	DMF	$Na_2C_2O_4$	4-CN-Py (10)	K_3PO_4	48	61
4	NC1 (3)	DMSO	Hantzsch Ester	4-CN-Py (10)	K_3PO_4	48	nd
5	NC1 (3)	DMSO	DIPEA	4-CN-Py (10)	K_3PO_4	48	78
6	NC1 (3)	DMSO	NEt_3	4-CN-Py (10)	K_3PO_4	48	69
7	NC1 (3)	DMSO	DIPEA	4-CN-Py (10)	K_2HPO_4	48	47
8	NC1 (3)	DMSO	DIPEA	4-CN-Py (10)	KF	48	63
9	NC1 (3)	DMSO	DIPEA	4-CN-Py (10)	Cs_2CO_3	48	74
10	NC1 (1)	DMSO	DIPEA	Py (10)	NaOMe	48	76
11	NC1 (3)	DMSO	DIPEA	Py (10)	K_3PO_4	48	83
12	NC1 (3)	DMSO	DIPEA	–	K_3PO_4	48	59
13	NC1 (3)	DMSO	DIPEA	Py (100)	K_3PO_4	48	60
14	NC1 (3)	DMSO	DIPEA	4-CN-Py (100)	K_3PO_4	48	nd
15	NC1 (3)	DMSO	DIPEA	Py (10)	K_3PO_4	12	93
16	NC1 (3)	DMSO	DIPEA	Py (10)	K_3PO_4	24	86
17	NC1 (3)	DMSO	DIPEA	Py (10)	K_3PO_4	36	82
18	NC3 (3)	DMSO	DIPEA	Py (10)	K_3PO_4	12	74
19	NC4 (3)	DMSO	DIPEA	Py (10)	K_3PO_4	12	75
20	NC2 (3)	DMSO	DIPEA	Py (10)	K_3PO_4	12	76
21	NC1 (0.5)	DMSO	DIPEA	Py (10)	K_3PO_4	12	73
**22**	**NC1 (1)**	**DMSO**	**DIPEA**	**Py (10)**	**K_3PO_4**	**12**	**88** [72][c]
23[d]	NC1 (1)	DMSO	DIPEA	Py(10)	K_3PO_4	12	74

24[e]	NC1 (1)	DMSO	DIPEA	Py(10)	K_3PO_4	12	69
25	NC2 (2)	DMSO	DIPEA	Py (10)	K_3PO_4	12	85
26	NC1 (1)	DMSO	DIPEA	Py (25)	K_3PO_4	12	72
27	NC1 (1)	DMSO	DIPEA	Py (50)	K_3PO_4	12	56
28[f]	NC1(1)	DMSO	DIPEA	Py (10)	K_3PO_4	12	50
29	–	DMSO	DIPEA	Py (10)	K_3PO_4	12	4
30	NC1 (1)	DMSO	–	Py (10)	K_3PO_4	12	57
31	NC1(1)	DMSO	DIPEA	Py (10)	–	12	29
32[g]	NC1(1)	DMSO	DIPEA	Py (10)	K_3PO_4	12	51
33[h]	NC1 (1)	DMSO	DIPEA	Py (10)	K_3PO_4	12	nd

[a]Reaction conditions: **1a** (0.3 mmol), B_2Pin_2 (0.6 mmol), reductant (1.5 equiv), base (2.0 equiv), solvent (0.3 mL, 1 M), nd = not detected. [b]NMR yields by taking mesitylene as internal standard. [c]Isolated yields. [d]Using 1 equiv of DIPEA. [e]Using 2 equiv of DIPEA. [f]1 Equiv of B_2Pin_2 and 1 equiv of K_3PO_4 has been used. [g]Under air. [h]Without degassing the solvent.

General Procedure for the Phosphorylation of Aryl Halides (GP-IV):

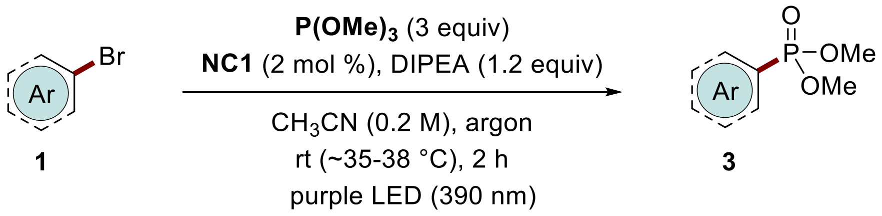

Aryl bromide **1** (if solid, 0.2 mmol, 1.0 equiv) and photocatalyst **NC1** (1.7 mg, 0.004 mmol, 2 mol%) were taken in an oven dried 12 mL glass reaction tube and it was capped with a rubber septum. Then the reaction vessel was evacuated and backfilled with argon (repeated twice). After that, aryl bromide **1** (if liquid, 0.2 mmol, 1.0 equiv) and trimethylphosphite (70 µL, 0.6 mmol, 3 equiv) were successively added using Hamilton syringe under positive argon pressure. Then 1 mL anhydrous degassed acetonitrile followed by *N*, *N*-diisopropylethylamine (42 µL 0.24 mmol,1.2 equv) were successively added by Hamilton syringe. Finally, the tube was placed 4 cm away from Kessil purple LED light (390 nm), and stirred vigorously under the irradiation of 390 nm purple LED light at room temperature (maintaining ~35-38 °C temperature by employing one cooling fan). Upon complete consumption of bromide (as monitored by TLC), the reaction mixture was diluted with ethyl acetate, the crude reaction mixture was transferred to a round bottom flask and organic solvent was evaporated under

reduced pressure. The crude residue was purified by silica gel column chromatography by using ethyl

acetate in hexane as eluent to afford the desired phosphonate products **3a-3f** in 49-90% yields.

General Procedure for the Reductive C-C Coupling of *ortho*-Bromo Substituted N-Phenyl Benzamide (GP-V):

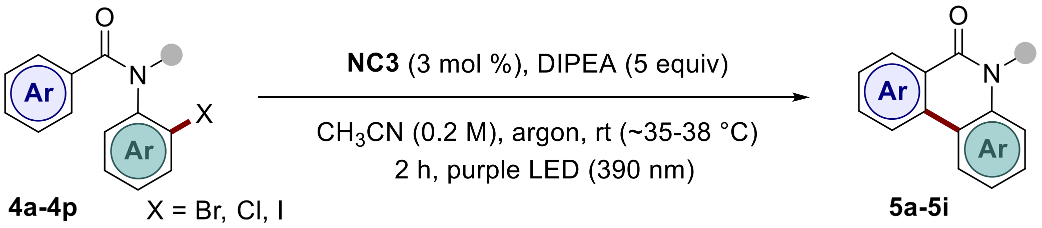

The halogen substituted *N*-methylbenzamide **4** (0.5 mmol, 1.0 equiv) and photocatalyst **NC3** (0.015 mmol, 3 mol%) were taken in an oven dried 12 mL glass reaction tube and it was capped with a rubber septum. Then the reaction vessel was evacuated and backfilled with argon (repeated twice). After that, 2.5 mL of anhydrous degassed acetonitrile and *N*, *N*-diisopropylethylamine (0.43 mL, 2.5 mmol, 5.0 equiv) were successively added using syringe. Finally, the tube was placed 4 cm away from purple LED light (390 nm), and stirred vigorously under the irradiation of 390 nm purple LED light at room temperature (maintaining ~35-38 °C temperature by employing one cooling fan). Upon complete consumption of starting material (as monitored by TLC), the reaction mixture was diluted with ethyl acetate, the crude reaction mixture was transferred to a round bottom flask and organic solvent was evaporated under reduced pressure. The crude residue was purified by silica gel column chromatography by using ethyl acetate in hexane as eluent to afford the desired phenanthridine products **5a-5i** in 55-89% yields.

Table S3: **Optimization of the C-C coupling Reaction Conditions**:[a]

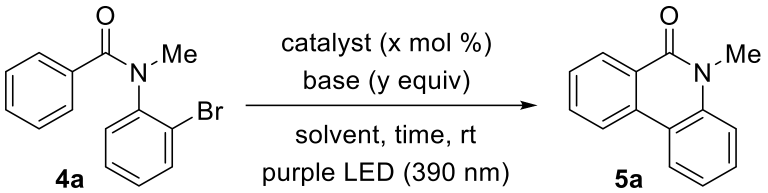

entry	catalyst (x mol%)	base (y equiv)	solvent	time	yield (%) [b]
1	NC1 (2)	NBu_3 (5)	CH_3CN	24	20
2	NC1 (2)	DIPEA (1)	CH_3CN	2	53
3	NC1 (2)	DIPEA (1)	CH_3CN	10	30
4	NC1 (2)	–	CH_3CN	10	0
5	–	DIPEA (1)	CH_3CN	10	0
6	NC1 (2)	DIPEA (0.5)	CH_3CN	12	<5
7	NC1 (2)	DIPEA (0.25)	CH_3CN	12	<5
8	NC1 (2)	DIPEA (9)	CH_3CN	2	55
9	NC1 (2)	DIPEA (2)	CH_3CN	2	66
10	NC1 (2)	DIPEA (5)	CH_3CN	2	69
11	NC1 (2)	NEt_3 (5)	CH_3CN	2	0
12	NC3 (2)	DIPEA (5)	CH_3CN	2	74
13	NC2 (2)	DIPEA (5)	CH_3CN	2	56
14	NC4 (2)	DIPEA (5)	CH_3CN	2	71
15	NC5 (2)	DIPEA (5)	CH_3CN	2	45
16	NC6(2)	DIPEA (5)	CH_3CN	2	73
17	NC3 (2)	NEt_3 (5)	CH_3CN	2	0
18	NC3 (2)	DIPEA (5)	DMF	2	70
19	NC3 (2)	DIPEA (2)	DMF	2	49
20	NC3 (2)	DIPEA (2)	CH_3CN	2	71
21	**NC3 (3)**	**DIPEA (5)**	**CH_3CN**	**2**	**91**
22	NC3 (1)	DIPEA (5)	CH_3CN	2	77

[a]Reaction conditions: **4a** (0.2 mmol), anhydrous degassed solvent (1 mL, 0.2 M). [b]NMR yield by taking mesitylene as internal standard.

General Procedure for the Reductive Dimerization of Aldehydes and Ketones (GP-VI):

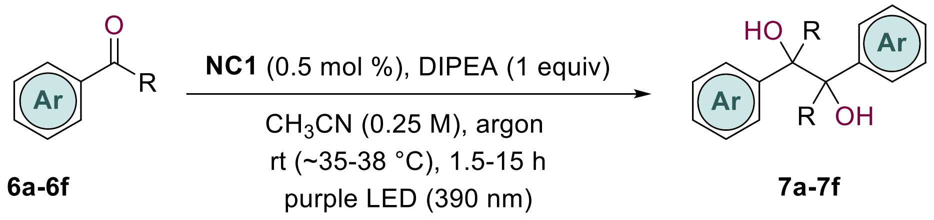

Aldehyde or ketone **6** (if solid, 0.5 mmol, 1.0 equiv) and photocatalyst **NC1** (1.1 mg, 0.0025 mmol, 0.5 mol%) were taken in a 12 mL oven dried glass reaction tube and it was capped with a rubber septum. Then the reaction vessel was evacuated and backfilled with argon (repeated twice). After that, aldehyde or ketone (if liquid, 0.5 mmol, 1.0 equiv) and *N*, *N*-diisopropylethylamine (87μL, 0.5 mmol, 1.0 equiv) were successively added using Hamilton syringe under positive argon pressure. Then 2 mL degassed anhydrous acetonitrile was added via syringe. Finally, the tube was placed 4 cm away from Kessil purple LED light (390 nm), and stirred vigorously under the irradiation of 390 nm purple LED light at room temperature (maintaining ~35-38 °C temperature by employing one cooling fan). Upon complete consumption of aldehyde or ketone (as monitored by TLC), the reaction mixture was diluted with ethyl acetate, the crude reaction mixture was transferred to a round bottom flask and organic solvent was evaporated under reduced pressure. The crude residue was purified by silica gel column chromatography by using ethyl acetate in hexane as eluent to afford the desired pinacol products **7a-7i** in 63-99% yields. The *meso:dl* diastereoisomeric ratio of the various products were evaluated by ^{1}H NMR analysis of the hydroxyl and benzylic proton (for aldehydes)/ methyl proton (for ketones) and comparing their chemical shift values with the reported data.

General Procedure for the Reductive Dimerization of Imines (GP-VII):

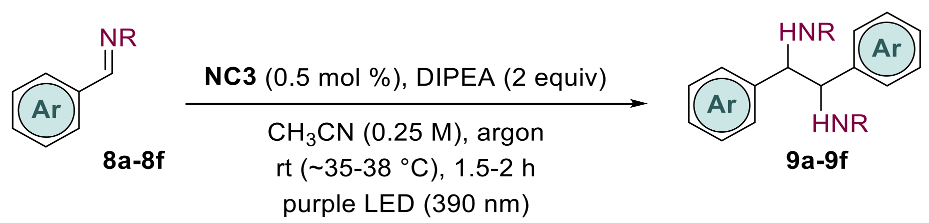

Imine **8** (0.5 mmol, 1.0 equiv) and photocatalyst **NC3** (1.2 mg, 0.0025 mmol, 0.5 mol%) were taken in an oven dried 12 mL glass reaction tube and it was capped with a rubber septum. Then the reaction vessel was evacuated and backfilled with argon (repeated twice). After that, 2 mL degassed anhydrous acetonitrile and *N*, *N*-diisopropylethylamine (0.180 μL, 1.0 mmol, 2.0

equiv) were successively added via syringe under positive argon pressure. Finally, the tube was placed 4 cm away from Kessil purple LED light (40 W), and stirred vigorously under the irradiation of 390 nm purple LED light at room temperature (maintaining ~35-38 °C temperature by employing one cooling fan). Upon complete consumption of imine (as monitored by TLC), the crude reaction mixture was diluted with ethyl acetate, the crude mixture was transferred to a round bottom flask and organic solvent was evaporated under reduced pressure. The crude residue was purified by column chromatography on neutral activated alumina to afford the desired imino-pinacol products **9a-9f** in 52-96% yield. The *meso:dl* diastereoisomeric ratios of the various products were evaluated by ^{1}H NMR analysis of the benzylic proton and comparing their chemical shift values with the reported data.

Table S4: **Optimization of the Piancol Coupling Reaction Conditions**:[a]

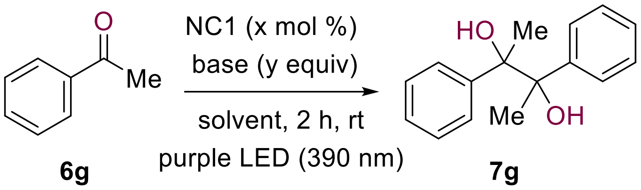

6g **7g**

entry	catalyst (x mol%)	base (y equiv)	solvent	yield (%)[d]
1	NC1 (1)	DIPEA (1)	CH_3CN	77
**2**	**NC1 (0.5)**	**DIPEA (1)**	**CH_3CN**	**76**
3	NC1 (0.5)	NEt_3 (1)	CH_3CN	30
4	NC1 (0.5)	Hantzsch ester (1)	CH_3CN	30
5	NC1 (0.5)	DIPA (1)	CH_3CN	0
6	NC1 (0.5)	DABCO (1)	CH_3CN	0
7	NC1 (0.5)	DIPEA (1)	DCM	30
8	NC1 (0.5)	DIPEA (1)	DMF	41
9	NC1 (0.5)	DIPEA (1)	DMSO	38
10	-	DIPEA (1)	CH_3CN	0
11	NC1 (0.5)	-	CH_3CN	0
12[b]	NC1 (0.5)	DIPEA (1)	CH_3CN	0
13[c]	NC1 (0.5)	DIPEA (1)	CH_3CN	0

[a]Reaction conditions: ketone **6g** (0.5 mmol), anhydrous degassed solvent (2.0 mL, 0.25 M). [b]Under air. [c]Without degassing the solvent. [d]Isolated yields.

Table S5: Catalyst Screening for Pinacol and Imino-Pinacol Reaction:

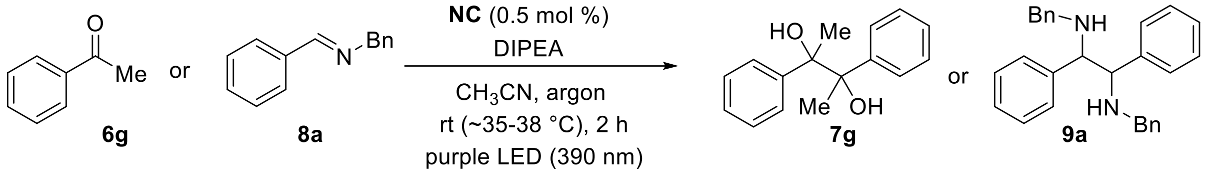

catalyst	**NC1**	**NC2**	**NC3**	**NC4**	**NC5**	**NC6**
meso:dl[a]	1:1.11	1:1.17	1:1.18	1:1.15	1:1.11	1:1.35
yield[a]	76%	61%	71%	62%	67%	65
meso:dl[b]	1:1.76	1:2.08	1:1.82	1:3.03	1:1.91	1:1.70
yield[b]	68%	73%	89%	66%	82	63%

Reaction conditions: *Pinacol reaction*:[a] Ketone **6g** (0.5 mmol), NC catalyst (0.0025 mmol, 0.5 mol%), DIPEA (0.5 mmol, 1.0 equiv), anhydrous degassed CH_3CN (2.0 mL, 0.25 M). *Imino-pinacol reaction:* [b]Imine **8a** (0.5 mmol), NC catalyst (0.0025 mmol, 0.5 mol%), DIPEA (1.0 mmol, 2.0 equiv), anhydrous degassed CH_3CN (2.0 mL, 0.25 M).

General Procedure for the Reductive Desulfonylation of *N*-Sulfonyl Heteroaromatic Compounds (GP-VIII):

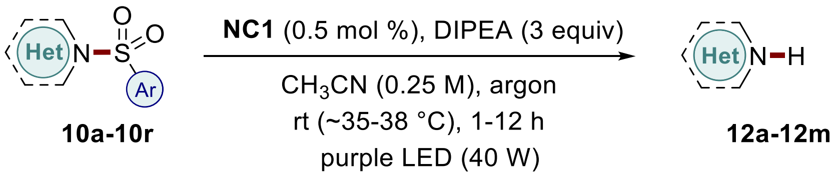

The N-sulfonyl heteroaromatic compound **10** (0.5 mmol, 1.0 equiv) and photocatalyst **NC1** (1.1 mg, 0.0025 mmol, 0.5 mol%) were taken in an oven dried 10 mL glass reaction tube and it was capped with a rubber septum. Then the reaction vessel was evacuated and backfilled with argon (repeated twice). After that, 2 mL of degassed anhydrous acetonitrile and *N*, *N*-diisopropylethylamine (0.26 mL, 1.5 mmol, 3.0 equiv) were successively added via syringe under positive argon pressure. Finally, the tube was placed 4 cm away from Kessil purple LED light and stirred vigorously under the irradiation of 390 nm purple LED light at room temperature (maintaining ~35-38 °C temperature by employing one cooling fan). Upon complete consumption of starting material (as monitored by TLC), the crude reaction mixture was diluted with ethyl acetate, the crude mixture was transferred to a round bottom flask and organic solvent was evaporated under reduced pressure. The crude residue was purified by

column chromatography on silica gel by using ethyl acetate in hexane to afford desulfonylated heteroaromatics **12a-12m** in 75-97%yields.

Table S6: **Optimization of the Desulfonylation Reaction Conditions**:[a]

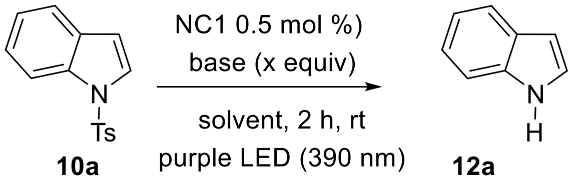

entry	base (x equiv)	solvent	yield (%)[b]
1	DIPEA (1)	CH_3CN	72
**2**	**DIPEA (3)**	**CH_3CN**	**90**
3	NEt_3 (3)	CH_3CN	30
4	Hantzsch ester (3)	CH_3CN	32
5	DIPA (3)	CH_3CN	0
6	DABCO (3)	CH_3CN	0
7	DIPEA (3)	DCM	25
8	DIPEA (3)	DMF	40
9	DIPEA (3)	DMSO	45
10	-	CH_3CN	0
11[c]	DIPEA (3)	CH_3CN	0
12[d]	DIPEA (3)	CH_3CN	0
13[e]	DIPEA (3)	CH_3CN	0

[a]Reaction conditions: **10a** (0.5 mmol), NC1 (0.0025 mmol), and anhydrous degassed solvent (2.0 mL, 0.1 M). [b]Isolated yields. [c]Under air. [d]Without degassing the solvent. [e]In the absence of NC1.

General Procedure for the Reductive Desulfonylation of *N*-Sulfonylated Peptides (GP-IX):

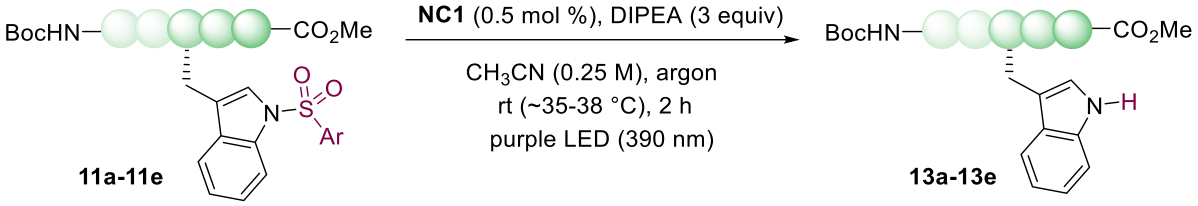

The sulfonyl protected tryptophan containing peptide **11** (0.1 mmol, 1.0 equiv) and photocatalyst **NC1** (0.2 mg,0.0005 mmol, 0.5 mol%) were taken in an oven dried 12 mL glass reaction tube and it was capped with a rubber septum. Then the reaction vessel was evacuated and backfilled with argon (repeated twice). After that, degassed anhydrous acetonitrile (1.5 mL) and *N*, *N*-diisopropylethylamine (52 µL, 0.3 mmol, 3.0 equiv) were successively added via syringe under positive argon pressure. Finally, the tube was placed 4 cm away from Kessil purple LED light, and stirred vigorously under the irradiation of 390 nm purple LED light at room temperature (maintaining ~35-38 °C temperature by employing one cooling fan). Upon complete consumption of starting material (as monitored by TLC), the crude reaction mixture was diluted with ethyl acetate, the crude mixture was transferred to a round bottom flask and organic solvent was evaporated under reduced pressure. The crude residue was purified by column chromatography on silica gel by using methanol in dichloromethane to afford desulfonylated peptide products **13a-13e** in 89-91% yields.

Gram-Scale Synthesis and Catalyst Recovery Experiment for Borylation Reaction:

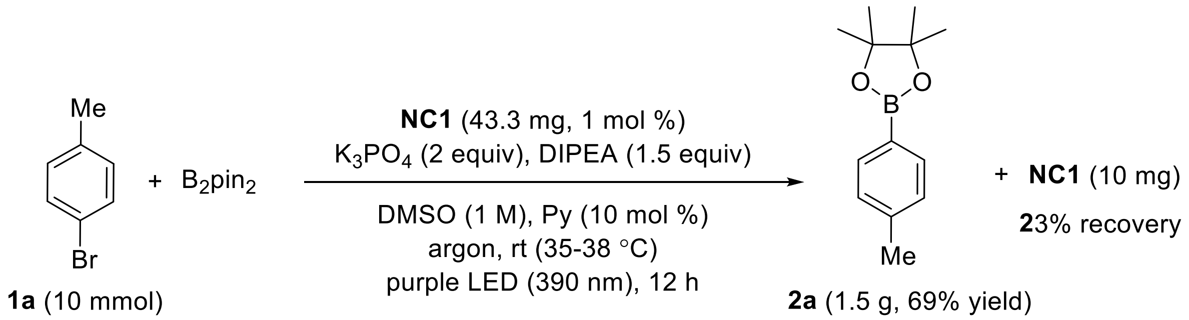

Photocatalyst **NC1** (43.3 mg, 0.1 mmol, 1 mol%), bis(pinacolato)diboron (B_2pin_2, 5.3 g, 20.0 mmol, 2.0 equiv), and tripotassium phosphate (K_3PO_4, 4.2 g, 20.0 mmol, 2.0 equiv) were taken in an oven dried 25 mL round bottom flask, and it was capped with rubber septum. Then, the reaction vessel was evacuated and backfilled with argon (repeated twice). After that, 1-bromo-4-methylbenzene **1a** (1.23 mL, 10.0 mmol, 1.0 equiv) and *N*, *N*-disopropylethylamine (DIPEA, 2.61 mL, 15.0 mmol, 1.5 equiv) and pyridine (0.1 mL, 10 mol%) were added successively by

Hamilton syringe under positive argon pressure. Anhydrous degassed 10 mL DMSO was added to the reaction vessel using syringe. Finally, the tube was placed 4 cm away from Kessil purple light (390 nm) and the reaction mixture was stirred under the irradiation of 390 nm at room temperature (maintaining ~35-38 °C temperature by employing one cooling fan) for 12 h. Upon complete consumption of starting material (as monitored by TLC), the reaction mixture was quenched with saturated aq. NH_4Cl solution and the organic layer was extracted with ethyl acetate. Then, the organic solvent was evaporated under reduced pressure. The crude residue was isolated after column chromatography on silica gel using 1% ethyl acetate/hexane as eluent to afford the borylated product **2a** (white solid, 1.5 g, 69% yield). In a second fractions, the catalyst NC1 was recovered by further eluting the column by using 2% ethyl acetate in hexane as eluent (light yellow solid, 10 mg, 23% recovered).

Gram-Scale Synthesis and Catalyst Recovery Experiment for C-C Coupling Reaction:

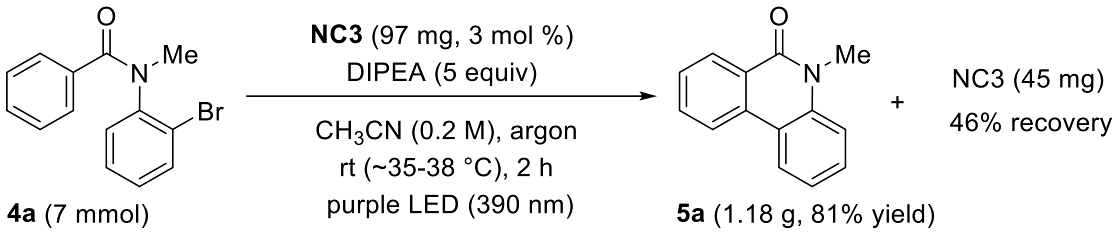

N-(2-Bromophenyl)-*N*-methylbenzamide **4a** (2 g, 7.0 mmol, 1.0 equiv) and photocatalyst **NC3** (97.3 mg, 0.21 mmol, 3 mol%) were taken in an oven dried 50 mL round bottom flask and it was capped with a rubber septum. Then the reaction vessel was evacuated and backfilled with argon (repeated twice). After that, degassed anhydrous acetonitrile (35 mL) and *N*, *N*-diisopropylethylamine (6.1 mL, 35 mmol, 5.0 equiv) were successively added using syringe under positive argon pressure. Finally, the tube was placed 4 cm away from purple LED light (390 nm), and stirred vigorously under the irradiation of 390 nm purple LED light at room temperature (maintaining ~35-38 °C temperature by employing one cooling fan). Upon complete consumption of starting material (as monitored by TLC), the reaction mixture was diluted with ethyl acetate, organic solvent was evaporated under reduced pressure. The catalyst **NC3** was recovered after column chromatography on silica gel using 2% ethyl acetate in hexane as eluent (light yellow solid, 45 mg, 46% recovered). In another fractions the desired product **5a** was also isolated by further eluting the column using 10% ethyl acetate in hexane as eluent (white solid, 1.18 g, 81% yield).

Gram-Scale Synthesis and Catalyst Recovery Experiment for Pinacol Coupling Reaction:

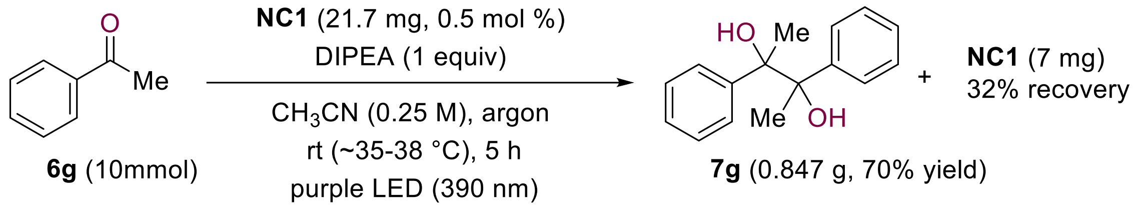

Photocatalyst **NC1** (21.65 mg, 0.05 mmol, 0.5 mol%) was taken in a 50 mL round bottom flask and it was capped with a rubber septum. Then the reaction vessel was evacuated and backfilled with argon (repeated twice). After that, ketone (1.16 mL, 10 mmol, 1.0 equiv) and *N*, *N*-diisopropylethylamine (1.74 mL, 10 mmol, 1.0 equiv) were successively added using syringe under positive argon pressure. Then 25 mL degassed anhydrous acetonitrile was added by syringe. Finally, the tube was placed 4 cm away from Kessil purple LED light (40 W), and stirred vigorously under the irradiation of 390 nm purple LED light at room temperature (maintaining ~35-38 °C temperature by employing one cooling fan). Upon complete consumption of aldehyde or ketone (as monitored by TLC), the reaction mixture was diluted with ethyl acetate and organic solvent was evaporated under reduced pressure. The catalyst NC1 was recovered after column chromatography on silica gel using 2% ethyl acetate in hexane as eluent (light yellow solid, 7 mg, 32% recovered). In a second fractions, the desired product **7g** was isolated as white solid by further eluting the column using 10% ethyl acetate in hexane as eluent (0.847 g, 70% yield).

Gram-Scale Synthesis and Catalyst Recovery Experiment for Imino-pinacol Coupling Reaction:

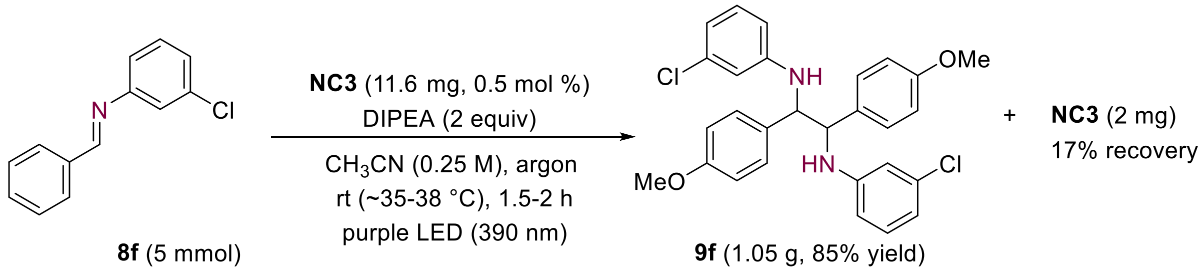

Imine **8f** (1.23 g, 5.0 mmol, 1.0 equiv) and photocatalyst **NC3** (11.6 mg, 0.025 mmol, 0.5 mol%) were taken in a 12 mL oven dried glass reaction tube and it was capped with a rubber septum. Then the reaction vessel was evacuated and backfilled with argon (repeated twice). Then 13 mL degassed anhydrous acetonitrile and *N*, *N*-diisopropylethylamine (1.74 mL, 10.0 mmol, 2.0 equiv) were added by syringe under positive argon pressure. Finally, the tube was placed 4 cm away from Kessil purple LED light, and stirred vigorously under the irradiation

of 390 nm purple LED light at room temperature (maintaining ~35-38 °C temperature by employing one cooling fan). Upon complete consumption of aldehyde or ketone (as monitored by TLC), the reaction mixture was diluted with ethyl acetate and organic solvent was evaporated under reduced pressure. The catalyst NC3 was recovered after column chromatography on silica gel using 2% ethyl acetate in hexane as eluent (light yellow solid, 3 mg, 17% recovered). In a second fractions, the desired product **9f** was isolated as white solid by further eluting the column using 10% ethyl acetate in hexane as eluent (1.05 g, 85% yield).

Gram-Scale Synthesis and Catalyst Recovery Experiment for Desulfonylation Reaction:

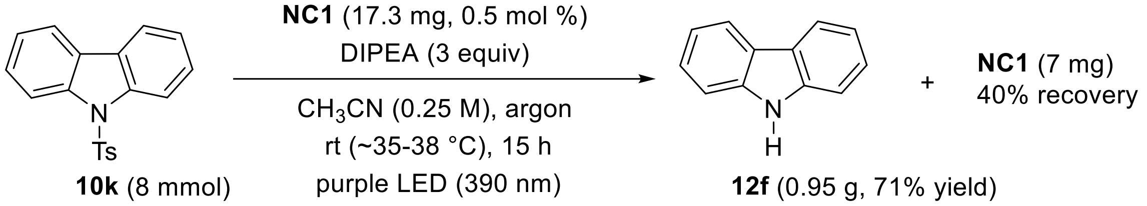

10k (2.6 g, 8.0 mmol, 1.0 equiv) and photocatalyst **NC1** (17.3 mg, 0.04 mmol, 0.5 mol%) were taken in an oven dried 50 mL round bottom flask and it was capped with a rubber septum. Then the reaction vessel was evacuated and backfilled with argon (repeated twice). After that, 32 mL degassed anhydrous acetonitrile and *N*, *N*-diisopropylethylamine (4.18 mL, 24.0 mmol, 3.0 equiv) were successively added using syringe under positive argon pressure. Finally, the tube was placed 4 cm away from Kessil purple LED light and stirred vigorously under the irradiation of 390 nm purple LED light at room temperature (maintaining ~35-38 °C temperature by employing one cooling fan). Upon complete consumption of starting material (as monitored by TLC), the crude reaction mixture was diluted with ethyl acetate and organic solvent was evaporated under reduced pressure. The catalyst NC1 was recovered after column chromatography on silica gel using 2% ethyl acetate in hexane as eluent (light yellow solid, 7 mg, 40% recovered). In a second fractions, the desired product **12f** was isolated as white solid by further eluting the column using 10% ethyl acetate in hexane as eluent (0.95 g, 71% yield).

13-Methyl-7,8-diphenyl-13H-naphtho[2,1-a]carbazole (***NC1***)***:***[6b] The titled compound **NC1** was synthesized according to the **GP-I**. The product **NC1** was isolated after column chromatography using 2% ethyl acetate in hexane as eluent (light yellow solid, 1.95 g, 90% yield). **^{1}H NMR** (400 MHz, $CDCl_3$): δ (ppm) 8.72 (d, *J* = 9.1 Hz, 1H), 7.88 (t, *J* = 7.5 Hz, 2H), 7.73 (d, *J* = 8.7 Hz, 1H), 7.53 (d, *J* = 8.2 Hz, 1H), 7.47 – 7.41 (m, 2H), 7.35 – 7.32 (m,

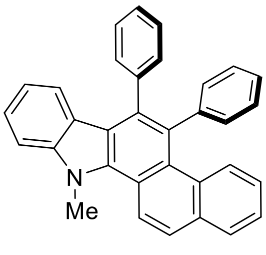

3H), 7.24 – 7.15 (m, 7H), 7.07 (t, J = 7.8 Hz, 1H), 6.98 (t, J = 7.5 Hz, 1H), 6.63 (d, J = 7.9 Hz, 1H), 4.42 (s, 3H).

8-(3-Methoxyphenyl)-13-methyl-7-phenyl-13H-naphtho[2,1-a]carbazole (NC2): The titled compound **NC2** was synthesized according to the **GP-I**. The product **NC2** was isolated after column chromatography using 2% ethyl acetate in hexane as eluent (light yellow solid, 2.1 g, 92% yield, mixture of two isomers). **^{1}H NMR** (500 MHz, $CDCl_3$): δ (ppm) 8.73 (d, J = 9.1 Hz, 2H), 7.88 (d, J = 8.4 Hz, 5H), 7.80 (d, J = 8.4 Hz, 1H), 7.71 (d, J = 8.8 Hz, 1H), 7.54 (d, J = 8.2 Hz, 2H), 7.47 – 7.39 (m, 5H), 7.37 – 7.31 (m, 5H), 7.23 – 7.13 (m, 7H), 7.11 – 7.03 (m, 3H), 7.01 – 6.94 (m, 2H), 6.88 – 6.85 (m, 2H), 6.74 (d, J = 7.8 Hz, 6H), 6.61 (d, J = 7.6 Hz, 1H), 4.45 (s, 7H), 3.68 (s, 3H), 3.63 (s, 4H).**^{13}C {^{1}H} NMR** (126 MHz, $CDCl_3$): δ (ppm) 159.5, 159.3, 144.6, 143.3, 142.8, 142.1, 140.8, 136.8, 136.7, 136.6, 132.84, 132.76, 131.8, 131.73, 131.69, 131.20, 131.0, 130.5, 130.3, 129.2, 129.0, 128.7, 128.6, 128.3, 128.18, 128.12, 128.1, 128.0, 127.9, 126.8, 126.3, 126.2, 125.63, 125.57, 125.4, 125.3, 124.8, 124.7, 124.6, 123.2, 123.1, 123.0, 122.5, 122.3, 121.3, 121.2, 120.22, 120.18, 119.69, 119.66, 119.6, 117.0, 115.6, 113.0, 112.5, 109.1, 55.4, 35.4. **FTIR**: (neat)/ cm^{-1} = 3052, 2935, 2832, 1597, 1575, 1464, 1425, 1385, 1322, 1244, 1167, 1025. **HRMS (ESI) *m/z***: $[M+H]^+$ calcd for $C_{34}H_{26}NO$, 464.2009; found 464.2021.

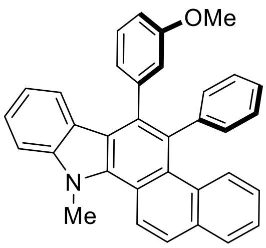

8-(4-Methoxyphenyl)-13-methyl-7-phenyl-13H-naphtho[2,1-a]carbazole (NC3):[6b] The titled compound **NC3** was synthesized according to the **GP-I**. The product **NC3** was isolated after column chromatography using 2% ethyl acetate in hexane as eluent (light yellow solid, 1.97 g, 85% yield, mixture of two isomers). **^{1}H NMR** (400 MHz, $CDCl_3$): δ (ppm) 8.72 (d, J = 9.1 Hz, 2H), 7.87 – 7.85 (m, 4H), 7.75 (d, J = 8.8 Hz, 1H), 7.67 (d, J = 8.8 Hz, 1H), 7.53 (d, J = 8.2 Hz, 2H), 7.45 – 7.38 (m, 4H), 7.35 – 7.31 (m, 3H), 7.23 – 7.17 (m, 5H), 7.14 – 7.07 (m, 5H), 7.06 – 7.00 (m, 3H), 7.00 – 6.96 (m, 1H), 6.93 (d, J = 7.2 Hz, 1H), 6.87 – 6.84 (m, 2H), 6.75 – 6.73 (m 2H), 6.70 (d, J = 8.0 Hz, 1H), 6.57 (d, J = 8.0 Hz, 1H), 4.44 (s, 6H), 3.85 (s, 3H), 3.79 (s, 3H).

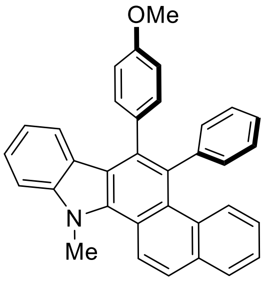

7-(4-Chlorophenyl)-13-methyl-8-phenyl-13H-naphtho[2,1-a]carbazole (NC4): The titled compound **NC4** was synthesized according to the **GP-I**. The product **NC4** was isolated after column chromatography using 2% ethyl acetate in hexane as eluent (light yellow solid, 2.1 g, 90 % yield, mixture of two isomer). **^{1}H NMR** (500 MHz, $CDCl_3$): δ 8.72 (d, J = 9.1 Hz, 3H), 7.90 – 7.87 (m, 5H), 7.69 – 7.66 (m, 3H), 7.56 – 7.53 (m, 3H), 7.47 – 7.39 (m, 6H), 7.35 – 7.32 (m, 5H), 7.30 – 7.28 (m, 2H), 7.22 – 7.13 (m, 12H), 7.12 – 7.03 (m, 8H), 7.02 – 6.98 (m, 1H), 6.95 (t, J = 7.5 Hz, 2H), 6.67 (d, J = 8.0 Hz, 1H), 6.57 (d, J = 8.0 Hz, 2H), 4.451 (s, 3H), 4.447 (s, 5H). **^{13}C {^{1}H} NMR** (126 MHz, $CDCl_3$): δ (ppm) 143.1, 142.9, 142.8, 142.0, 140.6, 139.4, 137.0, 136.9, 135.4, 133.2, 132.9, 132.7, 132.1, 132.0, 131.8, 131.7, 131.51, 131.48, 130.4, 129.8, 129.1, 128.7, 128.6, 128.4, 128.24, 128.20, 128.18, 128.1, 127.1, 126.43, 126.36, 126.3, 125.68, 125.66, 125.5, 125.4, 124.8, 123.1, 123.0, 122.3, 122.15, 121.22, 121.19, 120.4, 120.3, 119.81, 119.76, 119.7, 119.5, 109.2, 109.1, 35.3, 35.2. **FTIR**: (neat)/ cm^{-1} = 3046, 2936, 2832, 2248, 1597, 1466, 1385, 1323, 1257, 1125,1085. **HRMS (ESI) *m/z***: $[M+H]^+$ calcd for $C_{33}H_{23}ClN$, 468.1514; found 468.1523.

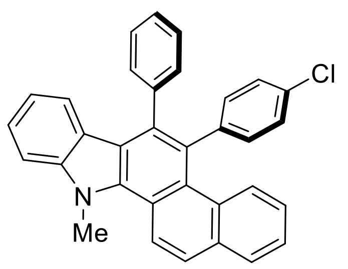

N-(2-Bromophenyl)-N,3-dimethylbenzamide (4d): The titled compound **4d** was synthesised according to **GP-II**. The product **4d** was isolated after column chromatography using 15% ethyl acetate in hexane as eluent (white solid, 433 mg, 95% yield). **^{1}H** NMR (400 MHz, $CDCl_3$): δ (ppm) 7.52 (d, J = 7.8 Hz, 1H), 7.22 (s, 1H), 7.16 – 7.12 (m, 1H), 7.07 – 6.98 (m, 5H), 3.37 (s, 3H), 2.20 (s, 3H). **^{13}C {^{1}H} NMR** (101 MHz, $CDCl_3$): δ (ppm) 170.8, 143.3, 137.1, 135.3, 133.3, 130.4, 130.3, 128.8, 128.6, 128.2, 127.1, 124.7, 122.3, 36.8, 21.0. **FTIR**: (neat)/ cm^{-1} =3750, 3671, 2966, 2922, 2865, 1645, 1581, 1474, 1361, 1304, 1158, 1107, 1033. **HRMS (ESI) *m/z***: $[M+H]^+$ calcd for $C_{15}H_{15}BrNO$, 304.0332; found 304.0322.

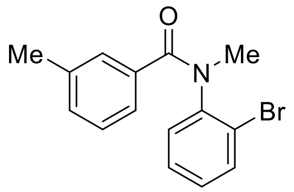

N-(2-Bromophenyl)-N,4-dimethylbenzamide (4e): The titled compound **4e** was synthesised according to **GP-II**. The desired product **4e** was purified by silica gel column chromatography by using 15% ethyl acetate in hexane as eluent (yellow solid, 424 mg, 93% yield, rotameric mixture as 28:1). The characterisation data of the major rotamer is given. **^{1}H NMR** (500 MHz, $CDCl_3$): δ (ppm) 7.51 (d, J = 7.7 Hz, 1H), 7.23 (d, J = 7.5 Hz, 2H), 7.14 (d, J = 7.4 Hz, 1H), 7.06 (dd, J = 12.2, 8.2 Hz, 2H), 6.92 (d, J = 7.1 Hz, 2H), 3.35 (s, 3H), 2.20 (s, 3H). **^{13}C {^{1}H} NMR** (126 MHz, $CDCl_3$): δ (ppm) 171.1, 144.0, 140.0, 133.8, 132.8, 130.7, 129.0, 128.5,

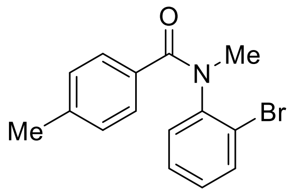

128.4, 128.3, 122.8, 37.3, 21.4. **FTIR**: (neat)/ cm^{-1} = 3058, 2923, 1645, 1477, 1417, 1355, 1306, 1254, 1178, 1110, 1048. **HRMS (ESI) *m/z***: $[M+H]^+$ calcd for $C_{15}H_{15}BrNO$, 304.0332; found 304.0321.

N-(2-Bromophenyl)-4-methoxy-N-methylbenzamide (4f): The titled compound **4f** was synthesised according to **GP-II**. The product **4f** was isolated after column chromatography using 50% ethyl acetate in hexane as eluent (gummy liquid, 418 mg, 87% yield, rotameric mixture as 15:1). The characterisation data of the major rotamer is given. **^{1}H NMR** (400 MHz, $CDCl_3$): δ (ppm) 7.55 (d, *J* = 7.6 Hz, 1H), 7.30 (d, *J* = 7.5 Hz, 2H), 7.18 (t, *J* = 6.9 Hz, 1H), 7.08 (d, *J* = 7.5 Hz, 2H), 6.65 (d, *J* = 7.6 Hz, 2H), 3.71 (s, 3H), 3.36 (s, 3H). **^{13}C {^{1}H} NMR** (126 MHz, $CDCl_3$): δ (ppm) 170.8, 160.9, 133.9, 132.3, 130.7, 130.4, 129.0, 128.6, 128.0, 122.9, 113.1, 55.3, 37.5. **FTIR**: (neat)/ cm^{-1} = 3060, 2933, 2838, 1647, 1608, 1513, 1478, 1419, 1361, 1305, 1253, 1171, 1029. **HRMS (ESI) *m/z***: $[M+H]^+$ calcd for $C_{15}H_{15}BrNO_2$, 320.0281; found 320.0291.

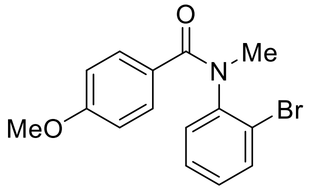

N-(2-Bromophenyl)-3,4-dimethoxy-N-methylbenzamide (4g): The titled compound **4g** was synthesised according to **GP-II**. The product **4g** was isolated after column chromatography using 60% ethyl acetate in hexane as eluent (white solid, 446 mg, 85% yield, rotameric mixture as16:1). **^{1}H NMR** (400 MHz, $CDCl_3$, major rotamer): δ (ppm) 7.55 (d, *J* = 7.6 Hz, 1H), 7.17 – 7.14 (m, 1H), 7.05 (t, *J* = 6.9 Hz, 2H), 6.96 (d, *J* = 7.3 Hz, 1H), 6.88 (s, 1H), 6.62 (d, *J* = 7.6 Hz, 1H), 3.77 (s, 3H), 3.66 (s, 3H), 3.34 (s, 3H). **^{13}C {^{1}H} NMR** (101 MHz, $CDCl_3$, rotameric mixture): δ (ppm) 170.6, 150.3, 147.8, 144.4, 133.8, 130.6, 129.3, 129.0, 128.7, 127.8, 126.8, 126.4, 122.8, 122.7, 122.1, 111.6, 110.0, 55.8, 55.7, 37.5. **FTIR**: (neat)/ cm^{-1} = 3058, 2920, 2280, 2168, 2052, 1984, 1635, 1609, 1420, 1255, 1139, 1019. **HRMS (ESI) *m/z***: $[M+H]^+$ calcd for $C_{16}H_{17}BrNO_3$, 350.0386; found 350.0392.

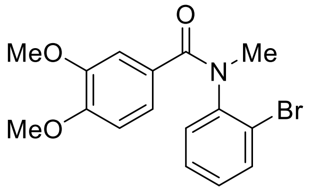

N-(2-Bromophenyl)-4-fluoro-N-methylbenzamide (4h): The titled compound **4h** was synthesised according to **GP-II**. The product **4h** was isolated after column chromatography using 15% ethyl acetate in hexane as eluent (white solid, 439 mg, 95% yield). **^{1}H NMR** (400 MHz, $CDCl_3$): δ (ppm) 7.53 (d, *J* = 7.9 Hz, 1H), 7.35 – 7.32 (m, 2H), 7.18 (t, *J* = 7.5 Hz, 1H), 7.12 – 7.05 (m, 2H), 6.82 (t, *J* = 8.5 Hz, 2H), 3.36 (s, 3H). **^{13}C {^{1}H} NMR** (101 MHz, $CDCl_3$, rotameric mixture): δ (ppm) 170.1, 168.5, 166.0 (d, *J* = 254.0 Hz) 163.4 (d, *J* = 250.4 Hz),

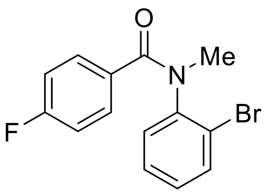

143.6, 133.9, 132.7 (d, *J* = 9.4 Hz), 131.8 (d, *J* = 3.3 Hz), 130.5 (d, *J* = 9.2 Hz), 129.3, 128.7, 122.7, 115.6 (d, *J* = 22.0 Hz), 114.9 (d, *J* = 21.8 Hz), 37.4. **^{19}F NMR** (470 MHz, $CDCl_3$, rotameric mixture): δ (ppm) -105.5, -109.7. **FTIR**: (neat)/ cm^{-1} =3068, 2927, 1652, 1611, 1513, 1479, 1419, 1365, 1307, 1228, 1157, 1112, 1048. **HRMS (ESI)** ***m/z***: $[M+H]^+$ calcd for $C_{14}H_{12}BrFNO$, 308.0081; found 308.0081.

N-(2-Bromophenyl)-N-methyl-3-(trifluoromethyl)benzamide (4i): The titled compound **4i** was synthesised according to **GP-II**. The product **4i** was isolated after column chromatography using 20% ethyl acetate in hexane as eluent (yellow liquid, 489 mg, 91% yield, rotameric mixture as 3:1). **^{1}H NMR** (500 MHz, $CDCl_3$, major rotamer): δ (ppm) 7.62 (s, 1H), 7.54 (t, *J* = 7.6 Hz, 2H), 7.47 (d, *J* = 7.7 Hz, 1H), 7.28 (t, *J* = 7.9 Hz, 1H), 7.20 (t, *J* = 7.5 Hz, 1H), 7.13 (d, *J* = 7.5 Hz, 1H), 7.08 (t, *J* = 7.5 Hz, 1H), 3.41 (s, 3H). **^{13}C {^{1}H} NMR** (126 MHz, $CDCl_3$, rotameric mixture): δ (ppm) 169.6, 168.8, 143.2, 136.5, 134.0, 133.4, 131.5, 131.2, 130.8, 130.6, 130.4, 130.1, 130.0 (q, *J* = 3.6 Hz), 129.7, 129.3, 128.8, 128.4, 127.2 (q, *J* = 3.8 Hz), 126.61, 126.58, 125.40, 15.38, 123.8 (q, *J* = 272.3 Hz), 123.7 (q, *J* = 272.4 Hz), 122.9, 37.3. **^{19}F NMR** (471 MHz, $CDCl_3$, rotameric mixture as 3:1): δ (ppm) -102.8, -103.0. **FTIR**: (neat)/ cm^{-1} = 3069, 2933, 1725, 1654, 1584, 1479, 1371, 1327, 1304, 1167, 1126, 1073. **HRMS (ESI)** ***m/z***: $[M+H]^+$ calcd for $C_{15}H_{12}F_3NO$, 358.0049; found 358.0056.

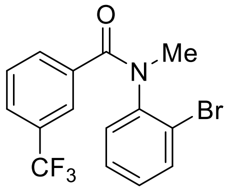

N-(2-Bromo-4-methylphenyl)-N-methylbenzamide (4j): The titled compound **4j** was synthesised according to **GP-II**. The product **4j** was isolated after column chromatography using 15% ethyl acetate in hexane as eluent (brown liquid, 419 mg, 92% yield, rotameric mixture as 1:1). **^{1}H NMR** (400 MHz, $CDCl_3$): δ (ppm) 7.35 (d, *J* = 7.6 Hz, 3H), 7.30 (d, *J* = 7.1 Hz, 2H), 7.24 – 7.18 (m, 2H), 7.18 – 7.14 (m, 4H), 7.01 (d, *J* = 8.1 Hz, 2H), 6.97 – 6.90 (m, 4H), 3.47 (s, 3H), 3.36 (s, 3H), 2.26 (s, 3H), 2.24 (s, 3H). **^{13}C {^{1}H} NMR** (101 MHz, $CDCl_3$, rotameric mixture): δ (ppm) 171.3, 170.9, 167.9, 142.2, 140.5, 139.5, 136.4, 135.8, 135.7, 134.4, 134.0, 133.0, 132.1, 130.1, 130.0, 129.8, 129.64, 129.55, 129.2, 128.7, 128.10, 128.05, 127.70, 127.66, 126.7, 122.2, 38.6, 37.3, 21.0, 20.7. **FTIR**: (neat)/ cm^{-1} = 3029, 2924, 1721, 1647, 1576, 1512, 1494, 1362, 1302, 1111, 1048. **HRMS (ESI)** ***m/z***: $[M+H]^+$ calcd for $C_{15}H_{15}BrNO$, 304.0332; found 304.0323.

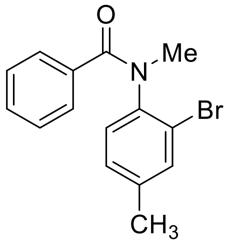

N-(2-Bromo-4-methoxyphenyl)-N-methylbenzamide (4k): The titled compound **4k** was synthesised according to **GP-II**. The product **4k** was isolated after column chromatography using 50% ethyl acetate in hexane as eluent (gummy liquid, 402 mg, 84% yield).

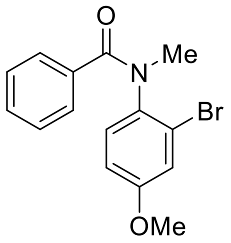

^{1}H NMR (500 MHz, $CDCl_3$): δ (ppm) 7.33 (d, *J* = 7.2 Hz, 2H), 7.19 (t, *J* = 7.3 Hz, 1H), 7.13 (t, *J* = 7.4 Hz, 2H), 7.01 (d, *J* = 2.7 Hz, 1H), 6.97 (d, *J* = 8.7 Hz, 1H), 6.65 (dd, *J* = 8.7, 2.7 Hz, 1H), 3.68 (s, 3H), 3.33 (s, 3H). **^{13}C {^{1}H} NMR** (126 MHz, $CDCl_3$): δ (ppm) 171.2, 159.1, 136.5, 136.0, 130.9, 129.6, 128.0, 127.7, 123.2, 118.4, 114.3, 55.7, 37.3. **FTIR**: (neat)/ cm^{-1} = 2938, 1645, 1600, 1494, 1364, 1312, 1286, 1228, 1113, 1031. **HRMS (ESI) *m/z***: $[M+H]^+$ calcd for $C_{15}H_{15}BrNO_2$, 320.0281; found 320.0274.

N-(2-Bromo-4-(trifluoromethyl)phenyl)-N-methylbenzamide (4l): The titled compound **4l** was synthesised according to **GP-II**. The product **4l** was isolated after column chromatography using 20% ethyl acetate in hexane as eluent (yellow gummy liquid, 495 mg, 92% yield).

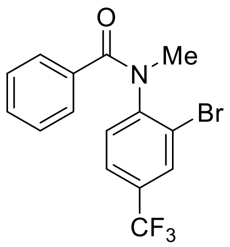

^{1}H NMR (400 MHz, $CDCl_3$): δ (ppm) 7.79 (s, 1H), 7.40 – 7.31 (m, 3H), 7.22 – 7.16 (m, 4H), 3.36 (s, 3H). **^{13}C {^{1}H} NMR** (101 MHz, $CDCl_3$): δ (ppm) 170.8, 147.1, 135.1, 131.1, 131.0 (q, *J* = 3.7 Hz), 130.3, 128.1, 128.0, 125.5, 123.1, 122.7 (q, *J* = 272.3 Hz). 37.02. **^{19}F NMR** (471 MHz, $CDCl_3$): δ (ppm) -62.7. **FTIR**: (neat)/ cm^{-1} = 3066, 2940, 1655, 1605, 1498, 1356, 1320, 1255, 1172, 1078. **HRMS (ESI) *m/z***: $[M+H]^+$ calcd for $C_{15}H_{12}F_3NO$, 358.0049; found 358.0043.

***N-(2-Bromophenyl)-N-ethylbenzamide (4n)**:* The titled compound **4n** was synthesised according to **GP-II** by using bromoethane (0.2 mL, 3.0 mmol, 2.0 equiv). The product **4n** was isolated after column chromatography using 15% ethyl acetate in hexane as eluent (white solid, 410 mg, 90% yield).

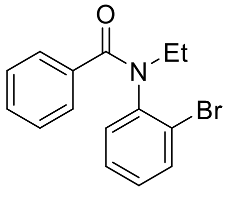

^{1}H NMR (500 MHz, $CDCl_3$): δ (ppm) 7.52 (d, *J* = 7.8 Hz, 1H), 7.33 (d, *J* = 7.4 Hz, 2H), 7.21 – 7.11 (m, 4H), 7.05 (t, *J* = 6.9 Hz, 2H), 4.27 (dq, *J* = 14.1, 7.1 Hz, 1H), 3.57 (dq, *J* = 14.1, 7.1 Hz, 1H), 1.22 (t, *J* = 7.1 Hz, 3H). **^{13}C {^{1}H} NMR** (126 MHz, $CDCl_3$): δ (ppm) 170.5, 141.9, 136.3, 133.9, 132.1, 129.7, 129.1, 128.1, 127.7, 123.5, 44.2, 12.6. **FTIR**: (neat)/ cm^{-1} = 3057, 2981, 2931, 2870, 1638, 1577, 1472, 1435, 1391, 1304, 1115, 1029. **HRMS (ESI) *m/z***: $[M+H]^+$ calcd for $C_{15}H_{15}BrNO$, 304.0332; found 304.0330.

N*-(2-Bromophenyl)-*N*-hexylbenzamide (4o):** The titled compound **4o** was synthesised according to **GP-II** by using 1-bromohexane (0.3 mL, 2.25 mmol, 1.5 equiv). The product **4o** was isolated after column chromatography using 5% ethyl acetate in hexane as eluent (liquid, 281 mg, 50% yield). **^{1}H NMR** (400 MHz, $CDCl_3$): δ (ppm) 7.51 (d, *J* = 7.7 Hz, 1H), 7.31 (d, *J* = 7.5 Hz, 2H), 7.21 – 7.10 (m, 4H), 7.06 – 7.02 (m, 2H), 4.26 – 4.19 (m, 1H), 3.47 – 3.40 (m, 1H), 1.71 – 1.65 (m, 1H), 1.58 – 1.49 (m, 1H), 1.40 – 1.25 (m, 6H), 0.87 – 0.85 (m, 3H). **^{13}C {^{1}H} NMR** (126 MHz, $CDCl_3$): δ (ppm) 170.6, 142.2, 136.3, 133.9, 132.0, 129.6, 129.0, 128.1, 128.0, 127.7, 123.5, 49.4, 31.7, 27.4, 26.8, 22.7, 14.1. **FTIR**: (neat)/ cm^{-1} = 3060, 2955, 2928, 2857, 1651, 1580, 1446, 1388, 1310, 1130, 1029. **HRMS (ESI) *m/z: $[M+H]^+$ calcd for $C_{19}H_{23}BrNO$, 360.0958; found 360.0956.

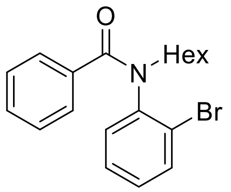

N*-(2-Bromo-4-methylphenyl)-*N*-tosylbenzamide (10q):**[10b,12] The titled compound **10q** was synthesised according to modified literature procedure by using the corresponding **S6** (578 mg, 2 mmol, 1.0 equiv), triethylamine (3 mmol, 1.5 equiv) and tosyl chloride (3 mmol, 1.5 equiv). The desired product **10q** was isolated after silica gel column chromatography by using 5% ethyl acetate in hexane as eluent (white solid, 799 mg, 90% yield). **^{1}H NMR** (400 MHz, $CDCl_3$): δ 8.00 (d, *J* = 8.2 Hz, 2H), 7.43 (d, *J* = 7.6 Hz, 2H), 7.40 (d, *J* = 8.2 Hz, 1H), 7.34 (d, *J* = 8.1 Hz, 1H), 7.30 – 7.26 (m, 1H), 7.18 – 7.11 (m, 3H), 2.45 (s, 3H), 2.28 (s, 3H). **^{13}C {^{1}H} NMR** (126 MHz, $CDCl_3$): δ (ppm) 169.3, 145.2, 141.7, 136.0, 134.4, 134.2, 133.9, 133.3, 131.5, 130.2, 129.3, 129.04, 128.96, 127.8, 124.6, 21.9, 21.0. **FTIR**: (neat)/ cm^{-1} = 1692, 1598, 1448, 1364, 1278, 1259, 1242, 1085, 1027. **HRMS (ESI) *m/z: $[M+H]^+$ calcd for $C_{21}H_{19}BrNO_3S$, 444.0264; found 444.0272.

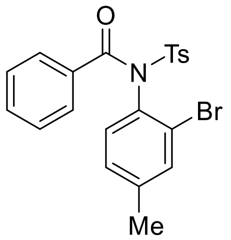

4,4,5,5-Tetramethyl-2-(p-tolyl)-1,3,2-dioxaborolane (2a):[13a] The titled compound **2a** was synthesised according to the **GP-III** by using 1-bromo-4-methylbenzene **1a** (62 μL, 0.5 mmol, 1.0 equiv) as prototype. The borylated product **2a** was isolated after column chromatography using 2% ethyl acetate in hexane as eluent (white solid, 79 mg, 72% yield). **^{1}H NMR** (400 MHz, $CDCl_3$): δ (ppm) 7.73 (d, *J* = 7.8 Hz, 2H), 7.21 (d, *J* = 7.9 Hz, 2H), 2.38 (s, 3H), 1.36 (s, 12H). **^{13}C {^{1}H} NMR** (101 MHz, $CDCl_3$): δ (ppm) 141.5, 134.9, 128.6, 83.7, 25.0, 21.9. (The carbon directly attached to the boron atom was not detected due to quadrupolar broadening). **^{11}B NMR** (160 MHz, $CDCl_3$): δ (ppm) 31.0.

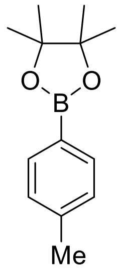

2-(4-(tert-Butyl)phenyl)-4,4,5,5-tetramethyl-1,3,2-dioxaborolane (2b):[13a] The titled compound **2b** was synthesised according to the **GP-III** by using 1-bromo-4-(*tert*-butyl)benzene **1b** (87 µL, 0.5 mmol, 1.0 equiv) as prototype. The product **2b** was isolated after column chromatography using 2% ethyl acetate in hexane as eluent (white solid, 110 mg, 85% yield). **^{1}H NMR** (400 MHz, $CDCl_3$): δ (ppm) 7.79 (d, *J* = 7.7 Hz, 2H), 7.43 (d, *J* = 7.6 Hz, 2H), 1.36 (s, 12H), 1.35 (s, 9H). **^{13}C {^{1}H} NMR** (101 MHz, $CDCl_3$): δ (ppm) 154.6, 134.8, 124.8, 83.7, 35.0, 31.3, 25.0. (The carbon directly attached to the boron atom was not detected due to quadrupolar broadening). **^{11}B NMR** (160 MHz, $CDCl_3$): δ (ppm) 31.0.

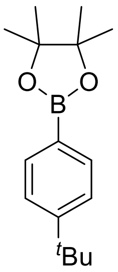

2-(4-Methoxyphenyl)-4,4,5,5-tetramethyl-1,3,2-dioxaborolane (2c):[13a] The titled compound **2c** was synthesised according to the **GP-III** by using 1-bromo-4-methoxylbenzene **1c** (63 µL, 0.5 mmol, 1.0 equiv). The borylated product **2c** was isolated after column chromatography using 2% ethyl acetate in hexane as eluent (white solid, 94.8 mg, 81% yield). **^{1}H NMR** (400 MHz, $CDCl_3$): δ (ppm) 7.78 (d, *J* = 8.4 Hz, 2H), 6.91 (d, *J* = 8.5 Hz, 2H), 3.83 (s, 3H), 1.34 (s, 12H). **^{13}C {^{1}H} NMR** (126 MHz, $CDCl_3$): δ (ppm) 162.3, 136.7, 113.5, 83.7, 55.2, 25.0. (The carbon directly attached to the boron atom was not detected due to quadrupolar broadening). **^{11}B NMR** (160 MHz, $CDCl_3$): δ (ppm) 30.79.

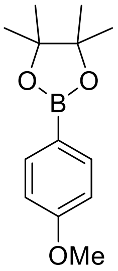

2-([1,1'-Biphenyl]-4-yl)-4,4,5,5-tetramethyl-1,3,2-dioxaborolane (2d):[13a] The titled compound **2d** was synthesised according to the **GP-III** by using 4-bromo-1,1'-biphenyl **1d** (117 mg, 0.5 mmol, 1.0 equiv). The product **2d** was isolated after column chromatography using 2% ethyl acetate in hexane as eluent (white solid, 126 mg, 90% yield). **^{1}H NMR** (400 MHz, $CDCl_3$): δ (ppm) 7.94 (d, *J* = 7.8 Hz, 2H), 7.66 (d, *J* = 7.2 Hz, 4H), 7.48 (t, *J* = 7.3 Hz, 2H), 7.39 (t, *J* = 7.0 Hz, 1H), 1.40 (s, 12H). **^{13}C {^{1}H} NMR** (126 MHz, $CDCl_3$): δ (ppm) 144.0, 141.2, 135.4, 128.9, 127.7, 127.4, 126.6, 83.9, 25.0. (The carbon directly attached to the boron atom was not detected due to quadrupolar broadening). **^{11}B NMR** (160 MHz, $CDCl_3$): δ (ppm) 31.2.

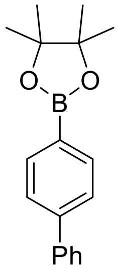

Methyl 4-(4,4,5,5-tetramethyl-1,3,2-dioxaborolan-2-yl)benzoate (2e):[13a] The titled compound **2e** was synthesised according to the **GP-III** by using methyl 4-bromobenzoate **1e** (108 mg, 0.5 mmol, 1.0 equiv). The product **2e** was isolated after column chromatography using 4% ethyl acetate in hexane as eluent (white solid, 106 mg, 81% yield). **^{1}H NMR** (500 MHz, $CDCl_3$): δ (ppm) 8.02 (d, *J* = 7.9 Hz, 2H), 7.87 (d, *J* = 7.9 Hz, 2H), 3.92 (s, 3H), 1.35 (s, 12H). **^{13}C {^{1}H} NMR** (126 MHz, $CDCl_3$):

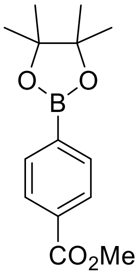

δ (ppm) 167.3, 134.8, 132.5, 128.7, 84.3, 52.3, 25.0. (The carbon directly attached to the boron atom was not detected due to quadrupolar broadening). **^{11}B NMR** (160 MHz, $CDCl_3$): δ (ppm) 30.8.

4-(4,4,5,5-Tetramethyl-1,3,2-dioxaborolan-2-yl)benzonitrile (2f):[13a] The titled compound **2f** was synthesised according to the **GP-III** by using 4-bromobenzonitrile **1h** (91 mg, 0.5 mmol, 1.0 equiv). The product **2f** was isolated after column chromatography using 3% ethyl acetate in hexane as eluent (white solid, 76.7 mg, 67% yield). **^{1}H NMR** (500 MHz, $CDCl_3$): δ (ppm) 7.87 (d, J = 8.1 Hz, 2H), 7.63 (d, J = 8.2 Hz, 2H), 1.34 (s, 12H). **^{13}C {^{1}H} NMR** (126 MHz, $CDCl_3$): δ (ppm) 135.2, 131.2, 119.1, 114.9, 84.6, 25.0. (The carbon directly attached to the boron atom was not detected due to quadrupolar broadening). **^{11}B NMR** (160 MHz, $CDCl_3$): δ (ppm) 30.6.

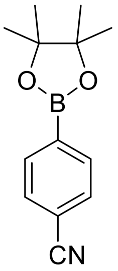

Methyl 2-(2-(4,4,5,5-tetramethyl-1,3,2-dioxaborolan-2-yl)phenyl)acetate (2g):[13b] The titled compound **2g** was synthesised according to the **GP-III** by using methyl 2-(2-bromophenyl)acetate **1i** (115 mg, 0.5 mmol, 1.0 equiv). The product **2g** was isolated after column chromatography using 5% ethyl acetate in hexane as eluent (colourless liquid, 81.4 mg, 59% yield). **^{1}H NMR** (400 MHz, $CDCl_3$): δ (ppm) 7.84 (d, J = 7.2 Hz, 1H), 7.39 (dt, J = 7.5, 3.8 Hz, 1H), 7.29 – 7.26 (m, 1H), 7.20 (d, J = 7.4 Hz, 1H), 3.99 (s, 2H), 3.67 (s, 3H), 1.33 (s, 12H). **^{13}C {^{1}H} NMR** (126 MHz, $CDCl_3$): δ (ppm) 173.0, 140.5, 136.2, 131.1, 130.2, 126.5, 83.8, 51.8, 41.1, 24.8. (The carbon directly attached to the boron atom was not detected due to quadrupolar broadening). **^{11}B NMR** (160 MHz, $CDCl_3$): δ (ppm) 31.1.

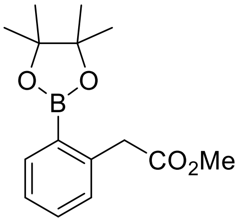

4,4,5,5-Tetramethyl-2-(naphthalen-1-yl)-1,3,2-dioxaborolane (2h):[13a] The titled compound **2h** was synthesised according to the **GP-III** by using 1-bromonaphthalene **1j** (104 mg, 0.5 mmol, 1.0 equiv). The product **2h** was isolated after column chromatography using 2% ethyl acetate in hexane as eluent (white solid, 58.4 mg, 46% yield). **^{1}H NMR** (400 MHz, $CDCl_3$): δ (ppm) 8.81 (d, J = 8.3 Hz, 1H), 8.13 (d, J = 6.4 Hz, 1H), 7.97 (d, J = 8.1 Hz, 1H), 7.87 (d, J = 8.0 Hz, 1H), 7.57 (t, J = 7.2 Hz, 1H), 7.51 (t, J = 7.4 Hz, 2H), 1.46 (s, 12H). **^{13}C {^{1}H} NMR** (101 MHz, $CDCl_3$): δ (ppm) 137.1, 135.8, 133.3, 131.7, 128.6, 128.5, 126.5, 125.6, 125.1, 83.8, 25.1. (The carbon directly attached to the boron atom was not detected due to quadrupolar broadening). **^{11}B NMR** (160 MHz, $CDCl_3$): δ (ppm) 31.8.

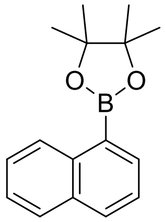

4,4,5,5-Tetramethyl-2-(pyren-1-yl)-1,3,2-dioxaborolane (2i):[13c] The titled compound **2i** was

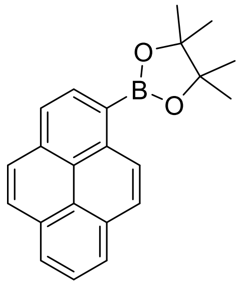

synthesised according to the **GP-III** by using 1-bromopyrene **1k** (104 mg, 0.5 mmol, 1.0 equiv). The product **2i** was isolated after column chromatography using 5% ethyl acetate in hexane as eluent (white solid, 131 mg, 80% yield). **^{1}H NMR** (500 MHz, $CDCl_3$): δ (ppm) 9.14 – 9.12 (m, 1H), 8.59 (ddd, *J* = 7.5, 3.1, 1.9 Hz, 1H), 8.24 (d, *J* = 7.6 Hz, 1H), 8.21 – 8.17 (m, 3H), 8.13 (d, *J* = 8.9 Hz, 1H), 8.08 (d, *J* = 8.9 Hz, 1H), 8.02 (t, *J* = 7.6 Hz, 1H), 1.53 (s, 12H). **^{13}C {^{1}H} NMR** (126 MHz, $CDCl_3$): δ (ppm) 136.6, 134.0, 133.6, 131.3, 130.9, 128.7, 128.2, 127.9, 127.6, 125.8, 125.5, 125.3, 124.8, 124.5, 124.2, 84.0, 25.2. (The carbon directly attached to the boron atom was not detected due to quadrupolar broadening). **^{11}B NMR** (160 MHz, $CDCl_3$): δ (ppm) 31.9.

5-(4,4,5,5-Tetramethyl-1,3,2-dioxaborolan-2-yl)-1H-indole (2j):[13d] The titled compound **2j**

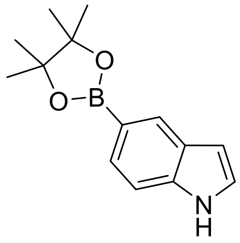

was synthesised according to the **GP-III** by using 5-bromo-1*H*-indole **1l** (98 mg, 0.5 mmol, 1.0 equiv). The product **2j** was isolated after column chromatography using 3% ethyl acetate in hexane as eluent (white solid, 36.5 mg, 30% yield). **^{1}H NMR** (400 MHz, $CDCl_3$): δ (ppm) 8.24 (br s, 1H), 8.20 (s, 1H), 7.65 (d, *J* = 8.2 Hz, 1H), 7.38 (d, *J* = 8.2 Hz, 1H), 7.18 (t, *J* = 2.7 Hz, 1H), 6.57 (s, 1H), 1.38 (s, 12H). **^{13}C {^{1}H} NMR** (101 MHz, $CDCl_3$): δ (ppm) 138.0, 128.8, 128.2, 127.7, 124.3, 110.6, 103.3, 83.6, 25.0. (The carbon directly attached to the boron atom was not detected due to quadrupolar broadening). **^{11}B NMR** (160 MHz, $CDCl_3$): δ (ppm) 31.6.

Methyl 5-(4,4,5,5-tetramethyl-1,3,2-dioxaborolan-2-yl)-1H-indole-1-carboxylate (2k): The

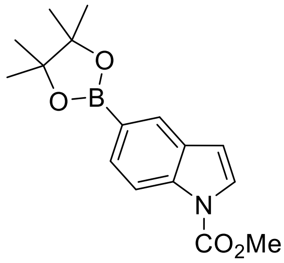

titled compound **2k** was synthesised according to the **GP-III** by using methyl 5-bromo-1*H*-indole-1-carboxylate **1m** (127 mg, 0.5 mmol, 1.0 equiv). The product **2k** was isolated after column chromatography using 5% ethyl acetate in hexane as eluent (white solid, 93.4 mg, 62% yield). **^{1}H NMR** (400 MHz, $CDCl_3$): δ (ppm) 8.17 (d, *J* = 7.8 Hz, 1H), 8.07 (s, 1H), 7.78 (d, *J* = 8.3 Hz, 1H), 7.59 (d, *J* = 3.3 Hz, 1H), 6.60 (d, *J* = 3.6 Hz, 1H), 4.04 (s, 3H), 1.37 (s, 12H). **^{13}C {^{1}H} NMR** (126 MHz, $CDCl_3$): δ (ppm) 151.6, 137.4, 131.0, 130.2, 128.4, 125.7, 114.6, 108.5, 83.9, 53.9, 25.0. (The carbon directly attached to the boron atom was not detected due to quadrupolar broadening). **^{11}B NMR** (160 MHz, $CDCl_3$): δ (ppm) 31.4. **FTIR**: (neat)/ cm^{-1} = 3672, 2978, 1745, 1612, 1540, 1444, 1243, 1214, 1145, 1087. **HRMS (ESI) *m/z***: $[M+H]^+$ calcd for $C_{16}H_{21}BNO_4$, 302.1558; found 302.1555.

2-(4-(4,4,5,5-Tetramethyl-1,3,2-dioxaborolan-2-yl)phenyl)-1H-indole (2l): The titled compound **2l** was synthesised according to the **GP-III** by using methyl 2-(4-bromophenyl)-1*H*-indole **1n** (135 mg, 0.5 mmol, 1.0 equiv). The product **2l** was isolated after column chromatography using 3% ethyl acetate in hexane as eluent (white solid, 104 mg, 65% yield). ^{1}**H NMR** (400 MHz, $CDCl_3$): δ (ppm) 8.42 (s, 1H), 7.88 (d, *J* = 8.0 Hz, 2H), 7.68 (d, *J* = 8.1 Hz, 2H), 7.63 (d, *J* = 7.8 Hz, 1H), 7.40 (d, *J* = 8.1 Hz, 1H), 7.20 (t, *J* = 7.6 Hz, 1H), 7.12 (t, *J* = 7.4 Hz, 1H), 6.91 (s, 1H), 1.37 (s, 12H). ^{13}**C {^{1}H} NMR** (101 MHz, $CDCl_3$): δ (ppm) 137.7, 137.0, 135.6, 134.9, 129.3, 124.3, 122.8, 120.9, 120.5, 111.1, 100.8, 84.1, 25.0. (The carbon directly attached to the boron atom was not detected due to quadrupolar broadening). ^{11}**B NMR** (160 MHz, $CDCl_3$): δ (ppm) 30.6. **FTIR**: (neat)/ cm^{-1} = 3344, 2977, 2924, 2853, 1609, 1453, 1360, 1283, 1121. **HRMS (ESI) *m/z***: $[M+H]^+$ calcd for $C_{20}H_{23}BNO_2$, 320.1816; found 320.1820.

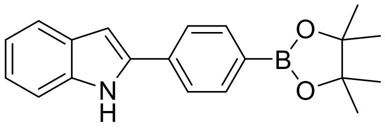

3-(4,4,5,5-Tetramethyl-1,3,2-dioxaborolan-2-yl)-9H-carbazole (2m):[13e] The titled compound **2m** was synthesised according to the **GP-III** by using 3-bromo-9*H*-carbazole **1o** (123 mg, 0.5 mmol, 1.0 equiv). The product **2m** was isolated after column chromatography using 2% ethyl acetate in hexane as eluent (white solid, 88 mg, 60% yield). ^{1}**H NMR** (400 MHz, $CDCl_3$): δ (ppm) 8.61 (s, 1H), 8.16 (br s, 1H), 8.12 (d, *J* = 7.3 Hz, 1H), 7.90 (d, *J* = 8.0 Hz, 1H), 7.44 – 7.39 (m, 3H), 7.25 (d, *J* = 7.5 Hz, 1H), 1.42 (s, 12H). ^{13}**C {^{1}H} NMR** (126 MHz, $CDCl_3$): δ (ppm) 141.9, 139.6, 132.5, 127.9, 126.0, 123.6, 123.2, 120.7, 120.0, 110.7, 110.2, 83.8, 25.1. (The carbon directly attached to the boron atom was not detected due to quadrupolar broadening). ^{11}**B NMR** (160 MHz, $CDCl_3$): δ (ppm) 31.6.

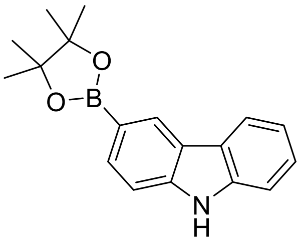

3,6-Bis(4,4,5,5-tetramethyl-1,3,2-dioxaborolan-2-yl)-9H-carbazole (2n):[13f] The titled compound **2n** was synthesised according to the **GP-III** by using 3,6-dibromo-9*H*-carbazole **1p** (163 mg, 0.5 mmol, 1.0 equiv). The product **2n** was isolated after column chromatography using 2% ethyl acetate in hexane as eluent (white solid, 46 mg, 22% yield). In another fractions, the debrominated mono-borylation product **2m** was isolated as major product (79 mg, 54% yield). ^{1}**H NMR** (400 MHz, $CDCl_3$): δ (ppm) 8.65 (s, 2H), 8.25 (br s, 1H), 7.86 (d, *J* = 8.1 Hz, 2H), 7.39 (d, *J* = 8.1 Hz, 2H), 1.39 (s, 24H). ^{13}**C {^{1}H} NMR** (101 MHz, $CDCl_3$): δ (ppm) 141.8,

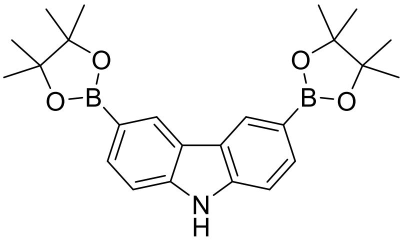

132.5, 128.3, 123.4, 110.2, 83.8, 25.2. (The carbon directly attached to the boron atom was not detected due to quadrupolar broadening). **^{11}B NMR** (160 MHz, $CDCl_3$): δ (ppm) 31.5.

1,3-Bis(4,4,5,5-tetramethyl-1,3,2-dioxaborolan-2-yl)benzene (2o):[13g] The titled compound **2o** was synthesised according to the **GP-III** by using 1,3-dibromobenzene **1q** (60 µL, 0.5 mmol, 1.0 equiv). The product **2o** was isolated after column chromatography using 2% ethyl acetate in hexane as eluent (white solid, 91 mg, 55% yield). **^{1}H** NMR (400 MHz, $CDCl_3$): δ (ppm) 8.28 (s, 1H), 7.90 (d, J = 8.1 Hz, 2H), 7.38 (t, J = 7.4 Hz, 1H), 1.34 (s, 24H). **^{13}C {^{1}H} NMR** (101 MHz, $CDCl_3$): 141.4, 137.8, 127.2, 83.9, 25.0. **^{11}B NMR** (160 MHz, $CDCl_3$): δ (ppm) 31.2.

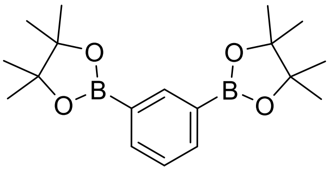

1,4-Bis(4,4,5,5-tetramethyl-1,3,2-dioxaborolan-2-yl)benzene (2p):[13a] The titled compound **2p** was synthesised according to the **GP-III** by using 1,4-dibromobenzene **1r** (65 µL, 0.5 mmol, 1.0 equiv). The product **2p** was isolated after column chromatography using 2% ethyl acetate in hexane as eluent (white solid, 119 mg, 72% yield). **^{1}H NMR** (400 MHz, $CDCl_3$): δ (ppm) 7.80 (s, 4H), 1.35 (s, 24H). **^{13}C {^{1}H} NMR** (101 MHz, $CDCl_3$): δ(ppm) 134.0, 84.0, 25.0. (The carbon directly attached to the boron atom was not detected due to quadrupolar broadening). **^{11}B NMR** (160 MHz, $CDCl_3$): δ (ppm) 31.01.

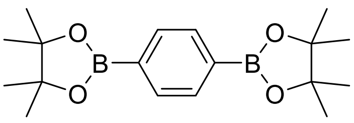

3,3'-Bis(4,4,5,5-tetramethyl-1,3,2-dioxaborolan-2-yl)-1,1'-biphenyl (2q):[13h] The titled compound **2q** was synthesised according to the **GP-III** by using 3,3'-dibromo-1,1'-biphenyl **1s** (156 mg, 0.5 mmol, 1.0 equiv). The product **2q** was isolated after column chromatography using 2% ethyl acetate in hexane as eluent (white solid, 144 mg, 71% yield). **^{1}H NMR** (400 MHz, $CDCl_3$): δ (ppm) 8.07 (s, 2H), 7.80 (d, J = 7.3 Hz, 2H), 7.73 (d, J = 7.8 Hz, 2H), 7.44 (t, J = 7.5 Hz, 2H), 1.37 (s, 24H). **^{13}C {^{1}H} NMR** (101 MHz, $CDCl_3$): δ (ppm) 140.7, 133.8, 133.7, 130.4, 128.2, 84.0, 25.0. (The carbon directly attached to the boron atom was not detected due to quadrupolar broadening). **^{11}B NMR** (160 MHz, $CDCl_3$): δ (ppm) 31.2.

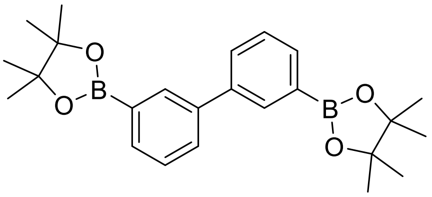

1,1'-(2,7-bis(4,4,5,5-tetramethyl-1,3,2-dioxaborolan-2-yl)-9H-fluorene-9,9-diyl)bis(hexan-1-one) (2r):[13i] The titled compound **2r** was synthesised according to the **GP-III** by using 1,1'-(2,7-dibromo-9H-fluorene-9,9-diyl)bis(hexan-1-one) **1t** (260 mg, 0.5

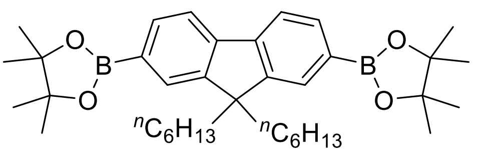

mmol, 1.0 equiv). The product **2r** was isolated after column chromatography using 5% ethyl acetate in hexane as eluent (white solid, 175 mg, 57% yield). **^{1}H NMR** (400 MHz, $CDCl_3$): δ (ppm) 7.80 (d, *J* = 7.5 Hz, 2H), 7.74 (s, 2H), 7.72 (d, *J* = 7.6 Hz, 2H), 2.02 – 1.97 (m, 4H), 1.39 (s, 24H), 1.10 – 1.00 (m, 12H), 0.76 – 0.72 (m, 6H), 0.57 – 0.50 (m, 4H). **^{13}C {^{1}H} NMR** (101 MHz, $CDCl_3$): δ (ppm) 150.6, 144.1, 133.8, 129.1, 119.5, 83.9, 55.3, 40.2, 31.6, 29.8, 25.1, 23.7, 22.7, 14.2. (The carbon directly attached to the boron atom was not detected due to quadrupolar broadening).

Dimethyl [1,1'-biphenyl]-4-ylphosphonate (3a):[14] The titled compound **3a** was synthesized according to **GP-IV** by using 4-bromo-1,1'-biphenyl **1d** (47 mg, 0.2 mmol). The desired product **3a** was isolated after silica gel column chromatography by using 60% ethyl acetate in hexane as eluent (yellow solid, 48.2 mg, 92% yield). **^{1}H NMR** (400 MHz, $CDCl_3$): δ (ppm) 7.89 – 7.84 (m, 2H), 7.69 (dd, *J* = 7.9, 3.8 Hz, 2H), 7.60 (d, *J* = 7.6 Hz, 2H), 7.46 (t, *J* = 7.5 Hz, 2H), 7.39 (t, *J* = 7.0 Hz, 1H), 3.78 (d, *J* = 11.1 Hz, 6H). **^{13}C {^{1}H} NMR** (101 MHz, $CDCl_3$): δ (ppm) 145.6 (d, *J* = 3.2 Hz) 139.9, 132.5 (d, *J* = 10.3 Hz), 129.1, 128.3, 127.37, 127.36 (d, *J* = 15.4 Hz), 125.4 (d, *J* = 190.7 Hz), 52.7 (d, *J* = 5.5 Hz). **^{31}P NMR** (202 MHz, $CDCl_3$): δ (ppm) 22.5.

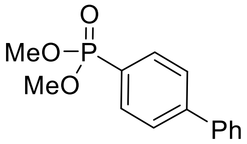

Dimethyl benzo[d][1,3]dioxol-5-ylphosphonate (3b): The titled compound **3b** was synthesized according to **GP-IV** by using 5-bromobenzo[*d*][1,3]dioxole **1u** (24 μL mg, 0.2 mmol). The desired product **3b** was isolated after silica gel column chromatography by using 80% ethyl acetate in hexane as eluent (yellow oil, 22 mg, 49% yield). **^{1}H NMR** (400 MHz, $CDCl_3$) δ (ppm) 7.37 (ddd, *J* = 14.0, 7.9, 0.9 Hz, 1H), 7.18 (d, *J* = 14.1 Hz, 1H), 6.90 (dd, *J* = 7.9, 3.7 Hz, 1H), 6.03 (s, 2H), 3.74 (d, *J* = 11.1 Hz, 6H). **^{13}C {^{1}H} NMR** (101 MHz, $CDCl_3$): δ (ppm) 151.6 (d, *J* = 3.5 Hz), 148.1 (d, *J* = 22.7 Hz), 127.8 (d, *J* = 11.1 Hz), 119.8 (d, *J* = 194.4 Hz), 111.4 (d, *J* = 12.3 Hz), 108.8 (d, *J* = 18.8 Hz), 101.8, 52.8 (d, *J* = 5.4 Hz). **^{31}P NMR** (202 MHz, $CDCl_3$): δ (ppm) 22.5. **FTIR**: (neat)/ cm^{-1} = 3458, 2923, 2853, 1722, 1604, 1485, 1428, 1344, 1246, 1184, 1029. **HRMS (ESI) *m/z***: $[M+H]^+$ calcd for $C_9H_{12}O_5P$, 231.0417; found 231.0414.

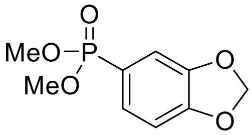

Dimethyl naphthalen-1-ylphosphonate (3c):[14] The titled compound **3c** was synthesized according to **GP-IV** by using 1-bromonaphthalene **1j** (28 μL, 0.2 mmol). The desired product **3c** was isolated after silica gel column chromatography by using 50% ethyl acetate in hexane as eluent (colourless oil, 40.5 mg, 86% yield). **^{1}H NMR** (400 MHz, $CDCl_3$): δ (ppm) 8.46 (d, *J* = 8.3 Hz, 1H), 8.23

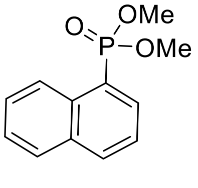

(dd, J = 16.2, 6.6 Hz, 1H), 8.05 (d, J = 7.9 Hz, 1H), 7.90 (d, J = 7.5 Hz, 1H), 7.63 – 7.60 (m, 1H), 7.57 – 7.51 (m, 2H), 3.79 (d, J = 11.3 Hz, 6H). **^{13}C {^{1}H} NMR** (101 MHz, $CDCl_3$): δ (ppm) 135.1 (d, J = 9.2 Hz), 134.0 (d, J = 3.4 Hz), 133.7 (d, J = 12.8 Hz), 132.8 (d, J = 11.0 Hz), 128.9 (d, J = 1.9 Hz), 127.8, 126.59, 126.55 (d, J = 4.4 Hz), 124.7 (d, J = 16.7 Hz), 123.3 (d, J = 183.6 Hz), 52.8 (d, J = 5.4 Hz). **^{31}P NMR** (202 MHz, $CDCl_3$): δ (ppm) 23.0.

Dimethyl phenanthren-9-ylphosphonate (3d): The titled compound **3d** was synthesized

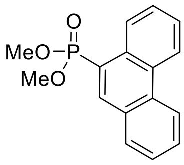

according to **GP-IV** by using 9-bromophenanthrene **1v** (52 mg, 0.2 mmol). The desired product **3d** was isolated after silica gel column chromatography by using 50% ethyl acetate in hexane as eluent (yellow solid, 36.6 mg, 64% yield). **^{1}H NMR** (400 MHz, $CDCl_3$): δ (ppm) 8.73 (d, J = 7.3 Hz, 1H), 8.69 (d, J = 8.4 Hz, 1H), 8.64 (d, J = 18.1 Hz, 1H), 8.48 (d, J = 7.6 Hz, 1H), 8.01 (d, J = 7.9 Hz, 1H), 7.77 (t, J = 7.6 Hz, 1H), 7.73 – 7.64 (m, 3H), 3.82 (d, J = 11.3 Hz, 6H). **^{13}C {^{1}H} NMR** (126 MHz, $CDCl_3$): δ (ppm) 138.7 (d, J = 8.7 Hz), 132.6 (d, J = 2.8 Hz), 130.7 (d, J = 12.1 Hz), 130.2, 130.0 (d, J = 10.2 Hz), 129.9, 129.5, 127.6, 127.47 (d, J = 3.6 Hz), 127.35, 127.3, 123.2 (d, J = 1.6 Hz), 122.9, 122.2 (d, J = 183.3 Hz), 52.9 (d, J = 5.3 Hz). **^{31}P NMR** (202 MHz, $CDCl_3$): δ (ppm) 23.3. **FTIR**: (neat)/ cm^{-1} = 3476, 2952, 2850, 1450, 1256, 1024. **HRMS (ESI)** ***m/z***: $[M+H]^+$ calcd for $C_{16}H_{16}O_3P$, 287.0832; found 287.0832.

Dimethyl (4-(1H-indol-2-yl)phenyl)phosphonate (3e): The titled compound **3e** was

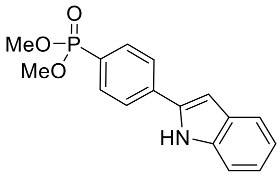

synthesized according to **GP-IV** by using methyl 2-(4-bromophenyl)-1*H*-indole **1n** (57.2mg, 0.2 mmol). The desired product **3e** was isolated after silica gel column chromatography by using 80% ethyl acetate in hexane as eluent (yellow solid, 40.4 mg, 67% yield). **^{1}H NMR** (400 MHz, $CDCl_3$): δ (ppm) 9.58 (br s, 1H), 7.80 (d, J = 8.4 Hz, 4H), 7.65 (d, J = 7.9 Hz, 1H), 7.47 (d, J = 8.2 Hz, 1H), 7.22 (t, J = 7.5 Hz, 1H), 7.13 (t, J = 7.4 Hz, 1H), 6.92 (s, 1H), 3.78 (d, J = 11.1 Hz, 6H). **^{13}C {^{1}H} NMR** (101 MHz, $CDCl_3$): δ (ppm) 137.7, 136.96 (d, J = 3.3 Hz), 136.6, 132.66 (d, J = 10.3 Hz), 129.1, 125.3 (d, J = 15.4 Hz), 124.9 (d, J = 191.9 Hz), 123.1, 121.0, 120.4, 111.5, 101.6, 53.0 (d, J = 5.6 Hz). **^{31}P NMR** (202 MHz, $CDCl_3$): δ (ppm) 22.2. **FTIR**: (neat)/ cm^{-1} = 3215, 2952, 1604, 1434, 1343, 1306, 1238, 1183, 1052, 1026. **HRMS (ESI)** ***m/z***: $[M+H]^+$ calcd for $C_{16}H_{17}NO_3P$, 302.0941; found 302.0941.

Dimethyl (1-benzyl-1H-indol-5-yl)phosphonate (3f): The titled compound **3f** was synthesized

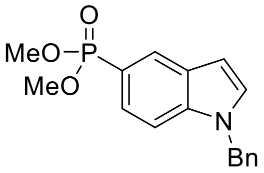

according to **GP-IV** by using 1-benzyl-5-bromo-1*H*-indole **1w** (55mg, 0.2 mmol). The desired product **3f** was isolated after silica gel column chromatography by using 60% ethyl acetate in hexane as eluent (yellow oil, 33 mg, 52% yield). **^{1}H NMR** (400 MHz, $CDCl_3$): δ (ppm) 8.18 (d, *J* = 14.4 Hz, 1H), 7.56 (dd, *J* = 11.9, 8.5 Hz, 1H), 7.37 (dd, *J* = 8.4, 3.1 Hz, 1H), 7.33 – 7.27 (m, 3H), 7.22 (d, *J* = 3.0 Hz, 1H), 7.11 (d, *J* = 7.3 Hz, 2H), 6.64 (d, *J* = 3.0 Hz, 1H), 5.35 (s, 2H), 3.75 (d, *J* = 11.1 Hz, 6H). **^{13}C {^{1}H} NMR** (126 MHz, $CDCl_3$): δ (ppm) 138.4 (d, *J* = 2.8 Hz), 136.8, 129.7, 128.9, 128.4 (d, *J* = 17.9 Hz), 127.9, 126.8, 126.7 (d, *J* = 11.3 Hz), 124.5 (d, *J* = 12.1 Hz), 116.6 (d, *J* = 192.3 Hz), 110.0 (d, *J* = 16.7 Hz), 102.9 (*J* = 1.3 Hz), 52.6 (d, *J* = 5.3 Hz), 50.28. **^{31}P NMR** (202 MHz, $CDCl_3$): δ (ppm) 25.6. **FTIR**: (neat)/ cm^{-1} = 3458, 3029, 2951, 2851, 1717, 1608, 1454, 1336, 1243,1183, 1026. **HRMS (ESI) *m/z***: $[M+H]^+$ calcd for $C_{17}H_{19}NO_3P$, 316.1097; found 316.1098.

5-Methylphenanthridin-6(5H)-one (5a):[15a] The titled compound **5a** was synthesized

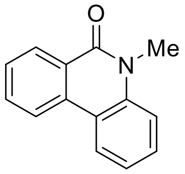

according to **GP-V** by using *N*-(2-bromophenyl)-*N*-methylbenzamide **4a** (145 mg, 0.5 mmol). The desired product **5a** was isolated after silica gel column chromatography by using 4% ethyl acetate in hexane as eluent (white solid, 93 mg, 89% yield). **^{1}H NMR** (400 MHz, $CDCl_3$): δ (ppm) 8.51 (dd, *J* = 8.0, 1.0 Hz, 1H), 8.19 (d, *J* = 8.1 Hz, 2H), 7.70 (t, *J* = 8.3 Hz, 1H), 7.54 (t, *J* = 8.0 Hz, 1H), 7.49 (t, *J* = 8.5 Hz, 1H), 7.34 (d, *J* = 8.1 Hz, 1H), 7.26 (t, *J* = 8.0 Hz, 1H), 3.75 (s, 3H). **^{13}C {^{1}H} NMR** (101 MHz, $CDCl_3$): δ (ppm) 161.6, 138.0, 133.5, 132.4, 129.6, 128.9, 128.0, 125.6, 123.2, 122.5, 121.6, 119.3, 115.0, 30.0.

5,8-Dimethylphenanthridin-6(5H)-one (5b):[15b] The titled compound **5b** was synthesized

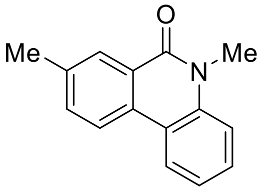

according to **GP-V** by using *N*-(2-bromophenyl)-*N*,3-dimethylbenzamide **4d** (152 mg, 0.5 mmol). The desired product **5b** was isolated after silica gel column chromatography by using 7% ethyl acetate in hexane as eluent (white solid, 61.5 mg, 55% yield). **^{1}H NMR** (400 MHz, $CDCl_3$): δ (ppm) 8.35 (s, 1H), 8.26 (d, *J* = 7.6 Hz, 1H), 8.18 (d, *J* = 8.2 Hz, 1H), 7.59 – 7.51 (m, 2H), 7.42 (d, *J* = 8.3 Hz, 1H), 7.32 (t, *J* = 7.6 Hz, 1H), 3.82 (s, 3H), 2.53 (s, 3H). **^{13}C {^{1}H} NMR** (101 MHz, $CDCl_3$): δ (ppm) 161.9, 138.3, 137.8, 133.9, 131.2, 129.2, 128.8, 125.6, 123.1, 122.6, 121.8, 119.6, 115.2, 30.2, 21.5.

5,9-Dimethylphenanthridin-6(5H)-one (5c):[15a] The titled compound **5c** was synthesized according to **GP-V** by using *N*-(2-bromophenyl)-*N*,4-dimethylbenzamide **4e** (153 mg, 0.5 mmol). The desired product **5c** was isolated after silica gel column chromatography by using 6% ethyl acetate in hexane as eluent (white solid, 73 mg, 65% yield). **^{1}H NMR** (400 MHz, $CDCl_3$): δ (ppm) 8.43 (d, *J* = 8.1 Hz, 1H), 8.28 (d, *J* = 7.7 Hz, 1H), 8.06 (s, 1H), 7.54 (t, *J* = 7.8 Hz, 1H), 7.41 (d, *J* = 8.6 Hz, 2H), 7.31 (t, *J* = 7.6 Hz, 1H), 3.81 (s, 3H), 2.56 (s, 3H). **^{13}C {^{1}H} NMR** (126 MHz, $CDCl_3$): δ (ppm) 161.9, 143.0, 138.4, 133.7, 129.6, 129.5, 129.1, 123.5, 123.3, 122.5, 121.8, 119.5, 115.2, 30.0, 22.3.

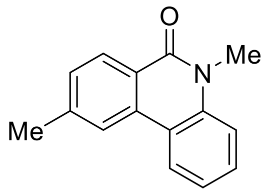

9-Methoxy-5-methylphenanthridin-6(5H)-one (5d):[15a] The titled compound **5d** was synthesized according to **GP-V** by using *N*-(2-bromophenyl)-4-methoxy-*N*-methylbenzamide **4f** (160 mg, 0.5 mmol). The desired product **5d** was isolated after silica gel column chromatography by using 20% ethyl acetate in hexane as eluent (white solid, 84 mg, 70% yield). **^{1}H NMR** (400 MHz, $CDCl_3$): δ (ppm) 8.48 (d, *J* = 8.8 Hz, 1H), 8.21 (d, *J* = 8.0 Hz, 1H), 7.64 (s, 1H), 7.55 (t, *J* = 7.7 Hz, 1H), 7.40 (d, *J* = 8.5 Hz, 1H), 7.31 (t, *J* = 7.5 Hz, 1H), 7.15 (d, *J* = 8.9 Hz, 1H), 3.99 (s, 3H), 3.79 (s, 3H). **^{13}C {^{1}H} NMR** (126 MHz, $CDCl_3$): δ (ppm) 163.1, 161.6, 138.7, 135.7, 131.3, 129.9, 123.4, 122.4, 119.6, 119.3, 116.0, 115.3, 104.7, 55.7, 29.9.

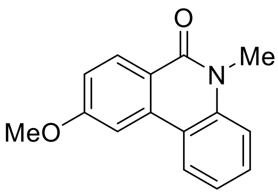

8,9-Dimethoxy-5-methylphenanthridin-6(5H)-one (5e):[15b] The titled compound **5e** was synthesized according to **GP-V** by using *N*-(2-bromophenyl)-3,4-dimethoxy-*N*-methylbenzamide **4g** (175 mg, 0.5 mmol). The desired product **5e** was isolated after silica gel column chromatography by using 25% ethyl acetate in hexane as eluent (white solid, 78 mg, 58% yield).**^{1}H NMR** (400 MHz, $CDCl_3$): δ (ppm) 8.16 (d, *J* = 8.0 Hz, 1H), 7.94 (s, 1H), 7.60 (s, 1H), 7.52 (t, *J* = 7.7 Hz, 1H), 7.42 (d, *J* = 8.4 Hz, 1H), 7.32 (t, *J* = 7.6 Hz, 1H), 4.09 (s, 3H), 4.04 (s, 3H), 3.82 (s, 3H). **^{13}C {^{1}H} NMR** (126 MHz, $CDCl_3$): δ (ppm) 161.4, 153.5, 150.1, 137.8, 128.8, 128.5, 119.9, 119.4, 109.4, 102.8, 56.4, 56.3, 30.1.

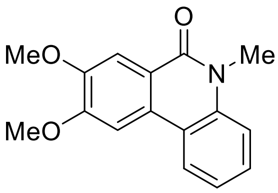

9-Fluoro-5-methylphenanthridin-6(5H)-one (5f):[15a] The titled compound **5f** was synthesized according to **GP-V** by using *N*-(2-bromophenyl)-4-fluoro-*N*-methylbenzamide **4h** (154 mg, 0.5 mmol). The desired product **5f** was isolated after silica gel column chromatography by using 5% ethyl acetate

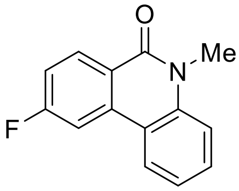

in hexane as eluent (white solid, 82 mg, 72% yield). **^{1}H NMR** (400 MHz, $CDCl_3$): δ (ppm) 8.56 (dd, *J* = 8.6, 6.3 Hz, 1H), 8.16 (d, *J* = 8.0 Hz, 1H), 7.89 – 7.86 (m, 1H), 7.59 (t, *J* = 7.8 Hz, 1H), 7.42 (d, *J* = 8.4 Hz, 1H), 7.34 (t, *J* = 7.6 Hz, 1H), 7.30 – 7.26 (m, 1H), 3.80 (s, 3H). **^{13}C {1H} NMR** (101 MHz, $CDCl_3$): δ (ppm) 165.7 (d, *J* = 252.1 Hz), 161.1, 138.6, 136.3 (d, *J* = 9.5 Hz), 132.3 (d, *J* = 9.8 Hz), 130.5, 123.6, 122.7, 122.3, 118.7 (d, *J* = 3.1 Hz), 116.4 (d, *J* = 22.9 Hz), 115.4, 107.7 (d, *J* = 23.3 Hz), 30.1. **^{19}F {^{1}H}** (471 MHz, $CDCl_3$): δ (ppm) -145.9.

2,5-Dimethylphenanthridin-6(5H)-one (5g):[15c] The titled compound **5g** was synthesized according to **GP-V** by using *N*-(2-bromo-4-methylphenyl)-*N*-methylbenzamide **4j** (153 mg, 0.5 mmol). The desired product **5g** was isolated as white solid after silica gel column chromatography by using 7% ethyl acetate in hexane as eluent (74 mg, 66% yield). **^{1}H NMR** (400 MHz, $CDCl_3$): δ (ppm) 8.55 (d, *J* = 8.0 Hz, 1H), 8.28 (d, *J* = 8.1 Hz, 1H), 8.09 (s, 1H), 7.75 (t, *J* = 7.6 Hz, 1H), 7.58 (t, *J* = 7.6 Hz, 1H), 7.37 (d, *J* = 8.6 Hz, 1H), 7.32 (d, *J* = 8.5 Hz, 1H), 3.81 (s, 3H), 2.50 (s, 3H). **^{13}C {^{1}H} NMR** (101 MHz, $CDCl_3$): δ (ppm) 161.6, 136.1, 133.6, 132.4, 132.1, 130.7, 129.1, 128.0, 125.8, 123.5, 121.7, 119.3, 115.1, 30.1, 21.1.

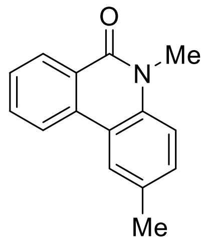

2-Methoxy-5-methylphenanthridin-6(5H)-one (5h):[15c] The titled compound **5h** was synthesized according to **GP-V** by using N-(2-bromo-4-methoxyphenyl)-N-methylbenzamide **4k** (160 mg, 0.5 mmol). The desired product **5h** was isolated as white solid after silica gel column chromatography by using 30% ethyl acetate in hexane as eluent (74.2 mg, 62% yield). **^{1}H NMR** (400 MHz, $CDCl_3$): δ (ppm) 8.56 (d, *J* = 8.0 Hz, 1H), 8.22 (d, *J* = 8.1 Hz, 1H), 7.77 – 7.74 (m, 2H), 7.59 (t, *J* = 7.5 Hz, 1H), 7.36 (d, *J* = 9.1 Hz, 1H), 7.15 (dd, *J* = 9.1, 2.8 Hz, 1H), 3.94 (s, 3H), 3.80 (s, 3H). **^{13}C {^{1}H} NMR** (126 MHz, $CDCl_3$): δ (ppm) 161.4, 155.3, 133.4, 132.6, 132.4, 129.2, 128.3, 126.1, 121.8, 120.4, 116.7, 116.4, 107.3, 55.9, 30.2.

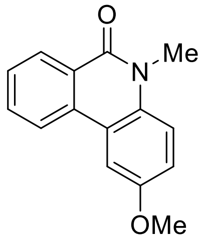

5-Methyl-2-(trifluoromethyl)phenanthridin-6(5H)-one (5i):[15d] The titled compound **5i** was synthesized according to **GP-V** by using *N*-(2-bromo-4-(trifluoromethyl)phenyl)-*N*-methylbenzamide **4l** (180 mg, 0.5 mmol). The desired product **5i** was isolated after silica gel column chromatography by using 9% ethyl acetate in hexane as eluent (white solid, 90 mg, 65% yield). **^{1}H NMR** (400 MHz, $CDCl_3$): δ (ppm) 8.55 (d, *J* = 7.9 Hz, 1H), 8.50 (s, 1H), 8.28 (d, *J* = 8.1 Hz, 1H), 7.81 (t, *J* = 7.7 Hz, 1H), 7.77 (d, *J* = 8.8 Hz, 1H), 7.65 (t, *J* = 7.6 Hz, 1H), 7.50 (d, *J* = 8.8 Hz, 1H), 3.83 (s, 3H). **^{13}C {^{1}H} NMR** (101 MHz, $CDCl_3$): δ (ppm) 161.7,

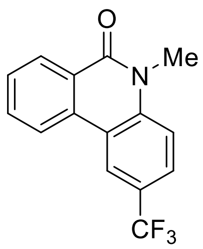

140.4, 133.0, 132.7, 129.2, 129.0, 126.2 (q, *J* = 3.5 Hz), 125.9, 124.7 (q, *J* = 33.2 Hz), 124.4 (q, *J* = 271.7 Hz), 121.9, 120.8 (q, *J* = 3.9 Hz), 119.4, 115.6, 30.4. **^{19}F {^{1}H}** NMR (471 MHz, $CDCl_3$): δ (ppm) -61.72.

1'-Methyl-3-(trifluoromethyl)spiro[cyclohexane-1,3'-indoline]-2,5-dien-2'-one (5'a): The

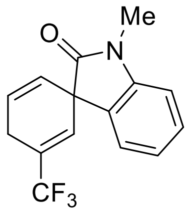

titled compound ***5'a*** was synthesized according to **GP-V** by using *N*-(2-bromophenyl)-*N*-methyl-3-(trifluoromethyl)benzamide **4i** (179 mg, 0.5 mmol). The desired product ***5'a*** was isolated after silica gel column chromatography by using 5% ethyl acetate in hexane as eluent (brown solid, 81 mg, 58% yield). **^{1}H NMR** (500 MHz, $CDCl_3$): δ (ppm) 7.36 – 7.31 (m, 1H), 7.09 – 7.08 (m, 2H), 6.88 (d, *J* = 7.8 Hz, 1H), 6.21 – 6.17 (m, 1H), 5.98 (s, 1H), 5.44 (dd, *J* = 9.9, 2.0 Hz, 1H), 3.25 (s, 3H), 3.11 (d, *J* = 22.6 Hz, 1H), 3.00 (d, *J* = 22.4 Hz, 1H). **^{13}C {^{1}H} NMR** (126 MHz, $CDCl_3$): δ (ppm) 176.1, 143.2, 132.4, 130.6, 129.4, 129.0 (q, *J* = 31.1 Hz), 126.7 (q, *J* = 5.8 Hz), 125.9, 125.1, 123.5 (q, *J* = 272.4 Hz), 123.4, 123.3, 108.5, 52.0, 26.9, 23.4. **^{19}F NMR** (471 MHz, $CDCl_3$): δ (ppm) -109.7. **FTIR**: (neat)/ cm^{-1} = 2924, 2854, 1711, 1610, 1490, 1470, 1211369, 1343, 1258, 1182, 1201. **HRMS (ESI)** ***m/z***: $[M+H]^+$ calcd for $C_{15}H_{13}F_3NO$, 280.0944; found 280.0946.

1-Methyl-4'H-spiro[indoline-3,1'-naphthalen]-2-one (5'b):[15e] The titled compound ***5'b*** was

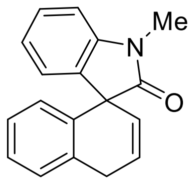

synthesized according to **GP-V** by using *N*-(2-bromophenyl)-*N*-methyl-1-naphthamide **4m** (170 mg, 0.5 mmol). The desired product ***5'b*** was isolated after silica gel column chromatography by using 5% ethyl acetate in hexane as eluent (white solid, 81 mg, 62% yield). **^{1}H** NMR (500 MHz, $CDCl_3$): δ (ppm) 7.32 (t, *J* = 7.9 Hz, 1H), 7.26 – 7.24 (d, *J* = 9.5Hz, 1H), 7.19 (t, *J* = 7.4 Hz, 1H), 7.06 – 7.01 (m, 3H), 6.94 (d, *J* = 8.5 Hz, 1H), 6.55 (d, *J* = 7.8 Hz, 1H), 6.35 – 6.31 (m, 1H), 5.57 (d, *J* = 9.8 Hz, 1H), 3.80 (d, *J* = 21.8 Hz, 1H), 3.59 (dd, *J* = 21.8, 3.8 Hz, 1H), 3.27 (s, 3H). **^{13}C {^{1}H} NMR** (126 MHz, $CDCl_3$): δ (ppm) 178.4, 143.9, 136.0, 134.5, 134.4, 128.9, 128.6, 128.2, 127.5, 127.0, 126.8, 125.6, 124.9, 123.4, 108.2, 54.9, 30.2, 26.8.

1,2-Diphenylethane-1,2-diol (7a):[11] The titled compound **7a** was synthesized according to the

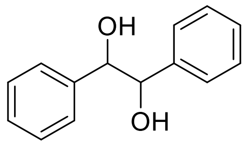

GP-VI by using benzaldehyde **6a** (51 µL, 0.5 mmol, 1.0 equiv). The reaction was continued for 2 h. The desired product **7a** was isolated after column chromatography on silica gel using 20% ethyl acetate in hexane as eluent (white solid, 45 mg, 84% yield). ***meso:dl*** 1:1.05. **^{1}H NMR** (400 MHz, $CDCl_3$): δ

(ppm) 7.33 – 7.30 (m, 6H), 7.23 – 7.22 (m, 10H), 7.11 – 7.09 (m, 4H), 4.80 (s, 2H, *meso*), 4.66 (s, 1.05 × 2H, *dl*), 3.12 (s,1.05 × 2H, *dl*), 2.46 (br s, 2H, *meso*).

1,2-Bis(2-methoxyphenyl)ethane-1,2-diol (7b):[11] The titled compound **7b** was synthesized according to the **GP-VI** by using 2-methoxybenzaldehyde **6b** (60 µL, 0.5 mmol, 1.0 equiv). The reaction was continued for 1.5 h. The desired product **7b** was isolated after column chromatography on silica gel using 30% ethyl acetate in hexane as eluent (colorless oil, 55.5 mg, 81% yield). ***meso:dl*** 1:1.1. **^{1}H NMR** (400 MHz, $CDCl_3$): δ (ppm) 7.23 – 7.15 (m 8H), 6.90 – 6.85 (m, 4H), 6.80 (d, *J* = 8.2 Hz, 2H), 6.75 (d, *J* = 8.6 Hz, 2H), 5.26 (s, 2H, *meso*), 5.04 (s, 1.1 × 2H, *dl*), 3.67 (s, 6H, *meso*), 3.64 (s, 1.1 × 6H, *dl*), 3.52 (br s, 1.1 × 2H, *dl*), 3.21 (br s, 2H, *meso*).

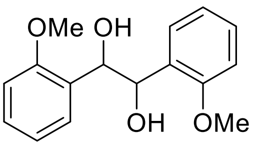

1,2-Bis(4-methoxyphenyl)ethane-1,2-diol (7c):[11] The titled compound **7c** was synthesized according to the **GP-VI** by using 4-methoxybenzaldehyde **6c** (60 µL, 0.5 mmol, 1.0 equiv). The reaction was continued for 2 h. The desired product **7c** was isolated after column chromatography on silica gel using 30% ethyl acetate in hexane as eluent (white solid, 48 mg, 70% yield). ***meso:dl*** 1:2.52. **^{1}H NMR** (400 MHz, $CDCl_3$): δ (ppm) 7.19 (dd, *J* = 8.6, 1.6 Hz, 4H), 7.02 (dd, *J* = 8.6, 1.6 Hz, 10H), 6.85 (dd, *J* = 8.6, 1.6 Hz, 4H), 6.75 (dd, *J* = 8.6, 1.7 Hz, 10H), 4.72 (s, 2H, *meso*), 4.61 (s, 2.52 × 2H, *dl*), 3.80 (s, 6H, *meso*), 3.76 (s, 2.52 × 6H, *dl*), 2.95 (br s, 2.52 × 2H, *dl*), 2.22 (br s, 2H, *meso*).

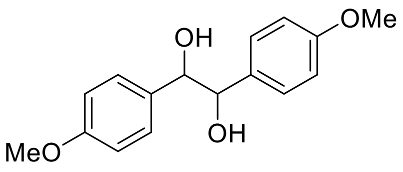

1,2-Bis(2-fluorophenyl)ethane-1,2-diol (7d): The titled compound **7d** was synthesized according to the **GP-VI** by using 2-fluorobenzaldehyde **6d** (53 µL, 0.5 mmol, 1.0 equiv). The reaction was continued for 2 h. The desired product **7d** was isolated after column chromatography on silica gel using 30% ethyl acetate in hexane as eluent (white solid, 40 mg, 64% yield). ***meso:dl*** 1:1.26. **^{1}H NMR** (400 MHz, $CDCl_3$): δ (ppm) 7.44 (t, *J* = 6.8 Hz, 2H), 7.26 – 7.25 (m, 1H), 7.23 – 7.18 (m, 6H), 7.10 (t, *J* = 7.5 Hz, 3H), 7.03 (t, *J* = 7.5 Hz, 2H), 6.93 – 6.88 (m, 4H), 5.35 (s, 2H, *meso*), 5.14 (s, 1.26 × 2H, *dl*), 2.98 (br s, 4.5H, 2H *meso* and 1.26×2 H *dl*). **^{13}C {^{1}H} NMR** (126 MHz, $CDCl_3$): δ (ppm) 161.2, 161.1, 159.3, 159.1, 129.7, 129.6, 129.41, 129.35, 128.60, 128.57, 128.44, 128.41, 127.2, 127.1, 126.6, 126.5, 124.2, 123.94, 123.92, 115.4, 115.2, 115.0, 114.8, 72.01, 70.64 (all signal for both the diastereomers are reported) **^{19}F** NMR (471 MHz, $CDCl_3$) δ (ppm) -118.16, -118.60. **FTIR**: (neat)/ cm^{-1} = 3372, 2925, 1618, 1588, 1489, 1456, 1398,

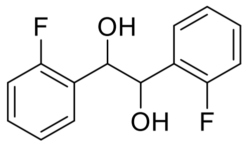

1271, 1227, 1102, 1034. **HRMS (ESI)** ***m/z***: $[M+Na]^+$ calcd for $C_{14}H_{12}F_2NaO_2$, 273.0698 found 273.0703.

1,2-Bis(4-fluorophenyl)ethane-1,2-diol (7e):[11] The titled compound **7e** was synthesized

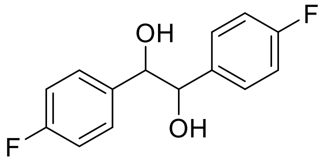

according to the **GP-VI** by using 4-fluorobenzaldehyde **6e** (54 µL, 0.5 mmol, 1.0 equiv). The reaction was continued for 2 h. The desired product **7e** was isolated after column chromatography on silica gel using 30% ethyl acetate in hexane as eluent (white solid, 62 mg, 99% yield). ***meso:dl*** 1.3:1. **^{1}H NMR** (500 MHz, $CDCl_3$): δ (ppm) 7.17 – 7.14 (m, 1.3 × 4H, *meso*), 7.07 – 7.04 (m, 4H, *dl*), 6.99 – 6.96 (m, 1.3 × 4H, *dl*), 6.93 – 6.90 (m, 4H, *meso*), 4.82 (s, 1.3 × 2H, *meso*), 4.63 (s, *dl*), 2.90 (s, 2H, *dl*), 2.31 (s, 1.3 × 2H *meso*).

4,4'-(1,2-Dihydroxyethane-1,2-diyl)dibenzonitrile (7f):[11] The titled compound **7f** was

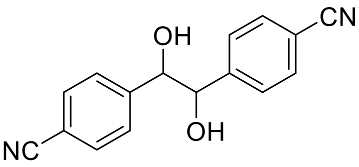

synthesized according to the **GP-VI** by using 4-formylbenzonitrile **6f** (66 mg, 0.5 mmol, 1.0 equiv). The reaction was continued for 15 h. The desired product **7f** was isolated after column chromatography on silica gel using 50% ethyl acetate in hexane as eluent (orange solid, 47 mg, 71% yield). ***meso:dl*** 1:1.5. **^{1}H NMR** (400 MHz, DMSO-d_6): δ (ppm) 7.73 (d, *J* = 7.9 Hz, 1.5 × 4H, *dl*), 7.68 (d, *J* = 7.9 Hz, 4H, *meso*), 7.43 (d, *J* = 7.8 Hz, 1.5×4H, *dl*), 7.34 (d, *J* = 7.9 Hz, 4H, *meso*), 5.75 (s, 2H, meso), 5.69 (s, 1.5 × 2H, *dl*), 4.83 (s, 2H, *meso*), 4.69 (s, 1.5 × 2H, *dl*).

2,3-Diphenylbutane-2,3-diol (7g):[11] The titled compound **7g** was synthesized according to the

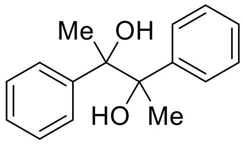

GP-VI by using acetophenone **6g** (58 µL, 0.5 mmol, 1.0 equiv). The reaction was continued for 2 h. The desired product **7g** was isolated after column chromatography on silica gel using 50% ethyl acetate in hexane as eluent (white solid, 46 mg, 76% yield). ***meso:dl*** 1:1.11. **^{1}H NMR** (400 MHz, $CDCl_3$) δ (ppm) 7.29-7.21 (m, 10H *meso* and 1.11 ×10H *dl*), 2.82 (s, 1.11 × 2H, *dl*), 2.51 (s, 2H, *meso*), 1.61 (s, 6H, *meso*), 1.52 (s, 1.11× 6H, *dl*).

2,3-Bis(4-methoxyphenyl)butane-2,3-diol (7h):[11] The titled compound **7h** was synthesized

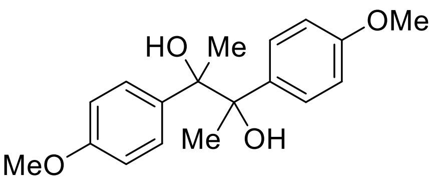

according to the **GP-VI** by using 1-(4-methoxyphenyl)ethan-1-one **6h** (75 mg, 0.5 mmol, 1.0 equiv). The reaction was continued for 2 h. The desired product **7h** was isolated after column chromatography on silica gel using 30% ethyl acetate in hexane as eluent (white solid, 48 mg, 63% yield). ***meso:dl***

1:1.2 **^{1}H NMR** (400 MHz, $CDCl_3$) δ 7.11 (dd, *J* = 12.8, 8.8 Hz, 8H), 6.76 (dd, *J* = 8.8, 6.2 Hz, 8H), 3.80 (s, 1.18 × 6H, *dl*), 3.78 (s, 6H, *meso*), 2.64 (s, 1.2 × 2H, *dl*), 2.35 (s, 2H, *meso*), 1.55 (s, 6H, *meso*), 1.46 (s, 1.2 × 6H, *dl*).

1,1,2,2-Tetraphenylethane-1,2-diol (7i):[11] The titled compound **7i** was synthesized according to the **GP-VI** by using benzophenone **6i** (91 mg, 0.5 mmol, 1.0 equiv). The reaction was continued for 2 h. The desired product **7i** was isolated after column chromatography on silica gel using 50% ethyl acetate in hexane as eluent (white solid, 87 mg, 95% yield). **^{1}H NMR** (500 MHz, $CDCl_3$) δ (ppm) 7.34 – 7.32 (m, 8H), 7.22 – 7.13 (m, 12H), 3.06 (br s, 2H).

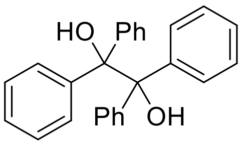

N^1,N^2-Dibenzyl-1,2-diphenylethane-1,2-diamine (9a):[11] The titled compound **9a** was synthesized according to the **GP-VII** by using (*E*)-*N*-benzyl-1-phenylmethanimine **8a** (98 mg, 0.5 mmol, 1.0 equiv). The reaction was continued for 2 h. The desired product **9a** was isolated after column chromatography on neutral activated aluminium oxide using 2% ethyl acetate in hexane as eluent (white solid, 87 mg, 89% yield). The ratio ***meso:dl*** 1:1.82 was determined by ^{1}H NMR analysis of the crude reaction mixture.**^{1}H NMR** (400 MHz, $CDCl_3$) δ (ppm): δ 7.36 – 7.28 (m, 10H), 7.24 – 7.15 (m, 6H), 6.98 (d, *J* = 6.6 Hz, 4H), 3.76 (s, 2H), 3.55 (d, *J* = 13.8 Hz, 2H), 3.31 (d, *J* = 13.7 Hz, 2H), 1.68 (s, 2H).

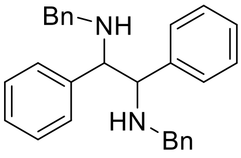

N^1,N^2-Dibenzyl-1,2-bis(2-methoxyphenyl)ethane-1,2-diamine (9b):[11] The titled compound **9b** was synthesized according to the **GP-VII** by using (*E*)-*N*-benzyl-1-(2-methoxyphenyl)methanimine **8b** (113 mg, 0.5 mmol, 1.0 equiv). The reaction was continued for 1.5 h. The desired product **9b** was isolated after column chromatography on neutral activated aluminium oxide using 5% ethyl acetate in hexane as eluent (greenish yellow liquid, 59 mg, 52% yield, major diastereomer). The ratio ***meso:dl*** 1:1.23 was determined by ^{1}H NMR analysis of the crude reaction mixture. **^{1}H NMR** (400 MHz, $CDCl_3$): δ (ppm) δ 7.28 – 7.18 (m, 14H), 6.88 (t, *J* = 7.0 Hz, 2H), 6.79 (d, *J* = 7.9 Hz, 2H), 4.53 (s, 2H), 3.65 (d, *J* = 13.3 Hz, 2H), 3.56 (s, 6H), 3.48 (d, *J* = 13.4 Hz, 2H), 2.04 (s, 2H).

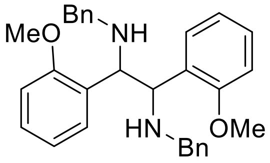

N^1,N^2-dibenzyl-1,2-bis(4-methoxyphenyl)ethane-1,2-diamine (9c):[11] The titled compound **9c** was synthesized according to the **GP-VII** by using (*E*)-*N*-benzyl-1-(4-methoxyphenyl)methanimine **8c** (113 mg, 0.5 mmol, 1.0 equiv). The reaction was continued for 2 h. The

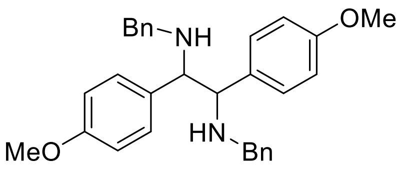

desired product **9c** was isolated after column chromatography on neutral activated aluminium oxide using 10% ethyl acetate in hexane as eluent (white solid, 77 mg, 68% yield). ***meso:dl*** 1:2.38. **^{1}H NMR** (500 MHz, $CDCl_3$): δ (ppm) 7.25 – 7.08 (m, 34H), 6.91 – 6.88 (m, 13H), 6.80 (d, *J* = 7.3 Hz, 10H), 6.64 (d, *J* = 8.2 Hz, 3H), 3.75 (s, 14H), 3.72 (s, 1H), 3.67 (s, 5H), 3.58 (d, *J* = 10.0 Hz, 8H), 3.46 (d, *J* = 13.8 Hz, 5H), 3.41 (d, *J* = 13.4 Hz, 2H, *meso*), 3.21 (d, *J* = 13.8 Hz, 2 × 2.38 H, *dl*), 1.76(br s, 8H).

Dimethyl 4,4'-(1,2-bis(benzylamino)ethane-1,2-diyl)dibenzoate (9d):[11] The titled compound

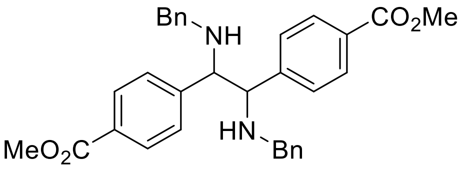

9d was synthesized according to the **GP-VII** by using methyl (*E*)-4-((benzylimino)methyl)benzoate **8d** (134 mg, 0.5 mmol, 1.0 equiv). The reaction was continued for 2 h. The desired product **9d** was isolated after column chromatography on neutral activated aluminium oxide using 15% ethyl acetate in hexane as eluent (white solid, 96.5 mg, 76% yield). ***meso:dl*** 1:1.36. **^{1}H NMR** (400 MHz, $CDCl_3$): δ (ppm) 7.99 (d, *J* = 8.4 Hz, 5H), 7.85 (d, *J* = 8.4 Hz, 4H), 7.35 – 7.20 (m, 26H), 7.11 (d, *J* = 8.3 Hz, 4H), 7.04 (d, *J* = 7.9 Hz, 5H), 3.94 (s, 8H), 3.90 (s, 3H), 3.88 (s, 4H), 3.78 (s, 2H), 3.65 (d, *J* = 13.2 Hz, 2H), 3.58 (d, *J* = 13.6 Hz, 3H), 3.49 (d, *J* = 13.3 Hz, 2H, *meso*), 3.35 (d, *J* = 13.6 Hz, 1.36×2H, *dl*), 2.08 (br s, 5H).

N1,N2-Dibenzyl-1,2-di(pyridin-3-yl)ethane-1,2-diamine (9e):[11] The titled compound **9e** was

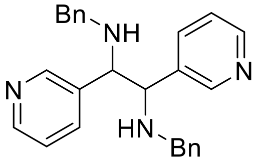

synthesized according to the **GP-VII** by using methyl (*E*)-4-((benzylimino)methyl)benzoate **8d** (98 mg, 0.5 mmol, 1.0 equiv). The reaction was continued for 2 h. The desired product **9e** was isolated after column chromatography on neutral activated aluminium oxide using 40% ethyl acetate in hexane as eluent (white solid, 114 mg, 58% yield) ***meso:dl*** 1:2.86. **^{1}H NMR** (400 MHz, $CDCl_3$): δ (ppm) 8.53 – 8.51 (m, 6H), 8.40 – 8.37 (m, 7H), 8.21 (s, 1H), 7.46 – 7.39 (m, 8H), 7.29 – 7.19 (m, 34H), 7.10 – 7.08 (m, 14H), 3.88 (s, 2.86× 2H *dl*), 3.75 (s, 2H, meso), 3.68 (d, *J* = 13.4 Hz, 2H, meso), 3.61 (d, *J* = 13.4 Hz, 2.86× 2H *dl*), 3.51 (d, *J* = 13.2 Hz, 2H, *meso*), 3.39 (d, *J* = 13.5 Hz, 2.86× 2H, *dl*), 2.34 (br s, 7H).

N*[1]*,N*[2]*-Bis(3-chlorophenyl)-1,2-bis(4-methoxyphenyl)ethane-1,2-diamine (9f):[11] The titled compound **9f** was synthesized according to the **GP-VII** by using (*E*)-*N*-(3-chlorophenyl)-1-(4-methoxyphenyl) methanimine **8f** (123 mg, 0.5 mmol, 1.0 equiv). The reaction was continued for 2 h. The desired product **9f** was isolated after column chromatography on neutral activated aluminium oxide using 2% ethyl acetate in hexane as eluent (gummy liquid, 118 mg, 96% yield). ***meso:dl*** 1.01:1. **^{1}H NMR** (400 MHz, $CDCl_3$): δ (ppm) 7.02 – 6.96 (m, 8H), 6.86 (d, *J* = 8.3 Hz, 4H), 6.80 – 6.77 (m, 8H), 6.64 (d, *J* = 7.8 Hz, 4H), 6.51 (s, 4H), 6.38 (d, *J* = 8.2 Hz, 4H), 4.83 (d, *J* = 7.4 Hz, 1.01 × 2H, meso), 4.54 (d, *J* = 7.8 Hz, 1.01 × 2H, *meso* and 2H *dl*), 4.48 (br s, 2H), 3.79 (s, 1.01 × 6H, *meso*), 3.77 (s, 6H, *dl).*

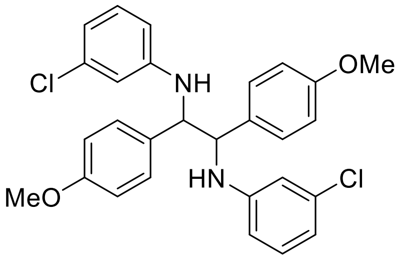

1H-Indole (12a):[12] The titled compound **12a** was synthesized according to **GP-VIII** by using 1-tosyl-1*H*-indole **10a**, **10f-10h** (0.5 mmol, 1.0 equiv). The reactions were continued for 1.5-4.5 h. The desired product **12a** was isolated after silica gel column chromatography by using 5% ethyl acetate in hexane as eluent (white solid, 85-97% yields).**^{1}H NMR** (400 MHz, $CDCl_3$): δ (ppm) 8.11 (br s, 1H), 7.74 (d, *J* = 7.8 Hz, 1H), 7.44 (d, *J* = 8.1 Hz, 1H), 7.28 (t, *J* = 7.5 Hz, 1H), 7.24 (s, 1H), 7.21 (t, *J* = 7.7 Hz, 1H), 6.63 (s, 1H).

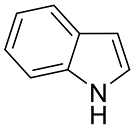

5-Methoxy-1H-indole (12b):[12] The titled compound **12b** was synthesized according to **GP-VIII** by using 5-methoxy-1-tosyl-1*H*-indole **10b** (150 mg, 0.5 mmol, 1.0 equiv). The reaction was continued for 3 h. The desired product **12b** was isolated after silica gel column chromatography by using 5% ethyl acetate in hexane as eluent (white solid, 67.6 mg, 92% yield). **^{1}H NMR** (500 MHz, $CDCl_3$): δ (ppm) 8.02 (br s, 1H), 7.24 (d, *J* = 7.9 Hz, 1H), 7.15 (t, *J* = 2.8 Hz, 1H), 7.08 (d, *J* = 2.3 Hz, 1H), 6.84 (dd, *J* = 8.8, 2.4 Hz, 1H), 6.45 (s, 1H), 3.83 (s, 3H).

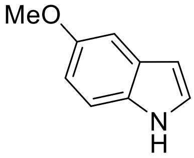

1H-Indole-5-carbonitrile (12c):[12] The titled compound **12c** was synthesized according to **GP-VIII** by using 1-tosyl-1*H*-indole-5-carbonitrile **10c** (148 mg, 0.5 mmol, 1.0 equiv). The reaction was continued for 3 h. The desired product **12c** was isolated after silica gel column chromatography by using 10% ethyl acetate in hexane as eluent (yellow solid, 64 mg, 90% yield). **^{1}H NMR** (400 MHz, $CDCl_3$): δ (ppm) 8.72 (br s, 1H), 8.00 (s, 1H), 7.48 (d, *J* = 8.5 Hz, 1H), 7.42 (d, *J* = 8.5 Hz, 1H), 7.36 – 7.35 (m, 1H), 6.64 – 6.63 (m, 1H).

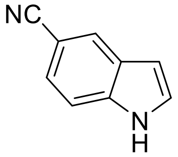

4-Bromo-1H-indole (12d):[12] The titled compound **12d** was synthesized according to **GP-VIII** by using 4-bromo-1-tosyl-1*H*-indole **10d** (175 mg, 0.5 mmol, 1.0 equiv) as prototype. The reaction was continued for 4.5 h. The desired product **12d** was isolated after silica gel column chromatography by using 5% ethyl acetate in hexane as eluent (liquid, 83.3 mg, 85% yield). **^{1}H NMR** (400 MHz, $CDCl_3$): δ (ppm) 8.23 (br s, 1H), 7.35 – 7.32 (m, 2H), 7.23 (s, 1H), 7.09 (t, *J* = 7.8 Hz, 1H), 6.65 (s, 1H).

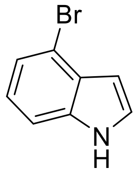

1-(1H-Indol-3-yl)ethan-1-one (12e):[12] The titled compound **12e** was synthesized according to **GP-VIII** by using 1-(1-(phenylsulfonyl)-1*H*-indol-3-yl)ethan-1-one **10e** (149 mg, 0.5 mmol, 1.0 equiv). The reaction was continued for 2 h. The desired product **12e** was isolated after silica gel column chromatography by using 60% ethyl acetate in hexane as eluent (yellow solid, 59.5 mg, 75% yield). **^{1}H NMR** (400 MHz, $CDCl_3$): δ (ppm) 8.91 (br s, 1H), 8.42 – 8.38 (m, 1H), 7.88 – 7.87 (m, 1H), 7.44 – 7.40 (m, 1H), 7.32 – 7.27 (m, 2H), 2.56 (s, 3H).

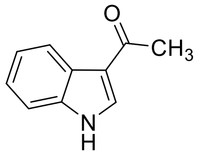

9H-Carbazole (12f):[12] The titled compound **12f** was synthesized according to **GP-VIII** by using 9-tosyl-9*H*-carbazole **10k** (160 mg, 0.5 mmol, 1.0 equiv). The reaction was continued for 10 h. The desired product **12f** was isolated after silica gel column chromatography by using 5% ethyl acetate in hexane as eluent (white solid, 62.7 mg, 75% yield). **^{1}H NMR** (400 MHz, DMSO-d_6): δ (ppm) 11.24 (br s, 1H), 8.11 (d, *J* = 7.5 Hz, 2H), 7.48 (d, *J* = 8.0 Hz, 2H), 7.38 (t, *J* = 7.2 Hz, 2H), 7.15 (t, *J* = 7.1 Hz, 2H).

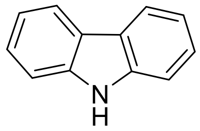

1H-Imidazole (12g):[12] The titled compound **12g** was synthesized according to **GP-VIII** by using 1-tosyl-1*H*-imidazole **10l** (111 mg, 0.5 mmol, 1.0 equiv). The reaction was continued for 2.5 h. The desired product **12g** was isolated after silica gel column chromatography by using DCM as eluent (white solid, 27 mg, 80% yield). **^{1}H NMR** (400 MHz, $CDCl_3$) δ (ppm) 11.17 (br s, 1H), 7.72 (s, 1H), 7.13 – 7.10 (m, 2H).

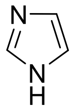

1H-1,2,4-Triazole (12h):[12] The titled compound **12h** was synthesized according to **GP-VIII** by using 1-tosyl-1*H*-imidazole **10m** (112 mg, 0.5 mmol, 1.0 equiv). The reaction was continued for 2.5 h. The desired product **12h** was isolated after silica gel column chromatography by using DCM as eluent (white solid, 29.3 mg, 85% yield). **^{1}H NMR** (400 MHz, $CDCl_3$) δ 8.23 (s, 2H), 5.89 (br s, 1H).

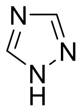

1H-Benzo[d][1,2,3]triazole (12i):[12] The titled compound **12i** was synthesized according to

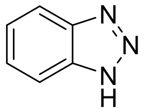

GP-VIII by using 1-tosyl-1*H*-benzo[*d*][1,2,3]triazole **10n** (136 mg, 0.5 mmol, 1.0 equiv). The reaction was continued for 1 h. The desired product **12i** was isolated after silica gel column chromatography by using 25% ethyl acetate in hexane as eluent (white solid, 45.2 mg, 76% yield). **^{1}H NMR** (500 MHz, $CDCl_3$): δ (ppm) 13.33 (br s, 1H), 7.94 (dd, *J* = 6.3, 3.0 Hz, 2H), 7.42 (d, *J* = 6.1 Hz, 2H).

N-Benzylbenzamide (12j):[12] The titled compound **12j** was synthesized according to **GP-VIII**

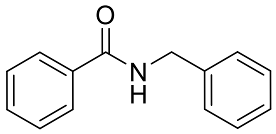

by using *N*-benzyl-*N*-tosylbenzamide **10o** (182 mg, 0.5 mmol, 1.0 equiv). The reaction was continued for 3.5 h The desired product **12j** was isolated after silica gel column chromatography by using 15% ethyl acetate in hexane as eluent (white solid, 91.7 mg, 87% yield). **^{1}H NMR** (400 MHz, $CDCl_3$): δ (ppm) 7.81–7.78 (m, 2H), 7.52–7.48 (m, 1H), 7.45 – 7.40 (m, 2H), 7.36 – 7.35 (m, 4H), 7.33 – 7.28 (m, 1H), 6.47 (br s, 1H), 4.65 (d, *J* = 5.7 Hz, 2H).

N-Benzyl-4-methylbenzenesulfonamide (12k):[12] The titled compound **12k** was synthesized

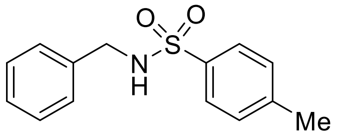

according to **GP-VIII** by using *N*-benzyl-4-methyl-*N*-tosylbenzenesulfonamide **10p** (207 mg, 0.5 mmol, 1.0 equiv). The reaction was continued for 1.5 h. The desired product **12k** was isolated after silica gel column chromatography by using 10% ethyl acetate in hexane as eluent (white solid, 116 mg, 89% yield). **^{1}H NMR** (400 MHz, $CDCl_3$): δ (ppm) 7.79 – 7.76 (m, 2H), 7.34 – 7.27 (m, 5H), 7.22 (s, 2H), 4.86 (br s, 1H), 4.15 – 4.12 (m, 2H), 2.45 (s, 3H).

N-(2-Bromo-4-methylphenyl)benzamide (12l):[10c] The titled compound **12l** was synthesized

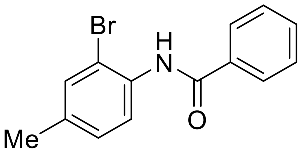

according to **GP-VIII** by using *N*-(2-bromo-4-methylphenyl)-*N*-tosylbenzamide **10q** (221 mg, 0.5 mmol, 1.0 equiv). The reaction was continued for 2 h. The desired product **12l** was isolated after silica gel column chromatography by using 5% ethyl acetate in hexane as eluent (white solid, 123 mg, 85% yield). **^{1}H NMR** (400 MHz, $CDCl_3$): δ (ppm) 8.40 (d, *J* = 8.5 Hz, 1H), 8.39 (br s, 1H), 7.93 (d, *J* = 7.4 Hz, 2H), 7.58 (t, *J* = 7.2 Hz, 1H), 7.52 (t, *J* = 7.3 Hz, 2H), 7.41 (s, 1H), 7.18 (d, *J* = 8.4 Hz, 1H), 2.33 (s, 3H).

Naphthalen-2-ol (12m):[12] The titled compound **12m** was synthesized according to **GP-VIII**

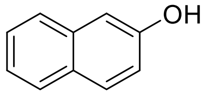

by using naphthalen-2-yl 4-methylbenzenesulfonate **10r** (149 mg, 0.5 mmol, 1.0 equiv). The reaction was continued for 12 h. The desired product **12m** was isolated after silica gel column chromatography by using 10% ethyl acetate in hexane as

eluent (white solid, 50.4 mg, 70% yield). **^{1}H NMR** (400 MHz, $CDCl_3$): δ (ppm) 7.78 (t, *J* = 8.3 Hz, 2H), 7.69 (d, *J* = 8.2 Hz, 1H), 7.45 (t, *J* = 7.5 Hz, 1H), 7.35 (t, *J* = 7.5 Hz, 1H), 7.16 (d, *J* = 1.4 Hz, 1H), 7.12 (dd, *J* = 8.8, 2.4 Hz, 1H), 5.23 (br s, 1H).

Methyl N^2,N^6-bis(tert-butoxycarbonyl)-L-lysyl-L-tryptophanate (13a):[12] The titled compound

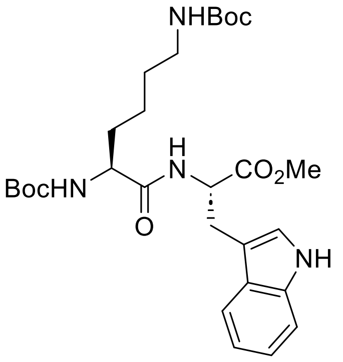

13a was synthesized according to **GP-IX** by using methyl N^a-(N^2,N^6-bis(tert-butoxycarbonyl)-*L*-lysyl)-1-tosyl-*L*-tryptophanate **12a** (70 mg, 0.1 mmol, 1.0 equiv). The reaction was continued for 2 h The desired product **11a** was isolated as after silica gel column chromatography by using 2% MeOH in DCM as eluent (yellow semiliquid, 49 mg, 90% yield). **^{1}H** NMR (500 MHz, DMSO-d_6): δ (ppm) 10.86 (s, 1H), 8.12 (d, *J* = 7.4 Hz, 1H), 7.47 (d, *J* = 7.9 Hz, 1H), 7.33 (d, *J* = 8.1 Hz, 1H), 7.15 – 7.11 (m, 1H), 7.06 (t, *J* = 7.9 Hz, 1H), 6.98 (t, *J* = 7.7 Hz, 1H), 6.77 – 6.69 (m, 2H), 4.51 (dd, *J* = 13.7, 7.1 Hz, 1H), 3.91 (dd, *J* = 13.1, 8.4 Hz, 1H), 3.54 (s, 3H), 3.14 (dd, *J* = 14.6, 5.8 Hz, 1H), 3.07 (dd, *J* = 14.7, 7.6 Hz, 1H), 2.89 – 2.84 (m, 2H), 1.56 – 1.41 (m, 2H), 1.37 (s, 18H), 1.23 – 1.16 (m, 4H). **HRMS (ESI) *m/z***: $[M+H]^+$ calcd for $C_{28}H_{43}N_4O_7$, 547.3126; found 547.3127.

Methyl (tert-butoxycarbonyl)-L-phenylalanyl-L-tryptophyl-L-leucyl-L-tyrosinate (13b):[12]

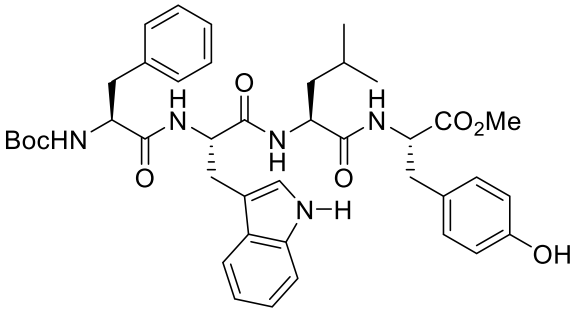

The titled compound **13b** was synthesized according to **GP-IX** by using methyl N^a-((tert-butoxycarbonyl)-*L*-phenylalanyl)-1-tosyl-*L*-tryptophyl-*L*-leucyl-*L*-tyrosinate **11b** (90 mg, 0.1 mmol, 1.0 equiv). The reaction was continued for 2 h. The desired product **13b** was isolated after silica gel column chromatography by using 5% MeOH in DCM as eluent (white solid, 67.4 mg, 91% yield). **^{1}H NMR** (400 MHz, DMSO-d_6): δ (ppm) 10.80 (s, 1H), 9.25 (s, 1H), 8.23 (d, *J* = 7.0 Hz, 1H), 8.08 (d, *J* = 8.3 Hz, 1H), 7.95 (d, *J* = 7.7 Hz, 1H), 7.59 (d, *J* = 7.8 Hz, 1H), 7.31 (d, *J* = 8.1 Hz, 1H), 7.20 – 7.11 (m, 6H), 7.05 (t, *J* = 7.5 Hz, 1H), 6.98 (t, *J* = 7.5 Hz, 2H), 6.96 – 6.90 (m, 2H), 6.66 (d, *J* = 7.6 Hz, 2H), 4.60 (dd, *J* = 12.9, 7.6 Hz, 1H), 4.41 – 4.37 (m, 2H), 4.13 – 4.07 (m, 1H), 3.54 (s, 3H), 3.11 (dd, *J* = 14.6, 4.2 Hz, 1H), 2.97 (dd, *J* = 14.7, 8.4 Hz, 1H), 2.91 – 2.81 (m, 3H), 2.68 – 2.62 (m, 1H), 1.58 – 1.51 (m, 1H), 1.42 – 1.38 (m, 2H), 1.27 (s, 9H), 0.86 (d, *J* = 6.2 Hz, 3H), 0.82 (d, *J* = 6.1 Hz, 3H). **HRMS (ESI) *m/z***: $[M+H]^+$ calcd for $C_{41}H_{52}N_5O_8$, 742.3810; found 742.3813.

Methyl N^2,N^6-bis(tert-butoxycarbonyl)-L-lysyl-L-tyrosyl-L-methionyl-D-alloisoleucyl-L-tryptophyl-L-phenylalaninate (13c):[12]

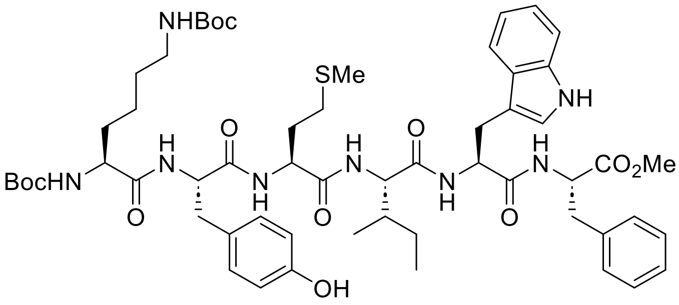

The titled compound **13c** was synthesized according to **GP-IX** by using methyl N^a-N^2,N^6-bis(tert-butoxycarbonyl)-*L*-lysyl-*L*-tyrosyl-*L*-methionyl-*D*-alloisoleucyl-1-tosyl-*L*-tryptophyl-*L*-phenylalaninate **11c** (125 mg, 0.5 mmol, 1.0 equiv). The reaction was continued for 2 h. The desired product **13c** was isolated after silica gel column chromatography by using 3% MeOH in DCM as eluent (99 mg, yellow foamy solid, 90% yield). **^{1}H NMR** (400 MHz, DMSO-*d*6): δ (ppm) 10.79 (s, 1H), 9.13 (s, 1H), 8.41 – 8.36 (m, 1H), 8.20 – 8.10 (m, 1H), 8.00 (d, *J* = 8.2 Hz, 1H), 7.78 (d, *J* = 8.5 Hz, 1H), 7.68 – 7.61 (m, 1H), 7.54 (d, *J* = 7.8 Hz, 1H), 7.31 (d, *J* = 8.1 Hz, 1H), 7.23 (d, *J* = 6.9 Hz, 2H), 7.19 (d, *J* = 7.1 Hz, 1H), 7.15 (d, *J* = 7.5 Hz, 2H), 7.07 – 7.03 (m, 2H), 6.97 – 6.92 (m, 3H), 6.86 (t, *J* = 8.3 Hz, 1H), 6.74 – 6.73 (m, 1H), 6.59 (d, *J* = 7.4 Hz, 2H), 4.63 – 4.56 (m, 1H), 4.47 – 4.35 (m, 3H), 3.79 – 3.77 (m, 1H), 3.54 – 3.50(m, 3H), 3.07 – 2.78 (m, 8H), 2.71 – 2.58 (m, 1H), 2.40 – 2.32 (m, 1H), 1.97 – 1.96 (m, 2H), 1.91 – 1.65 (m, 3H), 1.36 (s, 18H), 1.27 – 1.17 (m, 8H), 1.04 – 0.98 (m, 2H), 0.75 (t, *J* = 6.9 Hz, 3H), 0.71 (d, *J* = 6.6 Hz, 3H). **HRMS (ESI) *m/z***: $[M+H]^+$ calcd for $C_{57}H_{81}N_8O_{12}S$, 1101.5689; found 1101.5696.

Methyl N^2,N^6-bis(tert-butoxycarbonyl)-L-lysyl-L-tyrosyl-L-methionyl-L-alloisoleucyl-L-tryptophyl-L-alloisoleucinate (13d):[12]

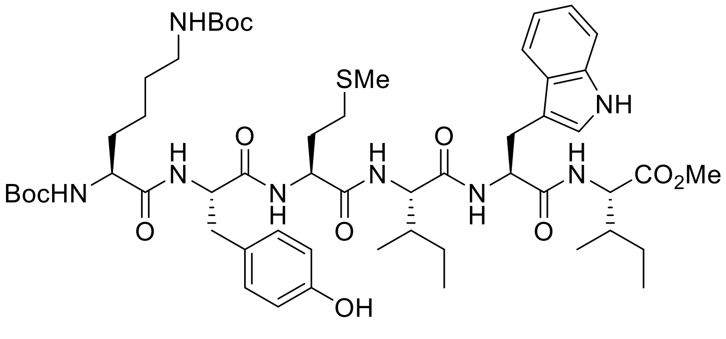

The titled compound **13d** was synthesized according to **GP-IX** by using methyl N^a-N^2,N^6-bis(tert-butoxycarbonyl)-*L*-lysyl-*L*-tyrosyl-*L*-methionyl-*L*-alloisoleucyl-1-tosyl-*L*-tryptophyl-*L*-alloisoleucinate **11d** (122 mg, 0.1 mmol, 1.0 equiv). The reaction was continued for 3 h. The desired product **13d** was isolated after silica gel column chromatography by using 5% MeOH in DCM as eluent (yellow foamy solid, 95 mg, 89% yield). **^{1}H NMR** (400 MHz, DMSO-*d*6): δ (ppm) 10.81 (br s, 1H), 9.11 (s, 1H), 8.32 – 8.07 (m, 3H), 7.62 (d, *J* = 7.9 Hz, 1H), 7.54 (d, *J* = 7.7 Hz, 1H), 7.30 (d, *J* = 8.1 Hz, 1H), 7.10 (s, 1H), 7.04 (t, *J* = 7.5 Hz, 1H), 6.97 – 6.92 (m, 4H), 6.86 (d, *J* = 7.5 Hz, 1H), 6.75 – 6.72 (m, 1H), 6.59 – 6.55 (m, 2H), 4.68 – 4.62 (m, 1H), 4.46 – 4.40 (m, 1H), 4.25 – 4.19 (m, 1H), 3.80 – 3.74 (m, 1H), 3.62–3.57 (m, 4H), 3.51 – 3.50

(m, 1H), 3.08 (dd, *J* = 14.9, 5.9 Hz, 1H), 2.94 (dd, *J* = 14.3, 7.6 Hz, 1H), 2.87 – 2.80 (m, 4H), 2.67 – 2.62 (m, 1H), 1.81 – 1.65(m, 2H), 1.36 – 1.16 (m, 34H), 0.85 – 0.75 (m, 12H). **HRMS (ESI) *m/z***: $[M+H]^+$ calcd for $C_{54}H_{83}N_8O_{12}S$, 1067.5846; found 1067.5874.

(3S,6S)-3-((1H-Indol-3-yl)methyl)-6-benzylpiperazine-2,5-dione (13e):[12] The titled compound **13e** was synthesized according to **GP-IX** by using (3S,6S)-3-benzyl-6-((1-tosyl-1*H*-indol-3-yl)methyl)piperazine-2,5-dione **11e** (49 mg, 0.1 mmol, 1.0 equiv). The reaction was continued for 4 h. The desired product **13e** was isolated after silica gel column chromatography by using 4% MeOH in DCM as eluent (white solid, 30 mg, 89% yield).

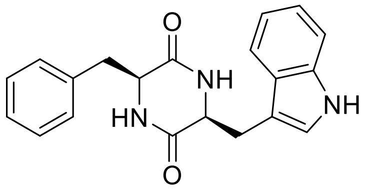

^{1}H NMR (400 MHz, DMSO-d_6): δ 10.91 (s, 1H), 7.94 (s, 1H), 7.73 (s, 1H), 7.49 (d, *J* = 7.9 Hz, 1H), 7.32 (d, *J* = 8.1 Hz, 1H), 7.20 – 7.14 (m, 3H), 7.07 (t, *J* = 7.5 Hz, 1H), 7.00 (d, *J* = 7.5 Hz, 1H), 6.96 (s, 1H), 6.70 (d, *J* = 7.3 Hz, 2H), 3.97 (s, 1H), 3.85 (s, 1H), 2.80 (dd, *J* = 14.4, 4.3 Hz, 1H), 2.54 (d, *J* = 5.8 Hz, 1H), 2.46 (dd, *J* = 13.5, 4.6 Hz, 1H), 1.84 (dd, *J* = 13.4, 7.1 Hz, 1H). **HRMS (ESI) *m/z***: $[M+H]^+$ calcd for $C_{20}H_{20}N_3O_2$, 334.1550; found 334.1555.

Computational Studies:

All the calculations were performed with the Gaussian 16, Revision B.01 program package.[16] We selected the hybrid density functional B3LYP[17] together with Pople's 6-311+G** triple-ξ valence basis set,[17] with diffuse function on heavy atoms and polarization function on all atoms, considering the Grimme's D3 (BJ-damping) dispersion effect. The SMD solvent model[18] was used to simulate the implicit solvent effect. Here, acetonitrile was utilized as the solvent. In all cases, ultrafine integral grid was employed. Harmonic vibrational frequencies were computed at the same level of theory to verify the optimized geometries as minima with all real modes in the Hessian matrix. TD-DFT calculations were conducted with the optimized geometries, at the same level of theory, with n states =5 and root =1 (Figure S14).

	S_0	S_1	T_1
**LUMO**	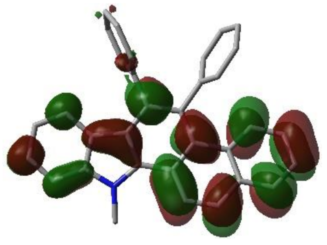	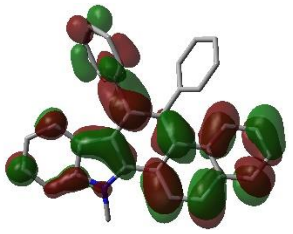	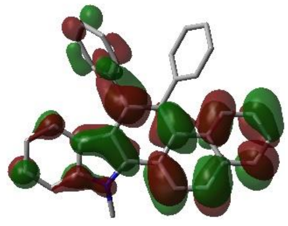
**HOMO**	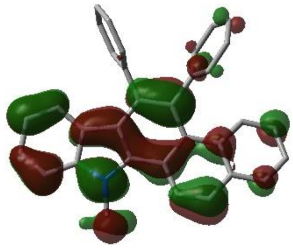	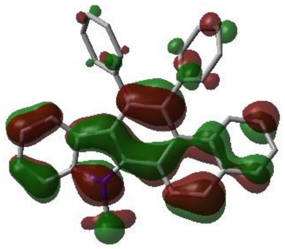	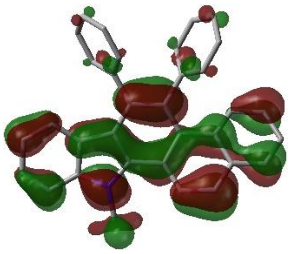

Figure S14. DFT (B3LYP-D3(BJ)/6-311+G**/SMD(ε=MeCN)) optimized geometries and calculated frontier MOs for NC1 in S_0, S_1 and T_1 states. HOMO (Highest Occupied Molecular Orbital); LUMO (Lowest Unoccupied Molecular Orbital); Color coding: N(blue), C(grey).

	S_0	S_1	T_1
**LUMO**	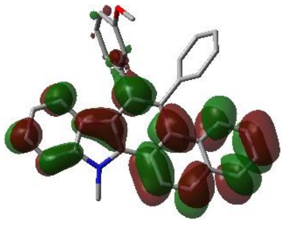	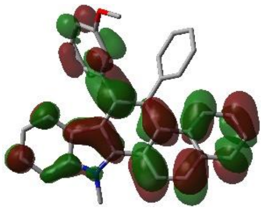	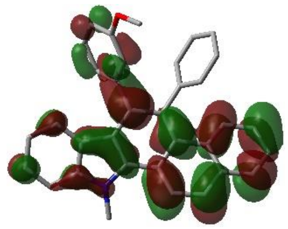
**HOMO**	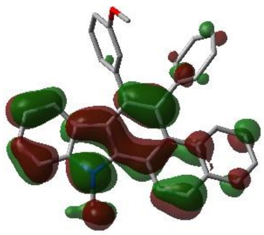	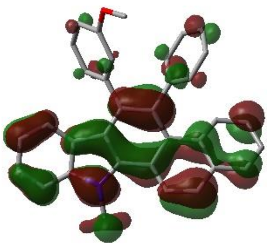	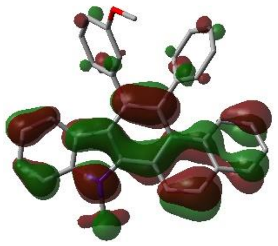

Figure S15. DFT (B3LYP-D3(BJ)/6-311+G**/SMD(ε=MeCN)) optimized geometries and calculated frontier MOs for NC2 in S_0, S_1 and T_1 states. HOMO (Highest Occupied Molecular Orbital); LUMO (Lowest Unoccupied Molecular Orbital); Color coding: N(blue), C(grey), O(red).

	S_0	S_1	T_1
**LUMO**	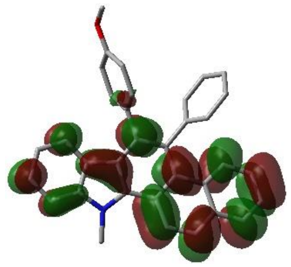	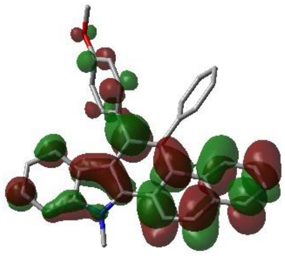	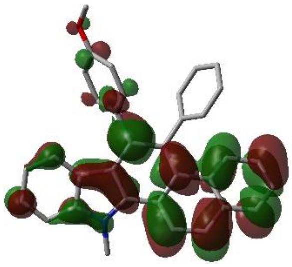
**HOMO**	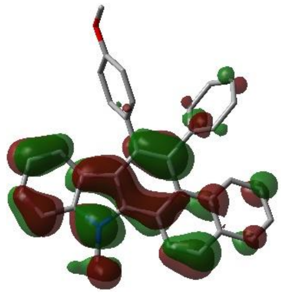	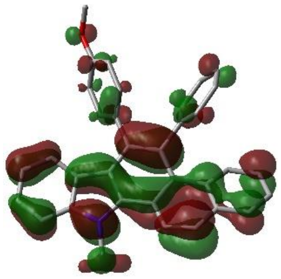	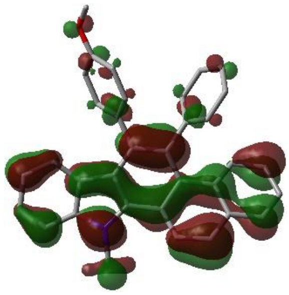

Figure S16. DFT (B3LYP-D3(BJ)/6-311+G**/SMD(ε=MeCN)) optimized geometries and calculated frontier MOs for NC3 in S_0, S_1 and T_1 states. HOMO (Highest Occupied Molecular Orbital); LUMO (Lowest Unoccupied Molecular Orbital); Color coding: N(blue), C(grey), O(red).

	S_0	S_1	T_1
**LUMO**	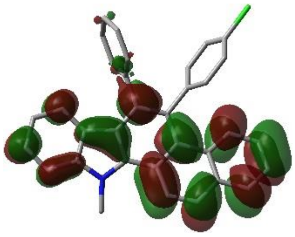	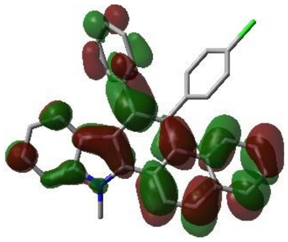	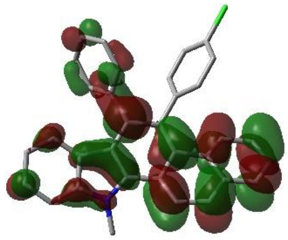
**HOMO**	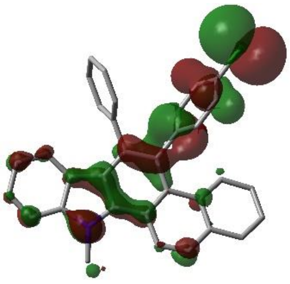	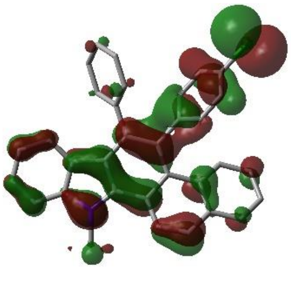	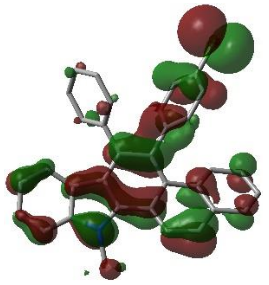

Figure S17. DFT (B3LYP-D3(BJ)/6-311+G**/SMD(ε=MeCN)) optimized geometries and calculated frontier MOs for NC4 in S_0, S_1 and T_1 states. HOMO (Highest Occupied Molecular Orbital); LUMO (Lowest Unoccupied Molecular Orbital); Color coding: N(blue), C(grey), Cl(green).

	**S_0**	**S_1**	**T_1**
**LUMO**	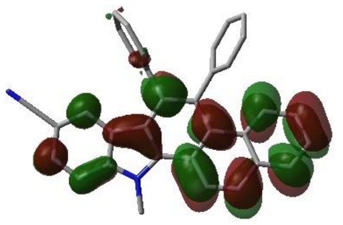	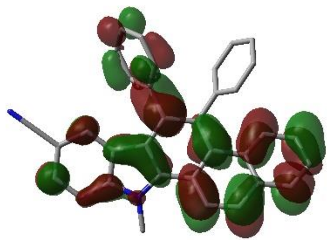	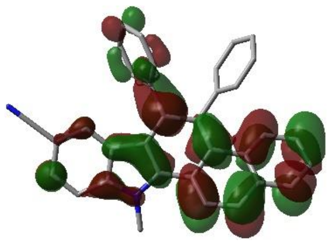
**HOMO**	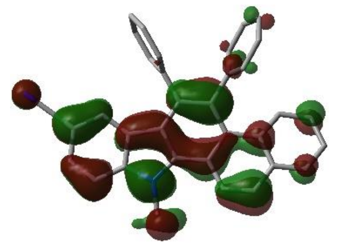	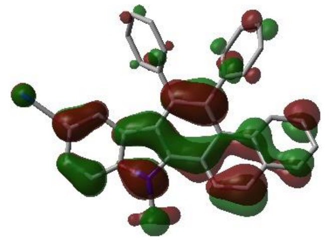	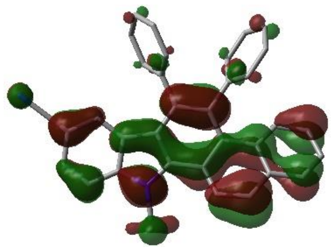

Figure S18. DFT (B3LYP-D3(BJ)/6-311+G**/SMD(ε=MeCN)) optimized geometries and calculated frontier MOs for NC5 in S_0, S_1 and T_1 states. HOMO (Highest Occupied Molecular Orbital); LUMO (Lowest Unoccupied Molecular Orbital); Color coding: N(blue), C(grey).

	**S_0**	**S_1**	**T_1**
**LUMO**	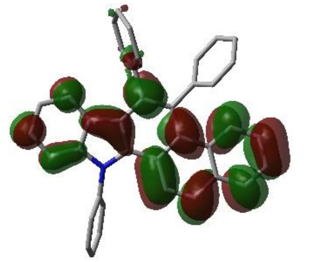	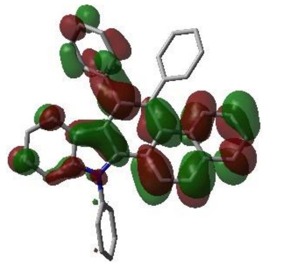	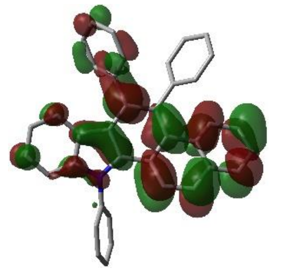
**HOMO**	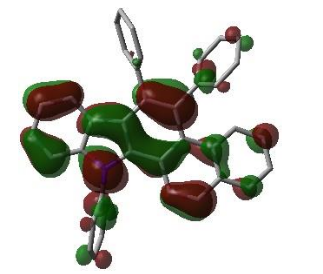	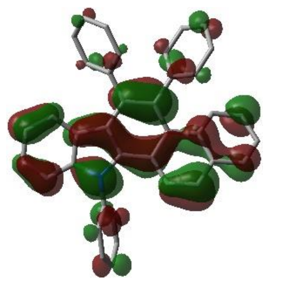	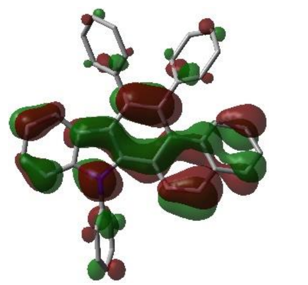

Figure S19. DFT (B3LYP-D3(BJ)/6-311+G**/SMD(ε=MeCN)) optimized geometries and calculated frontier MOs for NC6 in S_0, S_1 and T_1 states. HOMO (Highest Occupied Molecular Orbital); LUMO (Lowest Unoccupied Molecular Orbital); Color coding: N(blue), C(grey).

Table S7. Enthalpy and Gibbs free energies (in Hartree) of all the catalysts at the B3LYP-D3(BJ)/6311+G**/SMD(ε=MeCN) level of theory.

**Structures**	***E***	***G***	**Structures**	***E***	***G***
**NC1 (S_0)**	-1326.177785	-1326.259784	**NC4 (S_0)**	-1785.814685	-1785.900146
**NC1 (S_1)**	-1326.070065	-1326.152005	**NC4 (S_1)**	-1785.706897	-1785.792242
**NC1 (T_1)**	-1326.100888	-1326.183653	**NC4 (T_1)**	-1785.737848	-1785.824097
**NC2 (S_0)**	-1440.708793	-1440.797054	**NC5 (S_0)**	-1418.457123	-1418.543515
**NC2 (S_1)**	-1440.601364	-1440.689304	**NC5 (S_1)**	-1418.347961	-1418.434414
**NC2 (T_1)**	-1440.632087	-1440.721195	**NC5 (T_1)**	-1418.380004	-1418.467238
**NC3 (S_0)**	-1440.707928	-1440.796246	**NC6 (S_0)**	-1517.933781	-1518.024882
**NC3 (S_1)**	-1440.600504	-1440.688342	**NC6 (S_1)**	-1517.824193	-1517.914589
**NC3 (T_1)**	-1440.631177	-1440.719932	**NC6 (T_1)**	-1517.856101	-1517.947133

G = Sum of electronic and thermal free energies.

Table S8: Computational spectral data from TDDFT calculations at B3LYP-D3(BJ)/6311+G**/SMD(ε=MeCN)

**Catalyst**	**Transition**	**ΔE(eV)/λ (nm)**	**f (Oscillator Strength)**	**Transition Character**
**NC1**	$S_0 \rightarrow S_1$ (absorption)	3.30/375.4	0.15	HOMO → LUMO (67%)
**NC2**	$S_0 \rightarrow S_1$ (absorption)	3.30/375.9	0.15	HOMO → LUMO (67%)
**NC3**	$S_0 \rightarrow S_1$ (absorption)	3.30/376.0	0.15	HOMO → LUMO (67%)
**NC4**	$S_0 \rightarrow S_1$ (absorption)	3.30/375.3	0.14	HOMO → LUMO (67%)
**NC5**	$S_0 \rightarrow S_1$ (absorption)	3.33/372.3	0.08	HOMO → LUMO (66%)
**NC6**	$S_0 \rightarrow S_1$ (absorption)	3.37/368.2	0.13	HOMO → LUMO (66%)

Table S9. Cartesian coordinates (Å) of the optimized structures of all the catalysts (NC1-NC6) at B3LYP-D3(BJ)/6311+G**/SMD(ε=MeCN) level of theory.

NC1 (S_0)

N	8.781202000	1.181631000	15.438257000
C	8.411006000	1.660403000	14.188575000
C	8.828352000	1.234709000	12.924158000
H	9.534746000	0.423527000	12.802885000
C	8.290534000	1.876191000	11.813431000
H	8.593821000	1.559614000	10.821820000
C	7.357148000	2.917443000	11.953373000
H	6.950605000	3.392943000	11.068177000
C	6.945987000	3.342182000	13.211724000
H	6.225894000	4.142931000	13.305608000
C	7.475944000	2.717834000	14.350308000
C	7.292681000	2.874722000	15.777909000
C	8.087292000	1.895458000	16.411810000
C	8.062329000	1.725021000	17.820974000
C	8.651934000	0.588781000	18.467399000
H	9.097931000	-0.189624000	17.869881000
C	8.608615000	0.435004000	19.815164000
H	9.020513000	-0.453273000	20.281141000
C	8.073389000	1.462061000	20.648346000
C	8.179343000	1.355483000	22.054528000
H	8.610718000	0.451147000	22.470341000
C	7.776672000	2.383586000	22.878311000
H	7.869496000	2.295914000	23.954811000
C	7.278244000	3.564681000	22.301786000
H	7.008696000	4.403964000	22.933006000
C	7.136594000	3.675830000	20.932318000
C	7.483497000	2.618547000	20.054458000
C	7.333748000	2.683547000	18.598972000
C	6.485692000	3.635336000	17.934725000
C	6.470500000	3.724107000	16.543562000
C	5.562839000	4.673166000	15.833828000
C	6.053446000	5.886211000	15.341986000
H	7.095799000	6.140912000	15.496499000
C	5.214312000	6.764432000	14.659586000
H	5.606523000	7.704071000	14.286769000
C	3.873869000	6.435693000	14.456434000
H	3.219870000	7.118146000	13.925362000
C	3.378951000	5.224826000	14.940490000
H	2.338334000	4.961989000	14.786075000
C	4.219635000	4.348651000	15.625230000
C	5.534522000	5.845146000	18.711666000
C	4.548735000	6.583554000	19.362986000
H	4.618362000	7.665338000	19.391856000
C	3.476568000	5.934958000	19.975833000
H	2.708417000	6.508877000	20.481822000
C	3.396999000	4.542783000	19.929401000
H	2.563593000	4.029942000	20.396743000
C	4.384768000	3.807190000	19.278973000
C	5.467954000	4.448572000	18.667842000
C	9.869449000	0.231804000	15.627311000
H	10.509416000	0.554305000	16.447610000
H	9.498226000	-0.776535000	15.825810000
H	10.472594000	0.207872000	14.721745000
H	4.320225000	2.725553000	19.244422000
H	6.775025000	4.608981000	20.535946000
H	6.368726000	6.353055000	18.243568000
H	3.833463000	3.408916000	16.002839000

NC1 (S_1)

N	8.876976000	1.286110000	15.442320000
C	8.531117000	1.734150000	14.160010000
C	9.012937000	1.300789000	12.934103000
H	9.775006000	0.535754000	12.858740000
C	8.459483000	1.873596000	11.781629000
H	8.811222000	1.554925000	10.807753000
C	7.450995000	2.838177000	11.880195000
H	7.025409000	3.258898000	10.976244000
C	6.977225000	3.273561000	13.120865000
H	6.197191000	4.019105000	13.165413000
C	7.524634000	2.728825000	14.289960000
C	7.281688000	2.885540000	15.712239000
C	8.104222000	1.925610000	16.370757000
C	8.029293000	1.686967000	17.785705000
C	8.498084000	0.536032000	18.420117000
H	8.937994000	-0.266135000	17.847919000
C	8.365520000	0.369787000	19.804693000
H	8.699053000	-0.551973000	20.268365000
C	7.926363000	1.430012000	20.625698000
C	7.981987000	1.342465000	22.044743000
H	8.282559000	0.400905000	22.492001000
C	7.698908000	2.441230000	22.839354000
H	7.751250000	2.358798000	23.919662000
C	7.382380000	3.667041000	22.242300000
H	7.207718000	4.544842000	22.853941000
C	7.284928000	3.766258000	20.849592000
C	7.476504000	2.655677000	20.015289000
C	7.289797000	2.676542000	18.556089000
C	6.414887000	3.563381000	17.889942000

C	6.401912000	3.694968000	16.457084000
C	5.519526000	4.668637000	15.783946000
C	6.073172000	5.704416000	15.011807000
H	7.150550000	5.775501000	14.916995000
C	5.260756000	6.648911000	14.393615000
H	5.711405000	7.447706000	13.814811000
C	3.871486000	6.575965000	14.523894000
H	3.237651000	7.311818000	14.042201000
C	3.307206000	5.547588000	15.281273000
H	2.229878000	5.477041000	15.384321000
C	4.119910000	4.607646000	15.907226000
C	5.379347000	5.746286000	18.608383000
C	4.435566000	6.456091000	19.343334000
H	4.431154000	7.539689000	19.307941000
C	3.494514000	5.777267000	20.119017000
H	2.752865000	6.330819000	20.683704000
C	3.510034000	4.382330000	20.160378000
H	2.776938000	3.847554000	20.753748000
C	4.467911000	3.673411000	19.443053000
C	5.415465000	4.345963000	18.659442000
C	9.968922000	0.351077000	15.679439000
H	10.483032000	0.614911000	16.600799000
H	9.598459000	-0.674697000	15.743798000
H	10.673089000	0.423551000	14.853322000
H	4.484751000	2.590706000	19.480684000
H	7.043923000	4.725568000	20.417462000
H	6.106993000	6.277087000	18.007691000
H	3.671996000	3.813424000	16.491382000

NC1 (T_1)

N	8.910498000	1.287347000	15.422314000
C	8.561239000	1.741069000	14.157860000
C	9.064475000	1.348530000	12.916407000
H	9.843468000	0.601249000	12.832052000
C	8.513239000	1.934042000	11.781158000
H	8.878078000	1.643284000	10.802725000
C	7.482261000	2.883982000	11.884883000
H	7.062011000	3.314206000	10.982905000
C	6.989954000	3.279641000	13.124172000
H	6.194797000	4.009193000	13.180813000
C	7.534029000	2.718197000	14.289051000
C	7.283787000	2.857229000	15.711901000
C	8.122413000	1.929686000	16.357391000
C	8.032344000	1.674779000	17.778176000
C	8.451109000	0.515522000	18.394197000
H	8.892251000	-0.287830000	17.824016000
C	8.279332000	0.325073000	19.811449000
H	8.581450000	-0.612368000	20.263181000
C	7.867991000	1.390637000	20.633174000
C	7.915238000	1.291709000	22.050231000
H	8.163586000	0.333465000	22.493696000
C	7.684471000	2.401596000	22.852836000
H	7.728486000	2.309988000	23.932337000
C	7.428076000	3.638334000	22.260457000
H	7.287459000	4.521955000	22.871842000
C	7.333955000	3.744954000	20.859338000
C	7.478858000	2.637116000	20.030176000
C	7.292614000	2.679755000	18.557143000
C	6.423120000	3.551690000	17.908889000
C	6.385801000	3.653308000	16.462936000
C	5.467311000	4.603312000	15.794035000
C	5.975096000	5.697610000	15.080045000
H	7.048120000	5.838237000	15.018085000
C	5.119211000	6.609878000	14.468139000
H	5.531414000	7.455407000	13.928545000
C	3.736840000	6.439955000	14.552922000
H	3.069322000	7.149669000	14.077372000
C	3.219265000	5.351461000	15.256224000
H	2.146435000	5.208979000	15.324913000
C	4.076366000	4.444296000	15.874057000
C	5.516516000	5.791579000	18.630291000
C	4.593689000	6.561577000	19.333196000
H	4.659972000	7.643391000	19.300132000
C	3.586227000	5.944619000	20.075955000
H	2.864507000	6.543908000	20.619517000
C	3.510275000	4.552128000	20.110301000
H	2.726427000	4.063862000	20.678678000
C	4.439365000	3.783953000	19.412923000
C	5.455666000	4.393993000	18.670068000
C	10.019311000	0.375221000	15.667521000
H	10.510248000	0.635490000	16.603010000
H	9.680143000	-0.662877000	15.709383000
H	10.742823000	0.478328000	14.860360000
H	4.380087000	2.702024000	19.441746000
H	7.127397000	4.714401000	20.431322000
H	6.296946000	6.275457000	18.055733000
H	3.668372000	3.604909000	16.424210000

NC2 (S_0)

N	8.856831000	1.191168000	15.423135000

C	8.501459000	1.665472000	14.167415000
C	8.940116000	1.241052000	12.909736000
H	9.655481000	0.436132000	12.799582000
C	8.410770000	1.874501000	11.790331000
H	8.730316000	1.558456000	10.803661000
C	7.464932000	2.906459000	11.915116000
H	7.065113000	3.375329000	11.023333000
C	7.033197000	3.330440000	13.166784000
H	6.304465000	4.124533000	13.250061000
C	7.554829000	2.714692000	14.313793000
C	7.348284000	2.871291000	15.738249000
C	8.140605000	1.898552000	16.385272000
C	8.090578000	1.724771000	17.793416000
C	8.682223000	0.594936000	18.449177000
H	9.151743000	-0.175315000	17.859221000
C	8.612563000	0.436245000	19.795320000
H	9.027174000	-0.447591000	20.267315000
C	8.044732000	1.452603000	20.620022000
C	8.120421000	1.342360000	22.027871000
H	8.554891000	0.442605000	22.450344000
C	7.684887000	2.361771000	22.845724000
H	7.754506000	2.271457000	23.923751000
C	7.182885000	3.537920000	22.262117000
H	6.887484000	4.370966000	22.890027000
C	7.070473000	3.652020000	20.890201000
C	7.452086000	2.602854000	20.017079000
C	7.333439000	2.671125000	18.559027000
C	6.488300000	3.615010000	17.880664000
C	6.503588000	3.710749000	16.489866000
C	5.601747000	4.656354000	15.767360000
C	6.095724000	5.859385000	15.265335000
H	7.137734000	6.117508000	15.410471000
C	5.240947000	6.724922000	14.580674000
H	5.620590000	7.663360000	14.192199000
C	3.904142000	6.398784000	14.391907000
H	3.231904000	7.065211000	13.864135000
C	3.408138000	5.188780000	14.894090000
C	4.257935000	4.316814000	15.578200000
C	5.477572000	5.805394000	18.638507000
C	4.457188000	6.523595000	19.258491000
H	4.503830000	7.606587000	19.288779000
C	3.379553000	5.853509000	19.837644000
H	2.584235000	6.411820000	20.318496000
C	3.330098000	4.459906000	19.790528000
H	2.493037000	3.930328000	20.231974000
C	4.352198000	3.744696000	19.171465000
C	5.439798000	4.408038000	18.592326000
C	9.949842000	0.250411000	15.629398000
H	10.573359000	0.577338000	16.460473000
H	9.584085000	-0.761353000	15.820506000
H	10.568249000	0.232726000	14.734037000
H	4.309888000	2.662046000	19.133796000
H	6.704445000	4.581151000	20.488369000
H	6.314411000	6.330025000	18.194287000
H	3.891987000	3.381350000	15.976490000
O	2.086318000	4.943742000	14.671848000
C	1.530338000	3.730689000	15.187507000
H	2.011458000	2.854858000	14.742035000
H	1.621915000	3.687214000	16.276735000
H	0.477343000	3.746809000	14.910984000

NC2 (S_1)

N	9.056414000	1.306333000	15.425909000
C	8.738963000	1.731402000	14.129031000
C	9.279298000	1.306733000	12.924319000
H	10.072678000	0.571513000	12.882963000
C	8.743987000	1.847442000	11.748411000
H	9.140717000	1.534413000	10.790115000
C	7.695853000	2.772717000	11.803313000
H	7.285429000	3.168544000	10.881282000
C	7.164245000	3.200918000	13.022749000
H	6.356782000	3.918035000	13.034637000
C	7.691709000	2.687896000	14.215146000
C	7.392757000	2.845129000	15.627021000
C	8.226691000	1.921739000	16.321524000
C	8.106321000	1.684420000	17.733454000
C	8.598579000	0.555778000	18.390158000
H	9.098042000	-0.226031000	17.839384000
C	8.413845000	0.383214000	19.767661000
H	8.767970000	-0.522870000	20.246719000
C	7.890278000	1.421411000	20.567054000
C	7.885353000	1.334477000	21.987114000
H	8.207124000	0.406628000	22.448068000
C	7.518425000	2.418554000	22.767214000
H	7.525533000	2.337269000	23.848845000
C	7.175526000	3.629944000	22.155350000
H	6.935589000	4.498686000	22.757756000
C	7.136547000	3.726286000	20.759673000
C	7.414058000	2.626133000	19.935960000
C	7.291528000	2.640525000	18.469564000

C	6.408642000	3.488802000	17.766228000
C	6.452538000	3.621277000	16.332715000
C	5.568206000	4.565568000	15.622945000
C	6.118831000	5.608570000	14.863575000
H	7.194776000	5.708762000	14.791396000
C	5.285789000	6.529354000	14.232789000
H	5.718790000	7.341720000	13.659521000
C	3.901551000	6.430246000	14.340228000
H	3.245918000	7.146533000	13.859270000
C	3.343287000	5.387478000	15.092626000
C	4.168122000	4.459179000	15.724295000
C	5.237460000	5.619073000	18.440725000
C	4.217790000	6.277607000	19.119695000
H	4.159748000	7.359634000	19.083493000
C	3.267999000	5.549493000	19.837963000
H	2.466398000	6.062862000	20.356749000
C	3.352427000	4.157189000	19.880358000
H	2.613471000	3.583828000	20.428714000
C	4.385456000	3.500212000	19.219908000
C	5.340329000	4.222188000	18.491415000
C	10.175241000	0.416535000	15.707942000
H	10.643857000	0.704475000	16.646060000
H	9.843613000	-0.622960000	15.764297000
H	10.905899000	0.514237000	14.907787000
H	4.453546000	2.419454000	19.255821000
H	6.873896000	4.674505000	20.315992000
H	5.968969000	6.187737000	17.880585000
H	3.745545000	3.651546000	16.302356000
O	1.980553000	5.357085000	15.154491000
C	1.366286000	4.349235000	15.961960000
H	1.591604000	3.347221000	15.584377000
H	1.687286000	4.429703000	17.004763000
H	0.294312000	4.528968000	15.895225000

NC2 (T$_1$)

N	9.071436000	1.306665000	15.405411000
C	8.752459000	1.741454000	14.126690000
C	9.309833000	1.354911000	12.906688000
H	10.115060000	0.632824000	12.856446000
C	8.778139000	1.911851000	11.748084000
H	9.184770000	1.624973000	10.785104000
C	7.713061000	2.827371000	11.808574000
H	7.308331000	3.234654000	10.889011000
C	7.167643000	3.218169000	13.026956000
H	6.348007000	3.921890000	13.050741000
C	7.691672000	2.686833000	14.215050000
C	7.388415000	2.827811000	15.627790000
C	8.231047000	1.929084000	16.307843000
C	8.095360000	1.672899000	17.724548000
C	8.529322000	0.528457000	18.358251000
H	9.019117000	-0.259509000	17.806712000
C	8.308393000	0.331376000	19.766709000
H	8.626181000	-0.594607000	20.231167000
C	7.825369000	1.380893000	20.570383000
C	7.818714000	1.283333000	21.988166000
H	8.084071000	0.334783000	22.442511000
C	7.515584000	2.383765000	22.779179000
H	7.519602000	2.293740000	23.859704000
C	7.238182000	3.610783000	22.175335000
H	7.041320000	4.488509000	22.779668000
C	7.196677000	3.714144000	20.772062000
C	7.415532000	2.612180000	19.950821000
C	7.288791000	2.649519000	18.471588000
C	6.414801000	3.489898000	17.789059000
C	6.437382000	3.597466000	16.341332000
C	5.534624000	4.536332000	15.638267000
C	6.058706000	5.618033000	14.922355000
H	7.131328000	5.758710000	14.870732000
C	5.200501000	6.522278000	14.297072000
H	5.610376000	7.364752000	13.751029000
C	3.822079000	6.363505000	14.374165000
H	3.146791000	7.064981000	13.898379000
C	3.290907000	5.281280000	15.088457000
C	4.142463000	4.370369000	15.712769000
C	5.365787000	5.680413000	18.477624000
C	4.367312000	6.397075000	19.131669000
H	4.375656000	7.480975000	19.100947000
C	3.357115000	5.724515000	19.820464000
H	2.575587000	6.282326000	20.323965000
C	3.356551000	4.329941000	19.852898000
H	2.571736000	3.798268000	20.379437000
C	4.360720000	3.615482000	19.204405000
C	5.377901000	4.281587000	18.512429000
C	10.202248000	0.435154000	15.694551000
H	10.644269000	0.710358000	16.649770000
H	9.900348000	-0.614917000	15.720854000
H	10.953807000	0.567277000	14.917655000
H	4.357429000	2.531805000	19.228103000
H	6.972702000	4.675261000	20.334008000
H	6.145402000	6.207495000	17.941257000

H	3.744533000	3.535481000	16.270256000
O	1.930196000	5.197053000	15.124009000
C	1.340958000	4.155230000	15.907094000
H	1.609567000	3.167868000	15.519601000
H	1.642203000	4.233364000	16.955891000
H	0.264305000	4.296189000	15.824963000

NC3 (S_0)

N	8.852660000	1.156091000	15.453783000
C	8.503623000	1.618469000	14.191881000
C	8.948028000	1.181979000	12.940399000
H	9.661973000	0.374357000	12.841012000
C	8.426827000	1.807353000	11.812559000
H	8.751106000	1.481960000	10.830479000
C	7.483774000	2.843423000	11.923116000
H	7.091026000	3.306348000	11.025094000
C	7.045816000	3.279095000	13.168699000
H	6.318792000	4.075885000	13.240847000
C	7.558170000	2.670887000	14.323929000
C	7.345800000	2.842244000	15.745800000
C	8.134689000	1.875646000	16.405683000
C	8.081607000	1.719300000	17.815586000
C	8.665466000	0.594142000	18.486191000
H	9.128689000	-0.187486000	17.906156000
C	8.595525000	0.454282000	19.834415000
H	9.003594000	-0.426113000	20.318416000
C	8.036657000	1.486714000	20.645265000
C	8.114713000	1.396386000	22.054407000
H	8.543168000	0.499466000	22.488889000
C	7.689316000	2.431014000	22.858397000
H	7.760840000	2.355878000	23.937469000
C	7.195518000	3.602551000	22.258793000
H	6.908535000	4.447157000	22.875067000
C	7.080505000	3.697387000	20.885607000
C	7.451279000	2.632551000	20.026833000
C	7.329772000	2.680430000	18.567970000
C	6.488548000	3.618431000	17.876273000
C	6.500045000	3.693515000	16.483577000
C	5.600266000	4.628273000	15.747290000
C	6.088532000	5.837322000	15.237026000
H	7.128867000	6.100779000	15.390581000
C	5.261341000	6.705986000	14.540171000
H	5.640939000	7.643655000	14.150997000
C	3.915404000	6.378399000	14.331552000
C	3.414151000	5.172277000	14.828274000
H	2.379844000	4.894191000	14.679927000
C	4.260944000	4.312281000	15.530034000
C	5.505397000	5.829897000	18.606082000
C	4.501981000	6.569010000	19.228831000
H	4.562028000	7.651683000	19.243331000
C	3.424161000	5.920028000	19.831605000
H	2.642321000	6.494488000	20.315550000
C	3.356986000	4.526792000	19.803385000
H	2.519577000	4.013424000	20.262981000
C	4.362533000	3.790564000	19.181231000
C	5.451258000	4.432288000	18.580603000
C	9.942452000	0.215017000	15.674725000
H	10.561409000	0.547886000	16.506845000
H	9.573518000	-0.794325000	15.872643000
H	10.566476000	0.188271000	14.783472000
H	4.307250000	2.708036000	19.160575000
H	6.721182000	4.623606000	20.471228000
H	6.343202000	6.338297000	18.144923000
H	3.862193000	3.381040000	15.915659000
O	3.176630000	7.290272000	13.638728000
C	1.794825000	7.002754000	13.408842000
H	1.674004000	6.090529000	12.816938000
H	1.249805000	6.904122000	14.352395000
H	1.404304000	7.852170000	12.850557000

NC3 (S_1)

N	9.001226000	1.251786000	15.468195000
C	8.675038000	1.656408000	14.168807000
C	9.197976000	1.205457000	12.964754000
H	9.982608000	0.460574000	12.927863000
C	8.657252000	1.731790000	11.786071000
H	9.040372000	1.397992000	10.829192000
C	7.619001000	2.669929000	11.835236000
H	7.203492000	3.053824000	10.910395000
C	7.104795000	3.124409000	13.051622000
H	6.303566000	3.848712000	13.057378000
C	7.639012000	2.626911000	14.248249000
C	7.358158000	2.814159000	15.659559000
C	8.188989000	1.897504000	16.361443000
C	8.079893000	1.688219000	17.779273000
C	8.559510000	0.564600000	18.452539000
H	9.040140000	-0.236250000	17.912246000
C	8.385291000	0.423101000	19.836398000
H	8.727775000	-0.480045000	20.329550000
C	7.893114000	1.486853000	20.621413000

C	7.902308000	1.429337000	22.043519000
H	8.206670000	0.503709000	22.520581000
C	7.570108000	2.538631000	22.805040000
H	7.587783000	2.478930000	23.888065000
C	7.248294000	3.744636000	22.172983000
H	7.034673000	4.630806000	22.759727000
C	7.194008000	3.812368000	20.774554000
C	7.435364000	2.689254000	19.971622000
C	7.291299000	2.673420000	18.506774000
C	6.411540000	3.519647000	17.796500000
C	6.432652000	3.614766000	16.360450000
C	5.550464000	4.554594000	15.645992000
C	6.099612000	5.578300000	14.849849000
H	7.176456000	5.660619000	14.760533000
C	5.295218000	6.501452000	14.205180000
H	5.728930000	7.297774000	13.611347000
C	3.898816000	6.423983000	14.322966000
C	3.328540000	5.408368000	15.097575000
H	2.255621000	5.319234000	15.198364000
C	4.152488000	4.495694000	15.750984000
C	5.305265000	5.695217000	18.430025000
C	4.324025000	6.400544000	19.118820000
H	4.293767000	7.482312000	19.052975000
C	3.378546000	5.719147000	19.886685000
H	2.607703000	6.268502000	20.415231000
C	3.427530000	4.326525000	19.967182000
H	2.691558000	3.789542000	20.555001000
C	4.422470000	3.623131000	19.296470000
C	5.374317000	4.297779000	18.519630000
C	10.111784000	0.353642000	15.756695000
H	10.594594000	0.654549000	16.683571000
H	9.769269000	-0.680691000	15.837747000
H	10.835374000	0.425946000	14.947335000
H	4.464680000	2.542597000	19.364331000
H	6.946070000	4.756602000	20.313929000
H	6.036462000	6.227843000	17.835346000
H	3.696026000	3.715950000	16.347554000
O	3.188611000	7.371799000	13.654174000
C	1.761914000	7.347916000	13.760509000
H	1.352773000	6.413975000	13.364326000
H	1.442585000	7.479903000	14.798517000
H	1.406467000	8.184172000	13.160734000

NC3 (T_1)

N	9.022981000	1.245612000	15.450680000
C	8.702949000	1.666061000	14.167686000
C	9.247056000	1.254477000	12.949746000
H	10.040342000	0.518938000	12.904530000
C	8.718551000	1.805064000	11.786616000
H	9.115412000	1.498826000	10.825510000
C	7.669350000	2.739299000	11.840045000
H	7.267300000	3.141648000	10.917142000
C	7.136524000	3.154553000	13.055916000
H	6.327978000	3.871299000	13.073768000
C	7.657261000	2.629265000	14.248207000
C	7.364652000	2.795868000	15.659768000
C	8.198599000	1.896626000	16.348941000
C	8.069020000	1.668192000	17.770755000
C	8.488405000	0.528665000	18.422779000
H	8.961572000	-0.277374000	17.882952000
C	8.274581000	0.363103000	19.837139000
H	8.577847000	-0.559875000	20.317133000
C	7.819865000	1.437184000	20.624329000
C	7.822861000	1.366563000	22.043900000
H	8.071137000	0.421111000	22.514113000
C	7.550122000	2.488198000	22.816388000
H	7.561017000	2.418275000	23.898383000
C	7.294285000	3.709164000	22.191669000
H	7.121212000	4.602314000	22.780496000
C	7.242745000	3.786693000	20.786498000
C	7.430334000	2.665175000	19.984605000
C	7.288790000	2.675242000	18.505942000
C	6.424960000	3.519200000	17.813814000
C	6.429729000	3.593066000	16.365196000
C	5.527116000	4.522886000	15.652112000
C	6.045857000	5.602011000	14.917756000
H	7.119462000	5.740685000	14.863052000
C	5.212308000	6.507311000	14.279770000
H	5.621155000	7.345126000	13.726755000
C	3.821283000	6.353459000	14.350909000
C	3.283052000	5.280206000	15.067231000
H	2.213889000	5.133090000	15.133382000
C	4.136699000	4.384999000	15.710597000
C	5.457377000	5.756736000	18.462220000
C	4.500246000	6.523335000	19.121354000
H	4.546850000	7.605302000	19.066273000
C	3.483138000	5.902759000	19.848029000
H	2.734480000	6.499280000	20.357130000
C	3.432873000	4.510161000	19.910824000
H	2.642312000	4.018778000	20.467120000

C	4.396197000	3.745629000	19.256997000
C	5.421750000	4.359189000	18.530025000
C	10.140172000	0.359147000	15.746958000
H	10.597223000	0.645783000	16.691807000
H	9.819364000	-0.684401000	15.798264000
H	10.886259000	0.460965000	14.960272000
H	4.356311000	2.663601000	19.307522000
H	7.034933000	4.744000000	20.332476000
H	6.244441000	6.243477000	17.899313000
H	3.706056000	3.563650000	16.270469000
O	3.079911000	7.291128000	13.697870000
C	1.654756000	7.194204000	13.770011000
H	1.299139000	6.258965000	13.327627000
H	1.306594000	7.268121000	14.804662000
H	1.271194000	8.036341000	13.196139000

NC4 (S_0)

N	8.784534000	1.180796000	15.438222000
C	8.414626000	1.658856000	14.187996000
C	8.832010000	1.232208000	12.923953000
H	9.538341000	0.420906000	12.803232000
C	8.294297000	1.873063000	11.812838000
H	8.597568000	1.555809000	10.821446000
C	7.361034000	2.914541000	11.952012000
H	6.954581000	3.389444000	11.066467000
C	6.950029000	3.340332000	13.210008000
H	6.230056000	4.141246000	13.303478000
C	7.479905000	2.716551000	14.348890000
C	7.296724000	2.874520000	15.776269000
C	8.090984000	1.895034000	16.411077000
C	8.065441000	1.724472000	17.820408000
C	8.653676000	0.588263000	18.468100000
H	9.100972000	-0.190232000	17.871707000
C	8.607077000	0.434479000	19.815850000
H	9.017410000	-0.454095000	20.282603000
C	8.070837000	1.461708000	20.648193000
C	8.172590000	1.354953000	22.054648000
H	8.601036000	0.449842000	22.471752000
C	7.769596000	2.383923000	22.877234000
H	7.858957000	2.295976000	23.953987000
C	7.276112000	3.566522000	22.299515000
H	7.007648000	4.406726000	22.929964000
C	7.139020000	3.678059000	20.929670000
C	7.484641000	2.619316000	20.053114000
C	7.337009000	2.683233000	18.597726000
C	6.490205000	3.634423000	17.932206000
C	6.474392000	3.724000000	16.541018000
C	5.565062000	4.674036000	15.834900000
C	6.056467000	5.883881000	15.336093000
H	7.102155000	6.132692000	15.477188000
C	5.213228000	6.767240000	14.665449000
H	5.605830000	7.704668000	14.287572000
C	3.868049000	6.446845000	14.481398000
C	3.372553000	5.238695000	14.971684000
H	2.328245000	4.982403000	14.831986000
C	4.217222000	4.357375000	15.644600000
C	5.528146000	5.842535000	18.696807000
C	4.542271000	6.587773000	19.337808000
H	4.597704000	7.668664000	19.361581000
C	3.487267000	5.918283000	19.946256000
C	3.396060000	4.531256000	19.925790000
H	2.563298000	4.025943000	20.398191000
C	4.392572000	3.804869000	19.280730000
C	5.471442000	4.446046000	18.663736000
C	9.871347000	0.229330000	15.628005000
H	10.511638000	0.551439000	16.448175000
H	9.498299000	-0.778208000	15.827008000
H	10.474490000	0.203998000	14.722503000
H	4.328825000	2.723452000	19.257885000
H	6.783183000	4.613192000	20.532836000
H	6.356663000	6.357137000	18.227259000
H	3.830342000	3.420491000	16.028491000
H	3.210707000	7.133725000	13.960276000
Cl	2.227083000	6.853471000	20.760667000

NC4 (S_1)

N	8.881845000	1.287594000	15.442262000
C	8.538794000	1.735897000	14.159664000
C	9.025102000	1.304653000	12.934581000
H	9.789148000	0.541441000	12.860778000
C	8.473700000	1.877182000	11.781287000
H	8.828855000	1.560161000	10.808122000
C	7.462830000	2.839555000	11.877755000
H	7.038954000	3.260146000	10.972964000
C	6.984661000	3.272898000	13.117469000
H	6.202903000	4.016755000	13.160339000
C	7.529911000	2.728452000	14.287442000
C	7.282764000	2.883780000	15.709291000
C	8.105574000	1.925279000	16.369100000
C	8.027936000	1.685164000	17.783952000

C	8.493221000	0.533586000	18.419292000
H	8.934544000	-0.268588000	17.848161000
C	8.354683000	0.366229000	19.803592000
H	8.684722000	-0.556612000	20.267567000
C	7.914426000	1.426267000	20.623753000
C	7.963302000	1.337829000	22.043179000
H	8.257528000	0.394763000	22.491446000
C	7.681571000	2.437422000	22.836724000
H	7.728217000	2.354153000	23.917213000
C	7.374422000	3.665510000	22.239089000
H	7.202282000	4.544022000	22.850388000
C	7.283904000	3.765701000	20.845972000
C	7.471837000	2.654128000	20.012767000
C	7.287380000	2.674746000	18.553034000
C	6.414149000	3.560362000	17.885651000
C	6.399695000	3.691142000	16.452411000
C	5.513667000	4.662532000	15.781401000
C	6.062203000	5.699371000	15.006797000
H	7.138922000	5.771127000	14.905513000
C	5.245609000	6.644539000	14.395326000
H	5.692369000	7.444147000	13.814653000
C	3.857205000	6.571908000	14.535358000
C	3.297852000	5.542573000	15.294983000
H	2.221266000	5.472157000	15.405548000
C	4.114670000	4.601495000	15.913748000
C	5.381098000	5.746529000	18.594122000
C	4.446109000	6.469664000	19.325045000
H	4.434390000	7.551252000	19.285874000
C	3.523701000	5.778230000	20.103378000
C	3.520146000	4.388855000	20.169985000
H	2.788555000	3.866959000	20.773390000
C	4.476240000	3.683064000	19.449328000
C	5.419336000	4.347587000	18.655339000
C	9.974040000	0.353526000	15.682266000
H	10.484472000	0.616998000	16.605768000
H	9.604208000	-0.672619000	15.744332000
H	10.681048000	0.427595000	14.858737000
H	4.487584000	2.601259000	19.498614000
H	7.051738000	4.727037000	20.413404000
H	6.102344000	6.278081000	17.987297000
H	3.670310000	3.806399000	16.499517000
H	3.220287000	7.308812000	14.059397000
Cl	2.323227000	6.687410000	21.024093000

NC4 (T_1)

N	8.913112000	1.286541000	15.422173000
C	8.565249000	1.740309000	14.157292000
C	9.069752000	1.347736000	12.916372000
H	9.848571000	0.600197000	12.832778000
C	8.519980000	1.933659000	11.780663000
H	8.885789000	1.642932000	10.802589000
C	7.489295000	2.884044000	11.883335000
H	7.070329000	3.314602000	10.980930000
C	6.995691000	3.279692000	13.122082000
H	6.200740000	4.009543000	13.177963000
C	7.538236000	2.717697000	14.287368000
C	7.286460000	2.856702000	15.709847000
C	8.124473000	1.929017000	16.356380000
C	8.033334000	1.674067000	17.777100000
C	8.450201000	0.515111000	18.394938000
H	8.891870000	-0.289095000	17.826372000
C	8.274821000	0.325690000	19.812068000
H	8.574713000	-0.611967000	20.264788000
C	7.862560000	1.391849000	20.632540000
C	7.905153000	1.293856000	22.049731000
H	8.149962000	0.335469000	22.494802000
C	7.673903000	2.404942000	22.850771000
H	7.713861000	2.313867000	23.930458000
C	7.422849000	3.641993000	22.256997000
H	7.282844000	4.526460000	22.867282000
C	7.334023000	3.747907000	20.855359000
C	7.477662000	2.638726000	20.027933000
C	7.293349000	2.679343000	18.554974000
C	6.424516000	3.550173000	17.904916000
C	6.387100000	3.652300000	16.459537000
C	5.466547000	4.601726000	15.792795000
C	5.971695000	5.696404000	15.077453000
H	7.044500000	5.835773000	15.009426000
C	5.113303000	6.611222000	14.472939000
H	5.523374000	7.457170000	13.932408000
C	3.731205000	6.443837000	14.566975000
C	3.216217000	5.354801000	15.271291000
H	2.143614000	5.214327000	15.347150000
C	4.075759000	4.444754000	15.881294000
C	5.509038000	5.788422000	18.618253000
C	4.589250000	6.564990000	19.315688000
H	4.642218000	7.645601000	19.280651000
C	3.600663000	5.927292000	20.056801000
C	3.512993000	4.541330000	20.114539000
H	2.731689000	4.060949000	20.689510000

C	4.447061000	3.782363000	19.415931000
C	5.457394000	4.391557000	18.665950000
C	10.020719000	0.373268000	15.668715000
H	10.510831000	0.633126000	16.604750000
H	9.680287000	-0.664417000	15.710237000
H	10.745241000	0.475595000	14.862376000
H	4.387862000	2.701304000	19.454589000
H	7.133559000	4.718207000	20.426299000
H	6.282358000	6.278552000	18.040434000
H	3.669790000	3.605047000	16.432538000
H	3.061866000	7.156025000	14.097745000
Cl	2.422024000	6.903516000	20.941381000

NC5 (S_0)

N	8.775831000	1.186941000	15.426902000
C	8.406885000	1.664759000	14.187695000
C	8.821783000	1.240294000	12.919259000
H	9.525438000	0.428557000	12.792975000
C	8.291173000	1.877653000	11.812346000
H	8.586096000	1.568949000	10.817470000
C	7.355518000	2.927957000	11.963613000
C	6.942285000	3.356740000	13.231134000
H	6.226429000	4.160415000	13.319847000
C	7.472286000	2.726992000	14.354517000
C	7.290583000	2.881538000	15.781706000
C	8.082290000	1.900401000	16.409345000
C	8.062022000	1.726995000	17.817534000
C	8.655570000	0.592064000	18.462524000
H	9.103192000	-0.185405000	17.865518000
C	8.614169000	0.439359000	19.810171000
H	9.028956000	-0.447718000	20.275738000
C	8.078355000	1.465233000	20.644194000
C	8.187059000	1.358228000	22.050035000
H	8.620191000	0.454373000	22.464869000
C	7.784807000	2.385987000	22.874270000
H	7.879626000	2.298475000	23.950574000
C	7.284226000	3.566682000	22.298876000
H	7.015142000	4.405541000	22.930772000
C	7.139752000	3.678105000	20.929861000
C	7.486420000	2.621070000	20.051831000
C	7.334814000	2.685868000	18.596732000
C	6.484190000	3.638030000	17.935350000
C	6.467975000	3.731311000	16.545588000
C	5.561525000	4.678213000	15.832038000
C	6.052528000	5.891381000	15.340840000
H	7.092227000	6.150949000	15.504646000
C	5.216862000	6.762243000	14.644838000
H	5.608963000	7.701521000	14.271189000
C	3.880457000	6.426014000	14.428407000
H	3.229647000	7.102323000	13.885772000
C	3.385303000	5.215614000	14.913281000
H	2.347738000	4.947338000	14.748409000
C	4.222156000	4.346207000	15.611099000
C	5.529984000	5.845221000	18.713449000
C	4.543198000	6.581083000	19.366021000
H	4.610457000	7.662963000	19.395297000
C	3.472927000	5.929765000	19.979221000
H	2.703941000	6.501857000	20.485968000
C	3.395932000	4.537498000	19.932311000
H	2.563847000	4.022867000	20.399940000
C	4.384839000	3.804024000	19.281301000
C	5.465816000	4.448685000	18.670100000
C	9.851358000	0.220033000	15.615043000
H	10.502468000	0.542870000	16.425938000
H	9.461270000	-0.777459000	15.828023000
H	10.445148000	0.176739000	14.704587000
H	4.322910000	2.722274000	19.246574000
H	6.776609000	4.610965000	20.534421000
H	6.362750000	6.354854000	18.244733000
H	3.836685000	3.405865000	15.987833000
C	6.821589000	3.560865000	10.806021000
N	6.388578000	4.075449000	9.863427000

NC5 (S_1)

N	8.878267000	1.302332000	15.433862000
C	8.528827000	1.745750000	14.156904000
C	9.009838000	1.313447000	12.930830000
H	9.772580000	0.550277000	12.852140000
C	8.460213000	1.878649000	11.778034000
H	8.805581000	1.567945000	10.801044000
C	7.440784000	2.844812000	11.887348000
C	6.960840000	3.283532000	13.132647000
H	6.176191000	4.023242000	13.170378000
C	7.517357000	2.741189000	14.292462000
C	7.278397000	2.898414000	15.711209000
C	8.103836000	1.938598000	16.366222000
C	8.028326000	1.698562000	17.778031000
C	8.497050000	0.544681000	18.408796000
H	8.936764000	-0.255199000	17.833100000
C	8.366027000	0.371208000	19.789573000

H	8.700272000	-0.552616000	20.248150000
C	7.924651000	1.427605000	20.617154000
C	7.988079000	1.330730000	22.033051000
H	8.296976000	0.388235000	22.472249000
C	7.701081000	2.422501000	22.835794000
H	7.758583000	2.335446000	23.915192000
C	7.375152000	3.648662000	22.244810000
H	7.198508000	4.522270000	22.861870000
C	7.272304000	3.757367000	20.854576000
C	7.468127000	2.652682000	20.012820000
C	7.281665000	2.681452000	18.554551000
C	6.406960000	3.567305000	17.892846000
C	6.397652000	3.704612000	16.458117000
C	5.521761000	4.682292000	15.786163000
C	6.082638000	5.705190000	15.000734000
H	7.160203000	5.765328000	14.901000000
C	5.276649000	6.650678000	14.376510000
H	5.732258000	7.439037000	13.787540000
C	3.887309000	6.591644000	14.512892000
H	3.258469000	7.327622000	14.025002000
C	3.316109000	5.577135000	15.283857000
H	2.238595000	5.517662000	15.391021000
C	4.121661000	4.636769000	15.917386000
C	5.370493000	5.747749000	18.614248000
C	4.428970000	6.455241000	19.353996000
H	4.424309000	7.538878000	19.321801000
C	3.490254000	5.773918000	20.130284000
H	2.750091000	6.325889000	20.698402000
C	3.505403000	4.378703000	20.167912000
H	2.773297000	3.842641000	20.761244000
C	4.461371000	3.671711000	19.446803000
C	5.406908000	4.347052000	18.662677000
C	9.973385000	0.369465000	15.671131000
H	10.486798000	0.637714000	16.591730000
H	9.605018000	-0.656605000	15.737223000
H	10.677241000	0.443387000	14.844959000
H	4.478501000	2.588944000	19.481088000
H	7.025720000	4.718527000	20.430067000
H	6.096932000	6.279956000	18.013406000
H	3.668414000	3.852305000	16.510238000
C	6.874734000	3.396895000	10.701463000
N	6.416394000	3.844200000	9.737434000

NC5 (T_1)

N	8.904030000	1.295689000	15.410150000
C	8.557321000	1.750360000	14.155319000
C	9.061618000	1.362902000	12.910570000
H	9.840076000	0.616682000	12.820927000
C	8.519519000	1.946634000	11.778898000
H	8.879554000	1.666512000	10.797337000
C	7.482026000	2.902561000	11.893149000
C	6.982246000	3.296925000	13.140678000
H	6.187133000	4.025582000	13.192065000
C	7.527477000	2.730208000	14.291480000
C	7.276517000	2.864499000	15.713138000
C	8.113619000	1.934404000	16.351814000
C	8.026696000	1.677355000	17.772287000
C	8.442841000	0.516338000	18.385072000
H	8.882141000	-0.286729000	17.813109000
C	8.273106000	0.322772000	19.802964000
H	8.574568000	-0.616211000	20.251550000
C	7.867590000	1.388744000	20.626714000
C	7.920643000	1.288664000	22.043321000
H	8.167577000	0.329374000	22.485143000
C	7.697126000	2.398999000	22.847238000
H	7.745558000	2.306885000	23.926429000
C	7.442126000	3.636751000	22.256477000
H	7.306962000	4.520462000	22.868870000
C	7.341835000	3.744610000	20.855943000
C	7.480113000	2.636664000	20.026384000
C	7.288806000	2.682066000	18.554092000
C	6.418126000	3.555809000	17.911036000
C	6.379456000	3.660831000	16.465322000
C	5.461513000	4.609824000	15.794930000
C	5.970453000	5.703165000	15.080450000
H	7.043422000	5.846369000	15.022691000
C	5.114699000	6.610518000	14.461087000
H	5.526796000	7.455020000	13.919979000
C	3.732667000	6.436187000	14.539899000
H	3.065346000	7.141663000	14.057900000
C	3.214615000	5.349175000	15.245061000
H	2.141916000	5.204013000	15.309002000
C	4.071035000	4.446667000	15.870433000
C	5.510535000	5.794095000	18.635008000
C	4.588602000	6.562369000	19.340976000
H	4.652912000	7.644273000	19.307868000
C	3.584642000	5.943509000	20.086800000
H	2.863650000	6.541521000	20.632701000
C	3.510984000	4.550897000	20.121309000
H	2.729736000	4.061338000	20.692097000

C	4.439017000	3.784285000	19.420902000
C	5.451847000	4.396488000	18.675119000
C	10.007416000	0.374623000	15.656422000
H	10.504238000	0.640397000	16.587083000
H	9.656255000	-0.658370000	15.709062000
H	10.727070000	0.464930000	14.844839000
H	4.381654000	2.702267000	19.449745000
H	7.136065000	4.714790000	20.429138000
H	6.288435000	6.279349000	18.058197000
H	3.663268000	3.607934000	16.421693000
C	6.930800000	3.476928000	10.712798000
N	6.484782000	3.943050000	9.751314000

NC6 (S_0)

N	8.607260000	0.992443000	15.492843000
C	8.264322000	1.475576000	14.230075000
C	8.684563000	1.022322000	12.979295000
H	9.371297000	0.190818000	12.884820000
C	8.180726000	1.671710000	11.857018000
H	8.483805000	1.337958000	10.871084000
C	7.285166000	2.747799000	11.979778000
H	6.903484000	3.228465000	11.086365000
C	6.885185000	3.207481000	13.229797000
H	6.201712000	4.041328000	13.308584000
C	7.384497000	2.577964000	14.379490000
C	7.221037000	2.775279000	15.807163000
C	7.972934000	1.779009000	16.458314000
C	7.959652000	1.635613000	17.867955000
C	8.530149000	0.494983000	18.522196000
H	8.955918000	-0.297130000	17.926314000
C	8.499749000	0.366481000	19.872258000
H	8.896547000	-0.522352000	20.350181000
C	7.999623000	1.421341000	20.693665000
C	8.118605000	1.332269000	22.099829000
H	8.530682000	0.422787000	22.523804000
C	7.752331000	2.382165000	22.913147000
H	7.854826000	2.307283000	23.989710000
C	7.278324000	3.567281000	22.325024000
H	7.036631000	4.421875000	22.946896000
C	7.125233000	3.663306000	20.955547000
C	7.435090000	2.585468000	20.089034000
C	7.274604000	2.633250000	18.633794000
C	6.460895000	3.606080000	17.955599000
C	6.439569000	3.672058000	16.563084000
C	5.569172000	4.647191000	15.842262000
C	6.111583000	5.824361000	15.318637000
H	7.166797000	6.031574000	15.455956000
C	5.307271000	6.727729000	14.627186000
H	5.739666000	7.639177000	14.229667000
C	3.950097000	6.460180000	14.446816000
H	3.323157000	7.162332000	13.908835000
C	3.403557000	5.284927000	14.962111000
H	2.349697000	5.069491000	14.825115000
C	4.209422000	4.383577000	15.655942000
C	5.621486000	5.870121000	18.702116000
C	4.676336000	6.665048000	19.347133000
H	4.798723000	7.742421000	19.359712000
C	3.577275000	6.078341000	19.974531000
H	2.840735000	6.696364000	20.475553000
C	3.429720000	4.691180000	19.949245000
H	2.574933000	4.226394000	20.428054000
C	4.376895000	3.898850000	19.305134000
C	5.486433000	4.478091000	18.679717000
H	4.259924000	2.821226000	19.287111000
H	6.783666000	4.600054000	20.550347000
H	6.476788000	6.329855000	18.222351000
H	3.783035000	3.471956000	16.058290000
C	9.679499000	0.066880000	15.687579000
C	9.492779000	-1.278537000	15.378437000
C	10.907835000	0.524523000	16.163133000
C	10.543455000	-2.176555000	15.558574000
C	11.949960000	-0.379402000	16.352437000
C	11.769607000	-1.729975000	16.050654000
H	8.529506000	-1.613738000	15.013435000
H	11.033732000	1.575962000	16.392251000
H	10.400376000	-3.224618000	15.322408000
H	12.903303000	-0.028443000	16.730143000
H	12.582821000	-2.431457000	16.197031000

NC6 (S_1)

N	8.685813000	1.072848000	15.452758000
C	8.339588000	1.513014000	14.162628000
C	8.798400000	1.049293000	12.939342000
H	9.534972000	0.259630000	12.873197000
C	8.260799000	1.634241000	11.787486000
H	8.590015000	1.291632000	10.813863000
C	7.300018000	2.646599000	11.884224000
H	6.885566000	3.076685000	10.979652000
C	6.867298000	3.124198000	13.124052000
H	6.135019000	3.916642000	13.168016000

C	7.400410000	2.567464000	14.293044000
C	7.204562000	2.779462000	15.720424000
C	7.988627000	1.799437000	16.385243000
C	7.926844000	1.582218000	17.806026000
C	8.357842000	0.421213000	18.445784000
H	8.780512000	-0.391970000	17.875635000
C	8.219622000	0.270182000	19.833820000
H	8.517840000	-0.660116000	20.304409000
C	7.832630000	1.355136000	20.645483000
C	7.893002000	1.280166000	22.066782000
H	8.145406000	0.328524000	22.522466000
C	7.678338000	2.400283000	22.849942000
H	7.735436000	2.326296000	23.930696000
C	7.425732000	3.637292000	22.242687000
H	7.307903000	4.530075000	22.845938000
C	7.318856000	3.724971000	20.850007000
C	7.441199000	2.597852000	20.028034000
C	7.240943000	2.613779000	18.567239000
C	6.405735000	3.532439000	17.900377000
C	6.374925000	3.640815000	16.460739000
C	5.524257000	4.639705000	15.786564000
C	6.106690000	5.628027000	14.973522000
H	7.183276000	5.643989000	14.849462000
C	5.324961000	6.596161000	14.352564000
H	5.798187000	7.357087000	13.741666000
C	3.937969000	6.595560000	14.520879000
H	3.328114000	7.349791000	14.036708000
C	3.344767000	5.616275000	15.320354000
H	2.268588000	5.602349000	15.453670000
C	4.126952000	4.653466000	15.949698000
C	5.513925000	5.785961000	18.590165000
C	4.625490000	6.565524000	19.323443000
H	4.686159000	7.646505000	19.266872000
C	3.656195000	5.959719000	20.124279000
H	2.957456000	6.567736000	20.687641000
C	3.587108000	4.567270000	20.191834000
H	2.830735000	4.089320000	20.804147000
C	4.489973000	3.788116000	19.476354000
C	5.466398000	4.387084000	18.668711000
H	4.440980000	2.707289000	19.533836000
H	7.124664000	4.690887000	20.408787000
H	6.263474000	6.260307000	17.969361000
H	3.656530000	3.897669000	16.566083000
C	9.747715000	0.152331000	15.699915000
C	9.649150000	-1.157908000	15.235383000
C	10.871146000	0.584642000	16.405667000
C	10.688252000	-2.049640000	15.490371000
C	11.901901000	-0.315227000	16.658412000
C	11.811872000	-1.631766000	16.204191000
H	8.761618000	-1.474379000	14.701980000
H	10.927297000	1.610205000	16.748207000
H	10.614640000	-3.071845000	15.138554000
H	12.776927000	0.013144000	17.206853000
H	12.616437000	-2.329665000	16.404440000

NC6 (T_1)

N	8.686105000	1.043859000	15.435669000
C	8.343563000	1.499105000	14.161235000
C	8.815219000	1.067618000	12.922675000
H	9.557951000	0.284178000	12.845616000
C	8.284664000	1.670791000	11.786626000
H	8.623880000	1.351528000	10.807916000
C	7.311701000	2.679230000	11.890774000
H	6.904918000	3.121937000	10.988742000
C	6.865257000	3.123633000	13.130951000
H	6.122500000	3.906205000	13.188940000
C	7.393028000	2.546615000	14.295625000
C	7.191681000	2.741886000	15.722503000
C	7.983019000	1.783054000	16.375380000
C	7.907626000	1.558075000	17.801207000
C	8.291560000	0.392633000	18.426962000
H	8.703333000	-0.428588000	17.859443000
C	8.131397000	0.228844000	19.849353000
H	8.402067000	-0.713178000	20.311223000
C	7.777879000	1.323014000	20.659044000
C	7.842868000	1.242860000	22.077138000
H	8.054741000	0.280731000	22.530961000
C	7.677467000	2.373235000	22.865989000
H	7.735840000	2.295480000	23.945889000
C	7.468605000	3.612394000	22.258894000
H	7.380943000	4.510620000	22.858693000
C	7.354958000	3.702663000	20.858518000
C	7.435318000	2.577751000	20.043463000
C	7.230397000	2.609710000	18.572162000
C	6.404445000	3.520484000	17.920689000
C	6.353567000	3.601375000	16.471297000
C	5.480963000	4.590281000	15.798152000
C	6.040988000	5.638406000	15.054679000
H	7.119283000	5.713603000	14.973497000
C	5.230246000	6.587324000	14.437059000

H	5.682499000	7.396289000	13.874091000
C	3.841952000	6.500609000	14.546011000
H	3.209618000	7.238964000	14.065993000
C	3.272626000	5.458182000	15.278598000
H	2.194541000	5.380582000	15.365937000
C	4.084714000	4.514695000	15.902559000
C	5.636999000	5.820252000	18.613396000
C	4.768986000	6.653483000	19.314058000
H	4.896864000	7.728977000	19.261778000
C	3.737547000	6.107688000	20.079204000
H	3.058638000	6.756395000	20.621171000
C	3.582241000	4.722589000	20.138238000
H	2.779192000	4.289660000	20.724362000
C	4.456533000	3.890860000	19.443027000
C	5.496619000	4.429325000	18.678063000
H	4.335756000	2.814757000	19.490943000
H	7.186485000	4.674545000	20.419650000
H	6.436356000	6.248934000	18.021265000
H	3.637104000	3.711651000	16.475544000
C	9.766520000	0.146334000	15.687939000
C	9.690730000	-1.170374000	15.237275000
C	10.887438000	0.597932000	16.384506000
C	10.746850000	-2.043281000	15.490566000
C	11.933099000	-0.283620000	16.644718000
C	11.865590000	-1.604058000	16.198455000
H	8.807020000	-1.505966000	14.708702000
H	10.929043000	1.626302000	16.721822000
H	10.689255000	-3.068453000	15.143786000
H	12.802711000	0.062756000	17.191062000
H	12.682155000	-2.287381000	16.400882000

References:

1. Melhuish, W. H. Quantum efficiencies of Fluorescence of Organic Substances: Effect of Solvent and Concentration of the Florescent solute. *J. Phys. Chem.* **1961**, *65*, 229–235.

[2] Lakowicz, J. R. Principles of Fluorescence Spectroscopy; Plenum: New York, **1999**; 3rd edition.

[3] (a) Roth, H. G.; Romero, N. A.; Nicewicz, D. A. Experimental and Calculated Electrochemical Potentials of Common Organic Molecules for Applications to Single-Electron Redox Chemistry. *Synlett* **2016**, *27*, 714–723. (b) Connelly, N. G.; Geiger, W. E. Chemical Redox Agents for Organometallic Chemistry. *Chem. Rev.* **1996**, *96*, 877-910.

[4] Pavlishchuk, V. V.; Addision, A. W. Conversion Constants for Redox Potentials Measured versus Different Reference Electrodes in Acetonitrile Solutions at 25°C *Inorganic Chemica Acta* **2000**, *298*, 97–102.

[5] Buzzetti, L.; Crisenza, G. E. M.; Melchiorre, P. Mechanistic Studies in Photocatalysis. Angew. Chem., Int. Ed. **2019**, *58*, 3730–3747.

[6] (a) Kundu, S.; Banerjee, A.; Maji, M. S. Brønsted Acid-Catalyzed Tandem Pinacol-Type Rearrangement for the Synthesis of *α*-(3-Indolyl) Ketones by Using *α*-Hydroxy Aldehydes. *J. Org. Chem.* **2019**, *84*, 16003–16012. (b) Kundu, S.; Banerjee, A.; Pal, S. C.; Ghosh, M.; Maji, M. S. Cascade Annulative π-Extension for the Rapid Construction of Carbazole Based Polyaromatic Hydrocarbons. *Chem. Commun.* **2021**, *57*, 5762-5765.

[7] (a) Sun, K.; Xie, X.; Liu, Y.; Jiang, W.; Ban, X.; Huang, B.; Sun, Y. Thermally cross-linkable thermally activated delayed fluorescent materials for efficient blue solution-processed organic light-emitting diodes. *J. Mater. Chem. C,* **2016**, *4*, 8973–8979. (b) Yu, W.; Gu, S.; Fu, Y.; Xiong, S.; Pan, C.; Liu, Y.; Yu, G. Carbazole-decorated Covalent Triazine Frameworks: Novel Nonmetal Catalysts for Carbon dioxide Fixation and Oxygen Reduction Reaction. *J. Catal*. **2018**, *362*, 1–9.

[8] Jiang, w.; Duan, L.; Qiao, J.; Dong, G.; Zhang, D.; Wang, L.; Qiu, Y. High-triplet-energy Tri-carbazole Derivatives as Host Materials for Efficient Solution-processed Blue Phosphorescent Devices. *J. Mater. Chem*. **2011**, *21*, 4918–4926.

[9] (a) Wagner, D.; Hoffmann, S. T.; Heinemeyer, U.; Münster, I.; Köhler, A.; Strohriegl, P.; Triazine Based Bipolar Host Materials for Blue Phosphorescent OLEDs. *Chem. Mater*. **2013**, *25*, 3758−3765. (b) Wan, J.-L.; Cui, J.-F.; Zhong, W.-Q.; Huang, J. -M. Iminyl-Radicals by Electrochemical Decarboxylation of α-Imino-oxy acids:C of Indole-Fused Polycyclics. *Chem. Commun.* **2021**, *57*, 10242–10245. (c) Bernard, R.; Barsu, C.; Baldeck, P. L.; Andraud, C.; Cornu, D.; Scharff, J.-P.; Miele, P. Synthesis, and Two Photon Absorption Properties of 7,7′-(iminundecahydro-*closo*-dodecaborate)-9,9′-(dihexyl)-2,2′-bifluorene. *Chem. Commun.* **2008**, 3765–3767.

[10] (a) Yang, P.; Zheng, C.; Nie, Y.-H.; You, S.-L. Palladium-Catalyzed Dearomative 1,4-Difunctionalization of Naphthalenes. *Chem. Sci*. **2020**, *11*, 6830–6835. (c) Abel-Snape, X.; Whyte, A.; Lautens, M. Synthesis of Aminated Phenanthridinones via Palladium/Norbornene Catalysis. *Org. Lett.* **2020**, *22*, 7920–7925. (b) Barraza, S. J.; Denmark, S. E. Unexpected

Rearrangement of 2-Bromoaniline under Biphasic Alkylation Conditions. *Synlett* **2017**, *28*, 2891–2895.

[11] Kundu, S.; Roy, L.; Maji, M. S. Development of Carbazole-Cored Organo-Photocatalyst for Visible Light-Driven Reductive Pinacol/Imino-Pinacol Coupling. *Org. Lett.* **2022**, *24*, 9001–9006.

[12] Kundu, S.; Maji, M. S. Solution-Phase Late-Stage Chemoselective Photocatalytic Removal of Sulfonyl and Phenacyl Groups in Peptides. *Chem.–Eur. J.* **2024**, 30, e2024000.

[13] (a) Zhang L.; Jiao, L. Visible-Light-Induced Organocatalytic Borylation of Aryl Chlorides. *J. Am. Chem. Soc*. **2019**, *141*, 9124−9128. (b) Tsukamoto, H.; Kondo, Y. Palladium (II)-Catalyzed Annulation ofAlkynes withortho-Ester-ContainingPhenylboronic Acids. *Org. Lett.* **2007**, *9*, 4227–4230. (c) Diev, V. V.; Hanson, K.; Zimmerman, J. D.; Forrest, S. R.; Thompson, M. E. Fused Pyrene–Diporphyrins: Shifting Near-Infrared Absorption to 1.5 mm and Beyond. *Angew. Chem., Int. Ed*. **2010**, *49*, 5523–5526. (d) Zhao, J.-H.; Zhou, Z.-Z.; Zhang, Y.; Su, X.; Chen, X.-M.; Liang, Y.-M. Visible-light-mediated Borylation of Aryl and Alkyl Halides with a Palladium Complex. *Org. Biomol. Chem.* **2020**, *18*, 4390–4394. (g) Han, F. S.; Higuchi, M.; Kurth, D. G. Diverse Synthesis of Novel Bisterpyridines via Suzuki-Type Cross-Coupling. *Org. Lett.* **2007**, *9*, 559–562. (e) Wang, X.; Liu, W.-G.; Tung, C.-H.; Wu, L.-Z.; Cong, H. A Monophosphine Ligand Derived from Anthracene Photodimer: Synthetic Applications for Palladium-Catalyzed Coupling Reactions. *Org. Lett*. **2019**, *21*, 8158−8163 (f) Chua, M.-H.; Zhou, H.; Lin, T. T.;Wu, J.; Xu, J. W. Aggregation-Induced Emission Active 3,6-Bis(1,2,2-Triphenylvinyl) Carbazole and Bis(4-(1,2,2-Triphenylvinyl)Phenyl)Amine-Based Poly(Acrylates) for Explosive Detection. *J. Polym. Sci., Part A: Polym Chem*. **2017**, *55*, 672–681. (h) Taura, D.; Shimizu, K.; Yokota, C.; Ikeda, R.; Suzuki, Y.; Iida, H.; Ousaka, N.; Yashima, E. Fluorescent Molecular Spring that Visualizes the Extension and Contraction Motions of a Double-Stranded Helicate Bearing Terminal Pyrene Units Triggered by Release and Binding of Alkali Metal Ions. *Chem. Commun.* **2019**, *55*, 12084–12087. (i) Guo, X.; Zhang, Y.; Hu, Y.; Yang, J.; Li, Y.; Ni, Z.; Dong, H.; Hu, W. Molecular Weight Engineering in High-Performance Ambipolar Emissive Mesopolymers. *Angew. Chem., Int. Ed*. **2021**, *60*, 14902–14908.

[14] Zeng, H.; Dou, Q.; Li, C.-J. Photoinduced Transition-Metal-Free Cross-Coupling of Aryl Halides with H-Phosphonates. *Org. Lett*. **2019**, *21*, 1301−1305.

[15] (a) Manikandan, T. S.; Ramesh, R.; Semeril, D. The Tandem C−H/N−H Activation of *N*-Methyl Arylamide Catalyzed by Dinuclear Pd(II) Benzhydrazone Complex: A Concise Access to Phenanthridinone. *Organometallics* **2019**, *38*, 319−328. (b) Chen, Z.; Wang, X. A Pd-Catalyzed, Boron Ester-mediated, Reductive Cross-Coupling of Two Aryl Halides to Synthesize Tricyclic Biaryls. *Org. Biomol. Chem.* **2017**, *15*, 5790–5796. (c) Sharma, S.; Kumar, M.; Sharma, S.; Nayal, O. S.; Kumar, N.; Singh, B.; Sharma, U. Microwave Assisted Synthesis of Phenanthridinones and Dihydrophenanthridines by Vasicine/KO^{t}Bu Promoted Intramolecular C–H Arylation. *Org. Biomol. Chem*. **2016**, *14*, 8536–8544. (d) Conde, N.; Churruca, F.; SanMartin, R.; Herrero, M. T.; Dominguez, E. A Further Decrease in the Catalyst Loading for the Palladium-Catalyzed Direct Intramolecular Arylation of Amides and Sulfonamides. *Adv. Synth. Catal*. **2015**, *357*, 1525–1531. (e) Chi, Z.; Gao, Y.; Yang, L.; Zhou,

C.; Zhang, M.; Cheng, P.; Li, G. Dearomative Spirocyclization *via* Visible-Light-Induced Reductive Hydroarylation of Non-activated Arenes. *Chinese Chemical Letters* **2022**, *33*, 225–228.

[16] Frisch, M. J.; Trucks, G. W.; Schlegel, H. B.; Scuseria, G. E.; Robb, M. A.; Cheeseman, J. R.; Scalmani, G.; Barone, V.; Petersson, G. A.; Nakatsuji, H.; Li, X.; Caricato, M.; Marenich, A. V.; Bloino, J.; Janesko, B. G.; Gomperts, R.; Mennucci, B.; Hratchian, H. P.; Ortiz, J. V.; Izmaylov, A. F.; Sonnenberg, J. L.; Williams-Young, D.; Ding, F.; Lipparini, F.; Egidi, F.; Goings, J.; Peng, B.; Petrone, A.; Henderson, T.; Ranasinghe, D.; Zakrzewski, V. G.; Gao, J.; Rega, N.; Zheng, G.; Liang, W.; Hada, M.; Ehara, M.; Toyota, K.; Fukuda, R.; Hasegawa, J.; Ishida, M.; Nakajima, T.; Honda, Y.; Kitao, O.; Nakai, H.; Vreven, T.; Throssell, K.; Montgomery, J. A., Jr.; Peralta, J. E.; Ogliaro, F.; Bearpark, M. J.; Heyd, J. J.; Brothers, E. N.; Kudin, K. N.; Staroverov, V. N.; Keith, T. A.; Kobayashi, R.; Normand, J.; Raghavachari, K.; Rendell, A. P.; Burant, J. C.; Iyengar, S. S.; Tomasi, J.; Cossi, M.; Millam, J. M.; Klene, M.; Adamo, C.; Cammi, R.; Ochterski, J. W.; Martin, R. L.; Morokuma, K.; Farkas, O.; Foresman, J. B.; Fox, D. J. *Gaussian 16* (Revision B.01): Gaussian, Inc.: Wallingford CT (**2016**).

[17] Hehre, W. J.; Ditchfield, R.; Pople, J. A. Self–Consistent Molecular Orbital Methods. XII. Further Extensions of Gaussian–Type Basis Sets for Use in Molecular Orbital Studies of Organic Molecules. *J. Chem. Phys.* **1972**, *56*, 2257–2261.

[18] Marenich, A. V.; Cramer, C. J.; Truhlar, D. G. Universal Solvation Model Based on Solute Electron Density and on a Continuum Model of the Solvent Defined by the Bulk Dielectric Constant and Atomic Surface Tensions. *J. Phys. Chem. B* **2009**, *113*, 6378.

^{1}H, ^{13}C, ^{11}B, ^{31}P and ^{19}F NMR Spectra:

^{1}H NMR (400 MHz) of **NC1** in $CDCl_3$

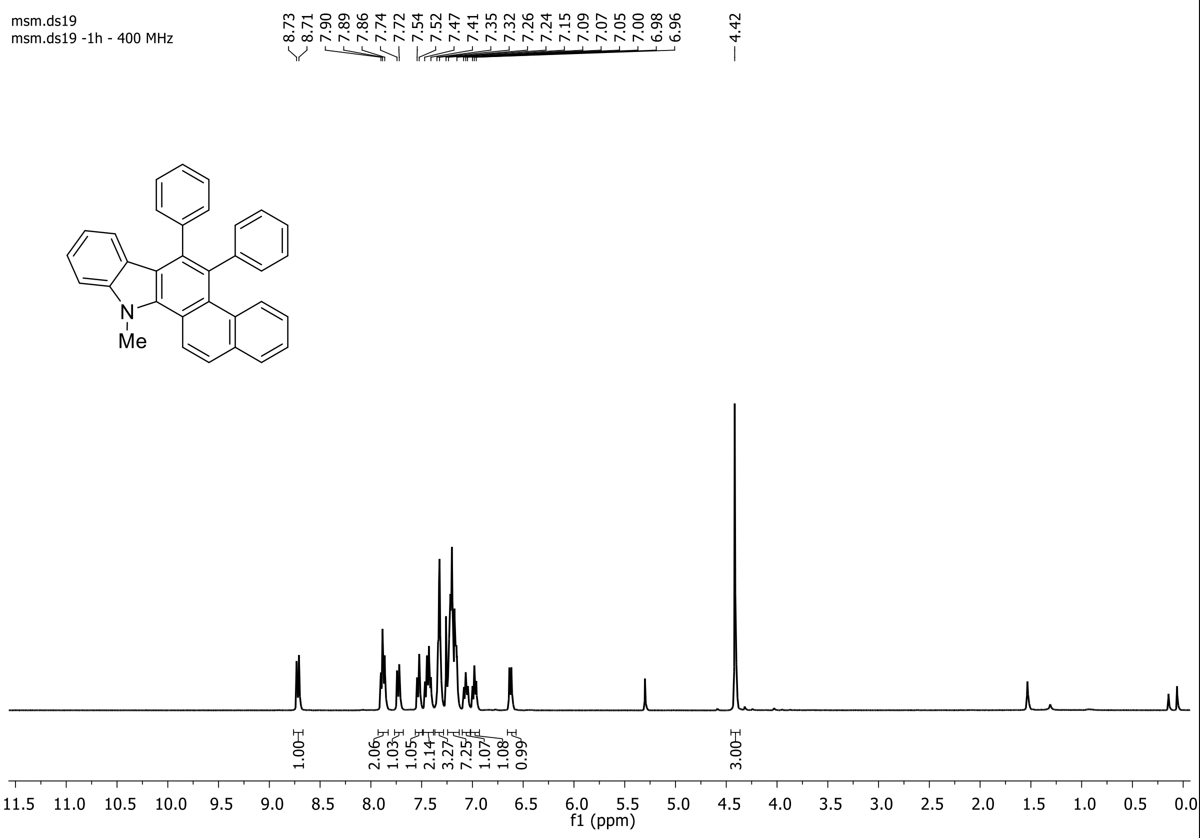

^{1}H (500 MHz) and ^{13}C (126 MHz) NMR of **NC2** in $CDCl_3$

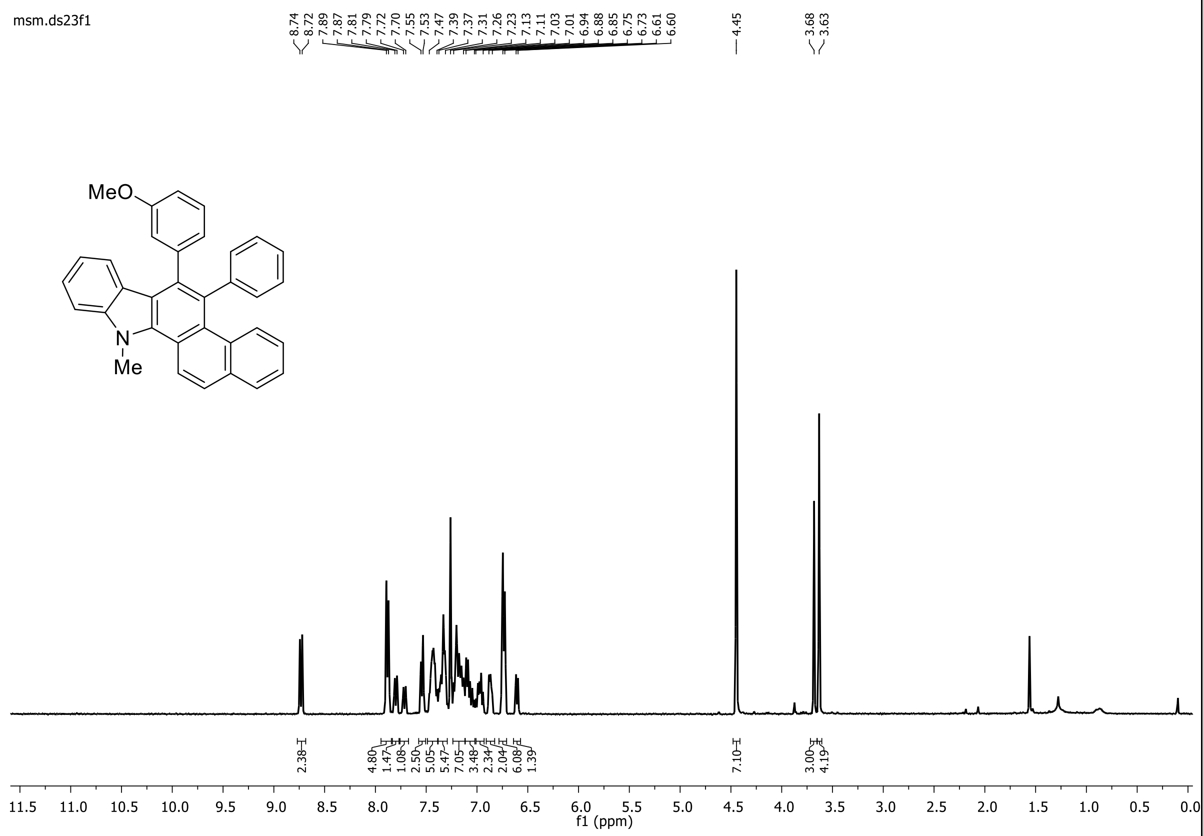

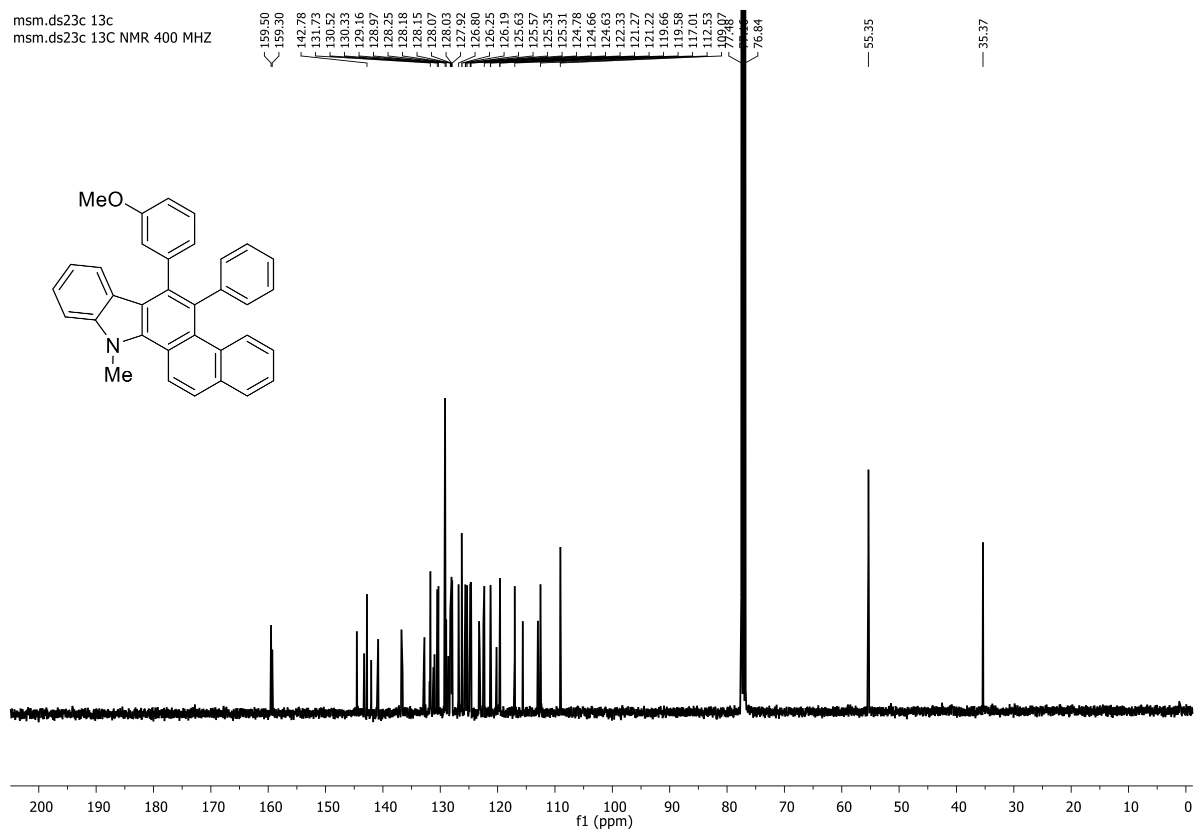

^{1}H (400 MHz) NMR of **NC3** in $CDCl_3$

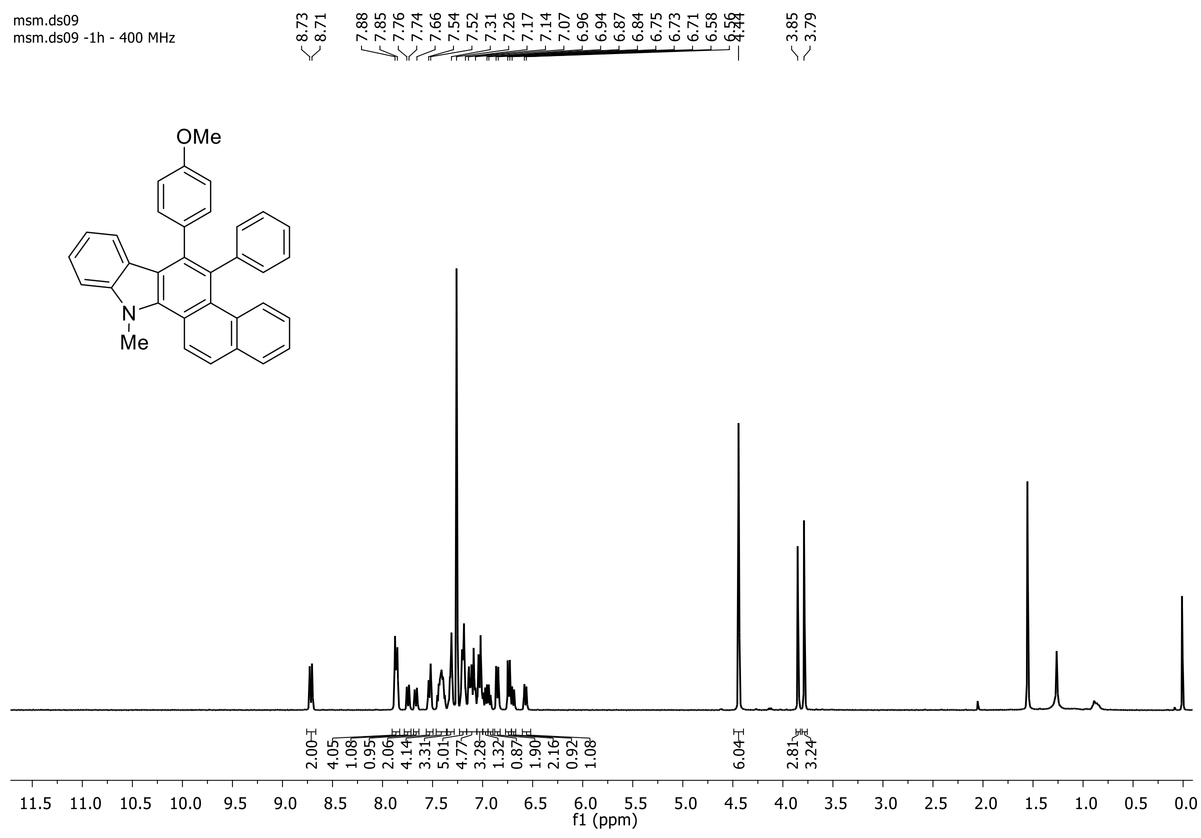

^{1}H (500 MHz) and ^{13}C (126 MHz) NMR of **NC4** in $CDCl_3$

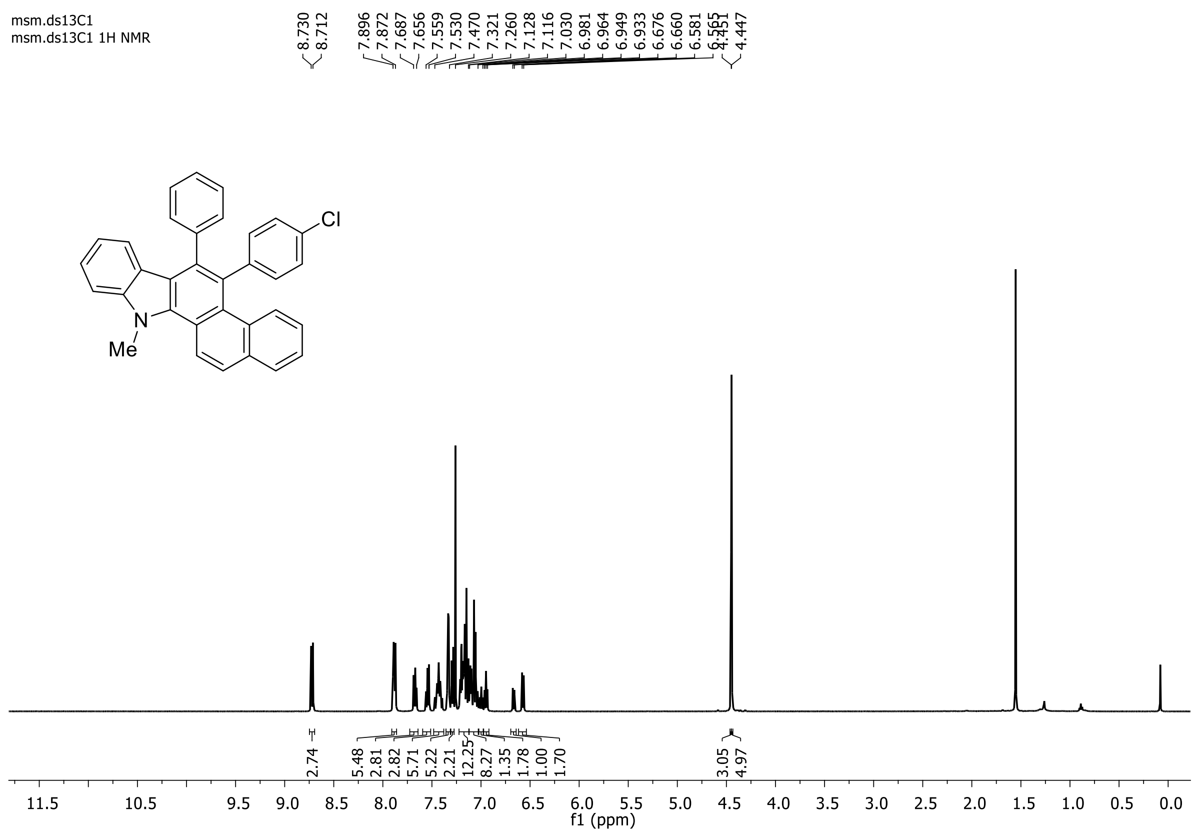

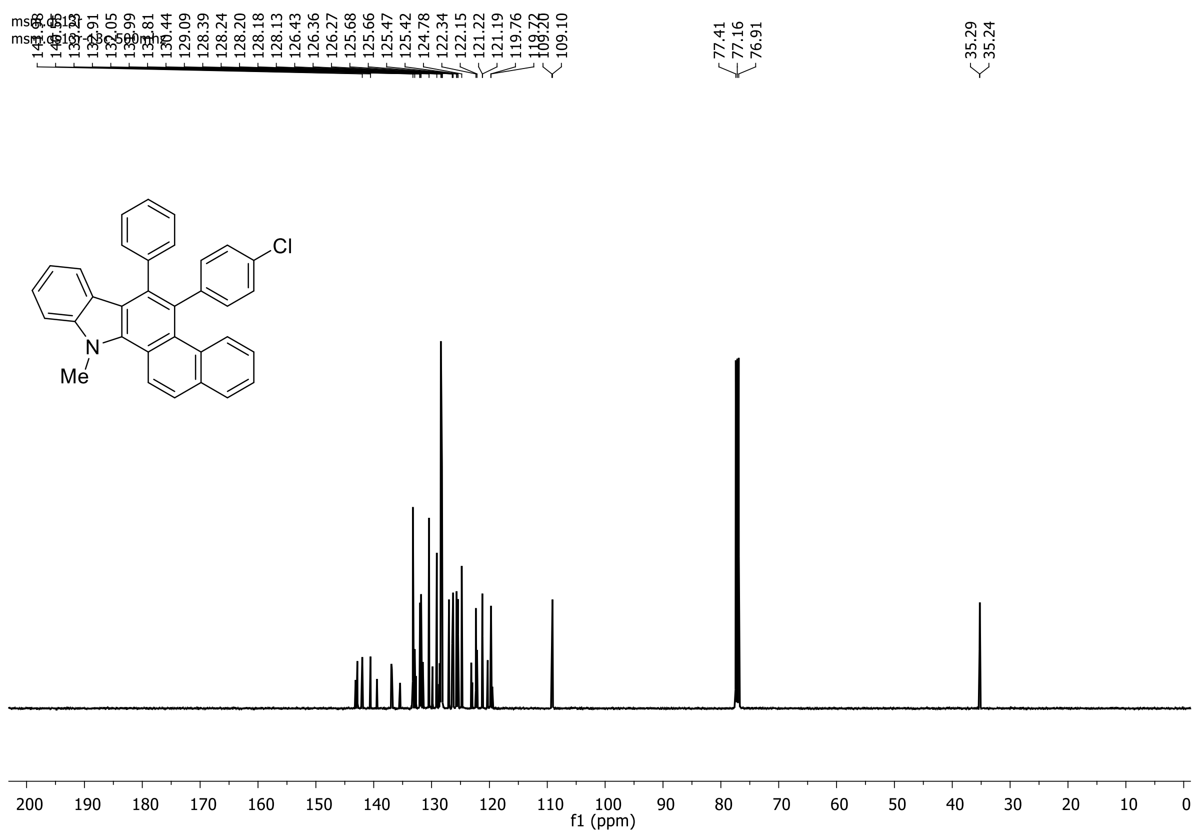

^{1}H (500 MHz) and ^{13}C (126 MHz) NMR of **NC5** in $CDCl_3$

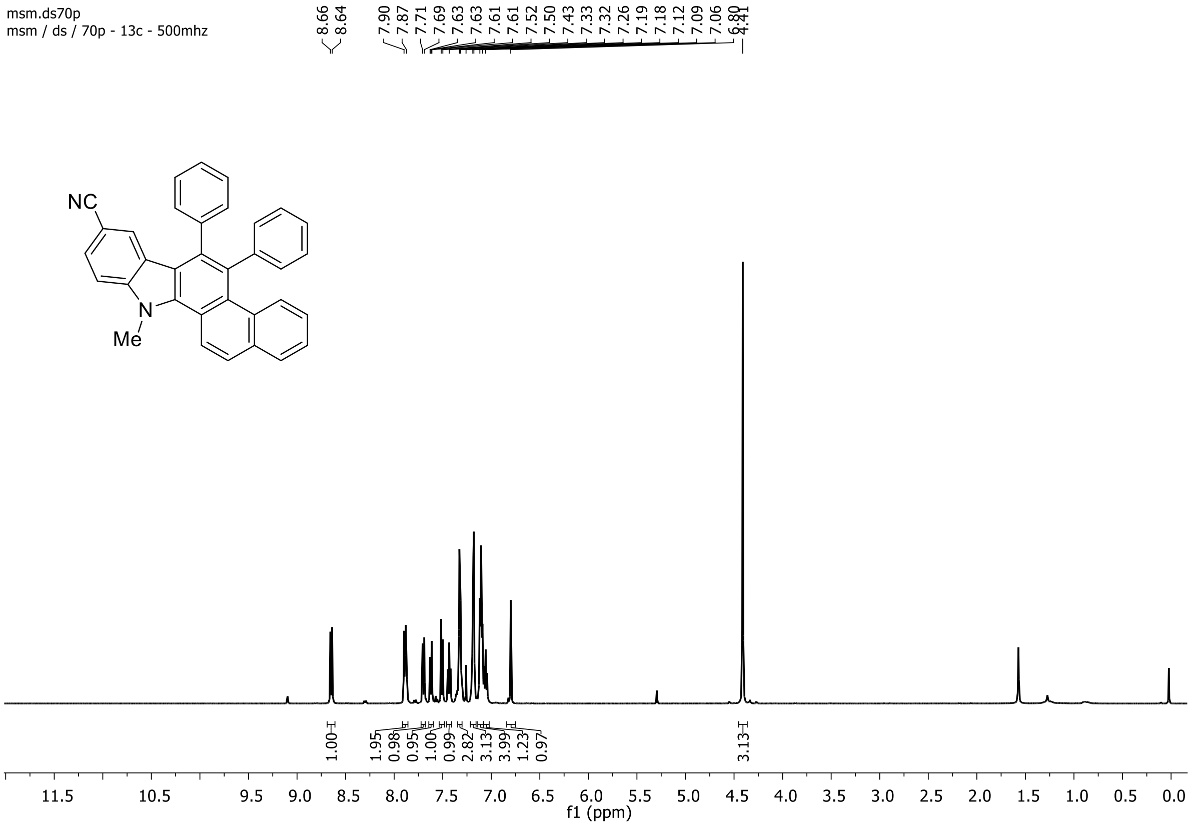

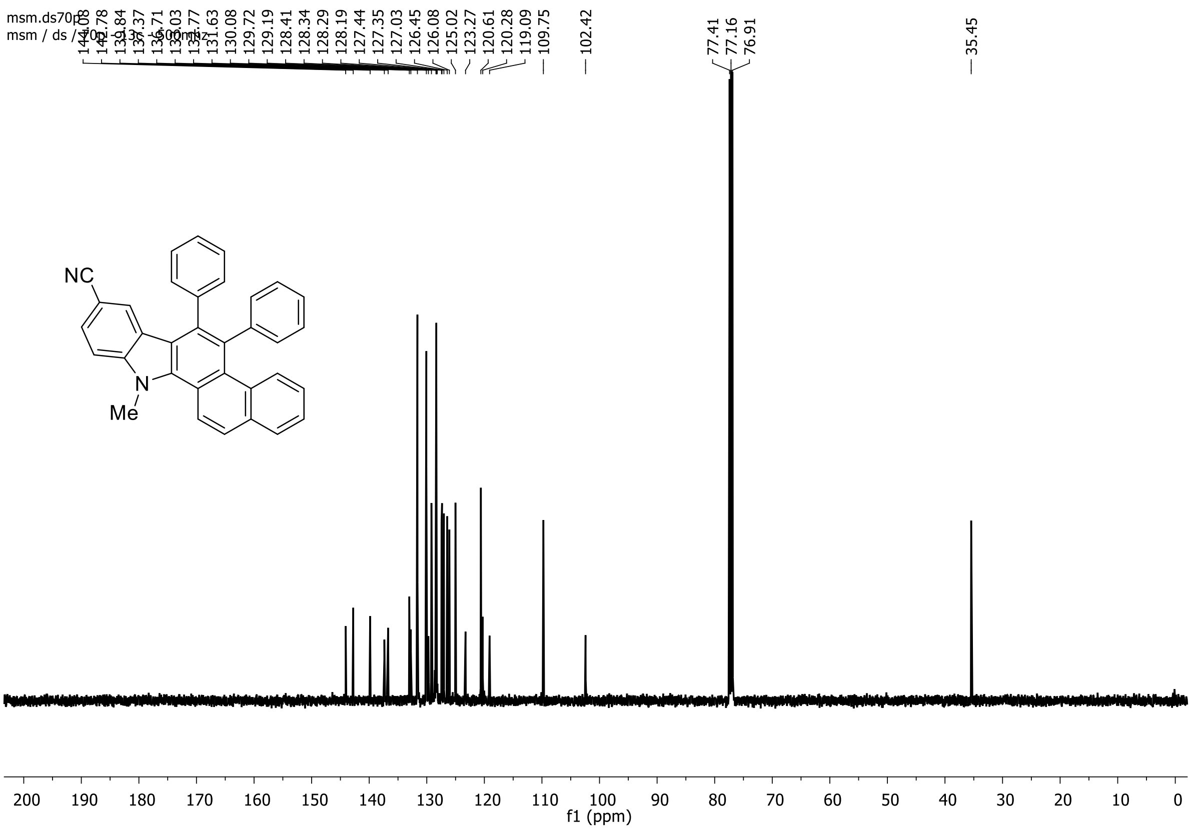

^{1}H (500 MHz) and ^{13}C (126 MHz) NMR of **NC6** in $CDCl_3$

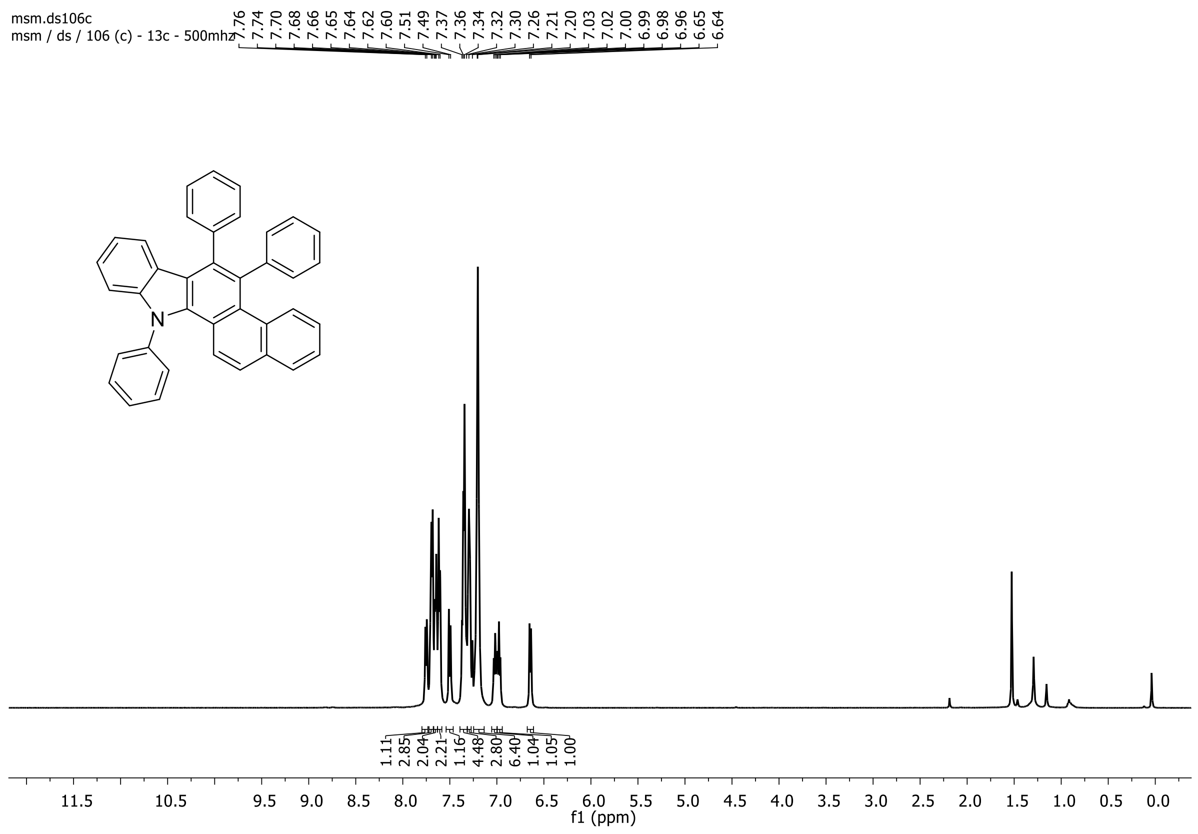

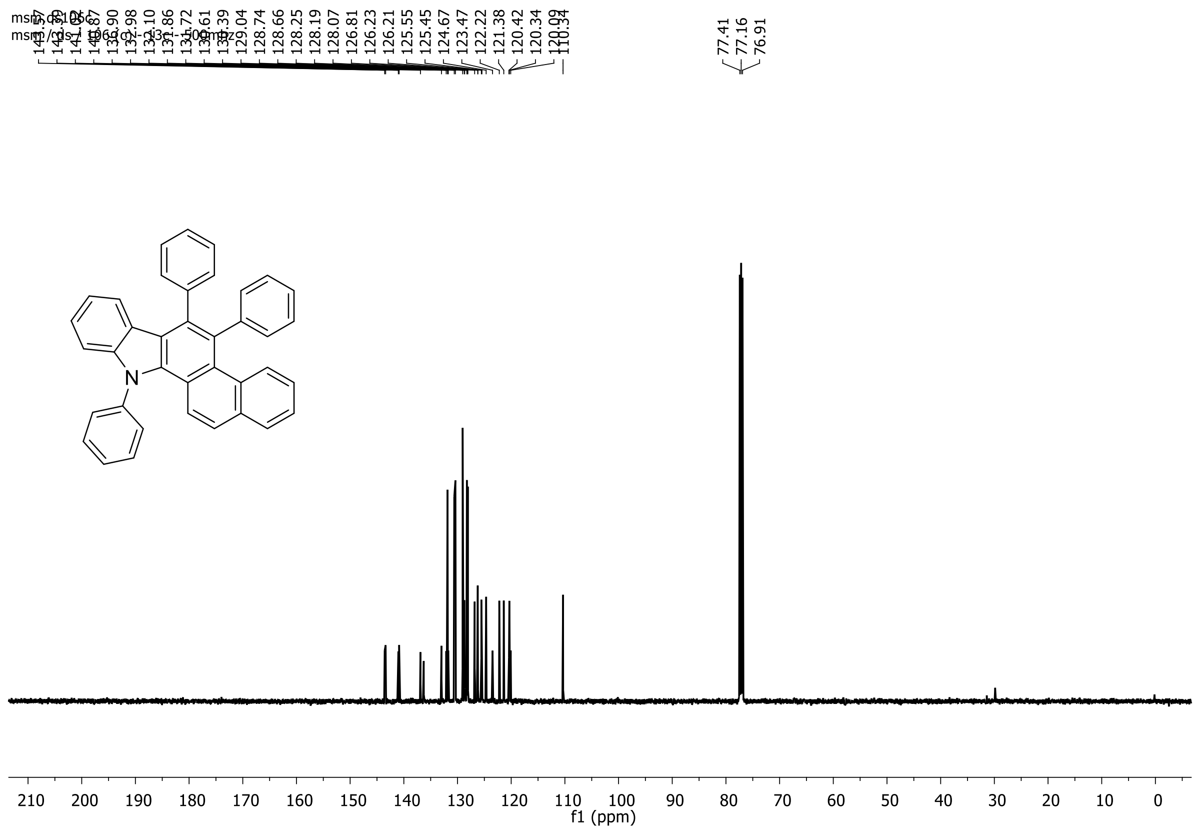

^{1}H (400 MHz) and ^{13}C (101 MHz) NMR of **4d** in $CDCl_3$

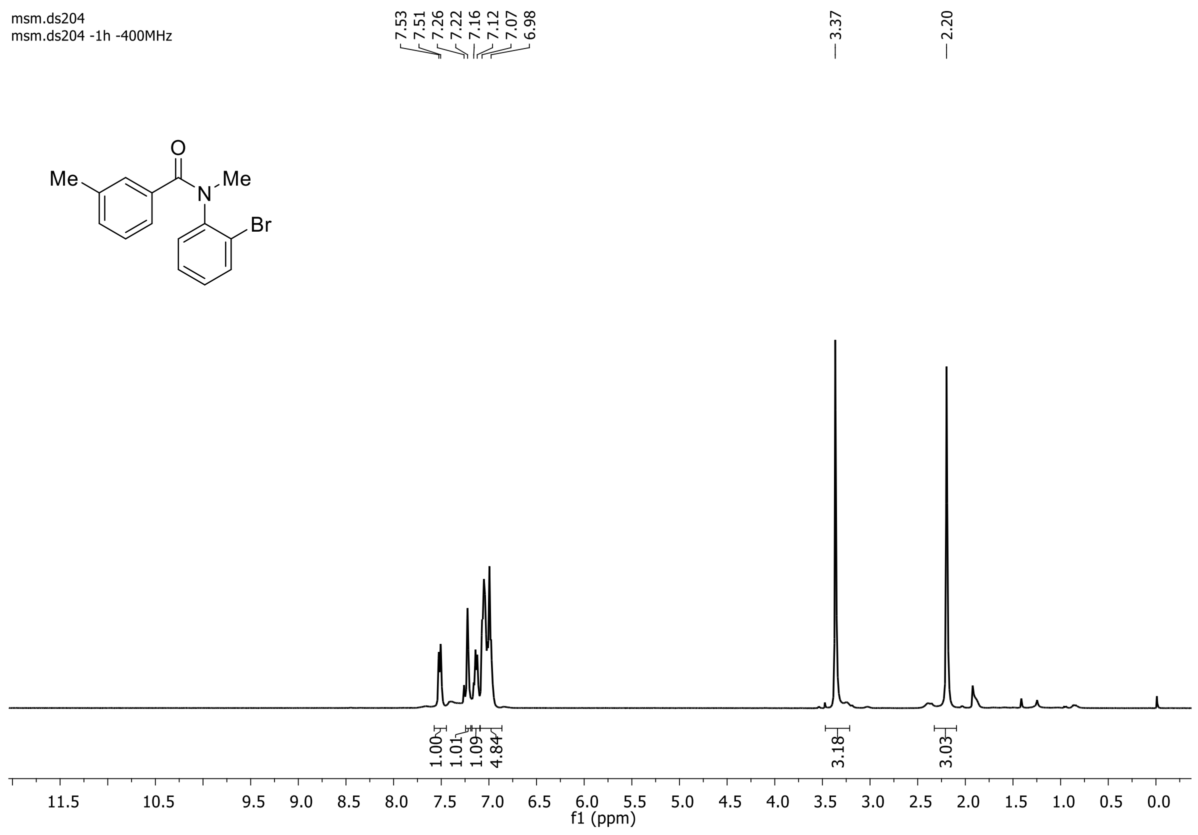

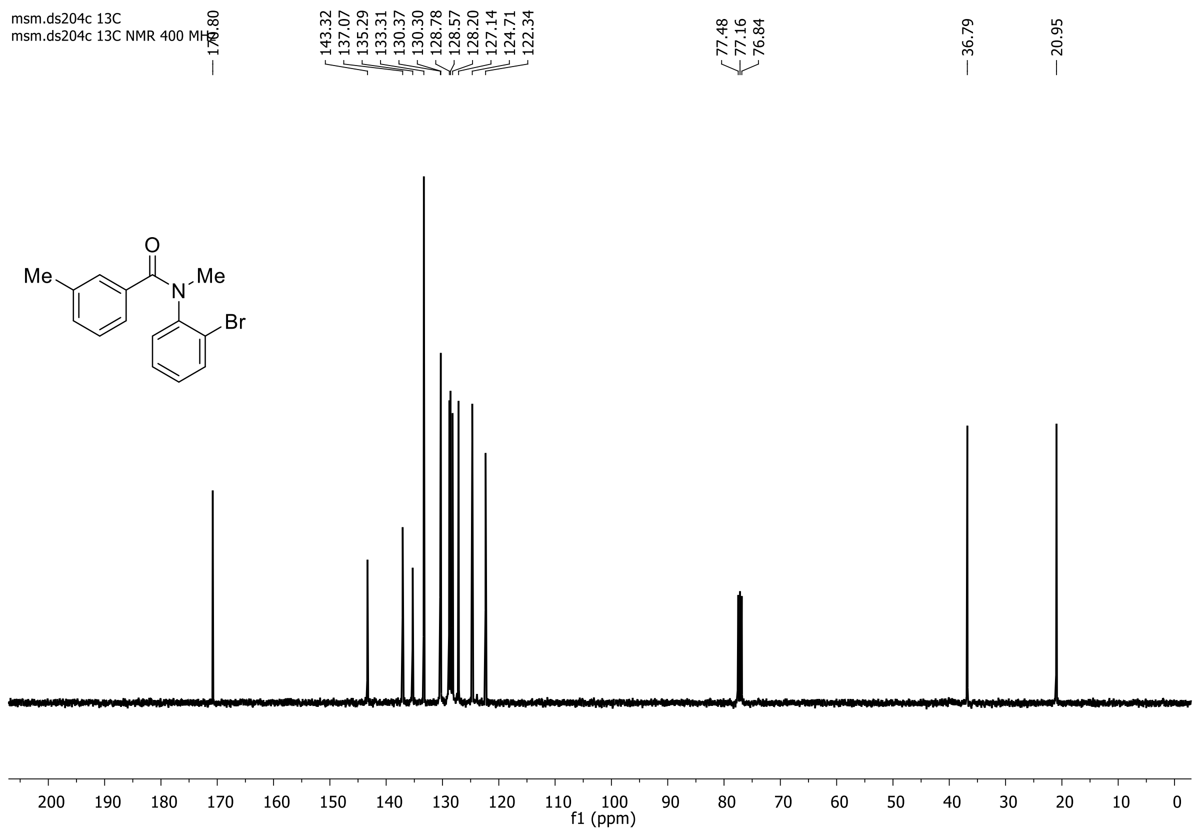

^{1}H (500 MHz) and ^{13}C (126 MHz) NMR of **4e** in $CDCl_3$

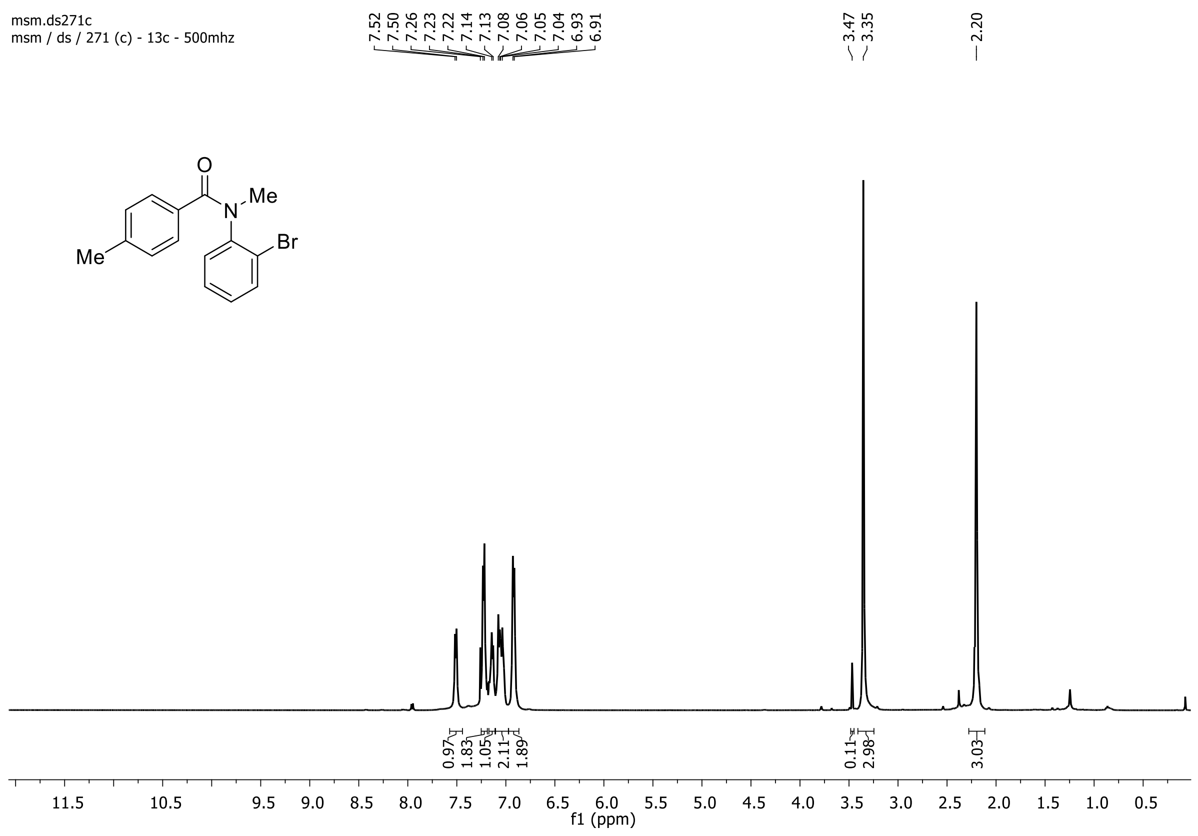

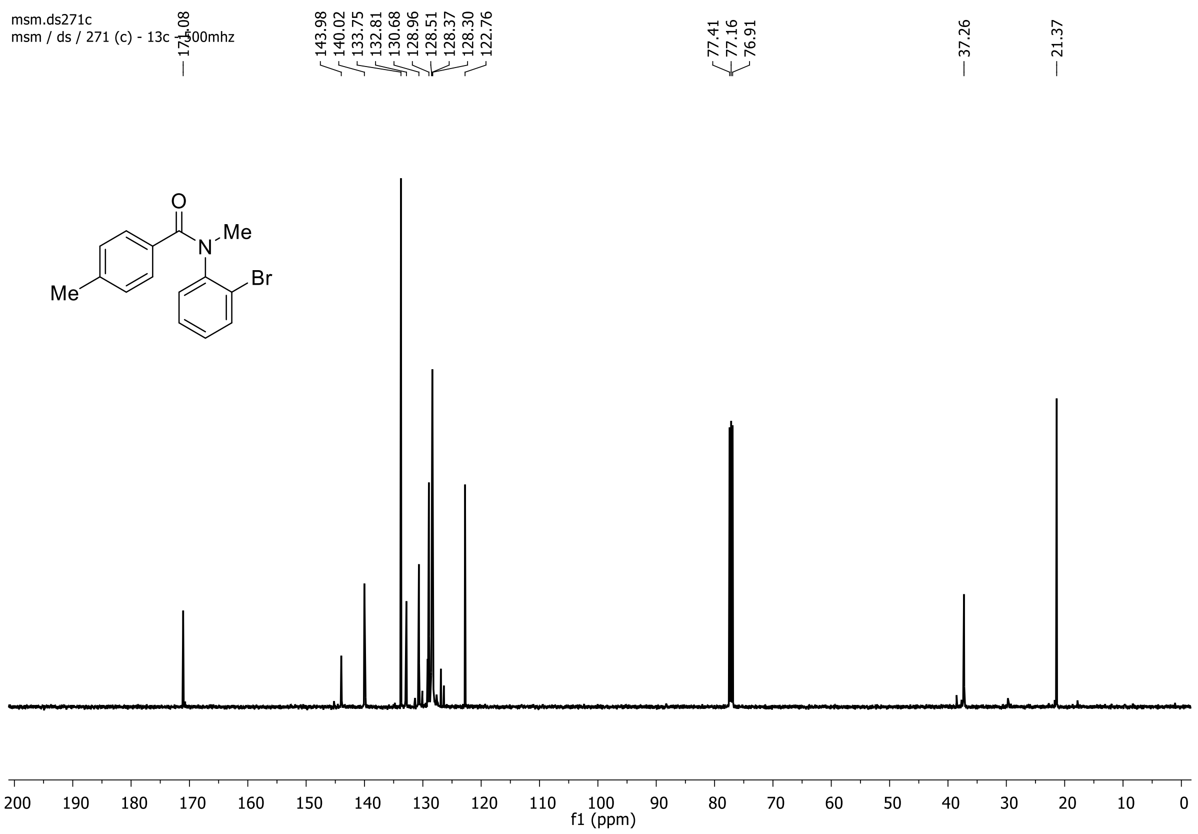

^{1}H (400 MHz) and ^{13}C (126 MHz) NMR of **4f** in $CDCl_3$

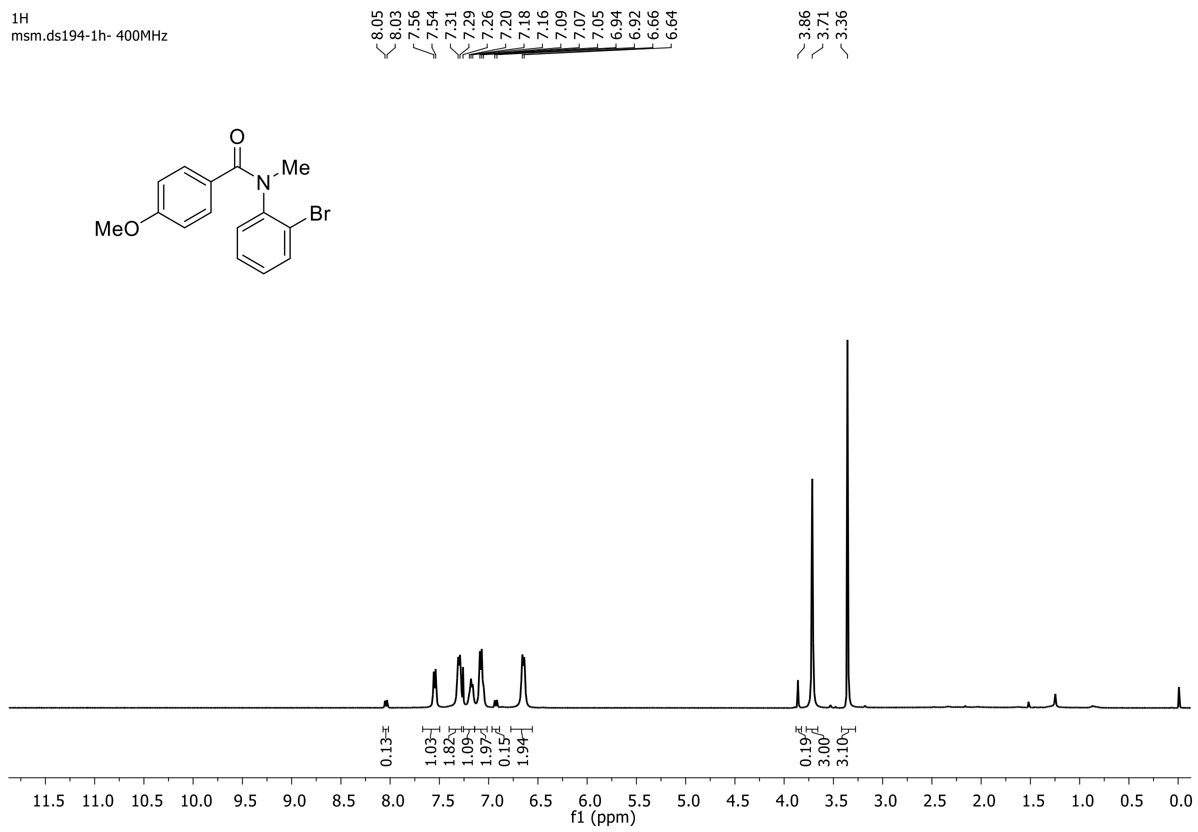

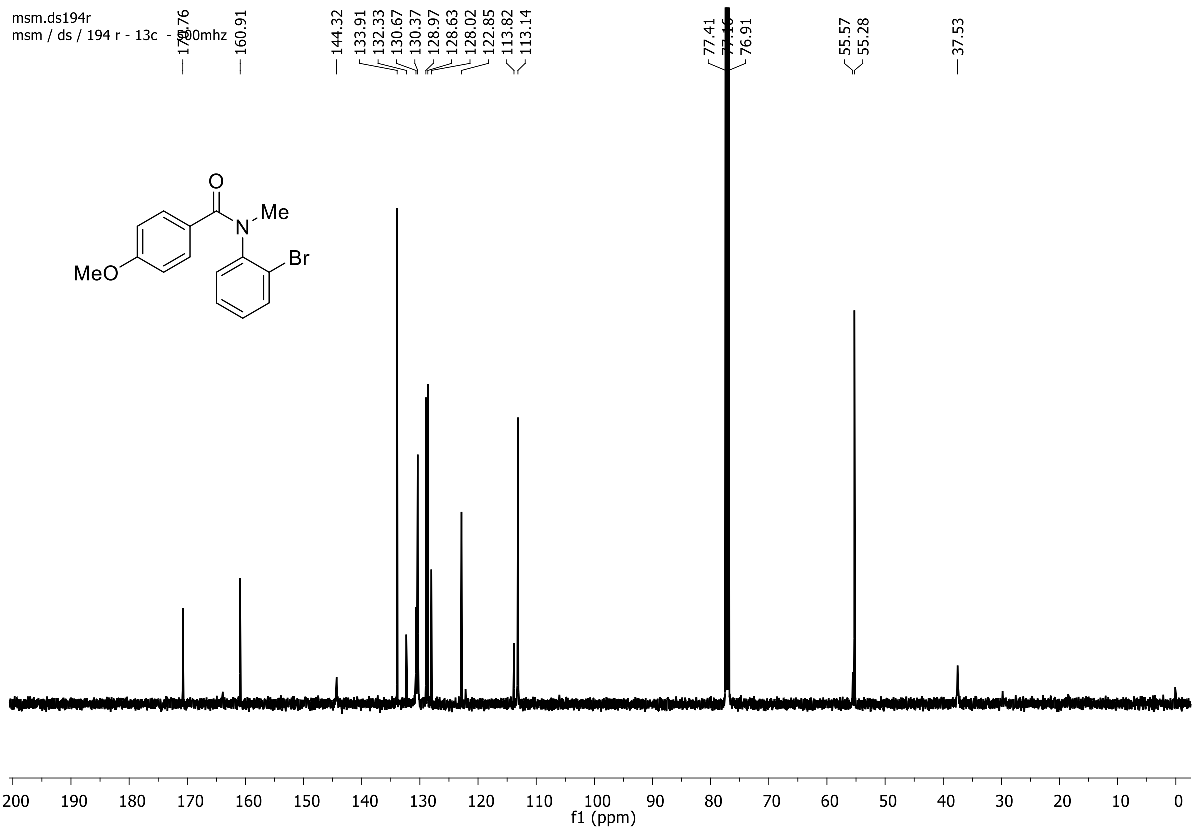

^{1}H (400 MHz) and ^{13}C (101 MHz) NMR of **4g** in $CDCl_3$

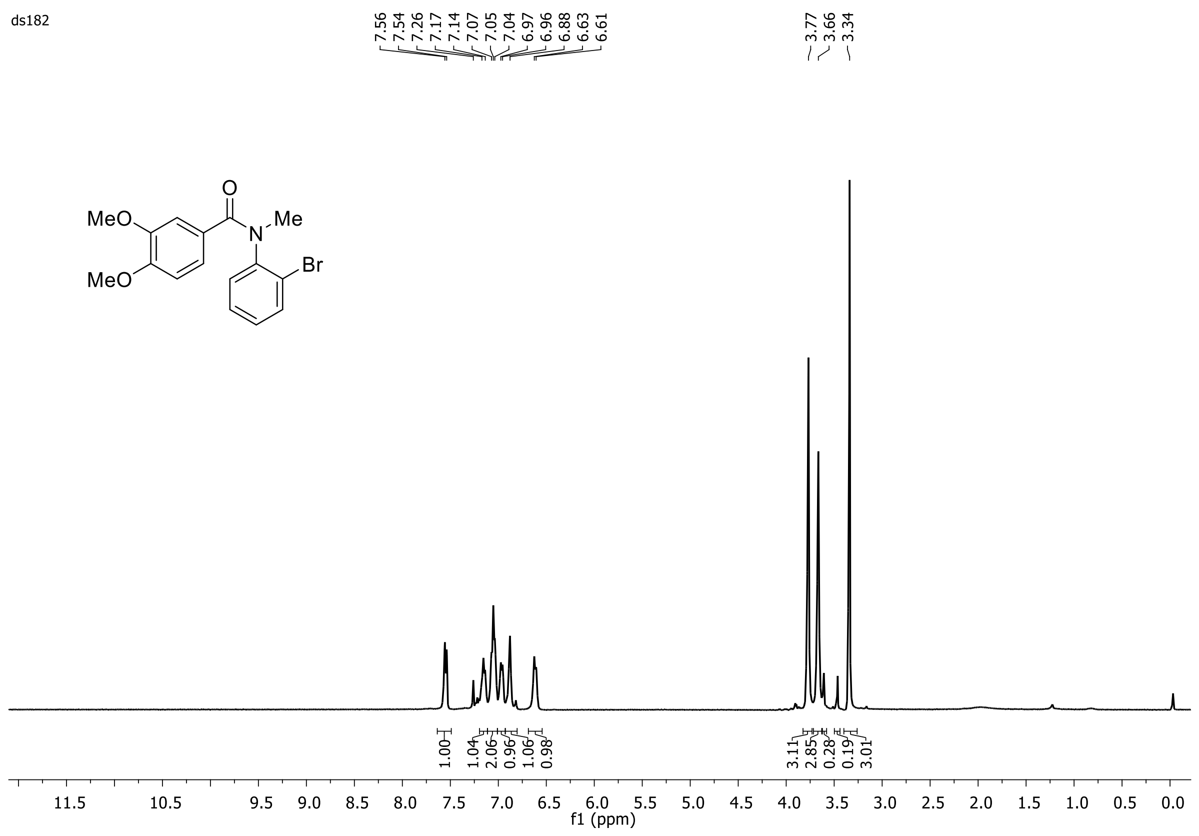

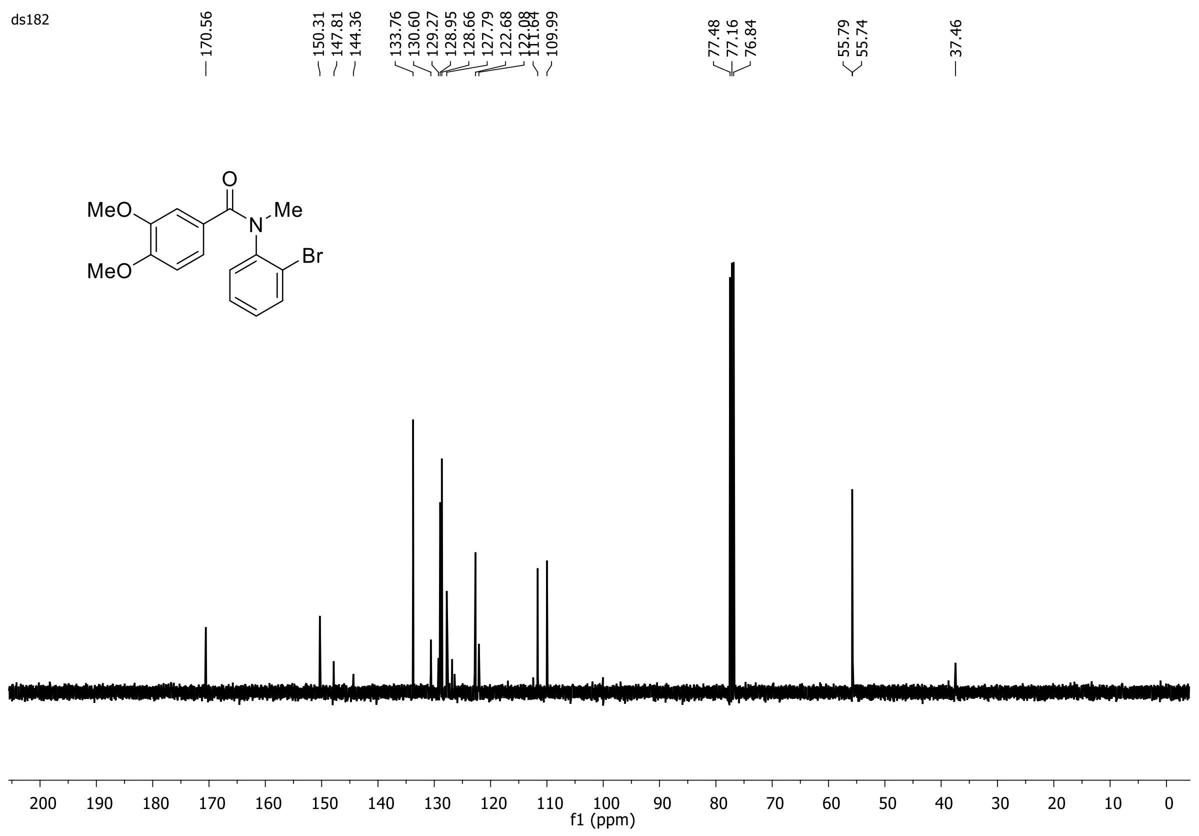

^{1}H (400 MHz) and ^{13}C (101 MHz) NMR of **4h** in $CDCl_3$

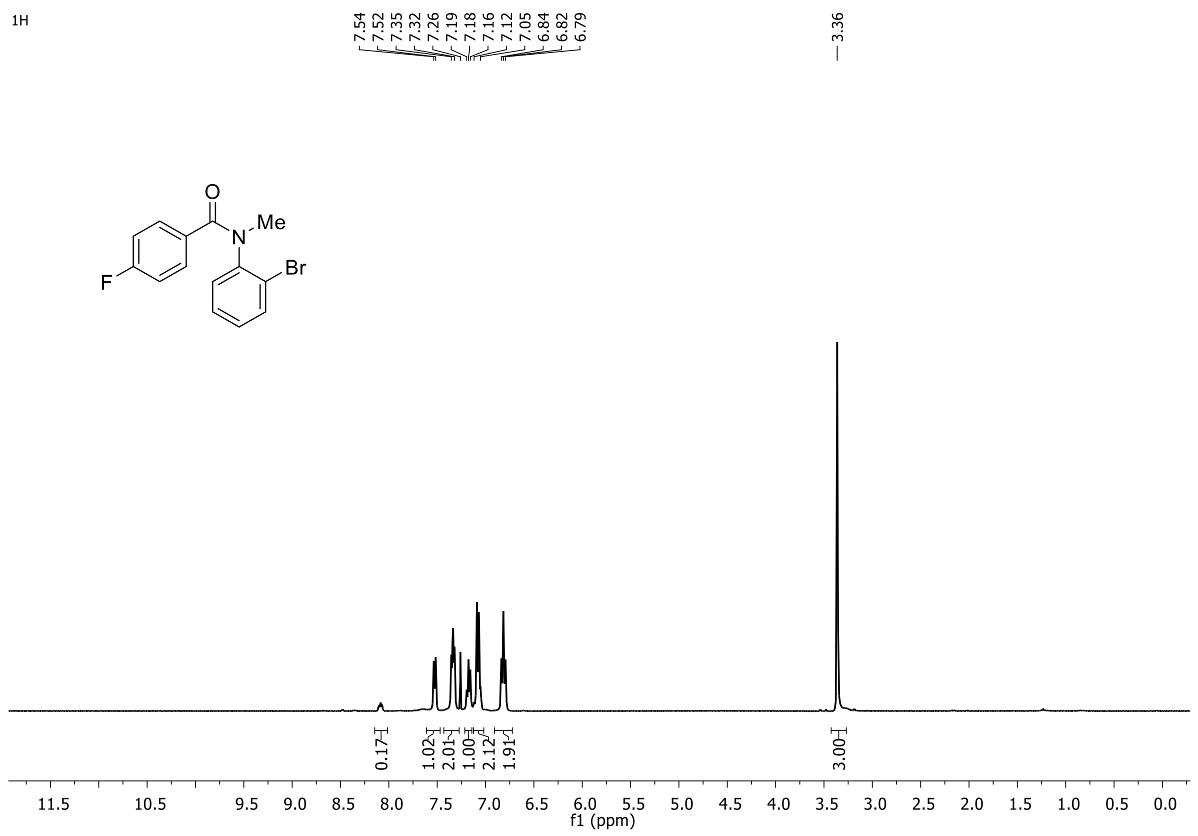

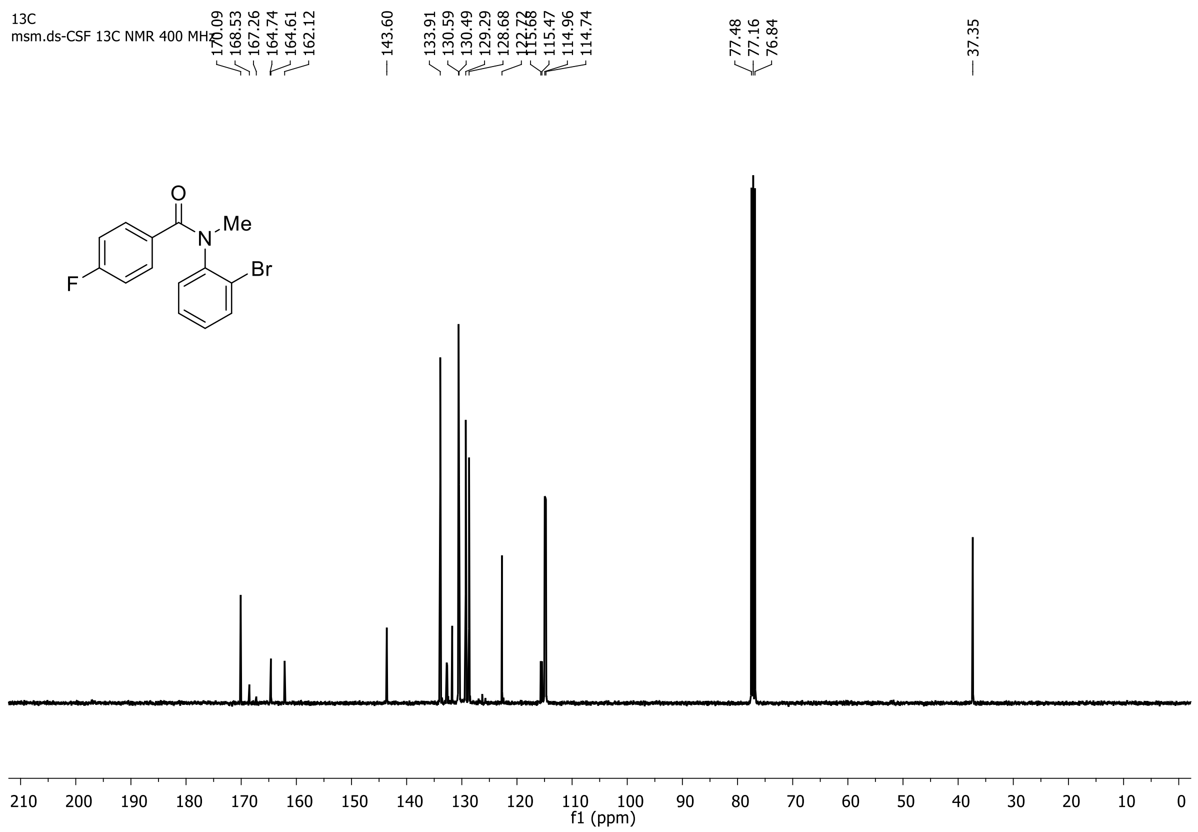

^{19}F (470 MHz) NMR of **4h** in $CDCl_3$

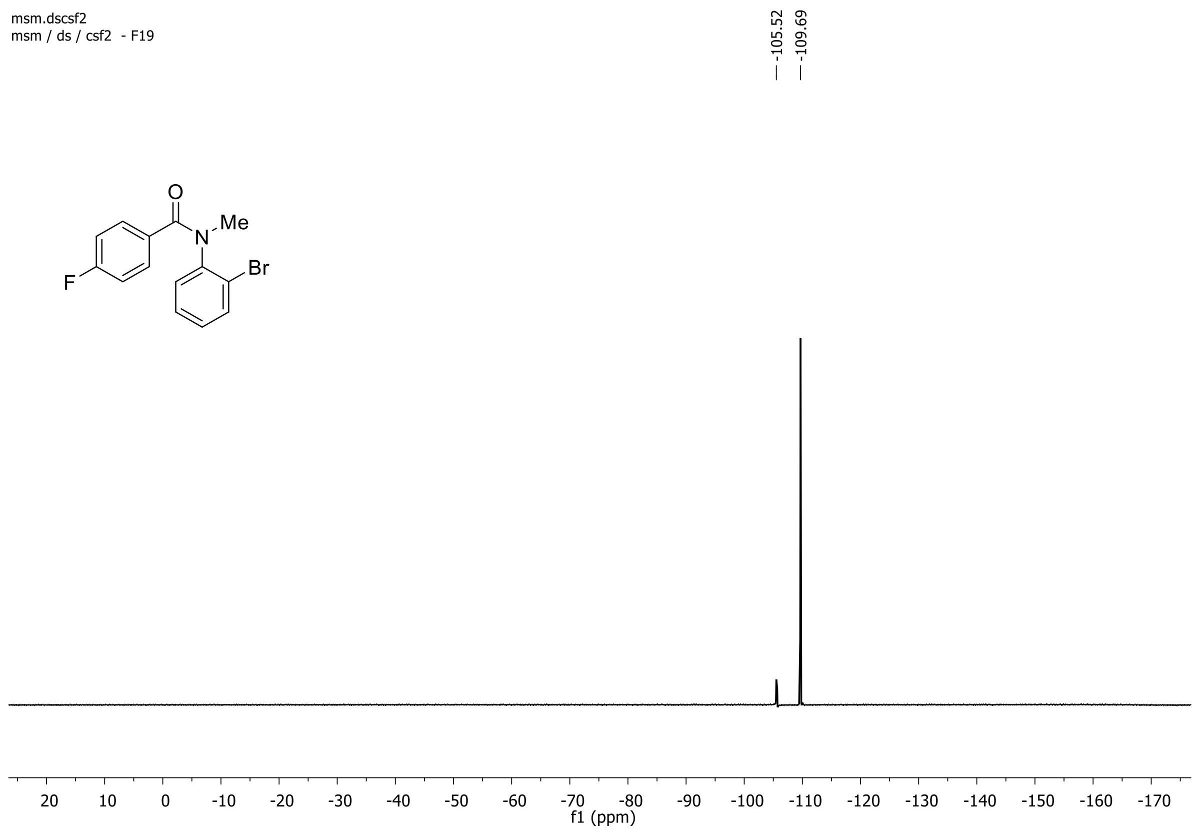

^{1}H (500 MHz) and ^{13}C (126 MHz) NMR of **4i** in $CDCl_3$

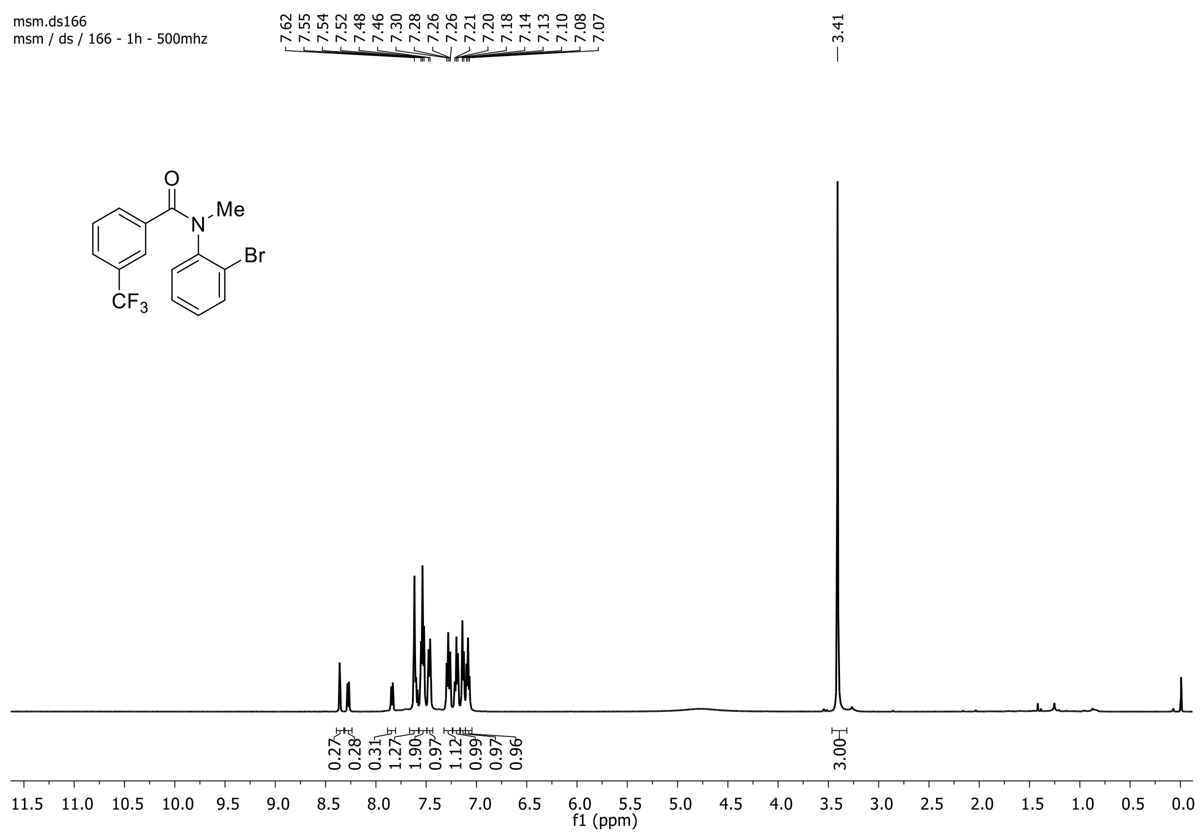

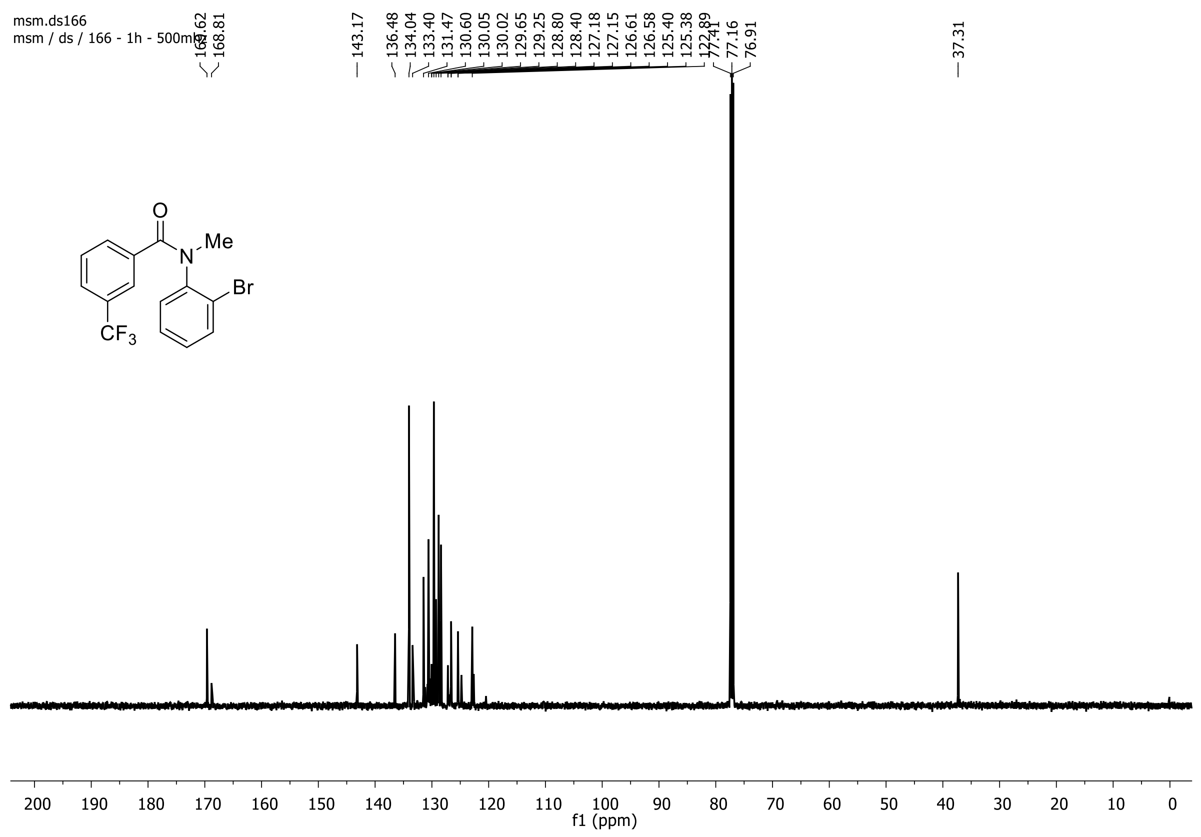

^{19}F (471 MHz) NMR of **4i** in $CDCl_3$

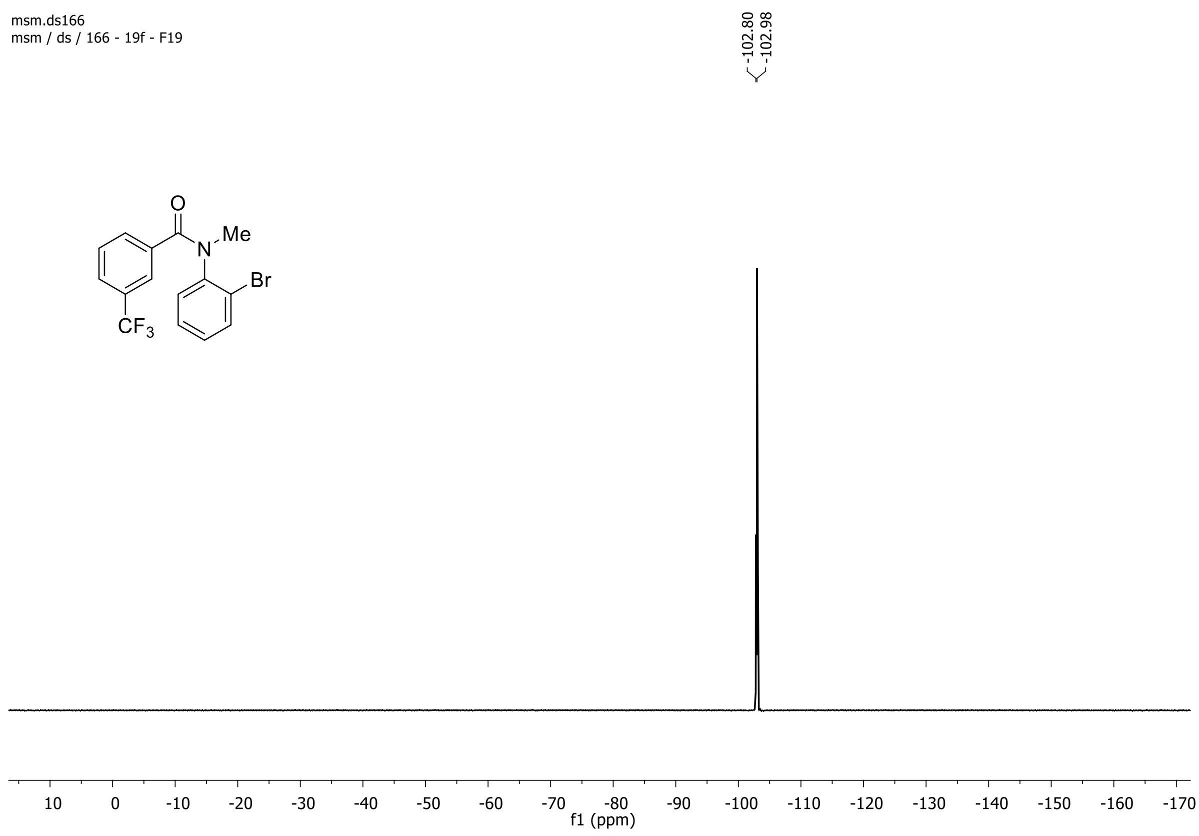

^{1}H (400 MHz) and ^{13}C (101 MHz) NMR of **4j** in $CDCl_3$

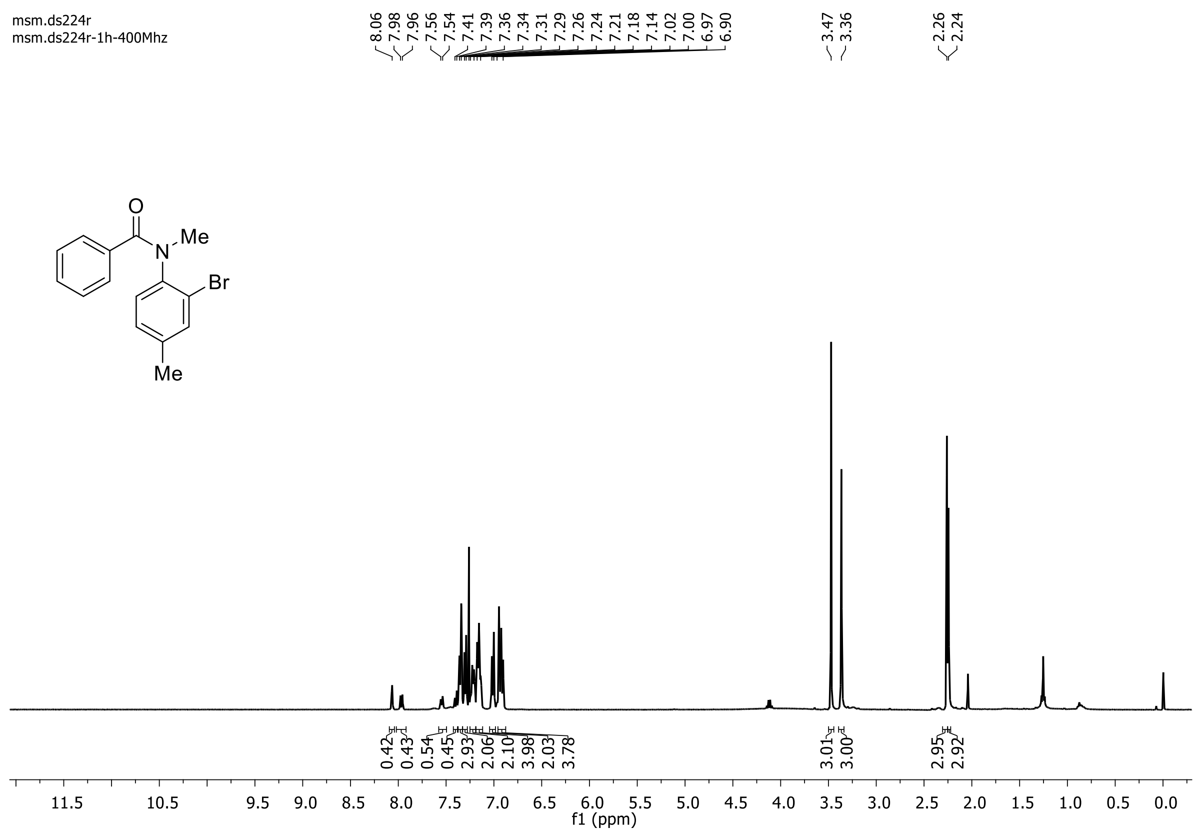

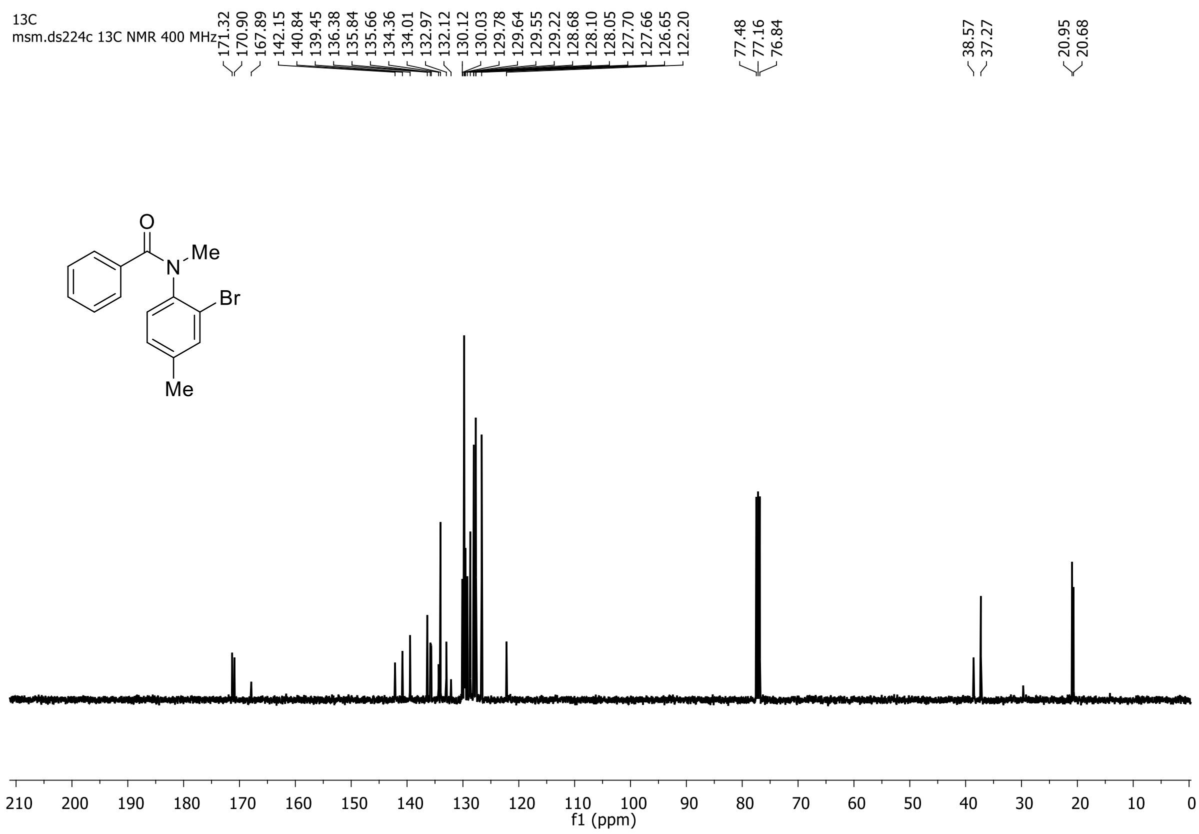

^{1}H (500 MHz) and ^{13}C (126 MHz) NMR of **4k** in $CDCl_3$

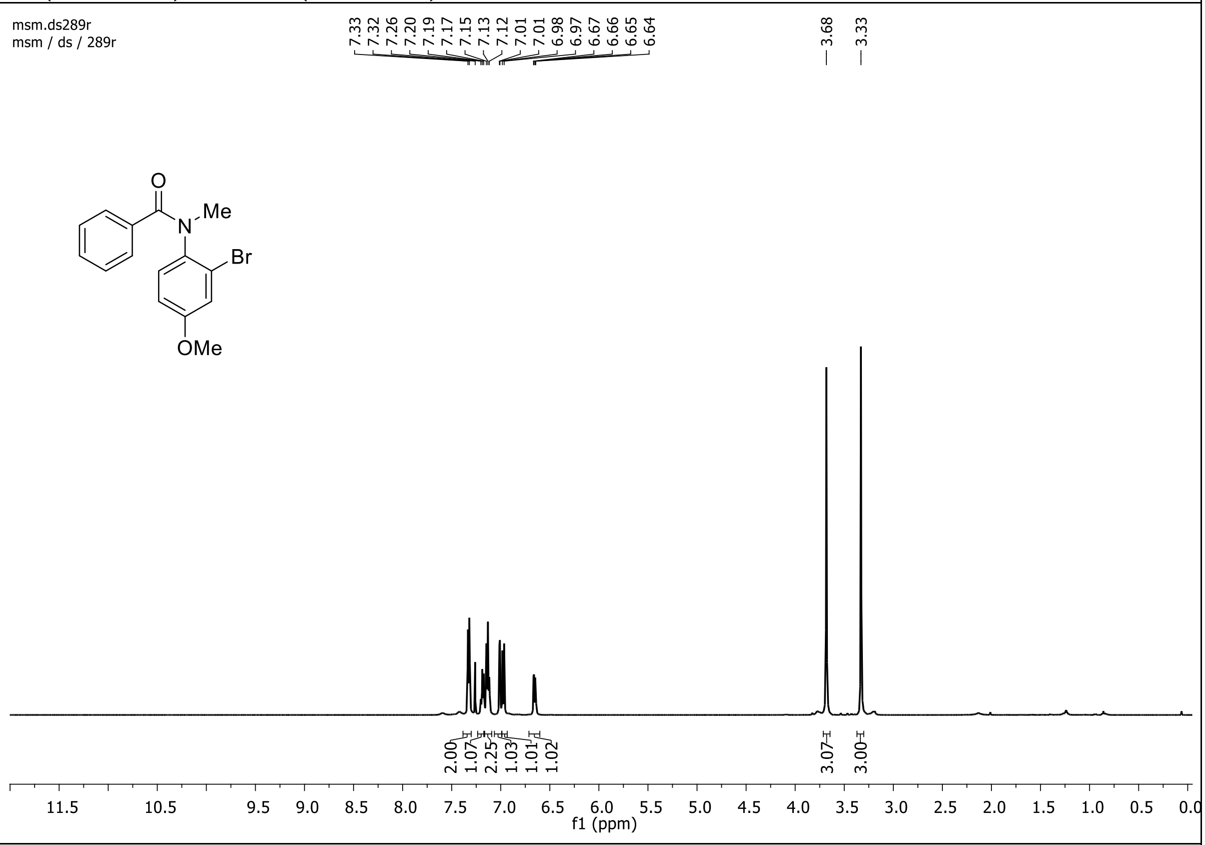

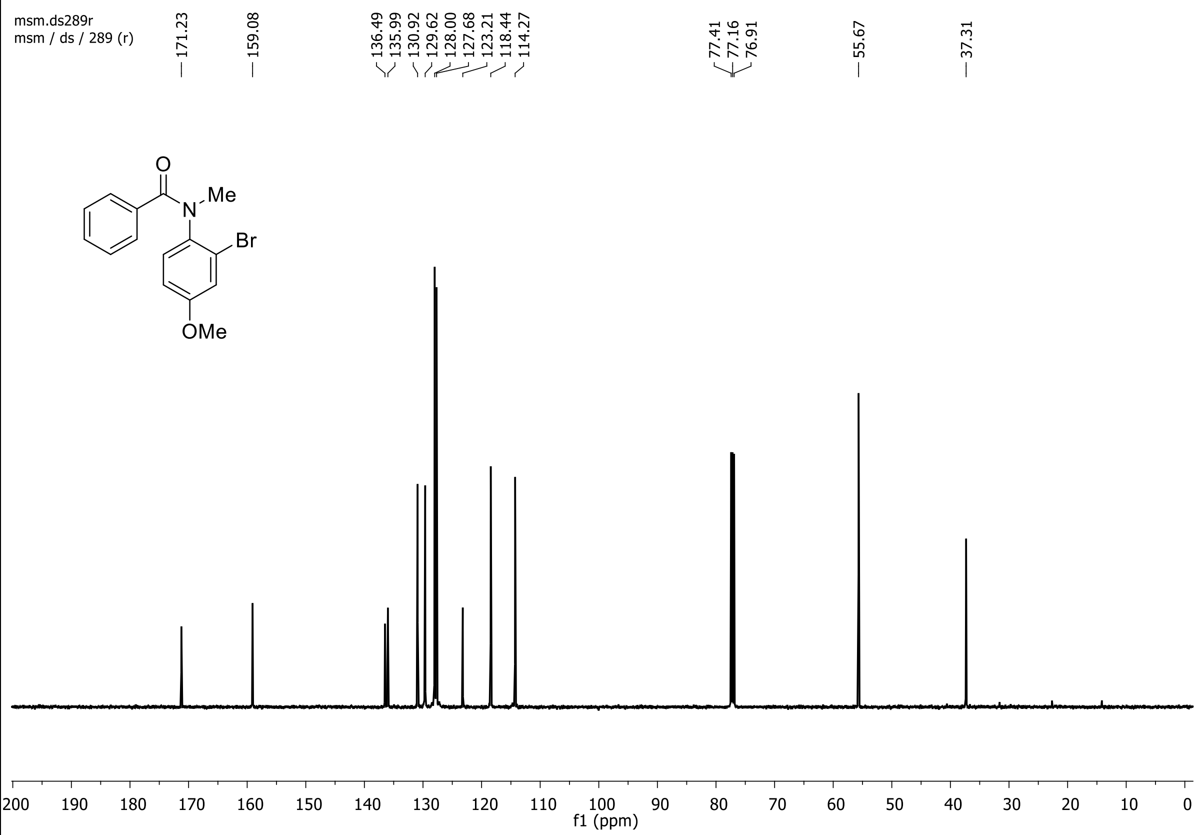

^{1}H (400 MHz) and ^{13}C (101 MHz) NMR of **4l** in $CDCl_3$

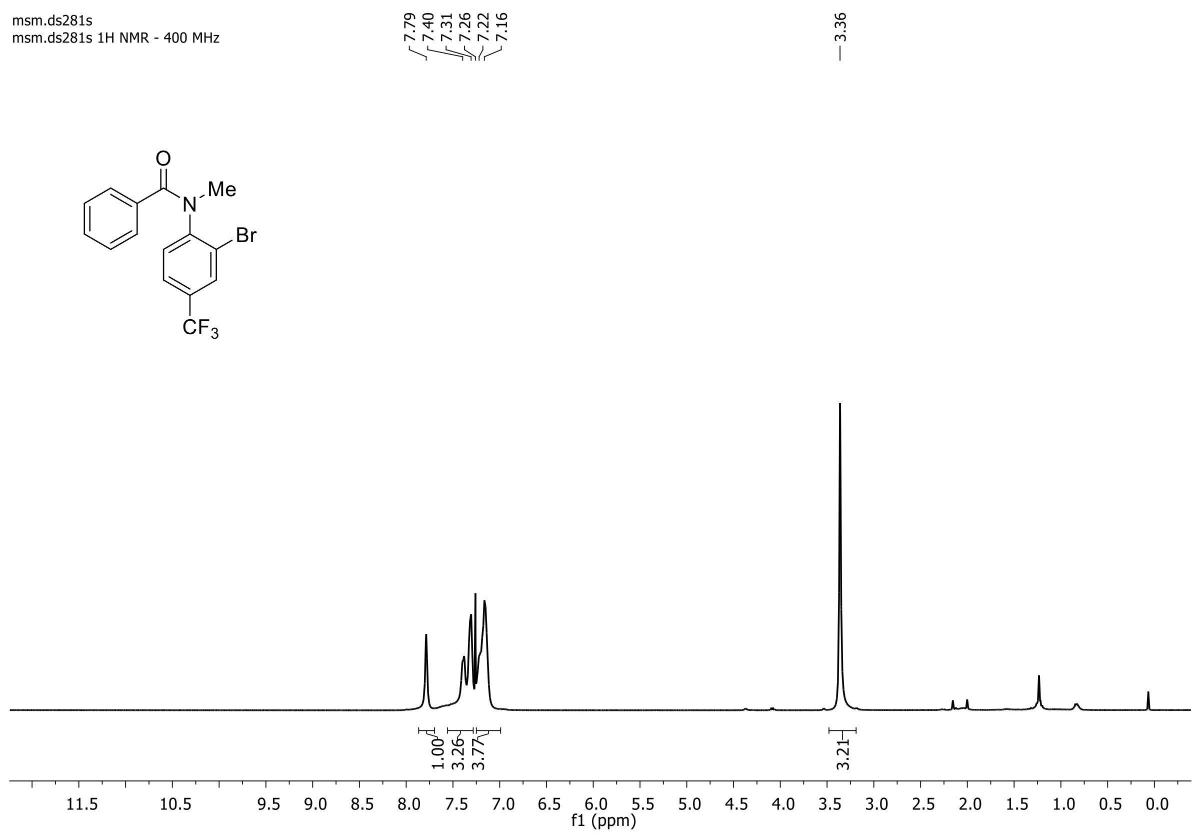

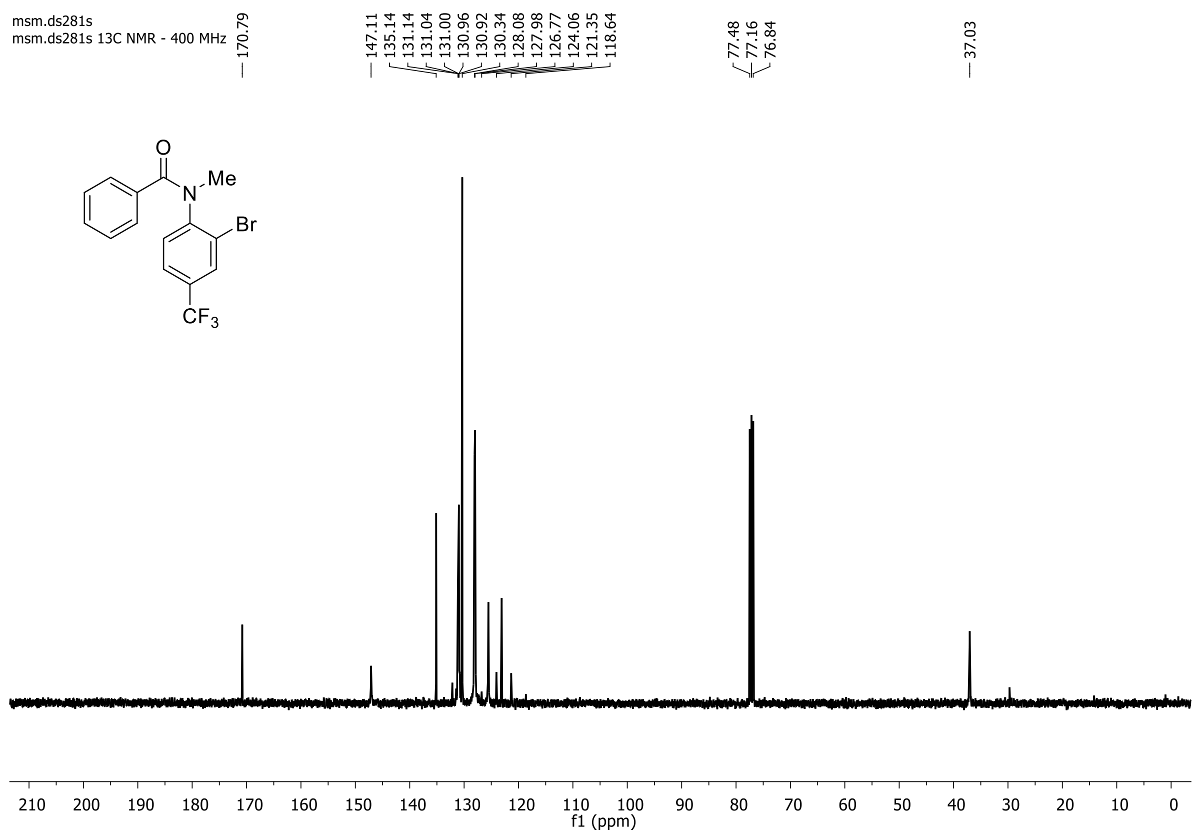

^{19}F (471 MHz) NMR of **4l** in $CDCl_3$

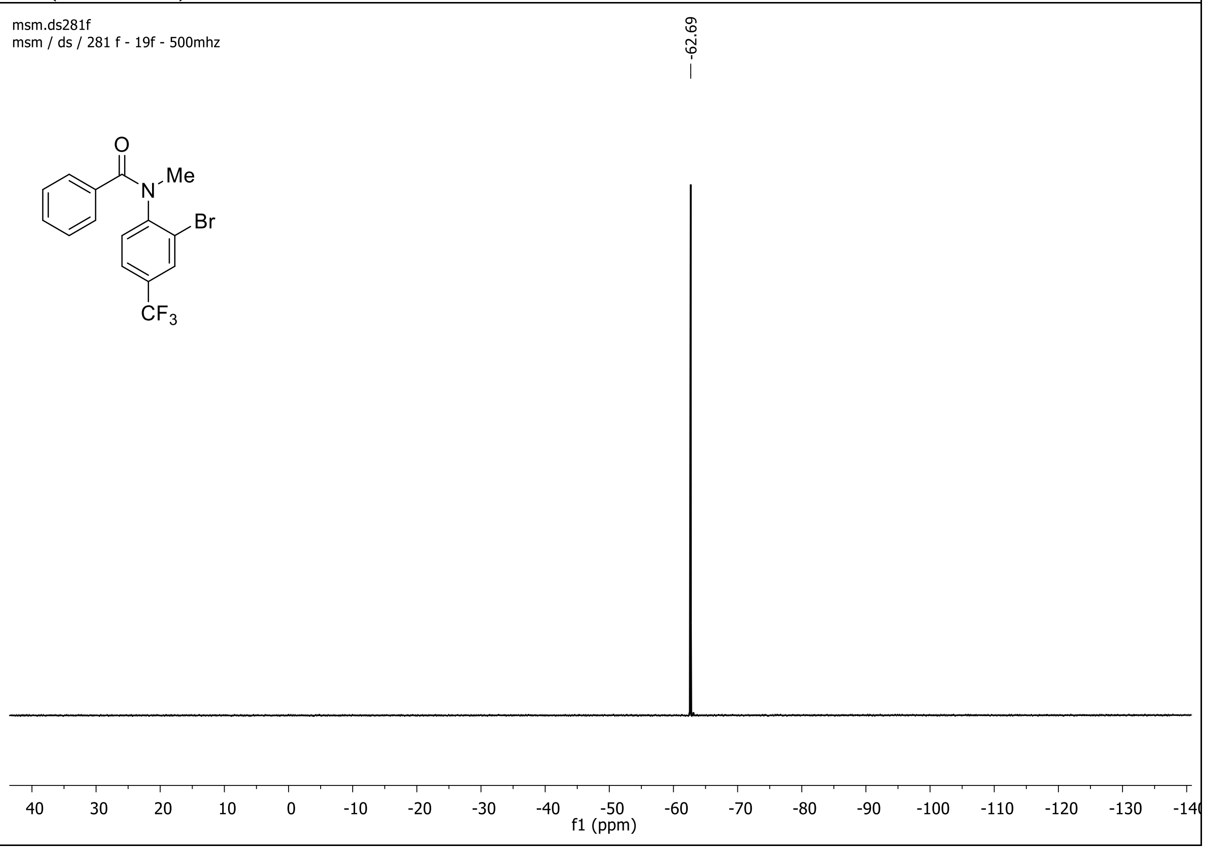

^{1}H (500 MHz) and ^{13}C (126 MHz) NMR of **4n** in $CDCl_3$

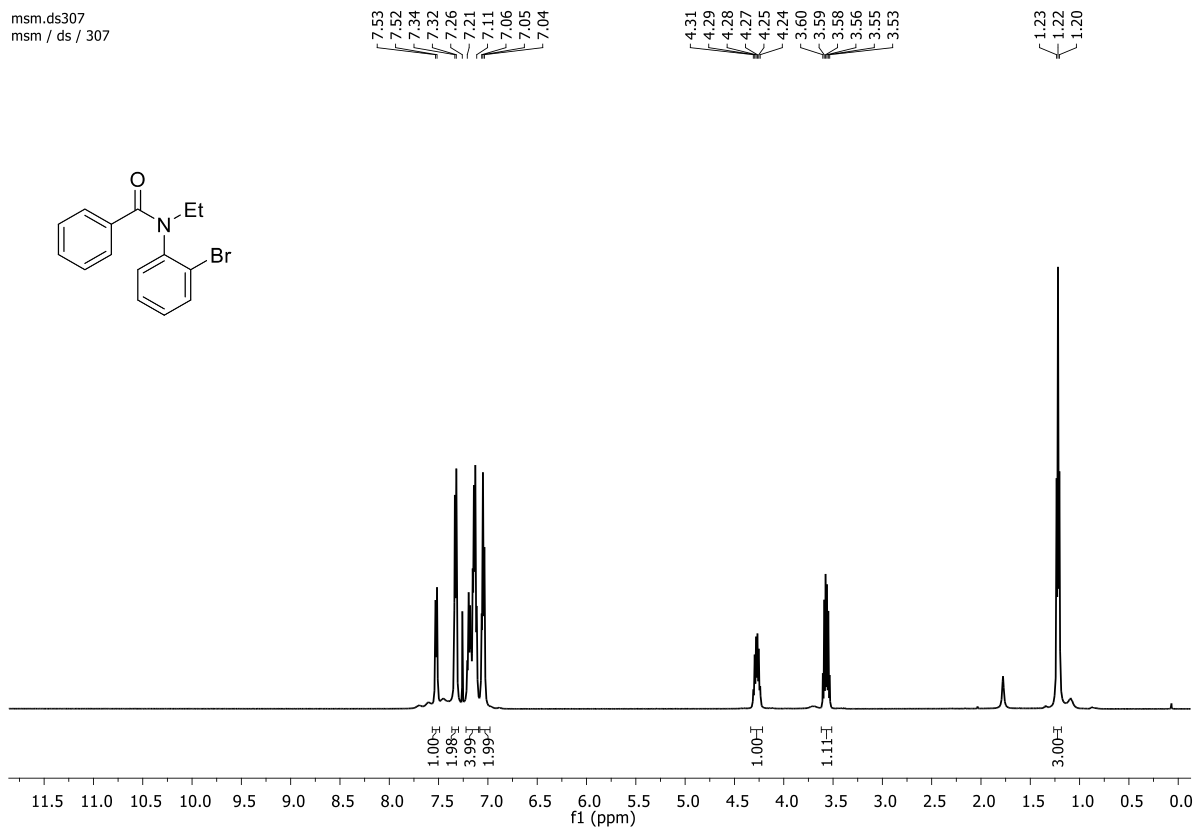

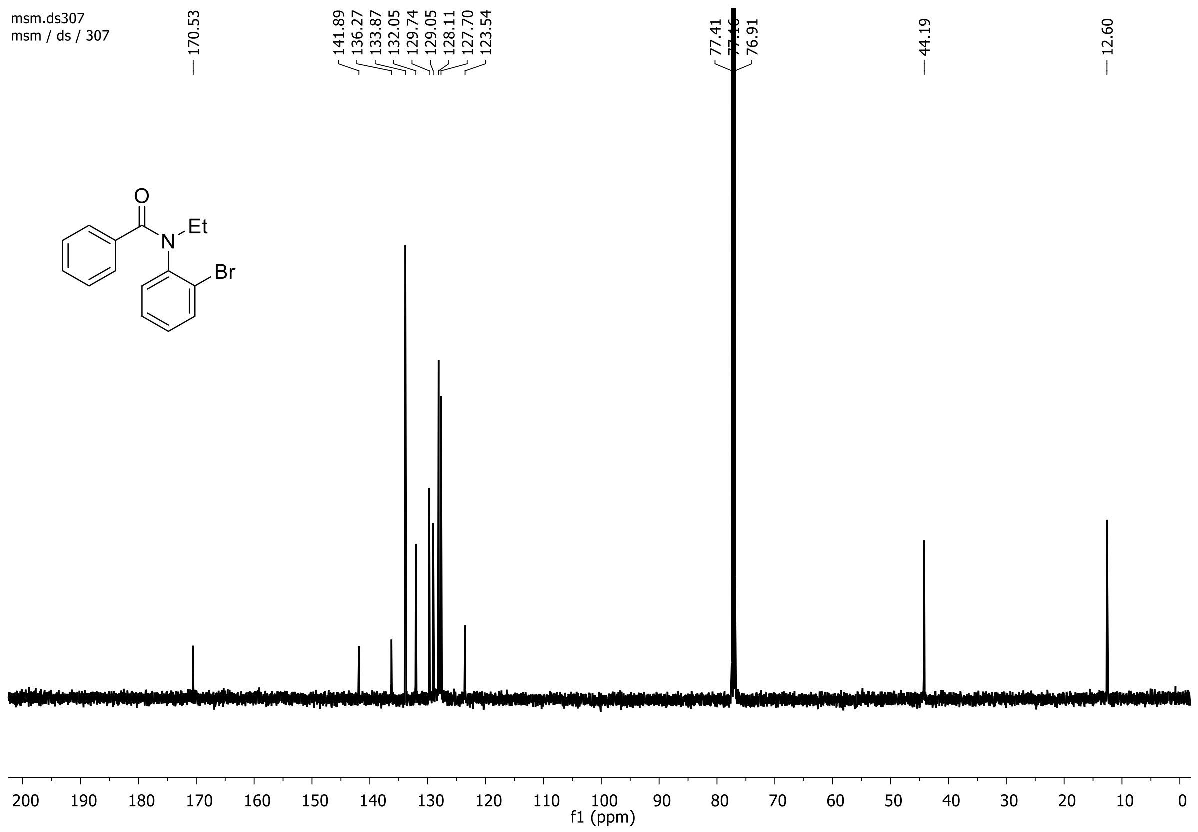

^{1}H (400 MHz) and ^{13}C (126 MHz) NMR of **4o** in $CDCl_3$

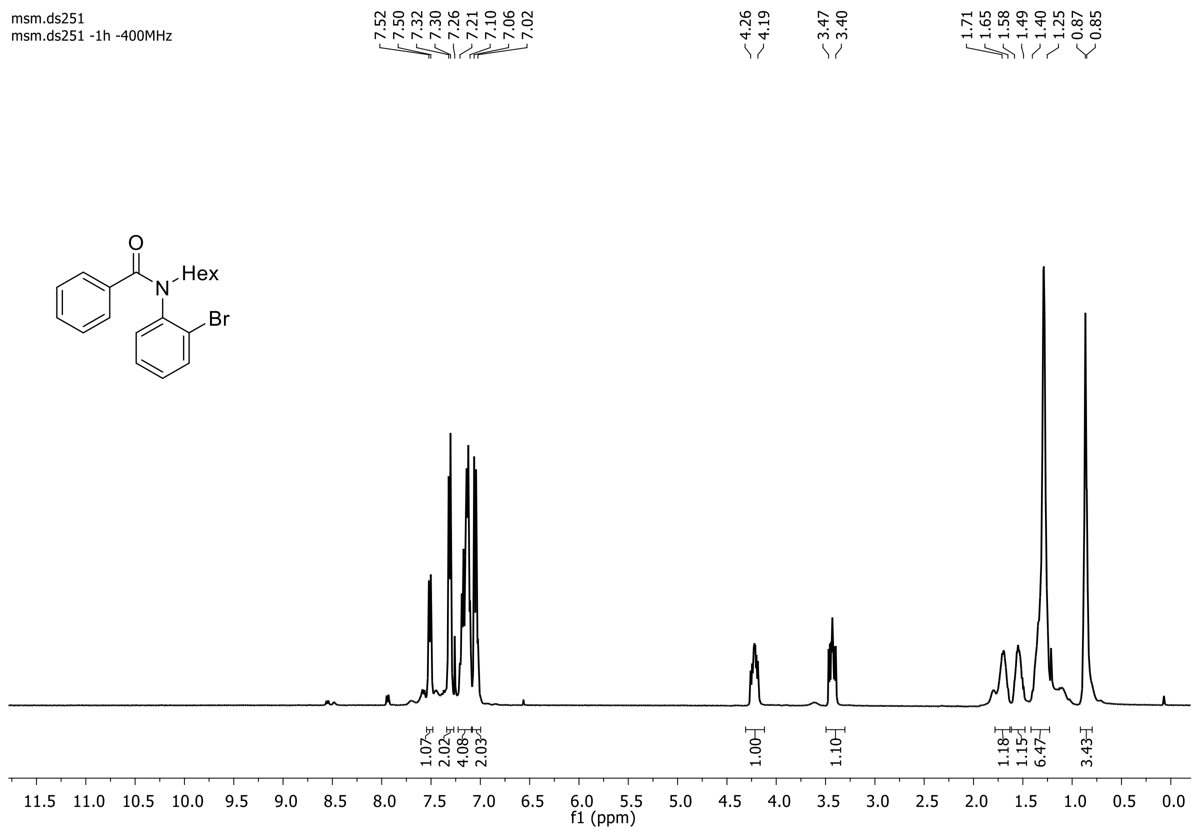

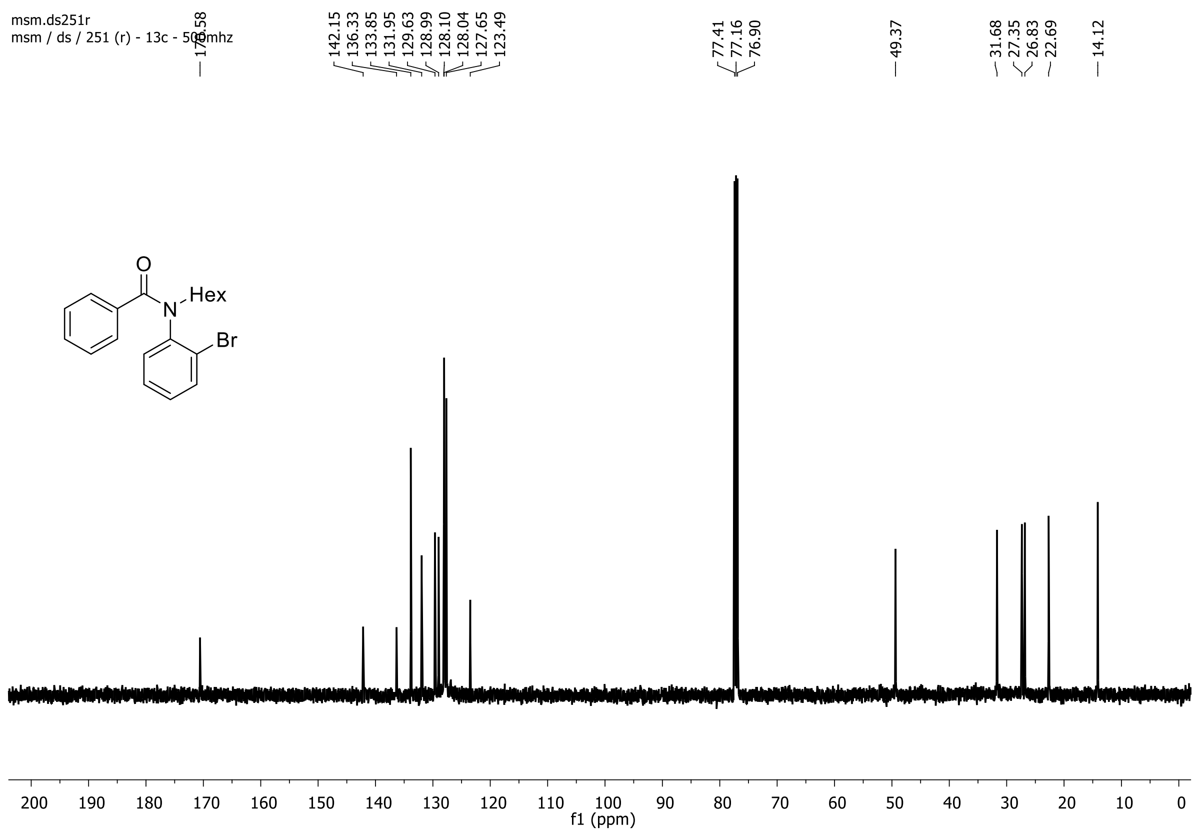

^{1}H (400 MHz) and ^{13}C (126 MHz) NMR of **10q** in $CDCl_3$

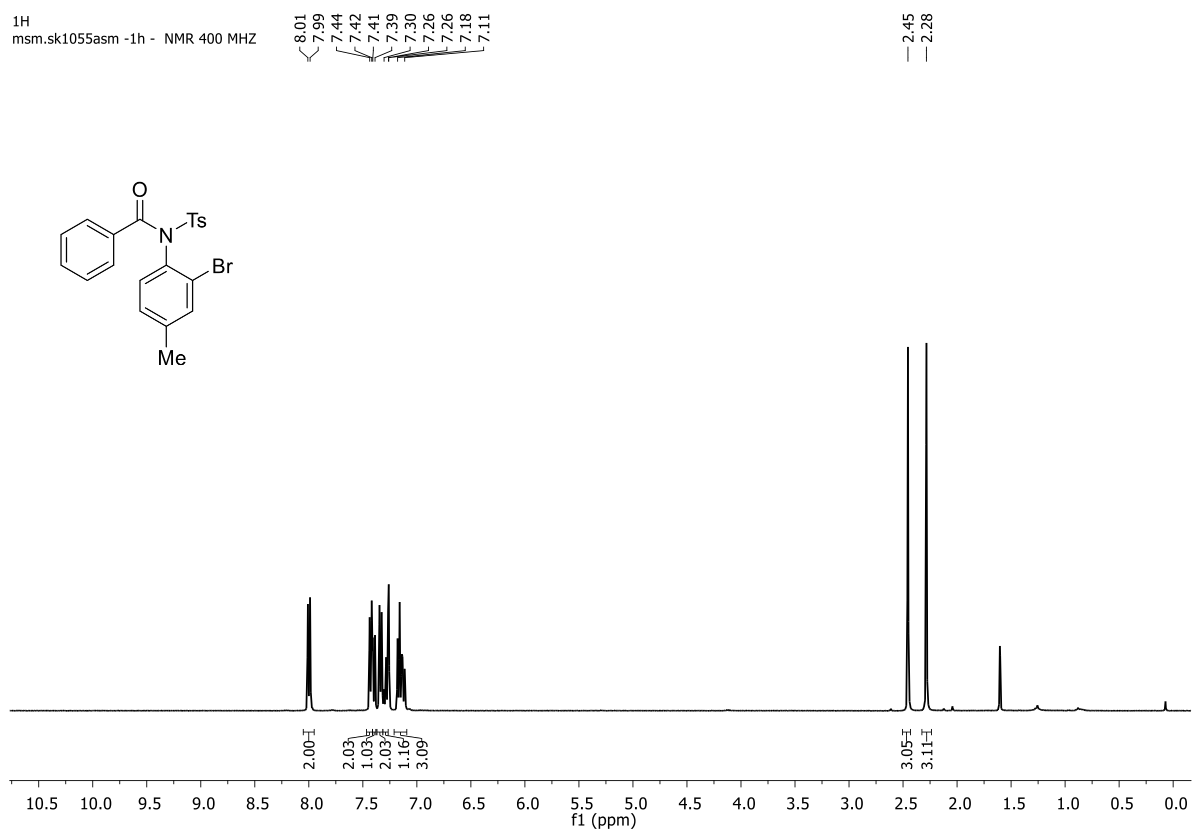

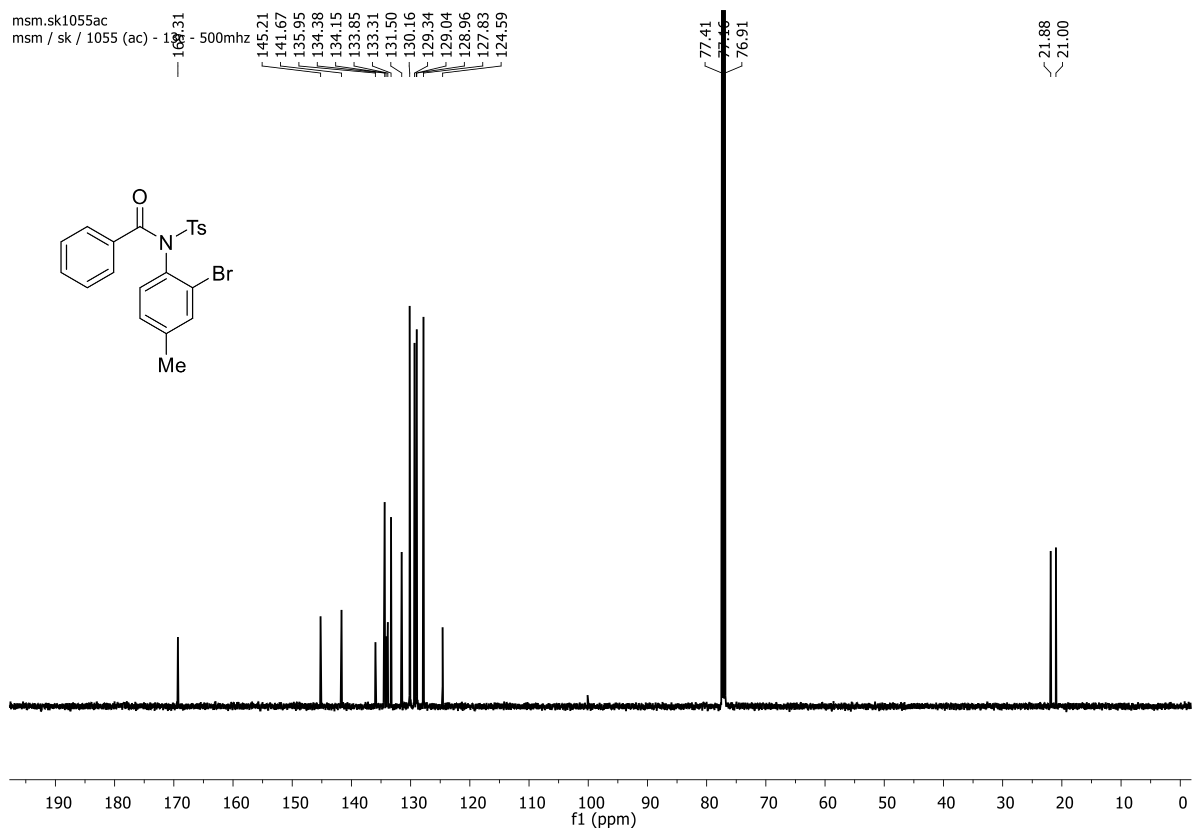

^{1}H (400 MHz) and ^{13}C (101 MHz) NMR of **2a** in $CDCl_3$

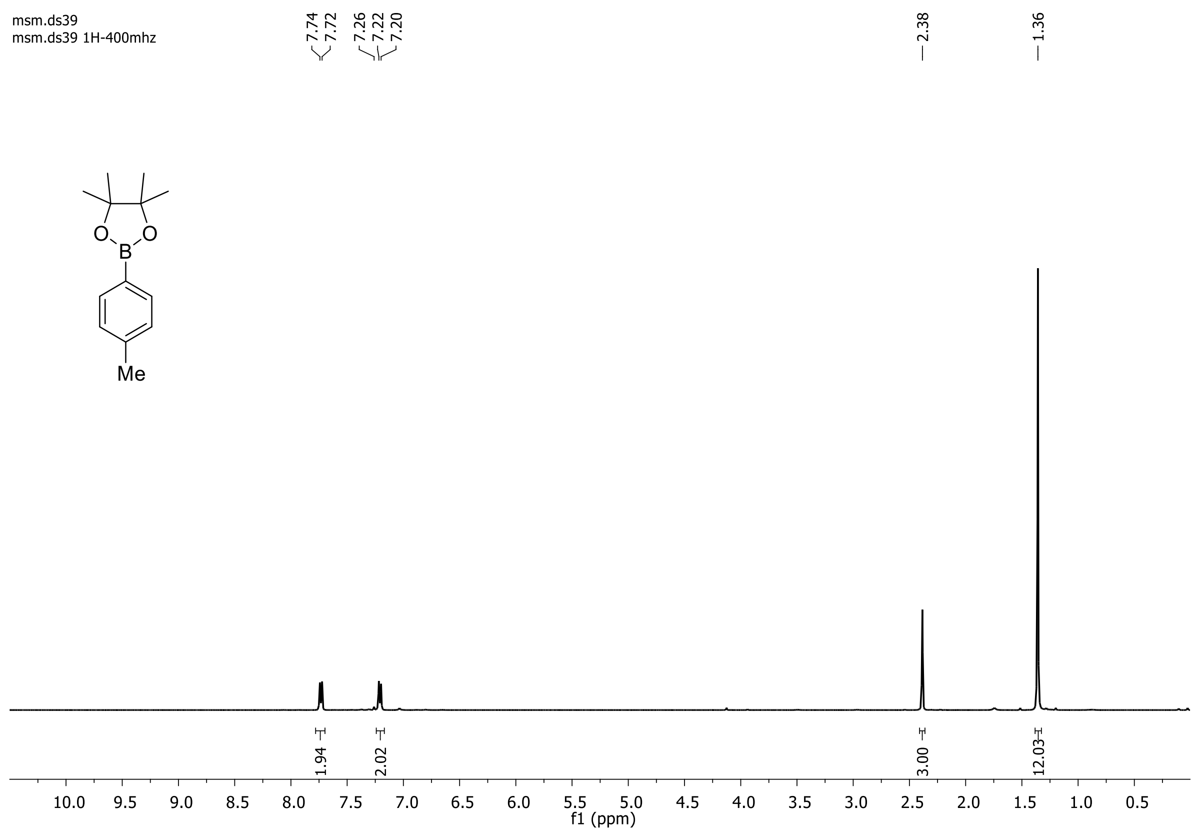

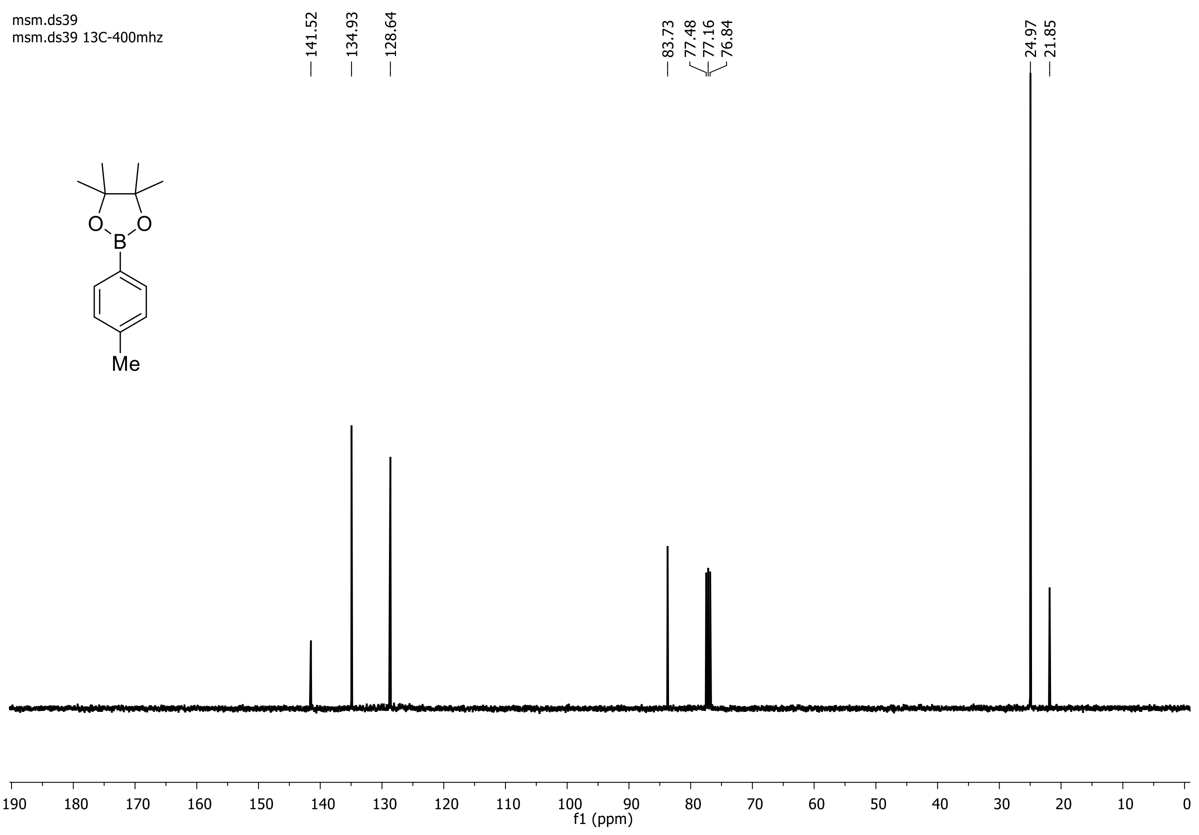

^{11}B (160 MHz) NMR of **2a** in $CDCl_3$

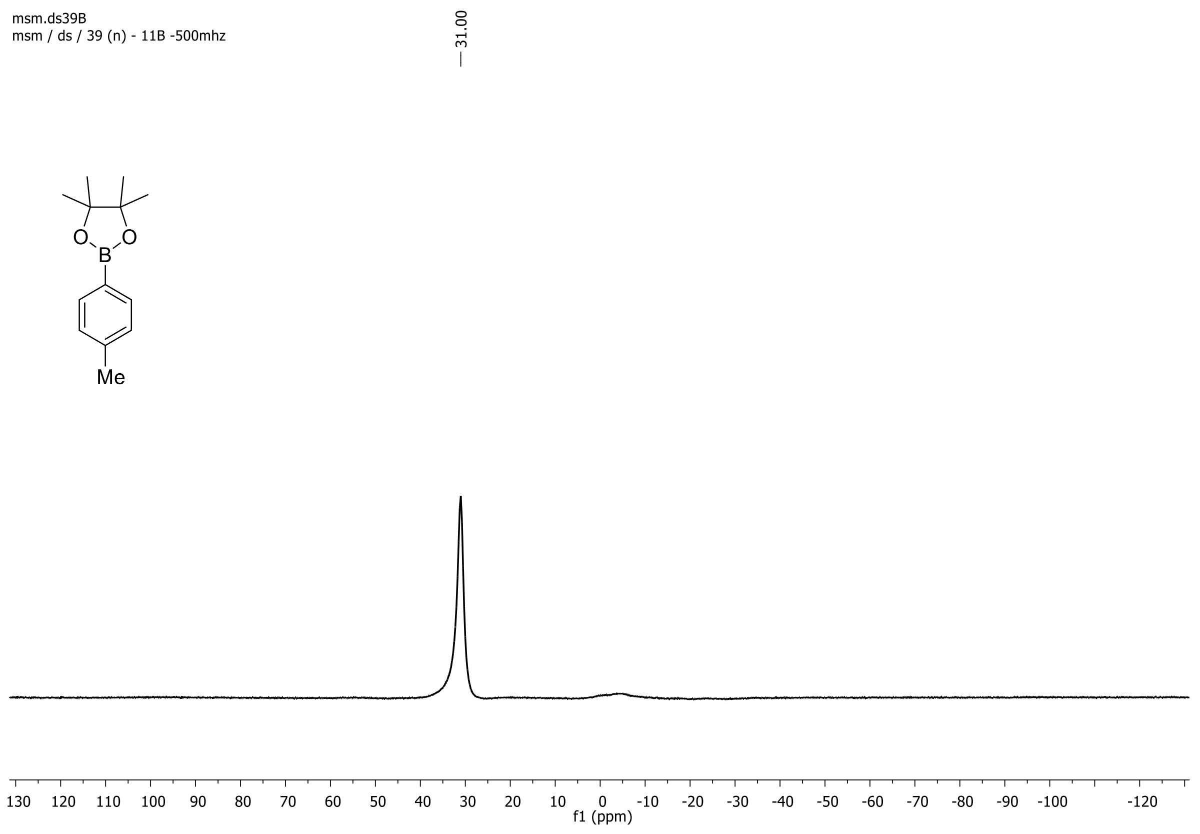

^{1}H (400 MHz) and ^{13}C (101 MHz) NMR of **2b** in $CDCl_3$

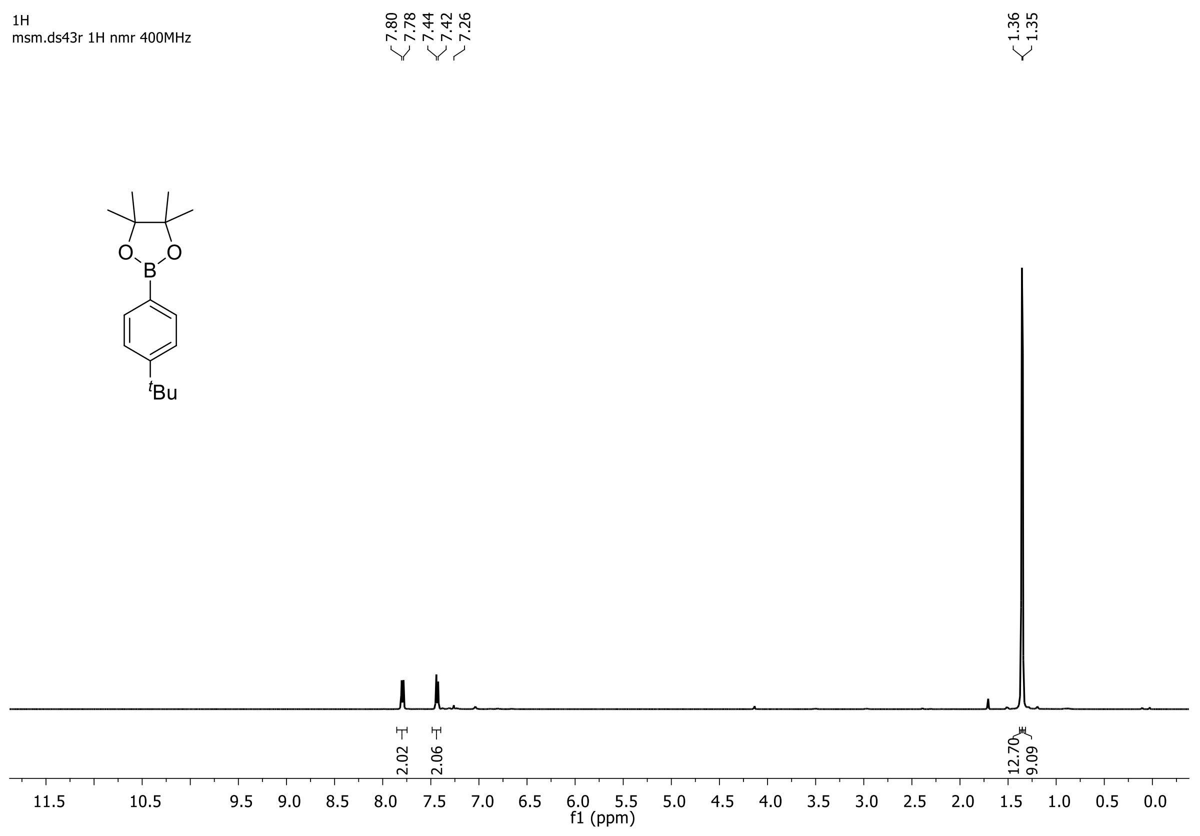

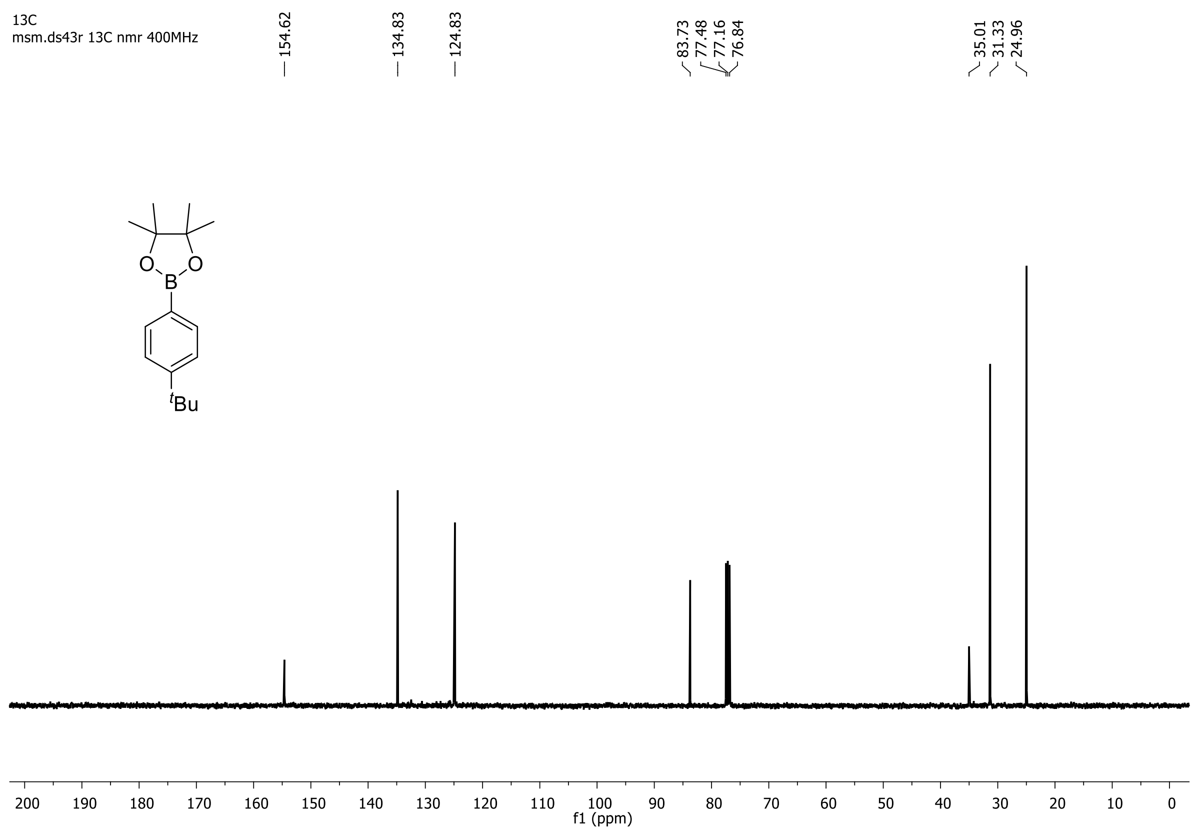

^{11}B(160 MHz) NMR of **2b** in $CDCl_3$

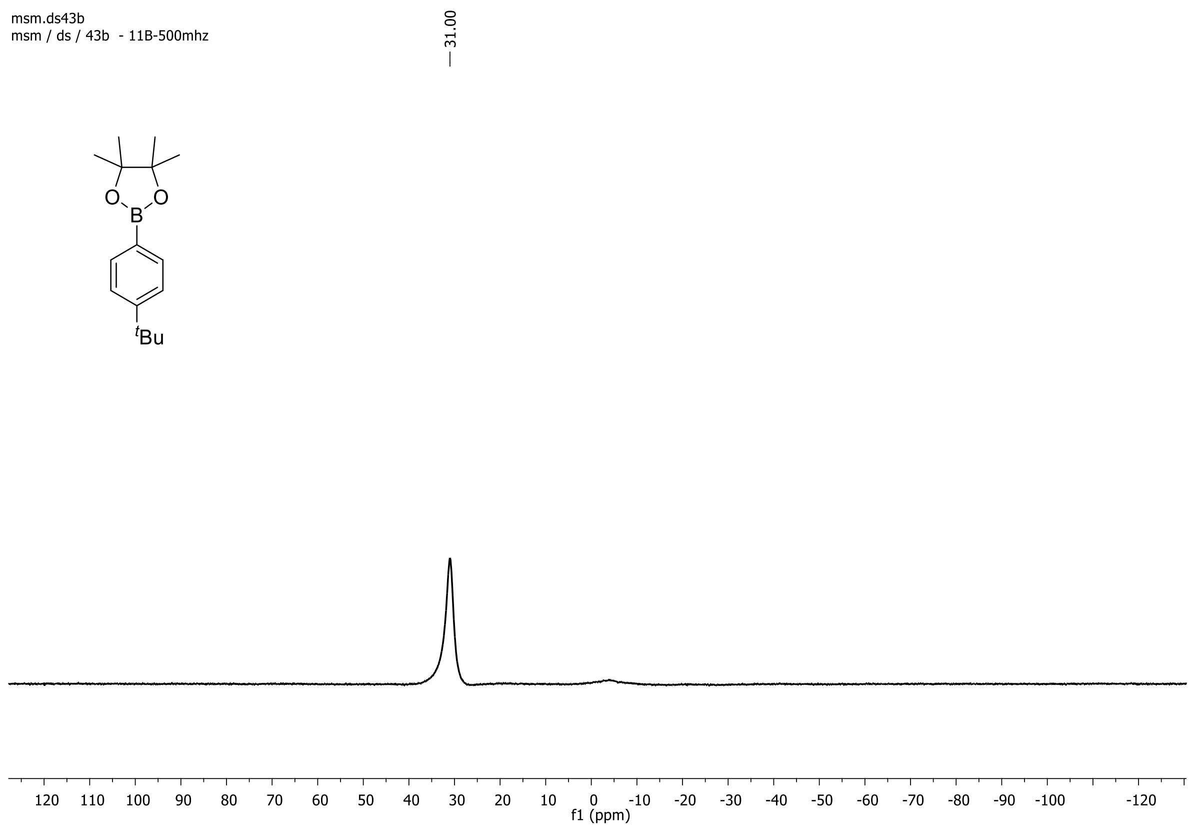

^{1}H (400 MHz) and ^{13}C (126 MHz) NMR of **2c** in $CDCl_3$

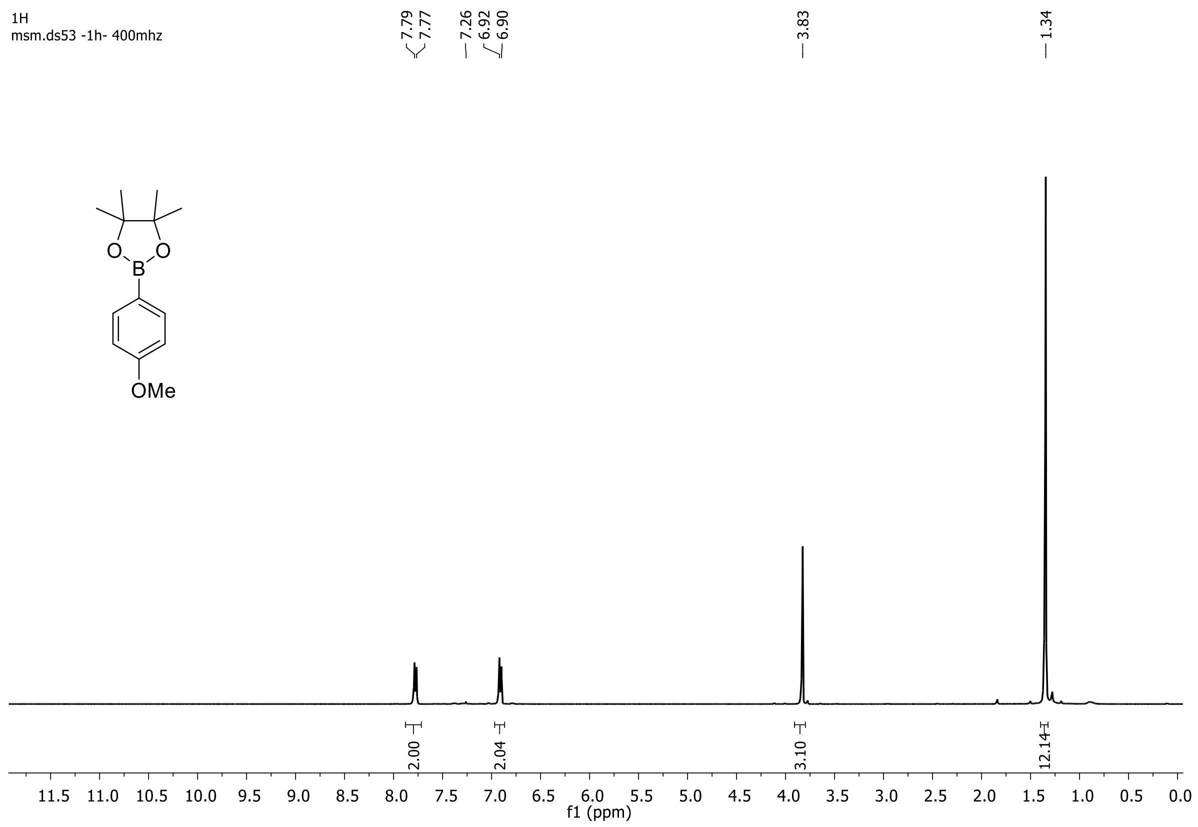

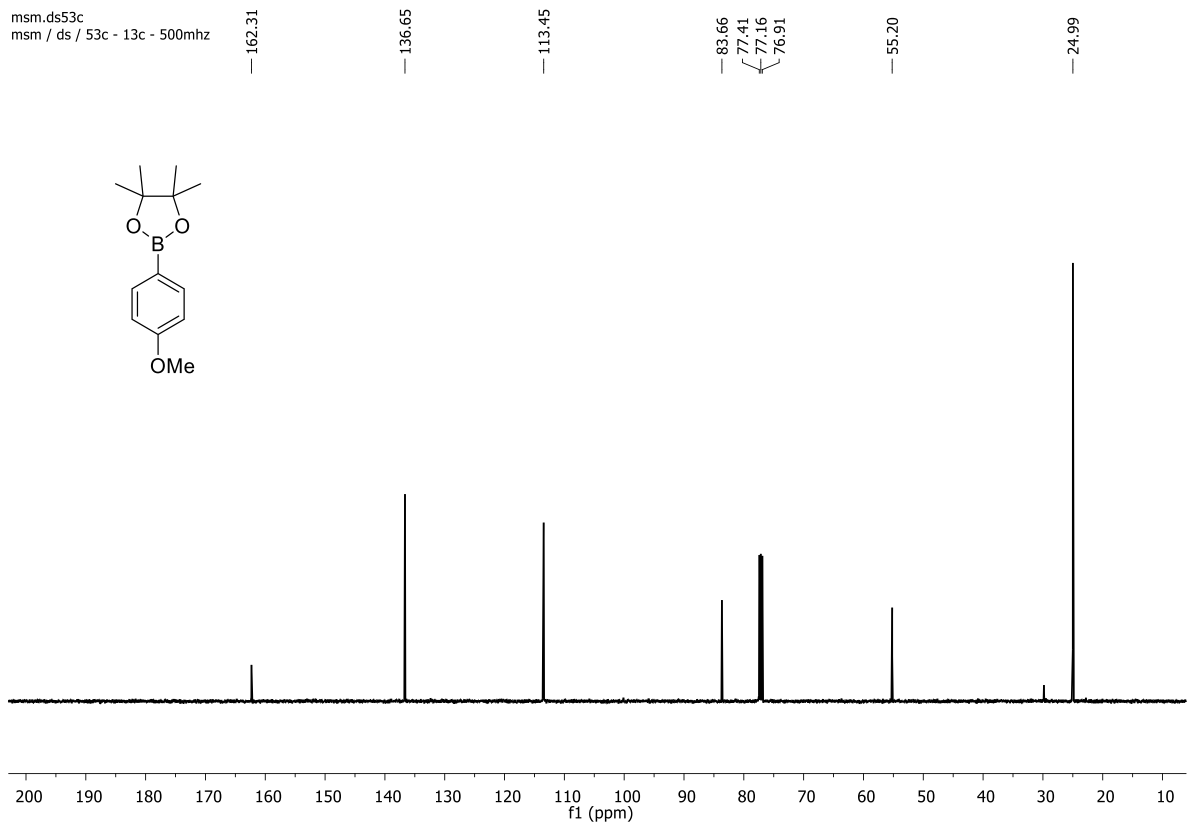

^{11}B (160 MHz) NMR of **2c** in $CDCl_3$

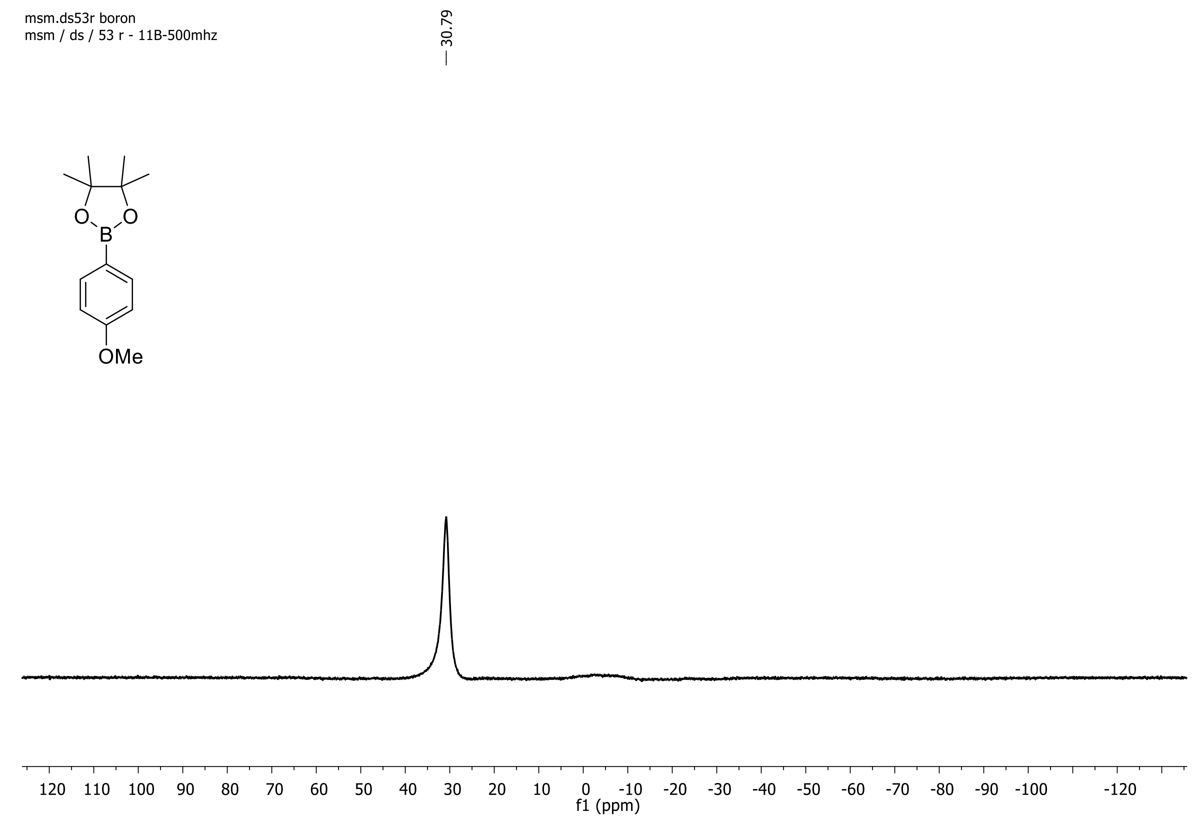

^{1}H (400 MHz) and ^{13}C (126 MHz) NMR of **2d** in $CDCl_3$

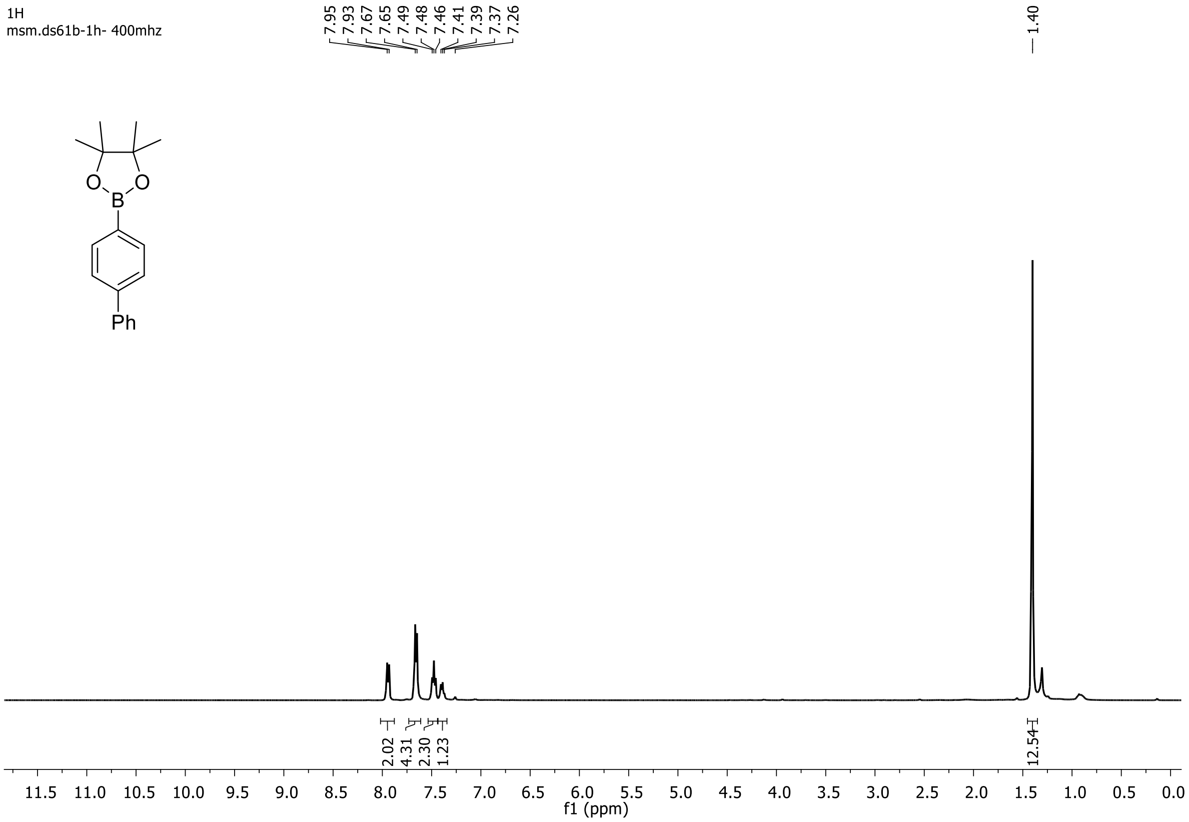

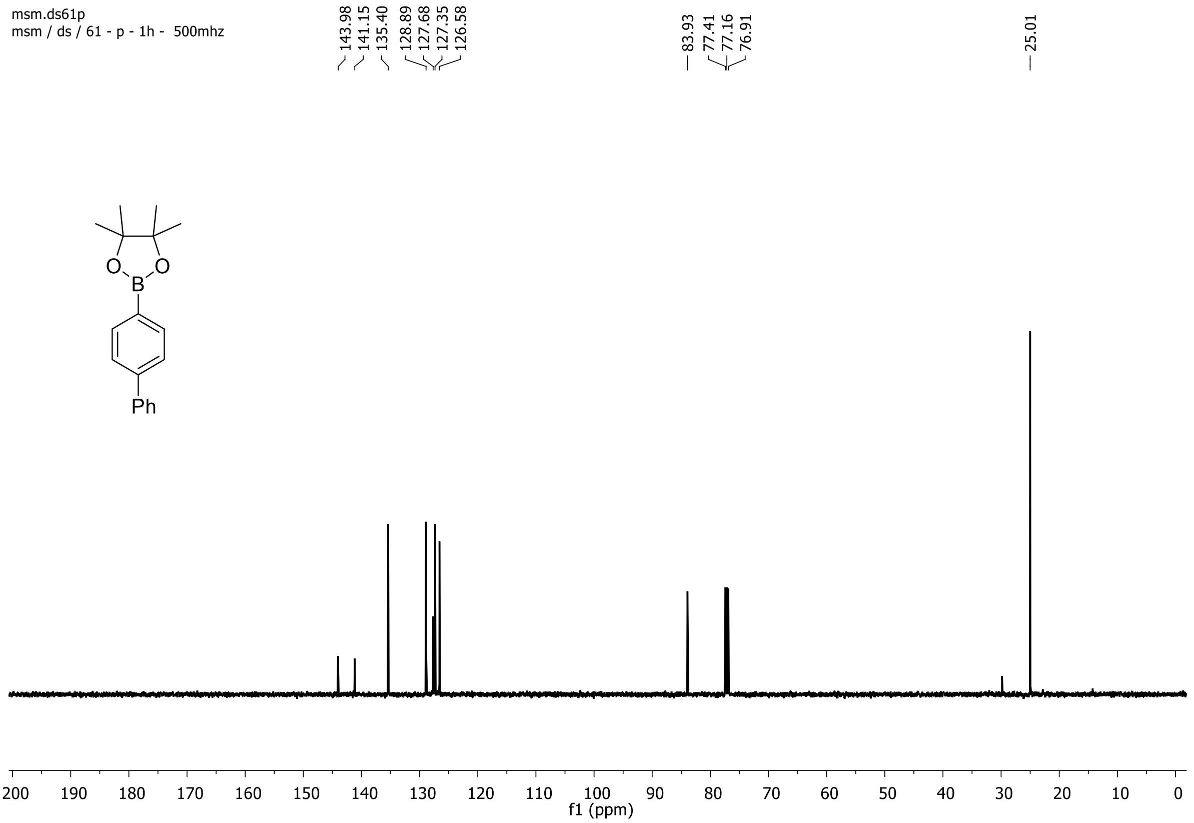

^{11}B (160 MHz) NMR of **2d** in $CDCl_3$

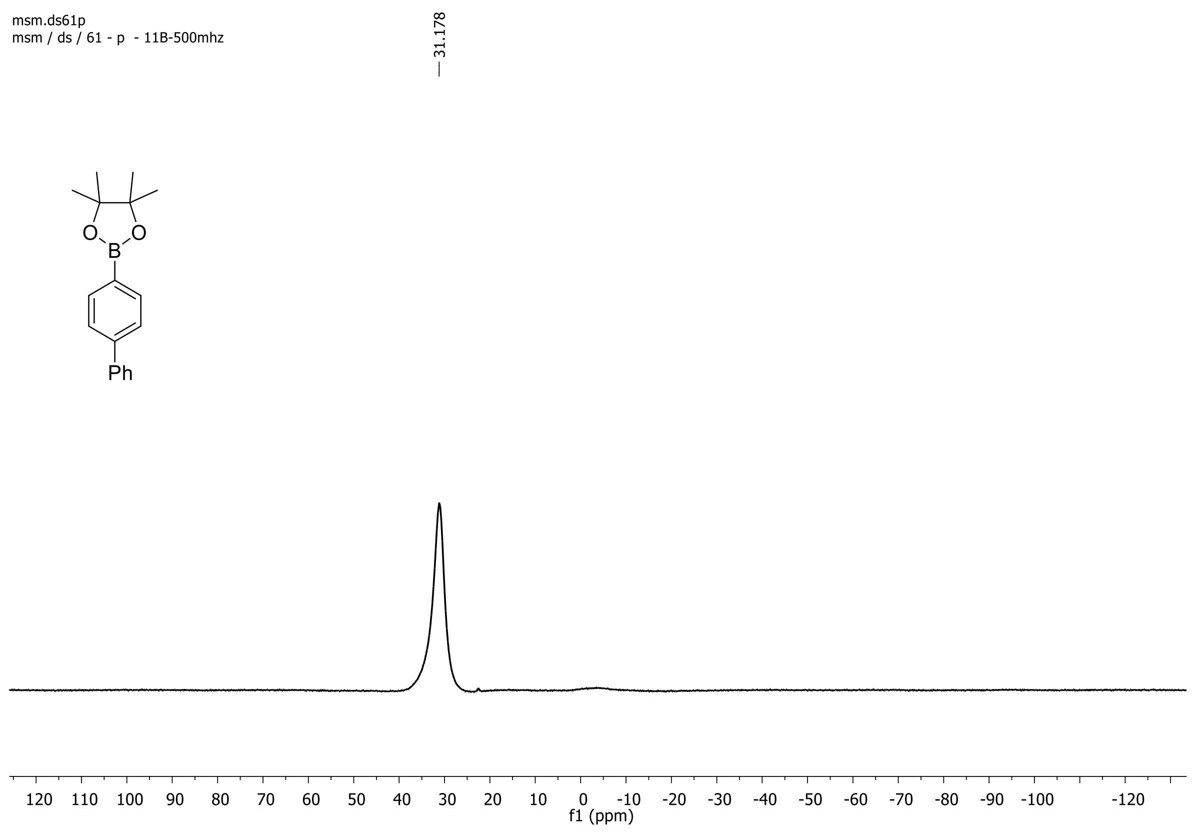

^{1}H (500 MHz) and ^{13}C (126 MHz) NMR of **2e** in $CDCl_3$

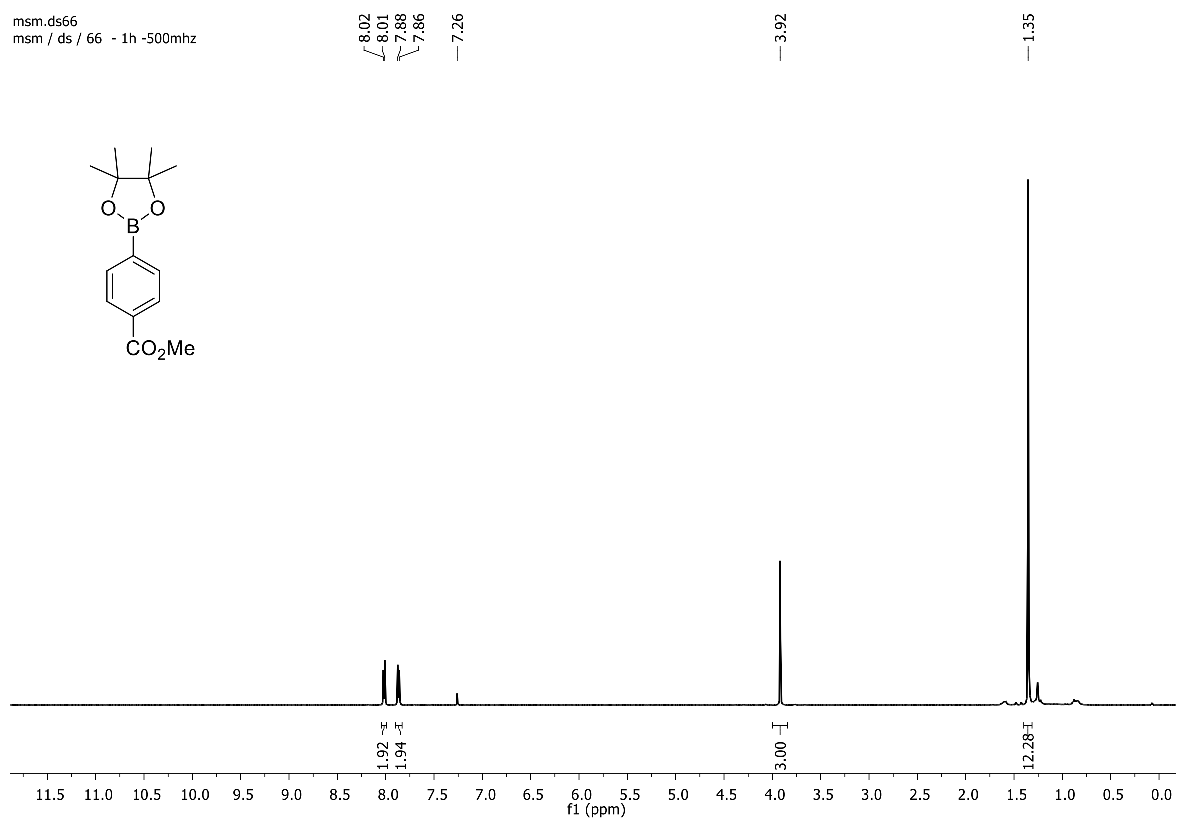

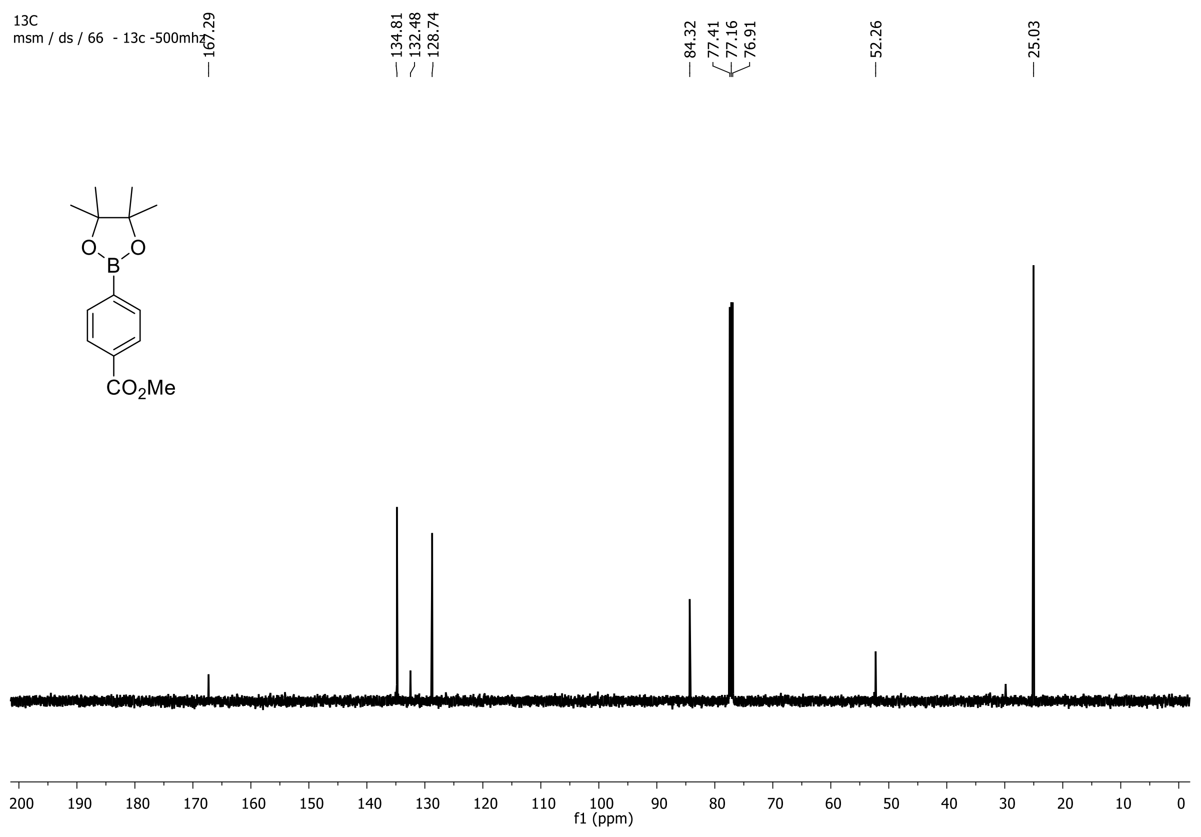

^{11}B (160 MHz) NMR of **2e** in $CDCl_3$

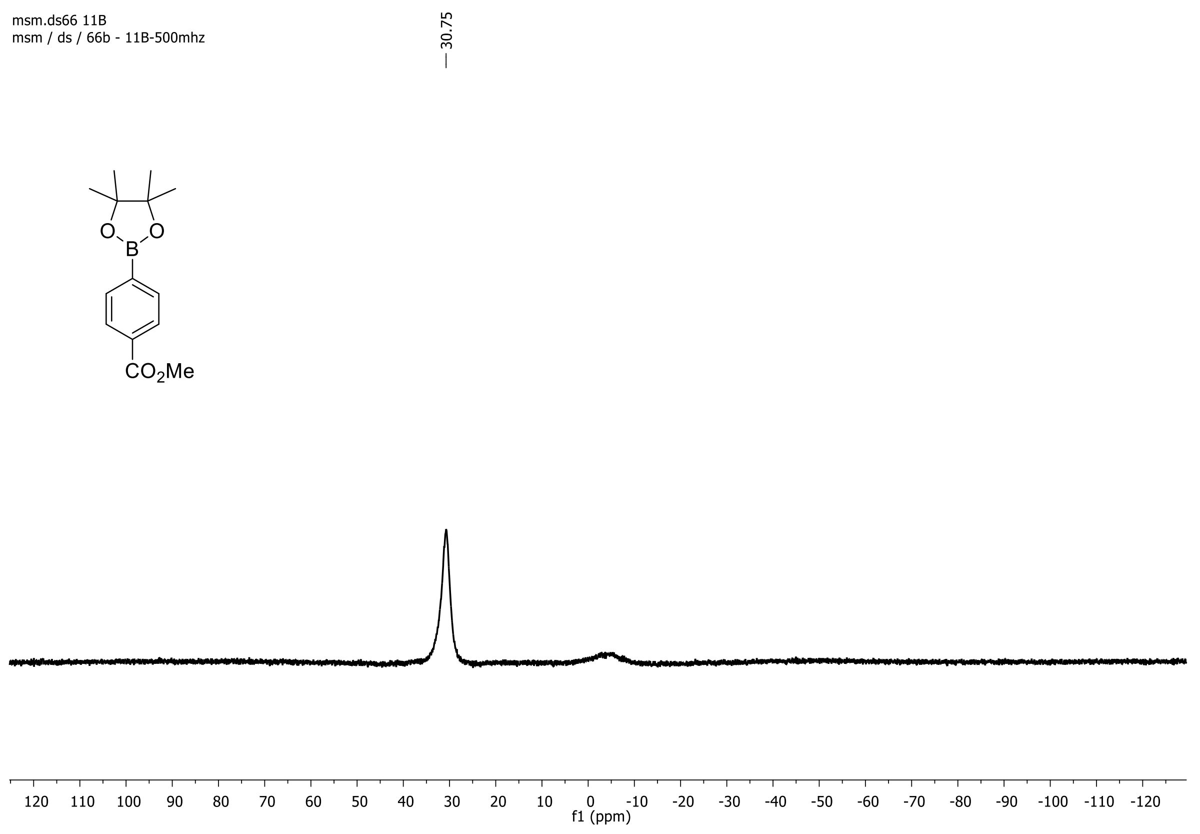

^{1}H (500 MHz) and ^{13}C (126 MHz) NMR of **2f** in $CDCl_3$

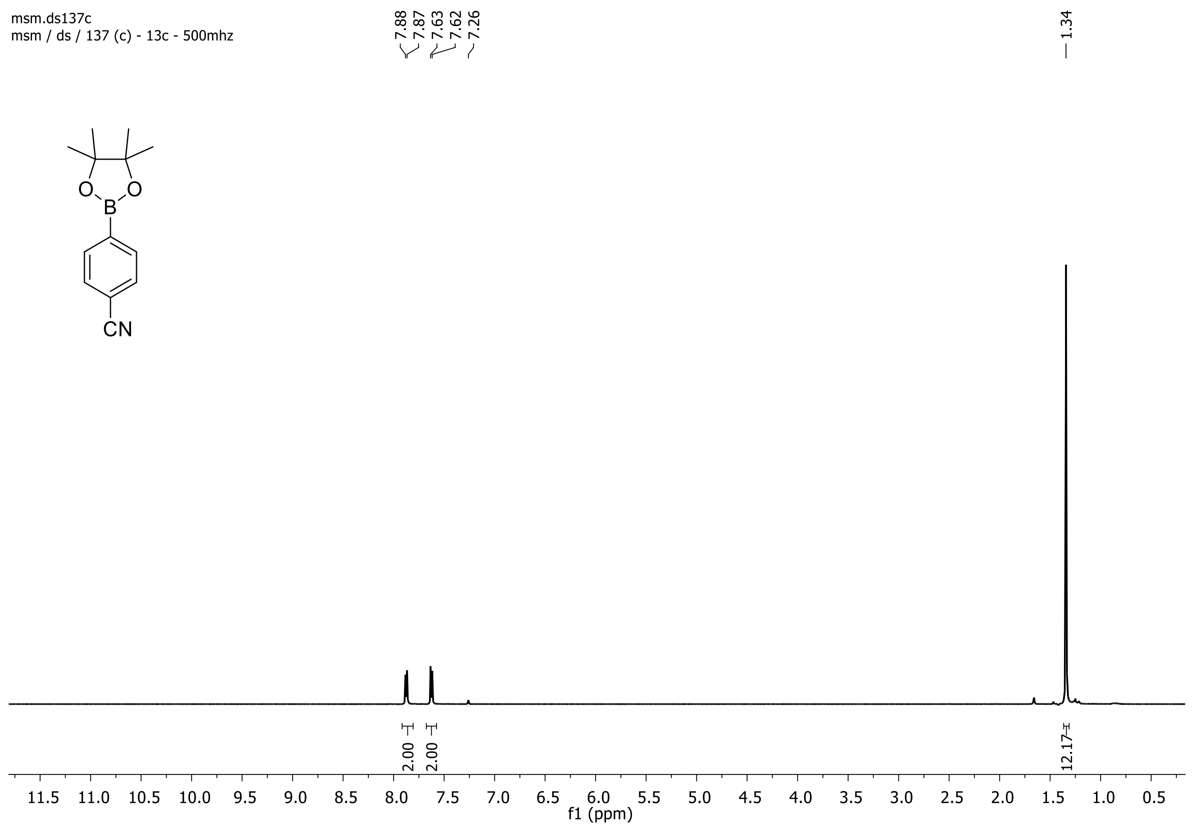

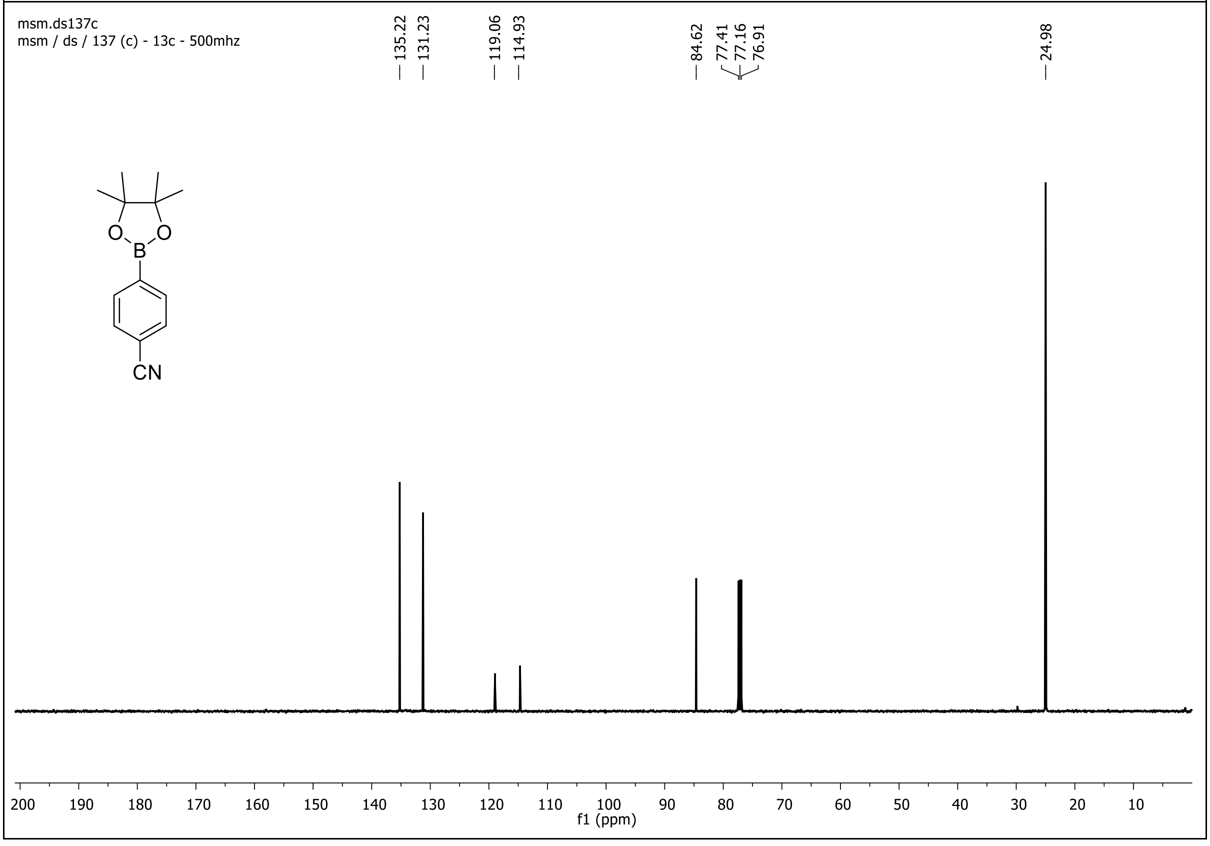

^{11}B (160 MHz) NMR of **2f** in $CDCl_3$

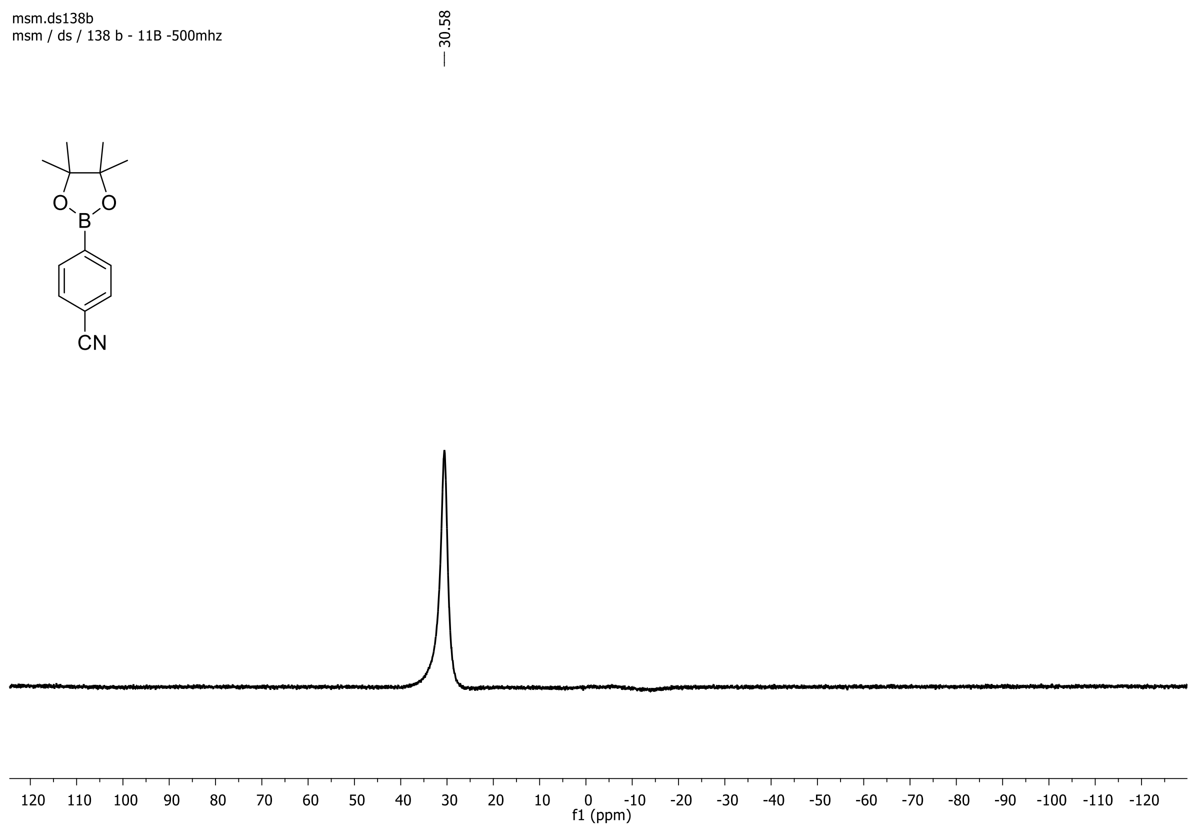

^{1}H (400 MHz) and ^{13}C (126 MHz) NMR of **2g** in $CDCl_3$

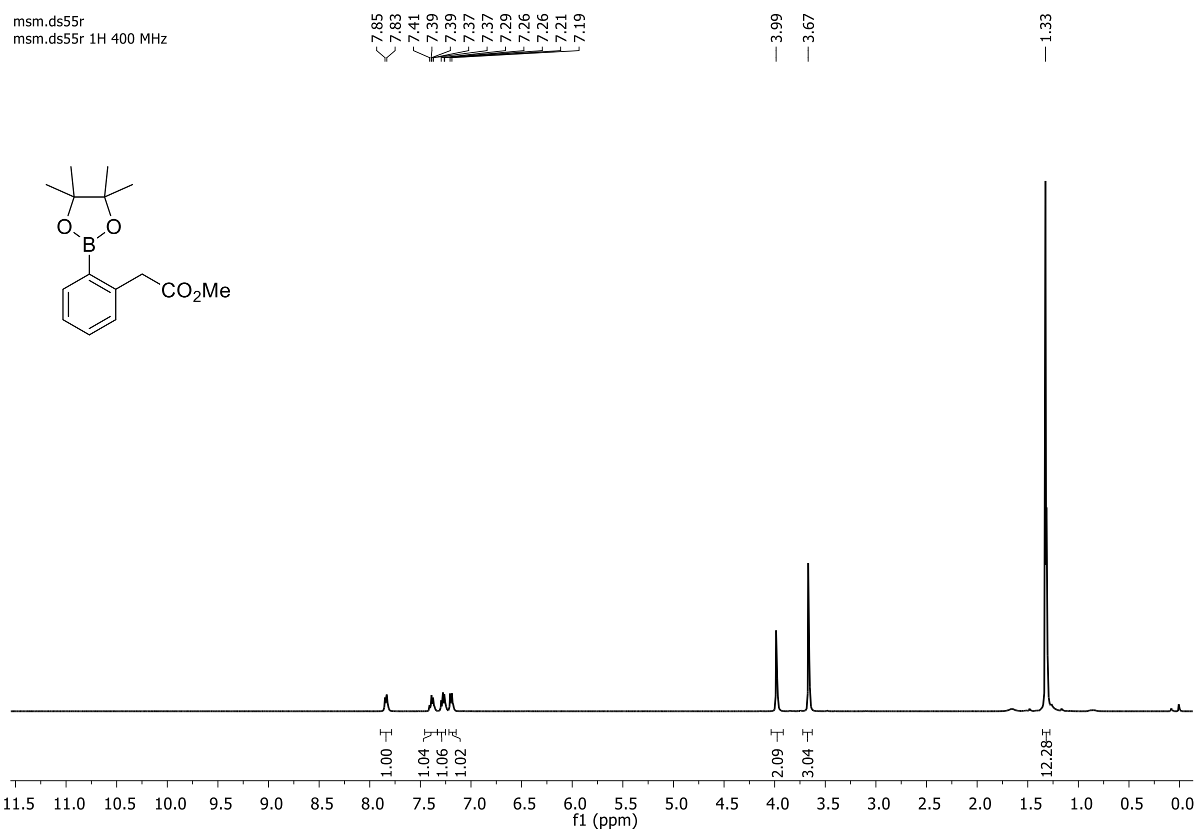

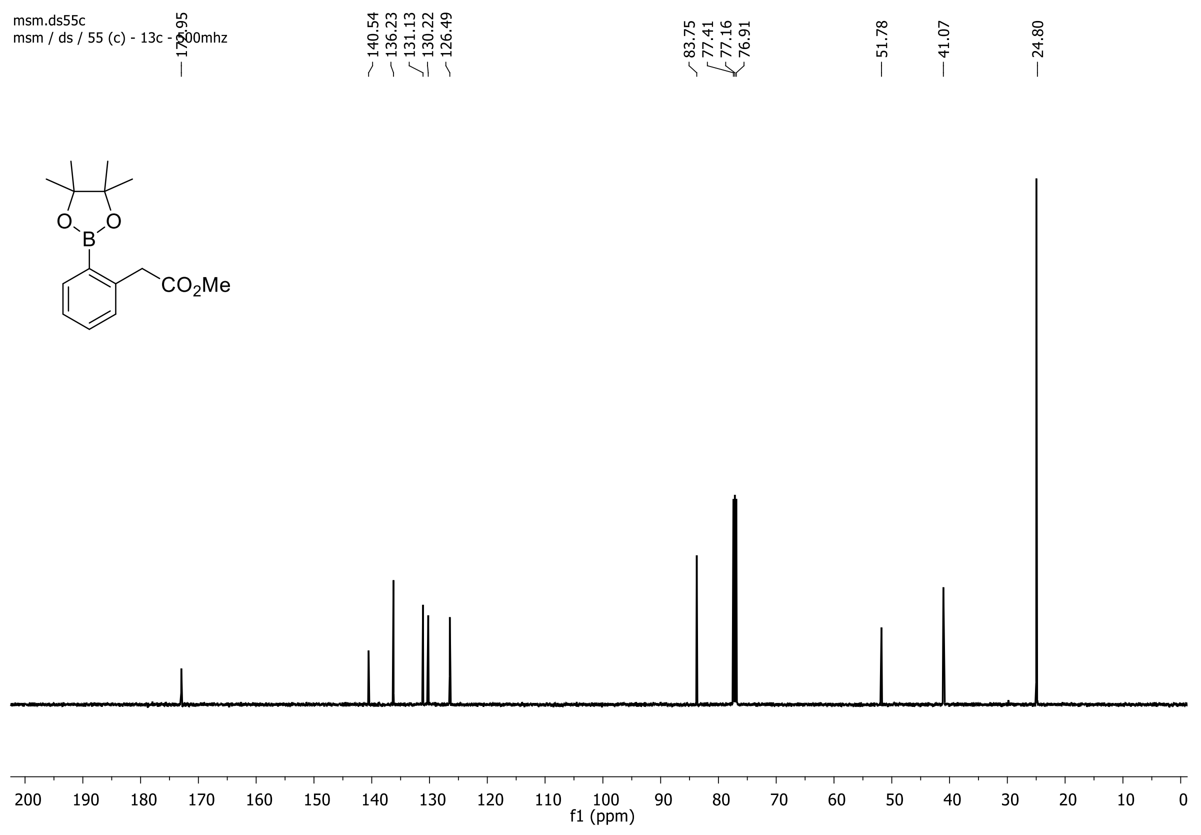

^{11}B (160 MHz) NMR of **2g** in $CDCl_3$

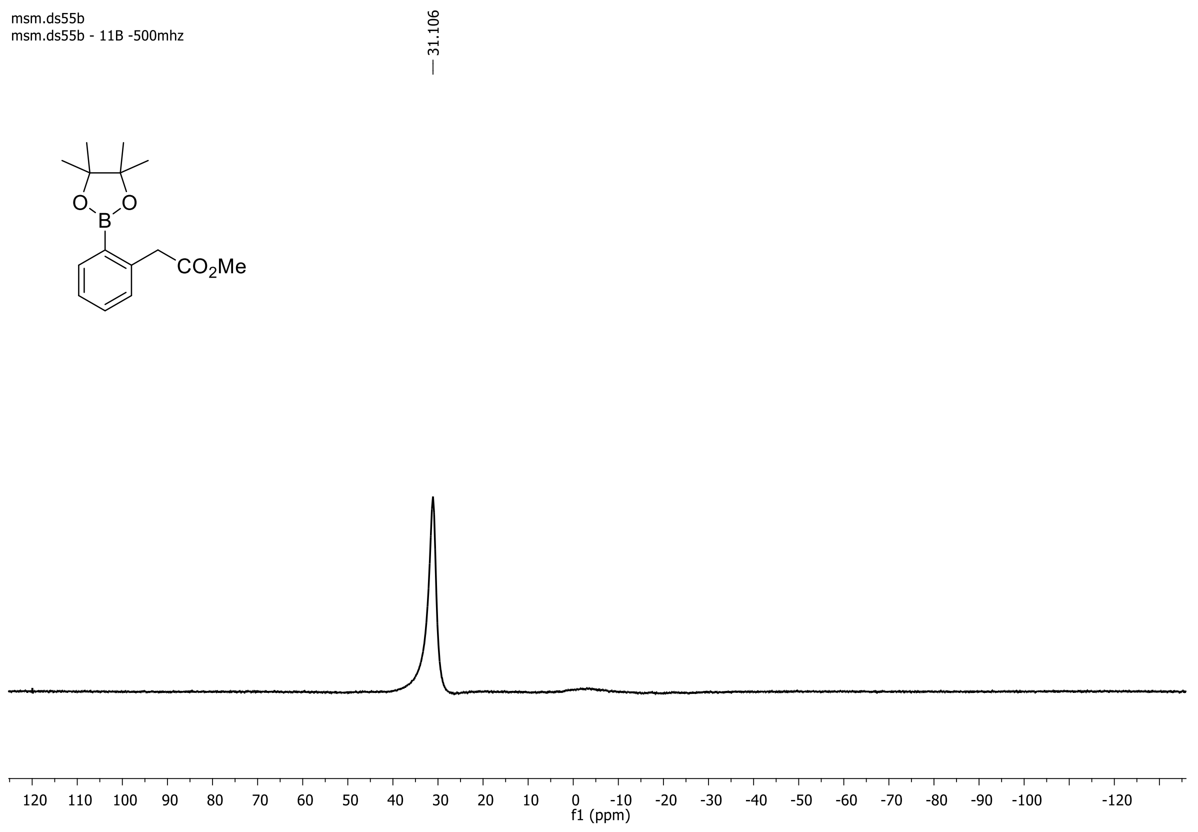

^{1}H (400 MHz) and ^{13}C (101 MHz) NMR of **2h** in $CDCl_3$

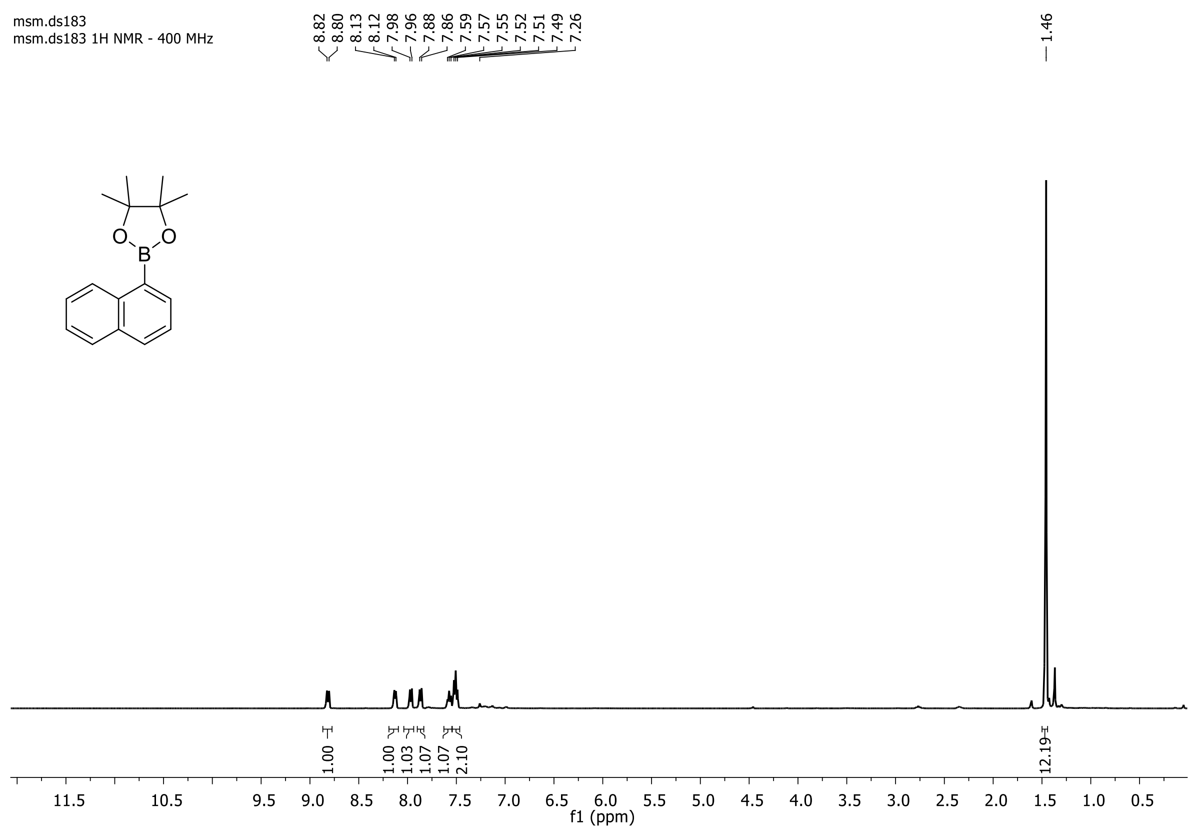

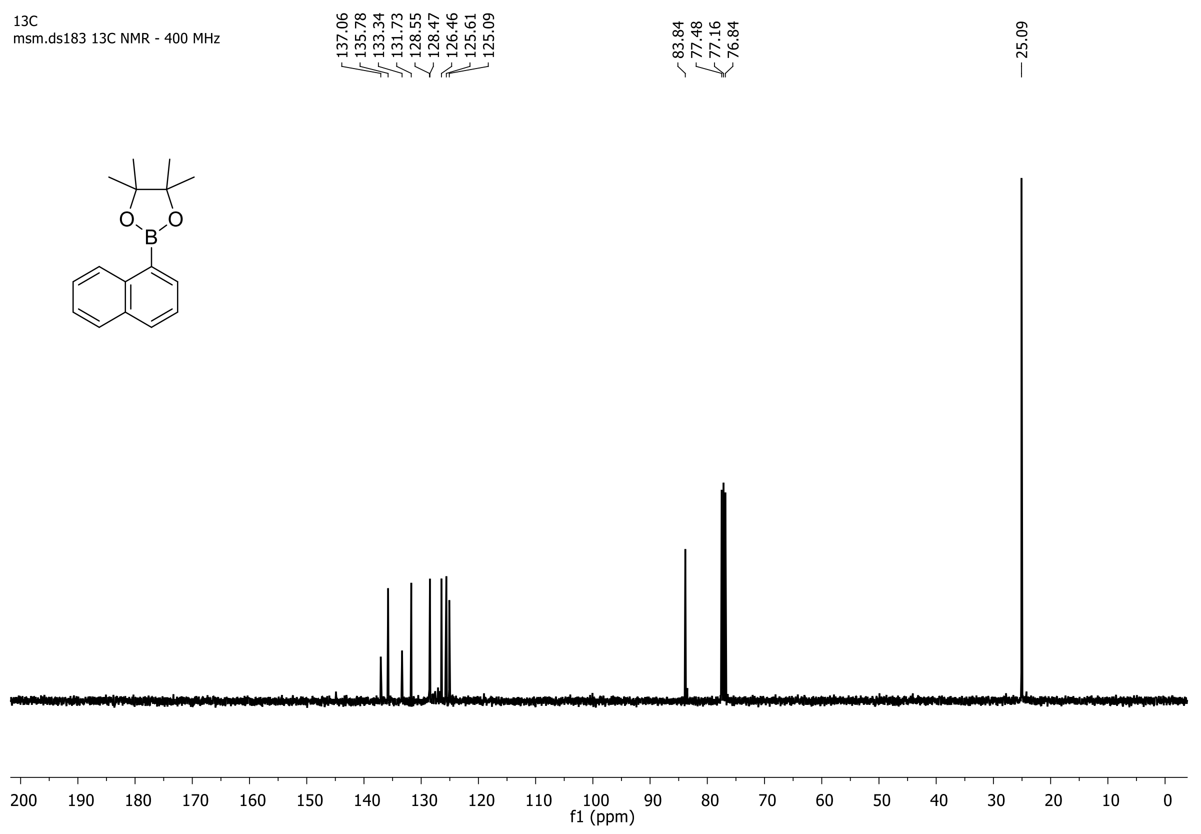

^{11}B (160 MHz) NMR of **2h** in $CDCl_3$

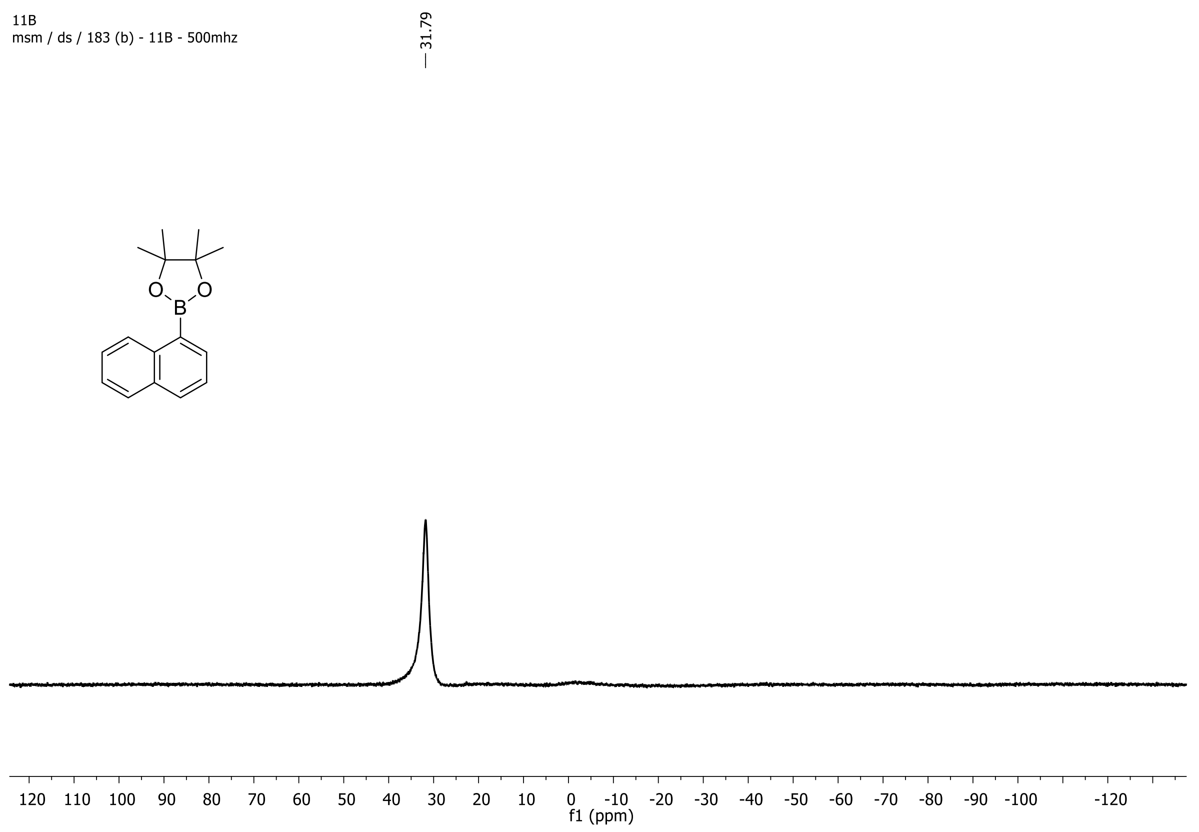

^{1}H (500 MHz) and ^{13}C (126 MHz) NMR of **2i** in $CDCl_3$

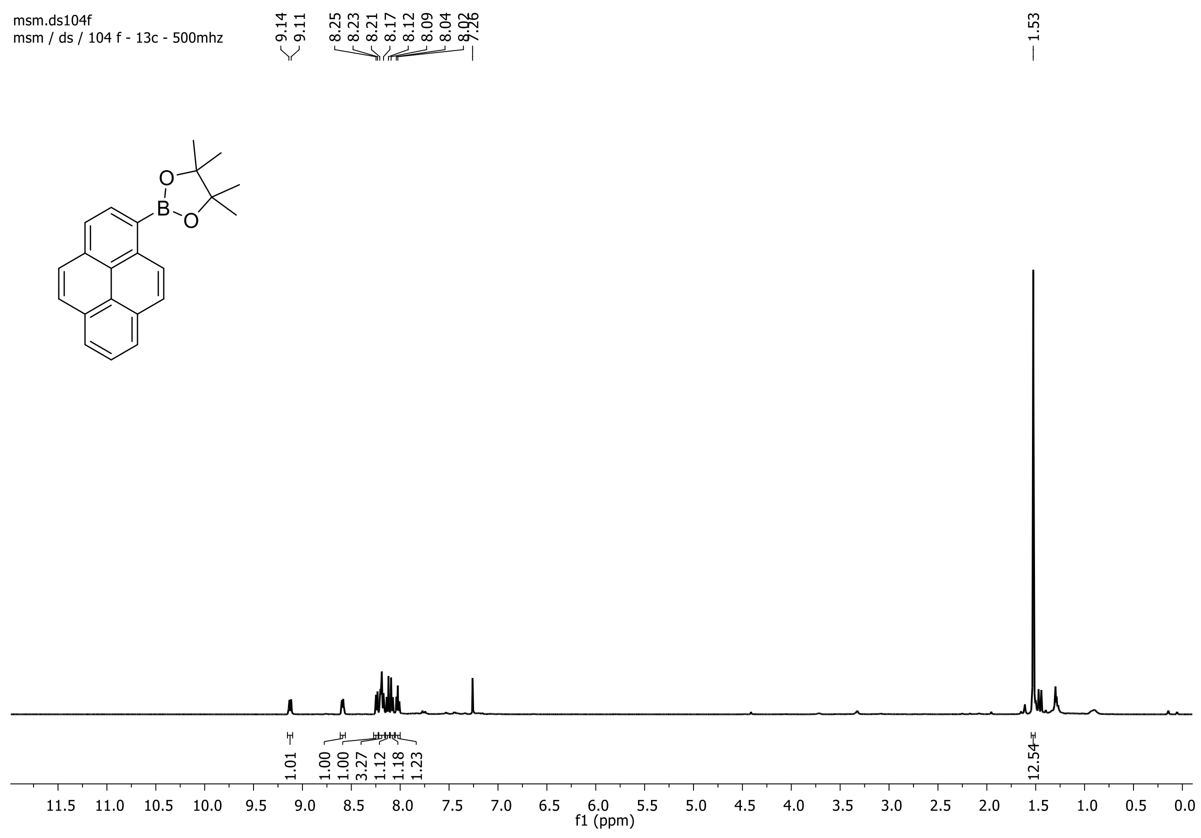

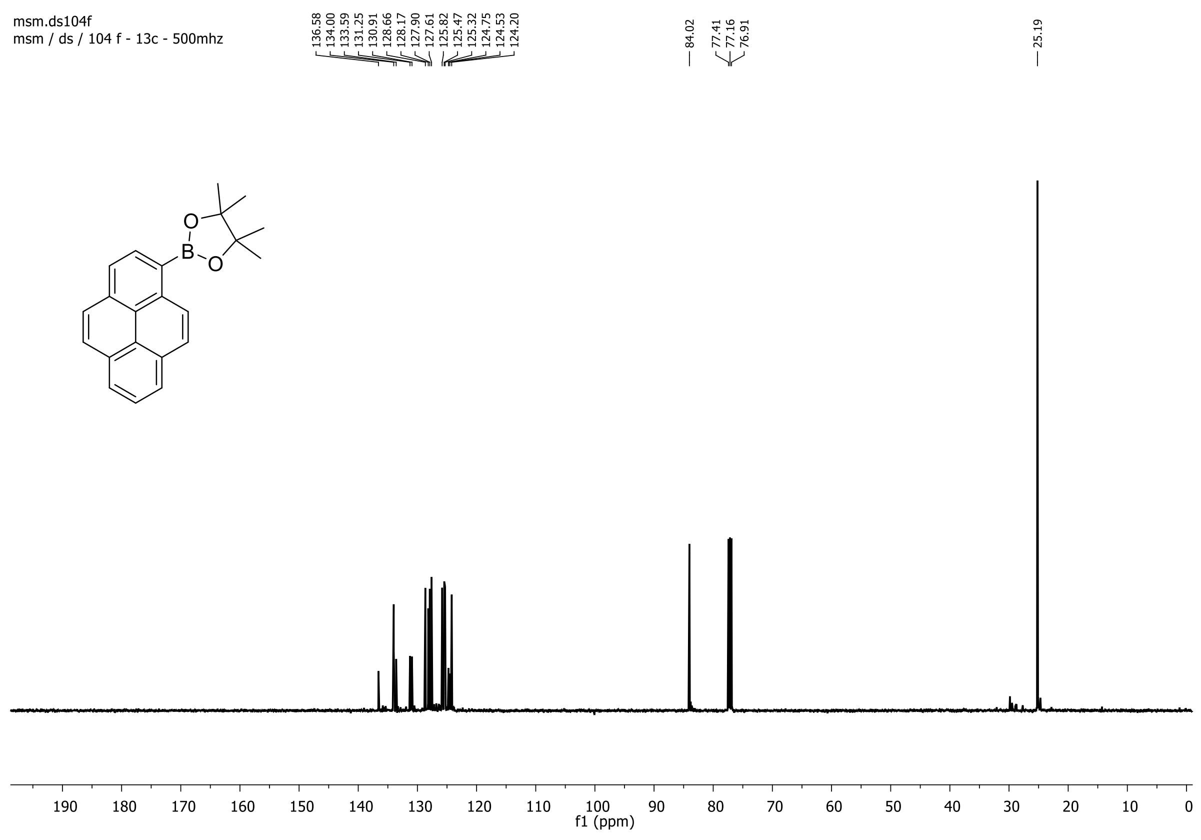

^{11}B (160 MHz) NMR of **2i** in $CDCl_3$

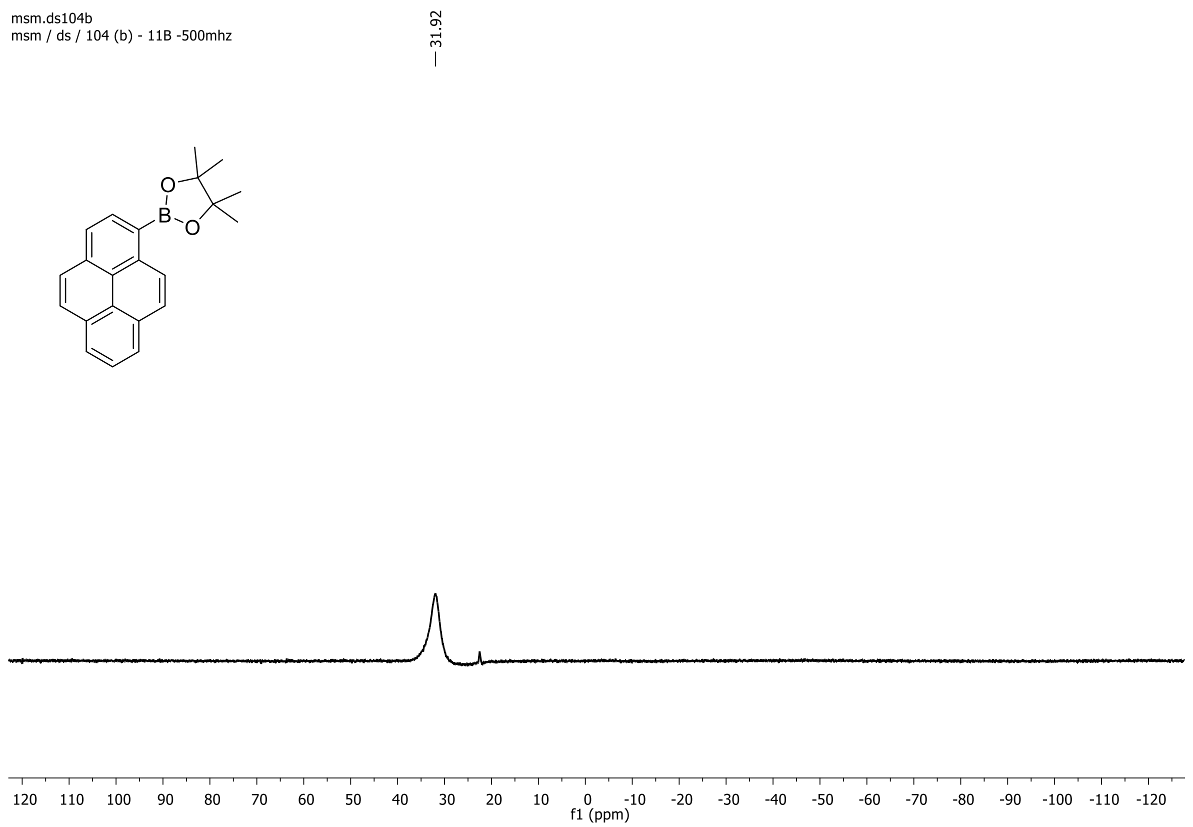

^{1}H (400 MHz) and ^{13}C (101 MHz) NMR of **2j** in $CDCl_3$

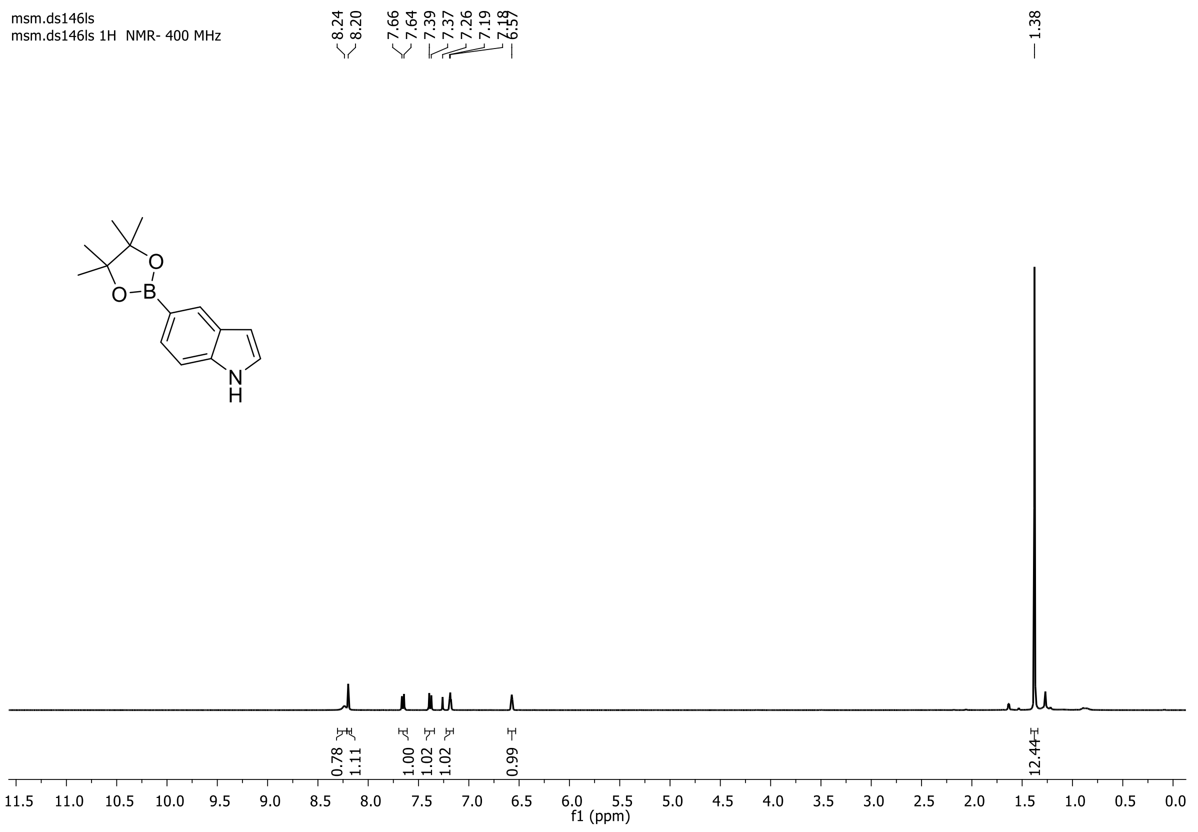

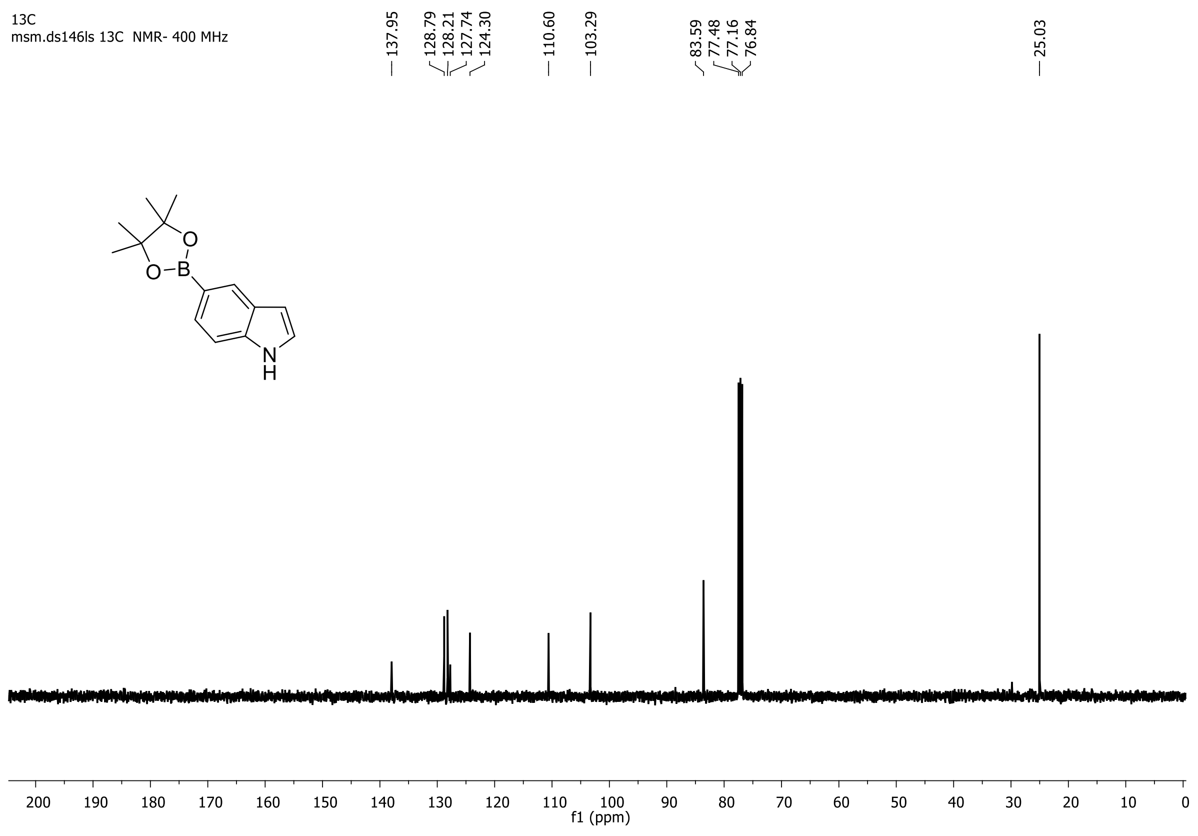

^{11}B (160 MHz) NMR of **2j** in $CDCl_3$

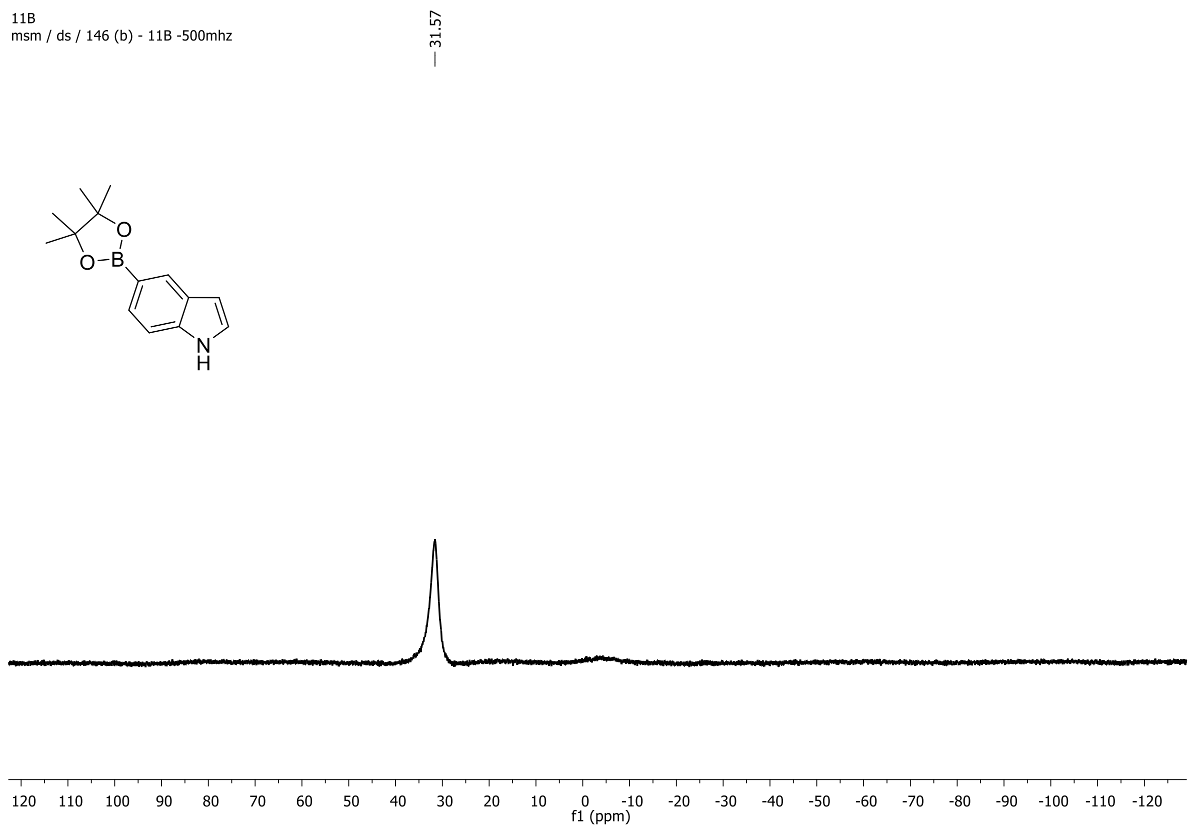

^{1}H (400 MHz) and ^{13}C (126 MHz) NMR of **2k** in $CDCl_3$

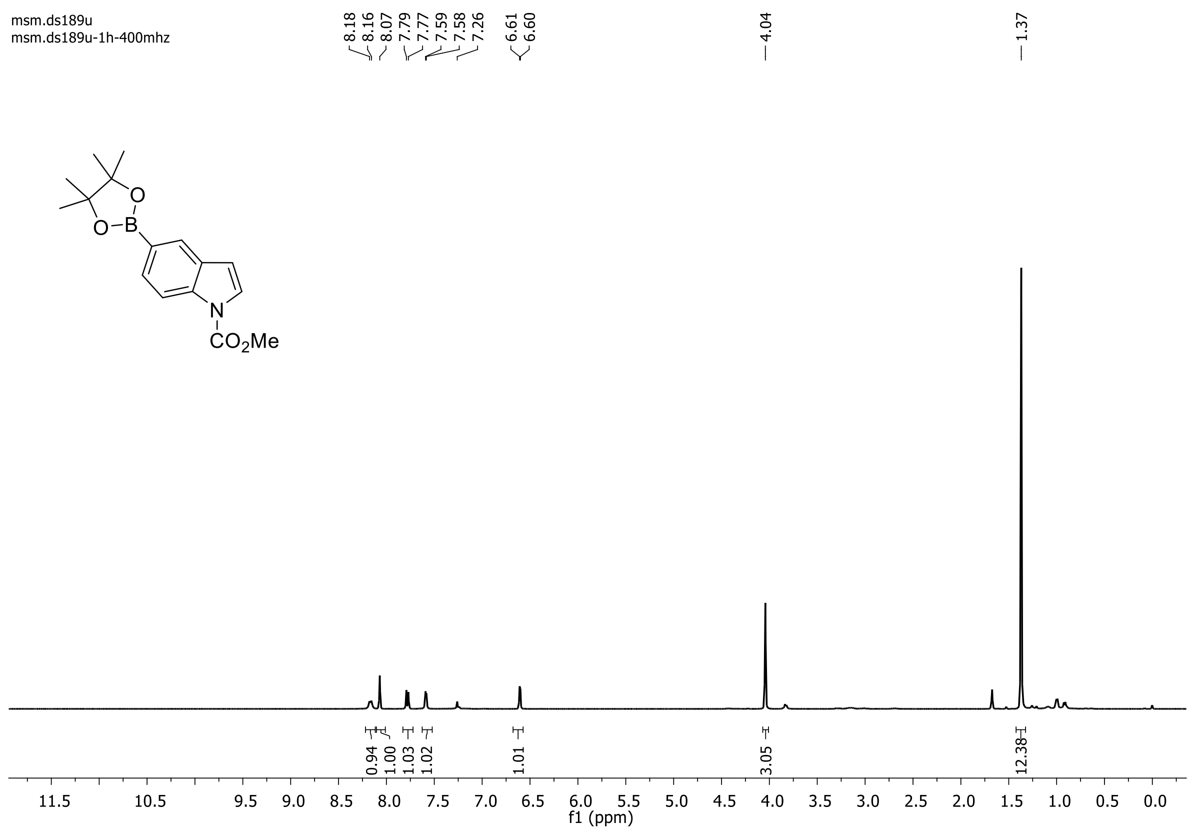

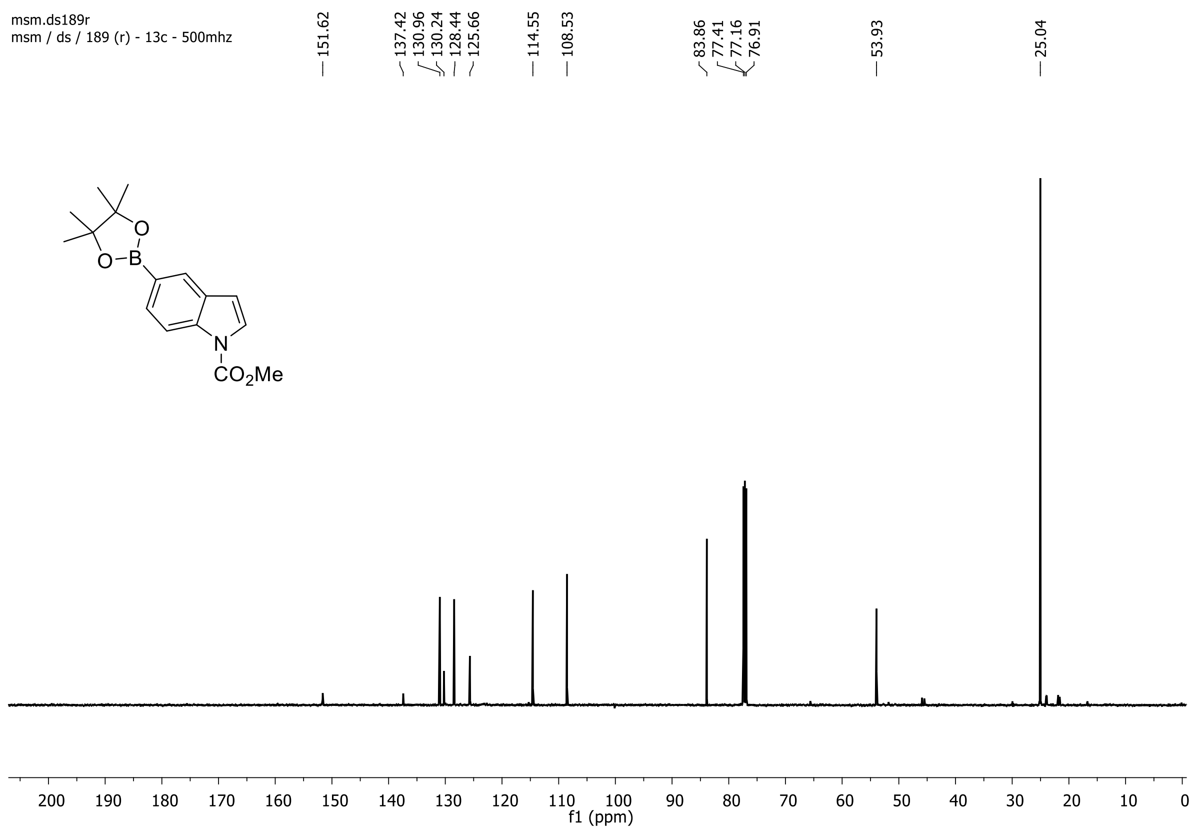

^{11}B (160 MHz) NMR of **2k** in $CDCl_3$

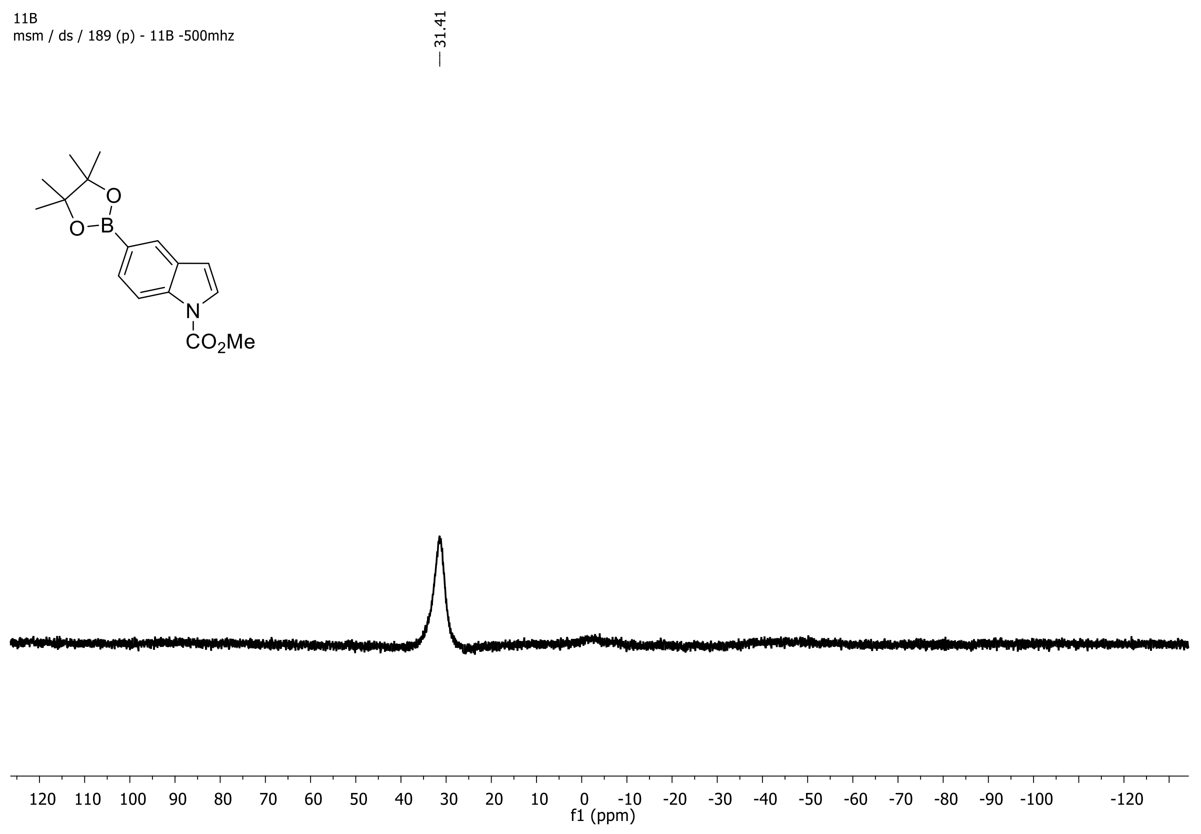

^{1}H (400 MHz) and ^{13}C (101 MHz) NMR of **2l** in $CDCl_3$

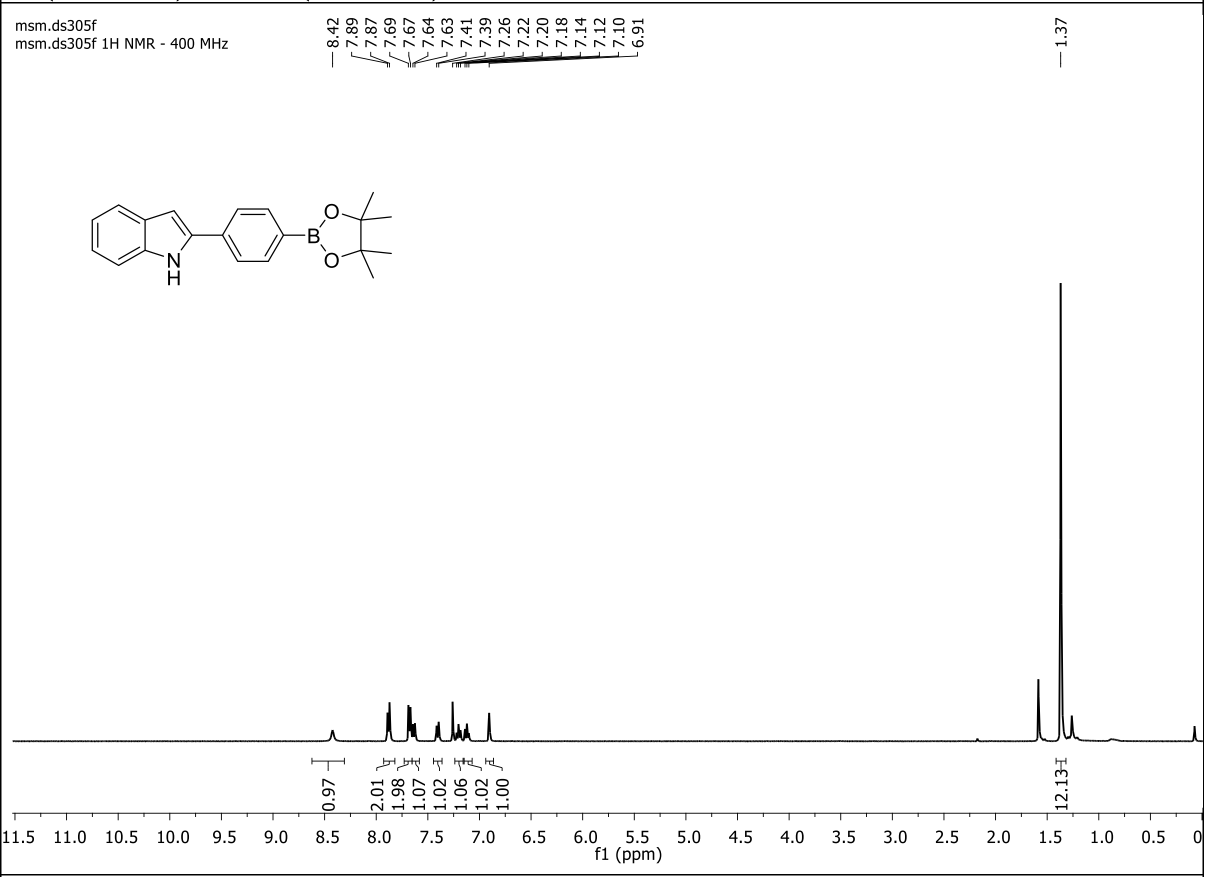

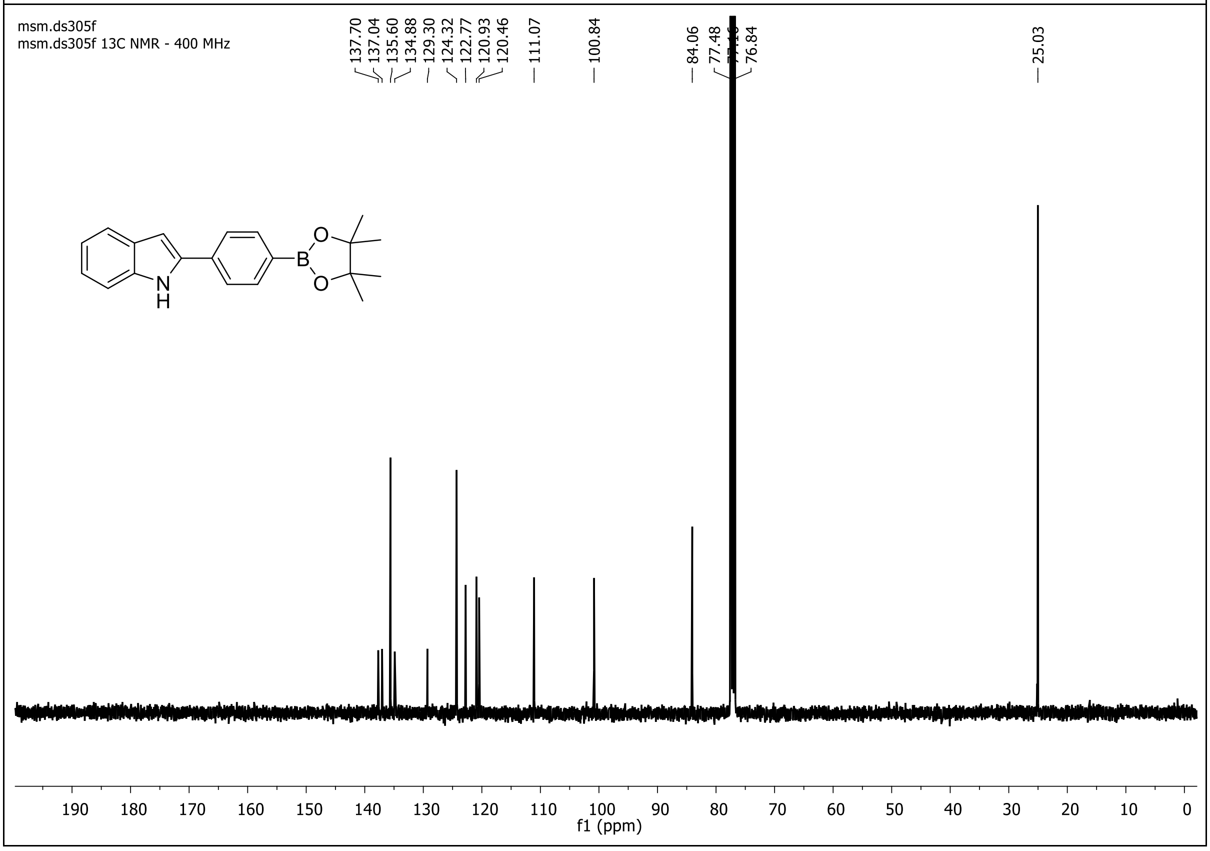

^{11}B (160 MHz) NMR of **2l** in $CDCl_3$

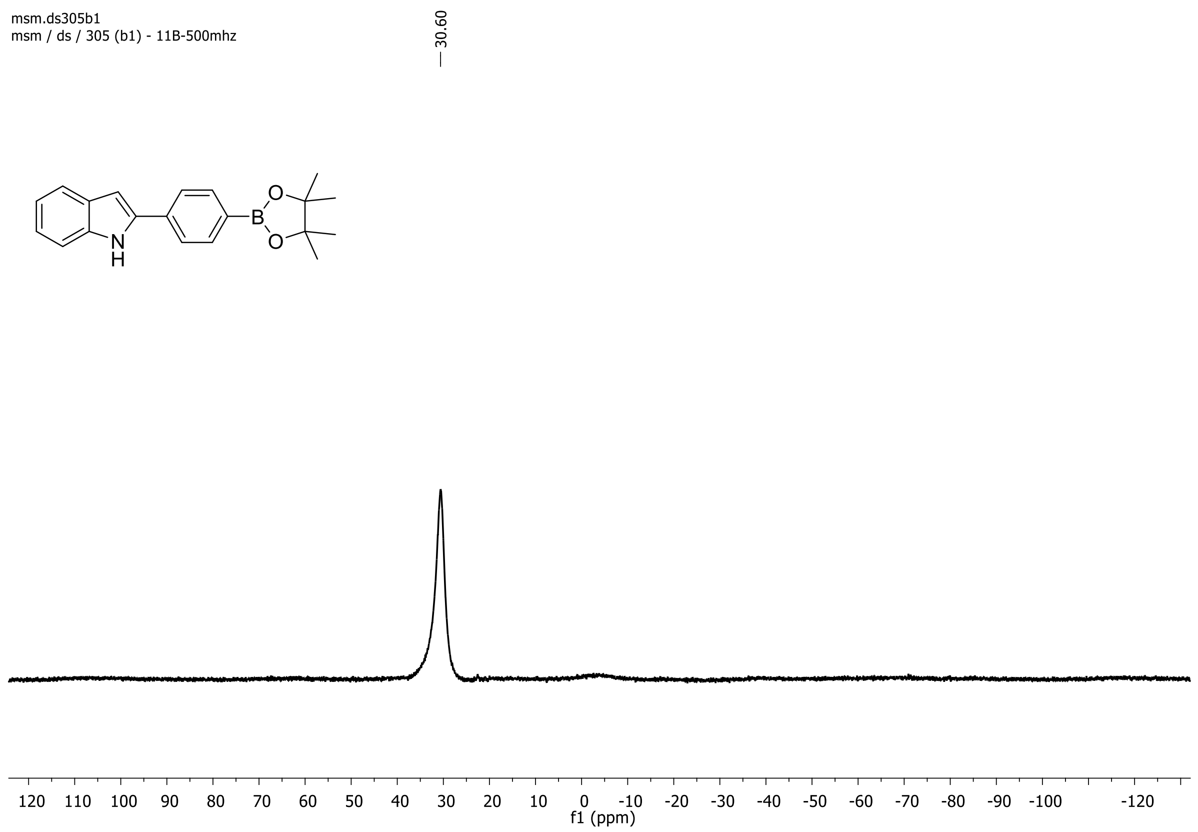

^{1}H (400 MHz) and ^{13}C (126 MHz) NMR of **2m** in $CDCl_3$

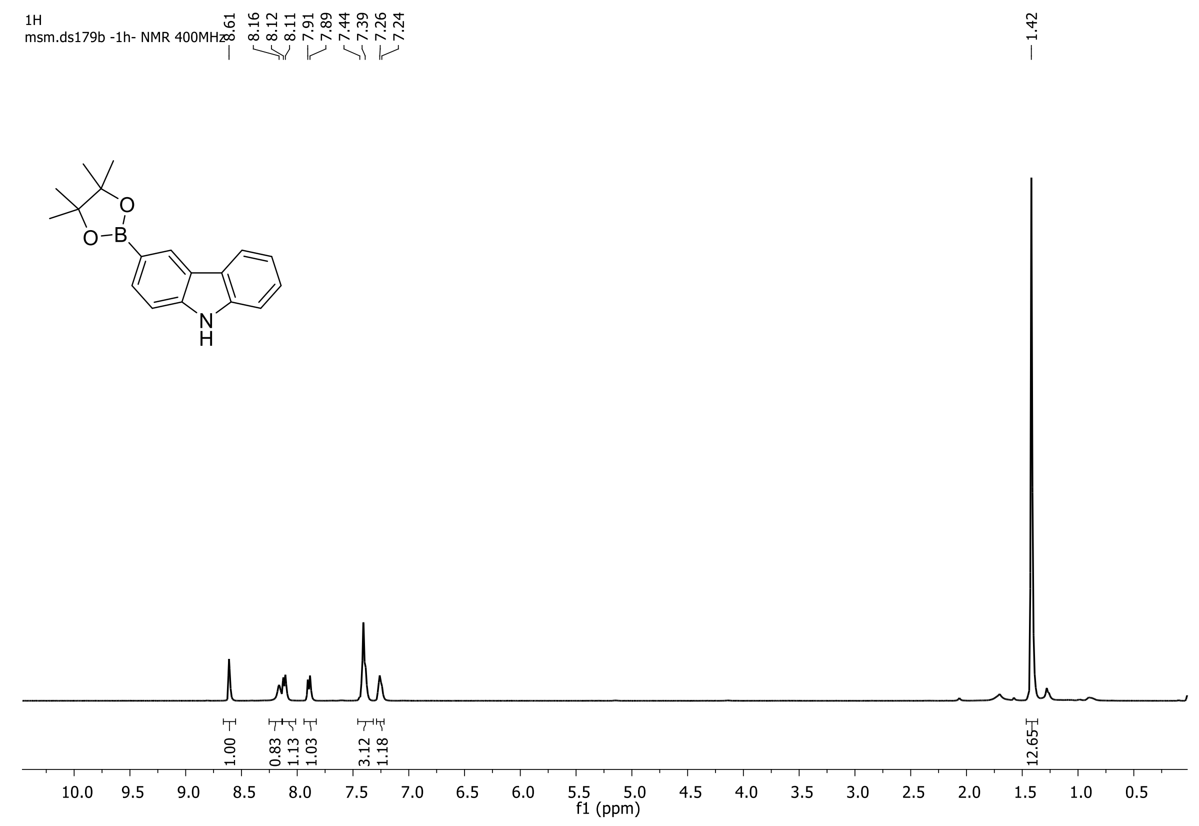

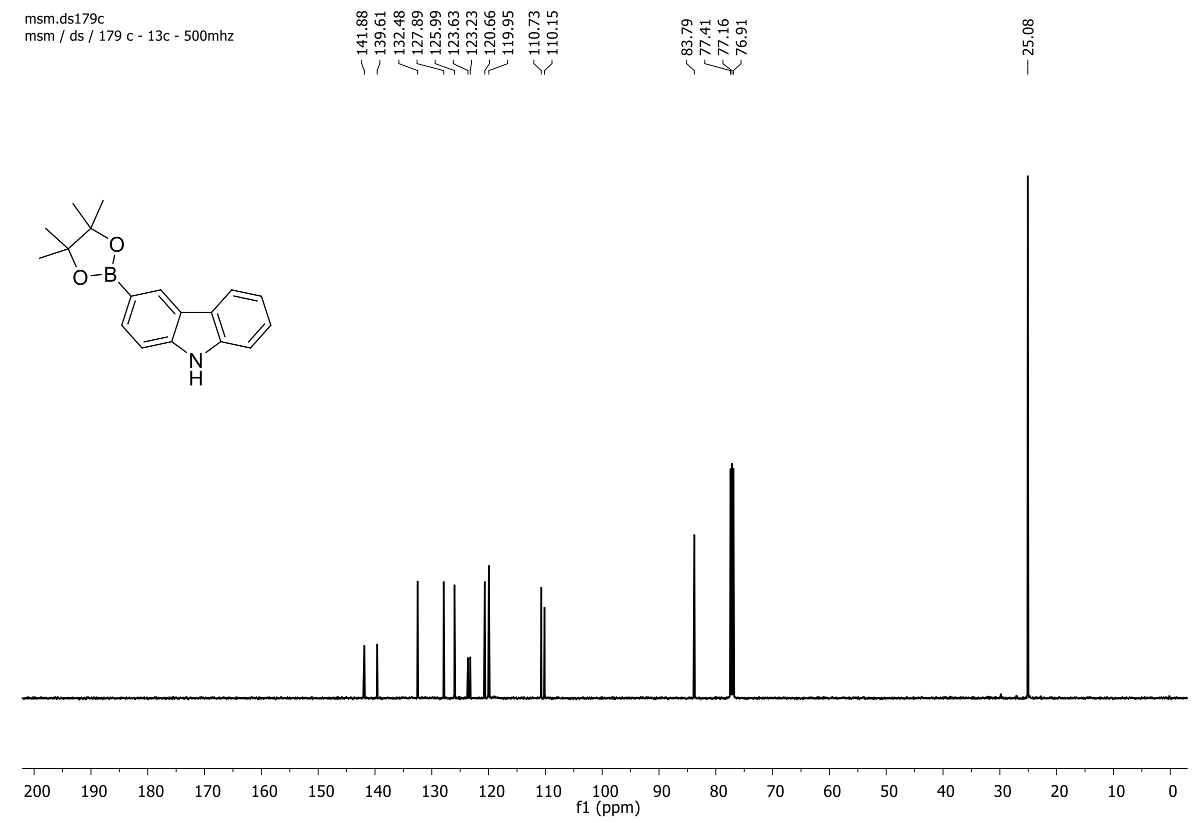

^{11}B (160 MHz) NMR of **2m** in $CDCl_3$

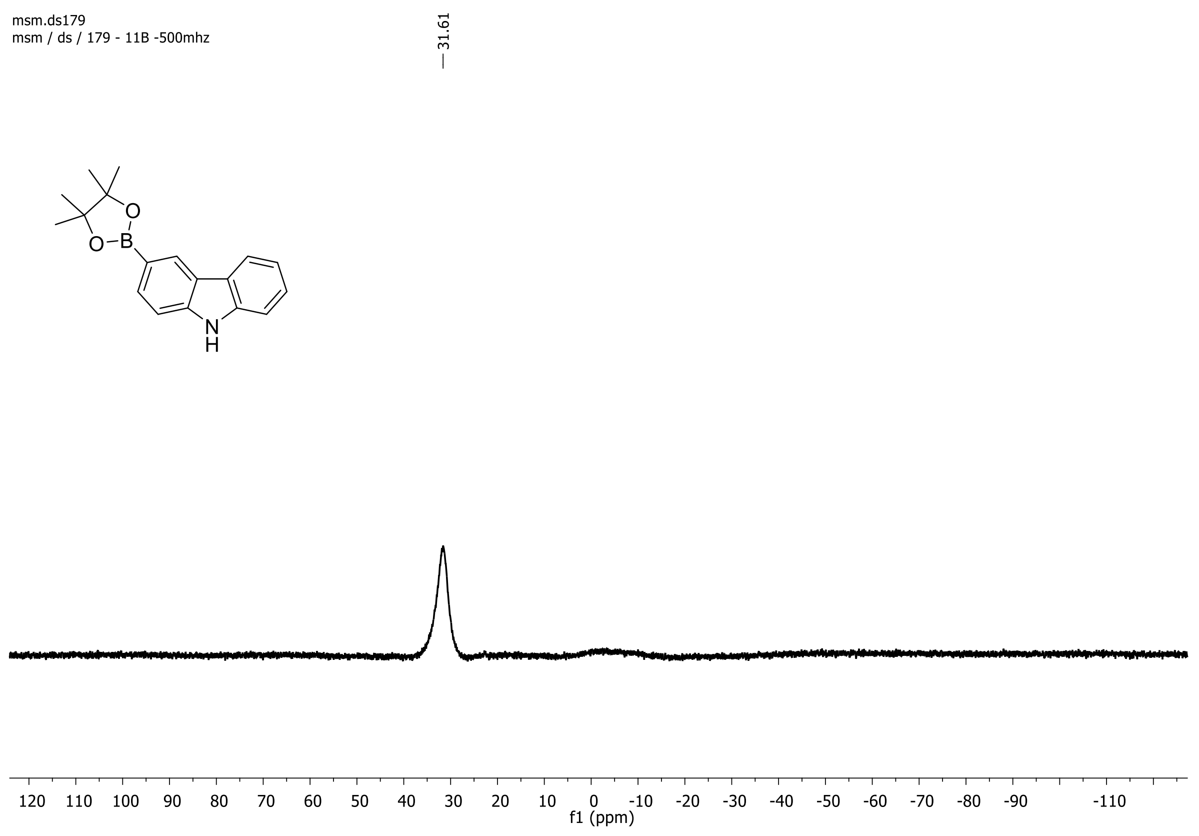

^{1}H (400 MHz) and ^{13}C (101 MHz) NMR of **2n** in $CDCl_3$

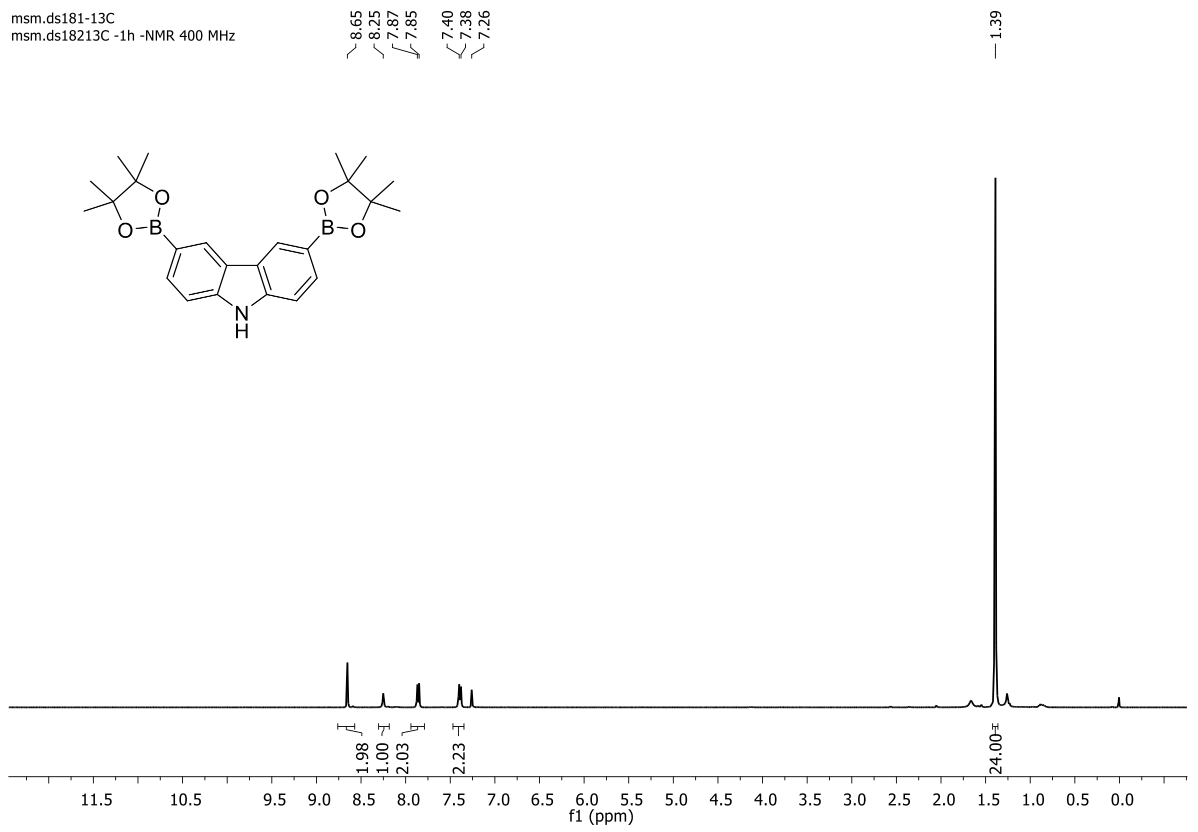

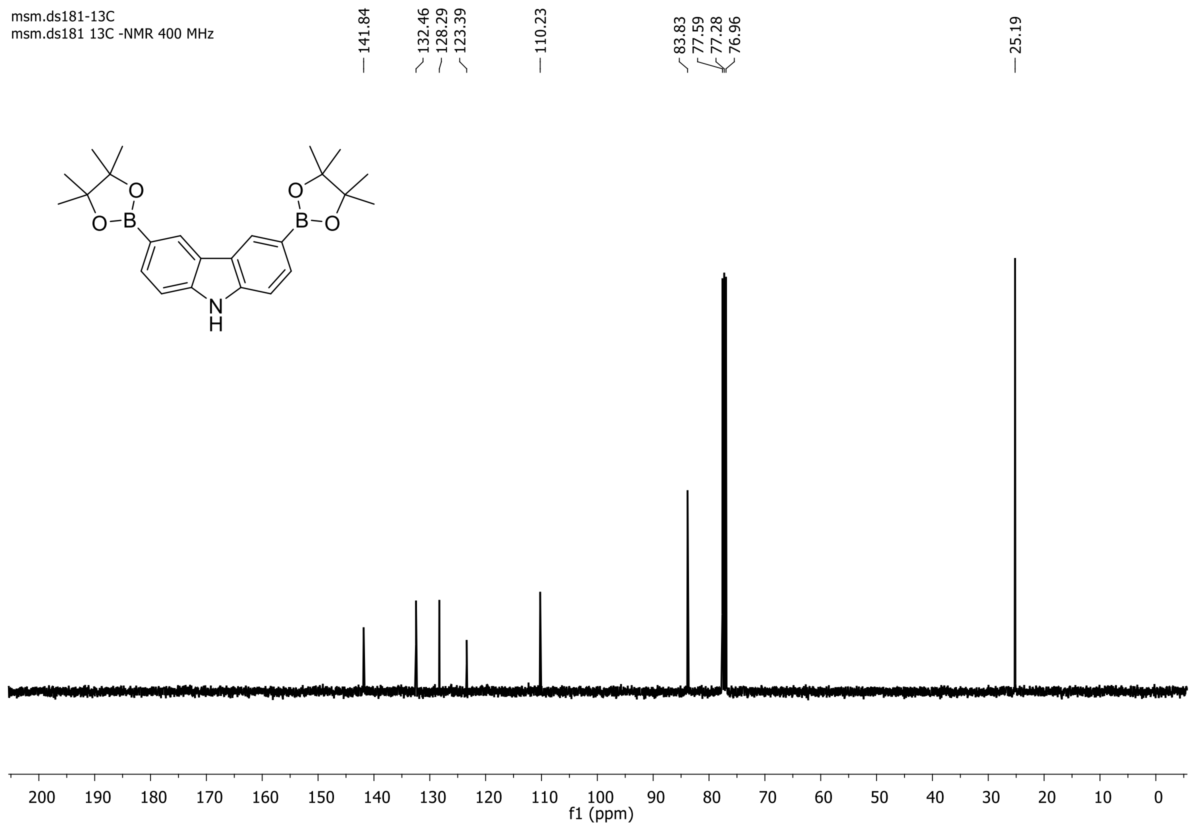

^{11}B (160 MHz) NMR of **2n** in $CDCl_3$

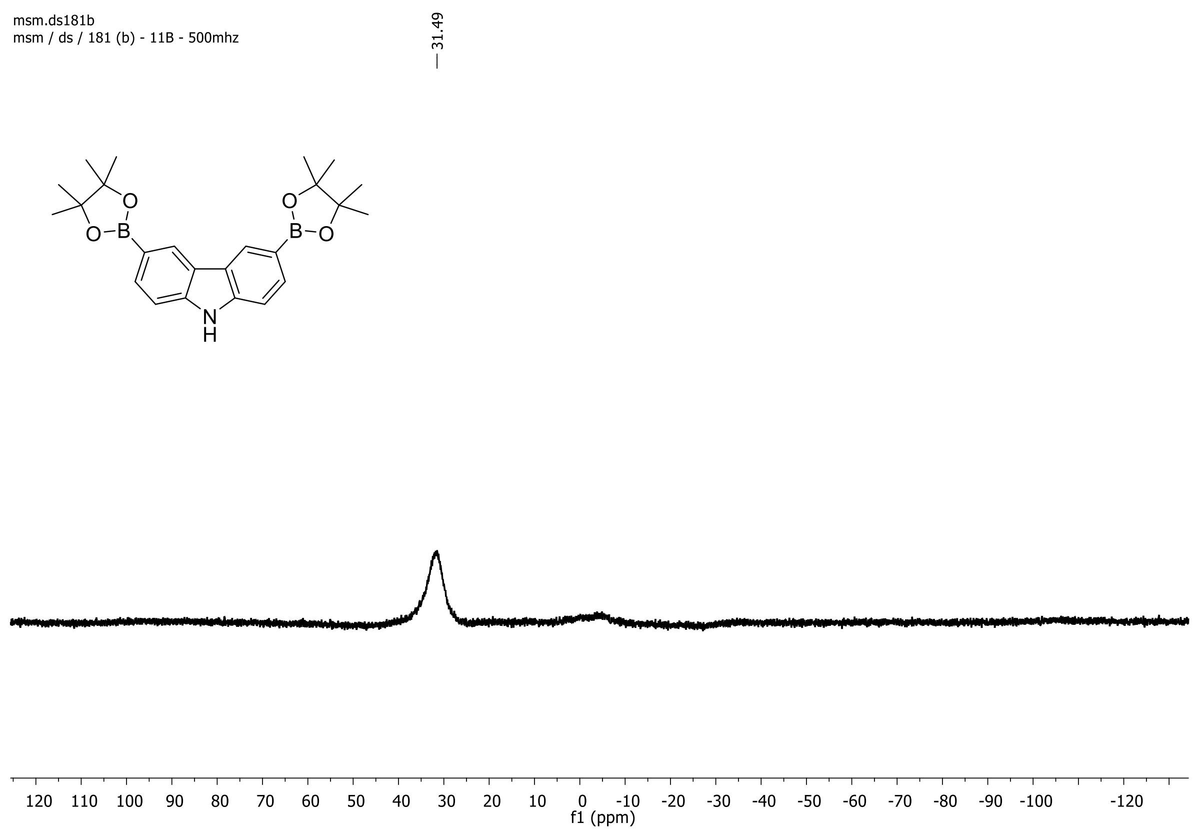

^{1}H (400 MHz) and ^{13}C (101 MHz) NMR of **2o** in $CDCl_3$

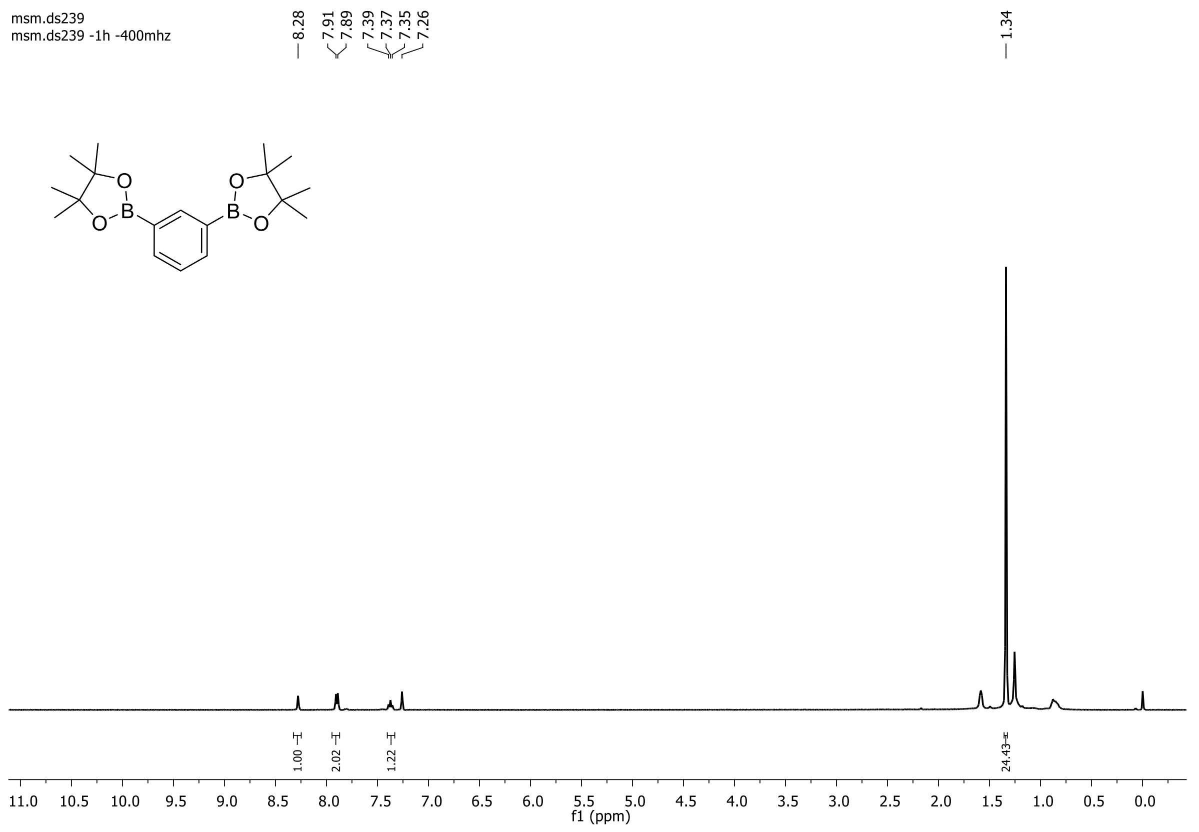

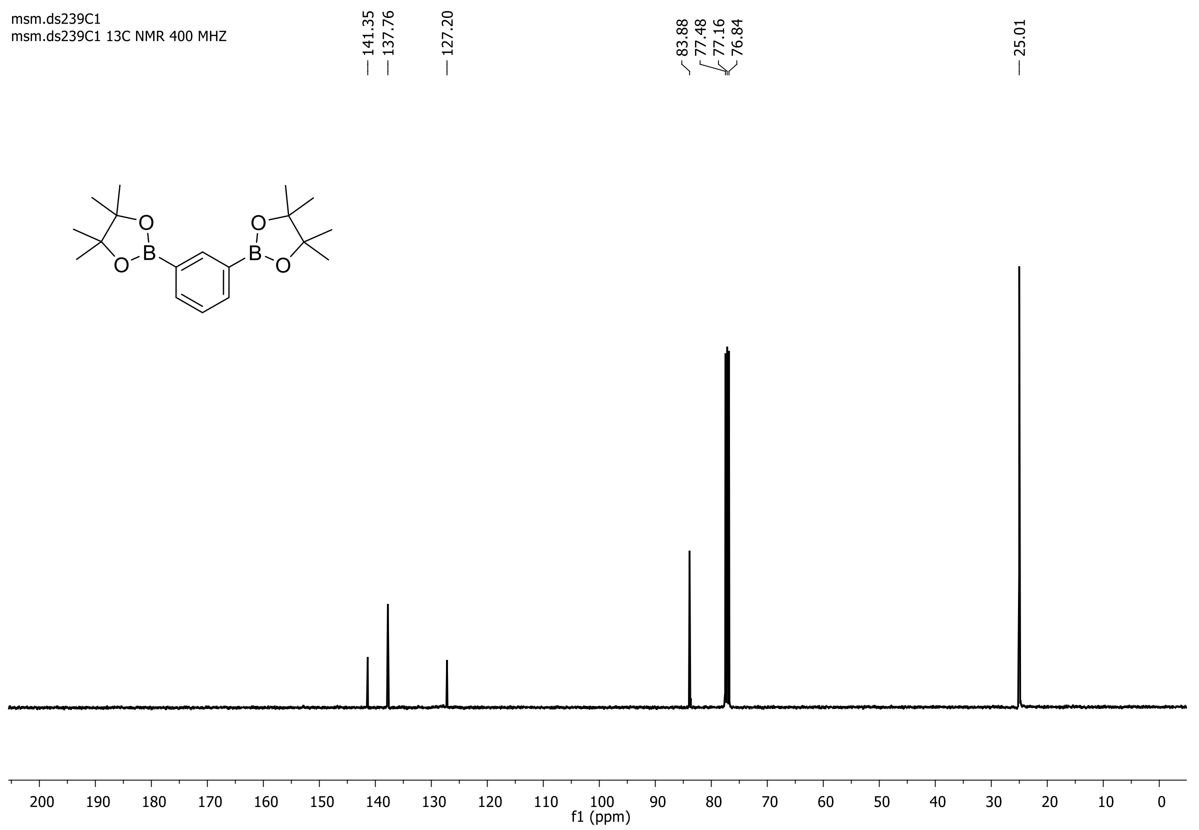

^{11}B (160 MHz) NMR of **2o** in $CDCl_3$

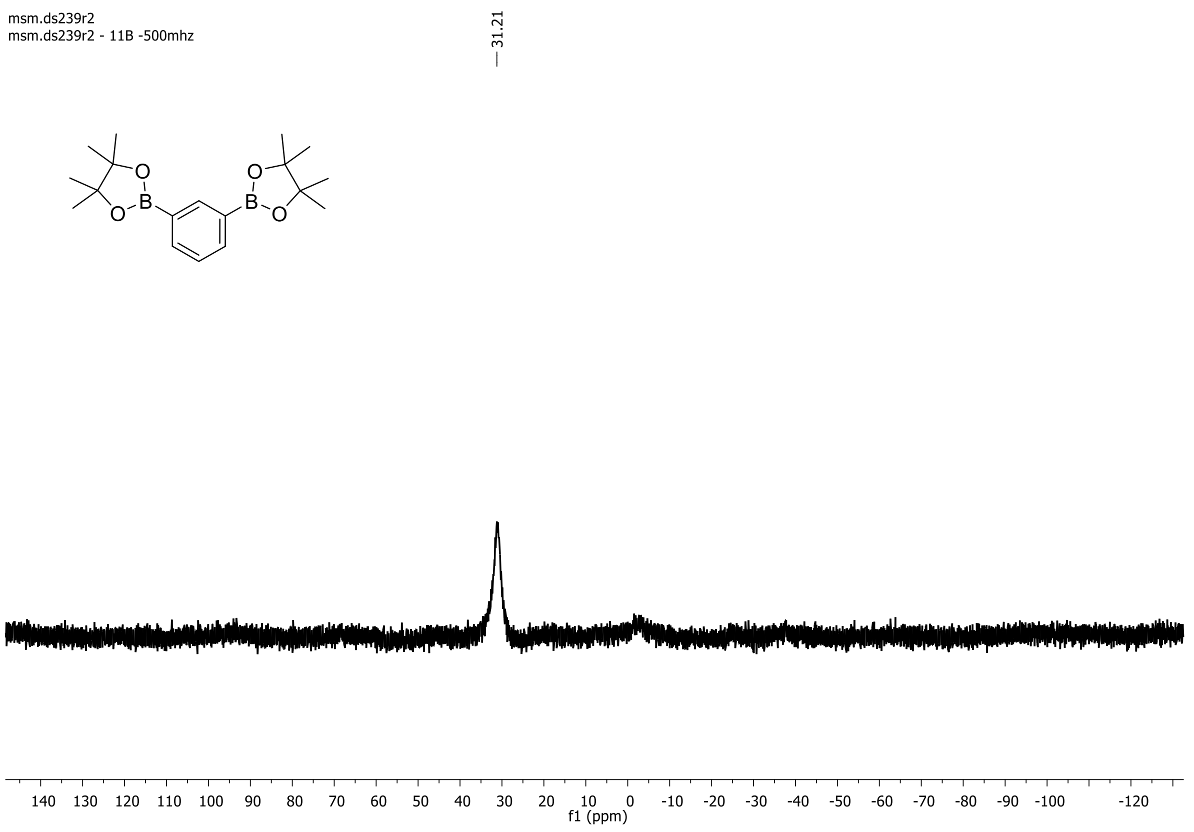

^{1}H (400 MHz) and ^{13}C (101 MHz) NMR of **2p** in $CDCl_3$

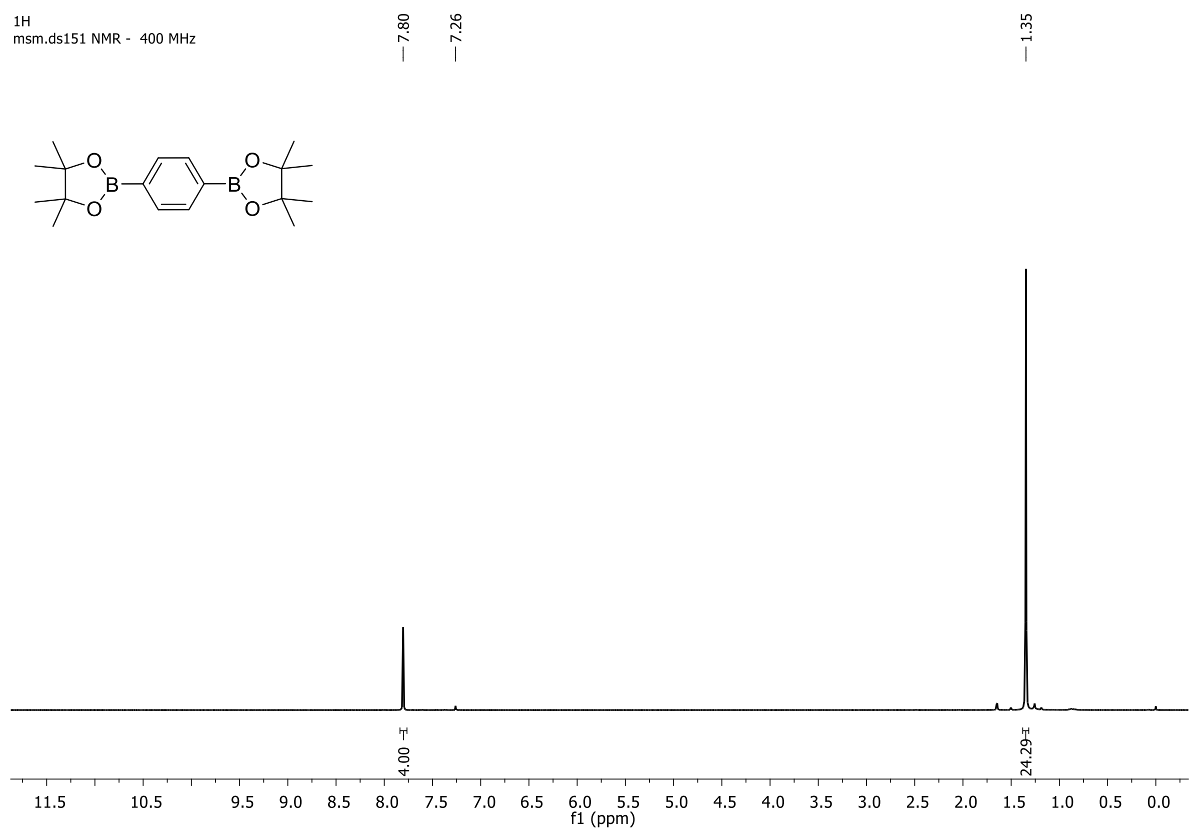

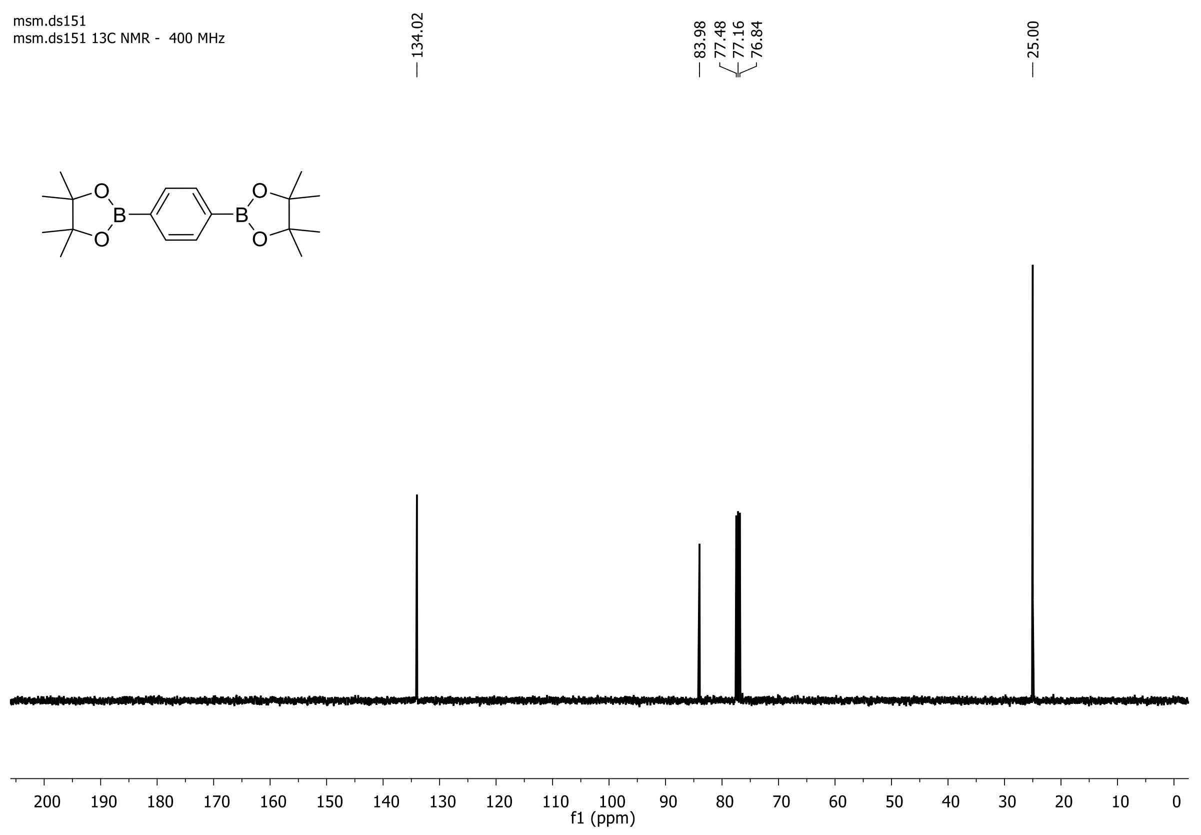

^{11}B (160 MHz) NMR of **2p** in $CDCl_3$

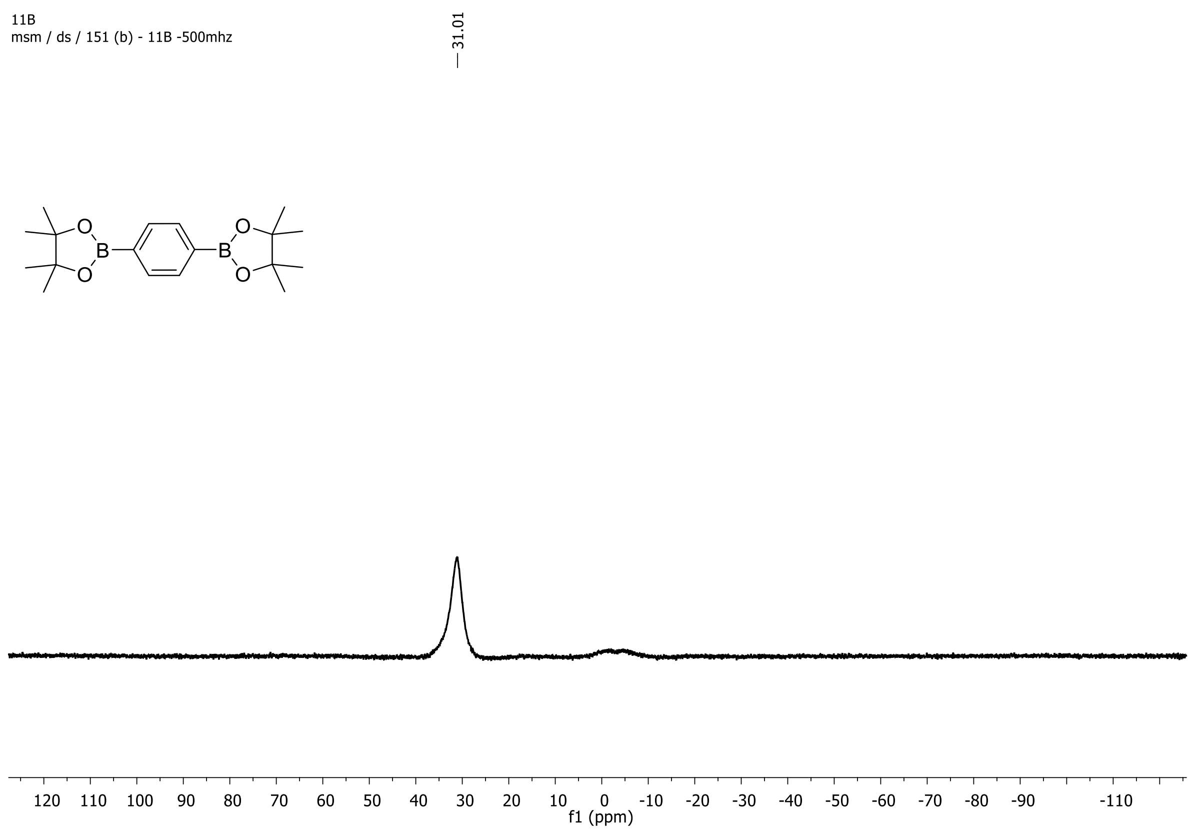

^{1}H (400 MHz) and ^{13}C (101 MHz) NMR of **2q** in $CDCl_3$

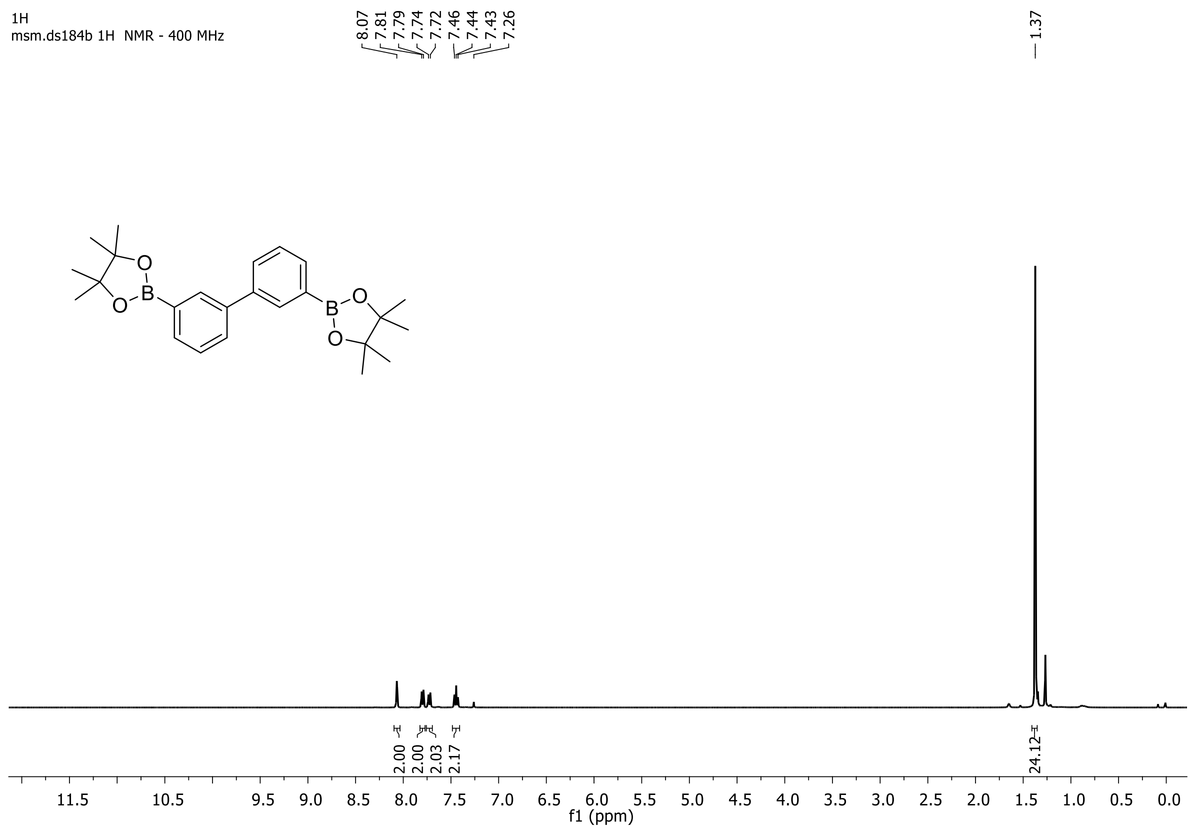

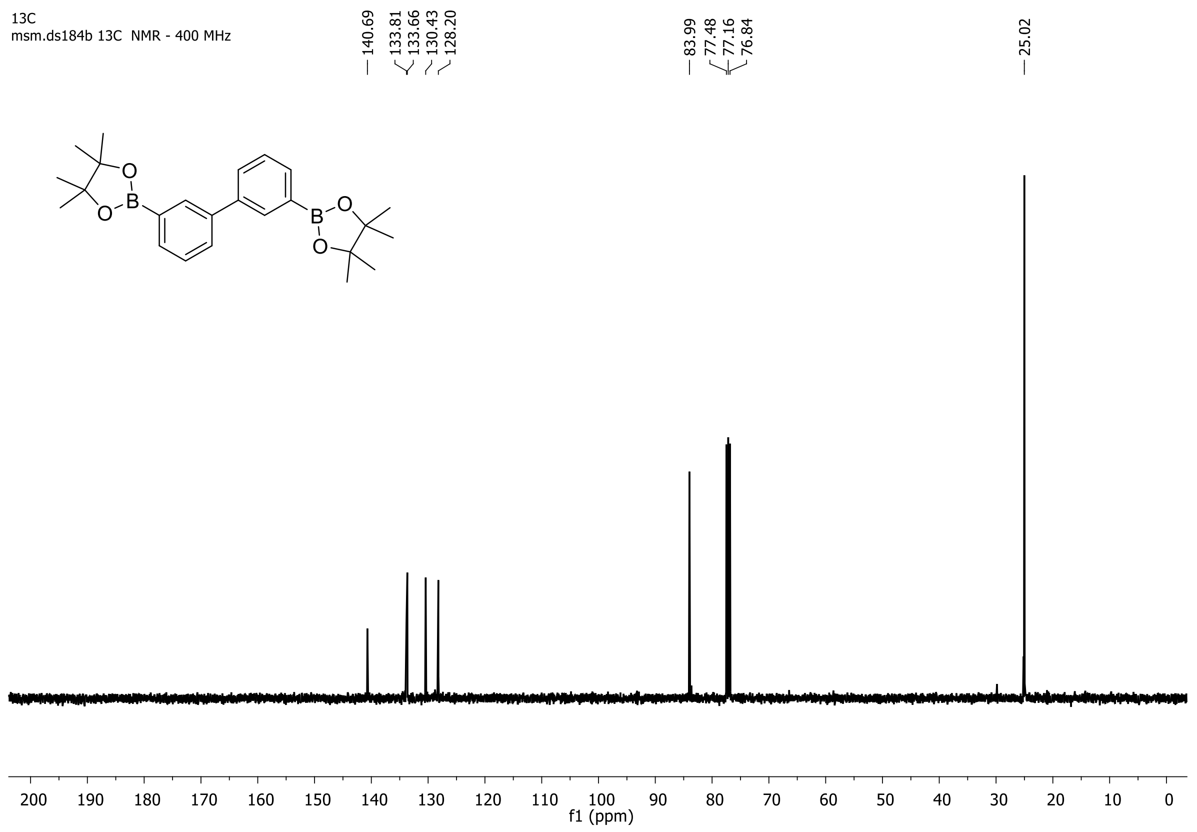

^{11}B (160 MHz) NMR of **2q** in $CDCl_3$

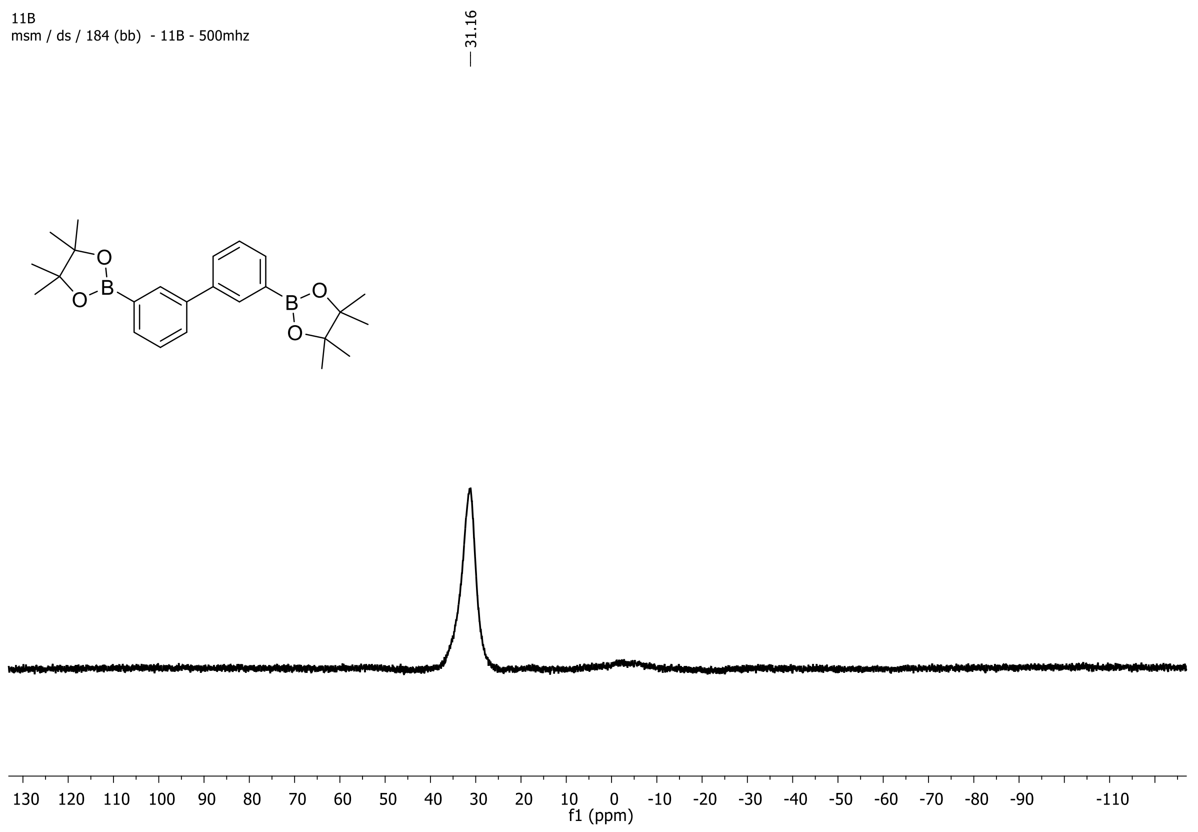

^{1}H (400 MHz) and ^{13}C (101 MHz) NMR of **2r** in $CDCl_3$

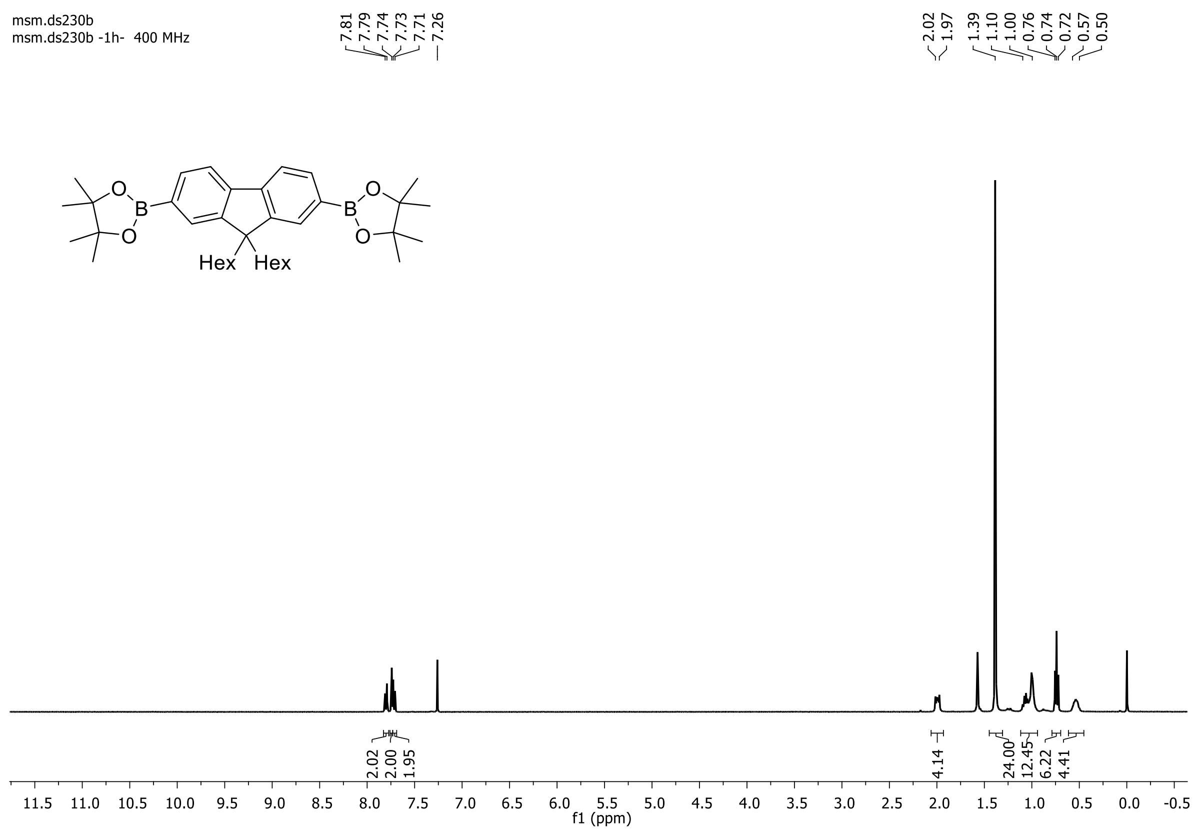

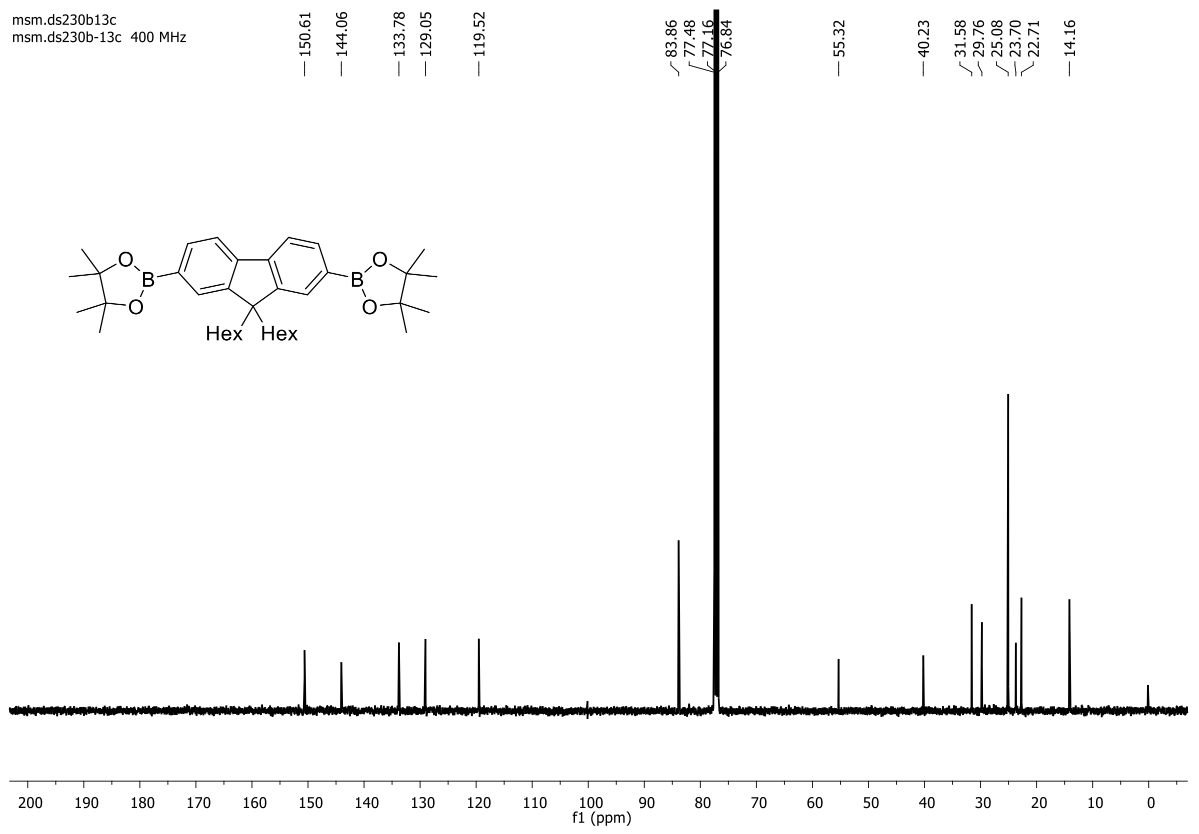

^{1}H (400 MHz) and ^{13}C (101 MHz) NMR of **3a** in $CDCl_3$

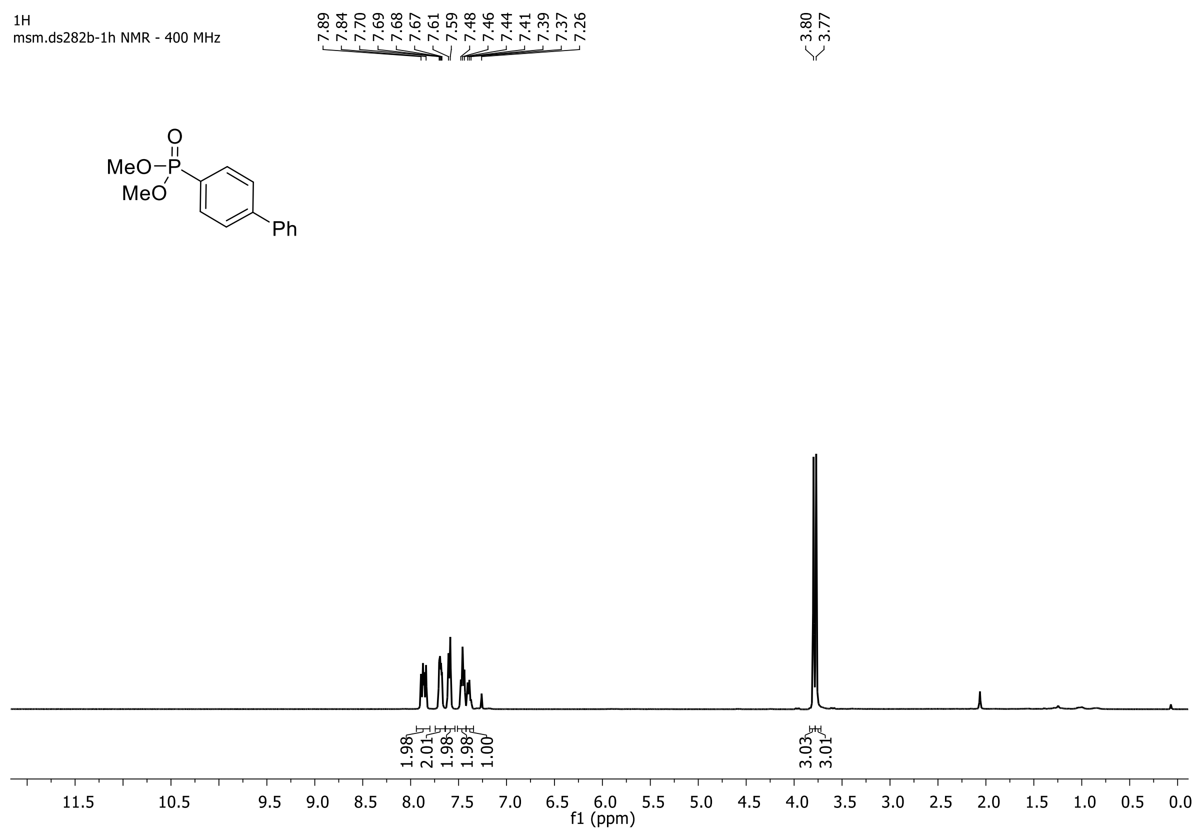

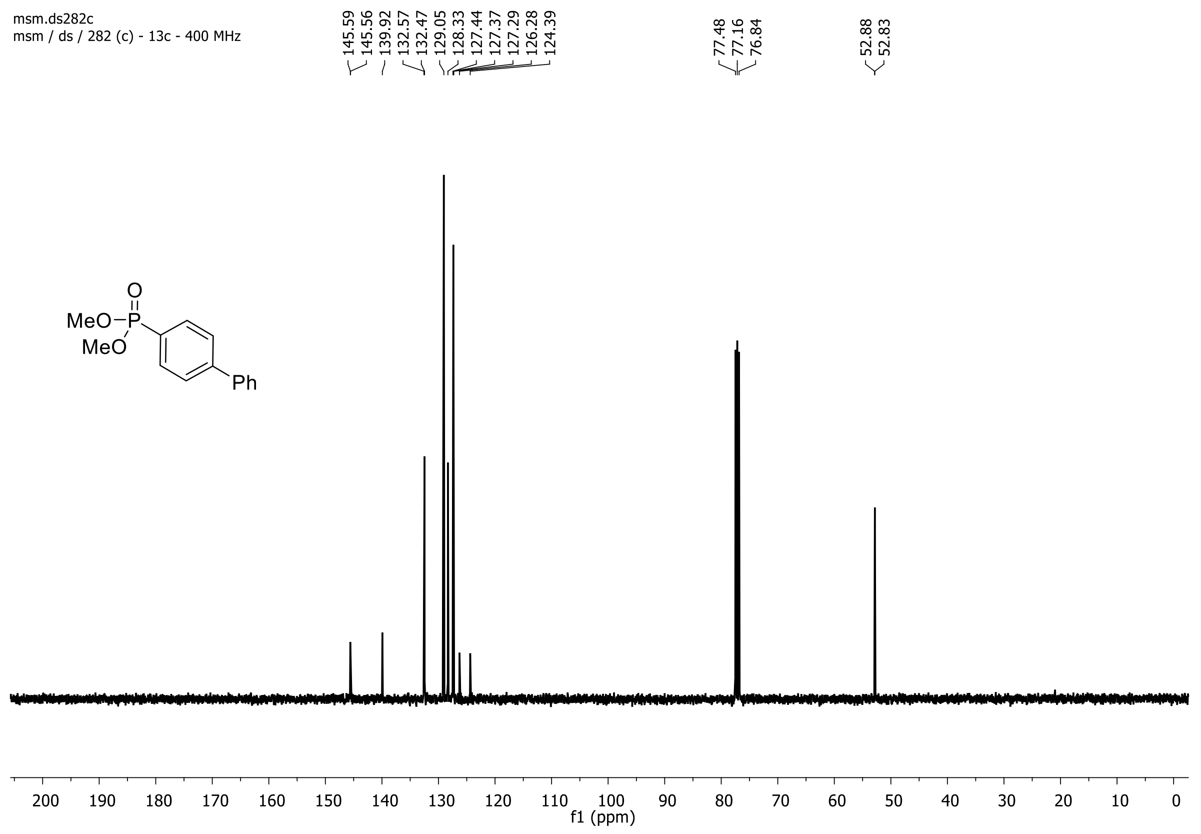

^{31}P NMR (202 MHz) of **3a** in $CDCl_3$

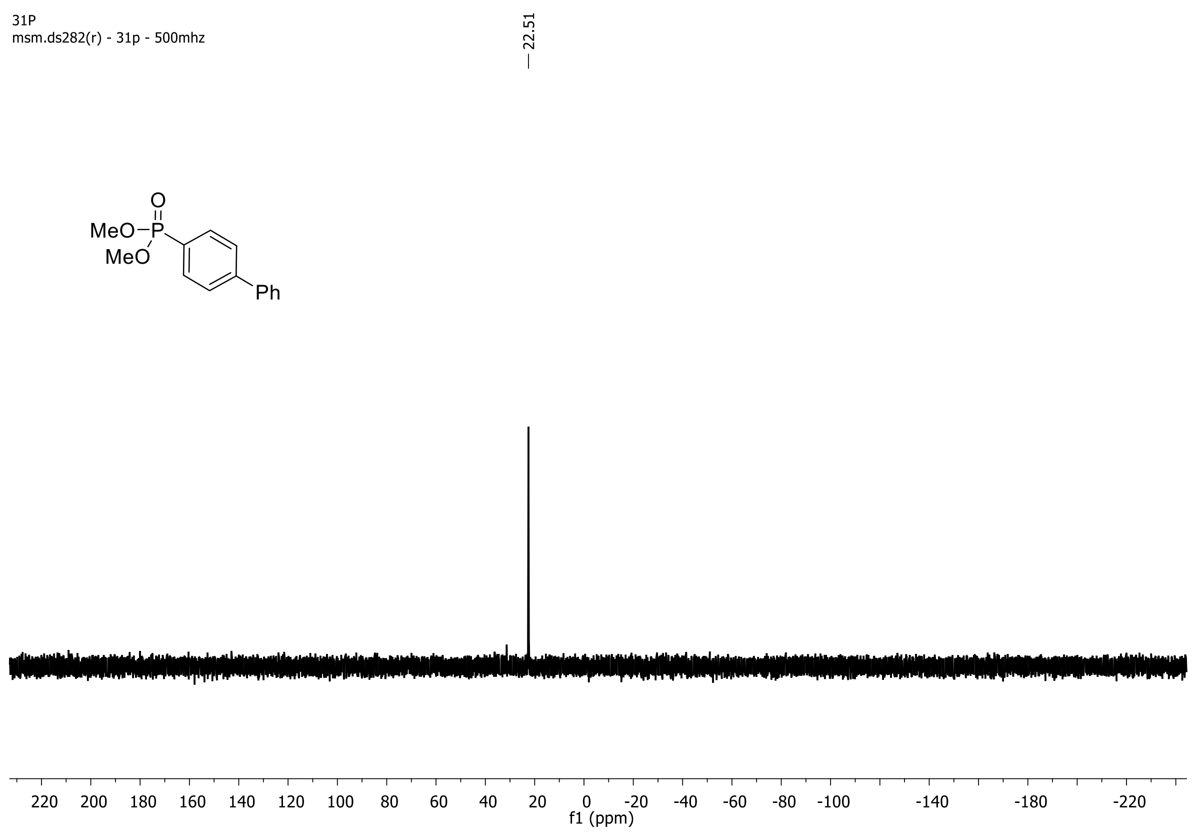

^{1}H (400 MHz) and ^{13}C (101 MHz) NMR of **3b** in $CDCl_3$

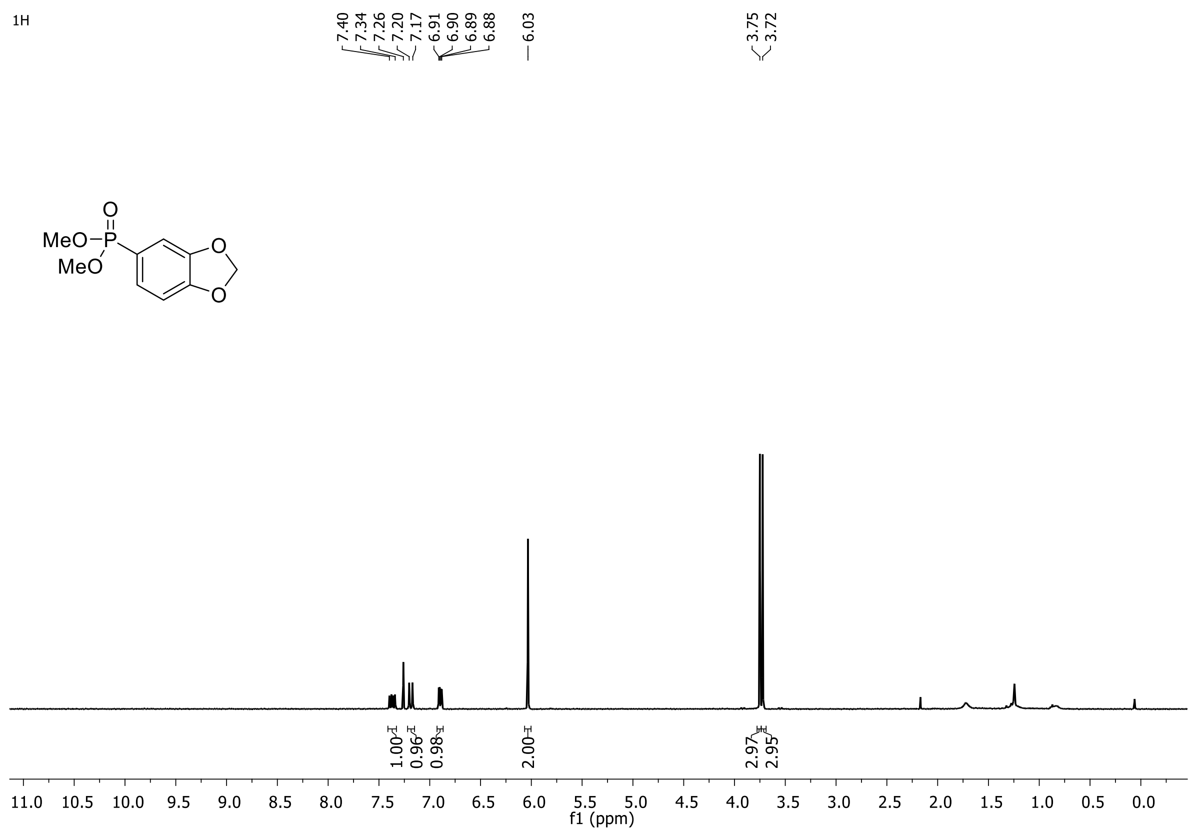

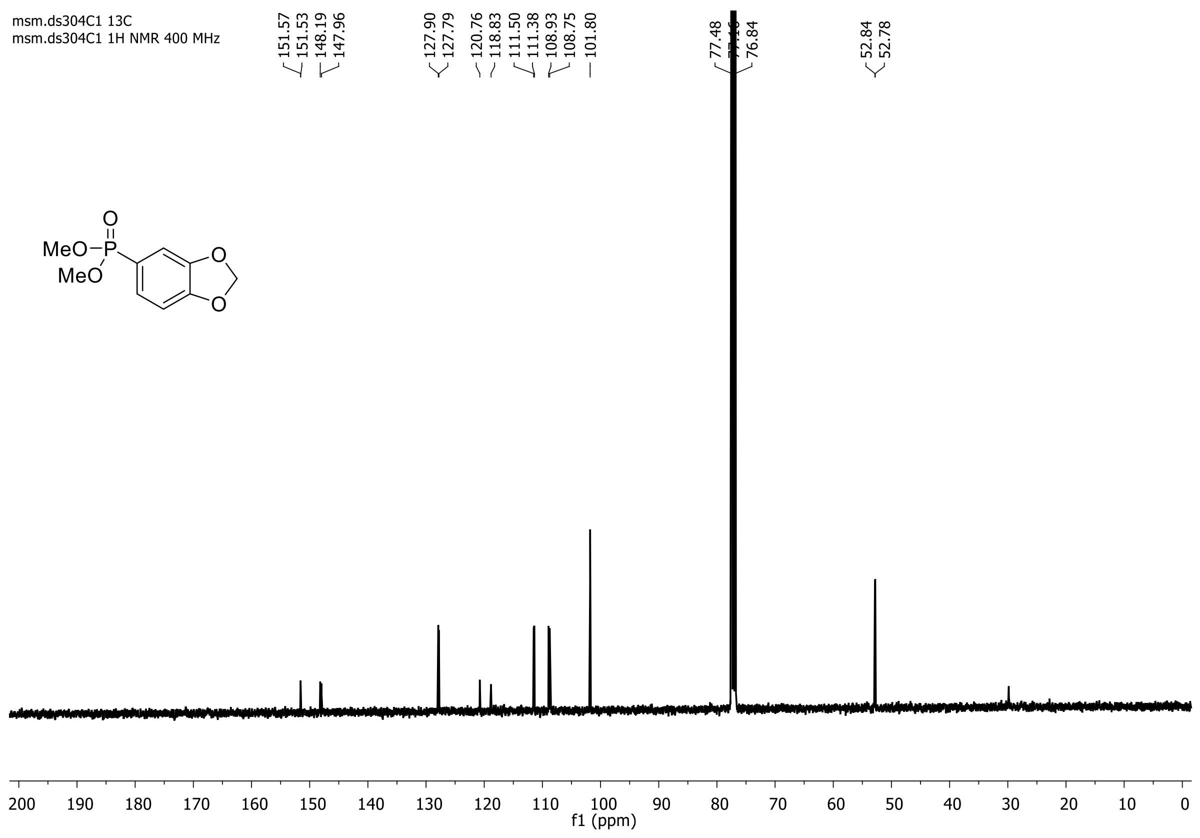

^{31}P (202 MHz) NMR of **3b** in $CDCl_3$

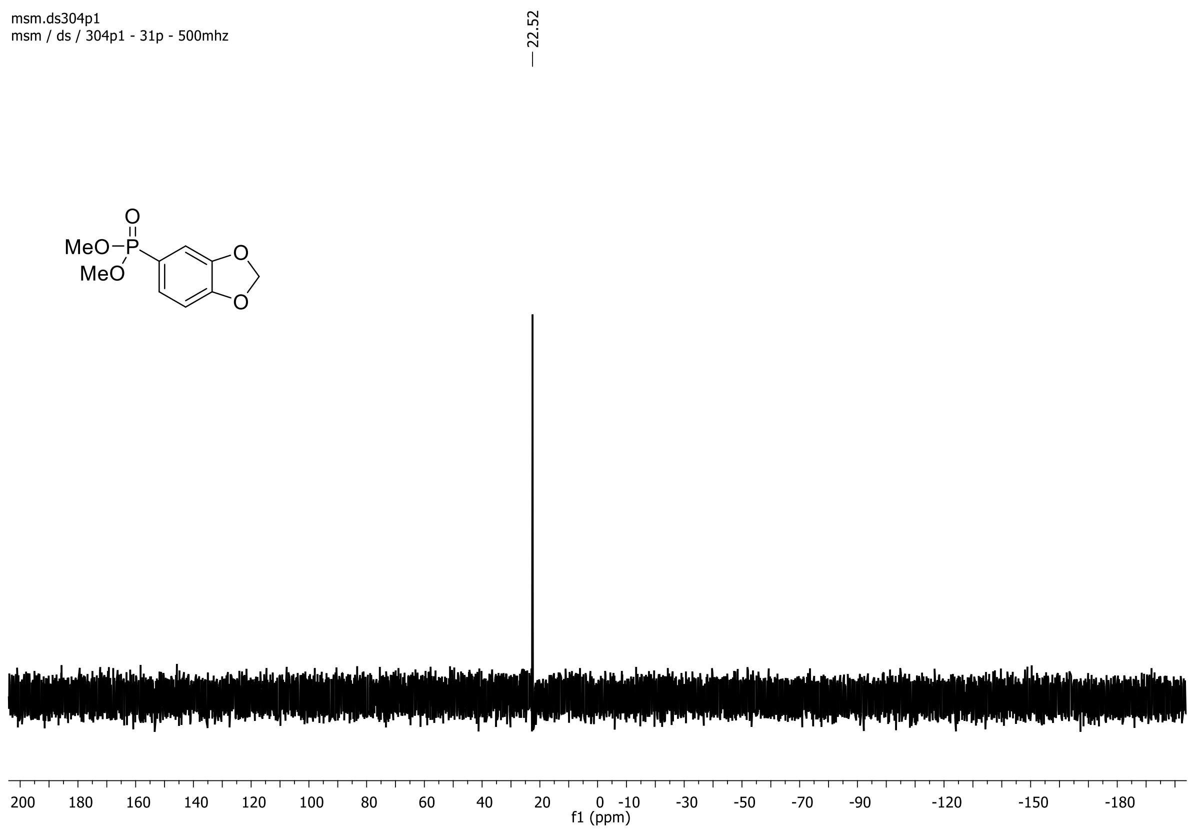

^{1}H (400 MHz) and ^{13}C (101 MHz) NMR of **3c** in $CDCl_3$

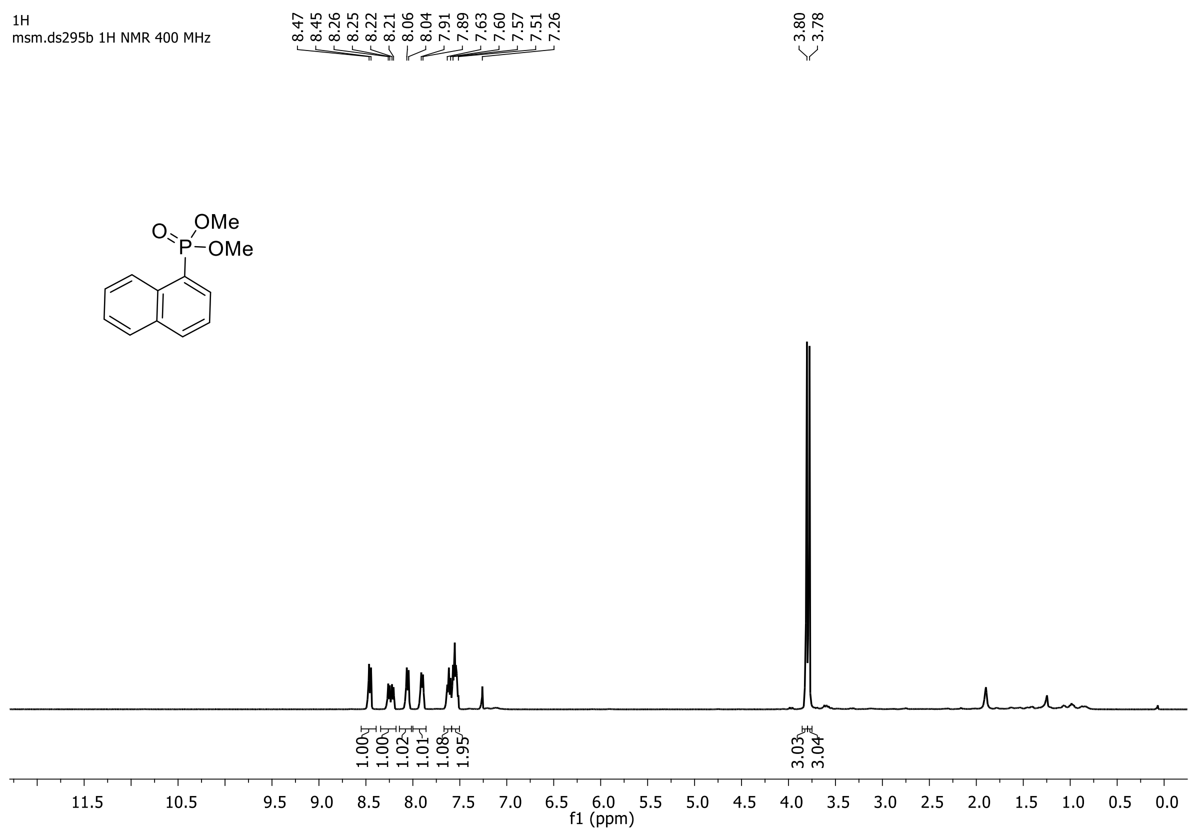

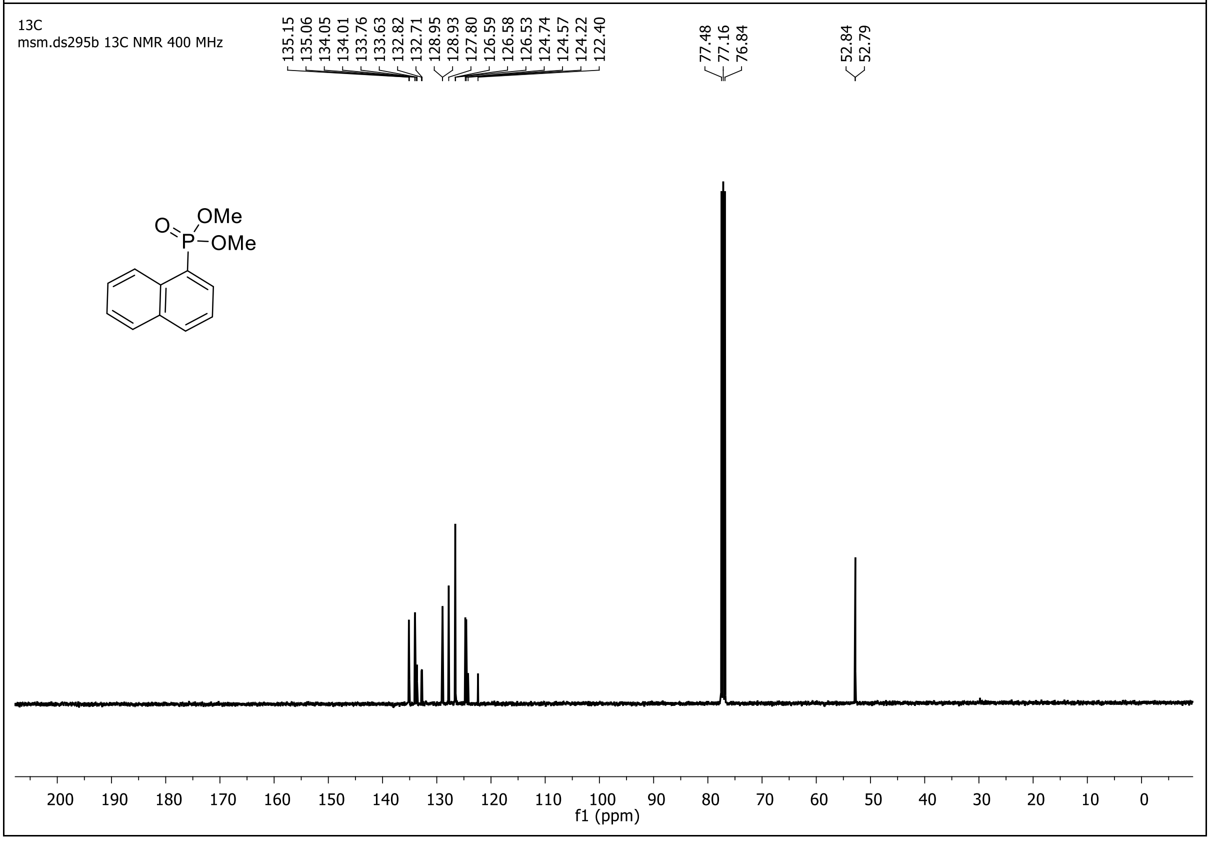

^{31}P (202 MHz) NMR of **3c** in $CDCl_3$

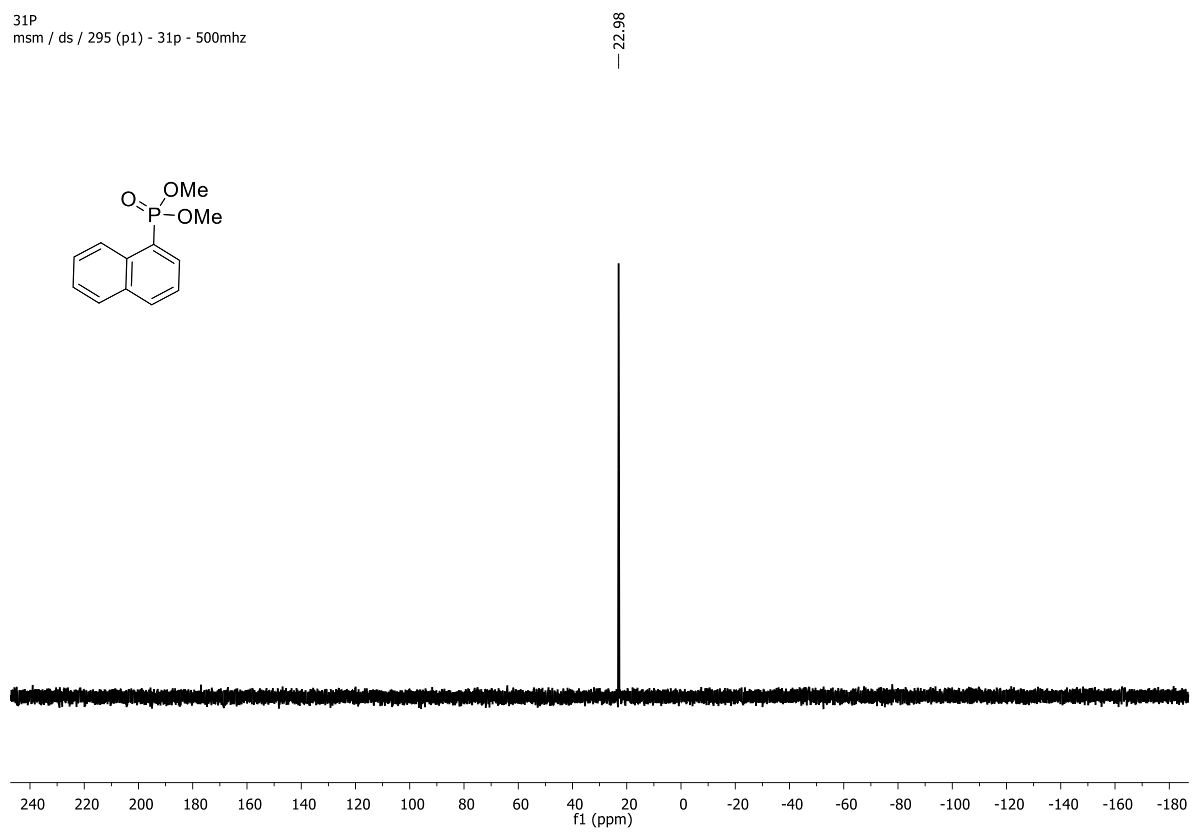

^{1}H (400 MHz) and ^{13}C (126 MHz) NMR of **3d** in $CDCl_3$

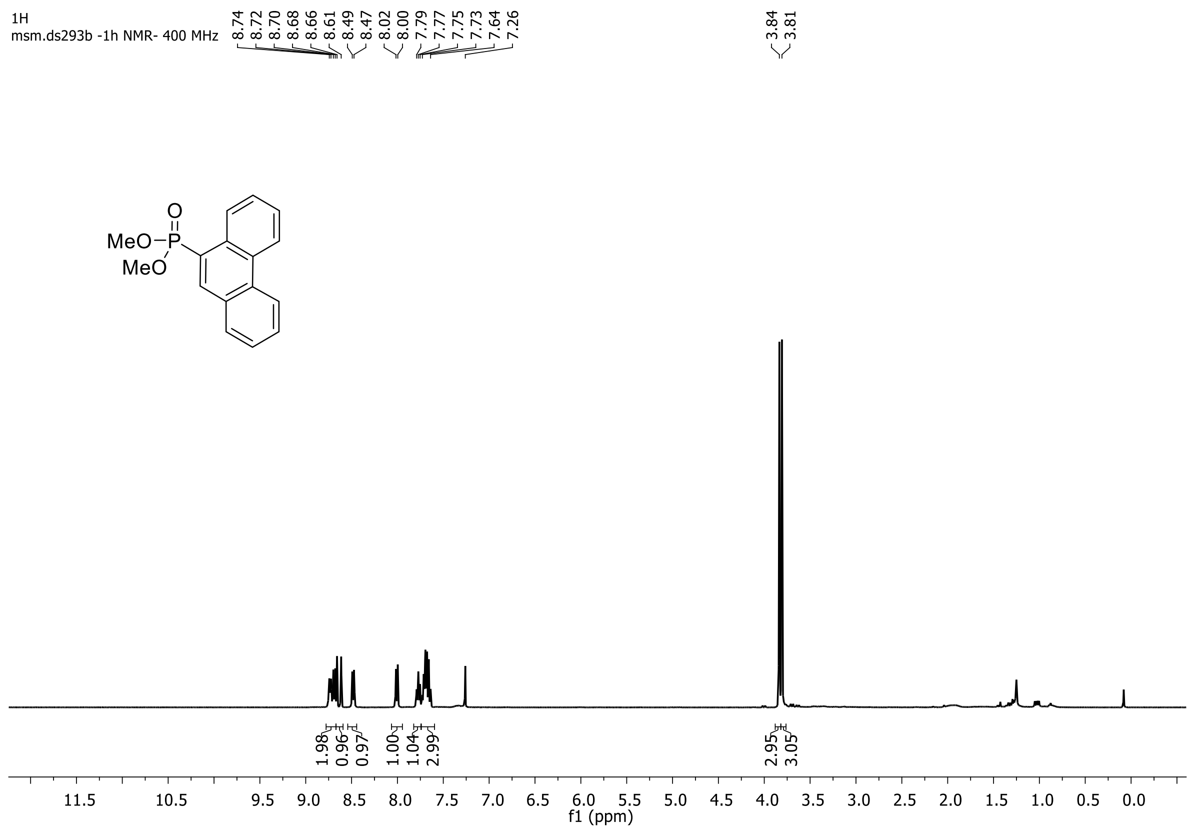

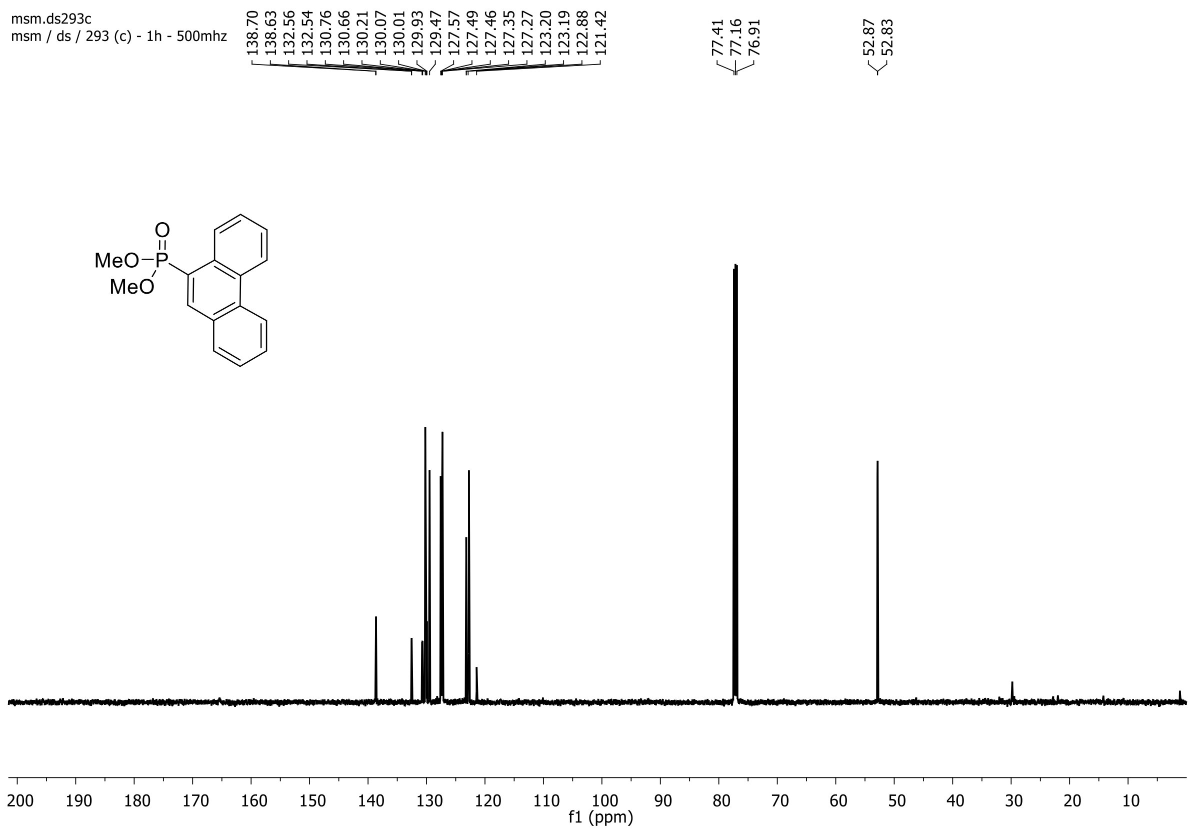

^{31}P (202 MHz) NMR of **3d** in $CDCl_3$

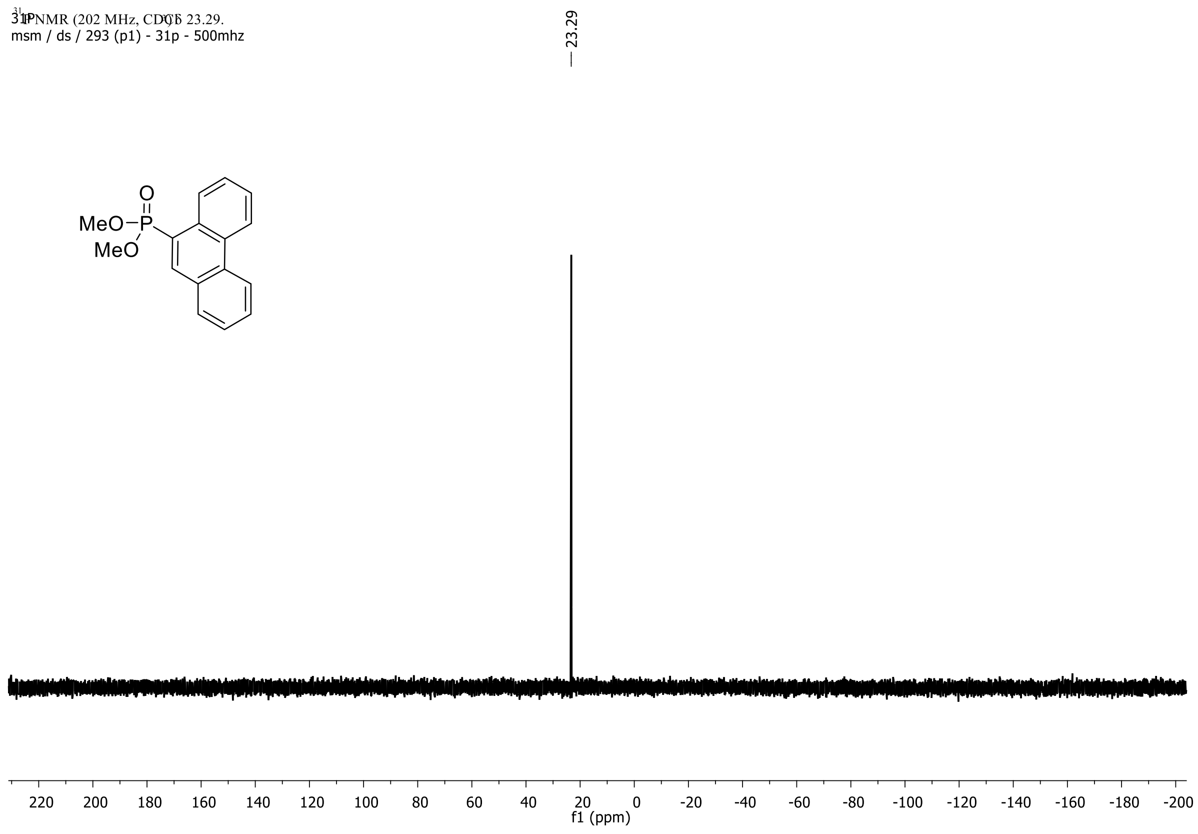

^{1}H (400 MHz) and ^{13}C (101 MHz) NMR of **3e** in $CDCl_3$

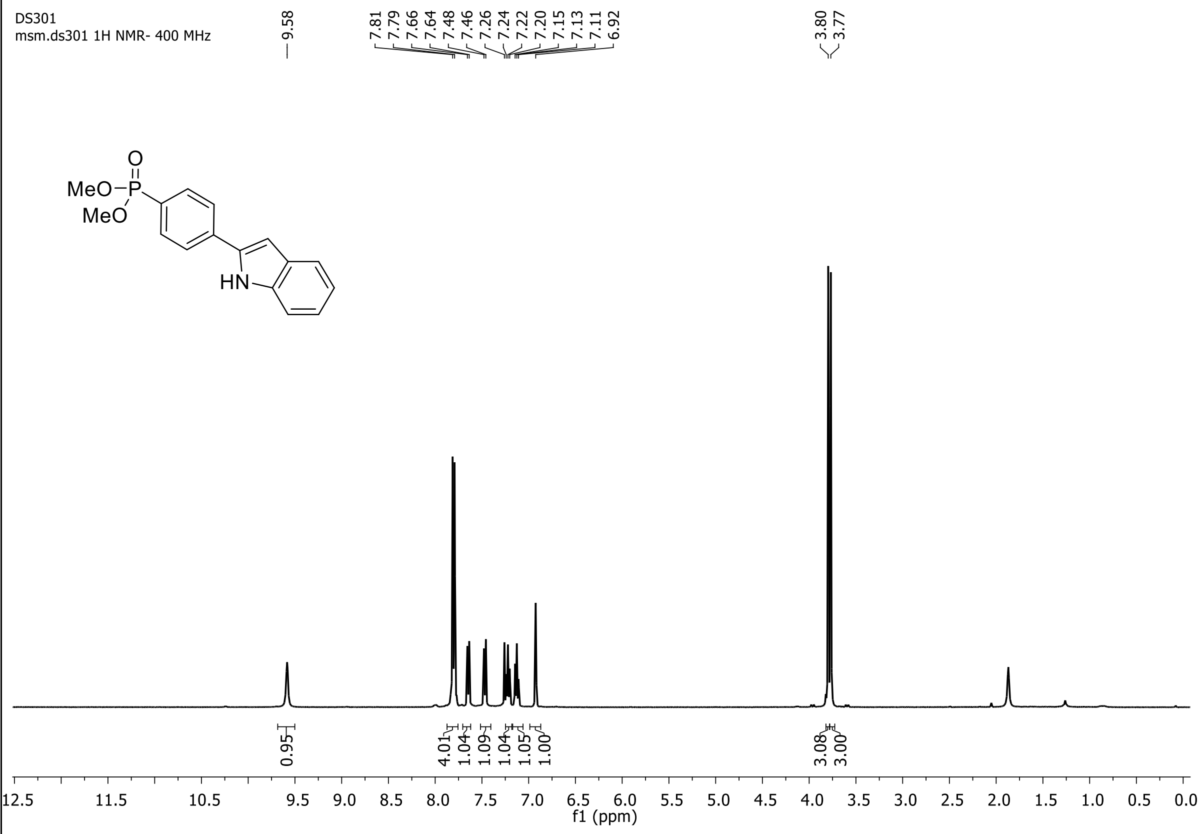

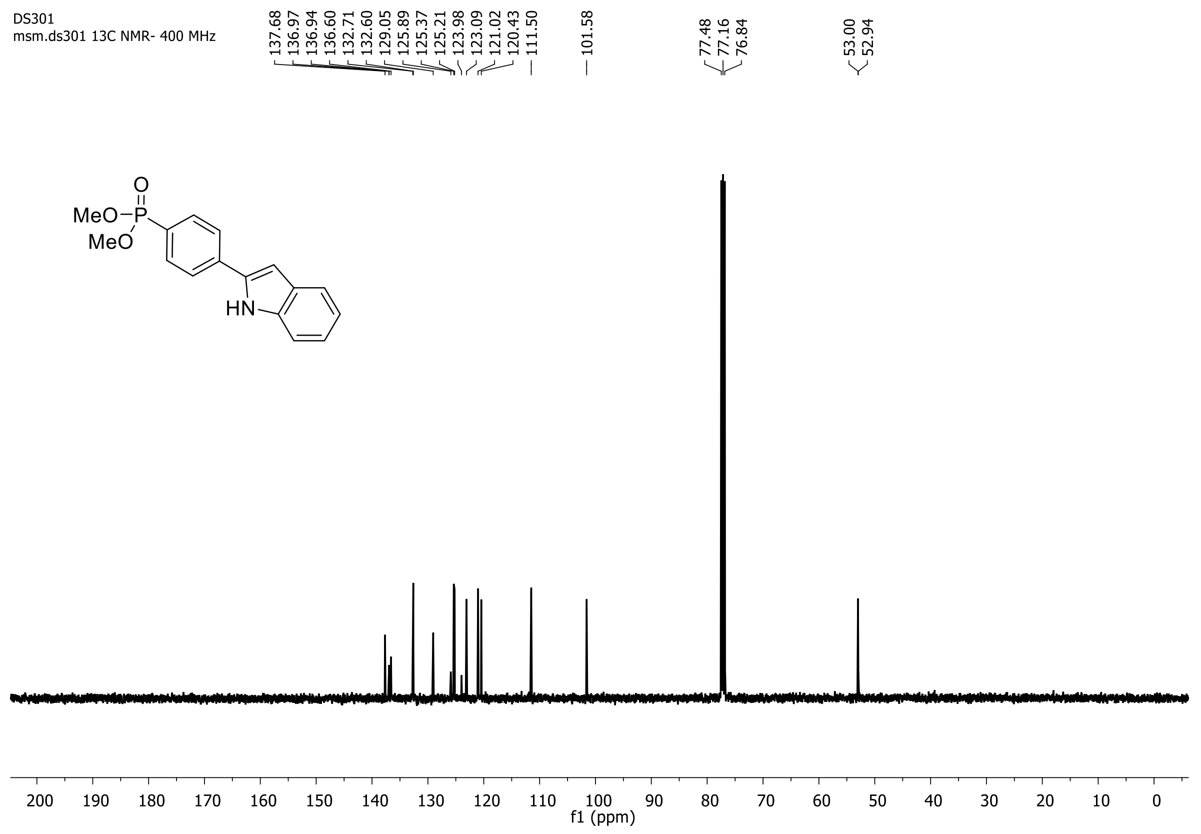

^{31}P (202 MHz) NMR of **3e** in $CDCl_3$

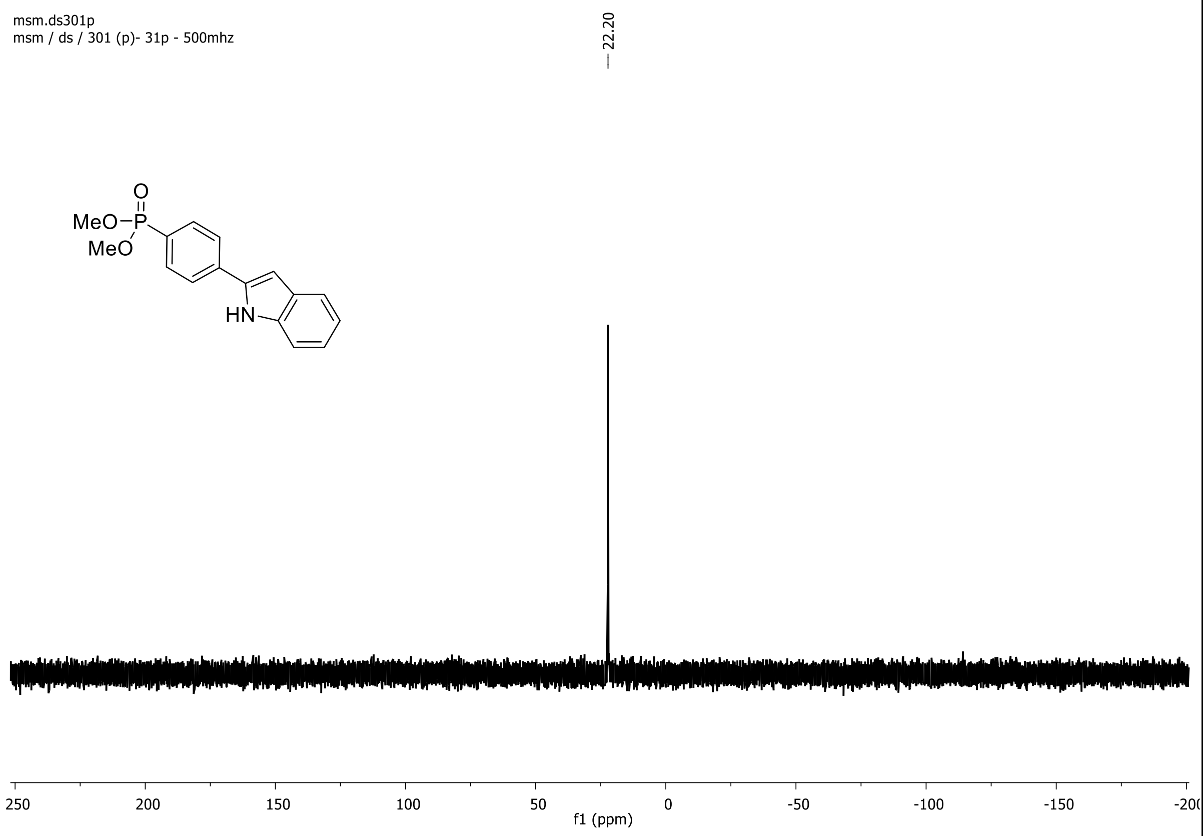

^{1}H (400 MHz) and ^{13}C (126 MHz) NMR of **3f** in $CDCl_3$

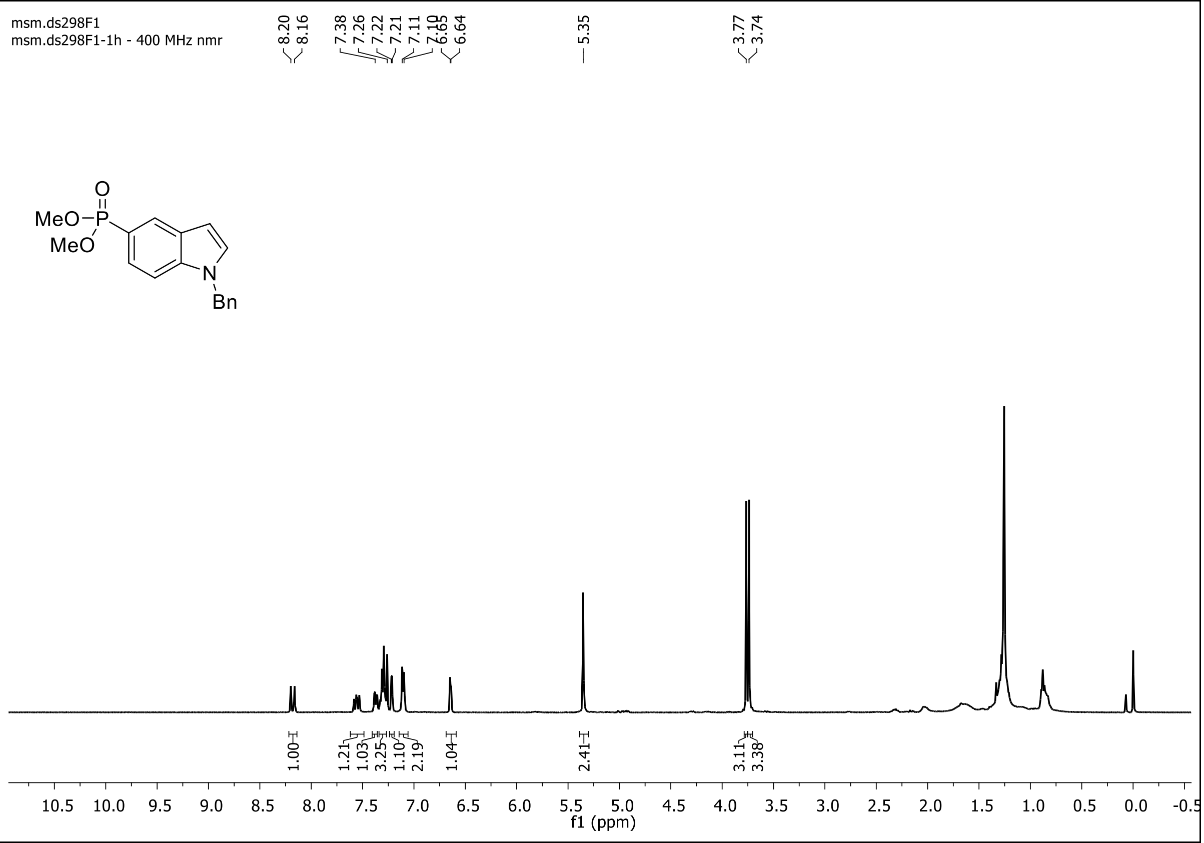

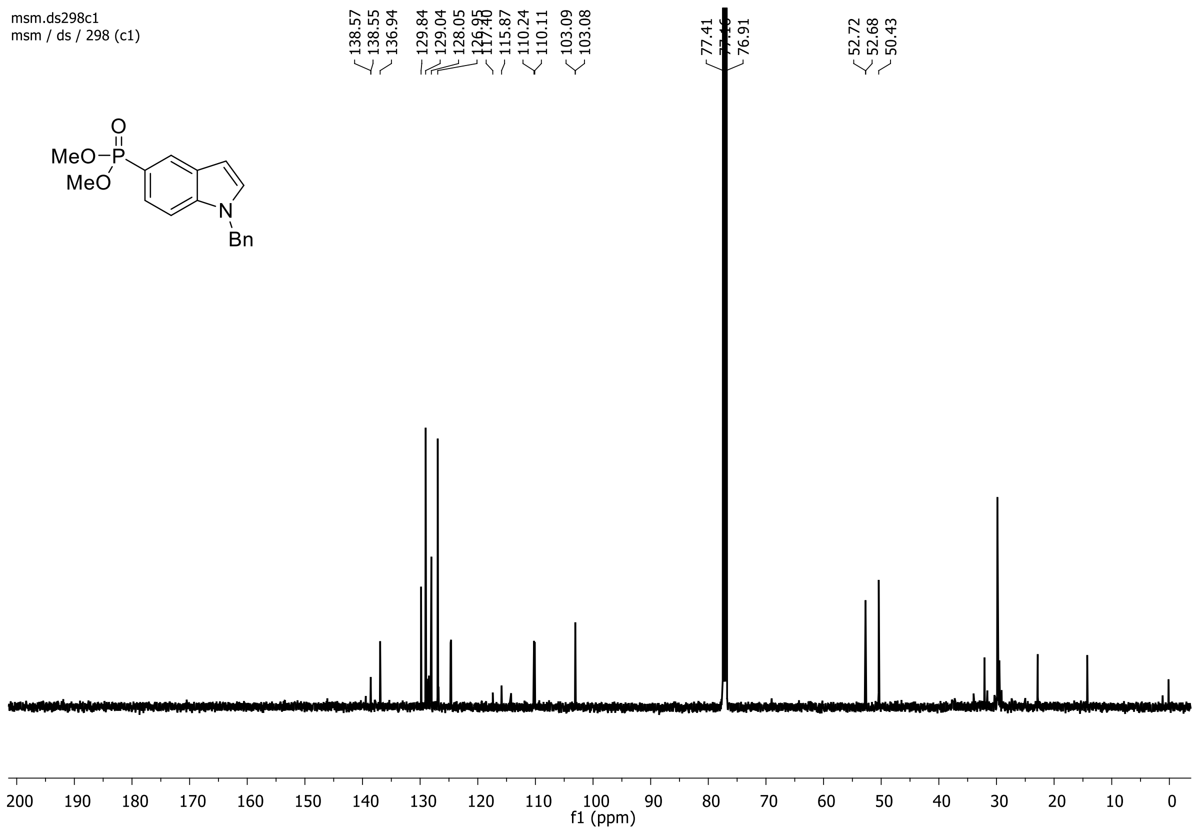

^{31}P (202 MHz) NMR of **3f** in $CDCl_3$

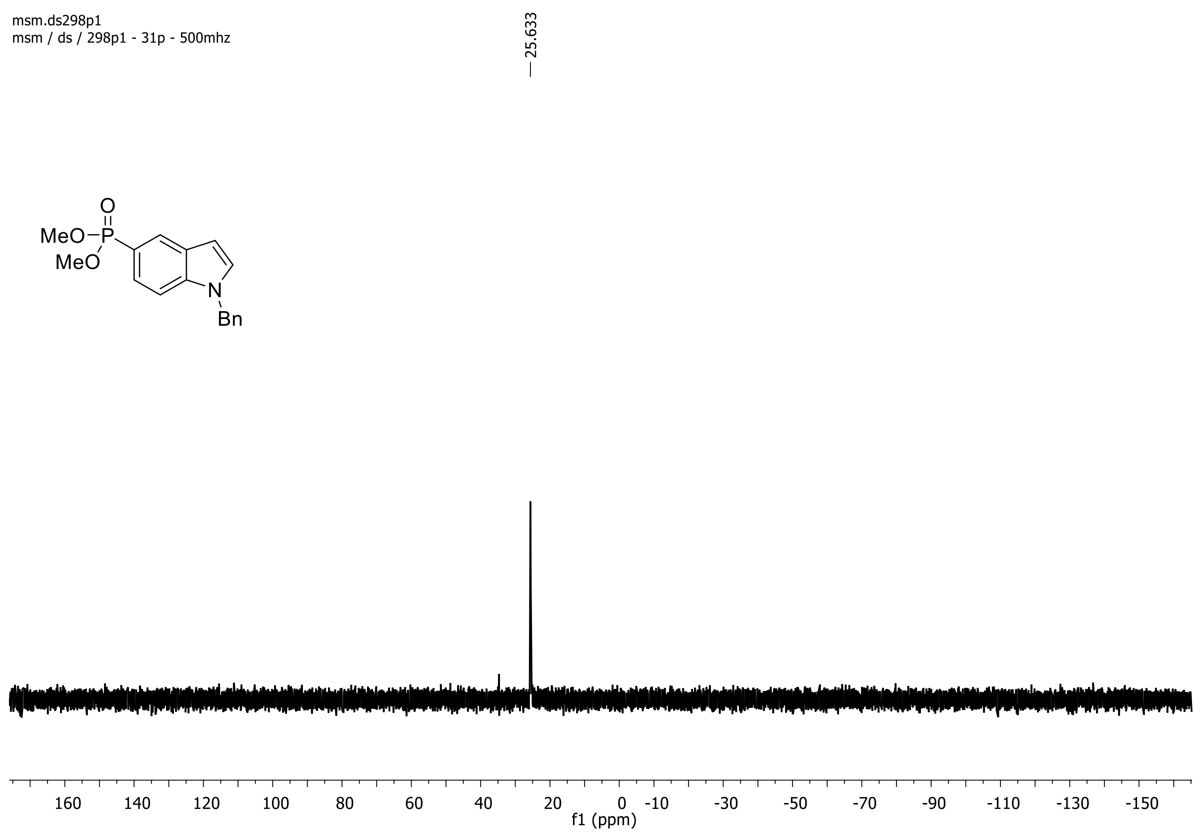

^{1}H (400 MHz) and ^{13}C (101 MHz) NMR of **5a** in $CDCl_3$

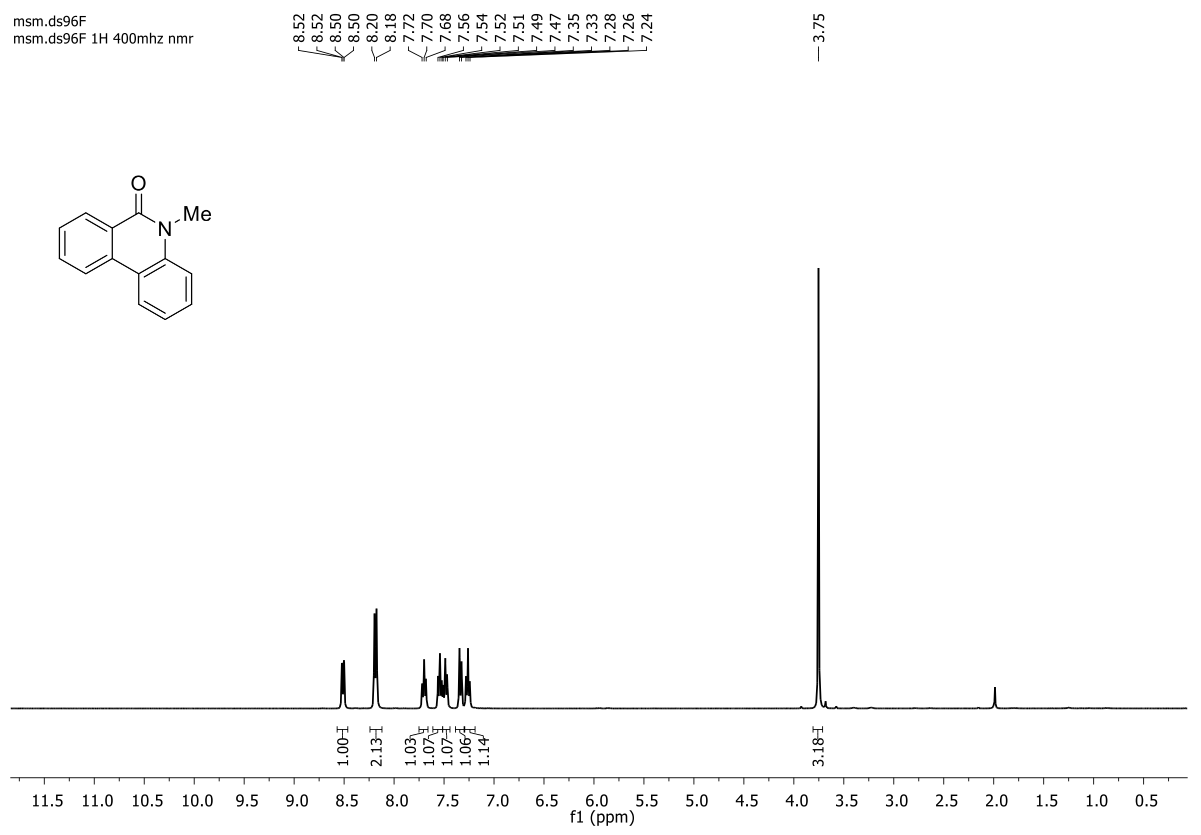

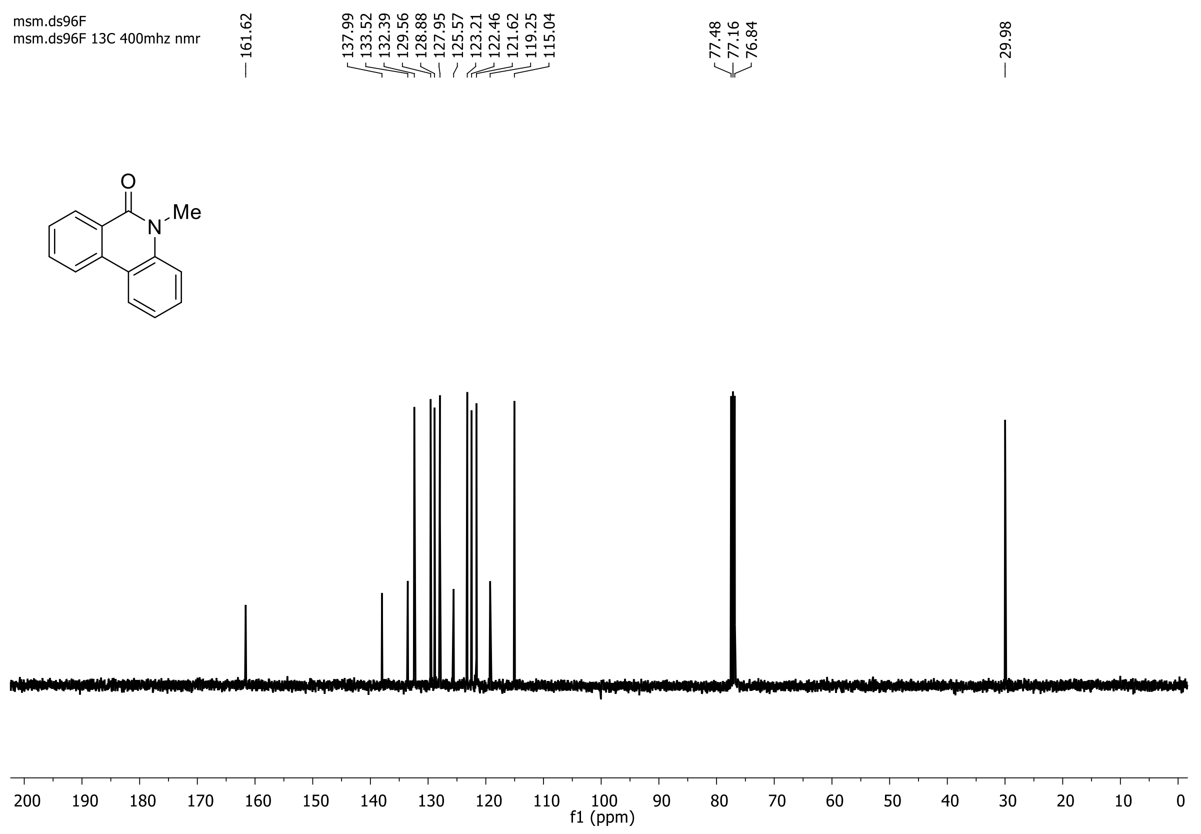

^{1}H (400 MHz) and ^{13}C (101 MHz) NMR of **5b** in $CDCl_3$

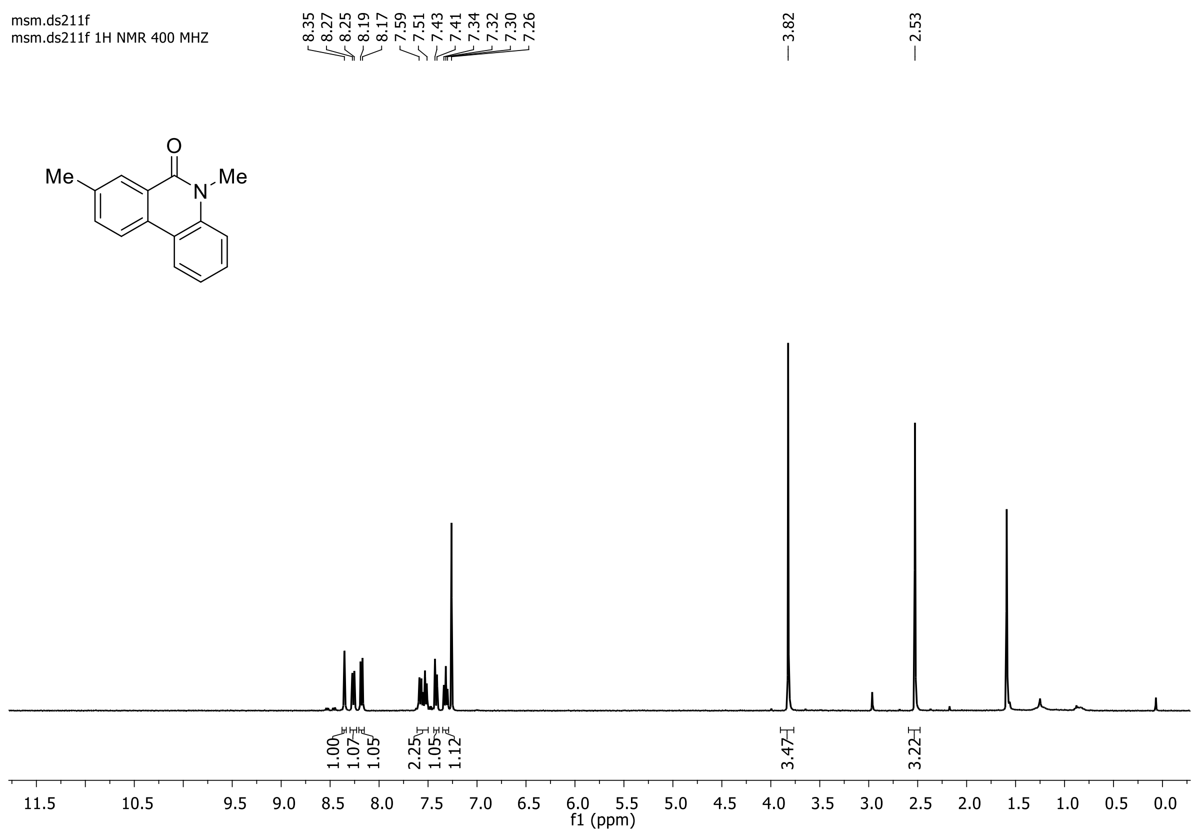

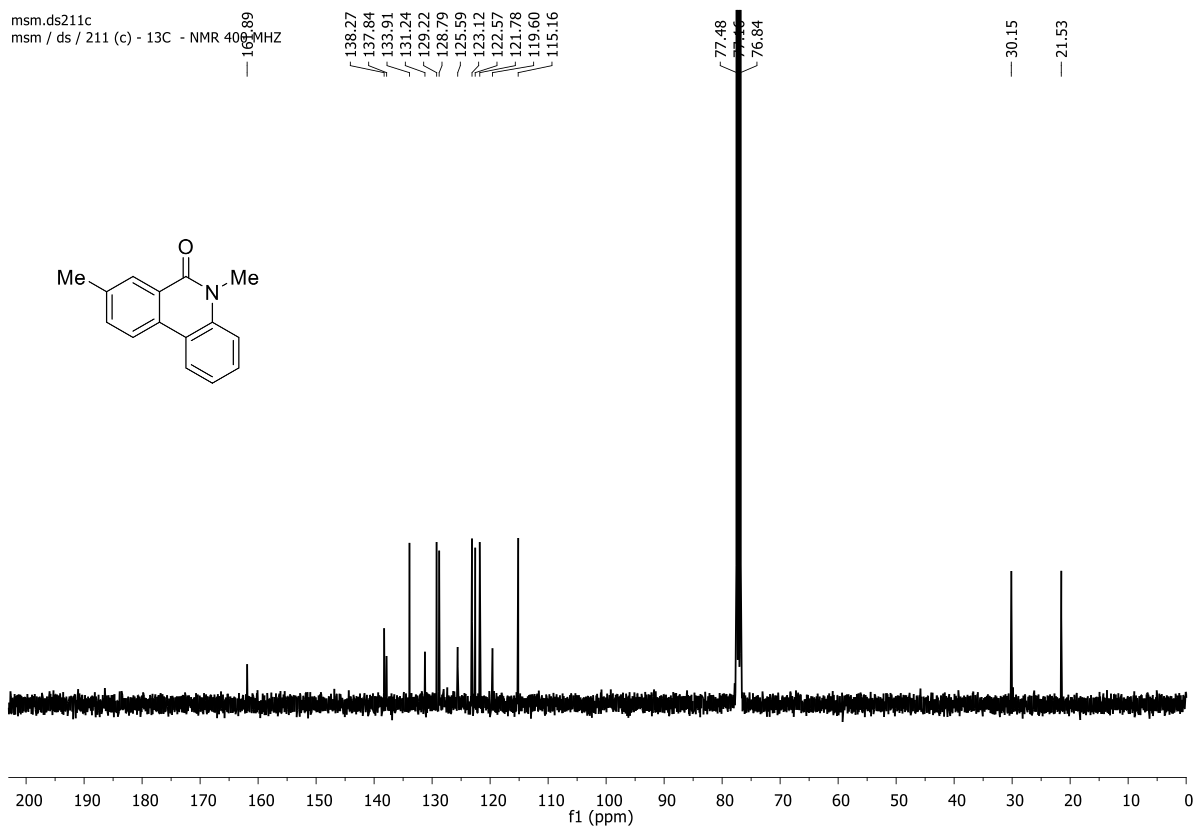

^{1}H (400 MHz) and ^{13}C (126 MHz) NMR of **5c** in $CDCl_3$

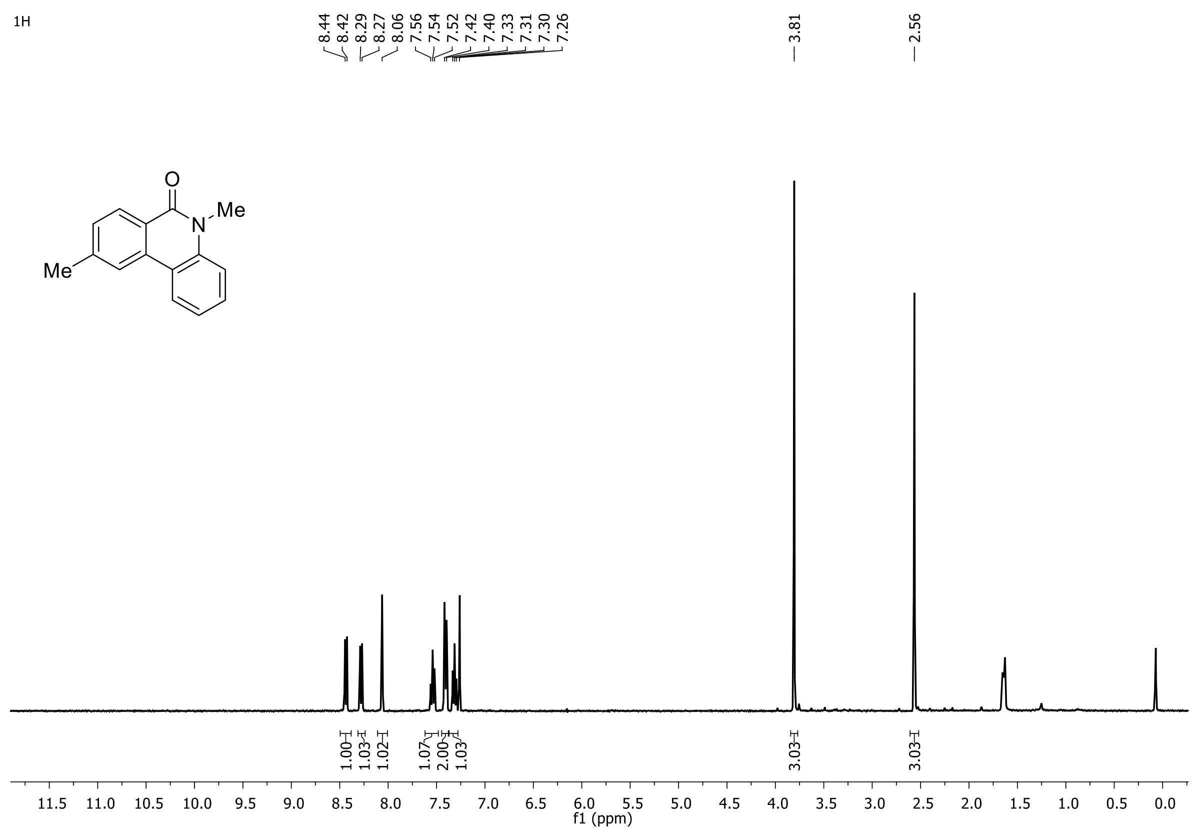

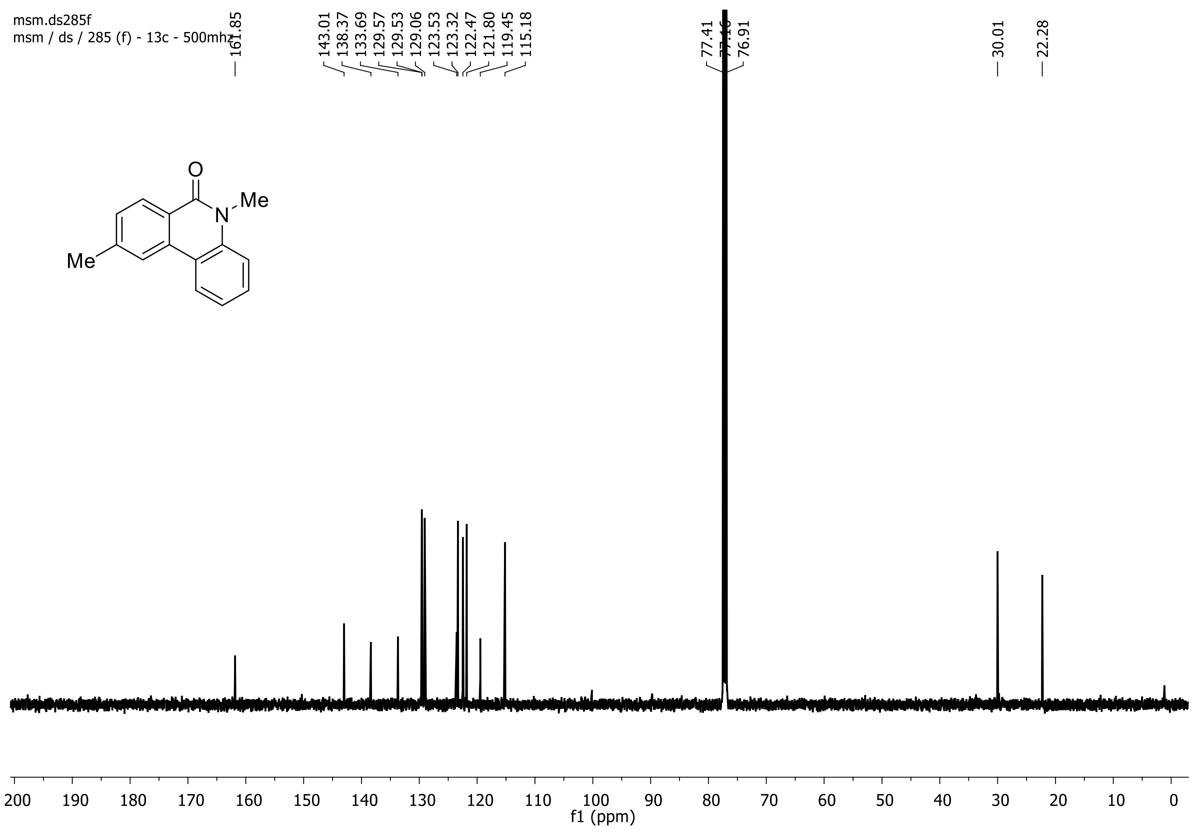

^{1}H (400 MHz) and ^{13}C (126 MHz) NMR of **5d** in $CDCl_3$

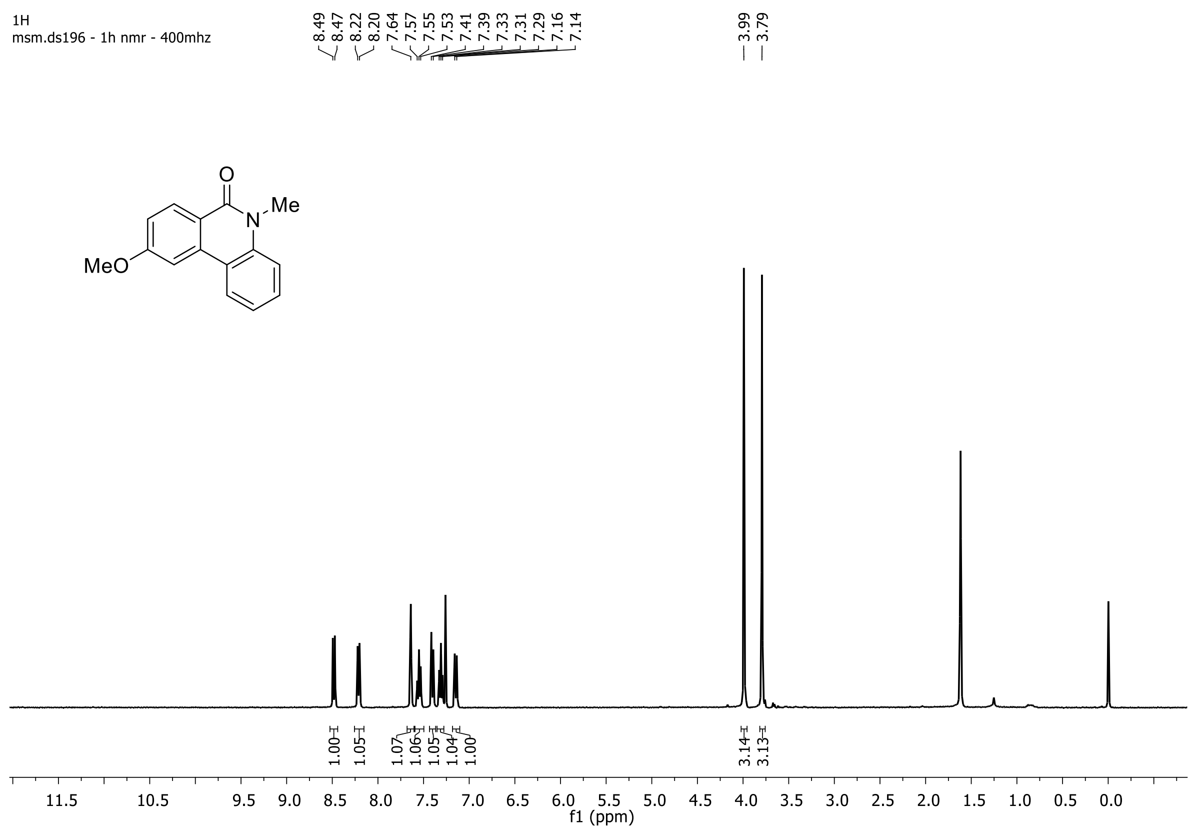

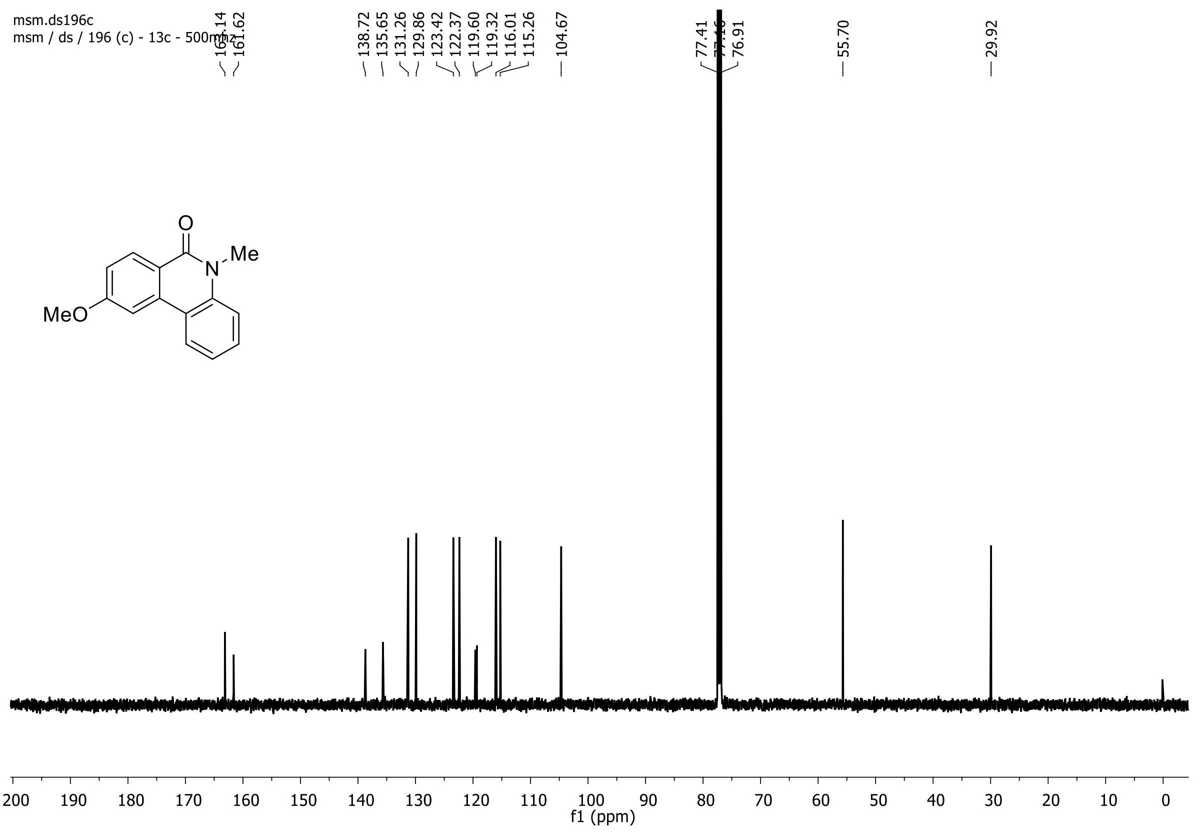

^{1}H (400 MHz) and ^{13}C (126 MHz) NMR of **5e** in $CDCl_3$

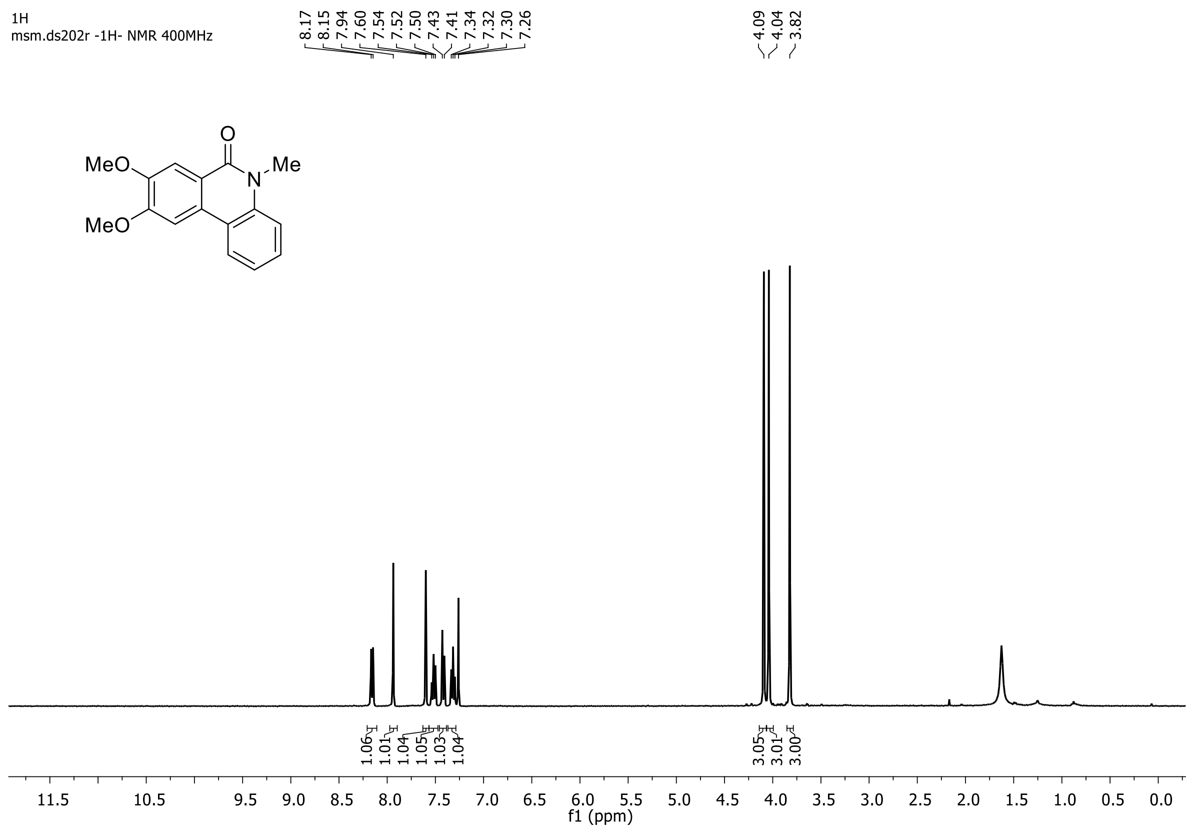

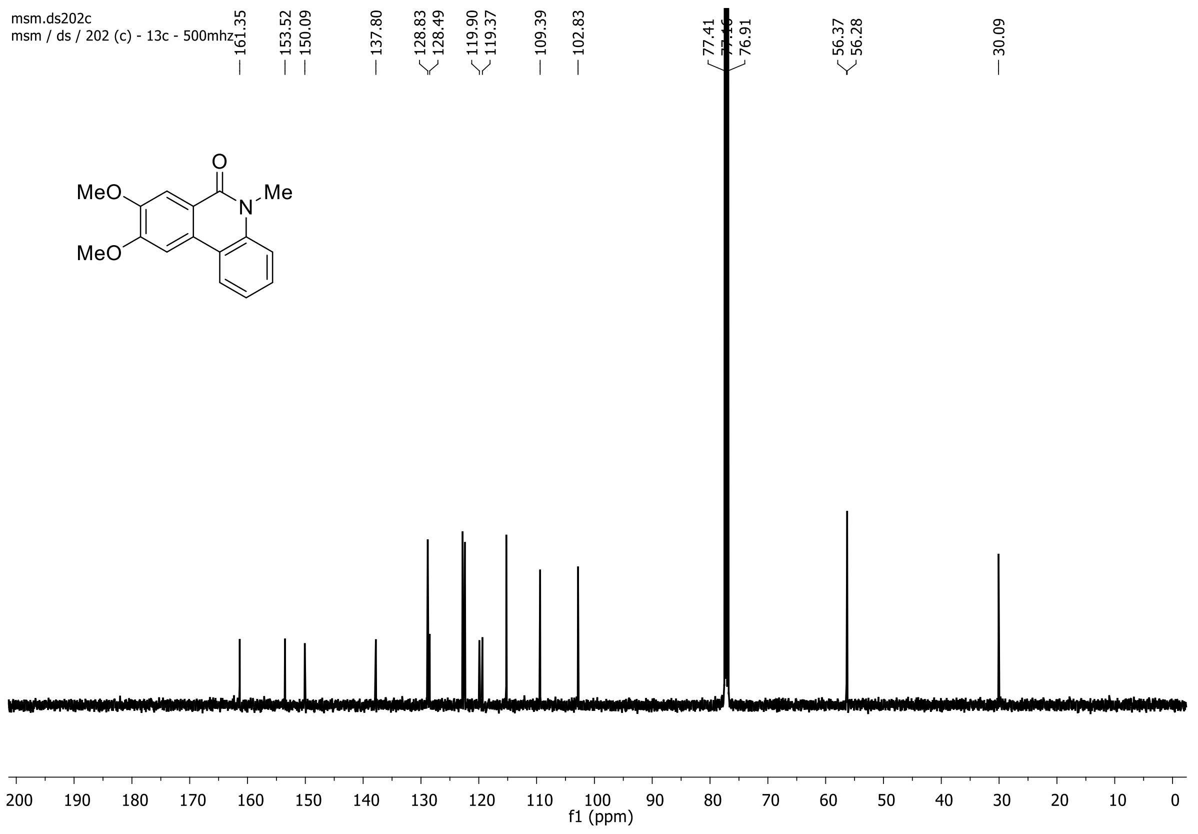

^{1}H (400 MHz) and ^{13}C (101 MHz) NMR of **5f** in $CDCl_3$

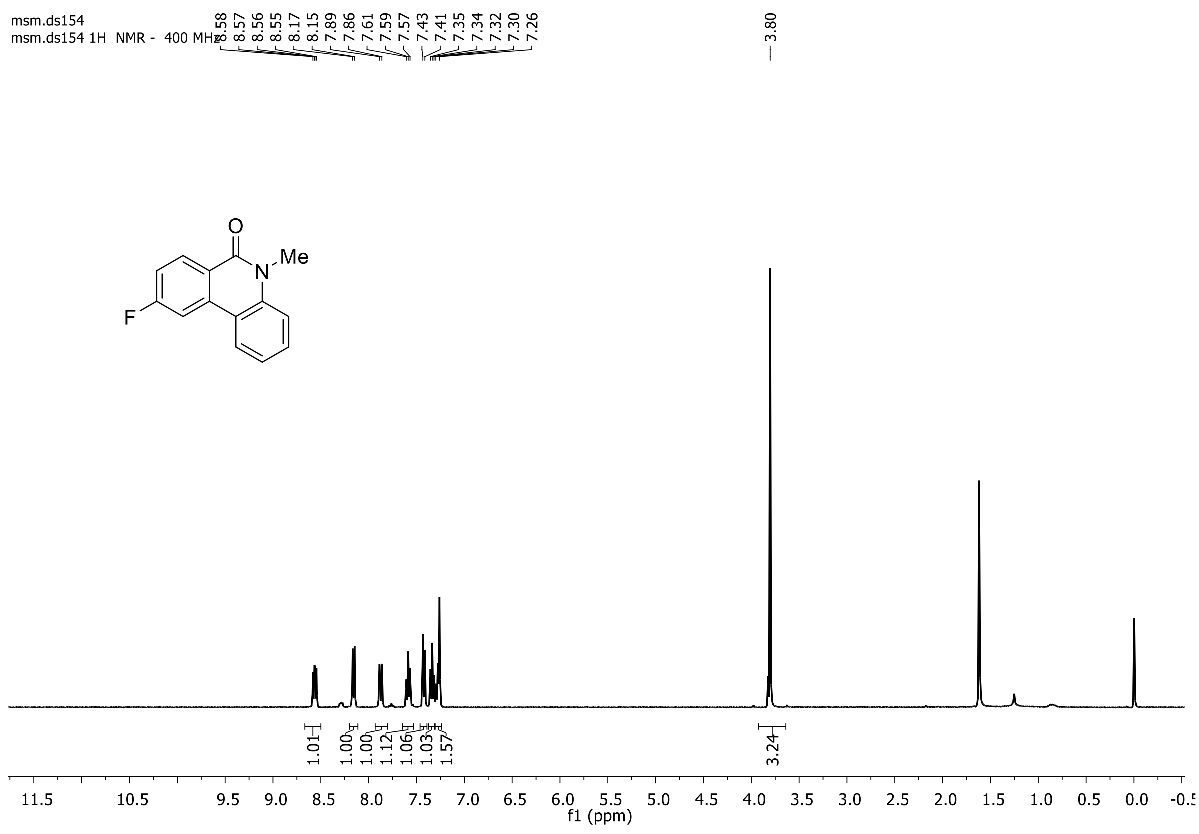

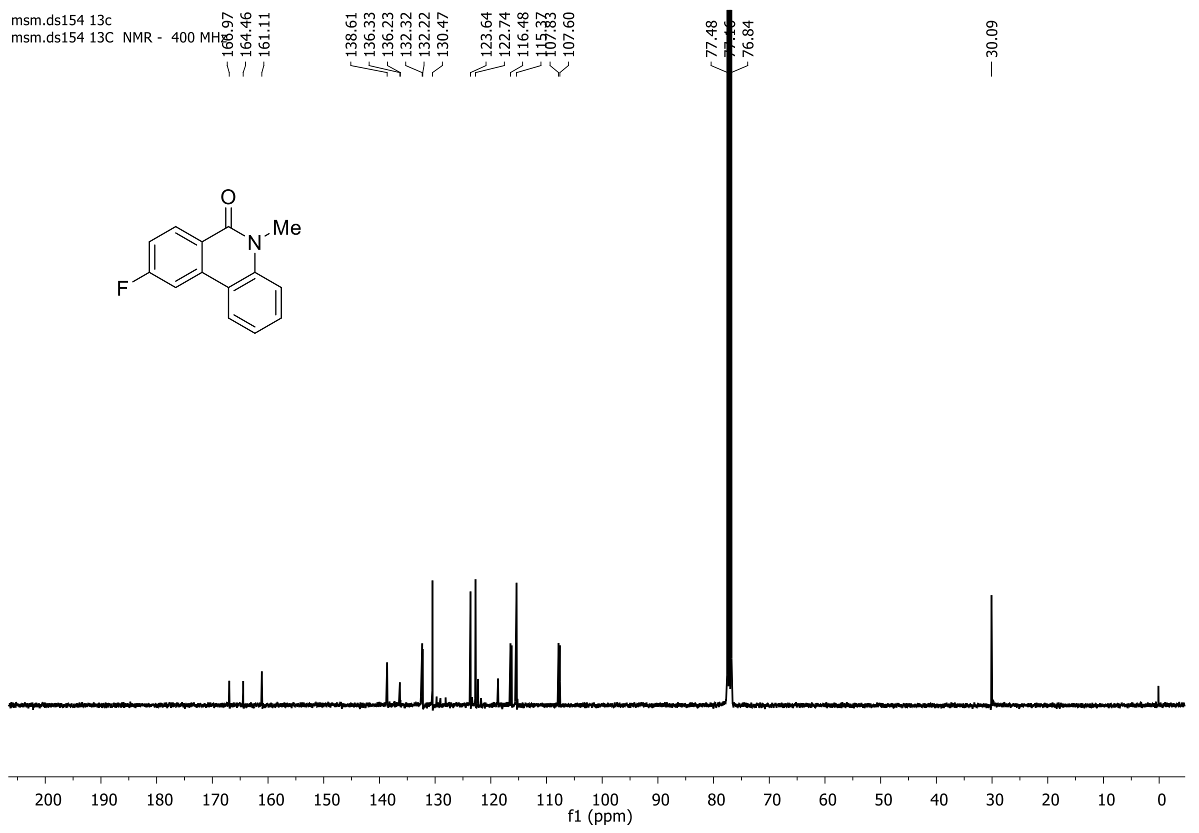

^{19}F NMR (471 MHz) of **5f** in $CDCl_3$

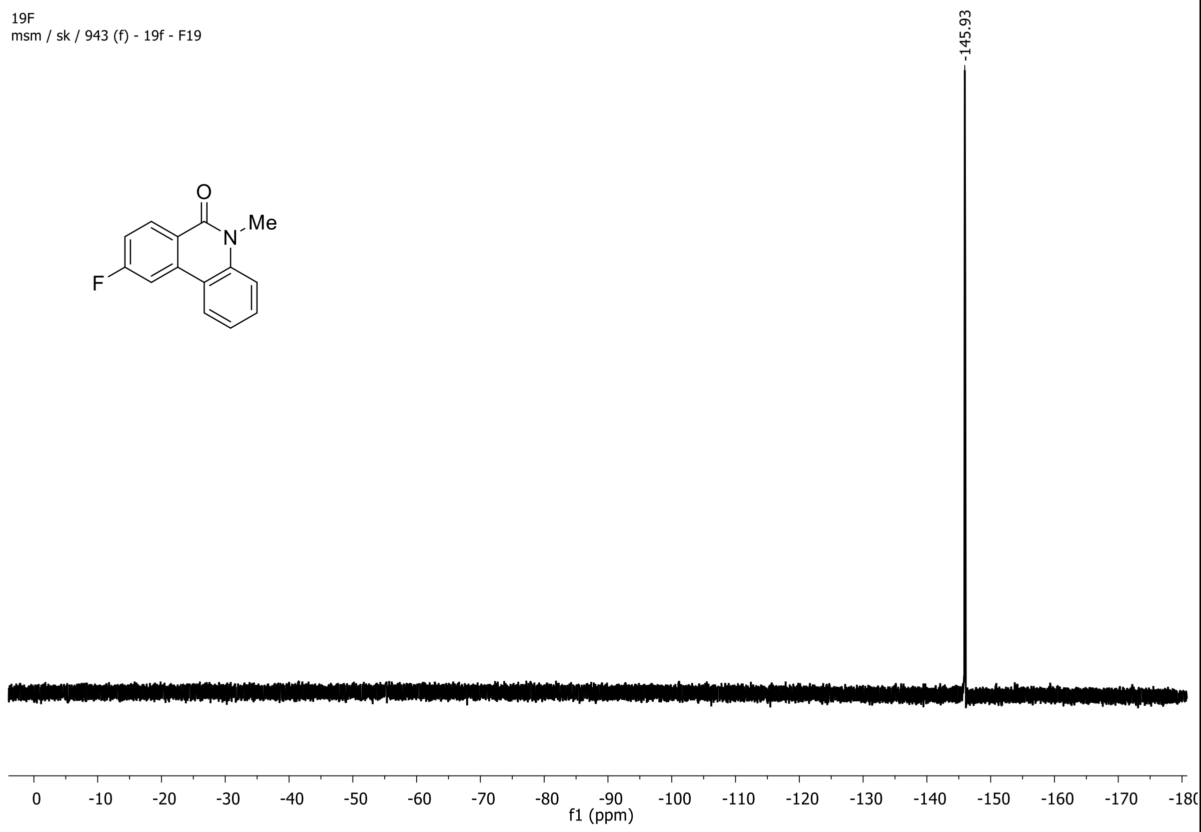

^{1}H (400 MHz) and ^{13}C (101 MHz) NMR of **5g** in $CDCl_3$

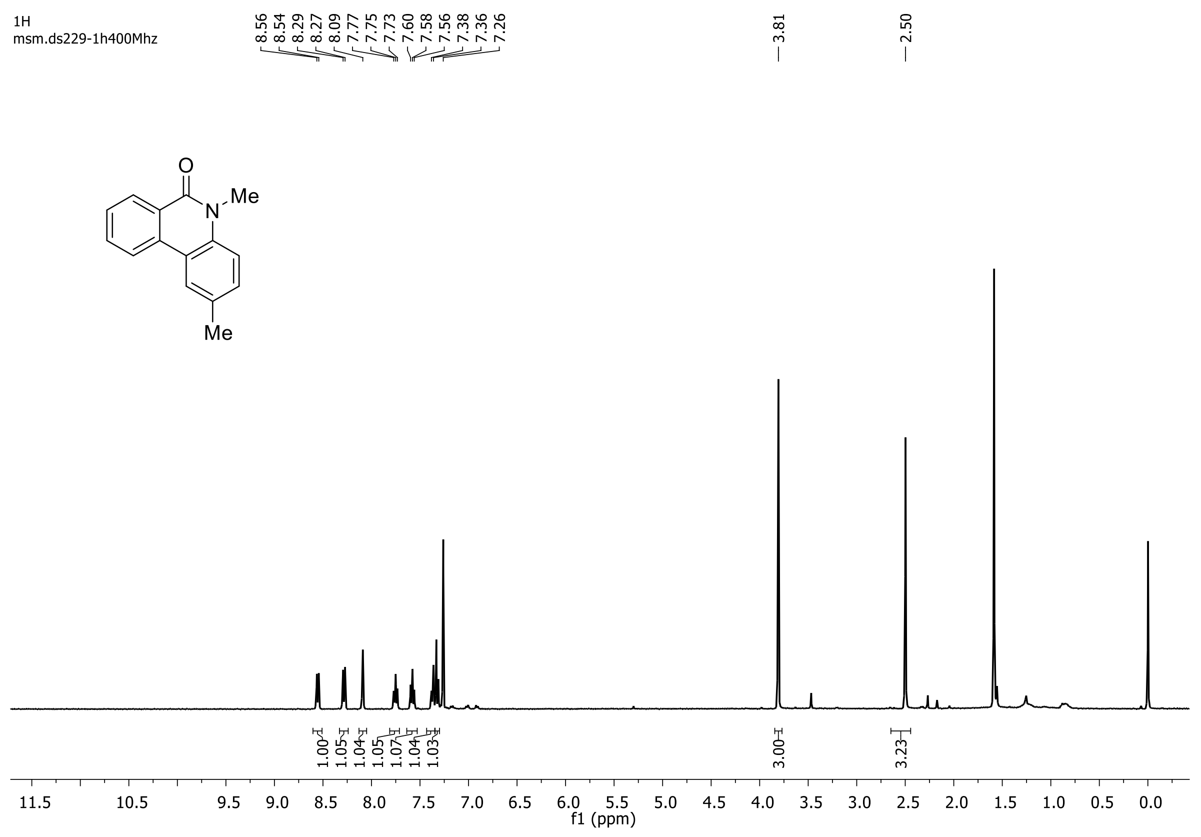

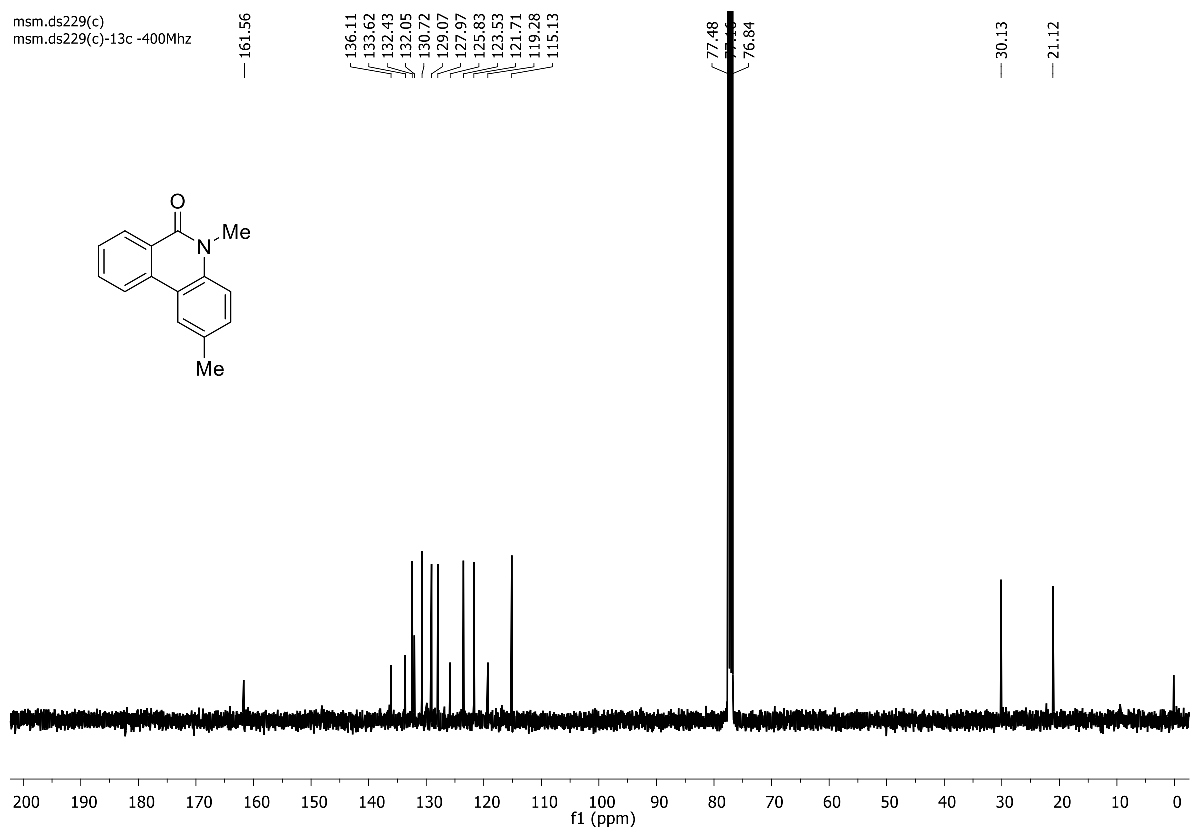

^{1}H (400 MHz) and ^{13}C (126 MHz) NMR of **5h** in $CDCl_3$

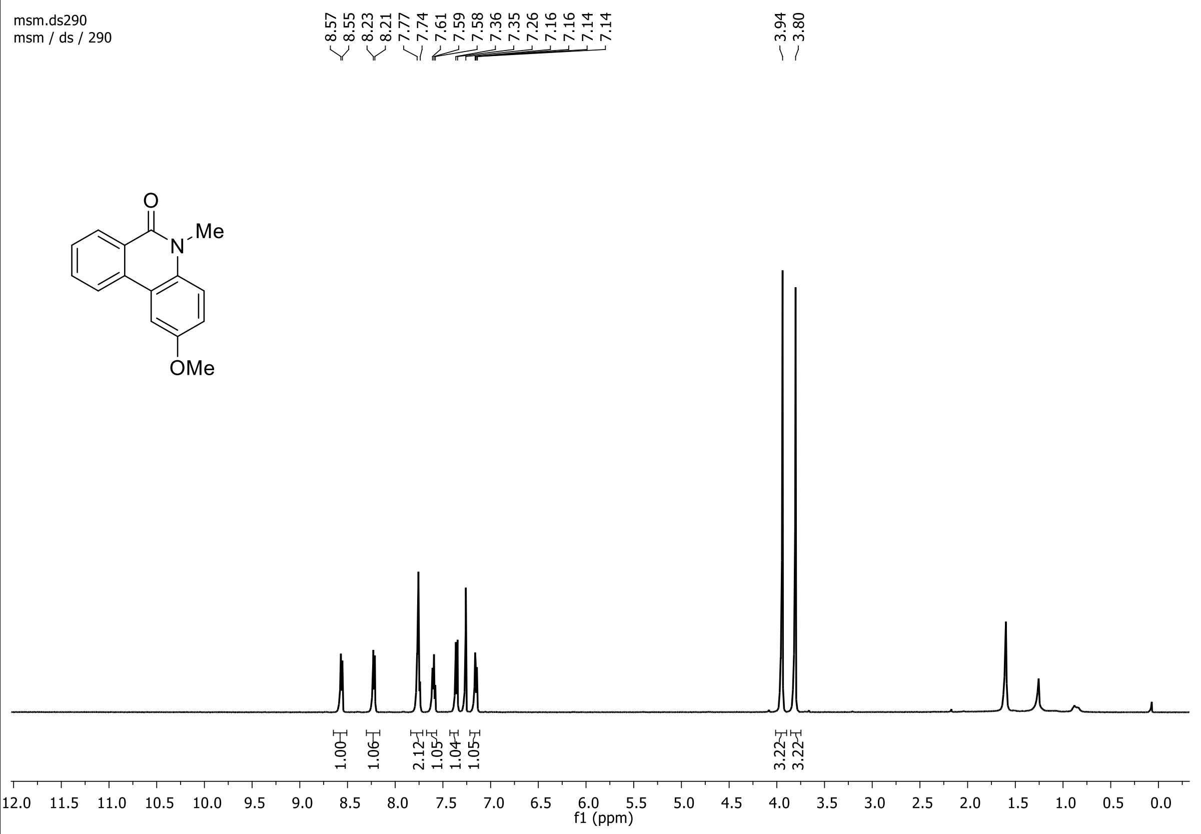

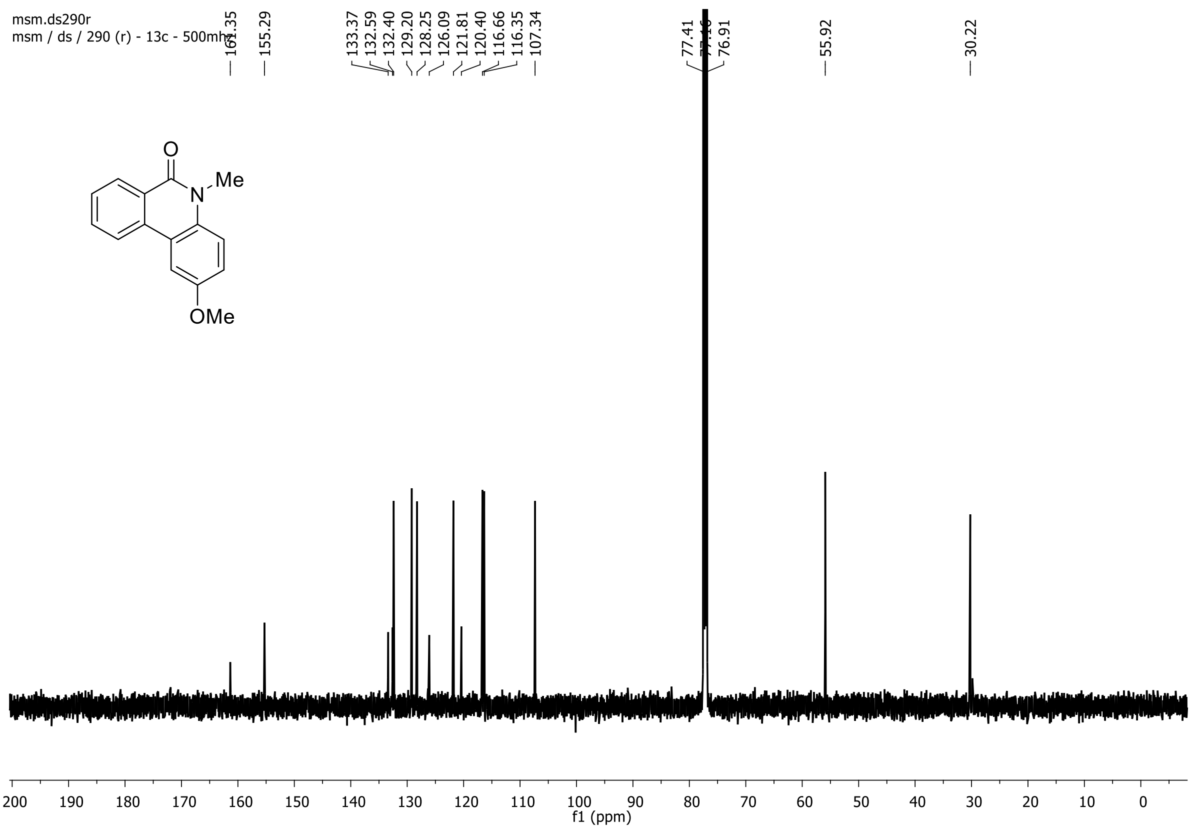

^{1}H (400 MHz) and ^{13}C (101 MHz) NMR of **5i** in $CDCl_3$

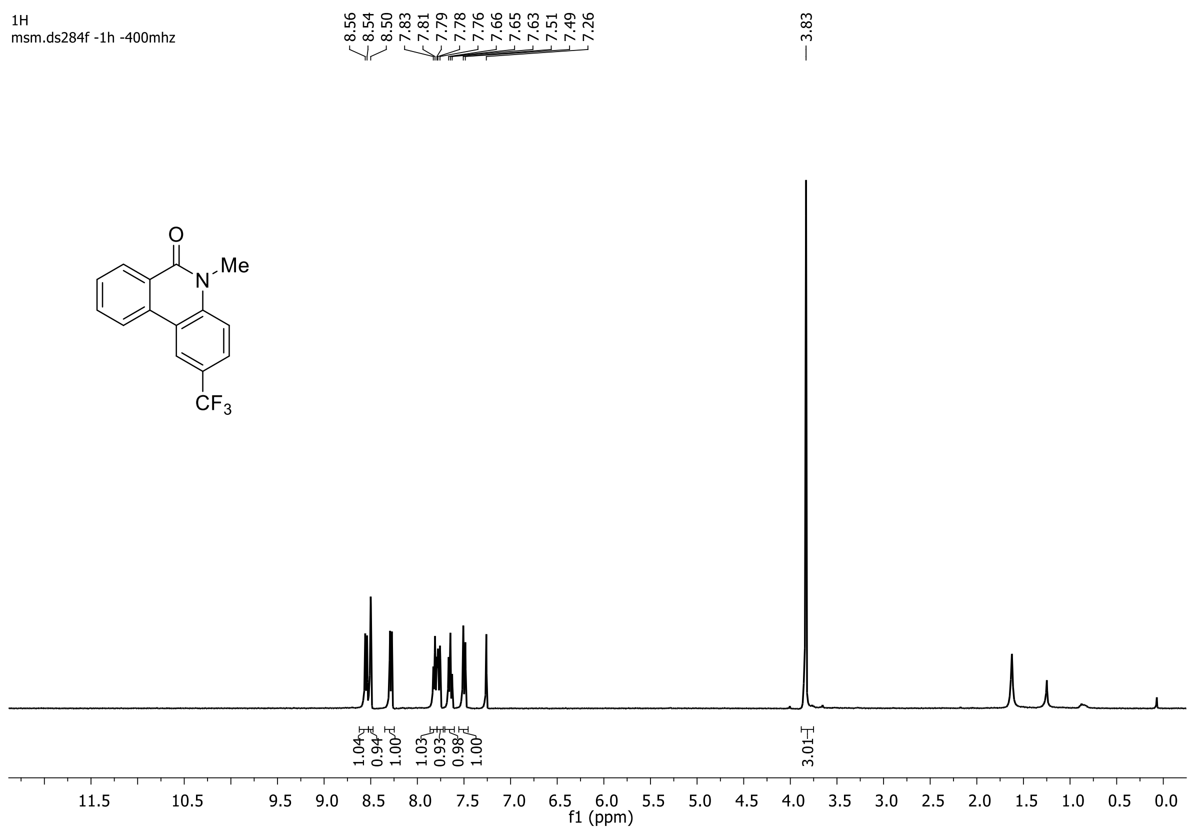

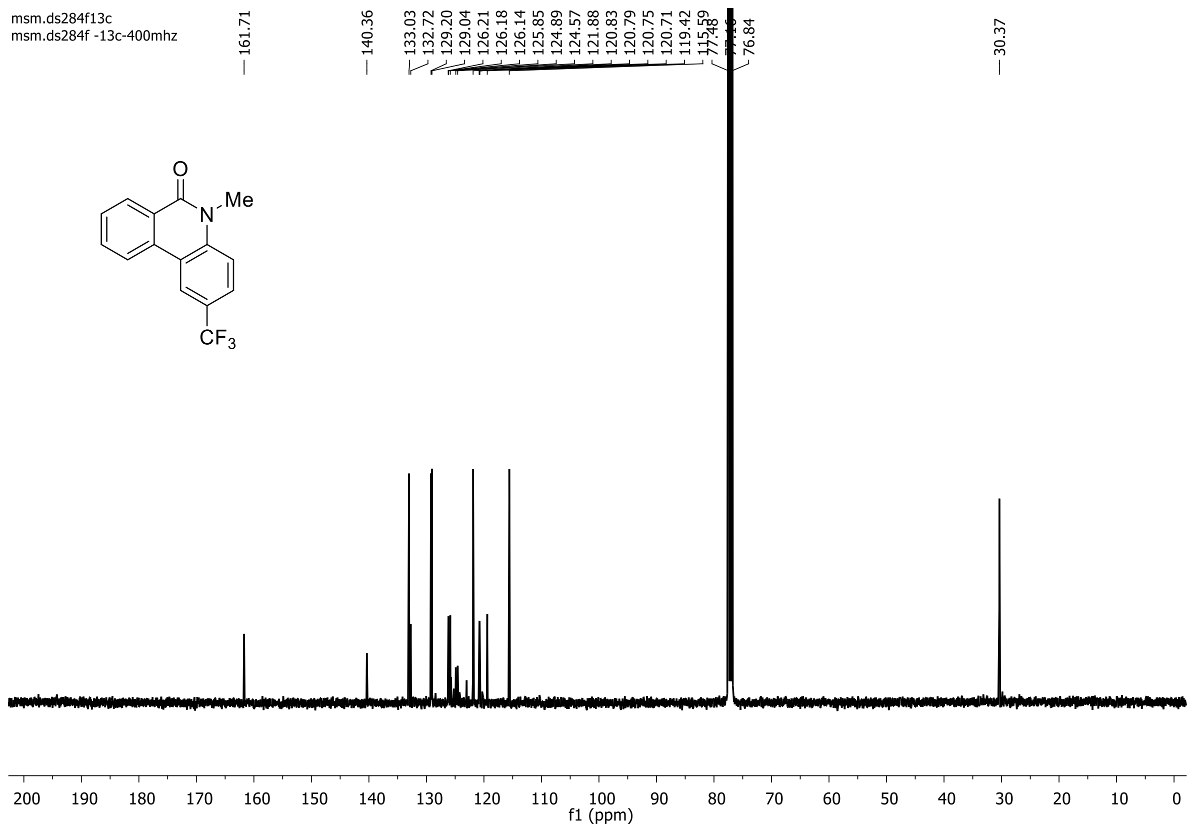

^{19}F (471 MHz) NMR of **5i** in $CDCl_3$

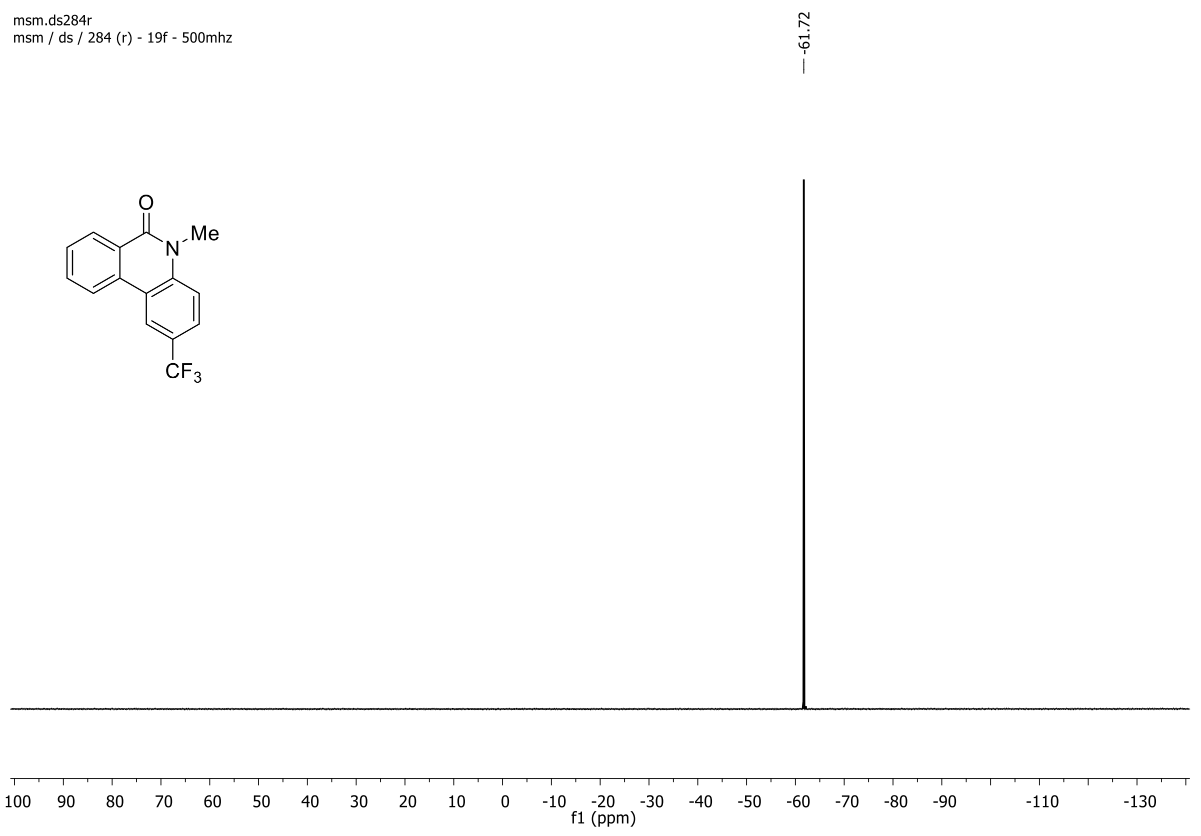

^{1}H (500 MHz) and ^{13}C (126 MHz) NMR of ***5'a*** in $CDCl_3$

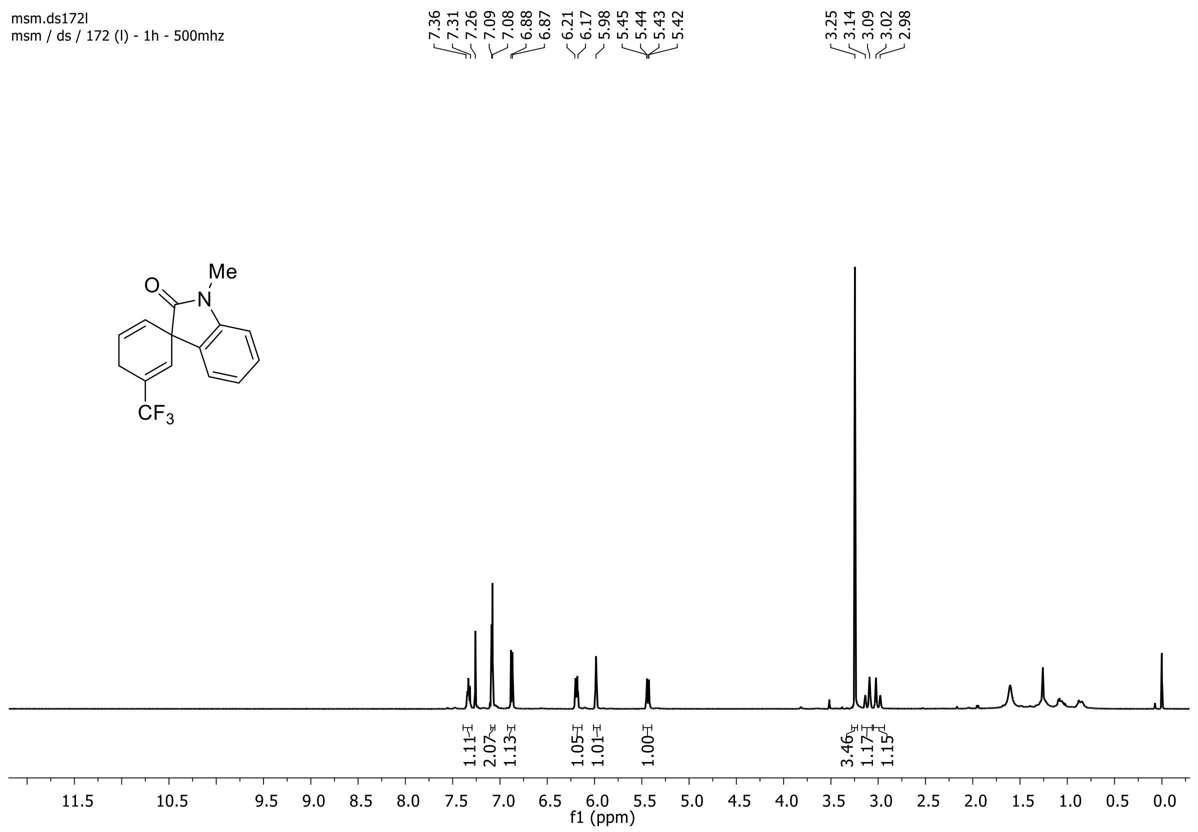

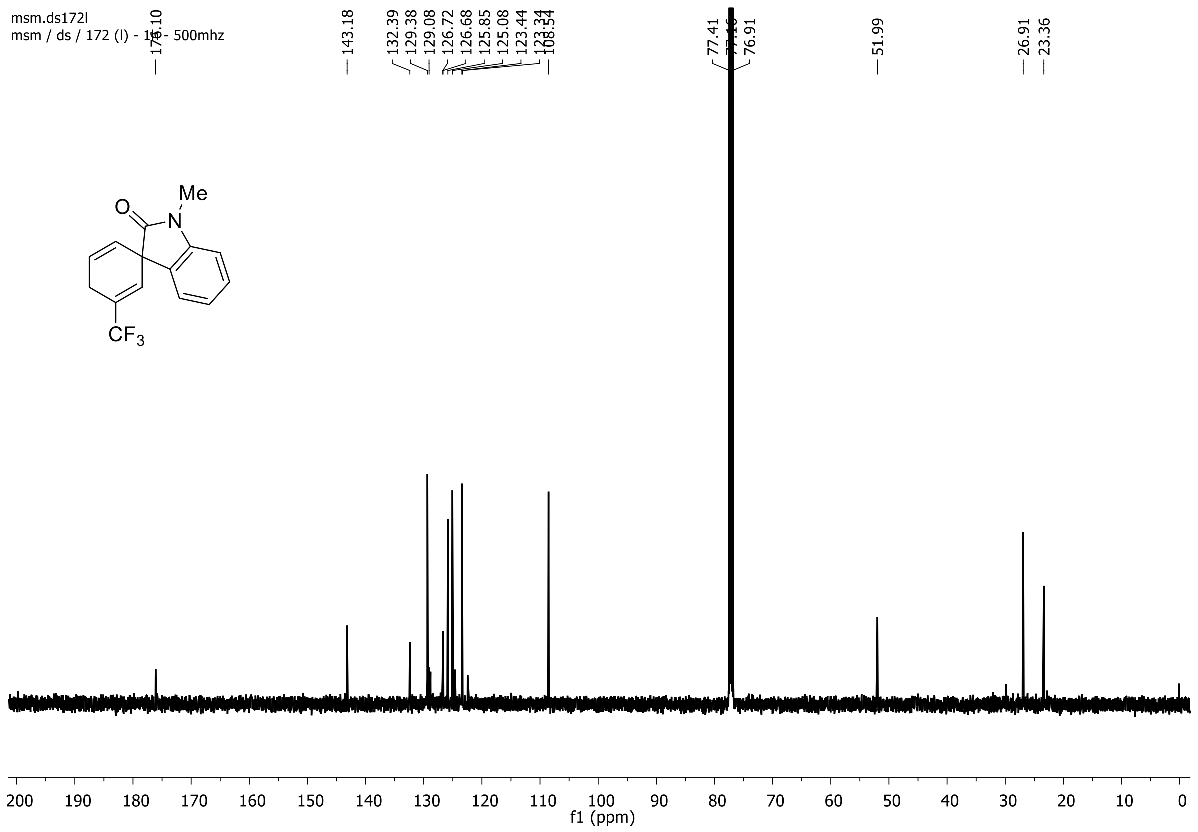

^{19}F (471 MHz) NMR of ***5'a*** in $CDCl_3$

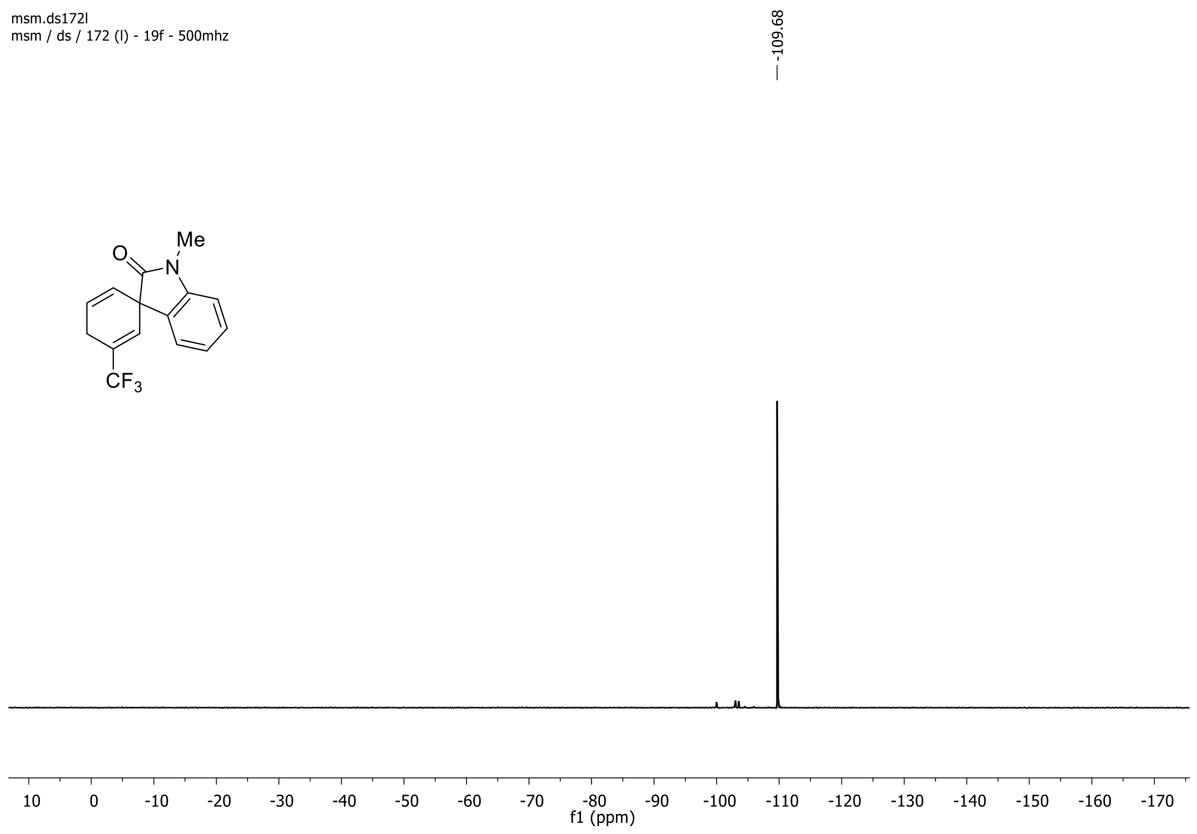

^{1}H (500 MHz) and ^{13}C (126 MHz) NMR of ***5'b*** in $CDCl_3$

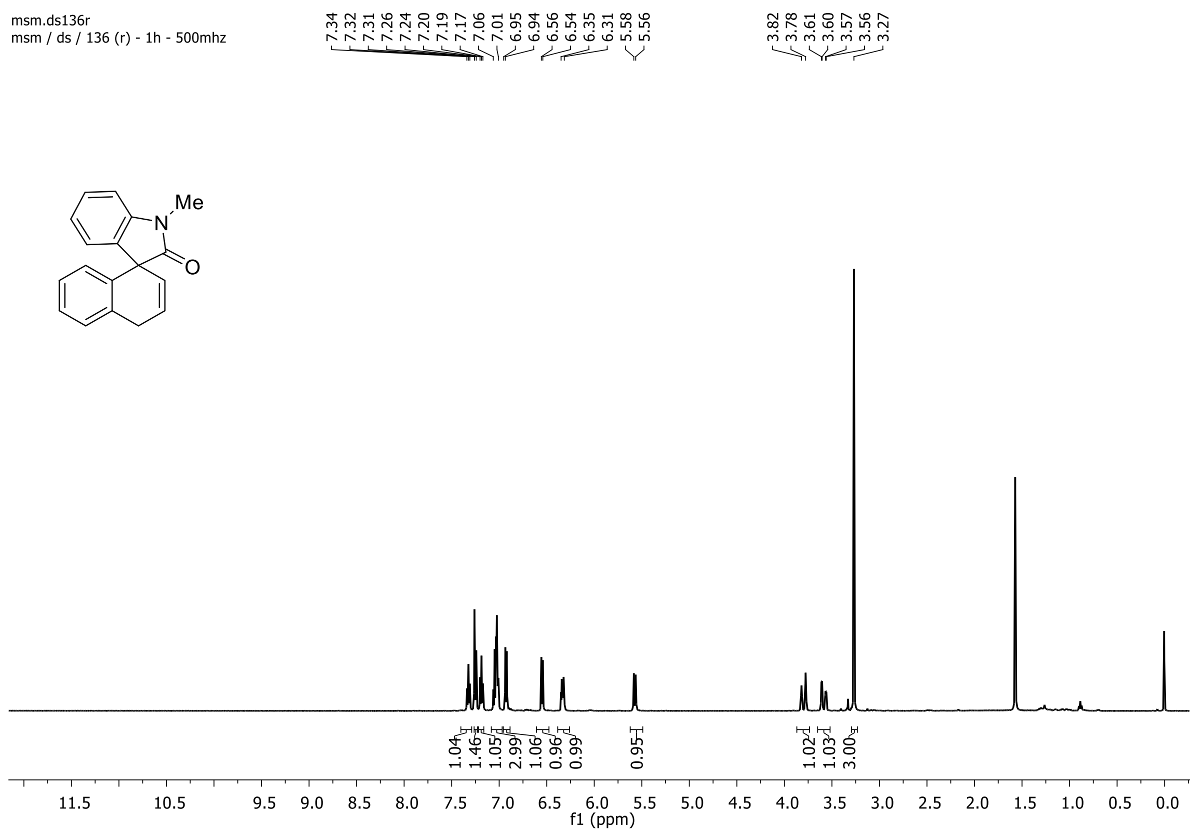

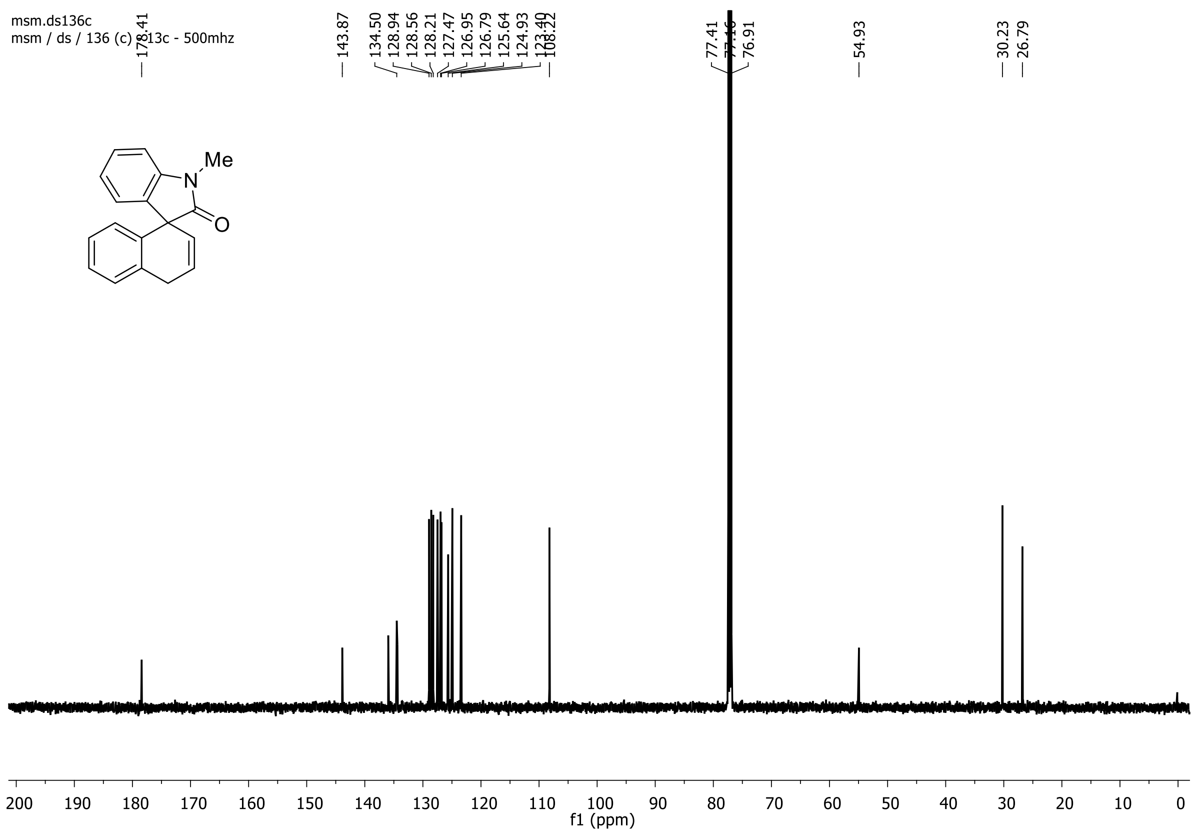

^{1}H (400 MHz) NMR of **7a** in $CDCl_3$

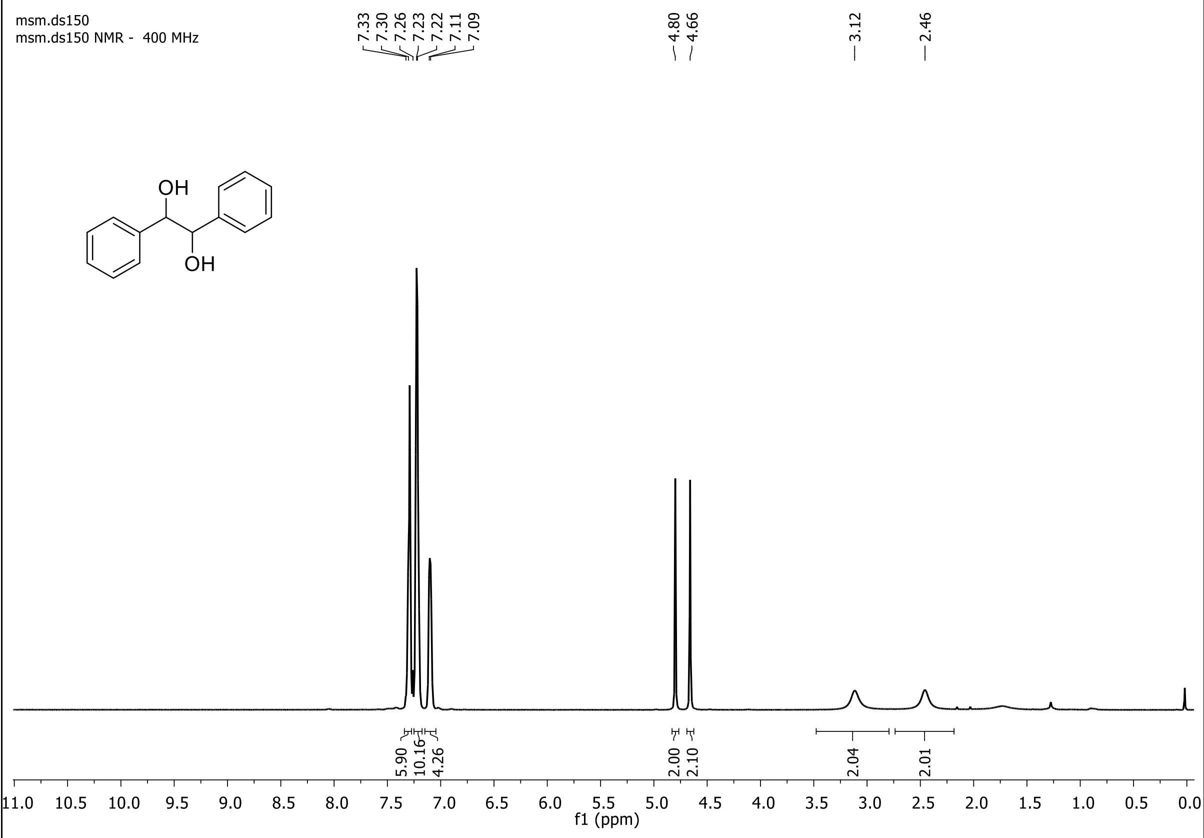

^{1}H (400 MHz) NMR of **7b** in $CDCl_3$

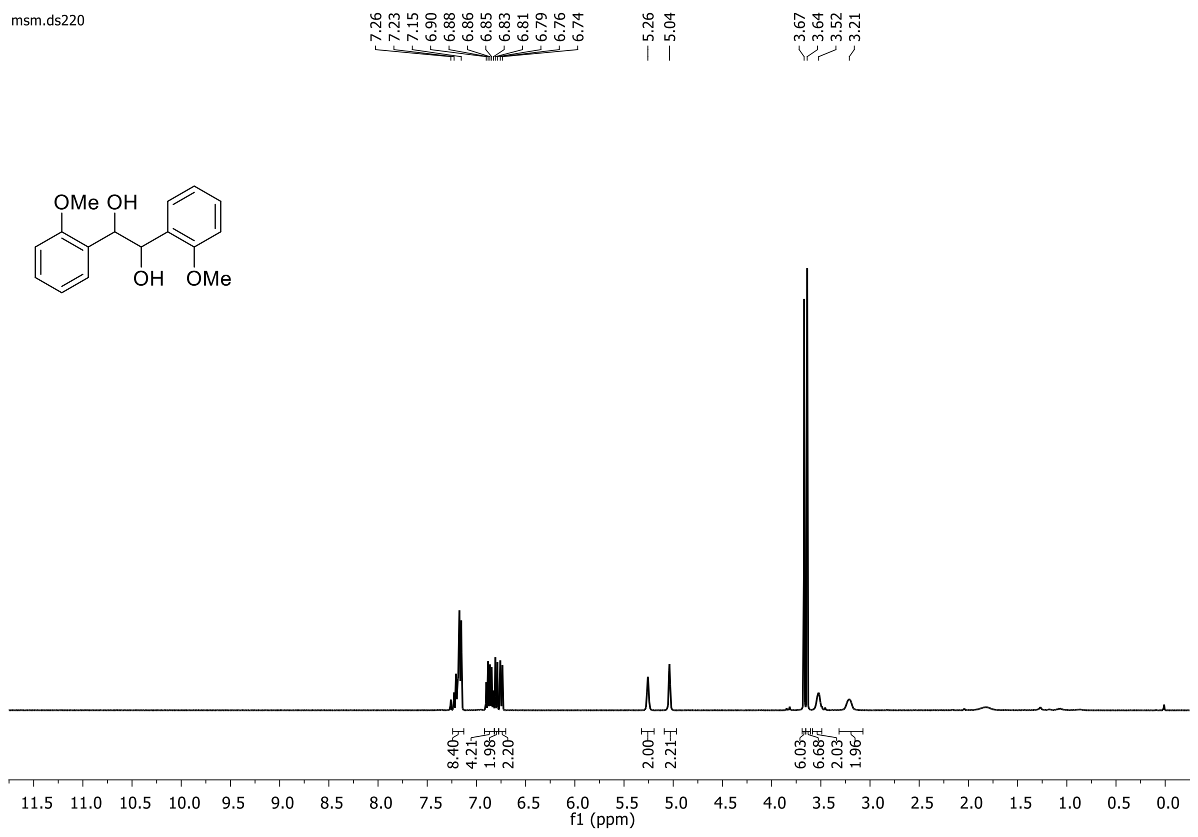

^{1}H (400 MHz) NMR of **7c** in $CDCl_3$

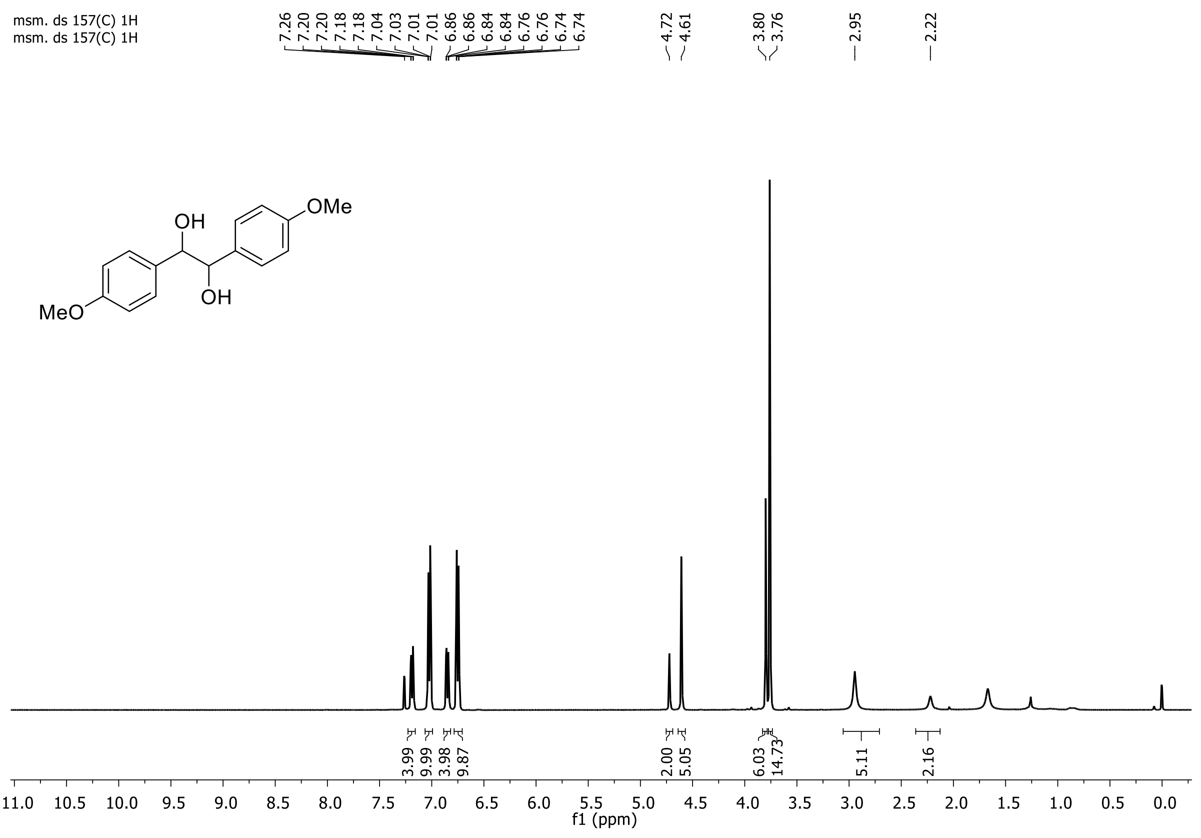

^{1}H (400 MHz) and ^{13}C (126 MHz) NMR of **7d** in $CDCl_3$

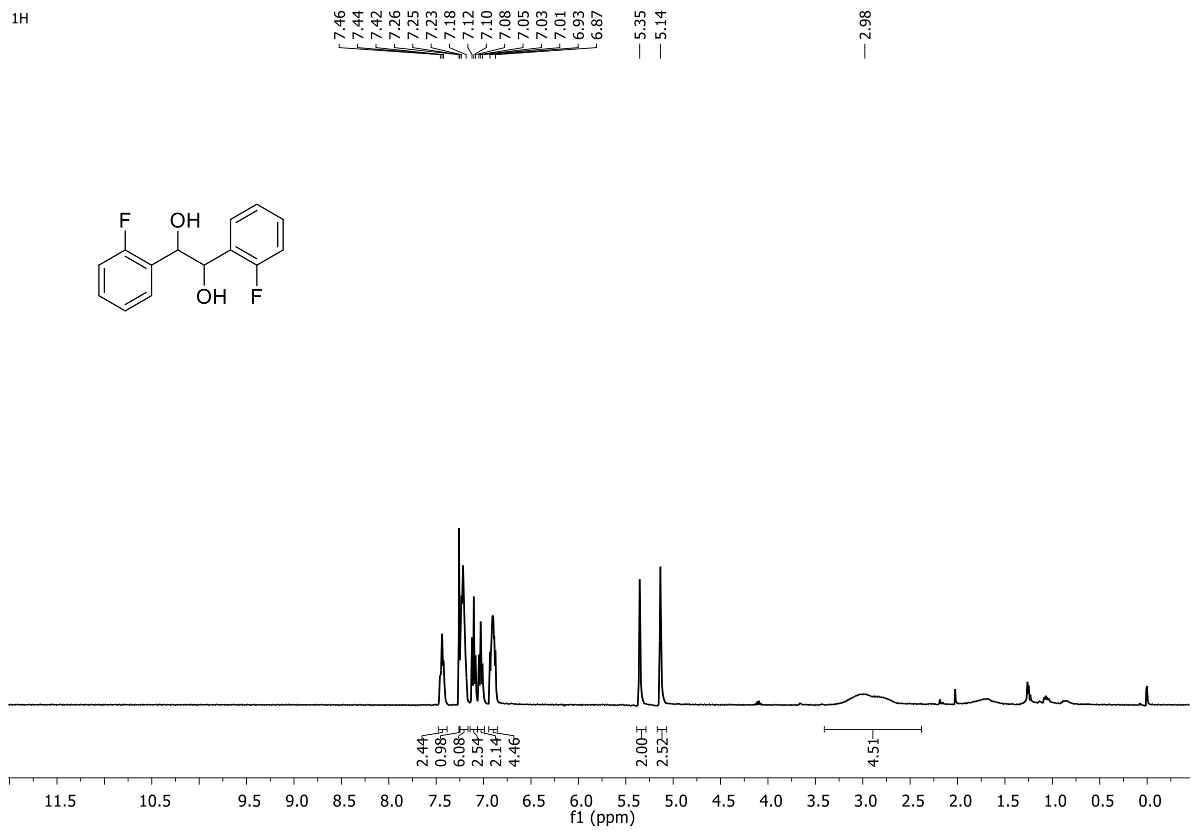

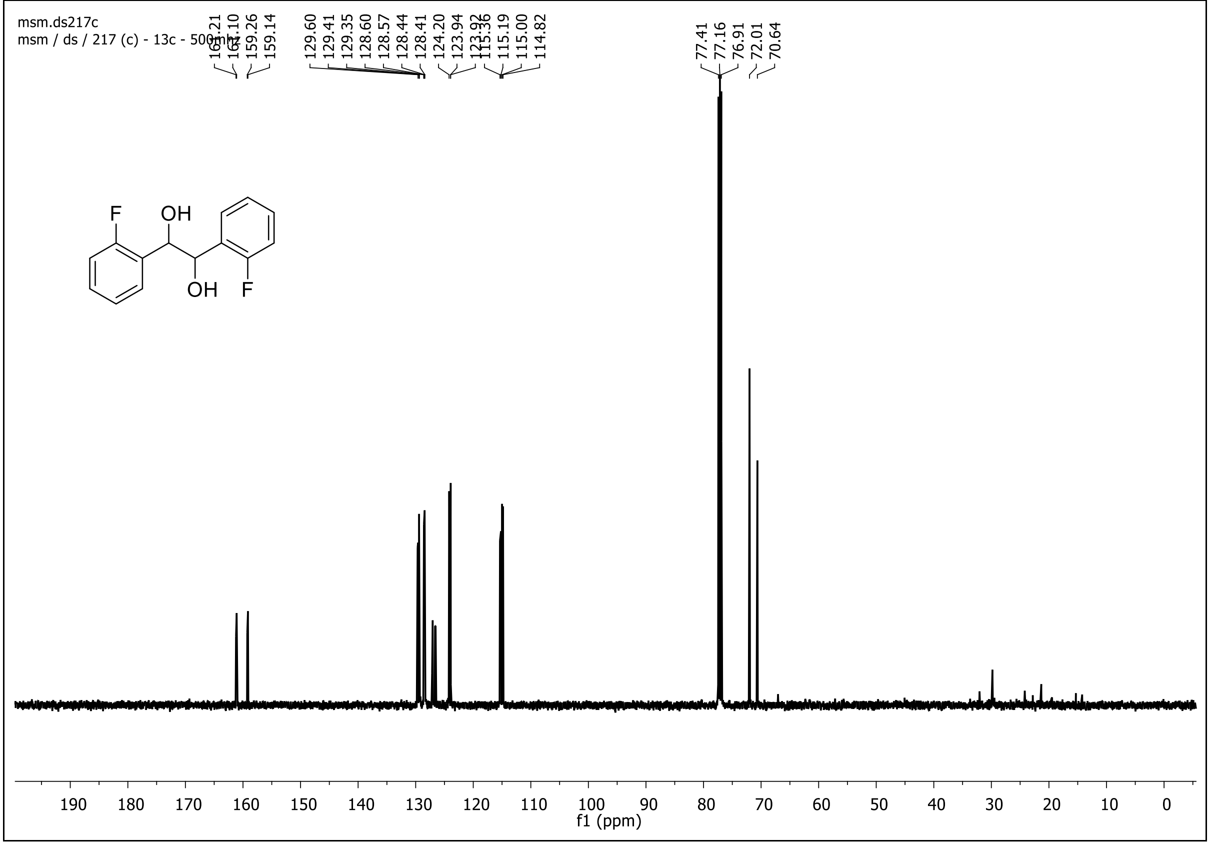

^{19}F (471 MHz) NMR of **7d** in $CDCl_3$

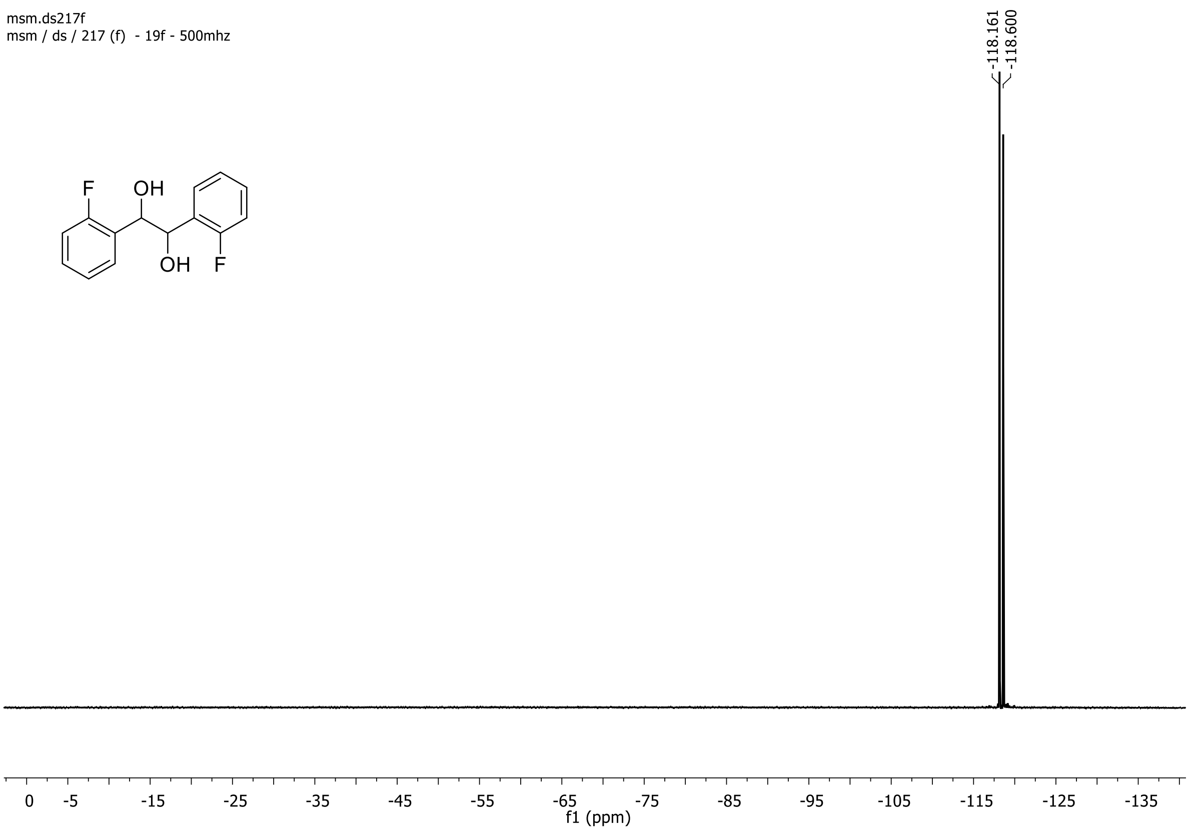

^{1}H (500 MHz) NMR of **7e** in $CDCl_3$

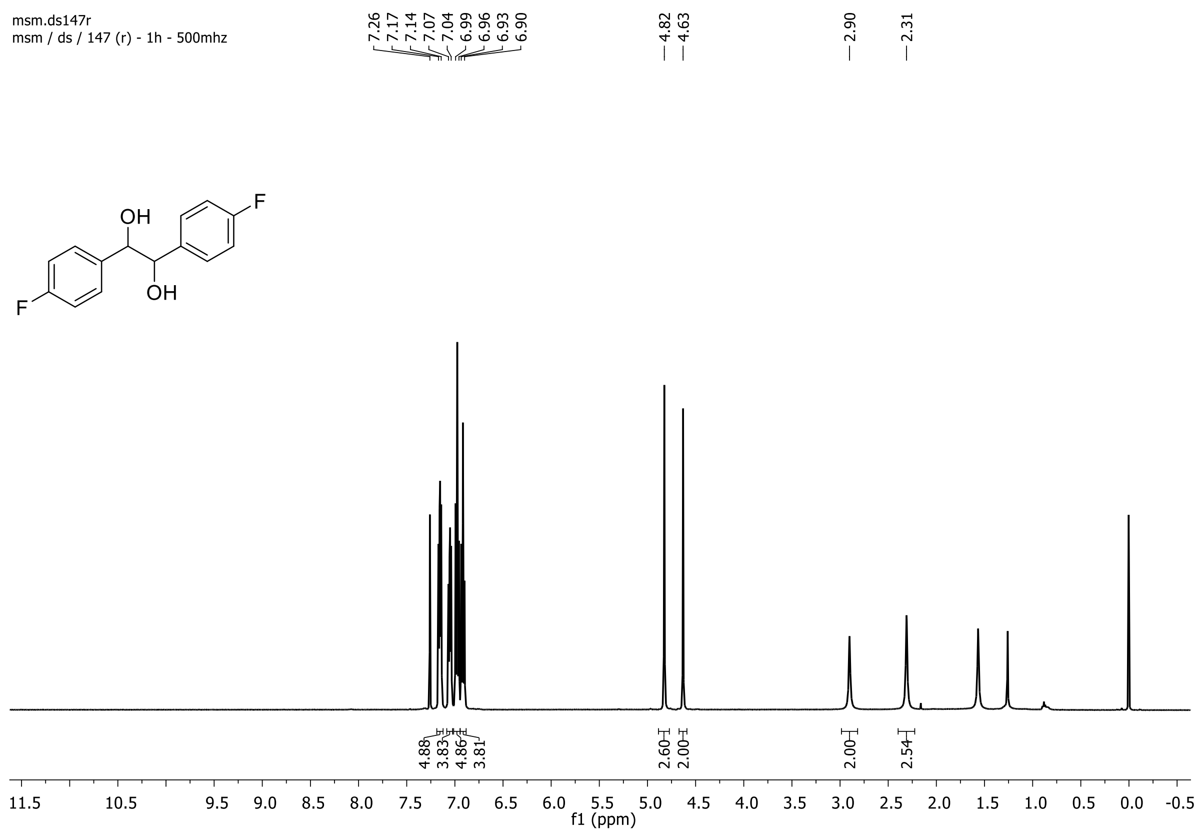

^{1}H (400 MHz) NMR of **7f** in DMSO-d_6

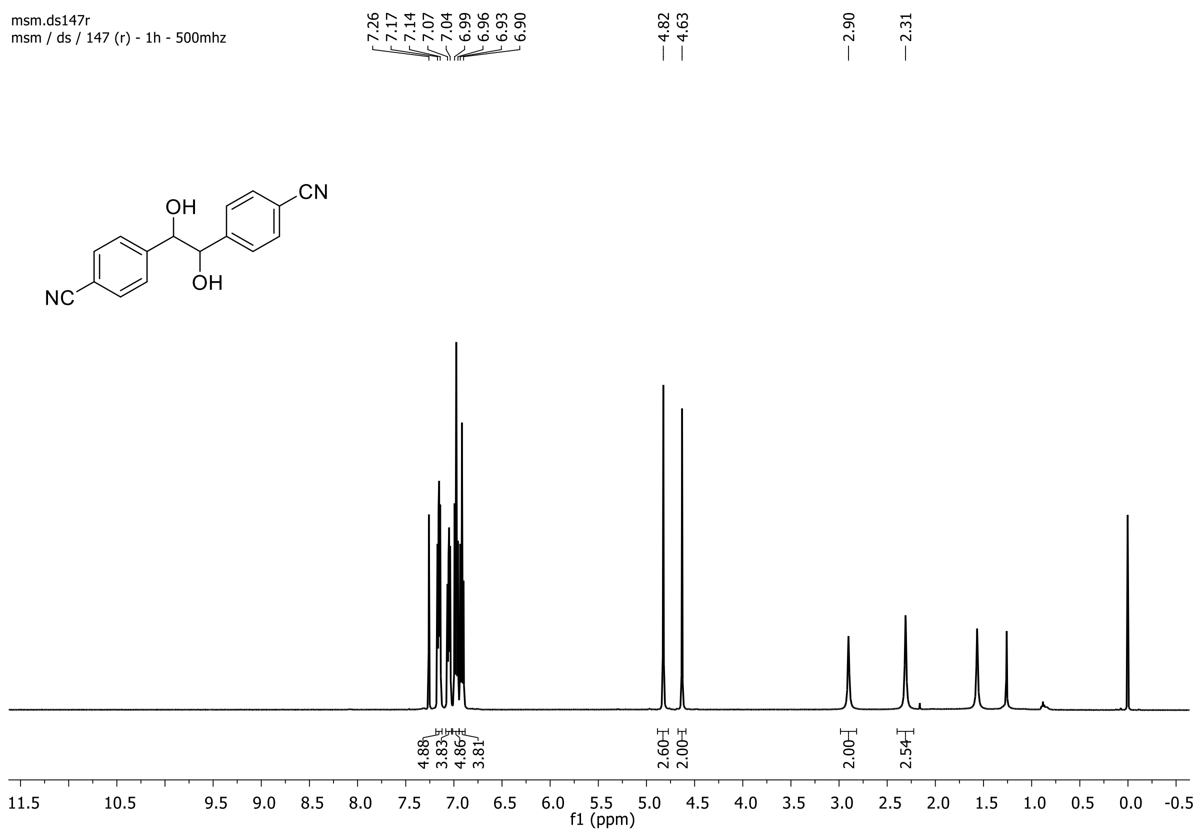

^{1}H (400 MHz) NMR of **7g** in $CDCl_3$

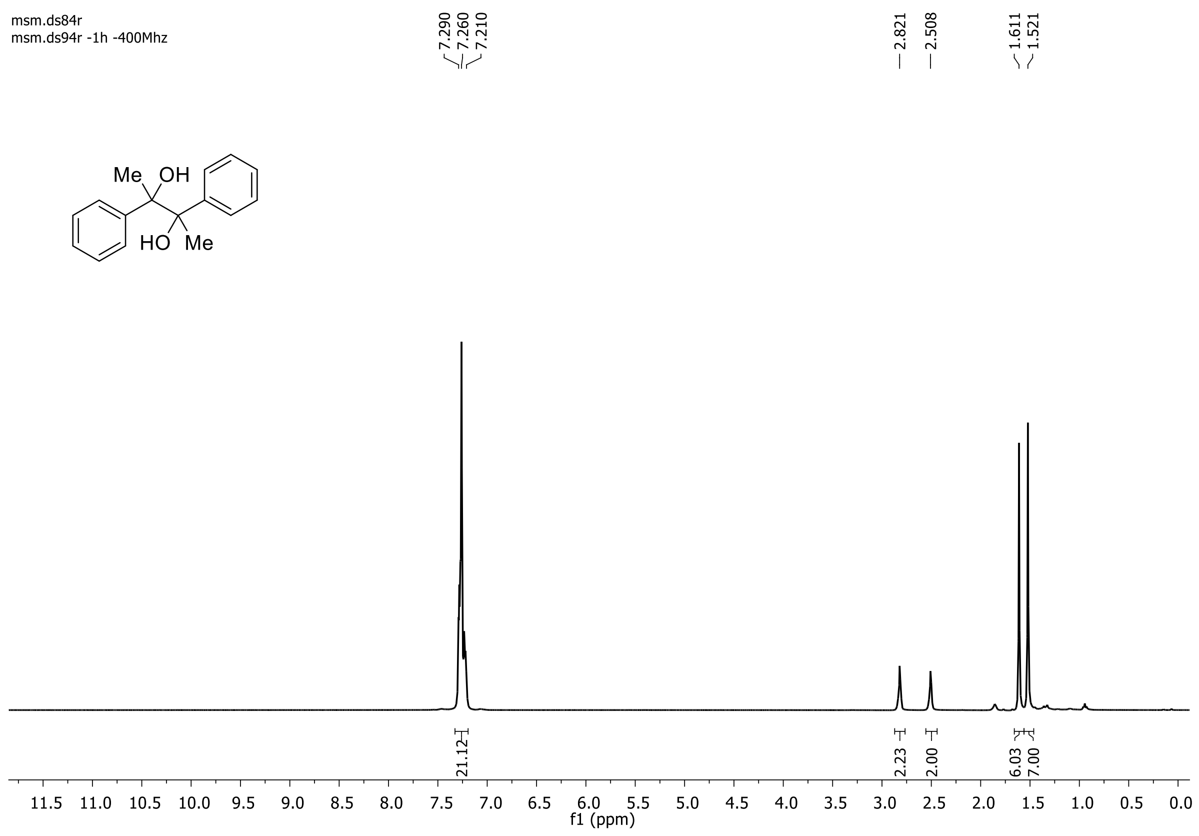

^{1}H (400 MHz) NMR of **7h** in $CDCl_3$

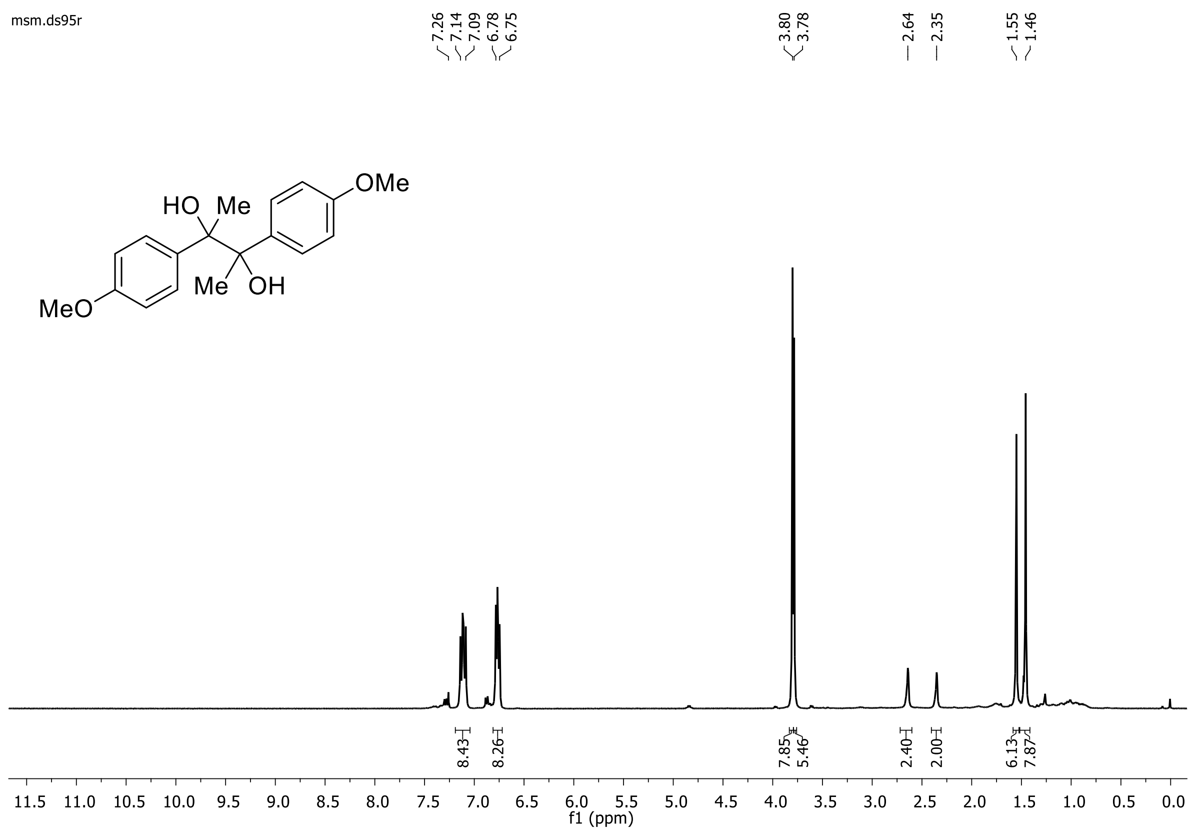

^{1}H (500 MHz) NMR of **7i** in $CDCl_3$

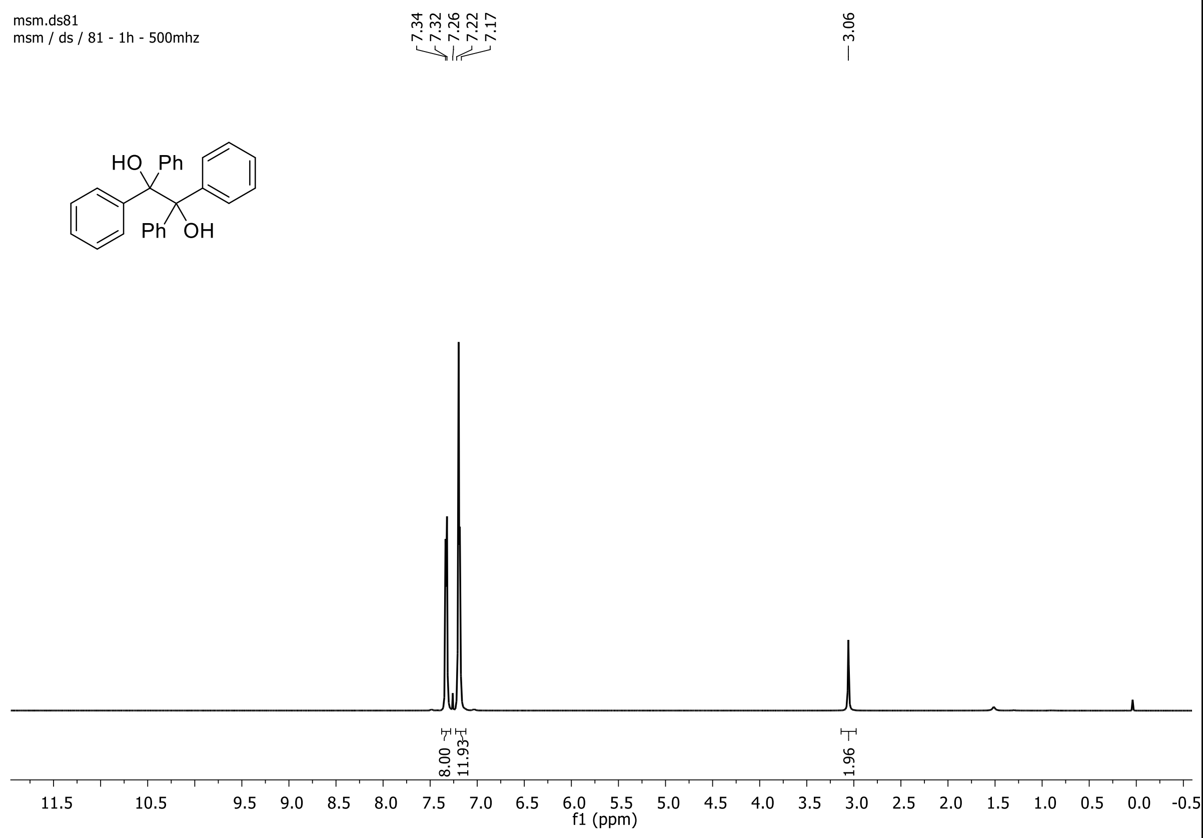

^{1}H (400 MHz) NMR of **9a** in $CDCl_3$

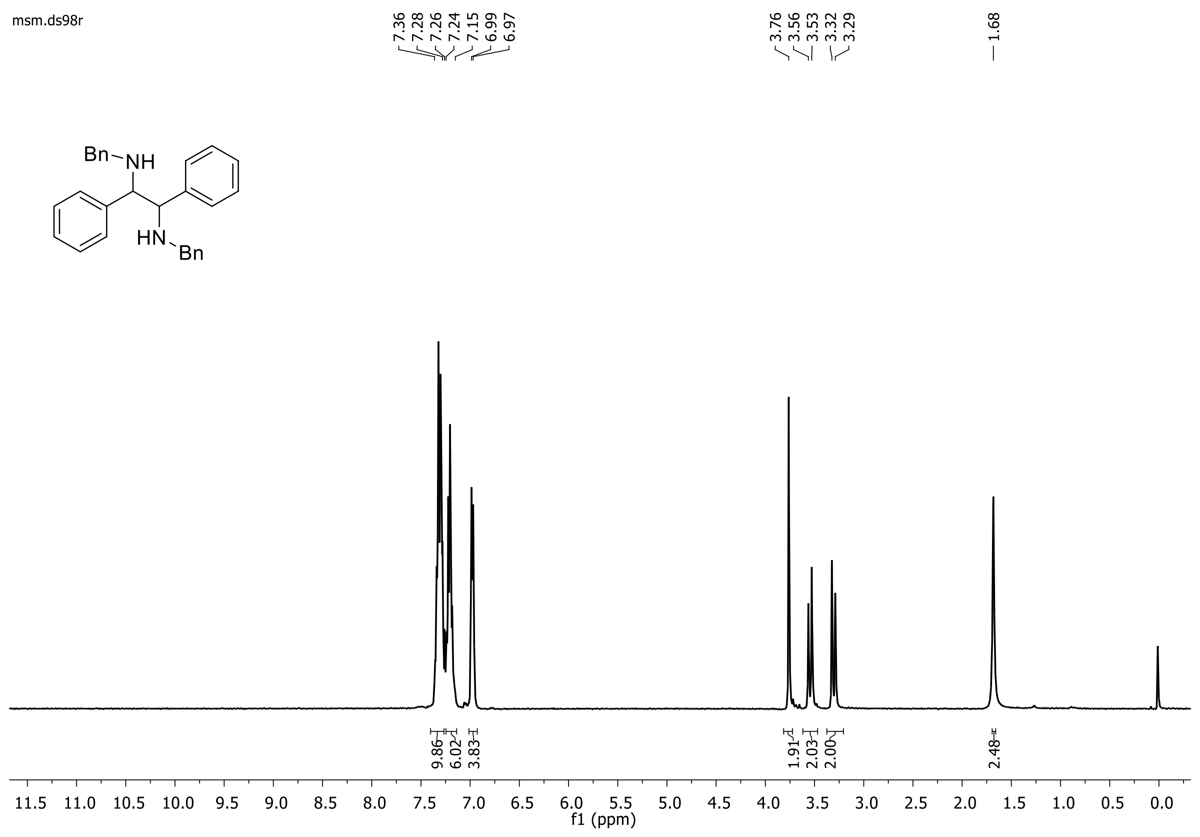

^{1}H (400 MHz) NMR of **9b** in $CDCl_3$

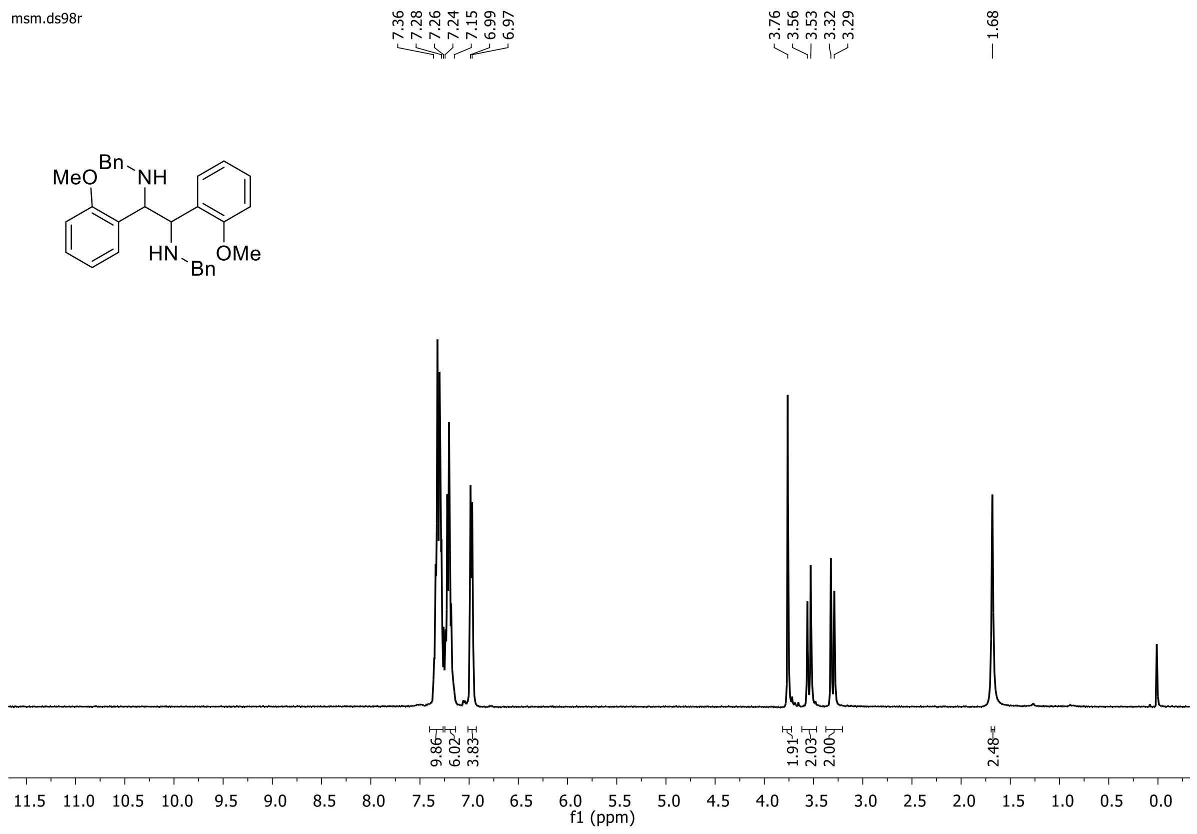

^{1}H (500 MHz) NMR of **9c** in $CDCl_3$

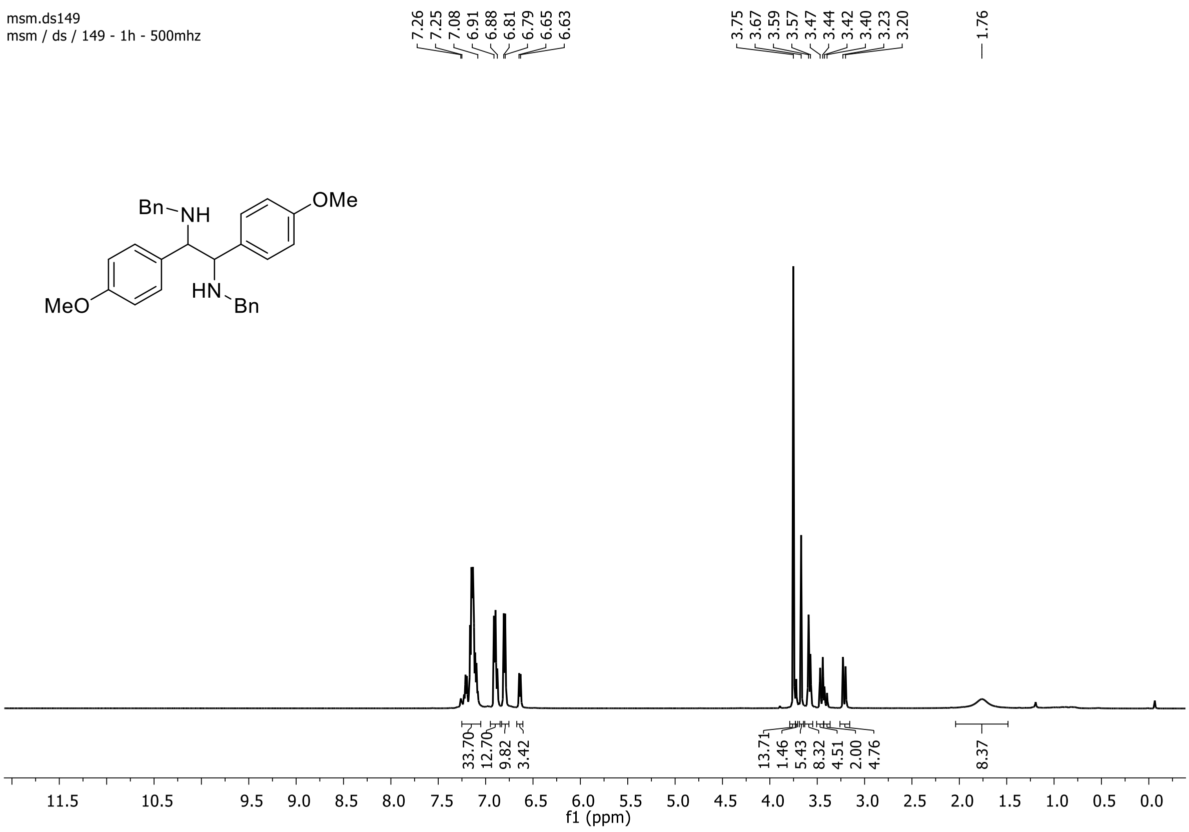

^{1}H (400 MHz) NMR of **9d** in $CDCl_3$

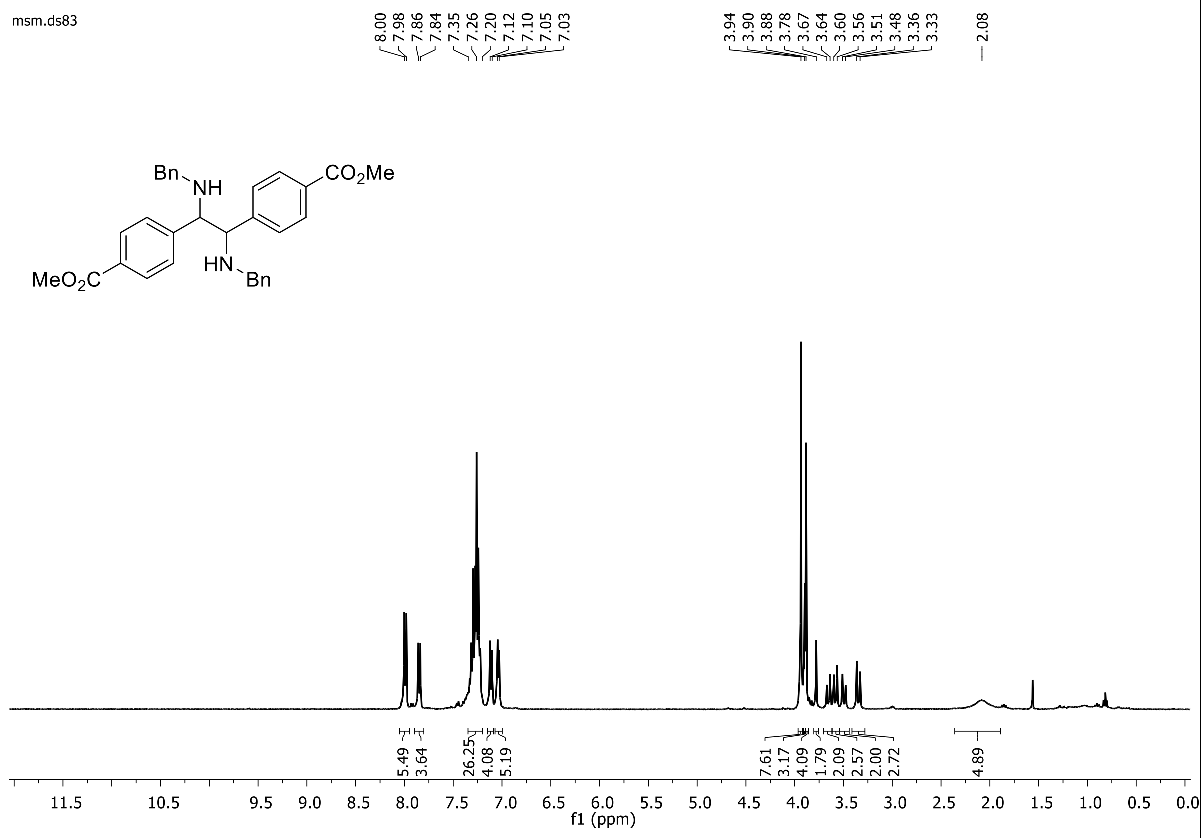

^{1}H (400 MHz) NMR of **9e** in $CDCl_3$

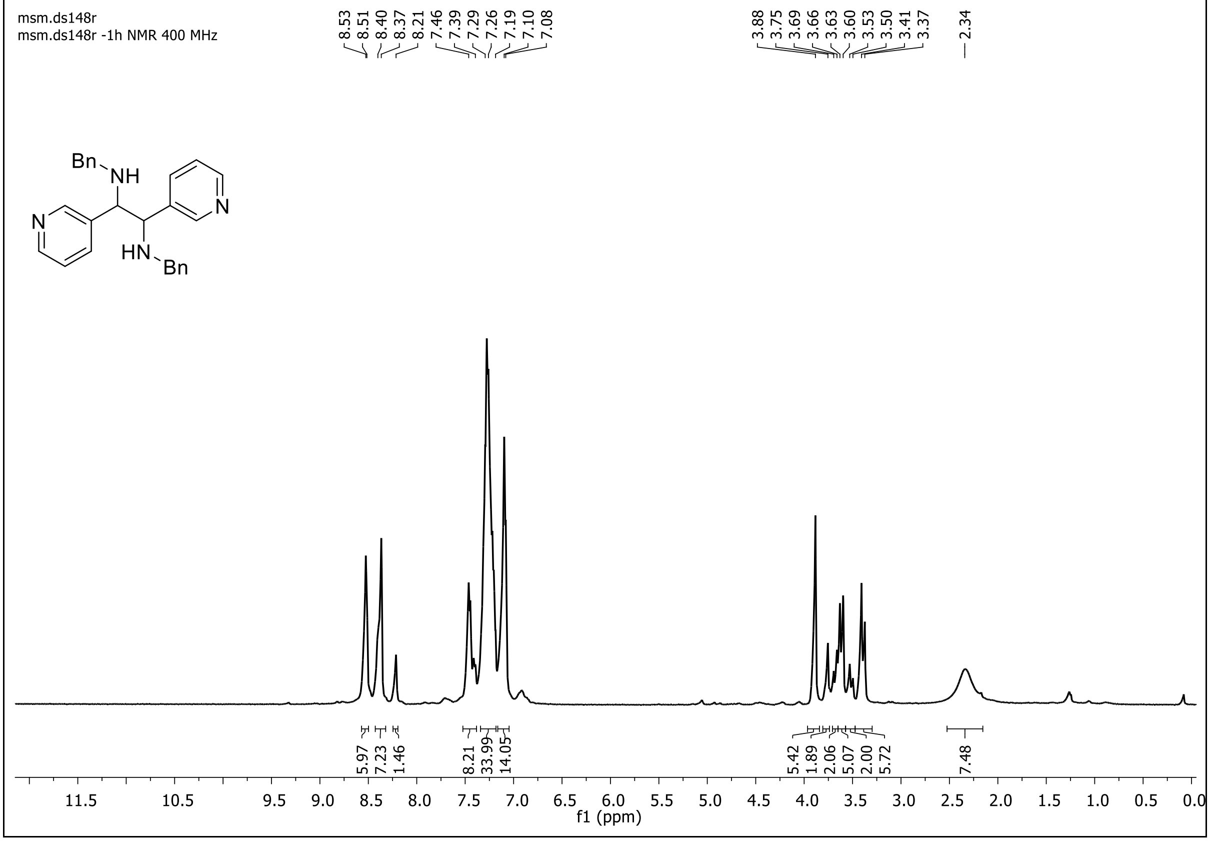

^{1}H (400 MHz) NMR of **9f** in $CDCl_3$

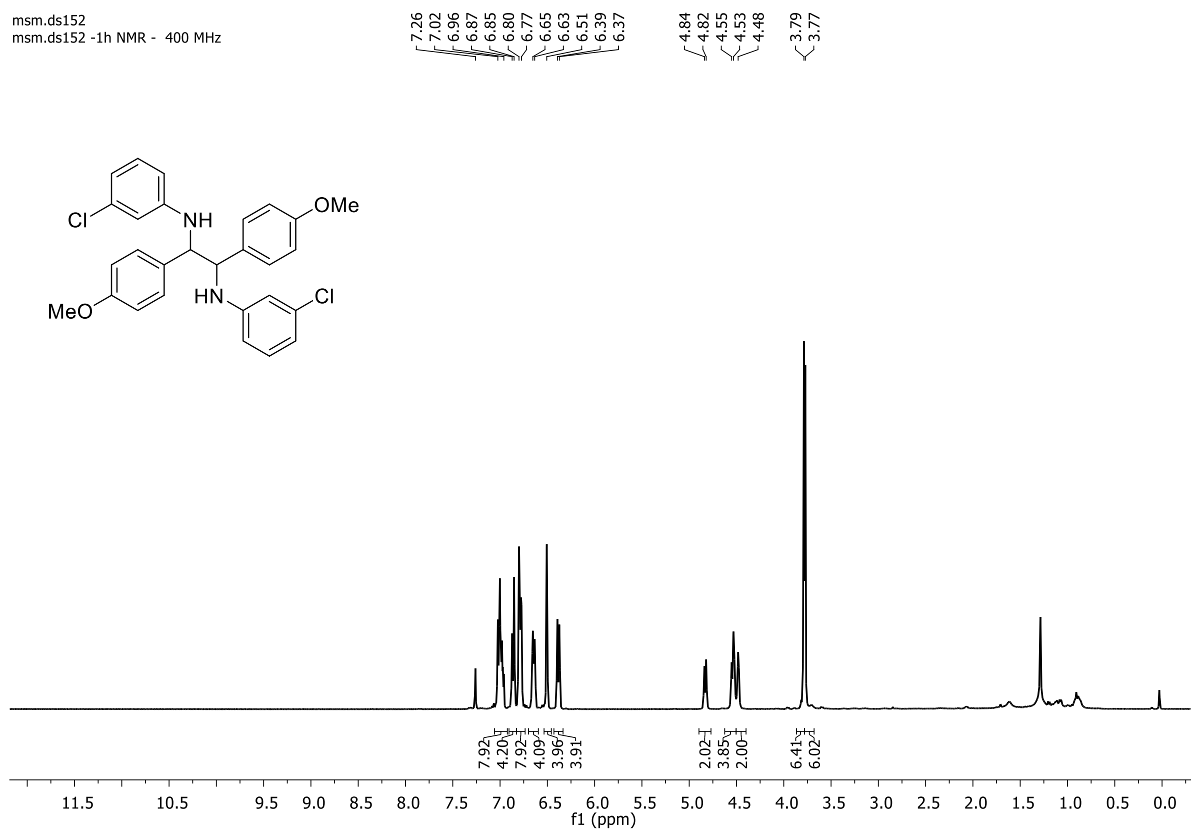

^{1}H (400 MHz) NMR of **12a** in $CDCl_3$

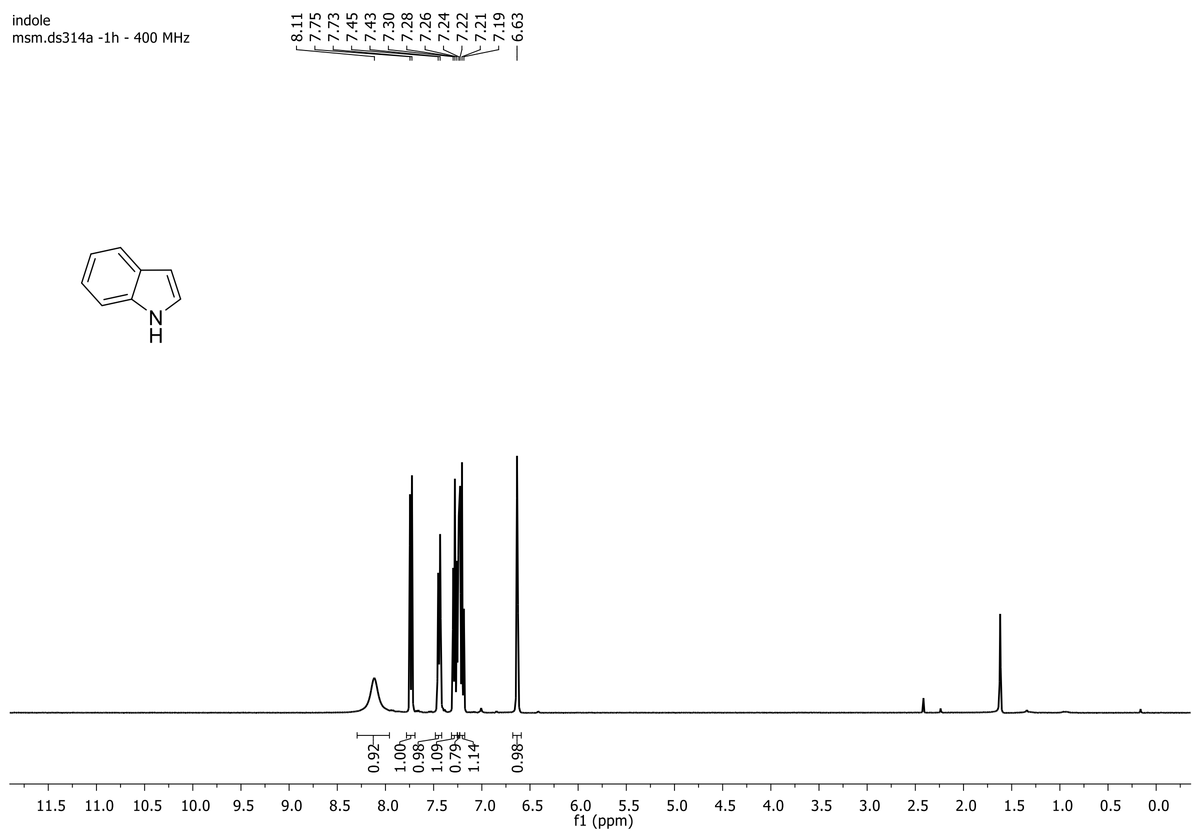

^{1}H (500 MHz) NMR of **12b** in $CDCl_3$

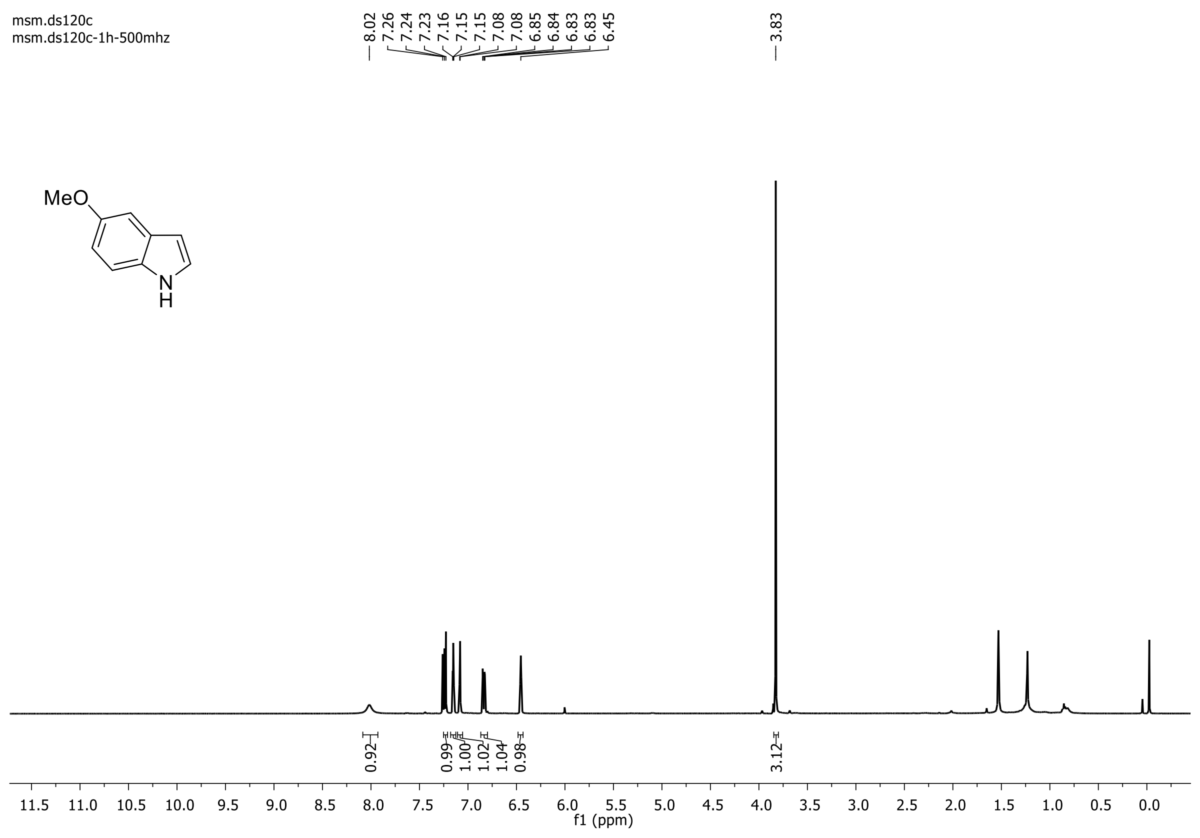

^{1}H (400 MHz) NMR of **12c** in $CDCl_3$

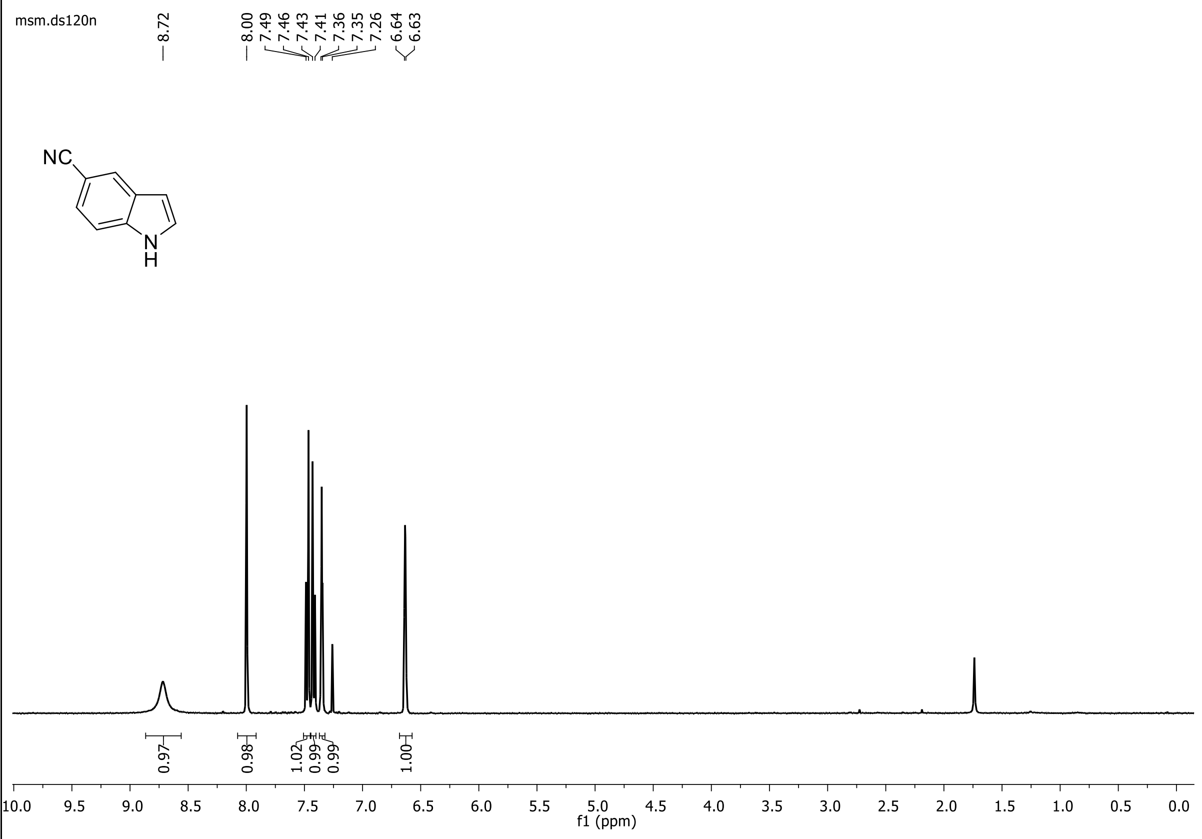

^{1}H (400 MHz) NMR of **12d** in $CDCl_3$

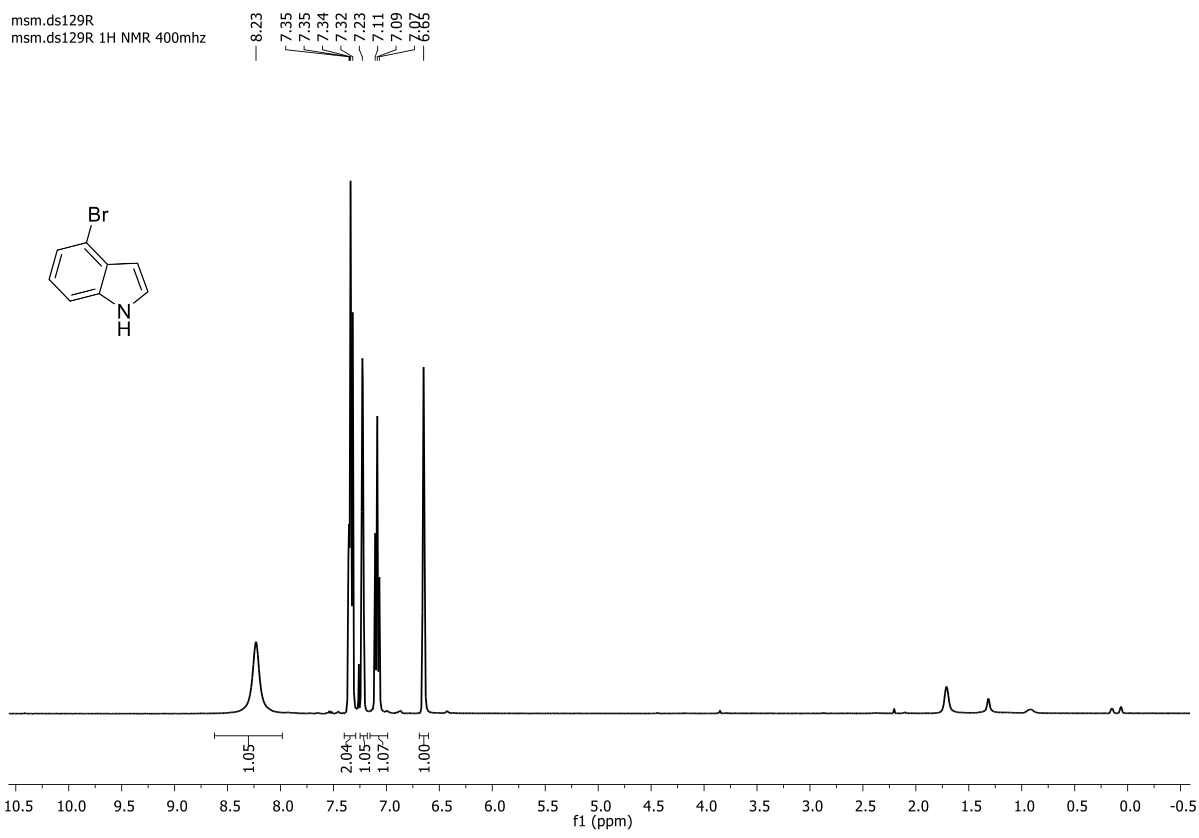

^{1}H (400 MHz) NMR of **12e** in $CDCl_3$

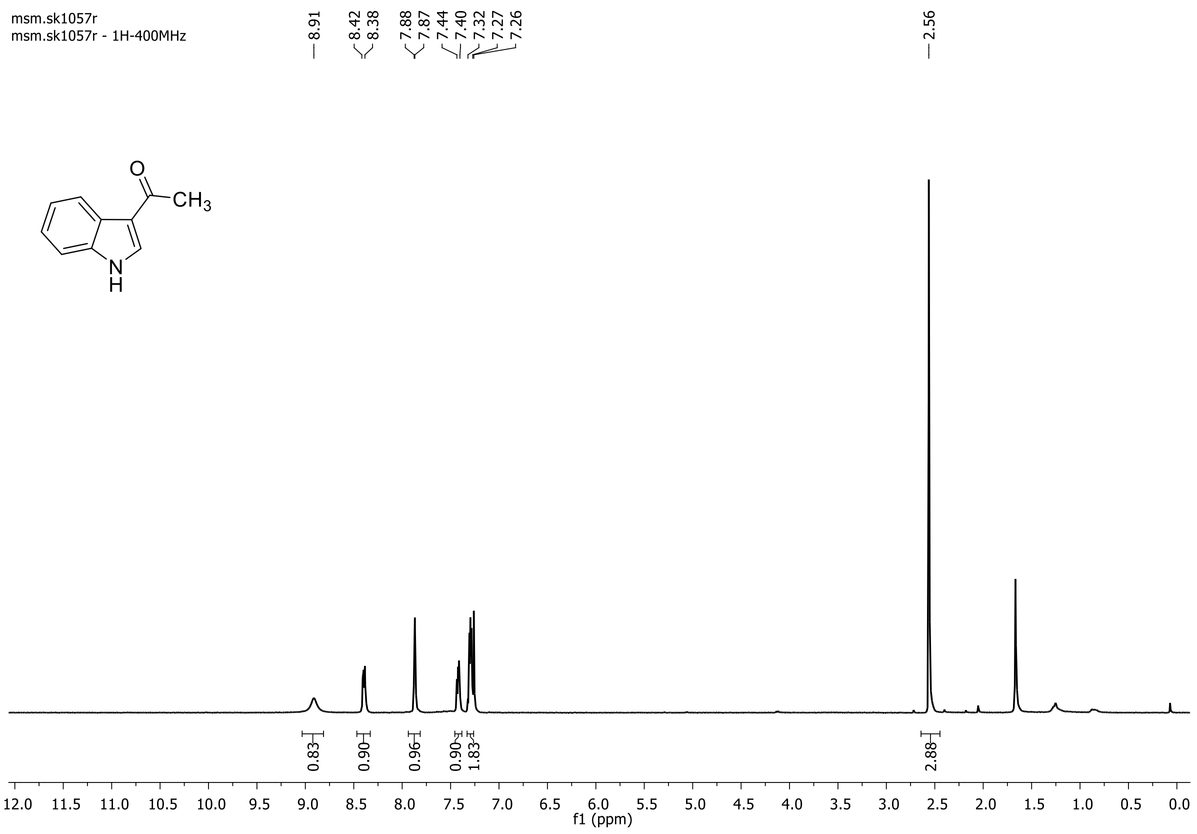

^{1}H (400 MHz) NMR of **12f** in DMSO-d_6

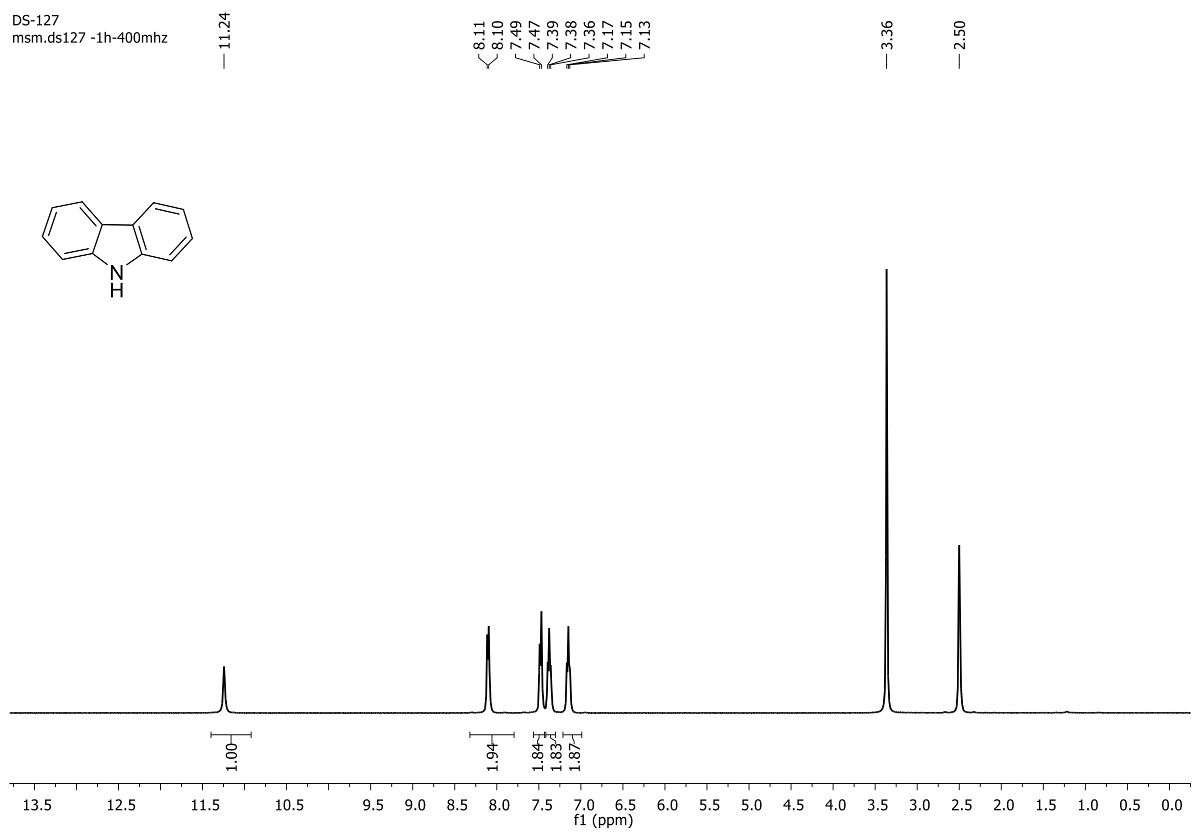

^{1}H (400 MHz) NMR of **12g** in $CDCl_3$

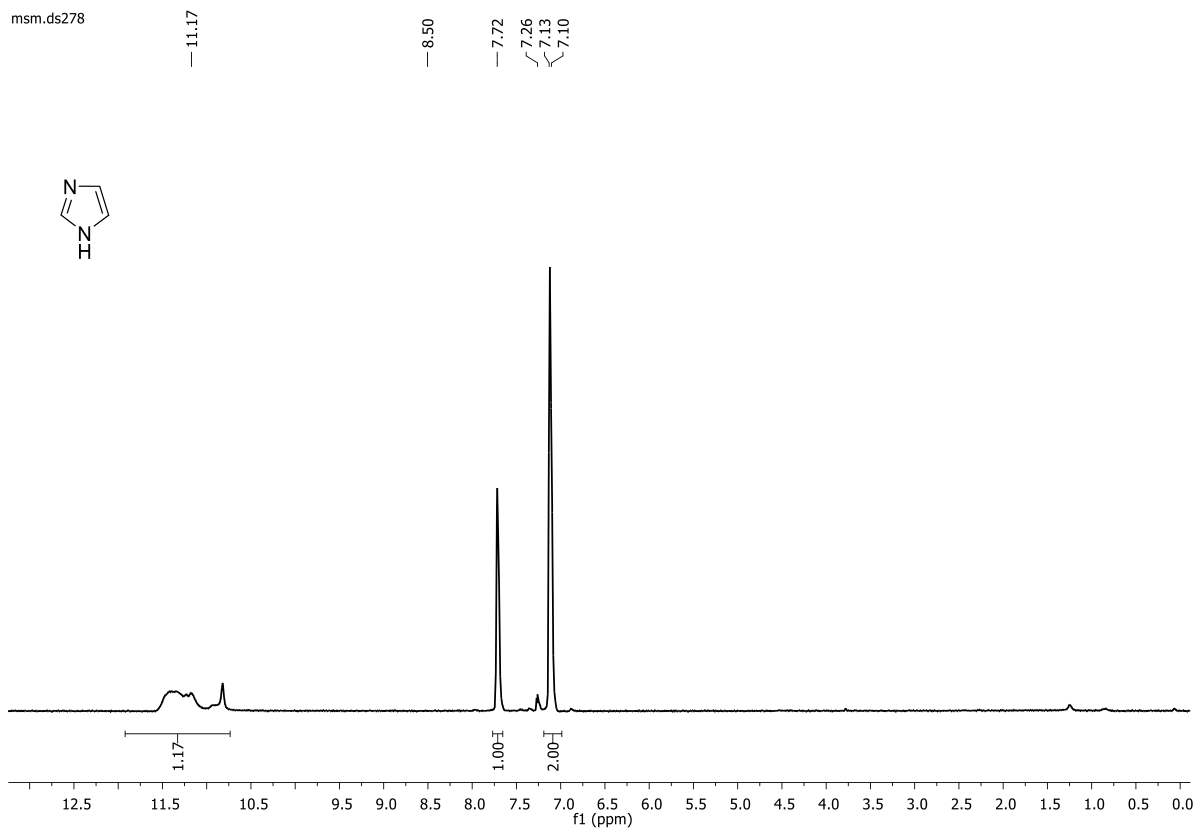

^{1}H (400 MHz) NMR of **12h** in $CDCl_3$

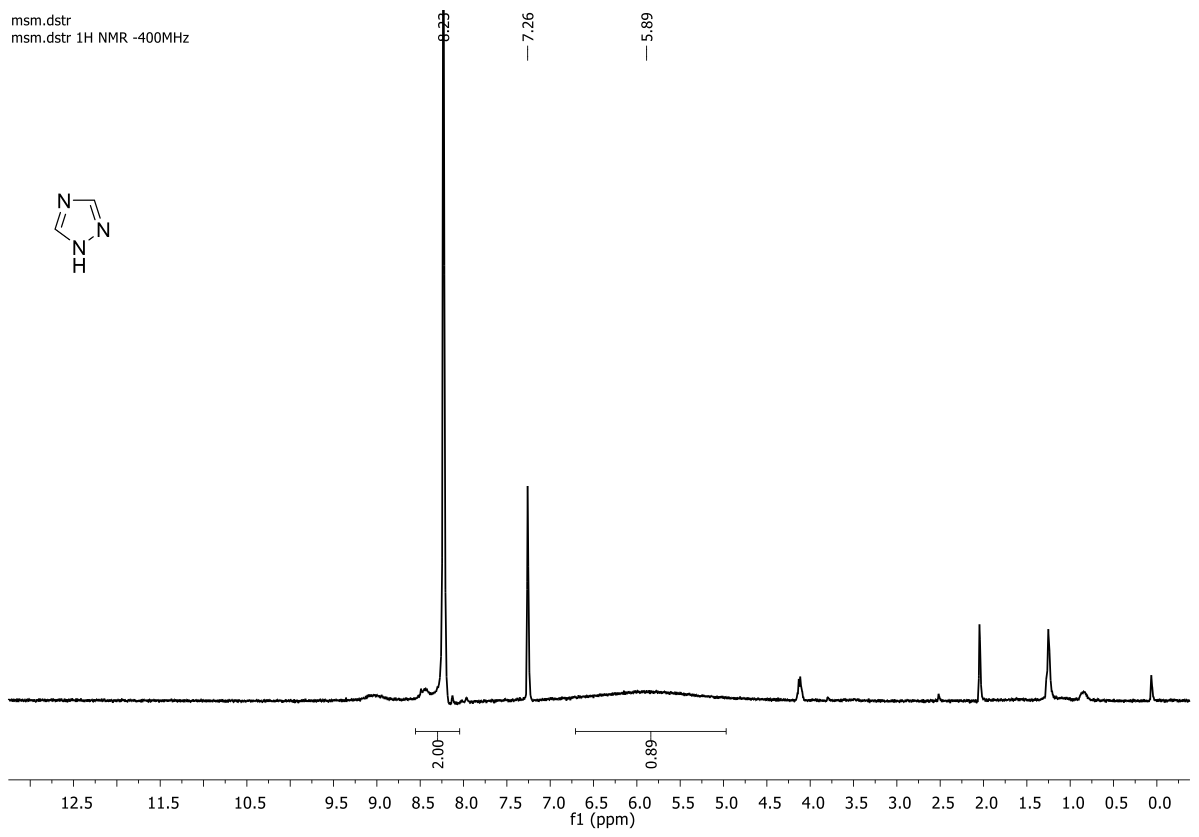

^{1}H (500 MHz) NMR of **12i** in $CDCl_3$

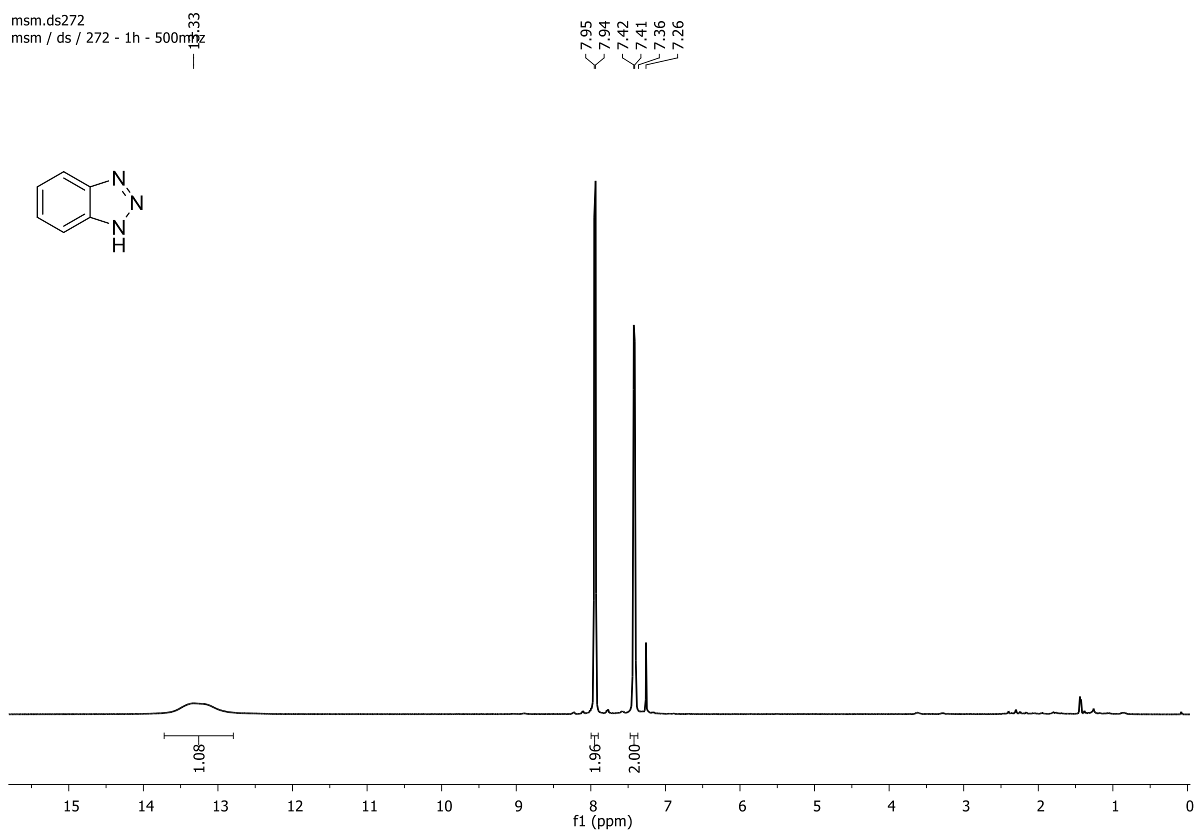

^{1}H (400 MHz) NMR of **12j** in $CDCl_3$

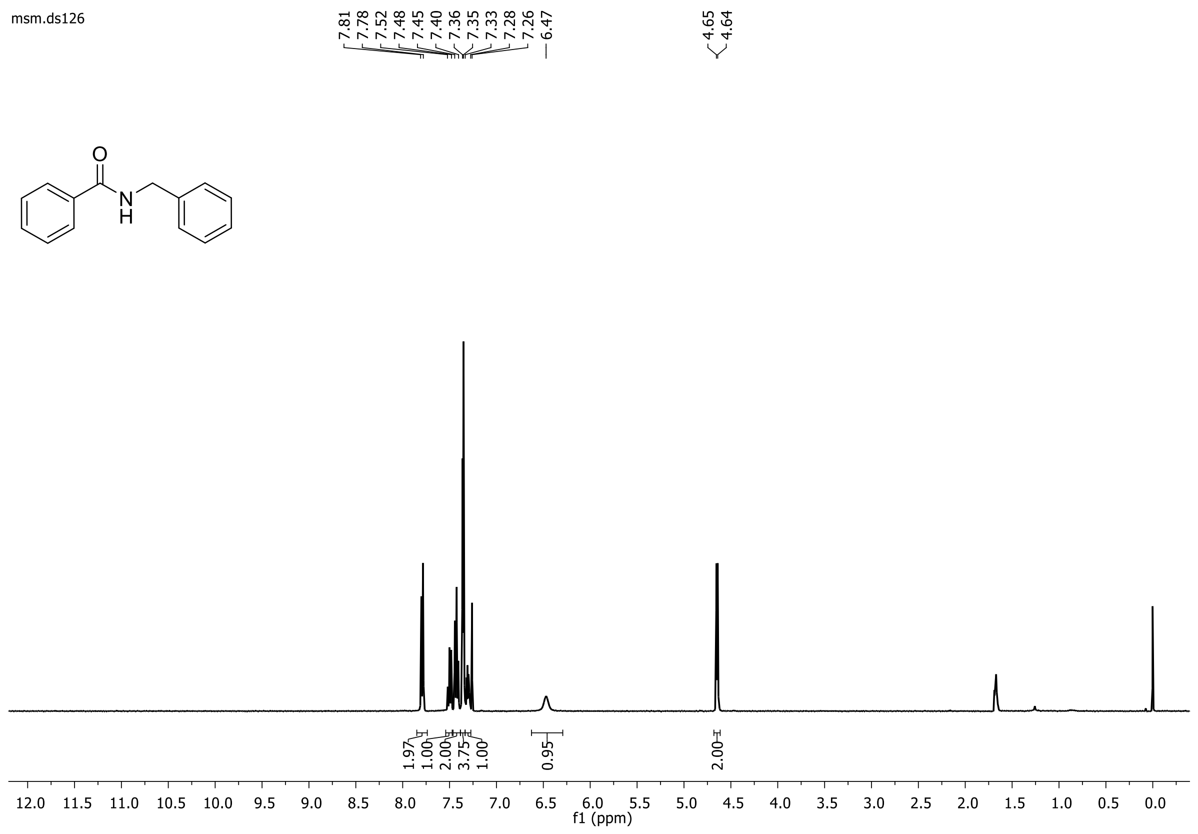

^{1}H (400 MHz) NMR of **12k** in $CDCl_3$

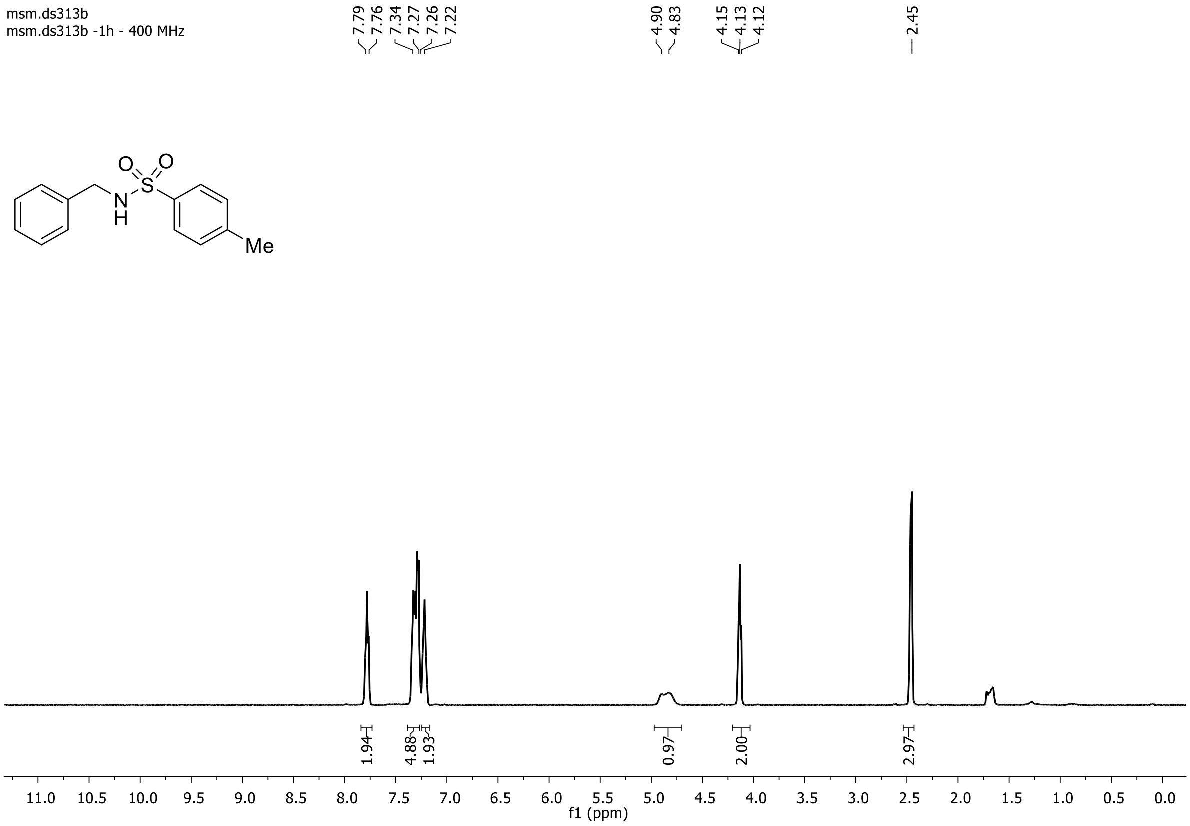

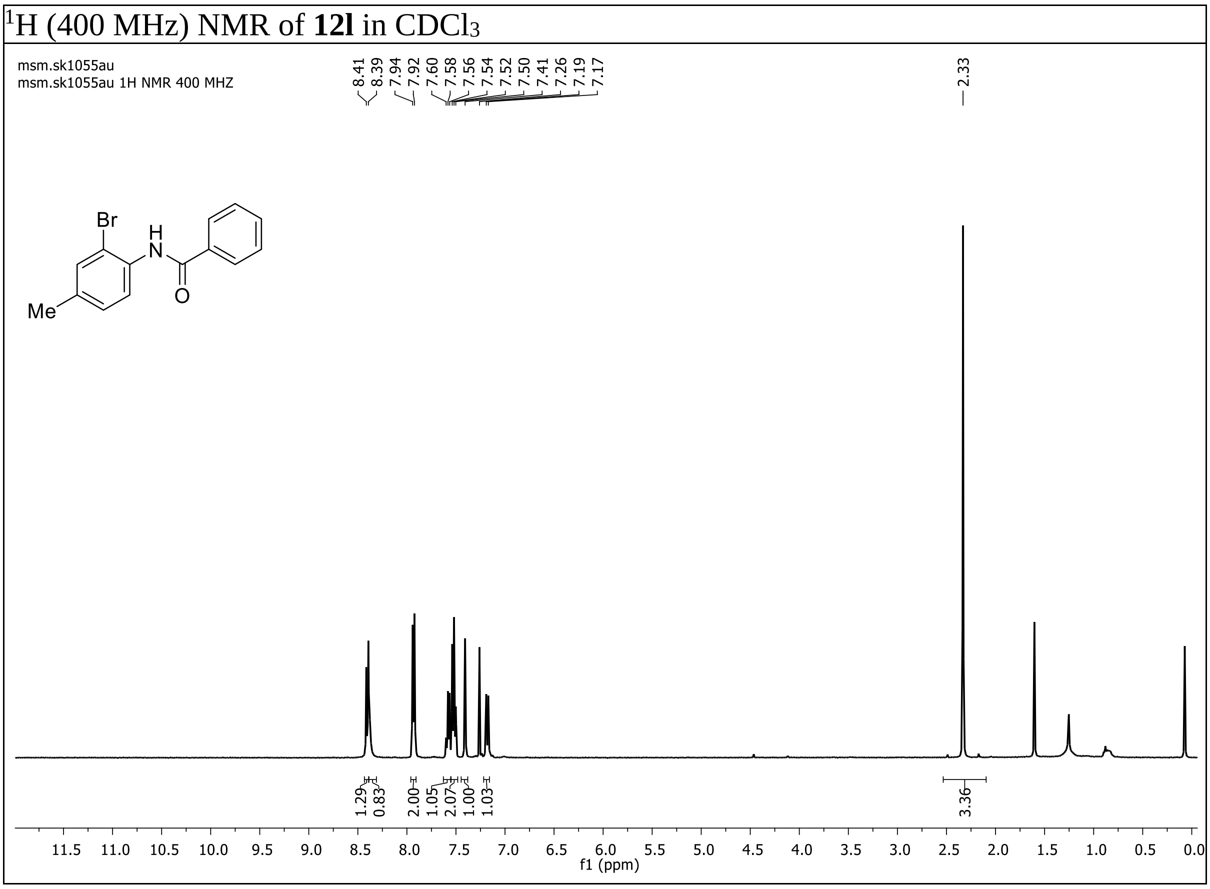
^{1}H (400 MHz) NMR of **12l** in CDCl3
msm.sk1055au
msm.sk1055au 1H NMR 400 MHZ
8.41
8.39
7.94
7.92
7.60
7.58
7.56
7.54
7.52
7.50
7.41
7.26
7.19
7.17
2.33
Br
H
N
O
Me
1.29
0.83
2.00
1.05
2.07
1.00
1.03
3.36
11.5
11.0
10.5
10.0
9.5
9.0
8.5
8.0
7.5
7.0
6.5
6.0
5.5
5.0
4.5
4.0
3.5
3.0
2.5
2.0
1.5
1.0
0.5
0.0
f1 (ppm)

^{1}H (400 MHz) NMR of **12m** in $CDCl_3$

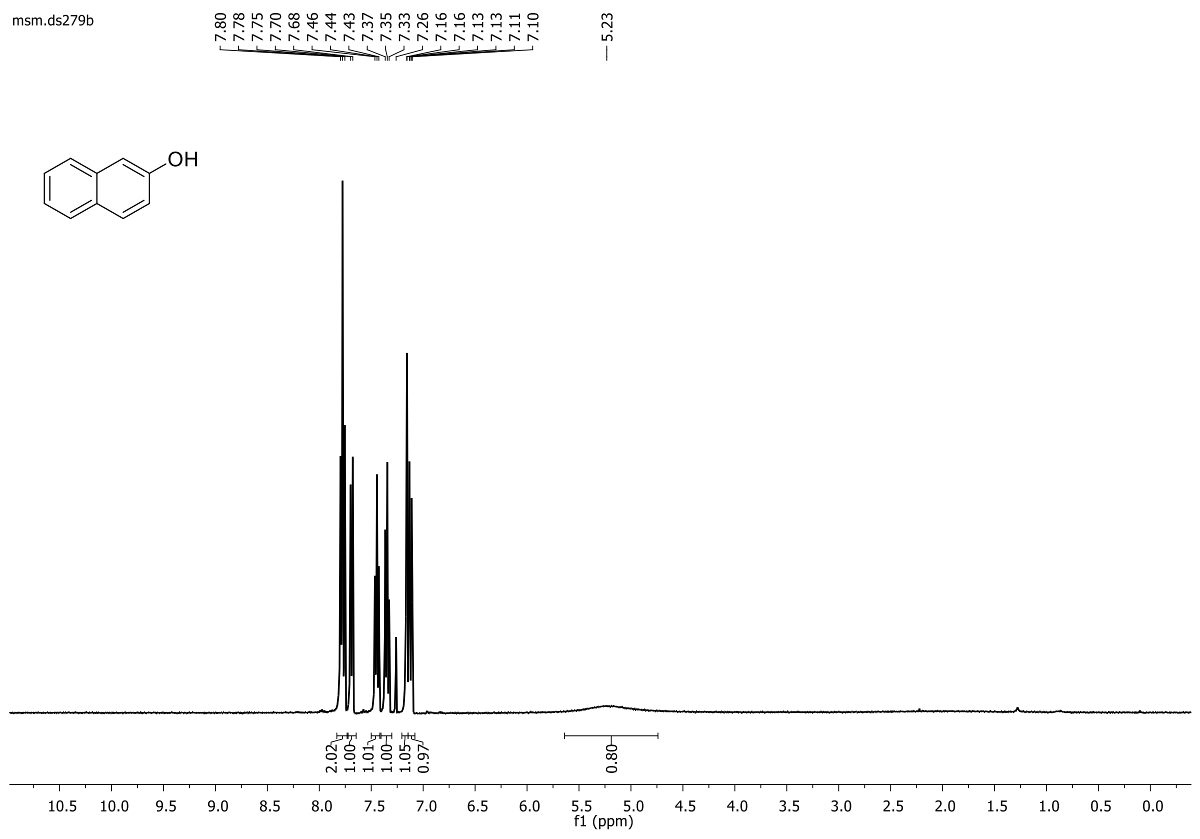

^{1}H (500 MHz) NMR of **13a** in DMSO-d_6

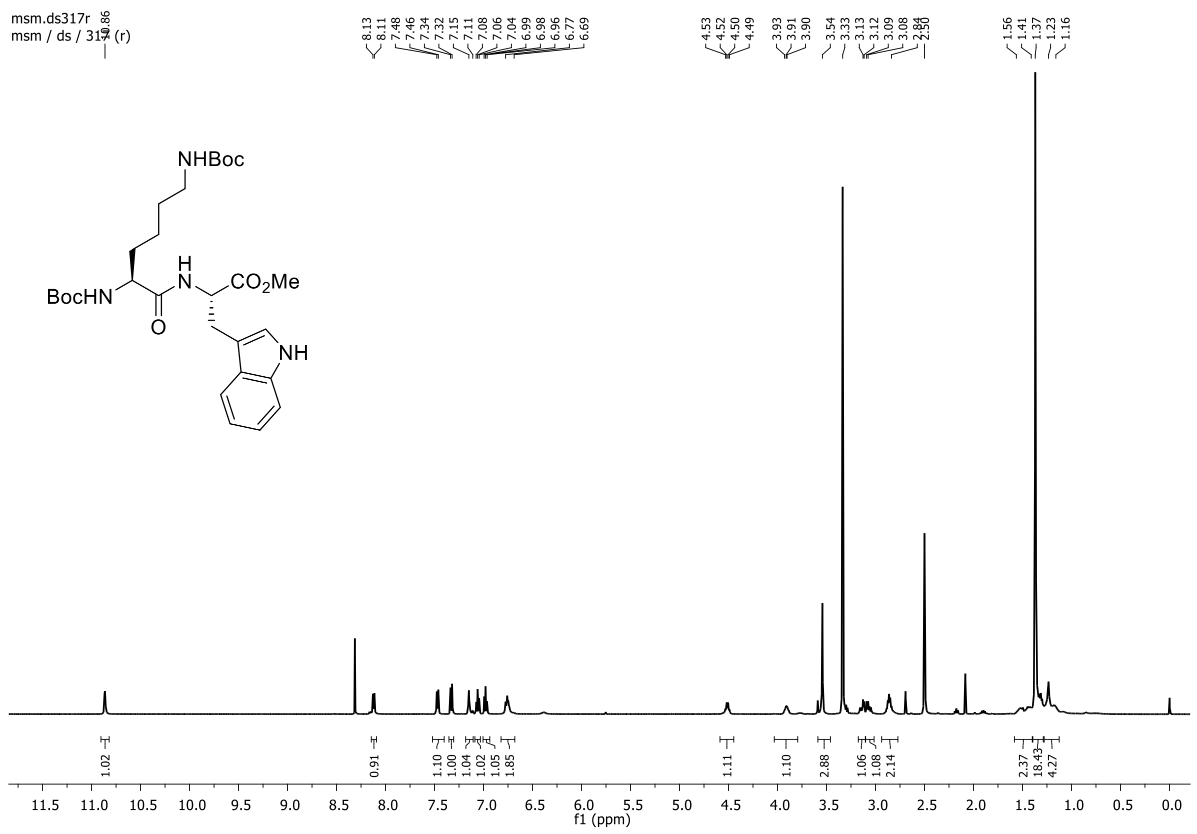

^{1}H (400 MHz) NMR of **13b** in DMSO-*d*$_6$

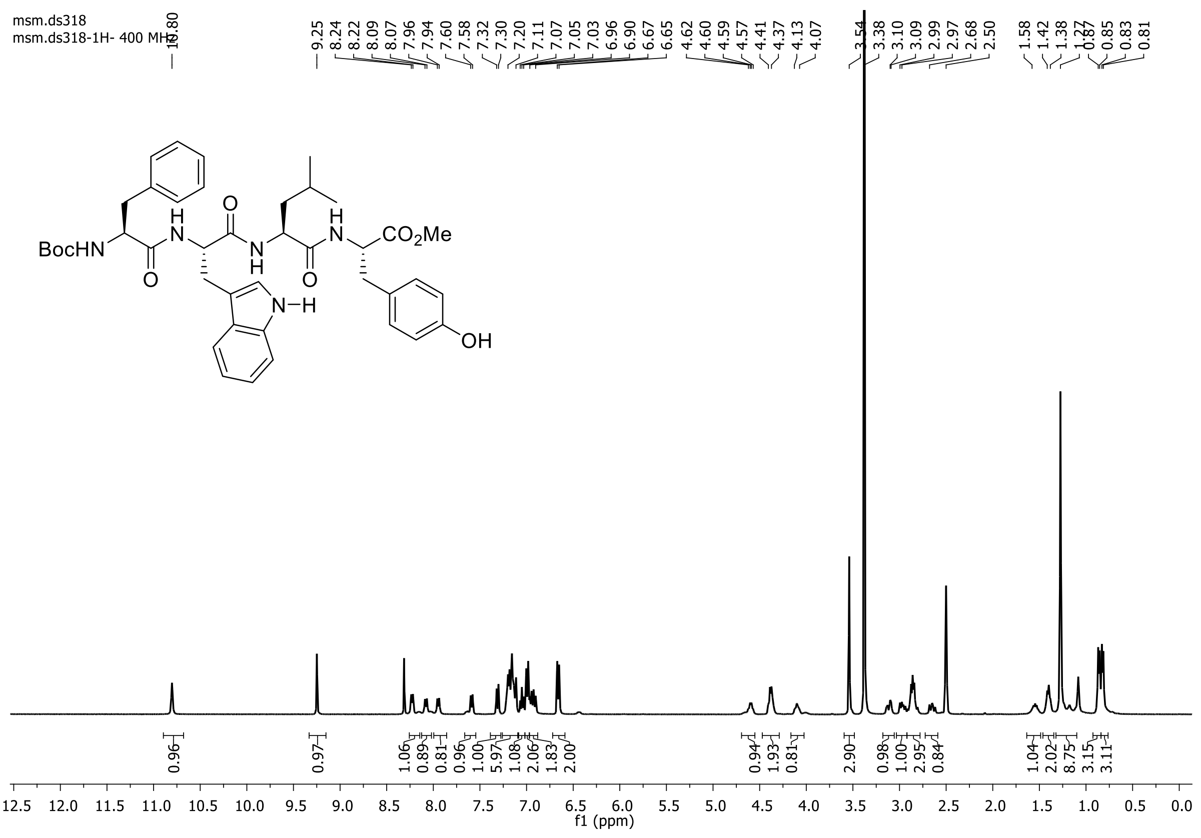

^{1}H (400 MHz) NMR of **13c** in DMSO-d_6

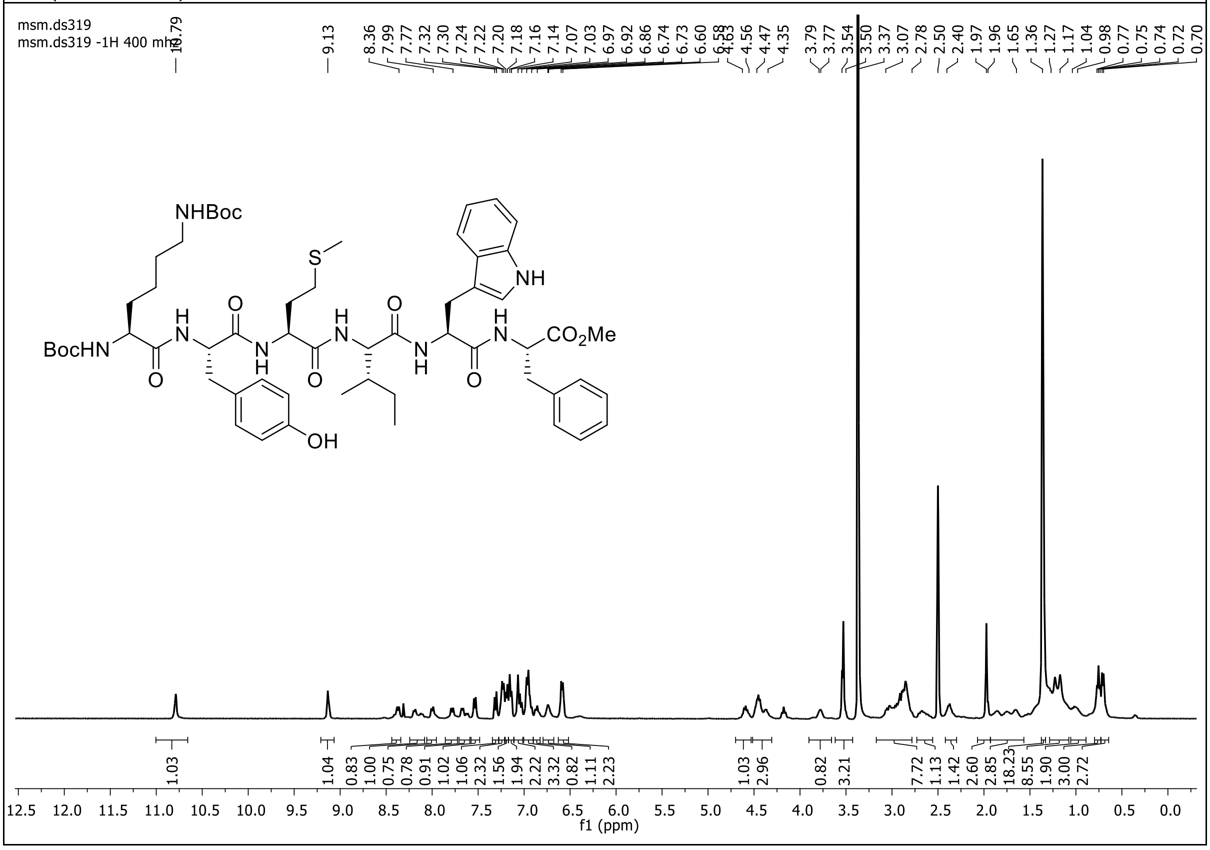

^{1}H (400 MHz) NMR of **13d** in DMSO-d_6

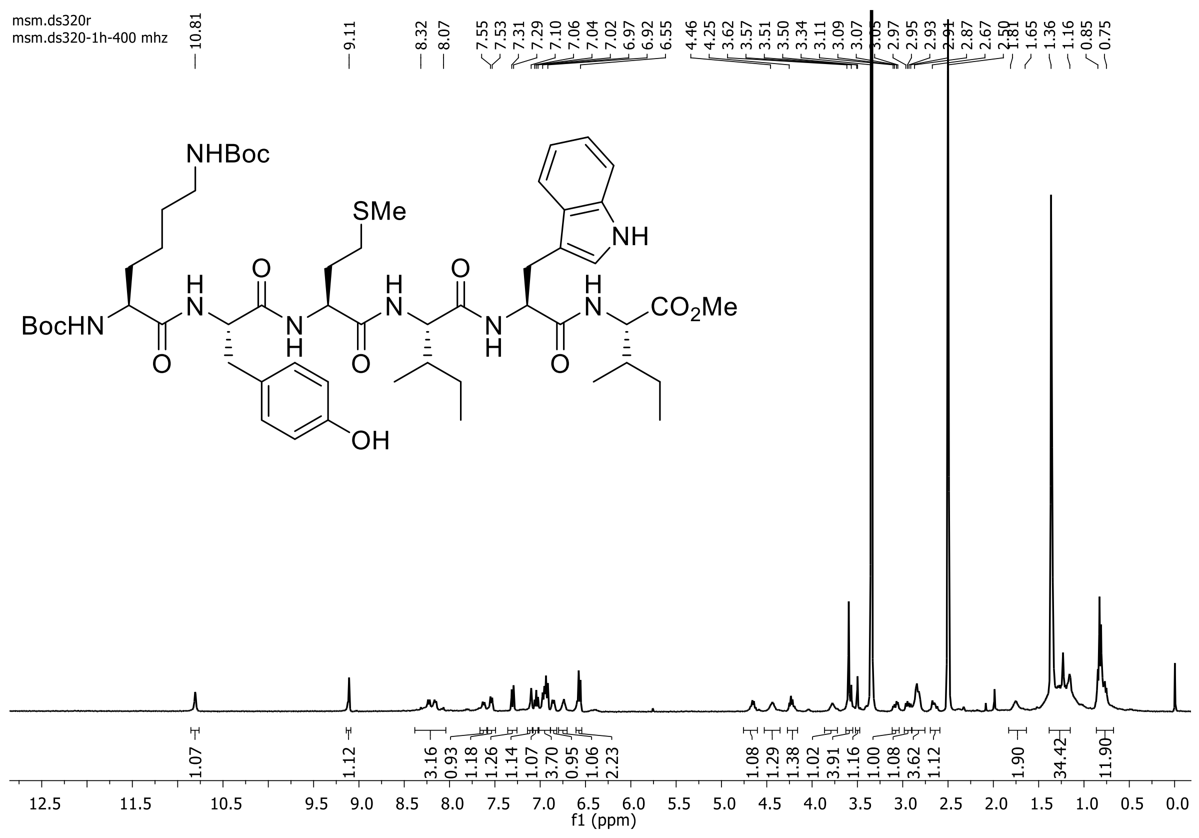

^{1}H (400 MHz) NMR of **13e** in DMSO-d_6

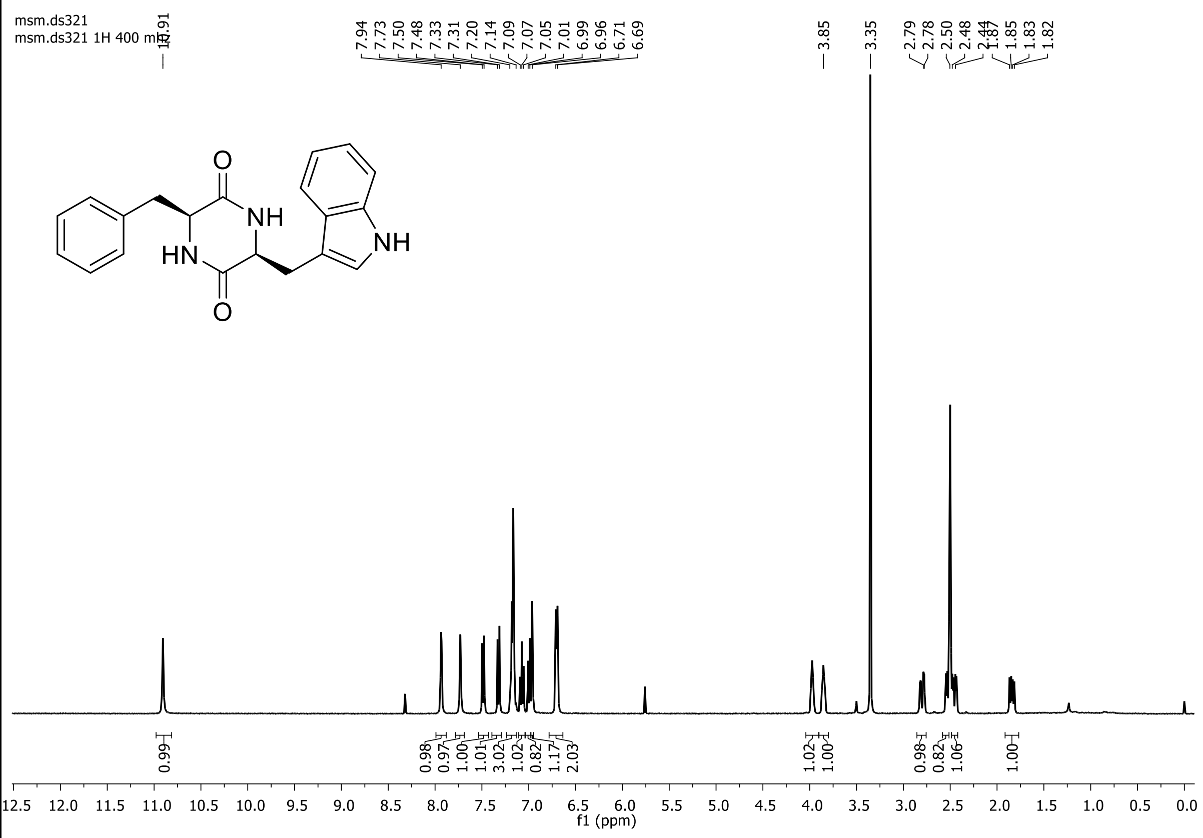

Supplement: SC-015-D4SC03438D-s001 [file SC-015-D4SC03438D-s001.pdf]
